# Triple energy transfer-enabled dearomative cycloaddition/rearrangement cascade of bicyclic azaarenes to structurally complex products

**DOI:** 10.1038/s41929-026-01566-z

**Published:** 2026-07-09

**Authors:** Preeti Chahar, Utpal Kundu, Subhabrata Dutta, Niklas Hölter, Florian Boser, Fabian M. Hugel, Luca Stadtmann, Constantin G. Daniliuc, Frank Glorius

**Affiliations:** Organisch-Chemisches Institut, Universität Münster, Münster, Germany

**Keywords:** Photocatalysis, Catalyst synthesis, Synthetic chemistry methodology

## Abstract

Pyridine-fused, C(*sp*^3^)-rich bridged frameworks are privileged scaffolds in natural products and pharmaceutical compounds, yet their synthesis remains a formidable challenge due to prevailing step-intensive routes. Energy transfer (EnT)-catalysed dearomative cycloadditions offer a milder approach to increase saturation in feedstock (hetero)arenes. Current methods exploiting the 1,4-biradical reactivity of bicyclic azaarenes mainly focus on [4+2] cycloadditions. However, achieving higher-order cycloadditions, such as [4+*n*] (*n* > 2), to construct pyridine-fused/bridged motifs, remains challenging. Here we report a cascade reaction combining a higher-order dearomative [5+4] photocycloaddition of bicyclic azaarenes and vinyl cyclopropanes with a formal [3,3]-sigmatropic rearrangement and a subsequent [2+2] photocycloaddition. This triple EnT-catalysed cascade provides a mild, one-pot protocol for efficiently accessing complex pyridine-fused/bridged pentacyclic motifs with excellent selectivity. Notably, the EnT-mediated formal [3,3]-sigmatropic rearrangement unveils a mechanistic paradigm in photochemical cascades. Mechanistic experiments and density functional theory studies support involvement of three sequential EnT steps, rationalizing the origin of regioselectivity and diastereoselectivity.

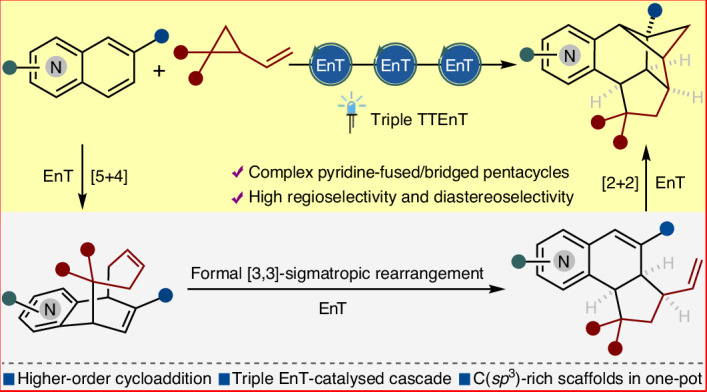

## Abstract (web summary)

Energy transfer (EnT) catalysed dearomative cycloadditions of bicyclic azaarenes are largely limited to [4+2] pathways, with higher-order cycloadditions being difficult to achieve. Now, the authors circumvent this limitation by establishing a higher-order cycloaddition followed by cascades. This triple EnT-enabled [5+4]/[3,3]/[2+2] cascade with vinyl cyclopropanes enables one-pot access to complex pyridine-fused, bridged pentacycles scaffolds that are widely embedded in bioactive molecules, but whose synthesis has remained challenging owing to scarcity of efficient strategies.

## Main

**Fig. 1:**
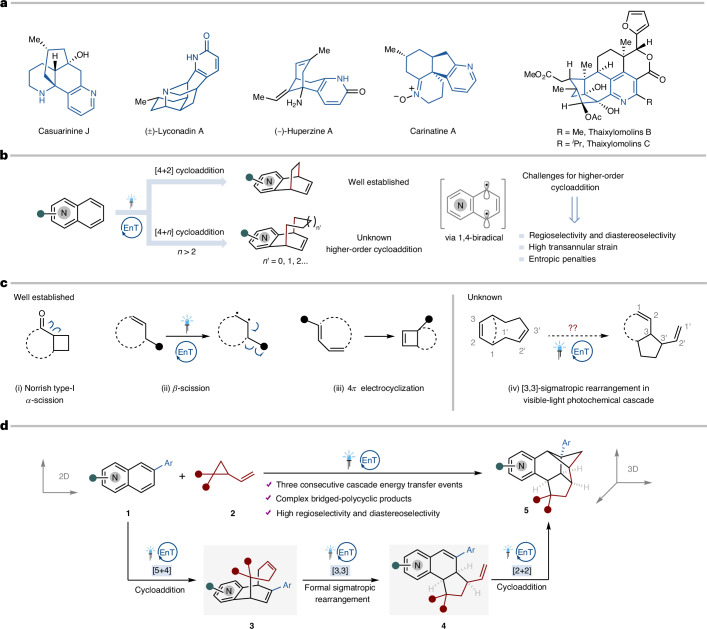
Cascade EnT-catalysed DAC for the synthesis of bridged/fused pentacyclic scaffolds. **a**, Representative motifs in bioactive molecules. **b**, State-of-the-art: known EnT-mediated [4+2] intermolecular dearomative cycloaddition and underexplored [4+*n*] variants of bicyclic azaarenes. **c**, Conceptual framework of sequential photochemical cascade transformations. **d**, This work: EnT-enabled cascade dearomative [5+4] cycloaddition/formal [3,3]-sigmatropic rearrangement/[2+2] cycloaddition of bicyclic azaarenes.

C(*sp*^3^)-rich molecular architectures are increasingly sought after in drug discovery owing to their favourable physiochemical properties such as enhanced solubility, metabolic stability and high lipophilicity^1–3^. In particular, pyridine-fused/bridged C(*sp*^3^)-rich motifs are prevalent in a plethora of bioactive molecules^4–9^ (Fig. 1a). The omnipresence of such privileged scaffolds warrants the development of general, mild and efficient synthetic methods. Photochemical dearomative cycloadditions (DACs) serve as a straightforward approach for constructing such frameworks directly from readily available bicyclic azaarenes (for example, quinolines, isoquinolines and quinazolines)^10–13^. However, traditional methods rely on direct ultraviolet (UV) irradiation for substrate activation, they often suffer from competing side reactions and poor chemoselectivity or regioselectivity issues^14–16^. Conversely, visible light-mediated energy transfer (EnT) catalysis has emerged as a milder, modular strategy for such DACs that effectively mitigates undesired cycloreversion pathways^17–27^. Recently, our group pioneered an EnT-catalysed highly regioselective and diastereoselective intermolecular [4+2] DAC reaction of bicyclic azaarenes with alkenes to forge bridged polycycles^28^. The transformation proceeds via triplet excitation of arenes to form a 1,4-biradical species that undergoes formal [4+2] cycloaddition with a range of alkenes^29–31^. The success of this reaction hinges on the judicious choice of Brønsted/Lewis acid additives or hydrogen bonding donor solvents, which lower the triplet energy, facilitating a more efficient triplet–triplet energy transfer (TEnT) event. Subsequently, the [4+2] DAC reactions of naphthalenes were independently reported by research groups in refs. ^32–39^, giving access to complex three-dimensional scaffolds.

Despite these advances, EnT-driven DACs have largely been confined to [4+2] cycloadditions, leveraging the 4*π* component of bicyclic azaarenes and naphthalenes. Expanding this repertoire to higher-order cycloadditions [4+*n*] (*n* > 2), such as [5+4], offers an exciting avenue to access (hetero)arene-fused medium-sized, bridged-ring systems with increased molecular complexity. Nevertheless, such endeavours remain underdeveloped owing to formidable intrinsic challenges such as transannular strain and entropic penalties that limit the key cyclization steps^40,41^ (Fig. 1b). We wondered whether highly strained vinyl cyclopropanes (VCPs) could be used as versatile five-carbon synthons in DAC with bicyclic azaarenes^42^. Successful implementation of this notion would provide a streamlined route to pyridine-fused C(*sp*^3^)-rich bridged systems. In this setting, intramolecular [5+4] and [5+2] cycloaddition of VCPs tethered with naphthalene and indole derivatives have been reported^42,43^. However, the intramolecular nature of these methodologies imposes substantial limitations, as they require tedious synthesis of prefunctionalized substrates with low step economy, which inherently restricts the generality of the reaction. Notably, an intermolecular approach, enabling the fusion of two distinct molecular fragments in a modular way, offers untapped potential for building complexity from readily available building blocks.

Here we show that under visible light-mediated EnT catalysis, bicyclic azaarenes could indeed undergo intermolecular [5+4] DAC with VCPs, leading to a pyridine-fused nine-membered bridged-ring system **3** (Fig. 1d). We discovered that the resulting alkene units in the intermediate **3** could rapidly undergo further cascade EnT events, comprising a formal [3,3]-sigmatropic rearrangement and a subsequent [2+2] cycloaddition, giving a highly complex bridged pentacyclic product. Of note, cascade reactions have proved invaluable in rapidly constructing molecular complexity via a well-orchestrated sequence of multiple bond-breaking and bond-making events in a single operation, greatly minimizing the synthetic efforts^44,45^. Nature exemplifies this strategy by masterfully executing catalytic cascade reactions to assemble multiple complex natural products with high efficiency^32^. Emulating this blueprint, visible light-driven EnT catalysis, with its mild and tunable reactivity, possesses great potential for enabling precisely controlled cascade transformations^46,47^. While key mechanistic events such as Norrish type-I *α*-scission, *β*-scission and 4*π* electrocyclization have been exploited in photochemical cascade reactions to selectively generate molecular complexity^32,48–51^, the formal [3,3]-sigmatropic rearrangement has, so far, remained unrealized in EnT-catalysed cascade process (Fig. 1c). Moreover, this work highlights an EnT-enabled [3,3]-sigmatropic rearrangement, which complements the conventional thermally allowed pericyclic paradigm dictated by orbital symmetry (Woodward–Hoffmann rules), unlocking access to uncharted chemical space^52–54^. In summary, we report a triple (TTEnT)-enabled intermolecular dearomative [5+4] cycloaddition/[3,3]-sigmatropic rearrangement/[2+2] cycloaddition of bicyclic azaarenes with VCPs. This cascade process harnesses several EnT steps without requiring any external reagents to furnish pyridine-fused/ bridged pentacyclic scaffolds **5** (Fig. 1d) with distinct ring topologies, high regioselectivity and stereoselectivity.

## Results

### Reaction development

**Fig. 2:**
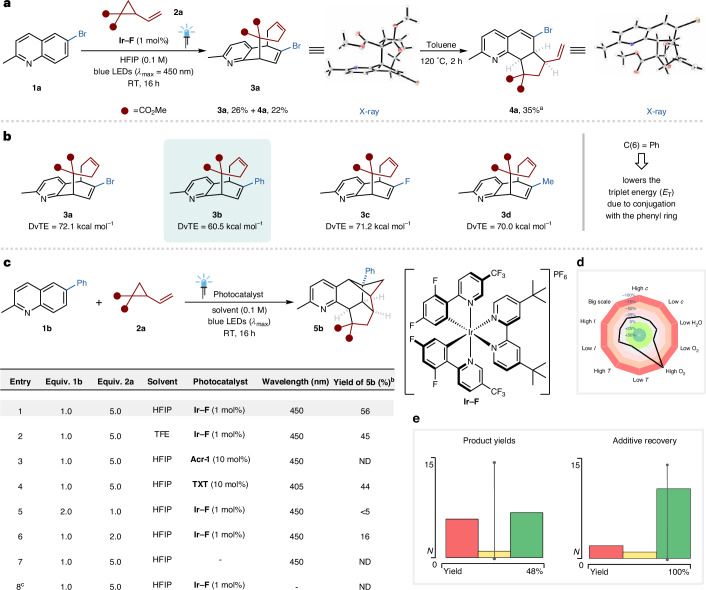
Reaction development and optimization studies. **a**, Reaction development. **b**, DvTE of [5+4] cycloadducts with different substituents at the 6-position. **c**, Reaction optimization. **d**, Reaction conditions-based sensitivity screening. **e**, Additive-based robustness assessment. Crude ^1^H NMR yields are provided. ^a^NMR yield was determined with respect to **1a** over two steps. ^b^Reactions were performed on 0.1 mmol scale, analysed by ^1^H NMR of the crude reaction mixture using CH_2_Br_2_ as an internal standard. ^c^No light. *c*, concentration; *I*, intensity; ND, not detected; RT, room temperature; *T*, temperature; TFE, 2,2,2-trifluoroethanol.

Our preliminary investigations started with the reaction of quinoline **1a** and VCP **2a**, using [Ir(dF(CF_3_)ppy)_2_(dtbbpy)][PF_6_] (**Ir–F**, *E*_T_ = 61.8 kcal mol^−1^)^17^ as the photosensitizer under blue light-emitting diode (LED) irradiation (*λ*_max_ = 450 nm) using 1,1,1,3,3,3-hexafluoro-2-propanol (HFIP) as a solvent. Under these conditions, a mixture of [5 + 4] cycloadduct **3a** and initially unexpected product **4a** was obtained (Fig. 2a). Notably, this mixture of **3a** and **4a** could be completely converted into the [3,3]-sigmatropic rearrangement product **4a** on heating in toluene at 120 °C. We then explored the possibility of carrying [3,3]-rearrangement photochemically via an EnT pathway in a single pot^55^. However, a crucial challenge was the rational design of the substrate such that the triplet energy of the [5+4] cycloadduct would be appropriately matched with excited state energy of the photocatalyst. In addressing this challenge, the dynamic-vertical triplet energies (DvTE)^56^ of [5+4] intermediates containing different substitution at the 6-position were assessed **3a**–**3d** (Fig. 2b). These results revealed that the introduction of an aromatic substituent at the 6-position lowered the triplet state notably compared with other substituents (**3b**), rendering it a viable substrate for the subsequent rearrangement under EnT conditions. Structural analysis of the [3,3]-rearranged intermediate **4b** showed that, again, a conjugated and potentially excitable alkene unit is obtained in close spatial proximity to the terminal alkene, which opens up the possibility of a subsequent [2+2] photocycloaddition, resulting in even more complex bridged pentacyclic products.

Motivated by the goal of achieving this cascade transformation, quinoline **1b** and VCP **2a** were subjected to the same reaction conditions. This led to the formation of a single isomer **5b** in 56% yield, featuring a complex bridged pentacyclic scaffold (Fig. 2c, entry 1). This represents a cascade reaction, which is consistent with the initial [5+4] intermolecular cycloaddition followed by a formal [3,3]-sigmatropic rearrangement and intramolecular [2+2] cycloaddition. During the photocatalyst screening, no product formation was observed on using a highly oxidizing [Mes-Acr-Mes][ClO_4_] (**Acr-1**) photocatalyst^57^ (entry 3). Thioxanthone (**TXT**, *E*_T_ = 65.5 kcal mol^−1^)^11^, a frequently used organophotocatalyst in EnT catalysis, produced lower yields (entry 4). Variation in the equivalence of the substrates did not improve reactivity (entries 5 and 6). Control experiments revealed that both visible light and photosensitizer were required for the transformation (entries 7 and 8). Condition-based sensitivity assessment^58^ confirmed the robustness of the reaction against variation in irradiance and temperature (Fig. 2d), whereas the presence of molecular oxygen was found to markedly supress the reaction efficiency.

Functional group tolerance was investigated through an additive-based screening^59^. The reaction exhibited broad compatibility with unprotected alcohols, aldehydes, amides and other functional groups (Fig. 2e and Supplementary Table 2). By contrast, basic amine-containing substrates such as anilines effectively suppressed product formation.

### Mechanistic studies

**Fig. 3:**
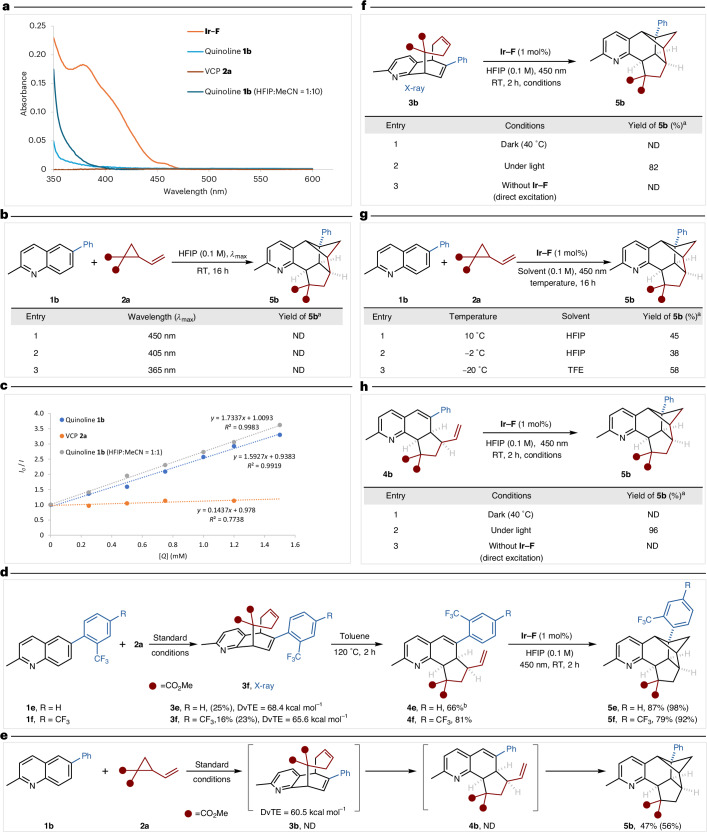
Mechanistic investigations. **a**, UV-visible absorption spectroscopy analysis. **b**, Direct excitation studies. **c**, Stern–Volmer luminescence quenching. **d**, Intermediate isolation involved in this cascade process. **e**, Optimized reaction condition with the complete pathway. **f**, Evidence of the EnT-catalysed formal [3,3]-sigmatropic shift. **g**, Low-temperature studies for supporting the photochemical pathway. **h**, Evidence of EnT-catalysed [2+2] cycloaddition. Yields of isolated compounds are provided. Crude ^1^H NMR yields are given in parentheses unless otherwise mentioned. ^a^Reactions were performed on 0.1 mmol scale, analysed by ^1^H NMR of the crude reaction mixture using CH_2_Br_2_ as an internal standard. ^b^Yield of **4e** was calculated with respect to NMR yield of **3e**. See Supplementary Information for X-ray data.

A series of mechanistic studies was conducted to determine the reaction pathway. UV-visible absorption analysis (Fig. 3a) revealed that **Ir–F** is the exclusive absorbing species near the irradiating wavelength of the reaction (*λ*_max_ = 450 nm), thereby eliminating the possibility of direct excitation of either **1b** or **2a**. Furthermore, when the reaction was investigated in absence of photocatalyst, no targeted product **5b** was detected under irradiation at *λ*_max_ = 365 nm, 405 nm and 450 nm (Fig. 3b). Next, cyclic voltammetry studies were performed for substrates **1b** and **2a**. VCP **2a** did not display any redox activity. Whereas **1b** showed an irreversible oxidation at *E*_1/2_ = +1.67 V versus the saturated calomel electrode (SCE), which lies outside the redox potential window of **Ir–F** (*E*_1/2_ PC^·^^+^/PC* = −0.89 V versus SCE and *E*_1/2_ PC*/PC^·^^−^ = +1.21 V versus SCE, where PC denotes the photocatalyst)^57^, thereby ruling out the putative photoredox mechanism (Supplementary Fig. 10). Stern–Volmer luminescence quenching studies (Fig. 3c) unveiled that **1b** is the sole quencher of the photocatalyst **Ir–F**. Quenching is slightly more efficient in the presence of HFIP than **1b** alone. This result is consistent with our previous studies that ground-state quinolines can be activated through a Brønsted/Lewis acid coordination—specifically by HFIP in this case, which facilitates EnT by lowering the triplet energy and the barrier to the radical addition^28^. Furthermore, the reaction quantum yield was experimentally determined as *Φ* = 0.11, rendering a radical chain mechanism unlikely (see Supplementary Information for details). Trapping experiments concluded that adding radical scavengers such as 2,2,6,6-tetramethyl-1-piperidinyloxy (TEMPO) or 2,6-di-*tert*-butyl-4-methylphenol (BHT) shuts down the reaction completely, indicating a radical-mediated pathway. The observed regioselectivity of the [5+4] cycloaddition step is consistent with our previous studies^28^ and appears in accordance with the spin density analysis of ^**3**^**1b** (Supplementary Fig. 11).

**Fig. 4:**
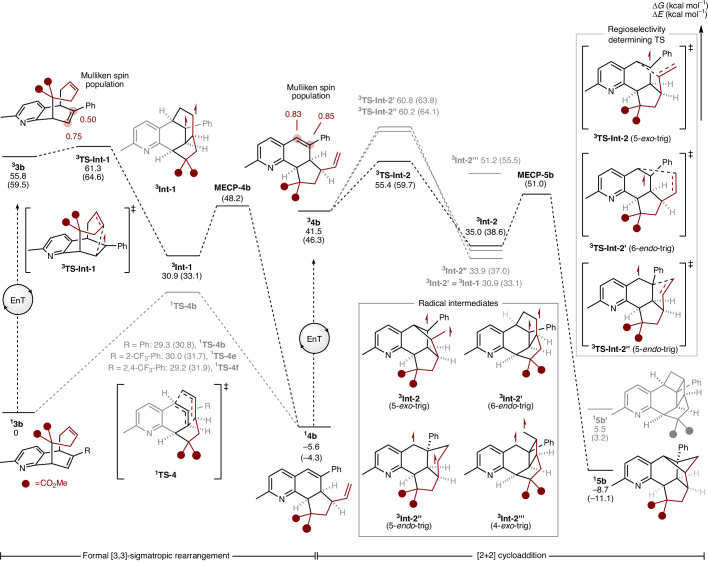
Computed energy profile. Density functional theory-computed energy profile for the reaction coordinate showing the EnT-mediated formal [3,3]-sigmatropic rearrangement of **3b** to **4b**, followed by an EnT-catalysed [2+2]-cycloaddition to form the final product **5b**. Calculations carried out at the ωB97X-D3/def2-QZVPPD//ωB97X-D3/def2-TZVP level of theory, all energies are given in kcal mol^−1^.

Next, we aimed to explain the key intermediates and mechanistic pathways involved in the cascade process (Fig. 3d). The [5+4] cycloaddition products formed in the initial step of the cascade reaction were successfully isolated only when 2-trifluoromethyl-substituted and 2,4-bis(trifluoromethyl)-substituted 6-phenylquinolines (**1e** and **1f**) were used, yielding compounds **3e** and **3f**, respectively. By contrast, intermediate **3b** was not detected in the photocatalytic reaction of 6-phenylquinoline **1b** bearing no *ortho*-substituent (Fig. 3e). These results can be rationalized by the diminished conjugation of the phenyl group and the olefin, arising from the twisted conformation caused by the *ortho*-substituents. This ultimately leads to the higher DvTE of [5+4] cycloaddition products **3e** (68.4 kcal mol^−1^) and **3f** (65.6 kcal mol^−1^), which exceeds that of the photocatalyst employed in the reaction, preventing a subsequent EnT process. By contrast, the DvTE of **3b**, without *ortho*-substituents at the 6-phenyl, was found to be sufficiently low (60.5 kcal mol^−1^) to allow for a consecutive EnT event. In agreement with this, no discrimination of the reactivities of **3b**, **3e** and **3f** was possible based on the thermal, pericyclic transition states ^**1**^**TS-4b**, ^**1**^**TS-4e** and ^**1**^**TS-4f** (Fig. 4). The computed barriers for all intermediates **3b**, **3e** and **3f** (Fig. 4) were found to vary from 29.2 kcal mol^−1^ to 30.0 kcal mol^−1^, rendering the classic, Woodward–Hoffmann allowed sigmatropic rearrangement unfeasible at the reaction temperature. However, consistent with the moderately high barrier, **3e** and **3f** can be thermally (120 °C) converted to the corresponding [3,3]-rearranged products **4e** and **4f** (Fig. 3d, 66% and 81%), proving that this thermal pathway becomes accessible at elevated temperatures^60^.

Since the calculated DvTE of **3b** is sufficiently low to undergo another EnT event, we investigated whether the formation of the [3,3]-rearranged product could also proceed at ambient temperature via an EnT-induced triplet pathway (Fig. 4). Indeed, the barrier for an intramolecular 6-*endo* attack in ^**3**^**3b** was found to be very low (Δ*G*^‡^ = 5.5 kcal mol^−1^), forming ^**3**^**Int-1** (Δ*G* = −24.9 kcal mol^−1^). From this intermediate, the [3,3]-rearranged species ^**1**^**4b** can be easily accessed via **MECP-4b** (Δ*E*^‡^ = 15.1 kcal mol^−1^). These computational results were confirmed experimentally following an independent synthesis of intermediate **3b**. Under the reaction conditions in the dark at 40 °C, **3b** yielded neither intermediate **4b** nor the final product **5b**, suggesting the involvement of photochemical activation (Fig. 3f). When **3b** was irradiated under photocatalytic reaction conditions, it produced the desired product **5b** in 82% yield, whereas direct excitation in the absence of **Ir–F** resulted in no product formation. Furthermore, the entire cascade reaction proceeded efficiently even at low temperatures such as 10 °C, −2 °C and −20 °C, which supports the photocatalytic pathway with low activation barriers (Fig. 3g).

After explaining both photocatalytic and thermal possibilities for the formation of [3,3]-rearranged intermediates **4b**, **4e** and **4f**, we next turned our attention to the mechanism and the origin of regioselectivity of the final [2+2] cycloaddition step. Given the low adiabatic triplet energy (*E*_T_ = 47.1 kcal mol^−1^) and DvTE (48.9 kcal mol^−1^) of **4b**, we propose that a third EnT event might be feasible to yield triplet ^**3**^**4b**, which could undergo intramolecular radical additions. This is in agreement with the observation that intermediates **4e** and **4f** yield corresponding [2+2] cycloadducts **5e** and **5f** when subjected to the reaction conditions. To rationalize the observed regioselectivity, all possible radical attacks of the C(5) and C(6) atoms, which possess most of the triplet state spin density in ^**3**^**4b**, to the terminal olefin in close spatial proximity were investigated computationally^61^ (Fig. 4). All the different pathways, except for a 4-*exo-*trig attack leading to ^**3**^**Int-2′′′** were found to be exergonic with similar thermodynamic driving forces between −6.5 kcal mol^−1^ for ^**3**^**Int-2** and −10.6 kcal mol^−1^ for ^**3**^**Int-2′**. However, the 5-*exo*-trig radical cyclization transition state ^**3**^**TS-Int-2** leading to ^**3**^**Int-2** is the most accessible (Δ*G*^‡^ = 13.9 kcal mol^−1^) and kinetically favoured by ΔΔ*G*^‡^ = 4.8 kcal mol^−1^ compared with the next lowest in energy, ^**3**^**TS-Int-2′′** (5-*endo*-trig). From ^**3**^**Int-2**, simultaneous intersystem crossing and radical–radical recombination via **MECP-5b** (Δ*E*^‡^ = 12.4 kcal mol^−1^) yields the observed [2+2] product regioisomer **5b** (Δ*G* = −43.7 kcal mol^−1^). The predicted selectivity matches the experimental confirmation by two-dimensional NMR and X-ray crystallography. The computational results were validated by experimental findings. When **4b** was subjected to the reaction conditions in the dark at 40 °C without a photocatalyst, no product formation was observed. Likewise, direct excitation of **4b** also failed to yield the desired product, confirming the EnT-catalysed activation. Under blue LED irradiation in the presence of **Ir–F**, the expected product **5b** was formed in 96% yield (Fig. 3h).

### Reaction scope

**Fig. 5:**
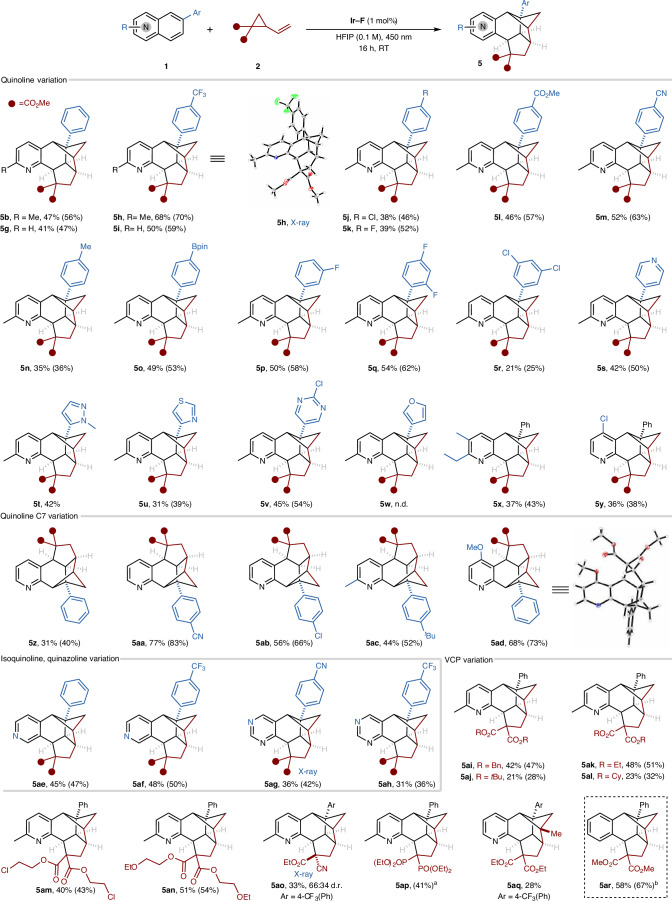
Substrate scope. Standard reaction conditions: 0.2 mmol of **1**, 1.0 mmol of **2**, and **Ir–F** (1 mol %) in HFIP (0.1 M) under irradiation with blue LEDs (*λ*_max_ = 450 nm), RT, 16 h. Yields of isolated compounds are provided. Crude ^1^H NMR yields are given in parentheses unless otherwise mentioned, calculated by using CH_2_Br_2_ as an internal standard. The diastereomeric ratio (d.r.) was determined from yields of isolated compounds. ^a^NMR yield is reported due to unsuccessful isolation. ^b^Ethyl acetate:HFIP (1:1, 0.1 M). Bpin, pinacol boronate ester.

With the optimized reaction condition in our hand, we set out to explore the substrate scope of the developed methodology (Fig. 5). In line with our investigations, a 6-(hetero)aryl or 7-(hetero)aryl group on quinoline was found crucial for the cascade reactivity. Notably, when quinoline without 2-methyl substitution was used, a single product (**5g**, 41%) was successfully formed indicating that the 2-methyl present in the model substrate is not essential for the transformation. First, we started to study the effect of different substituents on 6-phenylquinoline, noting that electron-deficient functional groups generally led to higher yields. A range of electron-withdrawing groups at the *para*-position, such as trifluoromethyl, chloro, fluoro, methylester and nitrile reacted smoothly to furnish the desired products (**5h**–**5m**) in moderate to good yields up to 68%. In addition to this, an electron-donating group such as *para*-methyl (**5n**) exhibited equal suitability for the reaction. Notably, a *para*- pinacol boronate ester substitution, which can act as a synthetic handle for a range of subsequent functionalizations, was also successfully converted to the product **5o**.

Next, we proceeded to investigate mono-substituted or di-substituted halogens at different positions on the 6-phenyl substituent. To our delight, a *meta-*fluoro-substitution (**5p**) showcased modest reactivity with a yield of 50%. Also, a 3,5-difluoro-substituted phenyl group worked well (**5q**, 54%), whereas the 3,5-dichloro variant produced 21% yield of the desired product **5r**. Heterocycles instead of a phenyl group in the 6-position, for instance, pyridine, pyrazole, thiazole and pyrimidine, were well tolerated (**5s**–**5v**, 31–45%), demonstrating the broad utility of the protocol. However, 6-furan substituted quinoline (**5w**) remained unreactive under these conditions. Subsequently, to further explore the method applicability, variation on the pyridine core was then attempted.

A multi-substituted quinoline bearing 2-ethyl-3-methyl was subjected to the reaction, which delivered the desired product (**5x**) in decent yield of 37%. A 4-chloro-substituted quinoline (**5y**) was also well tolerated and gave synthetically useful yield of 36%. Notably, when the phenyl ring was installed in the 7-position instead of the 6-position, the cascade reaction also proceeded efficiently to produce pyridine-fused/bridged polycyclic product (**5z**, 31%), however, inversion in the regioselectivity was observed. These findings were validated by X-ray analysis (Fig. 5). Electron-withdrawing groups at the *para*-position of the 7-phenyl substituent were readily tolerated, yielding the desired products **5aa** and **5ab** in moderate to good yields of 77% and 56%, respectively. An electron-donating substituent on the 7-phenyl group, such as *tert*-butyl delivered **5ac** in a good yield of 44%. Also, installing a strongly electron-donating methoxy group at the 4-position of the quinoline core yielded **5ad** in a good yield of 68%. Both isoquinolines and quinazolines exhibited good compatibility in the cascade transformation (**5ae**–**5ah**). Also, with these core structures, electron-withdrawing groups at the *para*-position of the 6-phenyl group were tolerated well, providing the desired cascade product up to 48% yields (**5af**–**5ah**). Despite the wide scope of the methodology, some limitations were identified. Heterocyclic substituents at the 6-position (furan, thiophene) as well as polyaromatic systems such as naphthalene were poorly tolerated with thiophene only giving 15% yield of desired product. Substituents at the *para*-position of the 6-phenyl substituent (methoxy, formyl and acetylene) failed to give any desired product, probably due to competing side reactions. The 8-fluoro-substituted quinoline yielded only trace amounts of product. In addition, substrate bearing free hydroxy group at the quinoline was also not compatible under the reaction conditions (Supplementary Fig. 3).

Next, we moved on to examine the scope of VCPs with the quinoline **1b**. Various 1,1-diesters were tested, such as benzyl (**5ai**), *tert*-butyl (**5aj**), ethyl (**5ak**) and cyclohexyl (**5al**)—participated successfully in the cascade reaction with excellent regiocontrol and diastereocontrol, although with an attenuated yield (21–41%). A highly versatile functional group, such as chlorine-bearing VCP, delivered the corresponding product (**5am**) in 40% yield. VCP containing an ethoxy substituent also showed compatibility under the reaction conditions, affording the product **5an** in 51% yield. Introduction of unsymmetrical VCP, such as 1-cyano-1-ethylester, offered the desired product **5ao** in 33% yield (diastereomeric ratio 66:34). Furthermore, we were pleased to find that ethylphosphonate bearing VCP was compatible under the optimized reaction conditions, yielding product **5ap** in 41% yield. Notably, VCP bearing substitution at the alkene was well tolerated to deliver **5aq** in synthetically useful yield. Unfortunately, cyano-substituted and phenylsulfonyl-substituted VCPs, vinyl aziridine and vinyl epoxide failed to deliver the products, possibly owing to decomposition under the reaction conditions. VCPs deprived of diester functionality also did not yield the productive reactivity (Supplementary Fig. 3). The methodology developed for bicyclic azaarenes proved equally effective to the non-heteroaromatic systems such as naphthalene derivative, affording the desired product **5ar** in 58% yield. This highlights the broad applicability of the methodology beyond heteroaromatic systems.

### Diversification

**Fig. 6:**
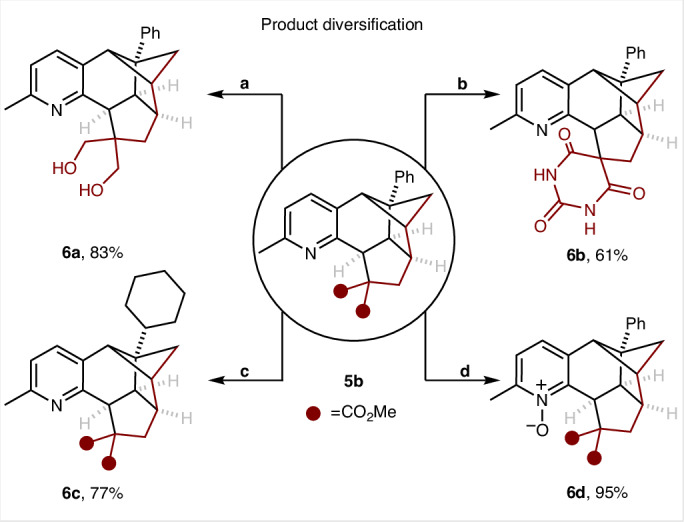
Postsynthetic modifications. Reaction conditions: **a**, **5b** (0.2 mmol), DIBAL-H (5.0 equiv.), tetrahydrofuran, 0 °C to RT, overnight. **b**, **5b** (0.2 mmol), CO(NH_2_)_2_ (6.0 equiv.), KO*t*Bu (2.0 equiv.), DMSO, RT, overnight. **c**, **5b** (0.2 mmol), PtO_2_ (5 mol%), activated charcoal, trifluoroacetic acid, 70 °C, 5 bar H_2_, 4 h. **d**, **5b** (0.2 mmol), *meta*-chloroperbenzoic acid (1.5 equiv.), CH_2_Cl_2_, RT. For additional experimental details, see the Supplementary Information.

To further demonstrate the synthetic use of the developed cascade reaction, the obtained products were readily converted into a range of structurally and functionally diverse derivatives (Fig. 6). In this regard, we managed to completely reduce geminal diester in **5b** using DIBAL-H to the corresponding diol derivative **6a** in 83% yield. These geminal diester groups offer an opportunity to further transform the product into spirocyclic frameworks. The diester groups in **5b** were smoothly converted into a spirocyclic product (**6b**, 61%), another class of medicinally relevant scaffolds, such as barbiturate analogues. Furthermore, we performed a selective hydrogenation of the 6-phenyl group, leading to an even more *sp*^3^-rich molecular framework (**6c**, 77%). Motivated by the importance of heterocyclic N-oxides as synthetic linchpins and their widespread occurrence in pharmaceuticals^62^, **5b** was converted into the respective N-oxide (**6d**, 95%).

## Conclusions

In summary, we report an EnT-catalysed cascade transformation of bicyclic azaarenes with VCPs to afford highly complex, C(*sp*^3^)-rich pentacyclic scaffolds. This reaction proceeds through a photocatalytic, EnT-mediated cascade involving intermolecular [5+4] cycloaddition, stepwise formal [3,3]-sigmatropic rearrangement and an intramolecular [2+2] cycloaddition. This cascade process was experimentally confirmed by the successful isolation of the key intermediates involved, mechanistic control experiments and computational studies, which also rationalize the observed regioselectivity. This strategy enabled the intermolecular higher-order [5+4] cycloaddition beyond the conventional [4+2] paradigms of bicyclic azaarenes, overcoming the transannular strain and entropic challenges associated with constructing medium-sized, bridged frameworks. A key insight from this work is that the formal [3,3]-sigmatropic rearrangement proceeds stepwise via a triplet EnT pathway, contrasting the well-known, concerted pericyclic mechanism. These mechanistic insights facilitate further research directions in intermolecular higher-order cycloadditions and photochemical rearrangements. In addition, downstream derivatizations of the product motifs demonstrated the synthetic applicability of the methodology. Collectively, this work brings the conceptual merger of three (TTEnT)-enabled distinct photochemical steps in a cascade fashion, which provides streamlined access to architecturally new ring systems with exceptional selectivity. Given the ubiquity of pyridine-fused rings in drug discovery, we anticipate that this methodology will unlock the readily and efficient synthesis of such scaffolds under mild visible light conditions.

## Methods

The general procedure for the cascade dearomative [5+4] cycloaddition/[3,3]-sigmatropic rearrangement/[2+2] cycloaddition of bicyclic azaarenes is as follows: To an oven-dried Schlenk tube equipped with a Teflon-coated magnetic stir bar, azaarene **1** (1.0 equiv., 0.2 mmol) and [Ir(dF(CF_3_)ppy)_2_(dtbbpy)][PF_6_] (**Ir–F**) (1 mol%, 0.002 mmol) were added. The tube was evacuated and backfilled with argon three times. Under an argon atmosphere, HFIP (0.1 M) and VCP **2** (5.0 equiv., 1.0 mmol) were added consecutively. The tube was sealed tightly with a screw-cap. The reaction mixture was irradiated with 30 W blue LEDs (*λ*_max_ = 450 nm) for 16 h. The solvent was removed under reduced pressure and ^1^H NMR yield was determined using dibromomethane (CH_2_Br_2_) as an internal standard. The crude product was purified by column chromatography on silica gel to afford the desired product **5**.

## Supplementary information

Supplementary Information Supplementary Methods, SupplementaryDiscussions, Supplementary Figs. 1–26 and Supplementary Tables 1–7.

Supplementary Data 1 CCDC_Nr for all the crystal structures.

Supplementary Data 2 Cif file for structure **3a**.

Supplementary Data 3 Cif file for structure **3b**.

Supplementary Data 4 Cif file for structure **3f**.

Supplementary Data 5 Cif file for structure **4a**.

Supplementary Data 6 Cif file for structure **5ad**.

Supplementary Data 7 Cif file for structure **5ag**.

Supplementary Data 8 Cif file for structure **5ao**.

Supplementary Data 9 Cif file for structure **5h**.

## Data Availability

All detailed experimental procedures, mechanistic and computational studies, sensitivity screening, robustness screening, NMR spectra and computational methodology details are provided in the Supplementary Information. All obtained geometries, as well as ORCA^61,63^ input and output files have deposited in the ioChem-BD repository^64^ and are available via the DOI at 10.19061/iochem-bd-6-603. Crystallographic data for the structures reported in this study have been deposited at the Cambridge Crystallographic Data Centre (CCDC) under deposition numbers CCDC 2502652 (**4a**), 2502653 (**3a**), 2502654 (**3f**), 2502655 (**5ad**), 2502656 (**5ag**), 2502657 (**5h**), 2502658 (**3b**) and 2502659 (**5ao**). Copies of the data can be obtained free of charge from the CCDC via www.ccdc.cam.ac.uk/data_request/cif.
