# Supplementary material for: Triple energy transfer-enabled dearomative cycloaddition/rearrangement cascade of bicyclic azaarenes to structurally complex products
Source: Nat Catal. 2026 Jul 9;9(7):750–60. doi: 10.1038/s41929-026-01566-z (PMC13395622; doi:10.1038/s41929-026-01566-z)
Supplement: Supplementary file 1 — Supplementary Methods, SupplementaryDiscussions, Supplementary Figs. 1–26 and Supplementary Tables 1–7. [file 41929_2026_1566_MOESM1_ESM.pdf]

nature catalysis

Supplementary information

https://doi.org/10.1038/s41929-026-01566-z

Triple energy transfer-enabled dearomative cycloaddition/rearrangement cascade of bicyclic azaarenes to structurally complex products

In the format provided by the authors and unedited

TABLE OF CONTENTS

SUPPLEMENTARY METHODS....3

Glassware, Solvents and Reagents....3

Photochemical set-up and light sources....3

Chromatography and Data Analysis....4

EXPERIMENTAL DATA....4

General Procedure A: Synthesis of Arylazaarenes....4

General Procedure B: Synthesis of Arylquinolines....5

Bicyclic Azaarenes Library....6

General Procedure C: Synthesis of Vinylcyclopropanes....26

Vinylcyclopropanes library....26

General Procedure D: Dearomative Cycloaddition/Rearrangement Cascade of Bicyclic Azaarenes....33

Substrate Limitations....59

Time Course Study....60

Additive-Based Robustness Screening....62

Condition-Based Sensitivity Screening....64

MECHANISTIC STUDIES....66

Reaction Development....66

Optimization studies....68

Intermediate Studies....69

UV-vis Absorption Spectroscopy....77

Stern-Volmer Luminescence Quenching Studies....77

Cyclic Voltammetry Analysis....78

Quantum Yield Analysis....79

Radical Trapping Studies....81

TEMPO trapping study....81

BHT trapping study....81

PRODUCT DIVERSIFICATION....82

Reduction of Diester Group....82

Synthesis of Barbituric Acid Derivative....83

Selective Hydrogenation of Phenyl ring....84

Synthesis of Pyridine *N*-Oxide Derivative....85

COMPUTATIONAL STUDIES....86

Reaction Coordinate Calculations by DFT....86

Structure assignments and species summary for calculated reaction coordinate....87

Energy summary 87
Spin density analysis 88
Comparison of SMD and CPCM solvation models 88
Investigation of the influence of aromatic substitutions 90
Dynamic Vertical Triplet Energies 91
Vertical triplet energy distributions and DvTEs 92
Acknowledgements 94
CRYSTAL STRUCTURES 94
SPECTROSCOPIC DATA 106
SUPPLEMENTARY REFERENCES 220

SUPPLEMENTARY METHODS

Glassware, Solvents and Reagents

All reactions were conducted under an inert atmosphere of argon using Schlenk manifold techniques unless stated otherwise. All glassware and Teflon-coated magnetic stir bars were dried in an oven at 120 °C prior to use. All anhydrous solvents were commercially supplied and stored over 3 Å mol sieves or dried using an activated alumina column drying system. Reagents were purchased from commercial sources and used as received.

Photochemical set-up and light sources

Unless otherwise stated, photochemical reactions were performed in a Hepatochem EvoluChemTM PhotoRedOx Box Duo device and irradiated with two EvoluChem™ HCK1012-02-012 LEDs (30 W, λ_{max} = 450 nm). When the internal fan was used, the reaction temperature was determined to be between 30 °C and 33 °C.

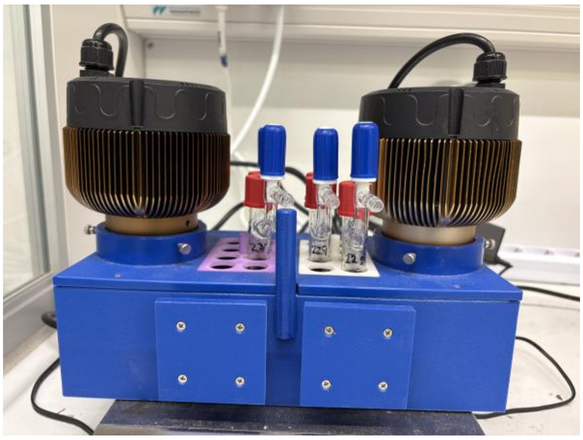

Supplementary Figure 1: Experimental set-up for photochemical reactions.

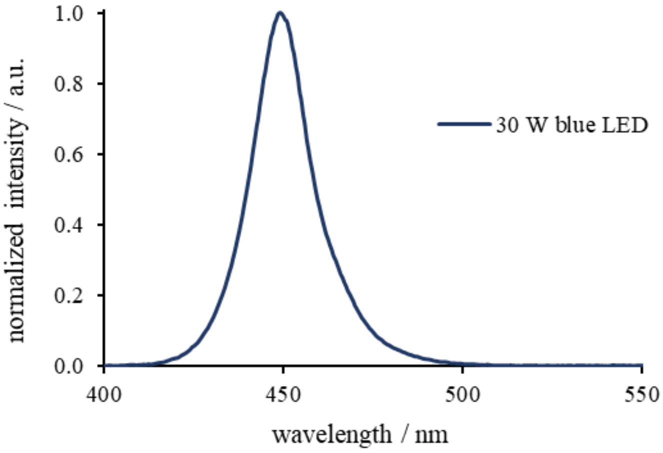

Supplementary Figure 2: Emission spectrum of LEDs used (λ_{max} = 450 nm).

Chromatography and Data Analysis

Thin layer chromatography (TLC) was performed to monitor reactions when practical using Merck silica gel 60 F_{254} aluminum plates and visualised under UV light, or by staining with aqueous basic potassium permanganate followed by heating. **Flash column chromatography** (FCC) was carried out using Acros Organics silica gel (35–70 mesh) or a Biotage Isolera™ flash purification system. **NMR spectra** were recorded on a Bruker Avance II 400, Agilent DD2 500 or DD2 600 spectrometers. All spectral data was acquired at 295 K. Deuterated solvents were purchased from Eurisotop ($CDCl_3$, deuteration > 99.8%). Chemical shifts (δ) are reported in parts per million (ppm) and referenced to $CDCl_3$ (^{1}H: 7.26 ppm; ^{13}C: 77.16 ppm). Coupling constants (*J*) are given in Hertz (Hz) and refer to corresponding multiplicities (s = singlet, d = doublet, t = triplet, q = quartet, quin = quintet, hex = hextet, h = heptet, m = multiplet, app = apparent, br. = broad signal, br. s = broad singlet, dd = doublet of doublets, etc.). The ^{1}H NMR spectra are reported as follows: chemical shift (multiplicity, coupling constants, number of protons). NMR assignments were made according to spin systems, using two-dimensional NMR spectroscopy (COSY, HSQC, HMBC) to assist the characterization. NMR yields were determined by ^{1}H NMR analysis using dibromomethane (CH_2Br_2) as an internal standard. **High-resolution mass spectra** (HRMS) were measured by the MS service division of the Institute of Organic Chemistry at the University of Münster. All spectra measurements were carried out on a Bruker Daltonics MicroToF, ThermoFisher Scientific Orbitrap LTQ XL, Thermo Fisher Scientific Exploris 120 Electrospray Orbitrap or Thermo FisherScientific Orbitrap Velos Pro mass spectrometer using electrospray ionization (ESI).

EXPERIMENTAL DATA

General Procedure A: Synthesis of Arylazaarenes

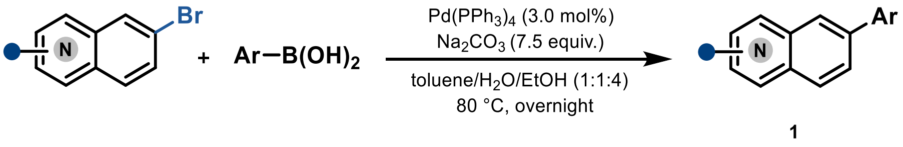

Following the modified literature procedure,[1] bromoazaarene derivative (1.0 equiv., if solid), arylboronic acid (1.3 equiv.), Na_2CO_3(7.5 equiv.), and $Pd(PPh_3)_4$ (3.0 mol%) were added to an oven-dried Schlenk tube with a Teflon-coated magnetic stir bar. The tube was evacuated and backfilled with argon three times. Under an argon atmosphere, toluene (0.3 M), H_2O (0.3 M), and EtOH (1.2 M) were added. Bromoazaarene (1.0 equiv., if liquid) was added to the tube. The mixture was stirred at 80 °C overnight. The mixture was allowed to cool to room temperature and quenched with saturated aqueous NH_4Cl solution. The phases were separated and the aqueous layer was extracted with ethyl acetate. The organic layer was dried over anhydrous $MgSO_4$ and the solvent was removed under reduced pressure. The crude product was purified by flash column chromatography on silica gel to afford the desired arylazaarenes **1**.

General Procedure B: Synthesis of Arylquinolines

Step 1: Synthesis of 2-methyl-6-(4,4,5,5-tetramethyl-1,3,2-dioxaborolan-2-yl)quinoline (S1)

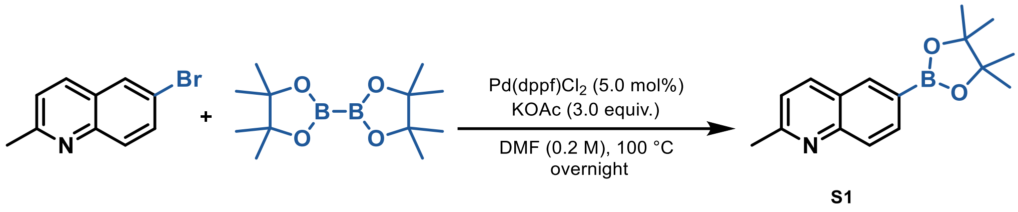

Following the modified literature procedure,[2] to an oven-dried 150.0 mL Schlenk tube with a Teflon-coated magnetic stir bar, 6-bromo-2-methylquinoline (1.0 equiv., 15.0 mmol, 3.33 g), B_2Pin_2 (1.1 equiv., 16.5 mmol, 4.19 g), KOAc (3.0 equiv., 45.0 mmol, 4.42 g), and Pd(dppf)Cl_2 (5.0 mol%, 549 mg) were added. The tube was evacuated and backfilled with argon three times. Then 75.0 mL of DMF (0.2 M) was added to the Schlenk tube under an argon atmosphere. After that, the reaction was stirred at 100 °C overnight. The crude reaction mixture was filtered through silica, and the filtrate was concentrated under reduced pressure. The crude product was purified by flash column chromatography on silica gel to afford compound **S1** (2.82 g, 70%) as a yellowish solid.

Step 2:

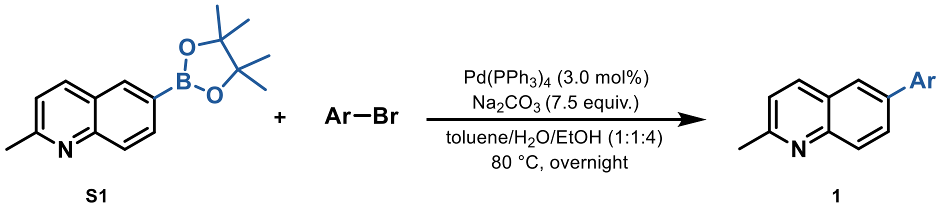

Following the modified literature procedure,[1] **S1** (1.3 equiv., if solid), aryl bromide (1.0 equiv.), Na_2CO_3 (7.5 equiv.), and Pd(PPh_3)$_4$ (3.0 mol%) were added to an oven-dried Schlenk tube with a Teflon-coated magnetic stir bar. The tube was evacuated and backfilled with argon three times. Under an argon atmosphere, toluene (0.3 M), H_2O (0.3 M), and EtOH (1.2 M) were added. **S1** (1.3 equiv., if liquid) was added to the tube. The mixture was stirred at 80 °C overnight. The mixture was allowed to cool to room temperature and quenched with saturated aqueous NH_4Cl solution. The phases were separated and the aqueous layer was extracted with ethyl acetate, dried over anhydrous $MgSO_4$, and the solvent was removed under reduced pressure. The crude product was purified by flash column chromatography on silica gel to afford the desired arylazaarenes.

Bicyclic Azaarenes Library

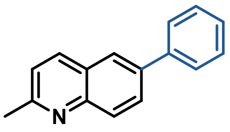
1b

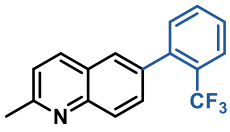
1e

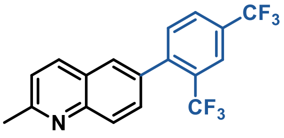
1f

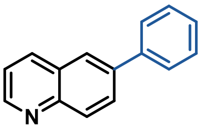
1g

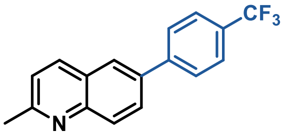
1h

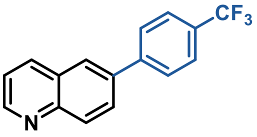
1i

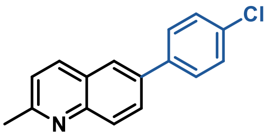
1j

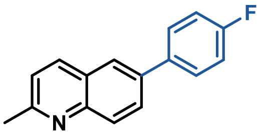
1k

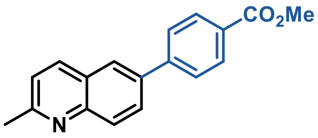
1l

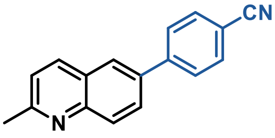
1m

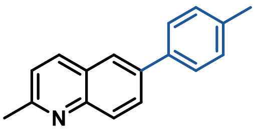
1n

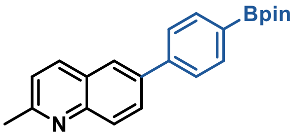
1o

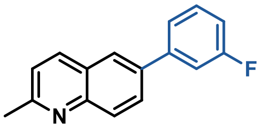
1p

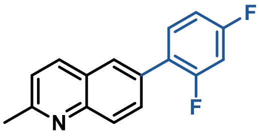
1q

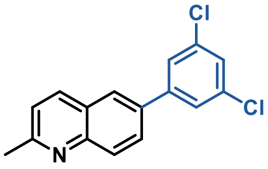
1r

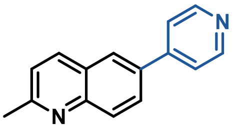
1s

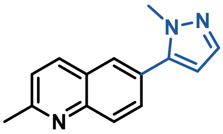
1t

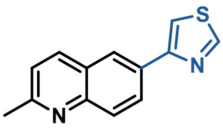
1u

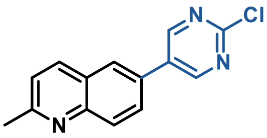
1v

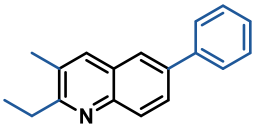
1x

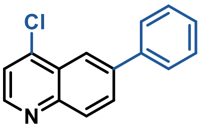
1y

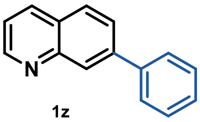
1z

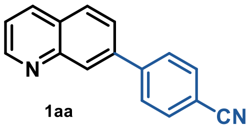
1aa

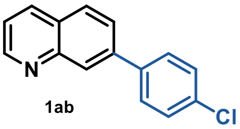
1ab

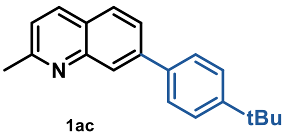
1ac

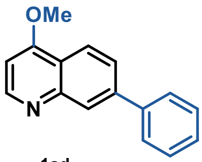
1ad

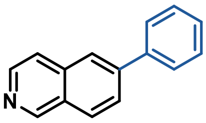
1ae

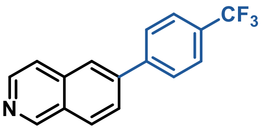
1af

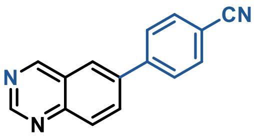
1ag

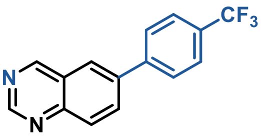
1ah

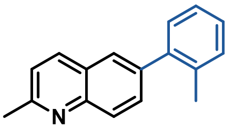
1ai

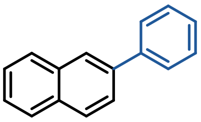
1aj

Note: All bicyclic azaarenes shown above were synthesized either using **General Procedure A** or **General Procedure B**.

2-methyl-6-(4,4,5,5-tetramethyl-1,3,2-dioxaborolan-2-yl)quinoline (S1)

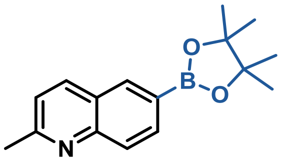

TLC: R_f = 0.3 (80:20 pentane/EtOAc).

NMR Spectroscopy:

^{1}H NMR (400 MHz, $CDCl_3$): δ_H 8.29 (br. s, 1H), 8.14 – 7.95 (m, 3H), 7.29 (d, *J* = 8.4 Hz, 1H), 2.76 (s, 3H), 1.38 (s, 12H) ppm.

^{13}C NMR (101 MHz, $CDCl_3$): δ_C 160.2, 137.2, 135.9, 134.6, 127.6, 126.0, 122.2, 84.2, 25.2, 25.1 ppm.

^{11}B NMR (128 MHz, $CDCl_3$): δ 31.20 ppm.

HRMS (ESI^+): m/z calc'd for $C_{16}H_{20}NO_2BNa$ $[M+Na]^+$: 292.14793, found: 292.14766.

2-methyl-6-phenylquinoline (1b)

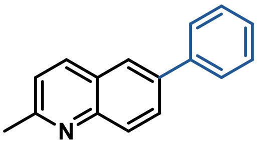

Following **General Procedure A**, the title compound was synthesized using 6-bromo-2-methylquinoline (1.0 equiv., 20.0 mmol, 4.44 g), phenylboronic acid (1.3 equiv., 26.0 mmol, 3.17 g), Na_2CO_3 (7.5 equiv., 150 mmol, 15.90 g), $Pd(PPh_3)_4$ (3.0 mol%, 693 mg), toluene (66.6 mL), H_2O (66.6 mL), and EtOH (16.6 mL). The crude product was purified by flash column chromatography (SiO_2; 100:0 to 90:10 pentane/EtOAc) to yield **1b** (3.73 g, 85%) as a yellowish solid.

TLC: R_f = 0.3 (90:10 pentane/EtOAc).

NMR Spectroscopy:

^{1}H NMR (400 MHz, $CDCl_3$): δ_H 8.10 (d, *J* = 8.7 Hz, 2H), 7.99 – 7.92 (m, 2H), 7.75 – 7.68 (m, 2H), 7.54 – 7.45 (m, 2H), 7.43 – 7.36 (m, 1H), 7.31 (d, *J* = 8.4 Hz, 1H), 2.77 (s, 3H) ppm.

^{13}C NMR (101 MHz, $CDCl_3$): δ_C 159.1, 147.3, 140.6, 138.6, 136.6, 129.3, 129.1, 129.1, 127.7, 127.5, 126.8, 125.4, 122.5, 25.5 ppm.

HRMS (ESI^+): m/z calc'd for $C_{16}H_{13}NNa$ $[M+Na]^+$: 242.09402, found: 242.09406.

2-methyl-6-(2-(trifluoromethyl)phenyl)quinoline (1e)

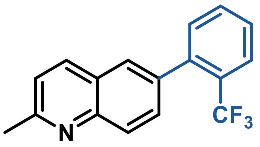

According to **General Procedure A**, the following compound was synthesized using 6-bromo-2-methylquinoline (1 equiv., 1.0 mmol, 222 mg), (2-(trifluoromethyl)phenyl)boronic acid (1.3 equiv., 1.3 mmol, 247 mg), Na_2CO_3 (7.5 equiv., 7.5 mmol, 795 mg), $Pd(PPh_3)_4$ (3.0 mol%, 35 mg), toluene (3.3 mL), H_2O (3.3 mL), and EtOH (0.8 mL). The crude product was purified by flash column chromatography (SiO_2; 100:0 to 85:15 pentane/EtOAc) to yield **1e** (236 mg, 82%) as a white solid.

TLC: R_f = 0.2 (90:10 pentane/EtOAc).

NMR Spectroscopy:

^{1}H NMR (400 MHz, $CDCl_3$): δ_H 8.07 (d, *J* = 8.5 Hz, 2H), 7.79 (dd, *J* = 7.9, 1.5 Hz, 1H), 7.73 (d, *J* = 2.0 Hz, 1H), 7.66 (dd, *J* = 8.7, 2.0 Hz, 1H), 7.63 – 7.56 (m, 1H), 7.55 – 7.48 (m, 1H), 7.44 – 7.38 (m, 1H), 7.34 (dd, *J* = 8.3, 1.2 Hz, 1H), 2.79 (s, 3H) ppm.

^{13}C{^{19}F} NMR (126 MHz, $CDCl_3$): δ_C 159.3, 140.6, 137.8, 137.4, 132.3, 131.6, 131.5, 128.8, 127.9, 127.7, 127.4, 126.4, 126.0, 124.2, 122.7, 25.0 ppm.

^{19}F NMR (376 MHz, $CDCl_3$): δ_F -56.8 ppm.

HRMS (ESI^+): m/z calc'd for $C_{17}H_{13}NF_3$ $[M+H]^+$: 288.09946, found: 288.09942.

6-(2,4-bis(trifluoromethyl)phenyl)-2-methylquinoline (1f)

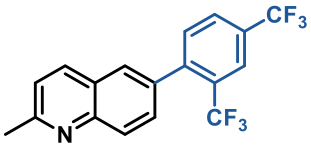

According to **General Procedure A**, the following compound was synthesized using 6-bromo-2-methylquinoline (1.0 equiv., 3.0 mmol, 666 mg), (2,4-bis(trifluoromethyl)phenyl)boronic acid (1.3 equiv., 3.9 mmol, 1.0 g), Na_2CO_3 (7.5 equiv., 22.5 mmol, 2.38 g), $Pd(PPh_3)_4$ (3.0 mol%, 104 mg), toluene (10.0 mL), H_2O (10.0 mL), EtOH (2.5 mL) and purified by flash column chromatography (SiO_2; 100:0 to 80:20 pentane/EtOAc) to afford compound **1f** (863 mg, 81%) as a yellowish solid.

TLC: R_f = 0.5 (70:30 pentane/EtOAc).

NMR Spectroscopy:

^{1}H NMR (400 MHz, $CDCl_3$): δ_H 8.13 – 8.04 (m, 3H), 7.87 (d, *J* = 10.0 Hz, 1H), 7.73 (d, *J* = 1.8 Hz, 1H), 7.64 (dd, *J* = 8.7, 2.0 Hz, 1H), 7.57 (d, *J* = 8.8 Hz, 1H), 7.37 (d, *J* = 8.4 Hz, 1H), 2.80 (s, 3H) ppm.

$^{13}C\{^{19}F\}$ NMR (126 MHz, $CDCl_3$): δ_C 160.1, 147.6, 144.6, 136.5, 135.8, 133.2, 130.4, 130.2, 129.8, 128.6, 128.3, 127.7, 125.9, 123.7, 123.6, 123.5, 122.9, 25.6 ppm.

^{19}F NMR (376 MHz, CDCl3): δ_F -57.25, -62.75 ppm.

HRMS (ESI^+): m/z calc'd for $C_{18}H_{12}F_6N$ $[M+H]^+$: 356.08684, found: 356.08674.

6-phenylquinoline (1g)

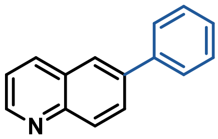

According to **General Procedure A**, the following compound was synthesized using 6-bromoquinoline (1.0 equiv., 10.0 mmol, 2.08 g), phenylboronic acid (1.3 equiv., 13.0 mmol, 1.58 g), Na_2CO_3 (7.5 equiv., 75.0 mmol, 7.90 g), $Pd(PPh_3)_4$ (3.0 mol%, 347 mg), toluene (33.0 mL), H_2O (33.0 mL), EtOH (8.0 mL). Purified by flash column chromatography (SiO_2; 100:0 to 75:25 pentane/EtOAc) to afford compound **1g** (1.70 g, 83%) as a yellowish solid.

TLC: R_f = 0.5 (70:30 pentane/EtOAc).

NMR Spectroscopy:**^1H NMR** (400 MHz, $CDCl_3$): δ_H 8.92 (dd, J = 4.2, 1.7 Hz, 1H), 8.27 – 8.10 (m, 2H), 8.02 – 7.94 (m, 2H), 7.80 – 7.68 (m, 2H), 7.55 – 7.45 (m, 2H), 7.43 – 7.37 (m, 2H) ppm.

^{13}C NMR (101 MHz, $CDCl_3$): δ_C 150.4, 147.7, 140.4, 139.4, 136.3, 130.0, 129.3, 129.1, 128.5, 127.8, 127.5, 125.5, 121.5 ppm.

HRMS (ESI^+): m/z calc'd for $C_{15}H_{12}N$ $[M+H]^+$: 206.09643, found: 206.09639.

2-methyl-6-(4-(trifluoromethyl)phenyl)quinoline (1h)

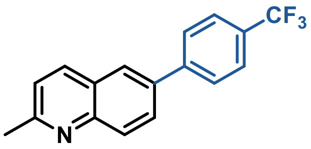

According to **General Procedure A**, the following compound was synthesized using 6-bromo-2-methylquinoline (1.0 equiv., 3.0 mmol, 666 mg), 4-(trifluoromethyl)phenyl)boronic acid (1.3 equiv., 3.9 mmol, 741 mg), Na_2CO_3 (7.5 equiv., 22.5 mmol, 2.38 g), $Pd(PPh_3)_4$ (3.0 mol%, 104 mg), toluene (10.0 mL), H_2O (10.0 mL), EtOH (2.5 mL). Purified by flash column chromatography (SiO_2; 100:0 to 60:40 pentane/EtOAc) to afford compound **1h** (784 mg, 91%) as a yellowish solid.

TLC: R_f = 0.3 (60:40 pentane/EtOAc).

NMR Spectroscopy:

^{1}H NMR (400 MHz, $CDCl_3$): δ_H 8.17 – 8.08 (m, 2H), 7.97 (d, *J* = 2.0 Hz, 1H), 7.93 (dd, *J* = 8.6, 2.1 Hz, 1H), 7.82 – 7.76 (m, 2H), 7.76 – 7.70 (m, 2H), 7.34 (d, *J* = 8.4 Hz, 1H), 2.78 (s, 3H) ppm.

^{13}C{^{19}F} NMR (126 MHz, $CDCl_3$): δ_C 159.8, 147.7, 144.1, 137.0, 136.5, 129.7, 129.6, 128.8, 127.7, 126.7, 126.0, 125.9, 124.4, 122.8, 25.6 ppm.

^{19}F NMR (376 MHz, CDCl3): δ_F -62.41 ppm.

HRMS (ESI$^+$): m/z calc'd for $C_{17}H_{13}F_3N$ $[M+H]^+$: 288.09946, found: 288.09938.

6-(4-(trifluoromethyl)phenyl)quinoline (1i)

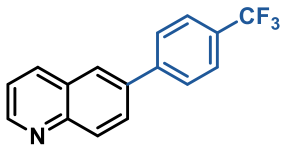

According to **General Procedure A**, the following compound was synthesized using 6-bromoquinoline (1.0 equiv., 2.0 mmol, 416 mg), 4-(trifluormethyl)phenylboronic acid (1.3 equiv., 2.6 mmol, 494 mg), Na_2CO_3 (7.5 equiv., 15.0 mmol, 1.59 g), $Pd(PPh_3)_4$ (3.0 mol%, 69 mg), toluene (6.6 mL), H_2O (6.6 mL), EtOH (1.6 mL). Purified by flash column chromatography (SiO_2; 100:0 to 75:25 pentane/EtOAc) to afford compound **1i** (497 mg, 91%) as a yellowish solid.

TLC: R_f = 0.5 (70:30 pentane/EtOAc).

NMR Spectroscopy:

^{1}H NMR (400 MHz, $CDCl_3$): δ_H 8.96 (dd, *J* = 4.3, 1.7 Hz, 1H), 8.27 – 8.23 (m, 2H), 8.03 (d, *J* = 2.0 Hz, 1H), 7.98 (dd, *J* = 8.8, 2.1 Hz, 1H), 7.83 – 7.74 (m, 4H), 7.48 (dd, *J* = 8.3, 4.3 Hz, 1H) ppm.

^{13}C{^{19}F} NMR (126 MHz, $CDCl_3$): δ_C 150.5, 147.3, 143.7, 138.2, 137.3, 130.0, 129.9, 129.3, 128.7, 127.9, 126.2, 126.0, 124.3, 121.8 ppm.

^{19}F NMR (376 MHz, CDCl3): δ_F -62.46 ppm.

HRMS (ESI$^+$): m/z calc'd for $C_{16}H_{11}F_3N$ $[M+H]^+$: 274.08381, found: 274.08378.

6-(4-chlorophenyl)-2-methylquinoline (1j)

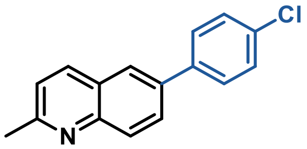

According to **General Procedure A**, the following compound was synthesized using 6-bromo-2-methylquinoline (1.0 equiv., 2.0 mmol, 444 mg), (4-chlorophenyl)boronic acid (1.3 equiv., 2.6 mmol, 407 mg),

Na_2CO_3 (7.5 equiv., 15.0 mmol, 1.59 g), $Pd(PPh_3)_4$ (3.0 mol%, 69 mg), toluene (6.6 mL), H_2O (6.6 mL), EtOH (1.6 mL). Purified by flash column chromatography (SiO_2; 100:0 to 88:12 pentane/EtOAc) to afford compound **1j** (335 mg, 66%) as a light-yellow solid.

TLC: R_f = 0.3 (90:10 pentane/EtOAc).

NMR Spectroscopy:

^{1}H NMR (400 MHz, $CDCl_3$): δ_H 7.93 (d, *J* = 9.3 Hz, 1H), 7.88 (d, *J* = 8.5 Hz, 1H), 7.77 – 7.66 (m, 2H), 7.43 (d, *J* = 8.3 Hz, 2H), 7.27 (d, *J* = 8.3 Hz, 2H), 7.13 (d, *J* = 8.3 Hz, 1H), 2.61 (s, 3H) ppm.

^{13}C NMR (101 MHz, $CDCl_3$): δ_C 159.3, 147.3, 138.9, 137.1, 136.4, 133.7, 129.3, 129.1, 128.7, 128.6, 126.6, 125.2, 122.6, 25.4 ppm.

HRMS (ESI$^+$): m/z calc'd for $C_{16}H_{13}NCl$ $[M+H]^+$: 254.07310, found: 254.07322.

6-(4-fluorophenyl)-2-methylquinoline (1k)

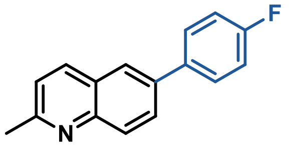

According to **General Procedure A**, the following compound was synthesized using 6-bromo-2-methylquinoline (1.0 equiv., 2.0 mmol, 444 mg), 4-florophenylboronic acid (1.3 equiv., 2.6 mmol, 364 mg), Na_2CO_3 (7.5 equiv., 15.0 mmol, 1.59 g), $Pd(PPh_3)_4$ (3.0 mol%, 69 mg), toluene (6.6 mL), H_2O (6.6 mL), EtOH (1.6 mL). Purified by flash column chromatography (SiO_2; 100:0 to 70:30 pentane/EtOAc) to afford compound **1k** (394 mg, 83%) as a yellowish solid.

TLC: R_f = 0.4 (60:40 pentane/EtOAc).

NMR Spectroscopy:

^{1}H NMR (400 MHz, $CDCl_3$): δ_H 8.11 – 8.06 (m, 2H), 7.90 – 7.87 (m, 2H), 7.66 – 7.63 (m, 2H), 7.31 (d, *J* = 8.4 Hz, 1H), 7.19 – 7.14 (m, 2H), 2.77 (s, 3H) ppm.

^{13}C{^{19}F} NMR (126 MHz, $CDCl_3$): δ_C 162.7, 159.2, 147.3, 137.5, 136.7, 136.3, 129.3, 129.0, 129.0, 126.7, 125.2, 122.6, 115.9, 25.5 ppm.

^{19}F NMR (376 MHz, CDCl3): δ_F -115.36 ppm.

HRMS (ESI$^+$): m/z calc'd for $C_{16}H_{13}FN$ $[M+H]^+$: 238.10265, found: 238.10248.

methyl 4-(2-methylquinolin-6-yl)benzoate (1l)

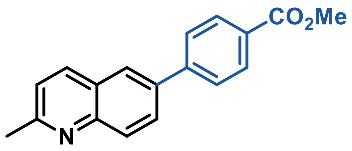

According to **General Procedure A**, the following compound was synthesized using 6-bromo-2-methylquinoline (1.0 equiv., 2.0 mmol, 444 mg), 4-methoxycarbonylphenylboronic acid (1.3 equiv., 2.6 mmol, 468 mg), Na_2CO_3 (7.5 equiv., 15.0 mmol, 1.59 g), $Pd(PPh_3)_4$ (3.0 mol%, 69 mg), toluene (6.6 mL), H_2O (6.6 mL), EtOH (1.6 mL). Purified by flash column chromatography (SiO_2; 100:0 to 75:25 pentane/EtOAc) to afford compound **1l** (299 mg, 54%) as a yellowish solid.

TLC: R_f = 0.3 (70:30 pentane/EtOAc).

NMR Spectroscopy:

^{1}H NMR (400 MHz, $CDCl_3$): δ_H 8.17 – 8.08 (m, 4H), 8.00 (d, *J* = 2.1 Hz, 1H), 7.95 (dd, *J* = 8.7, 2.1 Hz, 1H), 7.81 – 7.72 (m, 2H), 7.33 (d, *J* = 8.4 Hz, 1H), 3.95 (s, 3H), 2.77 (s, 3H) ppm.

^{13}C NMR (101 MHz, $CDCl_3$) δ_C 167.1, 159.7, 147.7, 145.0, 137.3, 136.6, 130.4, 129.5, 129.3, 128.9, 127.4, 126.7, 126.0, 122.8, 52.3, 25.6 ppm.

HRMS (ESI^+): m/z calc'd for $C_{18}H_{15}NO_2Na$ $[M+Na]^+$: 300.09950, found: 300.09937.

4-(2-methylquinolin-6-yl)benzonitrile (1m)

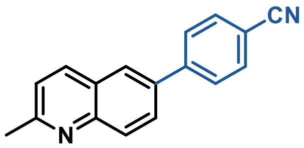

According to **General Procedure A**, the following compound was synthesized 6-bromo-2-methylquinoline (1.0 equiv., 3.0 mmol, 666 mg), (4-cyanophenyl)boronic acid (1.3 equiv., 3.9 mmol, 573 mg), Na_2CO_3 (7.5 equiv., 22.5 mmol, 2.38 g), $Pd(PPh_3)_4$ (3.0 mol%, 104 mg), toluene (10.0 mL), H_2O (10.0 mL), EtOH (2.5 mL). Purified by flash column chromatography (SiO_2; 100:0 to 80:20 pentane/EtOAc) to afford compound **1m** (616 mg, 84%) as a white solid.

TLC: R_f = 0.3 (70:30 pentane/EtOAc).

NMR Spectroscopy:

^{1}H NMR (400 MHz, $CDCl_3$): δ_H 8.21 (d, *J* = 8.8 Hz, 1H), 8.17 (d, *J* = 8.4 Hz, 1H), 8.00 (d, *J* = 2.1 Hz, 1H), 7.94 (dd, *J* = 8.8, 2.1 Hz, 1H), 7.85 – 7.71 (m, 4H), 7.38 (d, *J* = 8.4 Hz, 1H), 2.82 (s, 3H) ppm.

^{13}C NMR (101 MHz, $CDCl_3$): δ_C 159.9, 144.9, 137.3, 136.8, 132.9, 129.2, 129.1, 128.1, 126.8, 126.2, 123.1, 118.9, 111.5, 25.2 ppm.

HRMS (ESI$^+$): m/z calc'd for $C_{17}H_{13}N_2$ [M+H]$^+$: 245.10732, found: 245.10730.

2-methyl-6-(*p*-tolyl)quinoline (1n)

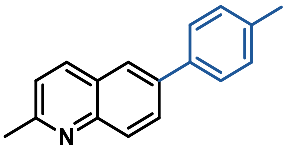

According to **General Procedure A**, the following compound was synthesized using 6-bromo-2-methylquinoline (1.0 equiv., 3.0 mmol, 666 mg,), *p*-tolylboronic acid (1.3 equiv., 3.9 mmol, 530 mg), Na_2CO_3 (7.5 equiv., 22.5 mmol, 2.38 g), $Pd(PPh_3)_4$ (3.0 mol%, 104 mg), toluene (10.0 mL), H_2O (10.0 mL), EtOH (2.5 mL). Purified by flash column chromatography (SiO_2; 100:0 to 85:15 pentane/EtOAc) to afford compound **1n** (504 mg, 72%) as an off white solid.

TLC: R_f = 0.2 (90:10 pentane/EtOAc).

NMR Spectroscopy:

^{1}H NMR (400 MHz, $CDCl_3$): δ_H 8.09 – 8.05 (m, 2H), 7.97 – 7.92 (m, 2H), 7.63 – 7.58 (m, 2H), 7.32 – 7.28 (m, 3H), 2.76 (s, 3H), 2.42 (s, 3H) ppm.

^{13}C NMR (101 MHz, $CDCl_3$): δ_C 159.0, 147.3, 138.5, 137.7, 137.5, 136.4, 129.8, 129.2, 129.1, 127.3, 126.8, 125.0, 122.5, 25.5, 21.3 ppm.

HRMS (ESI$^+$): m/z calc'd for $C_{17}H_{15}NNa$ [M+Na]$^+$: 256.10967, found: 256.10961.

2-methyl-6-(4-(4,4,5,5-tetramethyl-1,3,2-dioxaborolan-2-yl)phenyl)quinoline (1o)

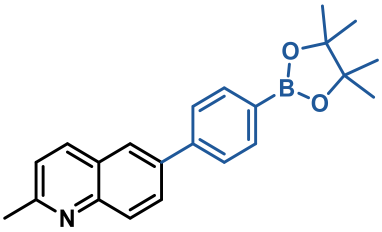

According to **General Procedure A**, the following compound was synthesized using 6-bromo-2-methylquinoline (1.0 equiv., 1.5 mmol, 333 mg), 1,4-bis(4,4,5,5-tetramethyl-1,3,2-dioxaborolan-2-yl)benzene (1.3 equiv., 1.95 mmol, 644 mg), Na_2CO_3 (7.5 equiv., 11.25 mmol, 1.19 g), $Pd(PPh_3)_4$ (3.0 mol%, 52 mg), toluene (5.0 mL), H_2O (5.0 mL), EtOH (1.2 mL). Purified by flash column chromatography (SiO_2; 100:0 to 98:02 CH_2Cl_2/MeOH) to afford compound **1o** (176 mg, 34%) as a white solid.

TLC: R_f = 0.6 (98:02 CH_2Cl_2/MeOH).

NMR Spectroscopy:

^{1}H NMR (400 MHz, $CDCl_3$): δ_H 8.15 – 8.05 (m, 2H), 8.03 – 7.88 (m, 4H), 7.73 (d, *J* = 7.9 Hz, 2H), 7.32 (d, *J* = 8.4 Hz, 1H), 2.77 (s, 3H), 1.38 (s, 12H) ppm.

^{13}C NMR (101 MHz, $CDCl_3$): δ_C 159.3, 147.6, 143.2, 138.4, 136.6, 135.5, 129.2, 126.8, 125.6, 122.6, 84.0, 25.6, 25.0 ppm.

^{11}B NMR (128 MHz, $CDCl_3$): δ 31.80 ppm.

HRMS (ESI$^+$): m/z calc'd for $C_{22}H_{24}NO_2BNa$ $[M+Na]^+$: 368.17963, found: 368.18025.

6-(3-fluorophenyl)-2-methylquinoline (1p)

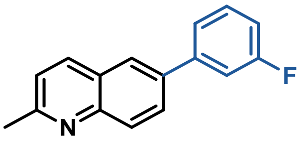

According to **General Procedure A**, the following compound was synthesized using 6-bromo-2-methylquinoline (1.0 equiv., 2.0 mmol, 444 mg), (3-fluorophenyl)boronic acid (1.3 equiv., 2.6 mmol, 364 mg), Na_2CO_3 (7.5 equiv., 15.0 mmol, 1.59 g), $Pd(PPh_3)_4$ (3.0 mol%, 69 mg), toluene (6.6 mL), H_2O (6.6 mL), EtOH (1.6 mL). Purified by flash column chromatography (SiO_2; 100:0 to 85:15 pentane/EtOAc) to afford compound **1p** (294 mg, 62%) as a light-yellow solid.

TLC: R_f = 0.2 (90:10 pentane/EtOAc).

NMR Spectroscopy:

^{1}H NMR (400 MHz, $CDCl_3$): δ_H 8.09 (d, *J* = 2.4 Hz, 1H), 8.07 (d, *J* = 2.0 Hz, 1H), 7.98 – 7.83 (m, 2H), 7.52 – 7.36 (m, 3H), 7.31 (d, *J* = 8.4 Hz, 1H), 7.12 – 7.02 (m, 1H), 2.76 (s, 3H) ppm.

^{13}C{^{19}F} NMR (126 MHz, $CDCl_3$): δ_C 163.4, 159.5, 147.6, 142.8, 137.2, 136.5, 130.5, 129.4, 128.9, 126.7, 125.6, 123.1, 122.7, 114.5, 114.3, 25.5 ppm.

^{19}F NMR (376 MHz, $CDCl_3$): δ_F -112.78 ppm

HRMS (ESI$^+$): m/z calc'd for $C_{16}H_{13}NF$ $[M+H]^+$: 238.10265, found: 238.10264.

6-(2,4-difluorophenyl)-2-methylquinoline (1q)

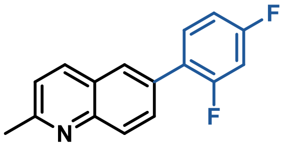

According to **General Procedure A**, the following compound was synthesized using 6-bromo-2-methylquinoline (1.0 equiv., 3.0 mmol, 666 mg), (2,4-difluorophenyl)boronic acid (1.3 equiv., 3.9 mmol, 616

mg), Na_2CO_3 (7.5 equiv., 22.5 mmol, 2.38 g), $Pd(PPh_3)_4$ (3.0 mol%, 104 mg), toluene (10.0 mL), H_2O (10.0 mL), EtOH (2.5 mL). Purified by flash column chromatography (SiO_2; 100:0 to 80:20 pentane/EtOAc) to afford compound **1q** (628 mg, 82%) as a yellowish solid.

TLC: R_f = 0.3 (70:30 pentane/EtOAc).

NMR Spectroscopy:

^{1}H NMR (400 MHz, $CDCl_3$): δ_H 8.12 – 8.04 (m, 2H), 7.91 – 7.86 (m, 1H), 7.82 (dt, *J* = 8.8, 1.9 Hz, 1H), 7.55 – 7.44 (m, 1H), 7.31 (d, *J* = 8.4 Hz, 1H), 7.03 – 6.89 (m, 2H), 2.77 (s, 3H) ppm.

^{13}C{^{19}F} NMR (126 MHz, $CDCl_3$): δ_C 162.6, 160.0, 159.6, 147.2, 136.5, 132.4, 131.7, 130.5, 128.9, 127.6, 126.5, 124.8, 122.6, 111.8, 104.6, 25.4 ppm.

^{19}F NMR (376 MHz, CDCl3): δ_F -110.50, -113.50 ppm.

HRMS (ESI$^+$): m/z calc'd for $C_{16}H_{12}F_2N$ $[M+H]^+$: 256.09323, found: 256.09317.

6-(3,5-dichlorophenyl)-2-methylquinoline (1r)

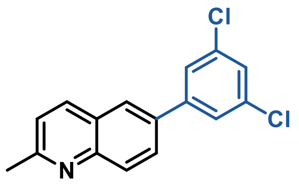

According to **General Procedure A**, the following compound was synthesized using 6-bromo-2-methylquinoline (1.0 equiv., 2.0 mmol, 444 mg), (3,5-dichlorophenyl)boronic acid (1.3 equiv., 2.6 mmol, 496 mg), Na_2CO_3 (7.5 equiv., 15.0 mmol, 1.59 g), $Pd(PPh_3)_4$ (3.0 mol%, 69 mg), toluene (6.6 mL), H_2O (6.6 mL), EtOH (1.6 mL). Purified by flash column chromatography (SiO_2; 100:0 to 75:25 pentane/EtOAc) to afford compound **1r** (225 mg, 39%) as a white solid.

TLC: R_f = 0.4 (70:30 pentane/EtOAc).

NMR Spectroscopy:

^{1}H NMR (400 MHz, $CDCl_3$): δ_H 8.13 – 8.02 (m, 2H), 7.89 (d, *J* = 2.1 Hz, 1H), 7.83 (dd, *J* = 8.8, 2.1 Hz, 1H), 7.55 (d, *J* = 1.9 Hz, 2H), 7.38 – 7.29 (m, 2H), 2.76 (s, 3H) ppm.

^{13}C NMR (101 MHz, $CDCl_3$): δ_C 160.0, 147.7, 143.6, 136.5, 135.8, 135.6, 129.6, 128.5, 127.5, 126.7, 126.0, 125.8, 122.9, 25.6 ppm.

HRMS (ESI$^+$): m/z calc'd for $C_{16}H_{12}Cl_2N$ $[M+H]^+$: 288.03413, found: 288.03046.

2-methyl-6-(pyridin-4-yl)quinoline (1s)

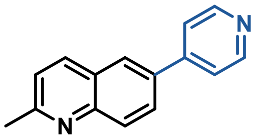

According to **General Procedure A**, the following compound was synthesized using 6-bromo-2-methylquinoline (1.0 equiv., 4.0 mmol, 888 mg), pyridin-4-ylboronic acid (1.3 equiv., 5.2 mmol, 639 mg), Na_2CO_3 (7.5 equiv., 30.0 mmol, 3.18 g), $Pd(PPh_3)_4$ (3.0 mol%, 139 mg), toluene (13.3 mL), H_2O (13.3 mL), EtOH (3.3 mL). Purified by flash column chromatography (SiO_2; 100:0 to 20:80 pentane/EtOAc) to afford compound **1s** (485 mg, 55%) as a yellowish solid.

TLC: R_f = 0.1 (40:60 pentane/EtOAc).

NMR Spectroscopy:

^{1}H NMR (400 MHz, $CDCl_3$): δ_H 8.70 – 8.63 (m, 2H), 8.10 (dd, *J* = 8.5, 4.5 Hz, 2H), 8.01 (d, *J* = 2.1 Hz, 1H), 7.93 (dd, *J* = 8.8, 2.1 Hz, 1H), 7.62 – 7.58 (m, 2H), 7.33 (d, *J* = 8.4 Hz, 1H), 2.76 (s, 3H) ppm.

^{13}C NMR (101 MHz, $CDCl_3$): δ_C 160.2, 150.5, 148.1, 147.7, 136.6, 135.4, 129.8, 128.2, 126.7, 126.0, 122.9, 121.9, 25.6 ppm.

HRMS (ESI$^+$): m/z calc'd for $C_{15}H_{12}N_2Na$ $[M+Na]^+$: 243.08927, found: 243.08929.

2-methyl-6-(1-methyl-1*H*-pyrazol-5-yl)quinoline (1t)

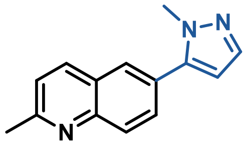

According to **General Procedure A**, the following compound was synthesized using 6-bromo-2-methylquinoline (1.0 equiv., 1.0 mmol, 222 mg), 1-methyl-5-(4,4,5,5-tetramethyl-1,3,2-dioxaborolan-2-yl)-1H-pyrazole (1.3 equiv., 1.3 mmol, 270 mg), Na_2CO_3 (7.5 equiv., 7.5 mmol, 795 mg), $Pd(PPh_3)_4$ (3.0 mol%, 35 mg), toluene (3.3 mL), H_2O (3.3 mL), EtOH (0.8 mL). Purified by flash column chromatography (SiO_2; 100:0 to 30:70 pentane/EtOAc) to afford compound **1t** (183 mg, 82%) as a yellowish solid.

TLC: R_f = 0.2 (30:70 pentane/EtOAc).

NMR Spectroscopy:

^{1}H NMR (400 MHz, $CDCl_3$): δ_H 8.09 – 8.01 (m, 2H), 7.77 (d, *J* = 2.1 Hz, 1H), 7.68 (dd, *J* = 8.7, 2.0 Hz, 1H), 7.52 (d, *J* = 1.9 Hz, 1H), 7.31 (d, *J* = 8.4 Hz, 1H), 6.37 (d, *J* = 1.9 Hz, 1H), 3.92 (s, 3H), 2.74 (s, 3H) ppm.

^{13}C NMR (101 MHz, $CDCl_3$): δ_C 160.0, 147.4, 143.0, 138.7, 136.3, 130.0, 129.2, 128.1, 127.5, 126.3, 122.9, 106.5, 37.7, 25.4 ppm.

HRMS (ESI$^+$): m/z calc'd for $C_{14}H_{14}N_3$ $[M+H]^+$: 224.11822, found: 224.11812.

4-(2-methylquinolin-6-yl)thiazole (1u)

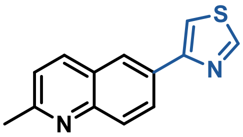

According to **General Procedure B**, the following compound was synthesized using 4-bromothiazole (1.0 equiv., 2.0 mmol, 328 mg), **S1** (1.3 equiv., 2.6 mmol, 700 mg), Na_2CO_3 (7.5 equiv., 15.0 mmol, 1.59 g), $Pd(PPh_3)_4$ (3.0 mol%, 69 mg), toluene (6.6 mL), H_2O (6.6 mL), EtOH (1.6 mL). Purified by flash column chromatography (SiO_2; 100:0 to 50:50 pentane/EtOAc) to afford compound **1u** (357 mg, 79%) as a yellowish solid.

TLC: R_f = 0.4 (50:50 pentane/EtOAc).

NMR Spectroscopy:

^{1}H NMR (400 MHz, $CDCl_3$): δ_H 8.94 – 8.86 (m, 1H), 8.43 – 8.33 (m, 1H), 8.15 (dd, *J* = 8.8, 1.9 Hz, 1H), 8.09 – 8.03 (m, 2H), 7.68 – 7.57 (m, 1H), 7.31 – 7.23 (m, 1H), 2.73 (s, 3H) ppm.

^{13}C NMR (126 MHz, $CDCl_3$): δ_C 159.4, 155.8, 153.2, 147.5, 136.7, 131.5, 129.2, 128.2, 126.8, 125.2, 122.7, 113.4, 25.5 ppm.

HRMS (ESI$^+$): m/z calc'd for $C_{13}H_{10}N_2SNa$ $[M+Na]^+$: 249.04569, found: 249.04563.

6-(2-chloropyrimidin-5-yl)-2-methylquinolinen (1v)

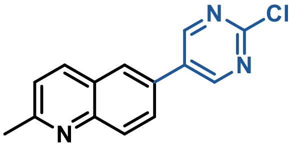

According to **General Procedure B**, the following compound was synthesized using 5-bromo-2-chloropyrimidine (1.0 equiv., 2.0 mmol, 387 mg), **S1** (1.3 equiv., 2.6 mmol, 700 mg), Na_2CO_3 (7.5 equiv., 15.0 mmol, 1.59 g), $Pd(PPh_3)_4$ (3.0 mol%, 69 mg), toluene (6.6 mL), H_2O (6.6 mL), EtOH (1.6 mL). Purified by flash column chromatography (SiO_2; 100:0 to 40:60 pentane/EtOAc) to afford compound **1v** (358 mg, 70%) as a yellowish solid.

TLC: R_f = 0.2 (50:50 pentane/EtOAc).

NMR Spectroscopy:

^{1}H NMR (400 MHz, $CDCl_3$): δ_H 8.95 (s, 2H), 8.17 (d, *J* = 8.8 Hz, 1H), 8.14 (d, *J* = 8.5 Hz, 1H), 7.96 (d, *J* = 2.1 Hz, 1H), 7.85 (dd, *J* = 8.7, 2.2 Hz, 1H), 7.39 (d, *J* = 8.4 Hz, 1H), 2.79 (s, 3H) ppm.

^{13}C NMR (101 MHz, $CDCl_3$): δ_C 160.7, 160.6, 157.7, 148.0, 136.5, 132.7, 130.5, 130.3, 127.8, 126.8, 126.1,

123.3, 25.7 ppm.

HRMS (ESI$^+$): m/z calc'd for $C_{14}H_{11}ClN_3$ [M+H]$^+$: 256.06360, found: 256.06356.

6-bromo-2-ethyl-3-methylquinoline (S2)

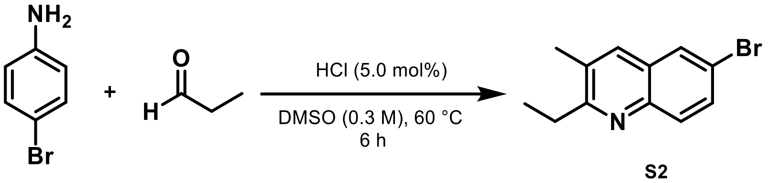

S2

Following a literature procedure,[3] 4-bromoaniline (1.0 equiv., 10.0 mmol, 1.72 g) and propionaldehyde (2.5 equiv., 25.0 mmol, 1.45 g) were dissolved in DMSO (30.0 mL), added HCl (4 M in dioxane, 5.0 mol%). The reaction mixture was stirred for 6 h at 60 °C. After completion, the reaction was quenched with aqueous $NaHCO_3$ solution. The solution was extracted with EtOAc, the combined organic layers were dried over $MgSO_4$, and the solvent was removed under reduced pressure. The crude product was purified by flash column chromatography (SiO_2; 100:0 to 95:05 pentane/EtOAc) to afford **S2** (1.0 g, 40%) as a yellow solid.

TLC: R_f = 0.7 (90:10 pentane/EtOAc).

NMR Spectroscopy:

^{1}H NMR (400 MHz, $CDCl_3$): δ_H 7.87 (d, J = 8.9 Hz, 1H), 7.84 (d, J = 2.2 Hz, 1H), 7.71 (s, 1H), 7.66 (dd, J = 9.0, 2.2 Hz, 1H), 2.96 (q, J = 7.5 Hz, 2H), 2.47 (d, J = 1.1 Hz, 3H), 1.36 (t, J = 7.5 Hz, 3H) ppm.

^{13}C NMR (101 MHz, $CDCl_3$): δ_C 163.9, 145.4, 134.7, 131.8, 130.7, 130.5, 128.8, 128.6, 119.4, 29.6, 19.3, 12.7 ppm.

HRMS (ESI$^+$): m/z calc'd for $C_{12}H_{13}NBr$ [M+H]$^+$: 250.02259, found: 250.02261.

2-ethyl-3-methyl-6-phenylquinoline (1x)

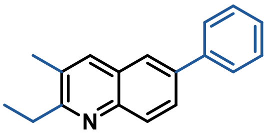

According to **General Procedure A**, the following compound was synthesized using **S2** (1.0 equiv., 2.0 mmol, 500 mg), phenylboronic acid (1.3 equiv., 2.6 mmol, 317 mg), Na_2CO_3 (7.5 equiv., 15.0 mmol, 1.59 g), $Pd(PPh_3)_4$ (3.0 mol%, 69 mg), toluene (6.6 mL), H_2O (6.6 mL), EtOH (1.6 mL). Purified by flash column chromatography (SiO_2; 100:0 to 92:08 pentane/EtOAc) to afford compound **1x** (425 mg, 86%) as a yellow solid.

TLC: R_f = 0.6 (90:10 pentane/EtOAc).

NMR Spectroscopy:

^{1}H NMR (400 MHz, $CDCl_3$): δ_H 8.17 – 8.03 (m, 1H), 7.93 – 7.86 (m, 3H), 7.77 – 7.68 (m, 2H), 7.58 – 7.44 (m,

2H), 7.44 – 7.34 (m, 1H), 3.02 (q, J = 7.5 Hz, 2H), 2.51 (s, 3H), 1.39 (t, J = 7.6 Hz, 3H) ppm.

^{13}C NMR (101 MHz, $CDCl_3$): δ_C 163.5, 146.2, 140.9, 138.5, 136.1, 130.0, 129.1, 129.0, 128.1, 127.6, 127.6, 127.5, 124.6, 29.7, 19.3, 13.0 ppm.

HRMS (ESI$^+$): m/z calc'd for $C_{18}H_{17}NNa$ [M+Na]$^+$: 270.12532, found: 270.12533.

4-chloro-6-phenylquinoline (1y)

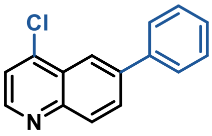

According to **General Procedure A**, the following compound was synthesized using 6-bromo-4-chloroquinoline (1.0 equiv., 3.0 mmol, 727 mg), phenylboronic acid (1.3 equiv., 3.9 mmol, 475 mg), Na_2CO_3 (7.5 equiv., 22.5 mmol, 2.38 g), $Pd(PPh_3)_4$ (3.0 mol%, 104 mg), toluene (10.0 mL), H_2O (10.0 mL), EtOH (2.5 mL). Purified by flash column chromatography (SiO_2; 100:0 to 75:25 pentane/EtOAc) to afford compound **1y** (309 mg, 43%) as a yellowish solid.

TLC: R_f = 0.2 (80:20 pentane/EtOAc).

NMR Spectroscopy:

^{1}H NMR (400 MHz, $CDCl_3$): δ_H 8.77 (d, J = 4.7 Hz, 1H), 8.40 (d, J = 2.0 Hz, 1H), 8.19 (d, J = 8.7 Hz, 1H), 8.03 (dd, J = 8.7, 2.0 Hz, 1H), 7.79 – 7.67 (m, 2H), 7.59 – 7.48 (m, 3H), 7.46 – 7.39 (m, 1H) ppm.

^{13}C NMR (101 MHz, $CDCl_3$): δ_C 149.8, 148.6, 142.8, 140.6, 140.1, 130.5, 130.2, 129.2, 128.2, 127.7, 126.8, 121.9, 121.7 ppm.

HRMS (ESI$^+$): m/z calc'd for $C_{15}H_{11}ClN$ [M+H]$^+$: 240.05745, found: 240.05742.

7-phenylquinoline (1z)

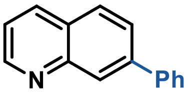

According to **General Procedure A**, the following compound was synthesized using 7-bromoquinoline (1.0 equiv., 2.0 mmol, 416 mg), phenylboronic acid (1.3 equiv., 2.6 mmol, 317 mg), Na_2CO_3 (7.5 equiv., 15.0 mmol, 1.59 g), $Pd(PPh_3)_4$ (3.0 mol%, 69 mg), toluene (6.6 mL), H_2O (6.6 mL), EtOH (1.6 mL). Purified by flash column chromatography (SiO_2; 100:0 to 80:20 pentane/EtOAc) to afford compound **1z** (337 mg, 82%) as a yellow oil.

TLC: R_f = 0.1 (90:10 pentane/EtOAc).

NMR Spectroscopy:

^{1}H NMR (400 MHz, $CDCl_3$): δ_H 8.94 (dd, J = 4.4, 1.7 Hz, 1H), 8.35 (s, 1H), 8.22 – 8.11 (m, 1H), 7.88 (d, J =

8.5 Hz, 1H), 7.82 (dd, J = 8.5, 1.8 Hz, 1H), 7.79 – 7.73 (m, 2H), 7.54 – 7.45 (m, 2H), 7.45 – 7.36 (m, 2H) ppm.

^{13}C NMR (101 MHz, $CDCl_3$): δ_C 151.0, 148.6, 142.3, 140.4, 135.9, 129.1, 128.3, 128.0, 127.6, 127.5, 127.2, 126.4, 121.1 ppm.

HRMS (ESI$^+$): m/z calc'd for $C_{15}H_{12}N$ $[M+H]^+$: 206.09643, found: 206.09709.

4-(quinolin-7-yl)benzonitrile (1aa)

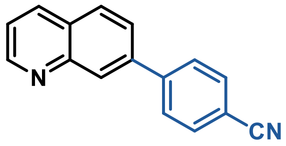

According to **General Procedure A**, the following compound was synthesized using 7-bromoquinoline (1.0 equiv., 1.5 mmol, 312 mg), (4-cyanophenyl)boronic acid (1.3 equiv., 1.95 mmol, 286 mg), Na_2CO_3 (7.5 equiv., 22.5 mmol, 1.19 g), $Pd(PPh_3)_4$ (3.0 mol%, 52 mg), toluene (5.0 mL), H_2O (5.0 mL), EtOH (1.2 mL). Purified by flash column chromatography (SiO_2; 100:0 to 40:60 pentane/EtOAc) to afford compound **1aa** (287 mg, 83%) as a pale-yellow solid.

TLC: R_f = 0.1 (80:20 pentane/EtOAc).

NMR Spectroscopy:

^{1}H NMR (400 MHz, $CDCl_3$): δ_H 8.98 (dd, J = 4.3, 1.8 Hz, 1H), 8.34 (d, J = 1.8 Hz, 1H), 8.27 – 8.16 (m, 1H), 7.94 (d, J = 8.5 Hz, 1H), 7.85 (d, J = 8.4 Hz, 2H), 7.79 (d, J = 8.3 Hz, 3H), 7.46 (dd, J = 8.3, 4.2 Hz, 1H) ppm.

^{13}C NMR (101 MHz, $CDCl_3$): δ_C 151.5, 148.5, 144.9, 140.2, 136.0, 132.9, 128.9, 128.2, 128.2, 128.1, 125.7, 121.9, 118.9, 111.7 ppm.

HRMS (ESI$^+$): m/z calc'd for $C_{16}H_{10}N_2Na$ $[M+Na]^+$: 253.07362, found: 253.07366.

7-(4-chlorophenyl)quinoline (1ab)

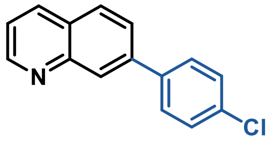

According to **General Procedure A**, the following compound was synthesized using 7-bromoquinoline (1.0 equiv., 1.5 mmol, 312 mg), (4-chlorophenyl)boronic acid (1.3 equiv., 1.95 mmol, 305 mg), Na_2CO_3 (7.5 equiv., 22.5 mmol, 1.19 g), $Pd(PPh_3)_4$ (3.0 mol%, 52 mg), toluene (5.0 mL), H_2O (5.0 mL), EtOH (1.2 mL). Purified by flash column chromatography (SiO_2; 100:0 to 70:30 pentane/EtOAc) to afford compound **1ab** (280 mg, 78%) as a yellow solid.

TLC: R_f = 0.4 (60:40 pentane/EtOAc).

NMR Spectroscopy:

^{1}H NMR (400 MHz, $CDCl_3$): δ_H 8.95 (dd, J = 4.3, 1.7 Hz, 1H), 8.33 (d, J = 1.8 Hz, 1H), 8.27 – 8.15 (m, 1H), 7.91 (d, J = 8.5 Hz, 1H), 7.79 (dd, J = 8.5, 1.8 Hz, 1H), 7.72 – 7.63 (m, 2H), 7.52 – 7.37 (m, 3H) ppm.

^{13}C NMR (101 MHz, $CDCl_3$): δ_C 150.8, 148.2, 141.4, 138.8, 136.4, 134.4, 129.4, 128.9, 128.6, 127.7, 126.9, 126.2, 121.3 ppm.

HRMS (ESI$^+$): m/z calc'd for $C_{15}H_{10}NClNa$ $[M+Na]^+$: 262.03940, found: 262.03939.

7-(4-(*tert*-butyl)phenyl)-2-methylquinoline (1ac)

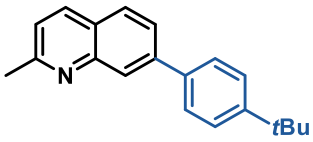

Following the modified literature procedure,[1] 7-chloro-2-methylquinoline (1.0 equiv., 1.5 mmol, 266 mg), 2-(4-(*tert*-butyl)phenyl)-4,4,5,5-tetramethyl-1,3,2-dioxaborolane (1.3 equiv., 1.95 mmol, 507 mg), K_2CO_3 (3.3 equiv., 4.95 mmol, 684 mg) and $Pd(dppf)Cl_2.CH_2Cl_2$ (10.0 mol%, 122 mg) were added to an oven-dried 10.0 mL Schlenk tube with a Teflon-coated magnetic stir bar. The tube was evacuated and backfilled with argon three times. Under an argon atmosphere, DMF (4.0 mL) was added. The mixture was stirred at 100 °C overnight. After the completion, ice cold water was added to the mixture and aqueous layer was extracted with ethyl acetate. Then dried over anhydrous $MgSO_4$ and the solvent was removed under reduced pressure. Purified by flash column chromatography (SiO_2; 100:0 to 85:15 pentane/EtOAc) to afford compound **1ac** (173 mg, 42%) as a white solid.

TLC: R_f = 0.5 (80:20 pentane/EtOAc).

NMR Spectroscopy:

^{1}H NMR (400 MHz, $CDCl_3$): δ_H 8.33 (s, 1H), 8.09 (d, J = 8.4 Hz, 1H), 7.87 – 7.76 (m, 2H), 7.75 – 7.69 (m, 2H), 7.55 – 7.50 (m, 2H), 7.29 (d, J = 8.4 Hz, 1H), 2.79 (s, 3H), 1.39 (s, 9H) ppm.

^{13}C NMR (101 MHz, $CDCl_3$): δ_C 159.4, 151.2, 147.9, 142.4, 137.4, 136.5, 128.0, 127.2, 126.1, 125.7, 125.6, 121.9, 34.8, 31.5, 25.3 ppm.

HRMS (ESI$^+$): m/z calc'd for $C_{20}H_{21}NNa$ $[M+Na]^+$: 298.15662, found: 298.15652.

Synthesis of 7-chloro-4-methoxyquinoline (S3)

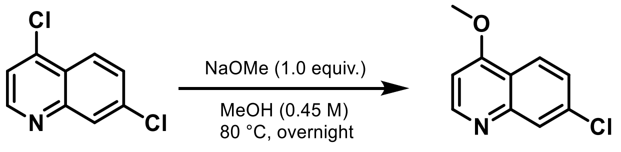

S3

Following a literature procedure,[4] to an oven-dried 150.0 mL Schlenk tube with a Teflon-coated magnetic stir

bar, 4,7-dichloroquinoline (1.0 equiv., 22.3 mmol, 4.42 g) and sodium methoxide (1.0 equiv., 22.3 mmol, 1.20 g) were added. The tube was evacuated and backfilled with argon three times and 50.0 mL of MeOH was added to the Schlenk tube under an argon atmosphere. The reaction was refluxed overnight. The solvent evaporated, ethyl acetate and water were added. The phases were separated and the aqueous layer was extracted with ethyl acetate. Purified by flash column chromatography on silica gel (SiO_2; 100:0 to 70:30 pentane/EtOAc) to afford the compound **S3** (4.01 g, 93%) as a white solid.

TLC: R_f = 0.2 (70:30 pentane/EtOAc).

NMR Spectroscopy:

^{1}H NMR (400 MHz, $CDCl_3$): δ_H 8.74 (d, J = 5.2 Hz, 1H), 8.13 (d, J = 8.9 Hz, 1H), 8.02 (d, J = 2.1 Hz, 1H), 7.44 (dd, J = 9.0, 2.1 Hz, 1H), 6.73 (d, J = 5.4 Hz, 1H), 4.04 (s, 3H) ppm.

^{13}C NMR (101 MHz, $CDCl_3$): δ_C 162.5, 152.7, 149.9, 135.9, 128.1, 126.7, 123.5, 120.0, 100.5, 55.9 ppm.

HRMS (ESI$^+$): m/z calc'd for $C_{10}H_9ClNO$ $[M+H]^+$: 194.03672, found: 194.03661.

Synthesis of 4-methoxy-7-phenylquinoline (1ad)

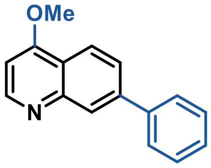

Following the modified literature procedure,[1] **S3** (1.0 equiv., 3.0 mmol, 581 mg), phenylboronic acid (1.3 equiv., 3.9 mmol, 475 mg), K_2CO_3(3.0 equiv., 9.0 mmol, 1.24 g) and Pd(dppf)Cl_2 (5.0 mol%, 110 mg) were added to an oven-dried 50.0 mL Schlenk tube with a Teflon-coated magnetic stir bar. The tube was evacuated and backfilled with argon three times. Under argon, DMF (8.0 mL) was added. The mixture was stirred at 100 °C overnight. After completion, 30.0 mL of ice-cold water was added to the mixture, and the aqueous layer was extracted with ethyl acetate. Then the organic layer was dried over anhydrous $MgSO_4$ and the solvent was removed under reduced pressure. Purified by flash column chromatography (SiO_2; 100:0 to 50:50 pentane/EtOAc) to afford the compound **1ad** (423 mg, 60%) as a yellow oil.

TLC: R_f = 0.2 (60:40 pentane/EtOAc).

NMR Spectroscopy:

^{1}H NMR (400 MHz, $CDCl_3$): δ_H 8.78 (d, J = 5.3 Hz, 1H), 8.30 – 8.22 (m, 2H), 7.76 (ddd, J = 8.1, 6.6, 1.5 Hz, 3H), 7.54 – 7.45 (m, 2H), 7.45 – 7.35 (m, 1H), 6.73 (d, J = 5.2 Hz, 1H), 4.05 (s, 3H) ppm.

^{13}C NMR (101 MHz, $CDCl_3$): δ_C 162.5, 152.0, 149.6, 142.6, 140.5, 129.1, 128.0, 127.6, 126.7, 125.3, 122.5, 120.6, 100.2, 55.9 ppm.

HRMS (ESI$^+$): m/z calc'd for $C_{16}H_{14}NO$ $[M+H]^+$: 236.10699, found: 236.10686.

6-phenylisoquinoline (1ae)

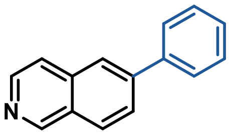

According to **General Procedure A**, the following compound was synthesized using 6-bromoisoquinoline (1.0 equiv., 2.0 mmol, 416 mg), phenylboronic acid (1.3 equiv., 2.6 mmol, 317 mg), Na_2CO_3 (7.5 equiv., 15.0 mmol, 1.59 g), $Pd(PPh_3)_4$ (3.0 mol%, 69 mg), toluene (6.6 mL), H_2O (6.6 mL), EtOH (1.6 mL). Purified by flash column chromatography (SiO_2; 100:0 to 50:50 pentane/EtOAc) to afford compound **1ae** (386 mg, 94%) as a yellow solid.

TLC: R_f = 0.2 (70:30 pentane/EtOAc).

NMR Spectroscopy:

^{1}H NMR (400 MHz, $CDCl_3$): δ_H 9.29 – 9.27 (m, 1H), 8.56 (d, *J* = 5.8 Hz, 1H), 8.09 – 8.02 (m, 1H), 8.03 – 7.98 (m, 1H), 7.87 (dd, *J* = 8.5, 1.8 Hz, 1H), 7.74 – 7.68 (m, 3H), 7.54 – 7.49 (m, 2H), 7.46 – 7.41 (m, 1H) ppm.

^{13}C NMR (101 MHz, $CDCl_3$): δ_C 152.5, 143.6, 143.3, 140.4, 136.3, 129.2, 128.3, 128.3, 127.9, 127.8, 127.3, 124.4, 120.8 ppm.

HRMS (ESI$^+$): m/z calc'd for $C_{15}H_{11}NNa$ $[M+Na]^+$: 228.07837, found: 228.07837.

6-(4-(trifluoromethyl)phenyl)isoquinoline (1af)

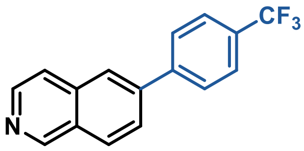

According to **General Procedure A**, the following compound was synthesized using 6-bromoisoquinoline (1.0 equiv., 2.0 mmol, 416 mg), (4-(trifluoromethyl)phenyl)boronic acid (1.3 equiv., 2.6 mmol, 494 mg), Na_2CO_3 (7.5 equiv., 15.0 mmol, 1.59 g), $Pd(PPh_3)_4$ (3.0 mol%, 69 mg), toluene (6.6 mL), H_2O (6.6 mL), EtOH (1.6 mL). Purified by flash column chromatography (SiO_2; 100:0 to 70:30 pentane/EtOAc) to afford compound **1af** (399 mg, 73%) as a yellow solid.

TLC: R_f = 0.3 (60:40 pentane/EtOAc).

NMR Spectroscopy:

^{1}H NMR (599 MHz, $CDCl_3$): δ_H 9.30 (s, 1H), 8.58 (d, *J* = 5.7 Hz, 1H), 8.07 (d, *J* = 8.5 Hz, 1H), 8.01 (d, *J* = 1.7 Hz, 1H), 7.84 (dd, *J* = 8.5, 1.8 Hz, 1H), 7.82 – 7.79 (m, 2H), 7.77 – 7.74 (m, 2H), 7.73 – 7.69 (m, 1H) ppm.

^{13}C{^{19}F} NMR (151 MHz, $CDCl_3$): δ_C 152.4, 143.8, 143.7, 141.7, 136.2, 130.4, 128.7, 128.1, 128.1, 126.9, 126.1, 125.0, 124.3, 120.9 ppm.

^{19}F NMR (564 MHz, $CDCl_3$): δ_F -62.54 ppm.

HRMS (ESI^+): m/z calc'd for $C_{16}H_{11}NF_3$ $[M+H]^+$: 274.08381, found: 274.08381.

4-(quinazolin-6-yl)benzonitrile (1ag)

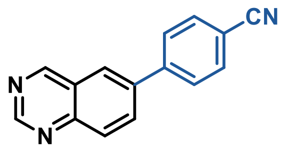

According to **General Procedure A** the following compound was synthesized using 6-bromoquinazoline (1.0 equiv., 1.0 mmol, 209 mg), 4-cyanophenylboronic acid (1.3 equiv., 1.3 mmol, 191 mg), Na_2CO_3 (7.5 equiv., 7.5 mmol, 795 mg), $Pd(PPh_3)_4$ (3.0 mol%, 35 mg), toluene (3.3 mL), H_2O (3.3 mL), EtOH (0.8 mL). Purified by flash column chromatography (SiO_2; 100:0 to 40:60 pentane/EtOAc) to afford compound **1ag** (220 mg, 95%) as a brownish yellow solid.

TLC: R_f = 0.2 (50:50 pentane/EtOAc).

NMR Spectroscopy:

^{1}H NMR (400 MHz, $CDCl_3$): δ_H 9.48 (s, 1H), 9.36 (s, 1H), 8.19 – 8.09 (m, 3H), 7.80 (br. s, 4H) ppm.

^{13}C NMR (101 MHz, $CDCl_3$): δ_C 160.7, 155.9, 149.9, 143.9, 138.9, 133.4, 133.0, 129.6, 128.2, 125.6, 125.3, 118.6, 112.2 ppm.

HRMS (ESI^+): m/z calc'd for $C_{15}H_9N_3Na$ $[M+Na]^+$: 254.06887, found: 254.06878.

6-(4-(trifluoromethyl)phenyl)quinazoline (1ah)

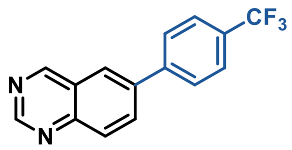

According to **General Procedure A**, the following compound was synthesized using 6-bromoquinazoline (1.0 equiv., 1.0 mmol, 209 mg), 4-(trifluormethyl)phenylboronic acid (1.3 equiv., 1.3 mmol, 247 mg), Na_2CO_3 (7.5 equiv., 7.5 mmol, 795 mg), $Pd(PPh_3)_4$ (3.0 mol%, 35 mg), toluene (3.3 mL), H_2O (3.3 mL), EtOH (0.8 mL). Purified by flash column chromatography (SiO_2; 100:0 to 65:35 pentane/EtOAc) to afford compound **1ah** (219 mg, 80%) as a yellowish solid.

TLC: R_f = 0.3 (60:40 pentane/EtOAc).

NMR Spectroscopy:

^{1}H NMR (400 MHz, $CDCl_3$): δ_H 9.47 (s, 1H), 9.35 (s, 1H), 8.18 – 8.08 (m, 3H), 7.88 – 7.67 (m, 4H) ppm.

^{13}C{^{19}F} NMR (126 MHz, $CDCl_3$): δ_C 160.6, 155.7, 149.8, 143.0, 139.4, 133.6, 130.5, 129.4, 127.9, 126.2,

125.3, 125.3, 124.2 ppm.

^{19}F NMR (376 MHz, CDCl3): δ_F -62.77 ppm.

HRMS (ESI$^+$): m/z calc'd for $C_{15}H_{10}F_3N_2$ [M+H]$^+$: 275.07906, found: 275.07898.

2-methyl-6-(*o*-tolyl)quinoline (1ai)

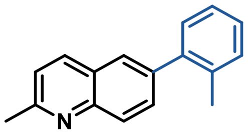

According to **General Procedure A**, the following compound was synthesized using 6-bromo-2-methylquinoline (1.0 equiv., 3.0 mmol, 666 mg), *o*-tolylboronic acid (1.3 equiv., 3.9 mmol, 530 mg), Na_2CO_3 (7.5 equiv., 22.5 mmol, 2.38 g), $Pd(PPh_3)_4$ (3.0 mol%, 104 mg), toluene (10.0 mL), H_2O (10.0 mL), EtOH (2.5 mL). Purified by flash column chromatography (SiO_2; 100:0 to 75:15 pentane/EtOAc) to afford compound **1ai** (602 mg, 86%) as a yellow oil.

TLC: R_f = 0.3 (80:20 pentane/EtOAc).

NMR Spectroscopy:

^{1}H NMR (400 MHz, $CDCl_3$): δ_H 8.10 – 8.02 (m, 2H), 7.72 – 7.64 (m, 2H), 7.35 – 7.27 (m, 5H), 2.78 (s, 3H), 2.31 (s, 3H) ppm.

^{13}C NMR (101 MHz, $CDCl_3$): δ_C 159.1, 147.0, 141.4, 139.5, 136.3, 135.7, 131.4, 130.6, 130.1, 128.3, 127.7, 127.5, 126.4, 126.0, 122.4, 25.5, 20.7 ppm.

GC-HRMS: m/z calc'd for $C_{17}H_{15}N$ [M]$^+$: 233.11990, found: 233.11194.

2-phenylnaphthalene (1aj)

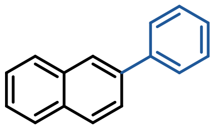

According to **General Procedure A**, the following compound was synthesized using 2-bromonaphthalene (1.0 equiv., 3.0 mmol, 621 mg), phenylboronic acid (1.3 equiv., 3.9 mmol, 475 mg), Na_2CO_3 (7.5 equiv., 22.5 mmol, 2.38 g), $Pd(PPh_3)_4$ (3.0 mol%, 104 mg), toluene (10.0 mL), H_2O (10.0 mL), EtOH (2.5 mL). Purified by flash column chromatography (SiO_2; 100:0 to 95:05 pentane/EtOAc) to afford compound **1aj** (502 mg, 82%) as a white solid.

TLC: R_f = 0.8 (90:10 pentane/EtOAc).

NMR Spectroscopy:

^{1}H NMR (400 MHz, $CDCl_3$): δ_H 8.09 – 8.04 (m, 1H), 7.96 – 7.83 (m, 3H), 7.81 – 7.68 (m, 3H), 7.55 – 7.47 (m, 4H), 7.43 – 7.36 (m, 1H) ppm.

^{13}C NMR (101 MHz, $CDCl_3$): δ_C 141.3, 138.7, 133.8, 132.7, 129.0, 128.6, 128.3, 127.8, 127.6, 127.5, 126.4, 126.1, 125.9, 125.7 ppm.

GC-HRMS: m/z calc'd for $C_{16}H_{12}$ $[M]^+$: 204.09335, found: 204.09317.

General Procedure C: Synthesis of Vinylcyclopropanes (2)

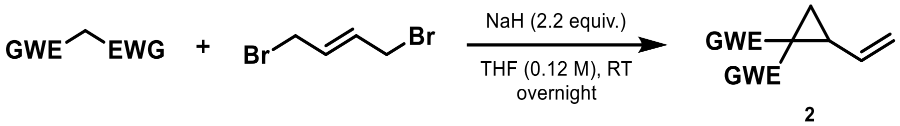

Following a literature procedure,[5] to an oven-dried 150.0 mL Schlenk tube with a Teflon-coated magnetic stir bar, (*E*)-1,4-dibromobut-2-ene (1.0 equiv.) and NaH (60% in mineral oil, 2.2 equiv.) were added. The tube was evacuated and backfilled with argon three times. THF (0.12 M) was added under an argon atmosphere. After that, malonate (either commercially available or synthesized, 1.0 equiv.) was added dropwise to the Schlenk tube at 0 °C. The reaction mixture was stirred at room temperature overnight. After consumption of all starting materials, the reaction was quenched with slow addition of saturated NH_4Cl solution at 0 °C. The aqueous layer was extracted with diethyl ether and dried over $MgSO_4$. The solvent was removed under reduced pressure and purified by flash column chromatography on silica gel to afford the corresponding VCP **2**.

Vinylcyclopropanes library

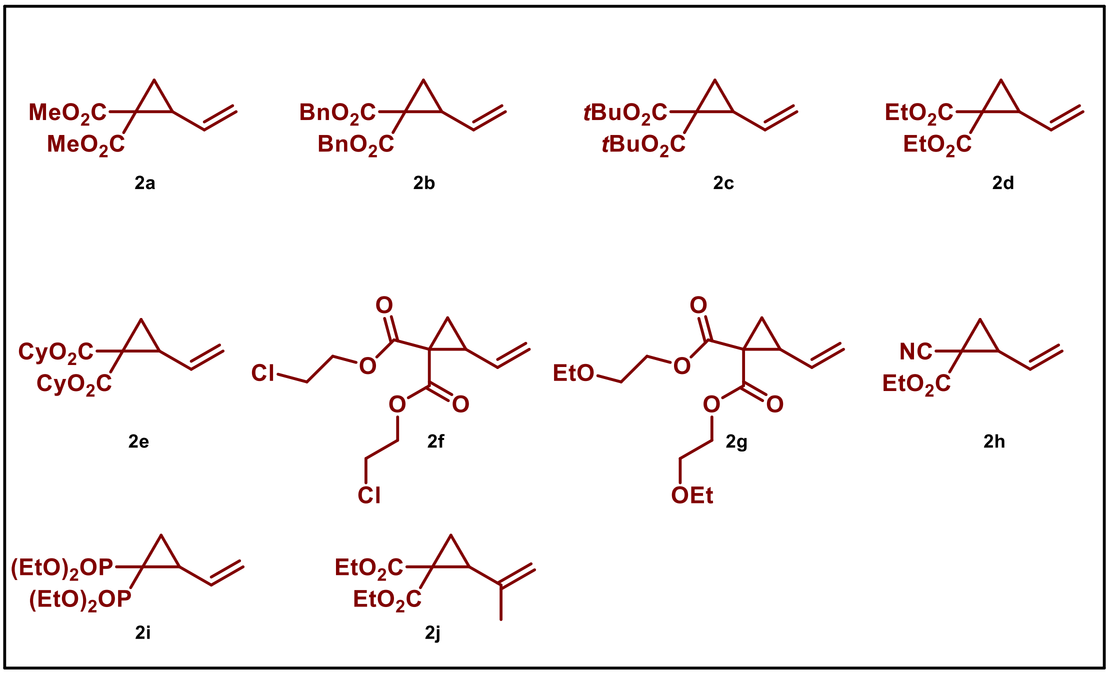

dimethyl 2-vinylcyclopropane-1,1-dicarboxylate (2a)

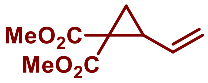

According to **General Procedure C**, the following compound was synthesized using (*E*)-1,4-dibromobut-2-ene (1.0 equiv., 10.0 mmol, 2.14 g), dimethyl malonate (1.0 equiv., 10.0 mmol, 1.32 g). Purified by flash column chromatography (SiO_2; 100:0 to 96:04 pentane/EtOAc) to afford the compound **2a** (1.53 g, 83%) as a colourless liquid.

TLC: R_f = 0.8 (90:10 pentane/EtOAc).

NMR Spectroscopy:

^{1}H NMR (400 MHz, $CDCl_3$): δ_H 5.43 (ddd, *J* = 16.8, 10.0, 8.2 Hz, 1H), 5.29 (dd, *J* = 17.1, 1.7 Hz, 1H), 5.19 – 5.09 (m, 1H), 3.74 (s, 6H), 2.64 – 2.51 (m, 1H), 1.72 (dd, *J* = 7.6, 4.9 Hz, 1H), 1.58 (dd, *J* = 9.0, 4.9 Hz, 1H) ppm.

^{13}C NMR (101 MHz, $CDCl_3$): δ_C 170.2, 167.9, 133.1, 118.8, 52.9, 52.7, 35.9, 31.6, 20.7 ppm.

HRMS (ESI^+): m/z calc'd for $C_9H_{12}O_4Na$ $[M+Na]^+$: 207.06278, found: 207:06257.

dibenzyl 2-vinylcyclopropane-1,1-dicarboxylate (2b)

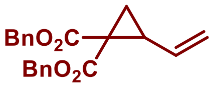

According to **General Procedure C**, the following compound was synthesized using (*E*)-1,4-dibromobut-2-ene (1.0 equiv., 10.0 mmol, 2.14 g), dibenzyl malonate (1.0 equiv., 10.0 mmol, 2.84 g). Purified by flash column chromatography (SiO_2; 100:0 to 95:05 pentane/EtOAc) to afford the compound **2b** (1.51 g, 45%) as a colourless liquid.

TLC: R_f = 0.7 (90:10 pentane/EtOAc).

NMR Spectroscopy:

^{1}H NMR (400 MHz, $CDCl_3$): δ_H 7.35 – 7.26 (m, 10H), 5.41 (ddd, *J* = 17.0, 10.1, 8.2 Hz, 1H), 5.28 (dd, *J* = 17.1, 1.7 Hz, 1H), 5.23 – 5.04 (m, 5H), 2.75 – 2.54 (m, 1H), 1.76 (dd, *J* = 7.6, 4.9 Hz, 1H), 1.61 (dd, *J* = 9.0, 4.9 Hz, 1H) ppm.

^{13}C NMR (101 MHz, $CDCl_3$): δ_C 169.5, 167.3, 135.6, 135.6, 133.0, 128.7, 128.6, 128.4, 128.4, 128.4, 128.2, 118.9, 67.5, 67.5, 36.1, 31.8, 20.9 ppm.

HRMS (ESI^+): m/z calc'd for $C_{21}H_{20}O_4Na$ $[M+Na]^+$: 359.12538, found: 359.12493.

di-*tert*-butyl 2-vinylcyclopropane-1,1-dicarboxylate (2c)

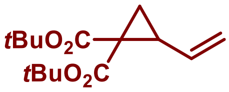

According to **General Procedure C**, the following compound was synthesized using (*E*)-1,4-dibromobut-2-ene (1.0 equiv., 10.0 mmol, 2.14 g), di-*tert*-butyl malonate (1.0 equiv., 10.0 mmol, 2.16 g). Purified by flash column chromatography (SiO_2; 100:0 to 98:02 pentane/EtOAc) to afford the compound **2c** (1.85 g, 69%) as a white solid.

TLC: R_f = 0.6 (95:05 pentane/EtOAc).

NMR Spectroscopy:

^{1}H NMR (400 MHz, $CDCl_3$): δ_H 5.43 (ddd, *J* = 17.2, 10.2, 8.3 Hz, 1H), 5.27 (ddd, *J* = 17.0, 1.7, 0.7 Hz, 1H), 5.10 (ddd, *J* = 10.2, 1.7, 0.7 Hz, 1H), 2.49 – 2.41 (m, 1H), 1.53 (dd, *J* = 7.3, 4.7 Hz, 1H), 1.47 (s, 9H), 1.45 (s, 9H), 1.37 (dd, *J* = 8.8, 4.8, 1H) ppm.

^{13}C NMR (101 MHz, $CDCl_3$): δ_C 169.2, 167.0, 133.7, 117.8, 81.8, 81.4, 37.8, 30.1, 28.2, 28.1, 19.8 ppm.

HRMS (ESI^+): m/z calc'd for $C_{15}H_{24}O_4Na$ $[M+Na]^+$: 291.15668, found: 291.15588.

diethyl 2-vinylcyclopropane-1,1-dicarboxylate (2d)

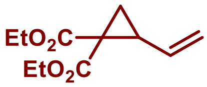

According to **General Procedure C**, the following compound was synthesized using (*E*)-1,4-dibromobut-2-ene (1.0 equiv., 10.0 mmol, 2.14 g), diethyl malonate (1.0 equiv., 10.0 mmol, 1.60 g). Purified by flash column chromatography (SiO_2; 100:0 to 92:08 pentane/EtOAc) to afford the compound **2d** (2.01 g, 95%) as a colourless liquid.

TLC: R_f = 0.5 (90:10 pentane/EtOAc).

NMR Spectroscopy:

^{1}H NMR (400 MHz, $CDCl_3$): δ_H 5.38 (ddd *J* = 16.8, 10.1, 8.3 Hz, 1H), 5.24 (dd, *J* = 17.1, 1.8 Hz, 1H), 5.08 (dd, *J* = 10.2, 1.8 Hz, 1H), 4.23 – 4.05 (m, 4H), 2.56 – 2.45 (m, 1H), 1.63 (dd, *J* = 7.5, 4.9 Hz, 1H), 1.49 (dd, *J* = 9.0, 4.9 Hz, 1H), 1.27 – 1.17 (m, 6H) ppm.

^{13}C NMR (101 MHz, $CDCl_3$): δ_C 169.6, 167.4, 133.2, 118.7, 61.6, 61.4, 35.9, 31.1, 20.3, 14.2, 14.1 ppm.

HRMS (ESI^+): m/z calc'd for $C_{11}H_{16}O_4Na$ $[M+Na]^+$: 235.09408, found: 235.09384.

dicyclohexyl 2-vinylcyclopropane-1,1-dicarboxylate (2e)

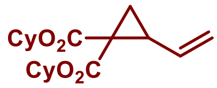

The title VCP **2e** was synthesized according to the literature procedure.[6] Spectroscopic data matches with the reported values.

Step 1: **Synthesis of bis(2-chloroethyl) malonate** (**S4**)

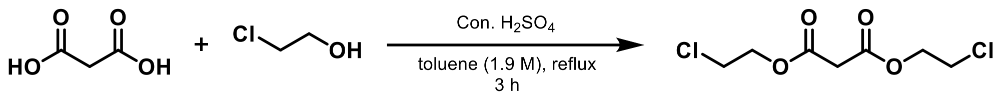

S4

Following the literature procedure,[7] malonic acid (1.0 equiv., 38.5 mmol, 4.00 g) was added to an oven-dried Schlenk tube with a Teflon-coated magnetic stir bar. The tube was evacuated and backfilled with argon three times. The anhydrous toluene (20.0 mL) was added to the tube under an argon atmosphere, followed by addition of 2-chloroethanol (5.4 equiv., 207.9 mmol, 13.9 mL), and concentrated H_2SO_4 (0.5 mL). Then the reaction was refluxed for 3 hours. The reaction mixture was allowed to cool to room temperature, after which it was quenched with slow addition of 10% Na_2CO_3 solution. The phases were separated and the aqueous layer was extracted with diethyl ether (3× 100.0 mL). The combined organic layer was washed with 5% $NaHCO_3$ (100.0 mL), H_2O (2 × 100.0 mL), brine (100.0 mL), and dried over anhydrous $MgSO_4$, filtered, and concentrated under reduced pressure to yield crude **S4** as a colourless oil.

Step 2: **bis(2-chloroethyl) 2-vinylcyclopropane-1,1-dicarboxylate (2f)**

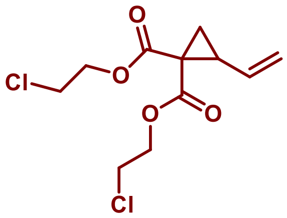

Without further purification of **S4** the following vinylcyclopropane was synthesized according to **General Procedure C**. After overnight stirring purified by flash column chromatography (SiO_2; 100:0 to 93:07 pentane/EtOAc) to afford the compound **2f** (4.33 g, 40%**)** as a liquid over two steps.

TLC: R_f = 0.6 (90:10 pentane/EtOAc).

NMR Spectroscopy:

^{1}H NMR (400 MHz, $CDCl_3$): δ_H 5.52 – 5.38 (m, 1H), 5.34 – 5.25 (m, 1H), 5.19 – 5.09 (m, 1H), 4.52 – 4.28 (m, 4H), 3.74 – 3.58 (m, 4H), 2.68 – 2.57 (m, 1H), 1.80 – 1.69 (m, 1H), 1.65 – 1.57 (m, 1H) ppm.

^{13}C NMR (101 MHz, $CDCl_3$): δ_C 169.0, 166.8, 132.5, 119.3, 65.1, 41.3, 35.7, 31.9, 20.8 ppm.

HRMS (ESI$^+$): m/z calc'd for $C_{11}H_{14}Cl_2O_4Na$ $[M+Na]^+$: 303.01614, found: 303.01617.

Step 1: **Synthesis of bis(2-ethoxyethyl) malonate (S5)**

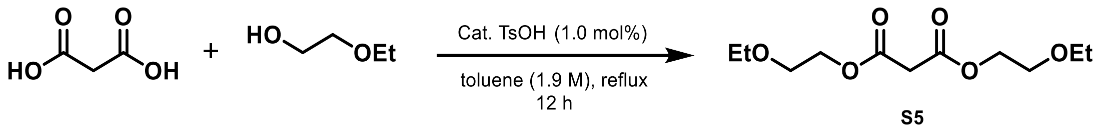

Following a literature procedure,[7] a mixture of malonic acid (1.0 equiv., 38.5 mmol, 4.00 g) and *p*-toluenesulfonic acid monohydrate (1.0 mol%, 0.4 mmol, 76 mg) was added to an oven-dried Schlenk tube with a Teflon-coated magnetic stir bar. The tube was evacuated and backfilled with argon three times. Anhydrous toluene (20.0 mL) was added to the tube under an argon atmosphere, followed by addition of 2-ethoxyethanol (2.0 equiv., 77.0 mmol, 7.5 mL). Then the reaction was refluxed for 12 hours. Afterwards, the reaction mixture was allowed to cool to room temperature. Then diethyl ether was added to the mixture. The organic layer was washed with 5% $NaHCO_3$ (100.0 mL), H_2O (2 × 100.0 mL), brine (100.0 mL), and dried over anhydrous $MgSO_4$, filtered, and concentrated under reduced pressure to yield crude **S5** as a colourless liquid.

Step: **2**: **bis(2-ethoxyethyl) 2-vinylcyclopropane-1,1-dicarboxylate (2g)**

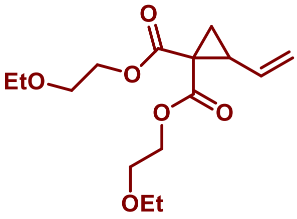

Without further purification of **S5** the following vinylcyclopropane was synthesized according to **General Procedure C**. Following overnight stirring, the crude mixture was purified by flash column chromatography (SiO_2; 100:0 to 85:15 pentane/EtOAc) to afford the compound **2g** (2.43 g, 21%) over two steps.

TLC: R_f = 0.5 (80:20 pentane/EtOAc).

NMR Spectroscopy:

^{1}**H NMR** (400 MHz, $CDCl_3$): δ_H 5.43 (ddd, *J* = 17.1, 10.2, 8.4 Hz, 1H), 5.25 (dd, *J* = 17.0, 1.6 Hz, 1H), 5.08 (dd, *J* = 10.1, 1.6 Hz, 1H), 4.32 – 4.12 (m, 4H), 3.66 – 3.53 (m, 4H), 3.52 – 3.43 (m, 4H), 2.56 (q, *J* = 8.3 Hz, 1H), 1.69 (dd, *J* = 7.6, 4.9 Hz, 1H), 1.55 (dd, *J* = 9.0, 4.9 Hz, 1H), 1.15 (t, *J* = 7.1 Hz, 6H) ppm.

^{13}**C NMR** (101 MHz, $CDCl_3$): δ_C 169.5, 167.2, 133.1, 118.6, 68.2, 68.0, 66.6, 66.5, 64.8, 64.5, 35.8, 31.6, 20.7, 15.1 ppm.

HRMS (ESI^+): m/z calc'd for $C_{15}H_{24}O_6Na$ $[M+Na]^+$: 323.14651, found: 323.14621.

ethyl 1-cyano-2-vinylcyclopropane-1-carboxylate (2h)

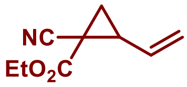

Synthesized following literature procedure,[8] using (*E*)-1,4-dibromobut-2-ene (1.0 equiv., 10.0 mmol, 2.14 g), ethyl 2-cyanoacetate (1.0 equiv., 10.0 mmol, 1.13 g). Purified by flash column chromatography (SiO_2; 100:0 to 90:10 pentane/EtOAc) to afford the compound **2h** (1.32 g, 80%) as a colourless liquid, mixture of inseparable diastereomers (d.r. = 73:27).

TLC: R_f = 0.5 (90:10 pentane/EtOAc).

NMR Spectroscopy:

^{1}H NMR (400 MHz, $CDCl_3$): δ_H 5.68 – 5.53 (m, 1H), 5.44 – 5.36 (m, 1H), 5.36 – 5.31 (m, 0.73H), 5.24 (dd, *J* = 10.3, 1.5 Hz, 0.27H), 4.29 – 4.11 (m, 2H), 2.62 – 2.46 (m, 1H), 1.97 – 1.82 (m, 1.27H), 1.62 (dd, *J* = 7.9, 5.0 Hz, 0.73H), 1.35 – 1.22 (m, 3H) ppm.

^{13}C NMR (101 MHz, $CDCl_3$): δ_C 167.1, 165.2, 132.2, 130.5, 121.3, 120.8, 118.7, 116.7, 62.8, 35.7, 33.7, 23.9, 22.5, 21.1, 20.3, 14.1, 14.1 ppm.

HRMS (ESI$^+$): m/z calc'd for $C_9H_{11}O_2Na$ $[M+Na]^+$: 188.06820, found: 188.06810.

tetraethyl (2-vinylcyclopropane-1,1-diyl)bis(phosphonate) (2i)

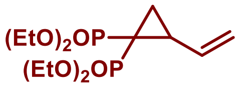

Synthesized following literature procedure,[9] using (*E*)-1,4-dibromobut-2-ene (1.0 equiv., 10.0 mmol, 2.14 g) and tetraethyl methylenebis(phosphonate) (1.0 equiv., 10.0 mmol, 2.88 g). Purified by flash column chromatography (SiO_2; 100:0 to 60:40 pentane/EtOAc) to afford the compound **2i** (2.55 g, 75%) as a yellow oil.

TLC: R_f = 0.6 (50:50 pentane/EtOAc).

NMR Spectroscopy:

^{1}H NMR (400 MHz, $CDCl_3$): δ_H 6.05 (dt, *J* = 17.0, 9.9 Hz, 1H), 5.35 (dd, *J* = 17.0, 1.7 Hz, 1H), 5.15 (dd, *J* = 10.2, 1.7 Hz, 1H), 4.23 – 3.98 (m, 8H), 2.63 – 2.42 (m, 1H), 1.74 – 1.53 (m, 2H), 1.38 – 1.22 (m, 12H) ppm.

^{13}C NMR (101 MHz, $CDCl_3$): δ_C 134.9 (dd, *J* = 4.9, 2.9 Hz), 118.3, 62.7 – 62.4 (m), 62.3 (d, *J* = 6.2 Hz), 28.8 – 28.4 (m), 17.5 (dd, *J* = 4.4, 3.1 Hz), 16.5 – 16.1 (m) ppm.

^{31}P NMR (162 MHz, $CDCl_3$): δ 23.92 (d, *J* = 21.5 Hz), 22.66 (d, *J* = 21.5 Hz) ppm.

HRMS (ESI$^+$): m/z calc'd for $C_{13}H_{26}O_6P_2Na$ $[M+Na]^+$: 363.10968, found: 363.10952.

diethyl 2-acetylcyclopropane-1,1-dicarboxylate (S6)

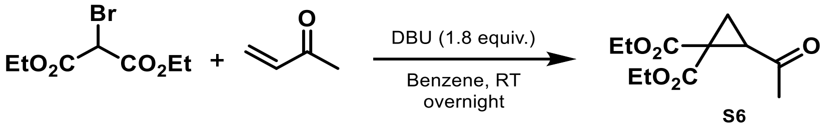

Following a literature procedure,[10] to an oven-dried 150.0 mL Schlenk tube with a Teflon-coated magnetic stir bar, 3-buten-2-one (1.0 equiv., 60 mmol, 4.9 mL), DBU (1.8 equiv., 108 mmol, 16.1 mL) and benzene (25 mL) were added. Dimethyl bromomalonate (1.5 equiv., 90.0 mmol, 21.52 g) was added dropwise over 30 min at 0 °C to the reaction mixture and stirred at room temperature overnight. The reaction mixture was filtered through a Celite® pad. After evaporation of the filtrate under reduced pressure, the crude product was purified by flash column chromatography (SiO_2; 100:0 to 85:15 pentane/EtOAc) to afford the diethyl 2-acetylcyclopropane-1,1-dicarboxylate **S6** (5.25 g, 38%) as a colourless oil.

TLC: R_f = 0.2 (90:10 pentane/EtOAc).

NMR Spectroscopy:

^{1}H NMR (400 MHz, $CDCl_3$): δ_H 4.24 – 4.11 (m, 4H), 2.84 (dd, *J* = 8.4, 6.8 Hz, 1H), 2.35 (s, 3H), 1.97 (dd, *J* = 6.8, 4.3 Hz, 1H), 1.60 (dd, *J* = 8.4, 4.3 Hz, 1H), 1.30 – 1.21 (m, 6H) ppm.

^{13}C NMR (101 MHz, $CDCl_3$): δ_C 203.2, 169.1, 166.0, 62.4, 61.9, 39.2, 34.2, 31.7, 20.6, 14.1, 14.0 ppm.

HRMS (ESI$^+$): m/z calc'd for $C_{11}H_{16}O_5Na$ $[M+Na]^+$: 251.08899, found: 251.08868.

diethyl 2-(prop-1-en-2-yl)cyclopropane-1,1-dicarboxylate (2j)

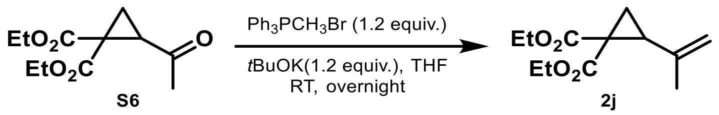

Following a literature procedure,[10] to an oven-dried 50.0 mL Schlenk tube with a Teflon-coated magnetic stir bar, Ph_3PCH_3Br (1.2 equiv., 5.26 mmol, 1.88 g) and *t*BuOK (1.2 equiv., 5.26 mmol, 590 mg) were added. THF (12 mL) was poured into the reaction mixture. After 1.5 h, **S6** (1.0 equiv., 4.38 mmol, 1.00 g) was added to the resultant yellow suspension. The mixture was warmed to 45 °C and stirred for 60 h. The reaction mixture was quenched with saturated aqueous NH_4Cl. The phases were separated and the aqueous layer was extracted with *t*BuOMe. The combined organic layer was dried over $MgSO_4$ and the solvent was evaporated under reduced pressure. The crude product was purified by flash column chromatography (SiO_2; 100:0 to 92:08 pentane/EtOAc) to afford diethyl 2-(prop-1-en-2-yl)cyclopropane-1,1-dicarboxylate **2j** (595 mg, 60%) as a colourless oil.

TLC: R_f = 0.6 (90:10 pentane/EtOAc).

^{1}H NMR (400 MHz, $CDCl_3$): δ_H 4.87 – 4.82 (m, 1H), 4.71 – 4.65 (m, 1H), 4.24 – 4.07 (m, 4H), 2.47 (t, *J* = 8.1 Hz, 1H), 1.86 – 1.78 (m, 4H), 1.43 (dd, *J* = 8.9, 5.0 Hz, 1H), 1.30 – 1.14 (m, 6H) ppm.

^{13}C NMR (101 MHz, $CDCl_3$): δ_C 170.3, 167.1, 139.2, 112.7, 61.7, 61.4, 36.5, 33.6, 23.0, 18.2, 14.2 ppm.

HRMS (ESI$^+$): m/z calc'd for $C_{12}H_{18}O_4Na$ $[M+Na]^+$: 249.10973, found: 249.10917.

General Procedure D: Dearomative Cycloaddition/Rearrangement Cascade of Bicyclic Azaarenes (5)

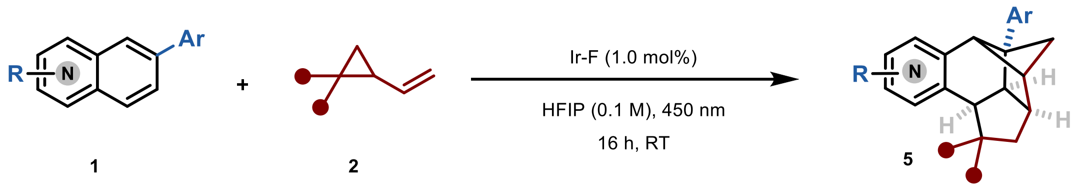

To an oven-dried 10.0 mL Schlenk tube equipped with a Teflon-coated magnetic stir bar was added azaarene **1** (1.0 equiv.) and [Ir(dF(CF_3)ppy)$_2$(dtbbpy)][PF_6] (**Ir-F**) (1.0 mol%). The tube was evacuated and backfilled with argon three times. Under an argon atmosphere, HFIP (0.1 M) and vinyl cyclopropane **2** (5.0 equiv.) were added consecutively, and the tube was sealed tightly. The reaction mixture was irradiated with 30 W blue LEDs (λ_{max} = 450 nm) for 16 hours. The solvent was removed under reduced pressure. The ^{1}H NMR yield was determined using dibromomethane (CH_2Br_2) as an internal standard. The crude product was purified by column chromatography on silica gel to get the desired product.

Note: Reaction time might vary according to different bicyclic azaarenes. All the products were obtained as a single diastereomer except **5ao** (unsymmetrical VCP was used in this case). The structure of **3a**, **3b**, **3f**, **4a**, **5h**, **5ad**, **5ag**, and **5ao** were confirmed according to X-ray crystallography. Using these crystal data as reference, the stereochemistry of remaining structures was assigned by NMR analysis.

dimethyl-3-methyl-10-phenyl-7,8,8a,9,10,10a-hexahydro-5,8,10-(epimethanetriyl)cyclobuta[3,4]cycloocta[1,2-*b*]pyridine-6,6(5*H*)-dicarboxylate (5b)

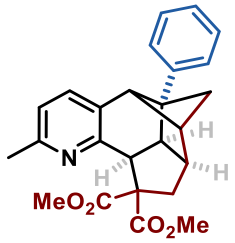

According to **General Procedure D**, the following compound was synthesized using **1b** (1.0 equiv., 0.2 mmol, 43.9 mg), **2a** (5.0 equiv., 1.0 mmol, 184.2 mg). Reaction mixture was irradiated with blue LEDs for 16 hours. Purified by flash column chromatography (SiO_2; 100:0 to 85:15 pentane/EtOAc) to afford **5b** (37.9 mg, 47%) as a white solid. **m.p.** 147-149 °C.

TLC: R_f = 0.4 (80:20 pentane/EtOAc).

NMR Spectroscopy:

^{1}H NMR (400 MHz, $CDCl_3$): δ_H 7.28 (d, *J* = 1.4 Hz, 1H), 7.18 – 7.02 (m, 5H), 6.86 (d, *J* = 7.6 Hz, 1H), 4.25 (d, *J* = 5.7 Hz, 1H), 3.81 (s, 3H), 3.73 (s, 3H), 3.53 (ddd, *J* = 8.6, 5.8, 2.0 Hz, 1H), 3.04 – 2.99 (m, 1H), 2.93 – 2.84 (m, 1H), 2.67 (q, *J* = 8.6 Hz, 1H), 2.42 (s, 3H), 2.24 (dd, *J* = 13.7, 8.4 Hz, 1H), 2.11 (dd, *J* = 7.1, 3.0 Hz, 1H), 1.83 (dd, *J* = 14.2, 8.3 Hz, 1H), 1.71 (d, *J* = 7.0 Hz, 1H) ppm.

^{13}C NMR (101 MHz, $CDCl_3$): δ_C 172.0, 169.5, 156.0, 155.3, 140.8, 136.2, 129.9, 128.2, 127.0, 126.5, 121.4, 70.0, 53.3, 52.9, 52.3, 51.3, 49.8, 49.5, 48.8, 47.5, 44.4, 31.7, 24.5 ppm.

HRMS (ESI^+): m/z calc'd for $C_{25}H_{25}NO_4Na$ $[M+Na]^+$: 426.16758, found: 426.16778.

<u>**Note**</u>: 2D-NMR data is provided (COSY, HSQC, HMBC) for confirmation of the structure. The multiplicity of proton at 2.67 ppm in ^{1}H NMR of **5b** product was assigned as quartet by analyzing the COSY spectra. All three correlations of this quartet can be observed with a similar coupling constant (*J* = 8.6 Hz).

dimethyl-3-methyl-10-phenyl-7,8,8a,9,10,10a-hexahydro-5,8,10-(epimethanetriyl)cyclobuta[3,4]cycloocta[1,2-*b*]pyridine-6,6(5*H*)-dicarboxylate (5g)

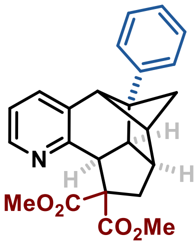

According to **General Procedure D**, the following compound was synthesized using **1g** (1.0 equiv., 0.2 mmol, 41.1 mg), **2a** (5.0 equiv., 1.0 mmol, 184.2 mg). The reaction mixture was irradiated with blue LEDs for 16 hours. Purified by flash column chromatography (SiO_2; 100:0 to 65:35 pentane/EtOAc) to afford **5g** (31.9 mg, 41%) as a white solid.

TLC: R_f = 0.4 (70:30 pentane/EtOAc).

NMR Spectroscopy:

^{1}H NMR (400 MHz, $CDCl_3$): δ_H 8.28 (dd, *J* = 4.8, 1.9 Hz, 1H), 7.37 (dd, *J* = 7.6, 1.8 Hz, 1H), 7.16 – 6.92 (m, 6H), 4.27 (d, *J* = 5.7 Hz, 1H), 3.78 (s, 3H), 3.68 (s, 3H), 3.59 – 3.51 (m, 1H), 3.05 – 2.99 (m, 1H), 2.95 – 2.88 (m, 1H), 2.68 (q, *J* = 8.7 Hz, 1H), 2.25 (ddd, *J* = 14.3, 8.9, 1.6 Hz, 1H), 2.11 (dd, *J* = 6.9, 2.9 Hz, 1H), 1.82 (dd, *J* = 14.1, 8.4 Hz, 1H), 1.71 (d, *J* = 6.9 Hz, 1H) ppm.

^{13}C NMR (101 MHz, $CDCl_3$): δ_C 171.9, 169.4, 156.2, 147.6, 140.7, 135.9, 133.1, 128.3, 126.9, 126.6, 121.9, 69.9, 53.3, 52.9, 52.5, 51.5, 49.9, 49.5, 48.9, 47.5, 44.4, 31.7 ppm.

HRMS (ESI^+): m/z calc'd for $C_{24}H_{23}NO_4Na$ $[M+Na]^+$: 412.15193, found: 412.15177.

dimethyl-3-methyl-10-(4-(trifluoromethyl)phenyl)-7,8,8a,9,10,10a-hexahydro-5,8,10(epimethanetriyl)cyclobuta[3,4]cycloocta[1,2-*b*]pyridine-6,6(5*H*)-dicarboxylate (5h)

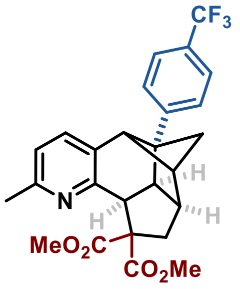

According to **General Procedure D**, the following compound was synthesized using **1h** (1.0 equiv., 0.2 mmol, 57.4 mg) and **2a** (5.0 equiv., 1.0 mmol, 184.2 mg). The reaction mixture was irradiated with blue LEDs for 16 hours. Purified by flash column chromatography (SiO_2; 100:0 to 80:20 pentane/EtOAc) to afford **5h** (64.1 mg, 68%) as a white solid. **m.p.** 139-144 °C.

TLC: R_f = 0.5 (80:20 pentane/EtOAc).

NMR Spectroscopy:

^{1}H NMR (400 MHz, $CDCl_3$): δ_H 7.41 – 7.37 (m, 2H), 7.29 (d, *J* = 7.7 Hz, 1H), 7.19 – 7.12 (m, 2H), 6.88 (d, *J* = 7.7 Hz, 1H), 4.21 (d, *J* = 5.8 Hz, 1H), 3.80 (s, 3H), 3.72 (s, 3H), 3.55 (ddd, *J* = 8.3, 5.8, 2.0 Hz, 1H), 3.04 (m, 1H), 2.94 (m, 1H), 2.70 (m, 1H), 2.42 (s, 3H), 2.24 (ddd, *J* = 14.0, 8.9, 1.4 Hz, 1H), 2.11 (dd, *J* = 6.9, 3.0 Hz, 1H), 1.84 (dd, *J* = 14.0, 8.4 Hz, 1H), 1.74 (d, *J* = 6.9 Hz, 1H) ppm.

^{13}C{^{19}F} NMR (151 MHz, $CDCl_3$): δ_C 171.9, 169.3, 156.4, 155.1, 144.9, 136.2, 129.5, 128.7, 127.3, 125.2, 124.3, 121.5, 69.9, 53.0, 52.9, 52.4, 51.2, 49.6, 49.6, 48.9, 47.5, 44.4, 31.6, 24.4 ppm.

^{19}F NMR (376 MHz, CDCl3): δ_F -62.15 ppm.

HRMS (ESI$^+$): m/z calc'd for $C_{26}H_{24}F_3NO_4Na$ $[M+Na]^+$: 494.15496, found: 494.15441.

X-ray: **see Supplementary Figure 23**.

dimethyl-10-(4-(trifluoromethyl)phenyl)-7,8,8a,9,10,1t0a-hexahydro-5,8,10-(epimethanetriyl)cyclobuta[3,4]cycloocta[1,2-*b*]pyridine-6,6(5*H*)-dicarboxylate (5i)

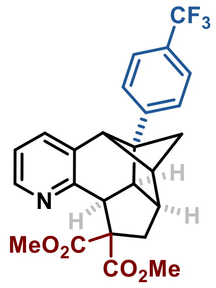

According to **General Procedure D**, the following compound was synthesized using **1i** (1.0 equiv., 0.2 mmol,

54.6 mg), **2a** (5.0 equiv., 1.0 mmol, 184.2 mg). The reaction mixture was irradiated with blue LEDs for 16 hours. Purified by flash column chromatography (SiO_2; 100:0 to 75:25 pentane/EtOAc) to afford **5i** (45.7 mg, 50%) as a white solid. **m.p.** 152-153 °C.

TLC: R_f = 0.52 (70:30 pentane/EtOAc).

NMR Spectroscopy:

^{1}H NMR (400 MHz, $CDCl_3$): δ_H 8.32 (dd, *J* = 4.9, 1.7 Hz, 1H), 7.45 – 7.36 (m, 3H), 7.14 (d, *J* = 8.0 Hz, 2H), 7.04 (dd, *J* = 7.6, 4.8 Hz, 1H), 4.25 (d, *J* = 5.8 Hz, 1H), 3.81 (s, 3H), 3.70 (s, 3H), 3.57 (ddd, *J* = 8.3, 5.8, 2.1 Hz, 1H), 3.09 – 3.04 (m, 1H), 3.03 – 2.94 (m, 1H), 2.73 (q, *J* = 8.6 Hz, 1H), 2.26 (ddd, *J* = 14.1, 8.9, 1.4 Hz, 1H), 2.13 (dd, *J* = 6.9, 3.0 Hz, 1H), 1.84 (dd, *J* = 14.1, 8.4 Hz, 1H), 1.77 (d, *J* = 6.9 Hz, 1H) ppm.

^{13}C{^{19}F} NMR (126 MHz, $CDCl_3$): δ_C 171.8, 169.3, 155.9, 147.8, 144.7, 136.1, 132.8, 128.9, 128.0, 127.2, 126.2, 125.3, 124.2, 122.1, 69.9, 53.0, 52.6, 51.5, 49.6, 49.1, 47.6, 44.5, 31.7 ppm.

^{19}F NMR (376 MHz, CDCl3): δ_F -62.27 ppm.

HRMS (ESI$^+$): m/z calc'd for $C_{25}H_{22}F_3NO_4Na$ $[M+Na]^+$: 480.13931.16285, found: 480.13908.

dimethyl-10-(4-chlorophenyl)-3-methyl-7,8,8a,9,10,10a-hexahydro-5,8,10-(epimethanetriyl)cyclobuta[3,4]cycloocta[1,2-*b*]pyridine-6,6(5*H*)-dicarboxylate (5j)

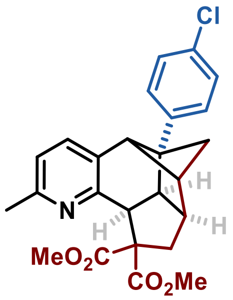

According to **General Procedure D**, the following compound was synthesized using **1j** (1.0 equiv., 0.2 mmol, 50.7 mg), **2a** (5.0 equiv., 1.0 mmol, 184.2 mg). Reaction mixture was irradiated with blue LEDs for 16 hours. Purified by flash column chromatography (SiO_2; 100:0 to 75:25 pentane/EtOAc) to afford **5j** (33.3 mg, 38%) as a white solid.

TLC: R_f = 0.3 (80:20 pentane/EtOAc).

NMR Spectroscopy:

^{1}H NMR (400 MHz, $CDCl_3$): δ_H 7.29 – 7.22 (m, 1H), 7.10 (d, *J* = 8.5 Hz, 2H), 7.00 (d, *J* = 8.6 Hz, 2H), 6.86 (d, *J* = 7.7 Hz, 1H), 4.22 (d, *J* = 5.8 Hz, 1H), 3.80 (s, 3H), 3.72 (s, 3H), 3.49 (ddd, *J* = 9.4, 4.5, 1.6 Hz, 1H), 3.02 – 2.94 (m, 1H), 2.94 – 2.86 (m, 1H), 2.67 (q, *J* = 8.7 Hz, 1H), 2.42 (s, 3H), 2.23 (ddd, *J* = 14.0, 8.8, 1.4 Hz, 1H), 2.07 (dd, *J* = 6.9, 2.9 Hz, 1H), 1.82 (dd, *J* = 14.0, 8.4 Hz, 1H), 1.69 (d, *J* = 6.9 Hz, 1H) ppm.

^{13}C NMR (101 MHz, $CDCl_3$): δ_C 171.9, 169.4, 156.3, 155.1, 139.3, 136.2, 132.3, 129.6, 128.4, 121.5, 70.0,

53.0, 52.7, 52.4, 51.4, 49.7, 49.4, 48.8, 47.5, 44.4, 31.7, 24.5 ppm.

HRMS (ESI$^+$): m/z calc'd for $C_{25}H_{24}NO_4ClNa$ $[M+Na]^+$: 460.12861, found: 460.12976.

dimethyl-10-(4-fluorophenyl)-3-methyl-7,8,8a,9,10,10a-hexahydro-5,8,10-(epimethanetriyl)cyclobuta[3,4]cycloocta[1,2-*b*]pyridine-6,6(5*H*)-dicarboxylate (5k)

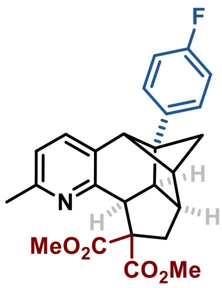

According to **General Procedure D**, the following compound was synthesized using **1k** (1.0 equiv., 0.2 mmol, 47.4 mg), **2a** (5.0 equiv., 1.0 mmol, 184.2 mg). Reaction mixture was irradiated with blue LEDs for 16 hours. Purified by flash column chromatography (SiO_2; 100:0 to 80:20 pentane/EtOAc) to afford **5k** (32.9 mg, 39%) as a white solid. **m.p.** 144-148 °C.

TLC: R_f = 0.4 (80:20 pentane/EtOAc).

NMR Spectroscopy:

^{1}H NMR (400 MHz, $CDCl_3$): δ_H 7.22 – 7.19 (m, 1H), 6.99 – 6.92 (m, 2H), 6.81 – 6.71 (m, 3H), 4.15 (d, *J* = 5.7 Hz, 1H), 3.73 (s, 3H), 3.65 (s, 3H), 3.46 – 3.39 (m, 1H), 2.93 – 2.89 (m, 1H), 2.86 – 2.81 (m, 1H), 2.60 (q, *J* = 8.7 Hz, 1H), 2.35 (s, 3H), 2.16 (dd, *J* = 13.9, 8.8 Hz, 1H), 1.99 (dd, *J* = 6.9, 3.0 Hz, 1H), 1.75 (dd, *J* = 14.0, 8.4 Hz, 1H), 1.62 (d, *J* = 6.8 Hz, 1H) ppm.

^{13}C{^{19}F} NMR (126 MHz, $CDCl_3$): δ_C 171.9, 169.4, 161.6, 156.2, 155.2, 136.5, 136.2, 129.7, 128.5, 121.5, 115.1, 69.9, 53.0, 52.6, 52.4, 51.4, 49.7, 49.5, 48.8, 47.7, 44.4, 31.7, 24.4 ppm.

^{19}F NMR (376 MHz, CDCl3): δ_F -116.37 ppm.

HRMS (ESI$^+$): m/z calc'd for $C_{25}H_{24}FNO_4Na$ $[M+Na]^+$: 444.15816, found: 444.15829.

dimethyl-10-(4-(methoxycarbonyl)phenyl)-3-methyl-7,8,8a,9,10,10a-hexahydro-5,8,10-(epimethanetriyl)cyclobuta[3,4]cycloocta[1,2-*b*]pyridine-6,6(5*H*)-dicarboxylate (5l)

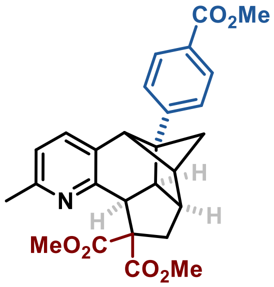

According to **General Procedure D**, the following compound was synthesized using **1l** (1.0 equiv., 0.2 mmol, 55.5 mg), **2a** (5.0 equiv., 1.0 mmol, 184.2 mg). The reaction mixture was irradiated with blue LEDs for 24 hours. Purified by flash column chromatography (SiO_2; 100:0 to 80:20 pentane/EtOAc) to afford **5l** (42.5 mg, 46%) as a white solid.

TLC: R_f = 0.7 (60:40 pentane/EtOAc).

NMR Spectroscopy:

^{1}H NMR (400 MHz, $CDCl_3$): δ_H 7.81 (d, *J* = 8.1 Hz, 2H), 7.28 (m, 1H), 7.12 (d, *J* = 8.4 Hz, 2H), 6.87 (d, *J* = 7.8 Hz, 1H), 4.23 (br. s, 1H), 3.84 (s, 3H), 3.81 (s, 3H), 3.72 (s, 3H), 3.55 (ddd, *J* = 8.3, 5.8, 2.0 Hz, 1H), 3.04 (m, 1H), 2.94 (m, 1H), 2.70 (q, *J* = 8.7 Hz, 1H), 2.42 (s, 3H), 2.31 – 2.20 (m, 1H), 2.12 (dd, *J* = 6.9, 3.0 Hz, 1H), 1.83 (dd, *J* = 14.0, 8.4 Hz, 1H), 1.74 (d, *J* = 6.9 Hz, 1H) ppm.

^{13}C NMR (101 MHz, $CDCl_3$): δ_C 171.9, 169.4, 167.0, 156.3, 155.1, 146.2, 136.2, 130.0, 129.6, 128.4, 127.0, 121.5, 69.9, 53.3, 53.0, 52.4, 52.1, 51.3, 49.7, 49.5, 49.0, 47.5, 44.4, 31.7, 24.4 ppm.

HRMS (ESI$^+$): m/z calc'd for $C_{27}H_{27}NO_6Na$ $[M+Na]^+$: 484.17306, found: 484.17331.

dimethyl-10-(4-cyanophenyl)-3-methyl-7,8,8a,9,10,10a-hexahydro-5,8,10-(epimethanetriyl)cyclobuta[3,4]cycloocta[1,2-*b*]pyridine-6,6(5*H*)-dicarboxylate (5m)

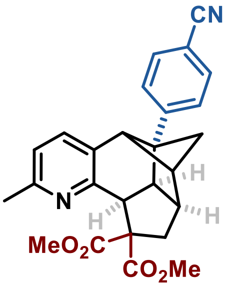

According to **General Procedure D**, the following compound was synthesized using **1m** (1.0 equiv., 0.2 mmol, 48.9 mg), **2a** (5.0 equiv., 1.0 mmol, 184.2 mg). The reaction mixture was irradiated with blue LEDs for 48 hours. Purified by flash column chromatography (SiO_2; 100:0 to 80:20 pentane/EtOAc) to afford **5m** (44.6 mg, 52%) as a white solid.

TLC: R_f = 0.7 (60:40 pentane/EtOAc).

NMR Spectroscopy:

^{1}H NMR (400 MHz, $CDCl_3$): δ_H 7.42 (d, *J* = 8.4 Hz, 2H), 7.31 (d, *J* = 10.8 Hz, 1H), 7.13 (d, *J* = 8.4 Hz, 2H), 6.90 (d, *J* = 7.8 Hz, 1H), 4.20 (br. s, 1H), 3.80 (s, 3H), 3.72 (s, 3H), 3.53 (ddd, *J* = 8.3, 5.8, 2.1 Hz, 1H), 3.07 – 3.01 (m, 1H), 3.00 – 2.90 (m, 1H), 2.71 (q, *J* = 8.7 Hz, 1H), 2.44 (s, 3H), 2.25 (dd, *J* = 14.0, 9.0 Hz, 1H), 2.10 (dd, *J* = 7.0, 3.0 Hz, 1H), 1.82 (dd, *J* = 14.1, 8.4 Hz, 1H), 1.74 (d, *J* = 6.9 Hz, 1H) ppm.

^{13}C NMR (101 MHz, $CDCl_3$): δ_C 171.8, 169.2, 156.5, 154.9, 146.4, 136.2, 132.5, 132.1, 129.4, 127.7, 121.6,

110.3, 69.9, 53.0, 53.0, 52.4, 51.2, 49.6, 49.5, 49.0, 47.4, 44.4, 31.6, 24.4 ppm.

HRMS (ESI^+): m/z calc'd for $C_{26}H_{24}N_2O_4Na$ $[M+Na]^+$: 451.16283, found: 451.16300.

dimethyl-3-methyl-10-(*p*-tolyl)-7,8,8a,9,10,10a-hexahydro-5,8,10-(epimethanetriyl)cyclobuta[3,4]cycloocta[1,2-*b*]pyridine-6,6(5*H*)-dicarboxylate (5n)

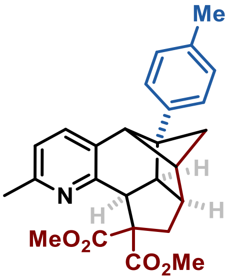

According to **General Procedure D**, the following compound was synthesized using **1n** (1.0 equiv., 0.2 mmol, 46.7 mg), **2a** (5.0 equiv., 1.0 mmol, 184.2 mg). Reaction mixture was irradiated with blue LEDs for 48 hours. Purified by flash column chromatography (SiO_2; 100:0 to 92:08 pentane/EtOAc) to afford **5n** (29.2 mg, 35%) as a white solid.

TLC: R_f = 0.6 (90:10 pentane/EtOAc).

NMR Spectroscopy:

^{1}H NMR (400 MHz, $CDCl_3$): δ_H 7.28 – 7.22 (m, 1H), 7.01 – 6.90 (m, 4H), 6.84 (d, *J* = 7.7 Hz, 1H), 4.26 (d, *J* = 5.8 Hz, 1H), 3.80 (s, 3H), 3.72 (s, 3H), 3.50 (ddd, *J* = 8.3, 5.8, 2.0 Hz, 1H), 3.00 – 2.94 (m, 1H), 2.92 – 2.84 (m, 1H), 2.66 (q, *J* = 8.7 Hz, 1H), 2.41 (s, 3H), 2.27 – 2.19 (m, 4H), 2.09 (dd, *J* = 6.9, 3.0 Hz, 1H), 1.82 (dd, *J* = 14.1, 8.5 Hz, 1H), 1.67 (d, *J* = 6.9 Hz, 1H) ppm.

^{13}C NMR (101 MHz, $CDCl_3$): δ_C 172.0, 169.5, 156.0, 155.4, 137.8, 136.2, 136.1, 129.9, 128.9, 126.9, 121.3, 70.0, 53.1, 52.9, 52.3, 51.5, 49.9, 49.3, 48.7, 47.5, 44.4, 31.7, 24.5, 21.1 ppm.

HRMS (ESI^+): m/z calc'd for $C_{26}H_{27}NO_4Na$ $[M+Na]^+$: 440.18323, found: 440.18461.

dimethyl-3-methyl-10-(4-(4,4,5,5-tetramethyl-1,3,2-dioxaborolan-2-yl)phenyl)-7,8,8a,9,10,10a-hexahydro-5,8,10-(epimethanetriyl)cyclobuta[3,4]cycloocta[1,2-*b*]pyridine-6,6(5*H*)-dicarboxylate (5o)

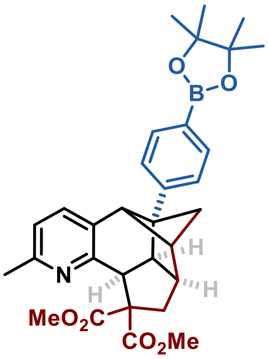

According to **General Procedure D**, the following compound was synthesized using **1o** (1.0 equiv., 0.2 mmol, 69.0 mg), **2a** (5.0 equiv., 1.0 mmol, 184.2 mg). Reaction mixture was irradiated with blue LEDs for 48 hours. Purified by flash column chromatography (SiO_2; 100:0 to 85:15 pentane/EtOAc) to afford **5o** (51.9 mg, 49%) as a white fluffy solid. **m.p.** 67-69 °C.

TLC: R_f = 0.2 (90:10 pentane/EtOAc).

NMR Spectroscopy:

^{1}H NMR (400 MHz, $CDCl_3$): δ_H 7.59 (d, *J* = 7.9 Hz, 2H), 7.24 (d, *J* = 8.8 Hz, 1H), 7.10 (d, *J* = 8.0 Hz, 2H), 6.83 (d, *J* = 7.7 Hz, 1H), 4.26 (d, *J* = 5.7 Hz, 1H), 3.80 (s, 3H), 3.72 (s, 3H), 3.54 (ddd, *J* = 8.3, 5.8, 2.0 Hz, 1H), 3.09 – 2.95 (m, 1H), 2.95 – 2.84 (m, 1H), 2.67 (q, *J* = 8.7 Hz, 1H), 2.40 (s, 3H), 2.29 – 2.18 (m, 1H), 2.12 (dd, *J* = 6.9, 3.0 Hz, 1H), 1.83 (dd, *J* = 14.0, 8.4 Hz, 1H), 1.69 (d, *J* = 6.9 Hz, 1H), 1.28 (s, 12H) ppm;

^{13}C NMR (101 MHz, $CDCl_3$): δ_C 172.0, 169.5, 156.1, 155.2, 144.0, 136.2, 134.8, 129.8, 126.5, 121.3, 83.7, 70.0, 53.5, 53.0, 52.4, 51.5, 49.9, 49.2, 48.8, 47.5, 44.4, 31.7, 24.9, 24.4 ppm.

^{11}B NMR (128 MHz, $CDCl_3$): δ 30.06 ppm.

HRMS (ESI^+): m/z calc'd for $C_{31}H_{36}NO_6BNa$ $[M+Na]^+$: 552.25279, found: 552.25480.

dimethyl-10-(3-fluorophenyl)-3-methyl-7,8,8a,9,10,10a-hexahydro-5,8,10-(epimethanetriyl)cyclobuta[3,4]cycloocta[1,2-*b*]pyridine-6,6(5*H*)-dicarboxylate (5p)

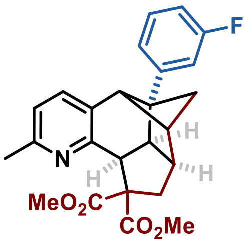

According to **General Procedure D**, the following compound was synthesized using **1p** (1.0 equiv., 0.2 mmol, 47.4 mg), **2a** (5.0 equiv., 1.0 mmol, 184.2 mg). Reaction mixture was irradiated with blue LEDs for 16 hours. Purified by flash column chromatography (SiO_2; 100:0 to 70:30 pentane/EtOAc) to afford **5p** (42.1 mg, 50%) as a white solid.

TLC: R_f = 0.3 (80:20 pentane/EtOAc).

NMR Spectroscopy:

^{1}H NMR (400 MHz, $CDCl_3$): δ_H 7.30 – 7.26 (m, 1H), 7.13 – 7.05 (m, 1H), 6.90 – 6.81 (m, 2H), 6.81 – 6.71 (m, 2H), 4.22 (d, *J* = 5.8 Hz, 1H), 3.80 (s, 3H), 3.72 (s, 3H), 3.51 (ddd, *J* = 8.3, 5.8, 2.0 Hz, 1H), 3.06 – 2.96 (m, 1H), 2.93 – 2.87 (m, 1H), 2.68 (q, *J* = 8.7 Hz, 1H), 2.42 (s, 3H), 2.24 (dd, *J* = 14.0, 8.8 Hz, 1H), 2.07 (dd, *J* = 6.9, 3.0 Hz, 1H), 1.82 (dd, *J* = 14.0, 8.4 Hz, 1H), 1.71 (d, *J* = 6.8 Hz, 1H) ppm.

^{13}C{^{19}F} NMR (126 MHz, $CDCl_3$): δ_C 171.9, 169.4, 162.8, 156.3, 155.2, 143.5, 136.2, 129.7, 129.6, 122.6,

121.5, 114.0, 113.5, 69.9, 53.0, 52.9, 52.4, 51.2, 49.6, 49.5, 48.8, 47.7, 44.4, 31.6, 24.5 ppm.

^{19}F NMR (376 MHz, $CDCl_3$): δ_F -113.42 ppm.

HRMS (ESI$^+$): m/z calc'd for $C_{25}H_{24}NO_4FNa$ [M+Na]$^+$: 444.15816, found: 444.15701.

dimethyl-10-(2,4-difluorophenyl)-3-methyl-7,8,8a,9,10,10a-hexahydro-5,8,10-(epimethanetriyl) cyclobuta[3,4]cycloocta[1,2-*b*]pyridine-6,6(5*H*)-dicarboxylate (5q)

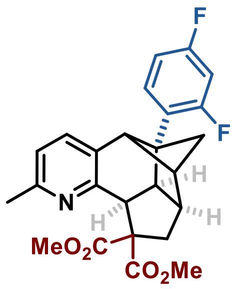

According to **General Procedure D**, the following compound was synthesized using **1q** (1.0 equiv., 0.2 mmol, 51.1 mg), **2a** (5.0 equiv., 1.0 mmol, 184.2 mg). The reaction mixture was irradiated with blue LEDs for 16 hours. Purified by flash column chromatography (SiO_2; 100:0 to 85:15 pentane/EtOAc) to afford **5q** (47.5 mg, 54%) as a white solid.

TLC: R_f = 0.5 (80:20 pentane/EtOAc).

NMR Spectroscopy:

^{1}H NMR (400 MHz, $CDCl_3$): δ_H 7.27 (d, *J* = 7.7 Hz, 1H), 6.82 (d, *J* = 7.7 Hz, 1H), 6.72 – 6.64 (m, 1H), 6.59 (ddd, *J* = 11.4, 8.9, 2.6 Hz, 1H), 6.52 – 6.44 (m, 1H), 4.03 (d, *J* = 5.1 Hz, 1H), 3.73 (s, 3H), 3.63 (s, 3H), 3.61 – 3.56 (m, 1H), 3.20 – 3.08 (m, 1H), 2.89 – 2.81 (m, 1H), 2.57 (q, *J* = 8.6 Hz, 1H), 2.34 (s, 3H), 2.16 (dd, *J* = 14.0, 8.8 Hz, 1H), 2.09 (dd, *J* = 7.0, 3.0 Hz, 1H), 1.77 – 1.67 (m, 2H) ppm.

^{13}C{^{19}F} NMR (126 MHz, $CDCl_3$): δ_C 171.8, 169.3, 161.9, 161.7, 156.2, 155.0, 136.0, 129.8, 129.7, 123.5, 121.5, 110.7, 104.1, 69.9, 52.9, 52.3, 49.6, 49.5, 49.5, 49.3, 49.0, 47.5, 43.7, 31.6, 24.4 ppm.

^{19}F NMR (376 MHz, CDCl3): δ_F -110.05 (d, *J* = 7.4 Hz), -112.74 (d, *J* = 7.3 Hz) ppm.

HRMS (ESI$^+$): m/z calc'd for $C_{25}H_{23}F_2NO_4Na$ [M+Na]$^+$: 462.14874, found: 462.14830.

dimethyl-10-(3,5-dichlorophenyl)-3-methyl-7,8,8a,9,10,10a-hexahydro-5,8,10-(epimethanetriyl)cyclobuta[3,4]cycloocta[1,2-*b*]pyridine-6,6(5*H*)-dicarboxylate (5r)

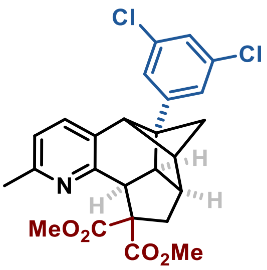

According to **General Procedure D**, the following compound was synthesized using **1r** (1.0 equiv., 0.2 mmol, 57.6 mg), **2a** (5.0 equiv., 1.0 mmol, 184.2 mg). The reaction mixture was irradiated with blue LEDs for 48 hours. Purified by flash column chromatography (SiO_2; 100:0 to 85:15 pentane/EtOAc) to afford **5r** (19.8 mg, 21%) as a white solid. **m.p.** 175-178 °C.

TLC: R_f = 0.4 (80:20 pentane/EtOAc).

NMR Spectroscopy:

^{1}H NMR (400 MHz, $CDCl_3$): δ_H 7.22 (d, *J* = 7.7 Hz, 1H), 7.03 (t, *J* = 1.9 Hz, 1H), 6.86 (d, *J* = 2.0 Hz, 2H), 6.82 (d, *J* = 7.8 Hz, 1H), 4.13 (d, *J* = 5.7 Hz, 1H), 3.74 (s, 3H), 3.65 (s, 3H), 3.44 (ddd, *J* = 8.4, 5.8, 2.2 Hz, 1H), 2.96 – 2.93 (m, 1H), 2.87 – 2.83 (m, 1H), 2.61 (q, *J* = 8.7 Hz, 1H), 2.36 (s, 3H), 2.16 (dd, *J* = 14.0, 8.9 Hz, 1H), 1.97 (dd, *J* = 6.6, 3.4 Hz, 1H), 1.75 (dd, *J* = 14.0, 8.4 Hz, 1H), 1.64 (d, *J* = 6.9 Hz, 1H) ppm.

^{13}C NMR (101 MHz, $CDCl_3$): δ_C 171.9, 169.3, 156.6, 154.9, 144.4, 136.2, 134.9, 129.2, 126.9, 125.7, 121.6, 70.0, 53.0, 52.6, 52.4, 51.0, 49.4, 49.2, 48.9, 48.0, 44.4, 31.6, 24.5 ppm.

HRMS (ESI$^+$): m/z calc'd for $C_{25}H_{23}Cl_2NO_4Na$ $[M+Na]^+$: 494.08963, found: 494.08838.

dimethyl-3-methyl-10-(pyridin-4-yl)-7,8,8a,9,10,10a-hexahydro-5,8,10-(epimethanetriyl)cyclobuta[3,4]cycloocta[1,2-*b*]pyridine-6,6(5*H*)-dicarboxylate (5s)

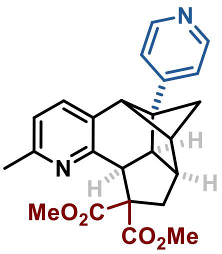

According to **General Procedure D**, the following compound was synthesized using **1s** (1.0 equiv., 0.2 mmol, 44.1 mg), **2a** (5.0 equiv., 1.0 mmol, 184.2 mg). The reaction mixture was irradiated with blue LEDs for 16 hours. Purified by flash column chromatography (SiO_2; 100:0 to 85:15 CH_2Cl_2/MeOH) to afford **5s** (34.0 mg, 42%) as a pale-yellow solid.

TLC: R_f = 0.3(80:20 CH_2Cl_2/MeOH).

NMR Spectroscopy:

^{1}H NMR (400 MHz, $CDCl_3$): δ_H 8.33 (d, *J* = 5.1 Hz, 2H), 7.26 (d, *J* = 7.7 Hz, 1H), 6.97 – 6.90 (m, 2H), 6.87

(d, J = 7.8 Hz, 1H), 4.19 (d, J = 5.8 Hz, 1H), 3.78 (s, 3H), 3.70 (s, 3H), 3.52 (ddd, J = 8.3, 5.9, 2.1 Hz, 1H), 3.03 – 2.99 (m, 1H), 2.95 – 2.91 (m, 1H), 2.68 (q, J = 8.7 Hz, 1H), 2.41 (s, 3H), 2.22 (ddd, J = 14.1, 8.8, 1.4 Hz, 1H), 2.09 (dd, J = 6.9, 3.0 Hz, 1H), 1.81 (dd, J = 14.0, 8.4 Hz, 1H), 1.71 (d, J = 6.9 Hz, 1H) ppm.

^{13}C NMR (101 MHz, $CDCl_3$): 171.8, 169.2, 156.5, 154.9, 149.8, 149.5, 136.1, 129.2, 122.2, 121.5, 69.9, 52.9, 52.3, 52.3, 51.1, 49.5, 49.4, 49.0, 46.8, 44.3, 31.5, 24.4 ppm.

HRMS (ESI$^+$): m/z calc'd for $C_{24}H_{24}N_2O_4Na$ [M+Na]$^+$: 427.16283, found: 427.16181.

dimethyl-3-methyl-10-(1-methyl-1H-pyrazol-5-yl)-7,8,8a,9,10,10a-hexahydro-5,8,10-(epimethanetriyl)cyclobuta[3,4]cycloocta[1,2-*b*]pyridine-6,6(5*H*)-dicarboxylate (5t)

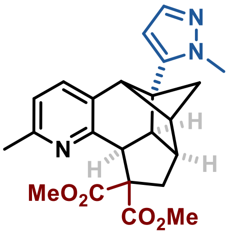

According to **General Procedure D**, the following compound was synthesized using **1t** (1.0 equiv., 0.2 mmol, 44.6 mg), **2a** (5.0 equiv., 1.0 mmol, 184.2 mg). The reaction mixture was irradiated with blue LEDs for 16 hours. Purified by flash column chromatography (SiO_2; 100:0 to 30:70 pentane/EtOAc) to afford **5t** (34.2 mg, 42%) as a white solid.

TLC: R_f = 0.2 (40:60 pentane/EtOAc).

NMR Spectroscopy:

^{1}H NMR (400 MHz, $CDCl_3$): δ_H 7.32 (d, J = 7.7 Hz, 1H), 7.11 (d, J = 1.9 Hz, 1H), 6.91 (d, J = 7.7 Hz, 1H), 5.34 (d, J = 1.9 Hz, 1H), 4.03 (d, J = 6.4 Hz, 1H), 3.84 (s, 3H), 3.79 (s, 3H), 3.70 (s, 3H), 3.59 (ddd, J = 8.4, 5.8, 2.2 Hz, 1H), 3.15 – 3.09 (m, 1H), 2.97 – 2.93 (m, 1H), 2.64 (q, J = 8.6 Hz, 1H), 2.43 (s, 3H), 2.22 (ddd, J = 14.1, 8.8, 1.4 Hz, 1H), 2.14 (dd, J = 6.9, 3.0 Hz, 1H), 1.90 – 1.78 (m, 2H) ppm.

^{13}C NMR (101 MHz, $CDCl_3$): δ_C 171.9, 169.2, 156.6, 155.0, 141.4, 137.9, 136.0, 129.0, 121.5, 105.4, 69.7, 53.0, 52.4, 50.4, 49.8, 49.3, 49.2, 46.6, 46.2, 43.7, 38.1, 31.7, 24.5 ppm.

HRMS (ESI$^+$): m/z calc'd for $C_{23}H_{25}N_3O_4Na$ [M+Na]$^+$: 430.17373, found: 430.17333.

dimethyl-3-methyl-10-(thiazol-4-yl)-7,8,8a,9,10,10a-hexahydro-5,8,10-(epimethanetriyl)cyclobuta[3,4]cycloocta[1,2-*b*]pyridine-6,6(5*H*)-dicarboxylate (5u)

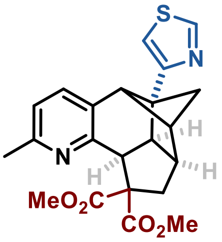

According to **General Procedure D**, the following compound was synthesized using **1u** (1.0 equiv., 0.2 mmol, 45.3 mg), **2a** (5.0 equiv., 1.0 mmol, 184.2 mg). The reaction mixture was irradiated with blue LEDs for 48 hours. Purified by flash column chromatography (SiO_2; 100:0 to 60:40 pentane/EtOAc) to afford **5u** (25.4 mg, 31%) as a white solid.

TLC: R_f = 0.4 (50:50 pentane/EtOAc).

NMR Spectroscopy:

^{1}H NMR (400 MHz, $CDCl_3$): δ_H 8.61 (d, *J* = 2.0 Hz, 1H), 7.30 (d, *J* = 7.5 Hz, 1H), 6.95 (d, *J* = 2.0 Hz, 1H), 6.86 (d, *J* = 7.7 Hz, 1H), 4.34 (br. s, 1H), 3.81 (s, 3H), 3.71 (s, 3H), 3.59 (ddd, *J* = 8.4, 5.9, 2.1 Hz, 1H), 3.25 – 3.19 (m, 1H), 2.98 – 2.90 (m, 1H), 2.67 (q, *J* = 8.8 Hz, 1H), 2.42 (s, 3H), 2.37 (dd, *J* = 6.9, 3.0 Hz, 1H), 2.25 (dd, *J* = 14.1, 8.8 Hz, 1H), 1.79 (dd, *J* = 14.0, 8.4 Hz, 1H), 1.68 (d, *J* = 6.9 Hz, 1H) ppm.

^{13}C NMR (101 MHz, $CDCl_3$): δ_C 171.9, 169.4, 156.4, 156.1, 154.8, 152.5, 136.3, 129.4, 121.4, 114.3, 70.0, 53.0, 52.4, 51.2, 50.5, 49.6, 49.3, 48.4, 47.4, 44.1, 31.6, 24.4 ppm.

HRMS (ESI^+): m/z calc'd for $C_{22}H_{22}N_2O_4SNa$ $[M+Na]^+$: 433.11925, found: 433.11961.

dimethyl-10-(2-chloropyrimidin-5-yl)-3-methyl-7,8,8a,9,10,10a-hexahydro-5,8,10-(epimethanetriyl)cyclobuta[3,4]cycloocta[1,2-*b*]pyridine-6,6(5*H*)-dicarboxylate (5v)

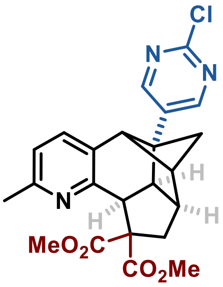

According to **General Procedure D**, the following compound was synthesized using **1v** (1.0 equiv., 0.2 mmol, 51.1 mg), **2a** (5.0 equiv., 1.0 mmol, 184.2 mg). The reaction mixture was irradiated with blue LEDs for 16 hours. Purified by flash column chromatography (SiO_2; 100:0 to 65:35 pentane/EtOAc) to afford **5v** (39.6 mg, 45%) as a white solid.

TLC: R_f = 0.6 (50:50 pentane/EtOAc).

NMR Spectroscopy:

^{1}**H NMR** (400 MHz, $CDCl_3$): δ_H 8.21 (s, 2H), 7.24 (d, *J* = 7.8 Hz, 1H), 6.84 (d, *J* = 7.7 Hz, 1H), 4.10 (d, *J* = 5.8 Hz, 1H), 3.74 (s, 3H), 3.65 (s, 3H), 3.49 (ddd, *J* = 8.3, 5.8, 2.1 Hz, 1H), 3.02 – 2.98 (m, 1H), 2.96 – 2.90 (m, 1H), 2.66 (q, *J* = 8.7 Hz, 1H), 2.36 (s, 3H), 2.19 (ddd, *J* = 14.2, 8.9, 1.4 Hz, 1H), 2.06 (dd, *J* = 7.0, 3.0 Hz, 1H), 1.78 (dd, *J* = 14.1, 8.4 Hz, 1H), 1.71 (d, *J* = 7.0 Hz, 1H) ppm.

^{13}**C NMR** (101 MHz, $CDCl_3$): δ_C 171.7, 169.0, 159.7, 158.1, 157.1, 156.8, 154.4, 136.3, 132.8, 128.6, 121.9, 69.9, 53.1, 52.5, 51.0, 49.7, 49.4, 49.2, 48.4, 46.7, 44.2, 31.5, 24.4 ppm.

HRMS (ESI$^+$): m/z calc'd for $C_{23}H_{22}ClN_3O_4Na$ $[M+Na]^+$: 462.11910, found: 462.11900.

dimethyl-3-ethyl-2-methyl-10-phenyl-7,8,8a,9,10,10a-hexahydro-5,8,10-(epimethanetriyl)cyclobuta[3,4]cycloocta[1,2-*b*]pyridine-6,6(5*H*)-dicarboxylate (5x)

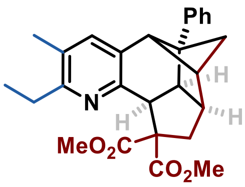

According to **General Procedure D**, the following compound was synthesized using **1x** (1.0 equiv., 0.2 mmol, 49.5 mg), **2a** (5.0 equiv., 1.0 mmol, 184.2 mg). Reaction mixture was irradiated with blue LEDs for 18 hours. Purified by flash column chromatography (SiO_2; 100:0 to 90:10 pentane/EtOAc) to afford **5x** (32.0 mg, 37%) as a white solid.

TLC: R_f = 0.5 (90:10 pentane/EtOAc).

NMR Spectroscopy:

^{1}**H NMR** (400 MHz, $CDCl_3$): δ_H 7.18 – 7.12 (m, 2H), 7.12 – 7.05 (m, 4H), 4.24 (d, *J* = 5.7 Hz, 1H), 3.80 (s, 3H), 3.73 (s, 3H), 3.50 (ddd, *J* = 8.4, 5.8, 2.0 Hz, 1H), 3.00 – 2.94 (m, 1H), 2.89 – 2.85 (m, 1H), 2.73 – 2.60 (m, 3H), 2.28 – 2.16 (m, 4H), 2.10 (dd, *J* = 6.9, 3.0 Hz, 1H), 1.82 (dd, *J* = 13.9, 8.4 Hz, 1H), 1.69 (d, *J* = 6.9 Hz, 1H), 1.17 (t, *J* = 7.6 Hz, 3H) ppm.

^{13}**C NMR** (101 MHz, $CDCl_3$): δ_C 172.2, 169.4, 159.5, 152.4, 141.1, 137.8, 130.2, 128.5, 128.2, 127.0, 126.5, 69.8, 53.2, 52.9, 52.3, 51.3, 49.5, 49.4, 48.6, 47.5, 44.3, 31.7, 29.2, 18.3, 12.8 ppm

HRMS (ESI$^+$): m/z calc'd for $C_{27}H_{29}NO_4Na$ $[M+Na]^+$: 454.19888, found: 454.19891.

dimethyl-1-chloro-10-phenyl-7,8,8a,9,10,10a-hexahydro-5,8,10-(epimethanetriyl)cyclobuta[3,4]cycloocta[1,2-*b*]pyridine-6,6(5*H*)-dicarboxylate (5y)

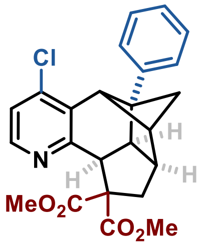

According to **General Procedure D**, the following compound was synthesized using **1y** (1.0 equiv., 0.2 mmol, 47.9 mg), **2a** (5.0 equiv., 1.0 mmol, 184.2 mg). The reaction mixture was irradiated with blue LEDs for 72 hours. Purified by flash column chromatography (SiO_2; 100:0 to 65:35 pentane/EtOAc) to afford **5y** (30.5 mg, 36%) as a white solid.

TLC: R_f = 0.3 (70:30 pentane/EtOAc).

NMR Spectroscopy:

^{1}H NMR (400 MHz, $CDCl_3$): δ_H 8.15 (d, *J* = 5.3 Hz, 1H), 7.19 – 7.14 (m, 2H), 7.14 – 7.05 (m, 4H), 4.30 (d, *J* = 5.1 Hz, 1H), 3.82 (s, 3H), 3.67 (s, 3H), 3.61 – 3.54 (m, 2H), 3.01 – 2.98 (m, 1H), 2.72 (q, *J* = 8.6 Hz, 1H), 2.28 (ddd, *J* = 14.2, 8.8, 1.4 Hz, 1H), 2.18 (dd, *J* = 7.0, 3.0 Hz, 1H), 1.83 – 1.73 (m, 2H) ppm.

^{13}C NMR (101 MHz, $CDCl_3$): δ_C 171.7, 169.2, 158.2, 147.3, 143.4, 140.0, 131.6, 128.4, 126.9, 126.8, 122.9, 70.0, 53.4, 53.1, 52.6, 50.2, 49.2, 48.6, 48.2, 47.1, 44.6, 31.8 ppm.

HRMS (ESI^+): m/z calc'd for $C_{24}H_{22}ClNO_4Na$ $[M+Na]^+$: 446.11296, found: 446.11328.

dimethyl-10-phenyl-7,8,8a,9,10,10a-hexahydro-5,8,10-(epimethanetriyl)cyclobuta[7,8]cycloocta[1,2-*b*]pyridine-6,6(5*H*)-dicarboxylate (5z)

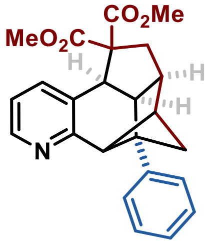

According to **General Procedure D**, the following compound was synthesized using **1z** (1.0 equiv., 0.2 mmol, 41.0 mg), **2a** (5.0 equiv., 1.0 mmol, 184.2 mg). Reaction mixture was irradiated with blue LEDs for 16 hours. Purified by flash column chromatography (SiO_2; 100:0 to 75:25 pentane/EtOAc) to afford **5z** (24.1 mg, 31%) as a yellow oil.

TLC: R_f = 0.1 (80:20 pentane/EtOAc).

^{1}H NMR (400 MHz, $CDCl_3$): δ_H 8.36 (dd, *J* = 5.1, 1.7 Hz, 1H), 7.31 – 7.26 (m, 1H), 7.19 – 7.08 (m, 3H), 7.05

– 6.97 (m, 3H), 4.14 (d, *J* = 5.9 Hz, 1H), 3.82 (s, 3H), 3.60 – 3.55 (m, 4H), 3.51 – 3.45 (m, 1H), 3.11 – 3.02 (m, 1H), 2.72 (q, *J* = 8.6 Hz, 1H), 2.30 (ddd, *J* = 14.2, 8.8, 1.4 Hz, 1H), 2.12 (dd, *J* = 7.0, 3.0 Hz, 1H), 1.86 – 1.69 (m, 2H) ppm.

^{13}C NMR (101 MHz, $CDCl_3$): δ_C 171.4, 169.1, 157.4, 146.7, 140.1, 136.1, 130.8, 128.3, 126.9, 126.6, 121.8, 70.7, 54.7, 53.2, 52.7, 52.5, 48.8, 48.7, 47.0, 44.6, 31.6 ppm.

HRMS (ESI$^+$): m/z calc'd for $C_{24}H_{23}NO_4Na$ $[M+Na]^+$: 412.15193, found: 412.15215.

dimethyl-10-(4-cyanophenyl)-7,8,8a,9,10,10a-hexahydro-5,8,10-(epimethanetriyl)cyclobuta[7,8]cycloocta[1,2-*b*]pyridine-6,6(5*H*)-dicarboxylate (5aa)

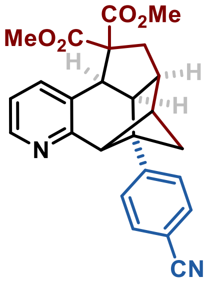

According to **General Procedure D**, the following compound was synthesized using **1aa** (1.0 equiv., 0.2 mmol, 46.0 mg), **2a** (5.0 equiv., 1.0 mmol, 184.2 mg). Reaction mixture was irradiated with blue LEDs for 16 hours. Purified by flash column chromatography (SiO_2; 100:0 to 60:40 pentane/EtOAc) to afford **5aa** (63.8 mg, 77%) as a white solid.

TLC: R_f = 0.2 (70:30 pentane/EtOAc).

NMR Spectroscopy:

^{1}H NMR (599 MHz, $CDCl_3$): δ_H 8.37 (dd, *J* = 4.9, 1.7 Hz, 1H), 7.44 – 7.38 (m, 2H), 7.25 – 7.22 (m, 1H), 7.06 – 7.04 (m, 2H), 7.02 (dd, *J* = 7.7, 4.9 Hz, 1H), 4.07 (d, *J* = 5.9 Hz, 1H), 3.81 (s, 3H), 3.58 (s, 3H), 3.56 (ddd, *J* = 8.3, 5.9, 2.0 Hz, 1H), 3.47 – 3.40 (m, 1H), 3.08 – 3.06 (m, 1H), 2.74 (q, *J* = 8.6 Hz, 1H), 2.29 (ddd, *J* = 14.2, 8.7, 1.4 Hz, 1H), 2.09 (dd, *J* = 7.0, 3.0 Hz, 1H), 1.81 – 1.71 (m, 2H) ppm.

^{13}C NMR (101 MHz, $CDCl_3$): δ_C 171.4, 168.9, 157.1, 147.9, 146.0, 135.4, 132.2, 130.0, 127.7, 121.9, 118.9, 110.4, 70.7, 60.5, 54.4, 53.3, 53.2, 52.5, 49.1, 48.8, 48.4, 46.7, 44.6, 31.4 ppm.

HRMS (ESI$^+$): m/z calc'd for $C_{25}H_{22}N_2O_4Na$ $[M+Na]^+$: 437.14718, found: 437.14586.

dimethyl-10-(4-chlorophenyl)-7,8,8a,9,10,10a-hexahydro-5,8,10-(epimethanetriyl)cyclobuta[7,8]cycloocta[1,2-*b*]pyridine-6,6(5*H*)-dicarboxylate (5ab)

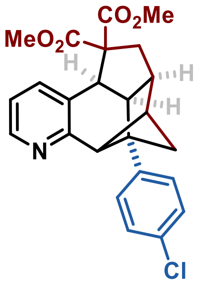

According to **General Procedure D**, the following compound was synthesized using **1ab** (1.0 equiv., 0.2 mmol, 47.9 mg), **2a** (5.0 equiv., 1.0 mmol, 184.2 mg). Reaction mixture was irradiated with blue LEDs for 16 hours. Purified by flash column chromatography (SiO_2; 100:0 to 75:25 pentane/EtOAc) to afford **5ab** (47.5 mg, 56%) as a white solid. **m.p.** 122-125 °C.

TLC: R_f = 0.3 (70:30 pentane/EtOAc).

NMR Spectroscopy:

^{1}H NMR (400 MHz, $CDCl_3$): δ_H 8.36 (dd, *J* = 4.9, 1.7 Hz, 1H), 7.29 – 7.24 (m, 1H), 7.13 – 7.08 (m, 2H), 7.03 (dd, *J* = 7.7, 4.9 Hz, 1H), 6.97 – 6.89 (m, 2H), 4.10 (d, *J* = 5.9 Hz, 1H), 3.82 (s, 3H), 3.59 (s, 3H), 3.54 (ddd, *J* = 8.2, 5.9, 2.0 Hz, 1H), 3.47 – 3.40 (m, 1H), 3.09 – 3.01 (m, 1H), 2.71 (q, *J* = 8.6 Hz, 1H), 2.35 – 2.23 (m, 1H), 2.08 (dd, *J* = 7.0, 3.0 Hz, 1H), 1.75 (dd, *J* = 14.4, 7.9 Hz, 2H) ppm.

^{13}C NMR (101 MHz, $CDCl_3$): δ_C 171.4, 169.0, 157.1, 147.0, 138.6, 136.0, 132.4, 130.5, 128.5, 128.3, 121.9, 70.7, 54.1, 53.3, 52.9, 52.5, 48.8, 48.8, 48.7, 46.8, 44.6, 31.5 ppm.

HRMS (ESI$^+$): m/z calc'd for $C_{24}H_{22}NO_4ClNa$ $[M+Na]^+$: 446.11296, found: 446.11309.

dimethyl-10-(4-(*tert*-butyl)phenyl)-2-methyl-7,8,8a,9,10,10a-hexahydro-5,8,10-(epimethanetriyl)cyclobuta[7,8]cycloocta[1,2-b]pyridine-6,6(5*H*)-dicarboxylate (5ac)

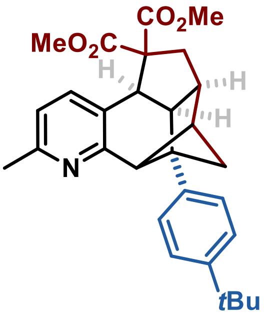

According to **General Procedure D**, the following compound was synthesized using **1ab** (1.0 equiv., 0.2 mmol, 55.1 mg), **2a** (5.0 equiv., 1.0 mmol, 184.2 mg). Reaction mixture was irradiated with blue LEDs for 16 hours. Purified by flash column chromatography (SiO_2; 100:0 to 70:30 pentane/EtOAc) to afford **5ab** (40.4 mg, 44%) as a white fluffy solid. **m.p.** 68-70 °C.

TLC: R_f = 0.2 (80:20 pentane/EtOAc).

NMR Spectroscopy:

^{1}H NMR (400 MHz, $CDCl_3$): δ_H 7.18 – 7.08 (m, 3H), 6.95 – 6.89 (m, 2H), 6.84 (d, J = 7.9 Hz, 1H), 4.12 (d, J = 5.8 Hz, 1H), 3.81 (s, 3H), 3.60 (s, 3H), 3.49 (ddd, J = 8.3, 5.9, 2.0 Hz, 1H), 3.37 – 3.31 (m, 1H), 3.03 – 2.96 (m, 1H), 2.67 (q, J = 8.5 Hz, 1H), 2.49 (s, 3H), 2.27 (ddd, J = 14.0, 8.7, 1.4 Hz, 1H), 2.08 (dd, J = 6.9, 3.0 Hz, 1H), 1.78 (dd, J = 14.1, 8.3 Hz, 1H), 1.70 (d, J = 6.8 Hz, 1H), 1.23 (s, 9H) ppm.

^{13}C NMR (101 MHz, $CDCl_3$): δ_C 171.5, 169.3, 156.8, 155.7, 149.2, 137.4, 136.0, 127.5, 126.6, 125.1, 121.4, 70.7, 54.4, 53.2, 53.0, 52.4, 48.8, 48.6, 48.4, 46.7, 44.6, 34.4, 31.5, 31.4, 23.8 ppm.

HRMS (ESI^+): m/z calc'd for $C_{29}H_{33}NO_4Na$ $[M+Na]^+$: 482.23018, found: 482.23154.

dimethyl-4-methoxy-10-phenyl-7,8,8a,9,10,10a-hexahydro-5,8,10-(epimethanetriyl)cyclobuta[7,8]cycloocta[1,2-*b*]pyridine-6,6(5*H*)-dicarboxylate (5ad)

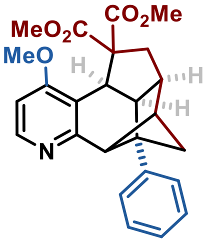

According to **General Procedure D**, the following compound was synthesized using **1ad** (1.0 equiv., 0.2 mmol, 47.0 mg), **2a** (5.0 equiv., 1.0 mmol, 184.2 mg). The reaction mixture was irradiated with blue LEDs for 16 hours. Purified by flash column chromatography (SiO_2; 100:0 to 45:55 pentane/EtOAc) to afford **5ad** (57.0 mg, 68%) as a white solid. **m.p.** 147-152 °C.

TLC: R_f = 0.2 (40:60 pentane/EtOAc).

NMR Spectroscopy:

^{1}H NMR (400 MHz, $CDCl_3$): 8.27 (d, J = 5.8 Hz, 1H), 7.21 – 7.05 (m, 3H), 7.05 – 6.98 (m, 2H), 6.54 (d, J = 5.8 Hz, 1H), 4.40 (d, J = 5.9 Hz, 1H), 3.85 (s, 3H), 3.70 (s, 3H), 3.57 (s, 3H), 3.46 – 3.42 (m, 1H), 3.39 (ddd, J = 8.2, 5.9, 2.0 Hz, 1H), 3.07 – 3.00 (m, 1H), 2.63 (q, J = 8.5 Hz, 1H), 2.36 (ddd, J = 13.7, 8.6, 1.5 Hz, 1H), 2.08 (dd, J = 6.9, 3.0 Hz, 1H), 1.78 (dd, J = 13.6, 8.4 Hz, 1H), 1.72 (d, J = 6.9 Hz, 1H) ppm.

^{13}C NMR (101 MHz, $CDCl_3$): δ_C 172.3, 169.4, 163.6, 158.2, 148.5, 140.4, 128.3, 127.0, 126.5, 118.9, 104.4, 68.4, 55.4, 54.5, 53.0, 52.8, 52.2, 49.2, 49.0, 48.9, 44.7, 42.4, 33.3 ppm.

HRMS (ESI^+): m/z calc'd for $C_{25}H_{25}NO_5Na$ $[M+Na]^+$: 442.16249, found: 442.16294.

X-ray: see Supplementary Figure 24.

dimethyl-10-phenyl-7,8,8a,9,10,10a-hexahydro-5,8,10-(epimethanetriyl)cyclobuta[3,4]cycloocta[1,2-c]pyridine-6,6(5*H*)-dicarboxylate (5ae)

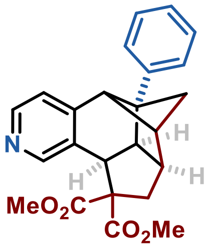

According to **General Procedure D**, the following compound was synthesized using **1ae** (1.0 equiv., 0.2 mmol, 41.0 mg), **2a** (5.0 equiv., 1.0 mmol, 184.2 mg). Reaction mixture was irradiated with blue LEDs for 16 hours. Purified by flash column chromatography (SiO_2; 100:0 to 60:40 pentane/EtOAc) to afford **5ae** (35.0 mg, 45%) as a off white solid. **m.p.** 58-62 °C.

TLC: R_f = 0.4 (40:60 pentane/EtOAc).

^{1}H NMR (400 MHz, $CDCl_3$): δ_H 8.32 (d, *J* = 4.9 Hz, 1H), 8.15 (s, 1H), 7.18 – 7.04 (m, 4H), 7.02 – 6.90 (m, 2H), 4.18 (d, *J* = 5.9 Hz, 1H), 3.82 (s, 3H), 3.66 (s, 3H), 3.57 (ddd, *J* = 8.1, 5.9, 1.9 Hz, 1H), 3.11 – 3.01 (m, 1H), 2.96 – 2.91 (m, 1H), 2.70 (q, *J* = 8.6 Hz, 1H), 2.35 – 2.22 (m, 1H), 2.09 (dd, *J* = 7.0, 3.0 Hz, 1H), 1.80 – 1.73 (m, 2H) ppm.

^{13}C NMR (101 MHz, $CDCl_3$): δ_C 171.5, 169.1, 148.5, 147.9, 147.1, 140.2, 131.0, 128.4, 126.8, 126.7, 123.4, 70.6, 53.9, 53.2, 52.7, 50.9, 49.0, 48.7, 48.3, 44.7, 44.6, 31.5 ppm.

HRMS (ESI$^+$): m/z calc'd for $C_{24}H_{23}NO_4Na$ $[M+Na]^+$: 412.15193, found: 412.15318.

dimethyl-10-(4-(trifluoromethyl)phenyl)-7,8,8a,9,10,10a-hexahydro-5,8,10-(epimethanetriyl)cyclobuta[3,4]cycloocta[1,2-*c*]pyridine-6,6(5*H*)-dicarboxylate (5af)

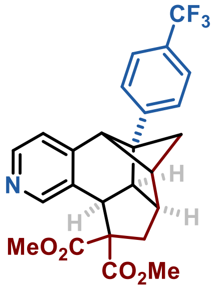

According to **General Procedure D**, the following compound was synthesized using **1af** (1.0 equiv., 0.2 mmol, 54.6 mg), **2a** (5.0 equiv., 1.0 mmol, 184.2 mg). Reaction mixture was irradiated with blue LEDs for 16 hours. Purified by flash column chromatography (SiO_2; 100:0 to 95:05 CH_2Cl_2/MeOH) to afford **5af** (43.9 mg, 48%) as a white solid.

TLC: R_f = 0.7 (90:10 CH_2Cl_2/MeOH).

NMR Spectroscopy:

^{1}H NMR (400 MHz, $CDCl_3$): δ_H 8.34 (d, *J* = 4.9 Hz, 1H), 8.17 (s, 1H), 7.40 (d, *J* = 8.1 Hz, 2H), 7.12 (d, *J* =

5.0 Hz, 1H), 7.06 (d, J = 8.1 Hz, 2H), 4.15 (d, J = 5.9 Hz, 1H), 3.82 (s, 3H), 3.67 (s, 3H), 3.59 (ddd, J = 8.2, 5.9, 1.9 Hz, 1H), 3.19 – 3.05 (m, 1H), 3.04 – 2.93 (m, 1H), 2.73 (q, J = 8.5 Hz, 1H), 2.28 (ddd, J = 14.2, 8.7, 1.4 Hz, 1H), 2.10 (dd, J = 7.0, 3.0 Hz, 1H), 1.90 – 1.65 (m, 2H) ppm.

^{13}C{^{19}F} NMR (126 MHz, $CDCl_3$): δ_C 171.4, 168.9, 148.3, 147.8, 147.0, 144.2, 130.9, 129.0, 127.1, 125.4, 124.2, 123.4, 70.6, 53.6, 53.2, 52.8, 50.8, 49.2, 49.0, 48.3, 44.6, 44.5, 31.5 ppm.

^{19}F NMR (376 MHz, $CDCl_3$): δ -62.60 ppm.

HRMS (ESI$^+$): m/z calc'd for $C_{25}H_{22}NO_4F_3Na$ [M+Na]$^+$: 480.13931, found: 480.13789.

dimethyl-10-(4-cyanophenyl)-7,8,8a,9,10,10a-hexahydro-5,8,10-(epimethanetriyl)cyclobuta[3,4]cycloocta[1,2-*d*]pyrimidine-6,6(5*H*)-dicarboxylate (5ag)

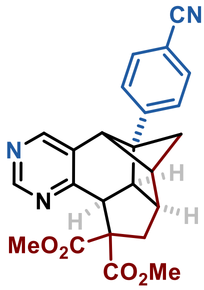

According to **General Procedure D**, the following compound was synthesized using **1ag** (1.0 equiv., 0.2 mmol, 46.2 mg), **2a** (5.0 equiv., 1.0 mmol, 184.2 mg). The reaction mixture was irradiated with blue LEDs for 16 hours. Purified by flash column chromatography (SiO_2; 100:0 to 40:60 pentane/EtOAc) to afford **5ag** (29.9 mg, 36%) as a white solid.

TLC: R_f = 0.4 (40:60 pentane/EtOAc).

NMR Spectroscopy:

^{1}H NMR (400 MHz, $CDCl_3$): δ_H 8.84 (s, 1H), 8.43 (s, 1H), 7.41 (d, J = 7.9 Hz, 2H), 7.09 (d, J = 7.9 Hz, 2H), 4.08 (d, J = 5.6 Hz, 1H), 3.76 (s, 3H), 3.64 (s, 3H), 3.60 – 3.53 (m, 1H), 3.13 – 3.04 (m, 1H), 3.01 – 2.90 (m, 1H), 2.70 (q, J = 8.7 Hz, 1H), 2.25 (dd, J = 14.3, 8.8 Hz, 1H), 2.14 (dd, J = 7.2, 3.0 Hz, 1H), 1.79 – 1.67 (m, 2H) ppm.

^{13}C NMR (101 MHz, $CDCl_3$): δ_C 171.2, 168.6, 165.2, 156.9, 155.2, 145.0, 132.4, 130.6, 127.7, 118.7, 110.9, 69.6, 53.3, 53.1, 52.8, 49.2, 49.0, 48.9, 48.1, 48.0, 44.3, 31.5 ppm.

HRMS (ESI$^+$): m/z calc'd for $C_{24}H_{21}N_3O_4Na$ [M+Na]$^+$: 438.14243, found: 438.14140.

X-ray: **see Supplementary Figure 25**.

dimethyl-10-(4-(trifluoromethyl)phenyl)-7,8,8a,9,10,10a-hexahydro-5,8,10-(epimethanetriyl)cyclobuta[3,4]cycloocta[1,2-*d*]pyrimidine-6,6(5*H*)-dicarboxylate (5ah)

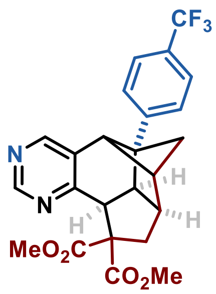

According to **General Procedure D**, the following compound was synthesized using **1ah** (1.0 equiv., 0.2 mmol, 54.8 mg), **2a** (5.0 equiv., 1.0 mmol, 184.2 mg). The reaction mixture was irradiated with blue LEDs for 16 hours. Purified by flash column chromatography (SiO_2; 100:0 to 60:40 pentane/EtOAc) to afford **5ah** (28.4 mg, 31%) as a white solid. **m.p.** 162-168 °C.

TLC: R_f = 0.6 (50:50 pentane/EtOAc).

NMR Spectroscopy:

^{1}H NMR (400 MHz, $CDCl_3$): δ_H 8.90 (s, 1H), 8.47 (s, 1H), 7.43 (d, *J* = 8.1 Hz, 2H), 7.16 (d, *J* = 8.0 Hz, 2H), 4.16 (d, *J* = 5.6 Hz, 1H), 3.83 (s, 3H), 3.71 (s, 3H), 3.63 (ddd, *J* = 8.3, 5.8, 2.2 Hz, 1H), 3.17 – 3.12 (m, 1H), 3.04 – 2.99 (m, 1H), 2.76 (q, *J* = 8.6 Hz, 1H), 2.32 (ddd, *J* = 14.4, 8.9, 1.4 Hz, 1H), 2.20 (dd, *J* = 7.1, 3.0 Hz, 1H), 1.84 – 1.77 (m, 2H) ppm.

^{13}C{^{19}F} NMR (126 MHz, $CDCl_3$): δ_C 171.3, 168.7, 165.2, 156.9, 155.3, 143.6, 130.7, 129.3, 127.3, 125.5, 124.1, 69.7, 53.3, 53.0, 52.8, 49.2, 49.1, 49.0, 48.2, 48.0, 44.4, 31.6 ppm.

^{19}F NMR (376 MHz, CDCl3): δ_F -62.44.ppm.

HRMS (ESI$^+$): m/z calc'd for $C_{24}H_{21}F_3N_2O_4Na$ $[M+Na]^+$: 481.13456, found: 481.13333.

dibenzyl-3-methyl-10-phenyl-7,8,8a,9,10,10a-hexahydro-5,8,10-(epimethanetriyl)cyclobuta[3,4]cycloocta[1,2-*b*]pyridine-6,6(5*H*)-dicarboxylate (5ai)

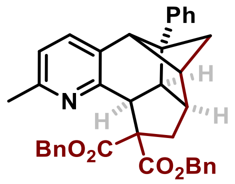

According to **General Procedure D**, the following compound was synthesized using **1b** (1.0 equiv., 0.2 mmol, 43.8 mg), **2b** (5.0 equiv., 1.0 mmol, 336.4 mg). Reaction mixture was irradiated with blue LEDs for 16 hours. Purified by flash column chromatography (SiO_2; 100:0 to 97:03 pentane/EtOAc) to afford **5ai** (46.7 mg, 42%) as a white solid.

TLC: R_f = 0.8 (90:10 pentane/EtOAc).

NMR Spectroscopy:

^{1}H NMR (400 MHz, $CDCl_3$): δ_H 7.30 – 7.19 (m, 11H), 7.15 – 7.08 (m, 2H), 7.08 – 6.98 (m, 3H), 6.82 (d, *J* = 7.7 Hz, 1H), 5.21 – 4.98 (m, 4H), 4.25 (d, *J* = 5.8 Hz, 1H), 3.52 (ddd, *J* = 8.4, 5.8, 2.0 Hz, 1H), 3.06 – 2.96 (m, 1H), 2.90 – 2.82 (m, 1H), 2.60 (q, *J* = 8.7 Hz, 1H), 2.35 (s, 3H), 2.24 (dd, *J* = 14.0, 8.9 Hz, 1H), 2.03 (dd, *J* = 6.9, 3.0 Hz, 1H), 1.83 (dd, *J* = 14.0, 8.3 Hz, 1H), 1.65 (d, *J* = 6.8 Hz, 1H) ppm.

^{13}C NMR (101 MHz, $CDCl_3$): δ_C 171.2, 168.7, 156.1, 155.3, 140.8, 136.1, 136.0, 135.7, 129.9, 128.6, 128.5, 128.3, 128.3, 128.2, 128.0, 128.0, 127.0, 126.5, 121.3, 70.2, 67.4, 66.8, 53.3, 50.9, 49.8, 49.4, 48.8, 48.0, 44.4, 31.8, 24.4 ppm.

HRMS (ESI$^+$): m/z calc'd for $C_{37}H_{33}NO_4Na$ $[M+Na]^+$: 578.23018, found: 578.23026.

di-*tert*-butyl-3-methyl-10-phenyl-7,8,8a,9,10,10a-hexahydro-5,8,10-(epimethanetriyl)cyclobuta[3,4]cycloocta[1,2-*b*]pyridine-6,6(5*H*)-dicarboxylate (5aj)

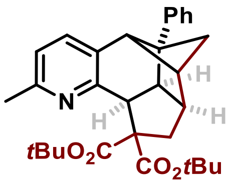

According to **General Procedure D**, the following compound was synthesized using **1b** (1.0 equiv., 0.2 mmol, 43.8 mg), **2c** (5.0 equiv., 1.0 mmol, 268.3 mg). Reaction mixture was irradiated with blue LEDs for 16 hours. Purified by flash column chromatography (SiO_2; 100:0 to 98:02 pentane/EtOAc) to afford **5ai** (20.5 mg, 21%) as a white solid.

TLC: R_f = 0.9 (90:10 pentane/EtOAc).

NMR Spectroscopy:

^{1}H NMR (400 MHz, $CDCl_3$): δ_H 7.27 (d, *J* = 3.7 Hz, 1H), 7.16 – 7.09 (m, 2H), 7.09 – 7.01 (m, 3H), 6.83 (d, *J* = 7.7 Hz, 1H), 4.19 – 4.13 (m, 1H), 3.55 (ddd, *J* = 8.3, 5.7, 2.1 Hz, 1H), 3.06 – 2.97 (m, 1H), 2.91 – 2.85 (m, 1H), 2.60 (q, *J* = 8.7 Hz, 1H), 2.38 (s, 3H), 2.16 (dd, *J* = 13.8, 8.7 Hz, 1H), 2.08 (dd, *J* = 6.9, 3.0 Hz, 1H), 1.77 – 1.66 (m, 2H), 1.53 (s, 9H), 1.49 (s, 9H) ppm.

^{13}C NMR (101 MHz, $CDCl_3$): δ_C 170.9, 167.9, 155.9, 140.9, 136.1, 132.3, 129.9, 128.2, 127.1, 126.3, 121.4, 71.0, 53.3, 51.5, 49.8, 49.4, 48.4, 47.8, 44.0, 32.3, 28.2, 28.1, 24.2 ppm.

HRMS (ESI$^+$): m/z calc'd for $C_{31}H_{37}NO_4Na$ $[M+Na]^+$: 510.26148, found: 510.26147.

diethyl-3-methyl-10-phenyl-7,8,8a,9,10,10a-hexahydro-5,8,10-(epimethanetriyl)cyclobuta[3,4]cycloocta[1,2-*b*]pyridine-6,6(5*H*)-dicarboxylate (5ak)

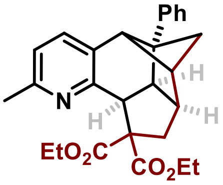

According to **General Procedure D**, the following compound was synthesized using **1b** (1.0 equiv., 0.2 mmol, 43.8 mg), **2d** (5.0 equiv., 1.0 mmol, 212.2 mg). The reaction mixture was irradiated with blue LEDs for 16 hours. Purified by flash column chromatography (SiO_2; 100:0 to 90:10 Pentane/EtOAc) to afford **5ak** (41.4 mg, 48%) as a white solid. **m.p.** 128-133 °C.

TLC: R_f = 0.2 (90:10 pentane/EtOAc).

NMR Spectroscopy:

^{1}H NMR (400 MHz, $CDCl_3$): δ_H 7.27 (d, *J* = 6.0 Hz, 1H), 7.18 – 7.02 (m, 5H), 6.85 (d, *J* = 7.7 Hz, 1H), 4.37 – 4.17 (m, 4H), 4.15 – 4.02 (m, 1H), 3.55 (ddd, *J* = 8.7, 5.8, 2.0 Hz, 1H), 3.02 (s, 1H), 2.90 (d, *J* = 3.6 Hz, 1H), 2.67 (q, *J* = 8.7 Hz, 1H), 2.40 (s, 3H), 2.23 (dd, *J* = 14.1, 8.9 Hz, 1H), 2.14 – 2.06 (m, 1H), 1.82 (dd, *J* = 14.0, 8.4 Hz, 1H), 1.71 (dd, *J* = 6.7, 2.7 Hz, 1H), 1.38 – 1.21 (m, 6H) ppm.

^{13}C NMR (101 MHz, $CDCl_3$): δ_C 171.6, 169.0, 155.9, 155.5, 140.9, 136.1, 130.1, 128.3, 127.0, 126.5, 121.3, 70.1, 61.6, 61.1, 53.3, 51.2, 49.7, 49.5, 48.8, 47.7, 44.4, 31.7, 24.3, 14.2, 14.1 ppm.

HRMS (ESI^+): m/z calc'd for $C_{27}H_{29}NO_4Na$ $[M+Na]^+$: 454.19888, found: 454.19880.

dicyclohexyl-3-methyl-10-phenyl-7,8,8a,9,10,10a-hexahydro-5,8,10-(epimethanetriyl)cyclobuta[3,4]cycloocta[1,2-*b*]pyridine-6,6(5*H*)-dicarboxylate (5al)

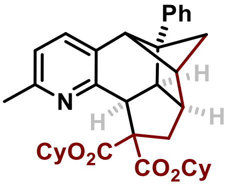

According to **General Procedure D,** the following compound was synthesized using **1b** (1.0 equiv., 0.2 mmol, 43.8 mg), **2e** (5.0 equiv., 1.0 mmol, 320.4 mg). Reaction mixture was irradiated with blue LEDs for 16 hours. Purified by flash column chromatography (SiO_2; 100:0 to 96:04 pentane/EtOAc) to afford **5al** (24.8 mg, 23%) as a white solid.

TLC: R_f = 0.7 (90:10 pentane/EtOAc).

NMR Spectroscopy:

^{1}H NMR (400 MHz, $CDCl_3$): δ_H 7.33 – 7.23 (m, 1H), 7.19 – 7.12 (m, 2H), 7.11 – 7.01 (m, 3H), 6.87 – 6.79 (m, 1H), 4.98 – 4.84 (m, 1H), 4.75 – 4.56 (m, 1H), 4.19 (br. s, 1H), 3.54 (ddd, *J* = 8.4, 5.7, 2.1 Hz, 1H), 3.09 – 3.01 (m, 1H), 2.95 – 2.83 (m, 1H), 2.64 (q, *J* = 8.7 Hz, 1H), 2.37 (s, 3H), 2.29 – 2.16 (m, 1H), 2.12 – 1.97 (m, 2H), 1.94 – 1.68 (m, 9H), 1.67 – 1.59 (m, 3H), 1.58 – 1.46 (m, 4H), 1.46 – 1.29 (m, 5H) ppm.

^{13}**C NMR** (101 MHz, $CDCl_3$): δ_C 171.1, 168.4, 155.9, 155.7, 140.9, 136.0, 129.8, 128.3, 127.0, 126.4, 121.2, 73.7, 70.4, 53.4, 50.9, 49.5, 48.7, 48.2, 44.4, 31.6, 31.6, 31.4, 31.4, 25.6, 25.5, 23.8, 23.8, 23.8 ppm.

HRMS (ESI$^+$): m/z calc'd for $C_{35}H_{41}NO_4Na$ $[M+Na]^+$: 562.29278, found: 562.29274.

bis(2-chloroethyl)-3-methyl-10-phenyl-7,8,8a,9,10,10a-hexahydro-5,8,10-(epimethanetriyl)cyclobuta[3,4]cycloocta[1,2-*b*]pyridine-6,6(5*H*)-dicarboxylate (5am)

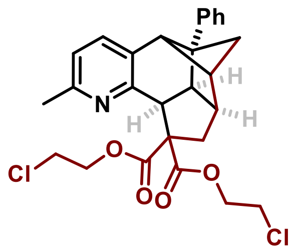

According to **General Procedure D**, the following compound was synthesized using **1b** (1.0 equiv., 0.2 mmol, 43.8 mg), **2f** (5.0 equiv., 1.0 mmol, 281.1 mg). The reaction mixture was irradiated with blue for 16 hours. Purified by flash column chromatography (SiO_2; 100:0 to 90:10 pentane/EtOAc) to afford **5am** (40.0 mg, 40%) as a white solid. **m.p.** 145-149 °C.

TLC: R_f = 0.2 (90:10 pentane/EtOAc).

NMR Spectroscopy:

^{1}**H NMR** (400 MHz, $CDCl_3$): δ_H 7.45 – 7.30 (m, 1H), 7.19 – 7.05 (m, 5H), 6.93 – 6.82 (m, 1H), 4.57 – 4.27 (m, 5H), 3.83 – 3.67 (m, 4H), 3.61 (ddd, *J* = 8.2, 5.7, 1.9 Hz, 1H), 3.07 (br. s, 1H), 2.97 – 2.91 (m, 1H), 2.73 (q, *J* = 8.6 Hz, 1H), 2.43 (s, 3H), 2.37 – 2.28 (m, 1H), 2.10 (dd, *J* = 7.0, 3.0 Hz, 1H), 1.86 – 1.78 (m, 1H), 1.73 (d, *J* = 6.9 Hz, 1H) ppm.

^{13}**C NMR** (101 MHz, $CDCl_3$): δ_C 170.9, 168.4, 156.0, 155.1, 140.6, 136.3, 130.0, 128.3, 127.0, 126.6, 121.5, 69.9, 65.2, 64.8, 53.3, 50.9, 49.8, 49.4, 48.9, 47.9, 44.4, 41.6, 41.4, 31.7, 24.3 ppm.

HRMS (ESI$^+$): m/z calc'd for $C_{27}H_{27}Cl_2NO_4Na$ $[M+Na]^+$: 522.12093, found: 522.12127.

bis(2-ethoxyethyl)-3-methyl-10-phenyl-7,8,8a,9,10,10a-hexahydro-5,8,10-(epimethanetriyl)cyclobuta[3,4]cycloocta[1,2-*b*]pyridine-6,6(5*H*)-dicarboxylate (5an)

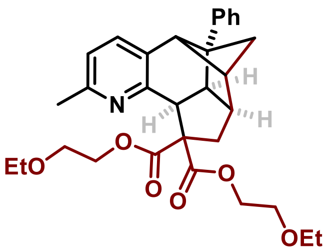

According to **General Procedure D** the following compound was synthesized using **1b** (1.0 equiv., 0.2 mmol, 43.8 mg), **2g** (5.0 equiv., 1.0 mmol, 300.3 mg). The reaction mixture was irradiated with blue LEDs for 16

hours. Purified by flash column chromatography (SiO_2; 100:0 to 90:10 pentane/EtOAc) to afford **5an** (53.0 mg, 51%) as a white solid.

TLC: R_f = 0.3 (90:10 pentane/EtOAc).

NMR Spectroscopy:

^{1}H NMR (400 MHz, $CDCl_3$): δ_H 7.26 (d, J = 7.7 Hz, 1H), 7.20 – 7.00 (m, 5H), 6.83 (d, J = 7.7 Hz, 1H), 4.47 – 4.20 (m, 4H), 4.14 (ddd, J = 11.5, 5.6, 4.5 Hz, 1H), 3.71 – 3.46 (m, 9H), 3.07 – 2.98 (m, 1H), 2.96 – 2.83 (m, 1H), 2.68 (q, J = 8.6 Hz, 1H), 2.38 (s, 3H), 2.28 (dd, J = 13.1, 9.5 Hz, 1H), 2.07 (dd, J = 6.9, 2.9 Hz, 1H), 1.82 (dd, J = 14.0, 8.4 Hz, 1H), 1.70 (d, J = 6.9 Hz, 1H), 1.17 (t, J = 7.0 Hz, 6H) ppm.

^{13}C NMR (101 MHz, $CDCl_3$): δ_C 171.3, 168.7, 155.9, 155.3, 140.8, 136.0, 129.8, 128.2, 126.9, 126.4, 121.2, 70.0, 68.2, 67.9, 66.6, 64.6, 64.4, 53.2, 51.0, 49.7, 49.3, 48.7, 47.8, 44.3, 31.6, 24.3, 15.3 ppm.

HRMS (ESI$^+$): m/z calc'd for $C_{31}H_{37}NO_6Na$ $[M+Na]^+$: 542.25131, found: 542.25087.

ethyl-6-cyano-3-methyl-10-(4-(trifluoromethyl)phenyl)-5,6,7,8,8a,9,10,10a-octahydro-5,8,10-(epimethanetriyl)cyclobuta[3,4]cycloocta[1,2-*b*]pyridine-6-carboxylate (5ao)

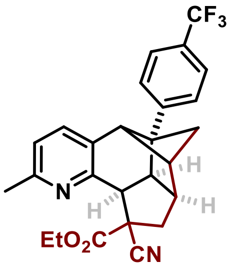

According to **General Procedure D**, the following compound was synthesized **1h** (1.0 equiv., 0.2 mmol, 57.5 mg), **2h** (5.0 equiv., 1.0 mmol, 165.2 mg). The reaction mixture was irradiated with blue LEDs for 16 hours. Purified by flash column chromatography (SiO_2; 100:0 to 90:10 pentane/EtOAc) to afford **5ao** (29.8 mg, 33% d.r.: 66:34) as a white solid. The structure of the major diastereomer was confirmed by X-ray crystallography.

ethyl-6-cyano-3-methyl-10-(4-(trifluoromethyl)phenyl)-5,6,7,8,8a,9,10,10a-octahydro-5,8,10-(epimethanetriyl)cyclobuta[3,4]cycloocta[1,2-*b*]pyridine-6-carboxylate (major diastereomer)

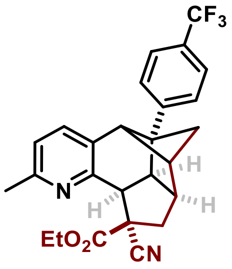

Yield: (19.9 mg, 22%) as a white solid. **m.p.** 117-119 °C.

TLC: R_f = 0.8 (90:10 pentane/EtOAc).

NMR Spectroscopy of major diastereomer:

^{1}H NMR (400 MHz, $CDCl_3$): δ_H 7.42 (d, *J* = 8.1 Hz, 2H), 7.35 (d, *J* = 7.8 Hz, 1H), 7.12 (d, *J* = 8.1 Hz, 2H), 6.93 (d, *J* = 7.7 Hz, 1H), 4.33 – 4.20 (m, 2H), 4.01 (d, *J* = 5.7 Hz, 1H), 3.75 (ddd, *J* = 8.1, 5.7, 2.0 Hz, 1H), 3.14 (t, *J* = 2.5 Hz, 1H), 3.09 – 3.00 (m, 2H), 2.41 (s, 3H), 2.13 (ddd, *J* = 16.0, 7.9, 2.2 Hz, 2H), 1.92 – 1.80 (m, 2H), 1.39 (t, *J* = 7.2 Hz, 3H) ppm.

^{13}C NMR (101 MHz, $CDCl_3$): δ_C 165.8, 157.0, 152.3, 144.2, 136.3, 129.9, 129.1 (q, *J* = 32.2 Hz), 127.1, 125.4 (q, *J* = 3.8 Hz), 124.2 (q, *J* = 272.0) 122.3, 120.5, 62.6, 57.7, 53.4, 52.0, 50.6, 50.0, 49.1, 48.1, 44.8, 32.2, 24.2, 14.0 ppm.

^{19}F NMR (376 MHz, CDCl3): δ_F -62.47 ppm.

HRMS (ESI^+): m/z calc'd for $C_{26}H_{23}F_3N_2O_2Na$ $[M+Na]^+$: 475.16038, found: 475.16155.

X-ray: **see Supplementary Figure 26**.

ethyl-6-cyano-3-methyl-10-(4-(trifluoromethyl)phenyl)-5,6,7,8,8a,9,10,10a-octahydro-5,8,10-(epimethanetriyl)cyclobuta[3,4]cycloocta[1,2-*b*]pyridine-6-carboxylate (Minor diastereomer)

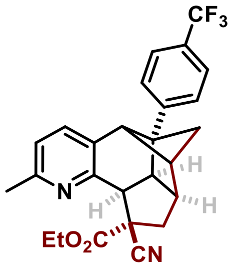

Yield: (9.9 mg, 11%) as a white solid.

TLC: R_f = 0.7 (90:10 pentane/EtOAc).

NMR Spectroscopy of Minor diastereomer:

^{1}H NMR (400 MHz, $CDCl_3$): δ_H 7.46 – 7.36 (m, 3H), 7.09 (d, *J* = 8.1 Hz, 2H), 7.02 (d, *J* = 7.7 Hz, 1H), 4.43 – 4.30 (m, 2H), 4.07 (s, 1H), 3.28 – 3.20 (m, 1H), 3.16 – 3.08 (m, 1H), 3.05 – 2.96 (m, 2H), 2.59 (s, 3H), 2.54 (ddd, *J* = 13.4, 8.8, 1.3 Hz, 1H), 2.13 (dd, *J* = 7.1, 3.0 Hz, 1H), 1.82 (d, *J* = 7.0 Hz, 1H), 1.50 (dd, *J* = 13.4, 7.7 Hz, 1H), 1.38 (t, *J* = 7.1 Hz, 3H) ppm.

^{13}C NMR (101 MHz, $CDCl_3$): δ_C 168.0, 157.4, 152.8, 144.2, 136.6, 131.2, 129.0 (q, *J* = 32.5 Hz), 127.0, 125.5 (q, *J* = 3.8 Hz), 125.4 (q, *J* = 271.8 Hz), 122.5, 117.2, 63.4, 56.6, 53.6, 50.7, 50.1, 49.1, 48.9, 47.9, 46.2, 34.1, 23.9, 14.2 ppm.

^{19}F NMR (376 MHz, CDCl3): δ_F -62.60 ppm.

HRMS (ESI^+): m/z calc'd for $C_{26}H_{23}F_3N_2O_2Na[M+Na]^+$: 475.16038, found: 475.16039.

tetraethyl-3-methyl-10-phenyl-5,6,7,8,8a,9,10,10a-octahydro-5,8,10-

(epimethanetriyl)cyclobuta[3,4]cycloocta[1,2-*b*]pyridine-6,6-diyl)bis(phosphonate) (5ap)

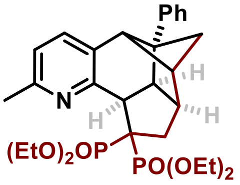

According to **General Procedure D**, the following compound was synthesized using **1b** (1.0 equiv., 0.2 mmol, 43.8 mg), **2i** (5.0 equiv., 1.0 mmol, 340.3 mg). The reaction mixture was irradiated with blue LEDs for 16 hours. Purified by flash column chromatography (SiO_2; 100:0 to 90:10 pentane/EtOAc) to afford **5ap** (NMR yield, 41%). The product could not be isolated by flash column chromatography, and clean spectra could not be obtained. The crude NMR spectrum has been included in the spectroscopic data section.

diethyl-8a-methyl-10-(4-(trifluoromethyl)phenyl)-7,8,8a,9,10,10a-hexahydro-5,8,10-(epimethanetriyl)cyclobuta[3,4]cycloocta[1,2-*b*]pyridine-6,6(5*H*)-dicarboxylate (5aq)

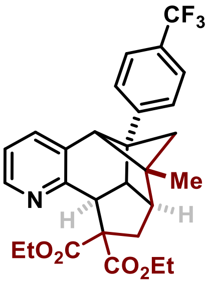

According to **General Procedure D,** the following compound was synthesized using **1i** (1.0 equiv., 0.2 mmol, 54.6 mg), **2j** (5.0 equiv., 1.0 mmol, 226.3 mg). The reaction mixture was irradiated with blue LEDs for 24 hours. Purified by flash column chromatography (SiO_2; 100:0 to 75:25 pentane/EtOAc) to afford **5aq** (28.0 mg, 28%) as a colourless oil along with some unidentified side product around 12%.

TLC: R_f = 0.5 (60:40 pentane/EtOAc).

NMR Spectroscopy:

^{1}H NMR (599 MHz, $CDCl_3$): δ_H 8.40 (dd, *J* = 4.7, 1.8 Hz, 1H), 7.59 – 7.54 (m, 2H), 7.40 – 7.31 (m, 3H), 7.07 (dd, *J* = 7.7, 4.6 Hz, 1H), 4.42 (d, *J* = 8.9 Hz, 1H), 4.34 – 4.27 (m, 1H), 4.27 – 4.08 (m, 3H), 3.75 (ddd, *J* = 12.7, 5.1, 1.6 Hz, 1H), 3.65 (ddd, *J* = 12.4, 8.9, 1.6 Hz, 1H), 2.79 – 2.64 (m, 2H), 2.58 – 2.45 (m, 2H), 2.37 – 2.30 (m, 1H), 1.30 (t, *J* = 7.1 Hz, 3H), 1.26 (t, *J* = 7.1 Hz, 3H), 1.21 (s, 3H) ppm.

^{13}C{^{19}F} NMR (151 MHz, $CDCl_3$): δ_C 172.1, 169.6, 154.1, 148.6, 147.8, 136.8, 133.4, 128.3, 126.8, 125.4, 124.4, 122.2, 70.2, 61.8, 61.4, 48.1, 44.8, 43.5, 42.6, 42.4, 40.0, 35.4, 32.5, 23.7, 14.2, 14.0 ppm.

^{19}F NMR (563 MHz, $CDCl_3$): δ_F -62.40 ppm.

HRMS (ESI$^+$): m/z calc'd for $C_{28}H_{28}NO_4F_3Na$ $[M+Na]^+$: 522.18626, found: 522.18645.

dimethyl-1-phenyl-2,2a,3,4,6,10b-hexahydro-1,3,6-(epimethanetriyl)benzo[a]cyclobuta[c][8]annulene-5,5(1H)-dicarboxylate (5ar)

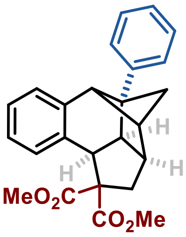

According to modified **General Procedure D,** the following compound was synthesized using **1aj** (1.0 equiv., 0.2 mmol, 40.8 mg), **2a** (5.0 equiv., 1.0 mmol, 184.2 mg), and 1:1 mixture of HFIP (1.0 mL) and EtOAc(1.0 ml). Reaction mixture was irradiated with blue LEDs for 16 hours. Purified by flash column chromatography (SiO_2; 100:0 to 95:05 pentane/EtOAc) to afford **5ar** (45.0 mg, 58%) as a white solid.

TLC: R_f = 0.7 (90:10 pentane/EtOAc).

NMR Spectroscopy:

^{1}H NMR (400 MHz, $CDCl_3$): δ_H 7.19 – 7.02 (m, 6H), 6.99 – 6.95 (m, 2H), 6.95 – 6.90 (m, 1H), 4.14 (d, *J* = 5.8 Hz, 1H), 3.80 (s, 3H), 3.60 (s, 3H), 3.46 (ddd, *J* = 8.3, 5.9, 1.9 Hz, 1H), 3.10 – 3.03 (m, 1H), 2.91 – 2.81 (m, 1H), 2.65 (q, *J* = 8.6 Hz, 1H), 2.22 (ddd, *J* = 13.8, 8.7, 1.4 Hz, 1H), 2.02 (dd, *J* = 6.8, 2.9 Hz, 1H), 1.84 (dd, *J* = 13.8, 8.4 Hz, 1H), 1.71 (d, *J* = 6.8 Hz, 1H) ppm.

^{13}C NMR (101 MHz, $CDCl_3$): δ_C 172.1, 169.6, 141.5, 138.3, 135.0, 128.9, 128.2, 127.5, 126.8, 126.8, 126.6, 126.3, 70.9, 53.7, 53.0, 52.3, 51.8, 49.5, 48.2, 48.0, 47.5, 44.6, 31.4 ppm.

HRMS (ESI$^+$): m/z calc'd for $C_{25}H_{24}O_4Na$ $[M+Na]^+$: 411.15668, found: 411.15660.

Substrate Limitations

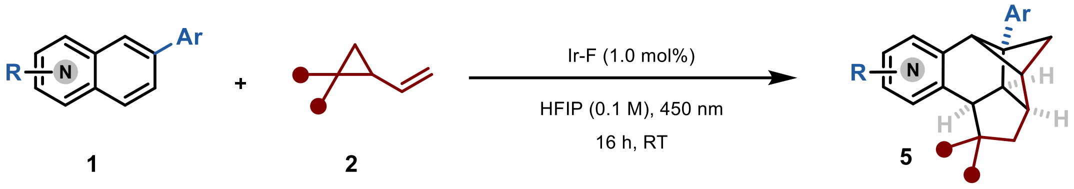

In the following figure, we present the limitations of the substrate scope.

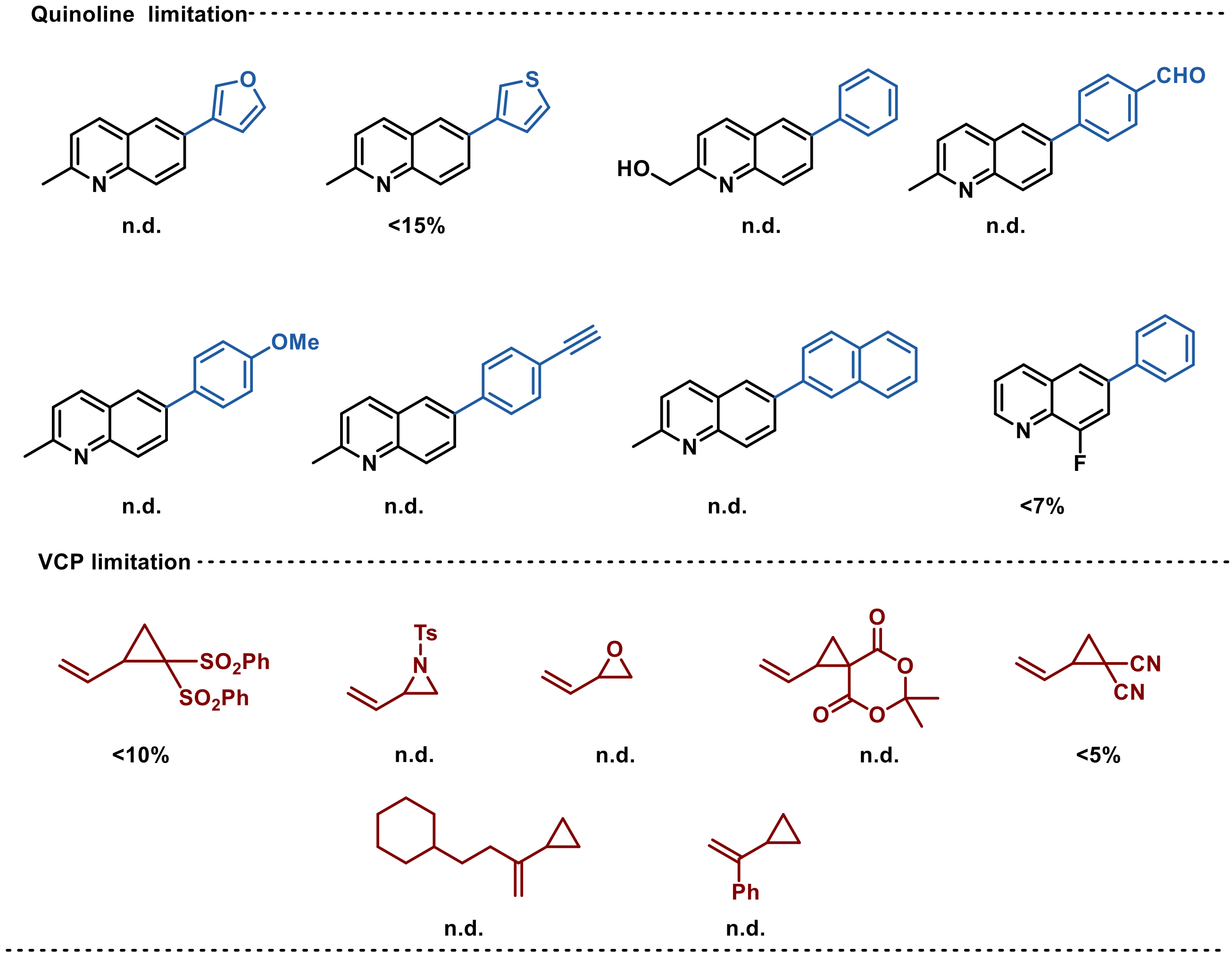

Supplementary Figure 3: Substrate scope limitations

In some cases, quinoline remains unreacted under standard photocatalytic conditions, while in case of VCPs, either decomposition or polymerization was observed. Additionally, some reactions produced very low yields. All the yields were calculated from crude ^{1}H NMR analysis using CH_2Br_2 as an internal standard.

Time Course Study

Quinoline **1b** (1.0 equiv., 1.0 mmol, 219.3 mg) and **Ir-F** (1.0 mol%, 11.2 mg) were added to an oven-dried 50.0 mL Schlenk tube equipped with a Teflon-coated magnetic stir bar. The tube was evacuated and backfilled with argon three times. Under an argon atmosphere, HFIP (10.0 mL, 0.1 M) and **2a** (5 equiv., 5.0 mmol, 920.9 mg) were added consecutively, and the tube was sealed tightly. Eight oven-dried Schlenk tubes were charged with a magnetic stir bar and connected with Schlenk line. The tubes were evacuated and backfilled with argon three times. To each Schlenk tube 1.0 ml of stock solution was added under argon and the tube was sealed tightly. The reaction mixtures were stirred under irradiation with blue LEDs (30 W, λ_{max} = 450 nm) for the indicated time. Then, the solvent was evaporated under reduced pressure and ^{1}H NMR yield was determined using CH_2Br_2 as an internal standard.

Supplementary Table 1: Kinetic study: Effect of reaction time on product yield

Time (h)	Yield of the reaction (%)
0	0
0.5	7
1	13
3	30
6	40
9	48
12	54
16	56
24	56

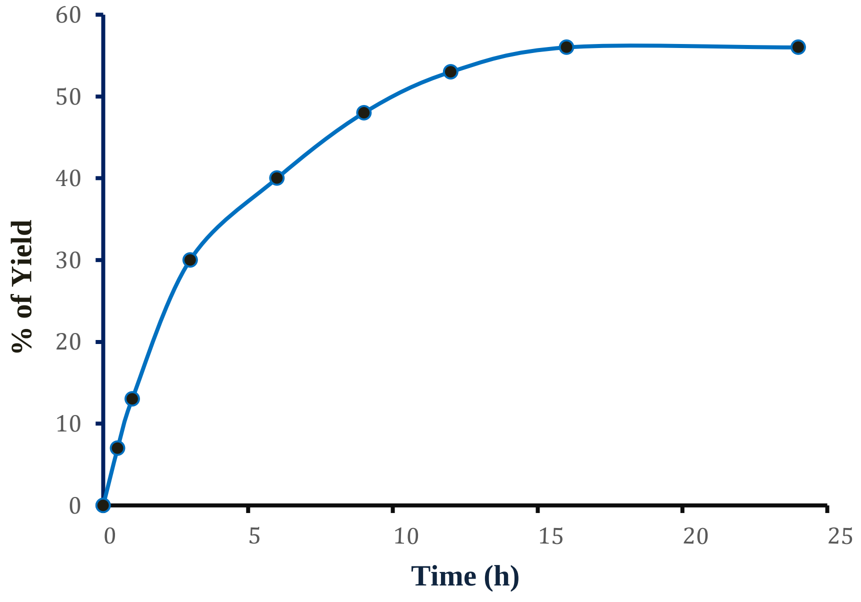

Supplementary Figure 4: Reaction progress over time

Additive-Based Robustness Screening

The functional group tolerance and robustness screening was conducted following the approach described by Glorius and coworkers,[11] with slight modifications to the conditions outlined in **General Procedure D**. A variety of additives bearing different functional groups and heterocycles were tested in an additive-based robustness screening. Consequently, the compatibility of these functional groups and the stability of the additives within the presented methodology were assessed based on the yield of product and recovery of additives.

Preparation of stock solution:

1b (1.0 equiv., 1.8 mmol, 394.7 mg) and **Ir-F** (1.0 mol%, 20.2 mg) were added to an oven-dried 50.0 mL Schlenk tube equipped with a Teflon-coated magnetic stir bar. The tube was evacuated and backfilled with argon three times. Under argon atmosphere, HFIP (18.0 mL, 0.1 M) and **2a** (5.0 equiv., 9.0 mmol, 1.66 g) were added consecutively, and the tube was sealed tightly.

Sample preparation with different additives:

Fifteen oven-dried 10.0 mL Schlenk tubes equipped with a Teflon-coated magnetic stir bar were evacuated and backfilled with argon three times. Under argon freshly prepared stock solution of 1.0 mL was added to the Schlenk tube. After that, respective additives (1.0 equiv., 0.1 mmol) were added to 14 of the Schlenk tubes and a control reaction without an additive were also prepared simultaneously. The reaction mixtures were stirred under irradiation with blue LEDs (30W, λ_{max} = 450 nm) for 16 hours. Then GC-FID samples were prepared by taking 30 μL of the crude reaction from every Schlenk tube and mixing with 30 μL of 0.1 M mesitylene solution. From the calibration curve the yield of the product and recovery of additives were calculated as shown in **Table S2**.

Recovered additive: green (>66%), yellow (33-66%), red (<33%). Product yield (with respect to the control reaction): green (>42%), yellow (21-42%), red (<21%).

Supplementary Table 2: Results of the additive-based robustness screening.

Entry	Additive	Additive Yield	Product Yield	Name
1	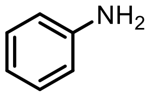	0	0	aniline
2	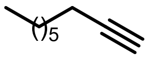	99	41	1-decyne

3	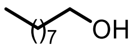	99	48	1-nonanol
4	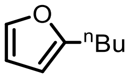	58	2	2-butylfuran
5	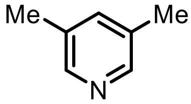	95	43	3,5-dimethylpyridine
6	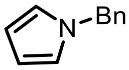	72	2	benzylpyrrole
7	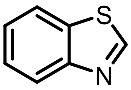	88	11	benzothiazole
8	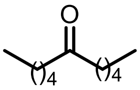	97	46	alkylketone
9	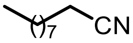	99	46	decanenitrile
10	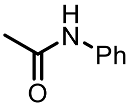	81	47	acetanilide
11	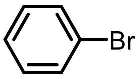	96	47	bromobenzene
12	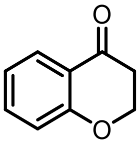	98	46	chromanone
13	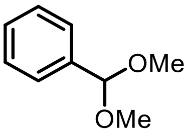	18	46	dimethyl acetal
14	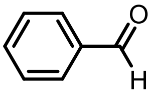	82	47	benzaldehyde
15	none	none	48	control

Average	77	24

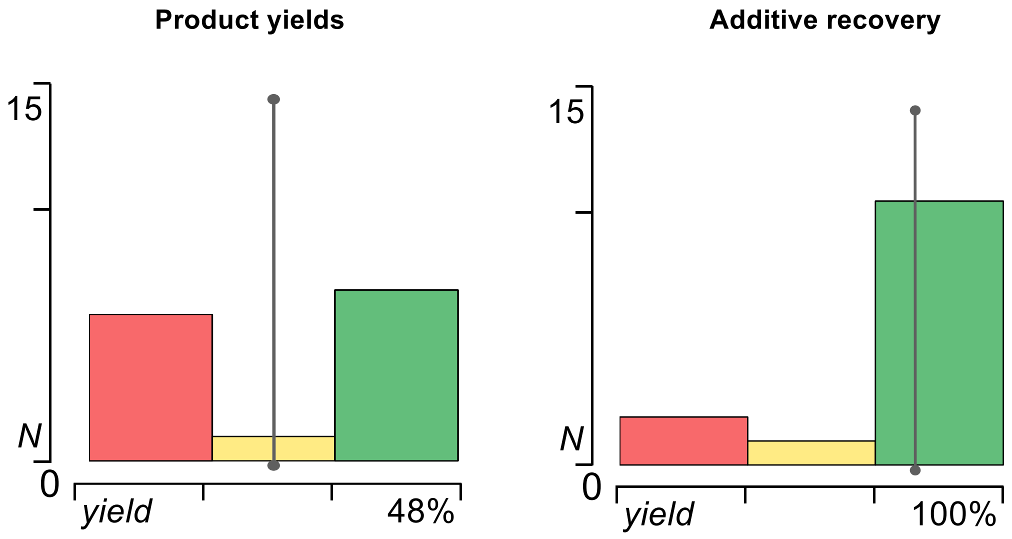

Supplementary Figure 5: Bar graph representation of robustness screening. Left: Impact of additive normed on product yield. Right: Recovery of additive. Grey Line: average.

Condition-Based Sensitivity Screening

The sensitivity screening was conducted following the modified literature protocol.[12] **1b** (1.0 equiv., 1.2 mmol, 263.1 mg) and **Ir-F** (1.0 mol%, 13.5 mg) were added to an oven-dried 50.0 mL Schlenk tube equipped with a Teflon-coated magnetic stir bar. The tube was evacuated and backfilled with argon three times. Under an argon atmosphere, HFIP (10.8 mL) and **2a** (5.0 equiv., 6.0 mmol, 1.10 g) were added sequentially, and the reaction vessel was sealed tightly.

A set of 10.0 mL Schlenk tubes was then evacuated and backfilled with argon three times. Subsequently, 0.9 mL of the stock solution and 0.1 mL of the corresponding additive were introduced under an argon atmosphere to all the tubes. The yield of the product was calculated by ^{1}H NMR analysis using CH_2Br_2 as an internal standard. The deviations from the standard reaction conditions are summarized in **Supplementary Table 3**.

Entry	Parameter	Deviation from standard condition	Yield (%)	Deviation from benchmark
1	None	Standard condition	57	-
2	High c	Without 0.1 mL HFIP	46	-19
3	Low c	+ 0.1 mL HFIP	48	-6
4	H_2O	10 µL H_2O	47	-18
5	Low O_2	Freeze pump thaw	54	-5
6	High O_2	Open air	0	-100
7	Low T	Using cooled photoreactor	51	-10
8	High T	Fan off	57	0
9	Low I	LED distance of 30 cm	40	-30
10	High I	LED distance of 3 cm	43	-25
11	Big Scale	1 mmol scale	41	-28

Supplementary Table 3: Sensitivity screening for the formation of product.

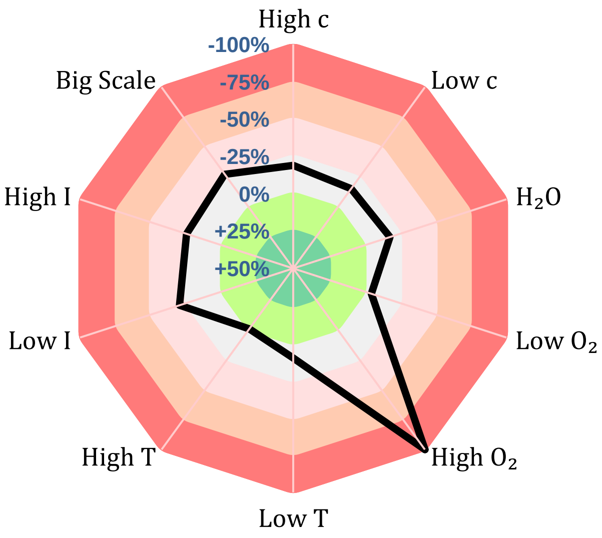

Supplementary Figure 6: Radar diagram representation of sensitivity screen.

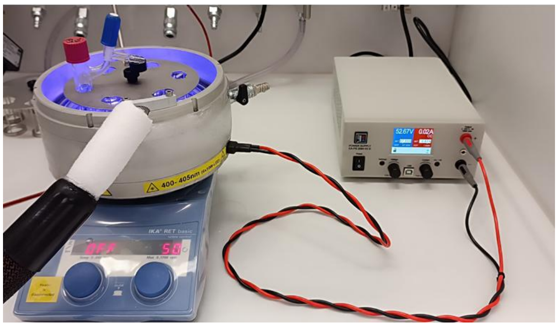

Supplementary Figure 7: Low-temperature reactions in a photoreactor cooled with an external cryostat and cooling liquid.

MECHANISTIC STUDIES

Reaction Development

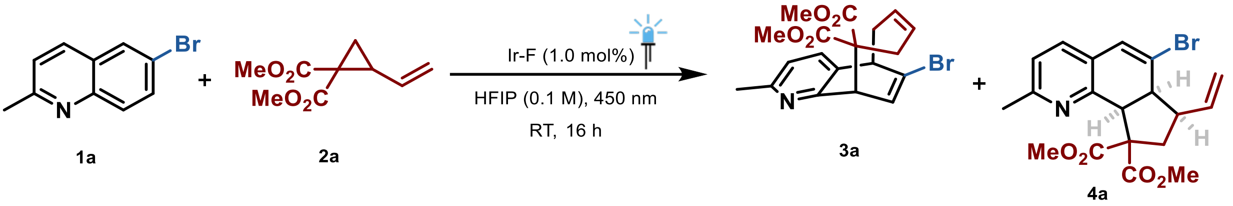

To an oven-dried 10.0 mL Schlenk tube equipped with a Teflon-coated magnetic stir bar was charged **1a** (1.0 equiv., 0.2 mmol, 44.4 mg) and [Ir(dF(CF$_3$)ppy)$_2$(dtbbpy)][PF$_6$] (**Ir-F**) (1.0 mol%, 2.2 mg). The Schlenk tube was evacuated and backfilled with argon three times. Subsequently, under positive argon atmosphere HFIP (2.0 mL, 0.1 M) and **2a** (2.0 equiv., 0.4 mmol, 73.7 mg) were added. Then tube was tightly sealed and irradiated with 30 W blue LEDs (λ_{max} = 450 nm) for 16 hours. After removal of the solvent, CH_2Br_2 was added as an internal standard and ^1H NMR was recorded. The crude mixture was purified by column chromatography on silica gel to yield the desired [5+4] cycloadduct **3a** (19.5 mg, 24%) and unexpected product **4a** (17.1 mg, 21%) as a white solid.

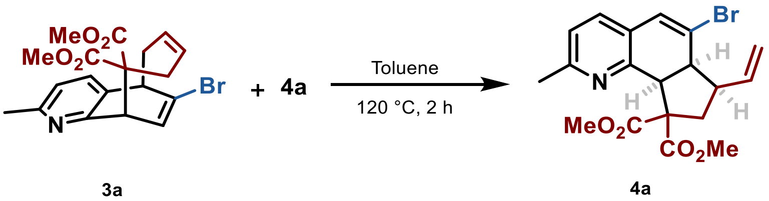

Without further purification, solvent was removed from the crude mixture of **3a** and **4a** as obtained in the previous procedure. Subsequently, toluene (1.0 mL, 0.1 M) was added to the Schlenk tube equipped with a Teflon-coated magnetic stir. The resulting mixture was refluxed for 2 hours at 120 °C. After completion, solvent

was removed under reduce pressure and ^{1}H NMR yield was determined using CH_2Br_2 as an internal standard. The crude product was purified by flash column chromatography (SiO_2; 100:0 to 95:05 pentane/EtOAc) to yield **4a** (24.4 mg, 30%) as a white solid.

dimethyl-13-bromo-2-methyl-5,6,9,11-tetrahydro-10*H*-5,11-ethenocyclonona[*b*]pyridine-10,10-dicarboxylate (3a)

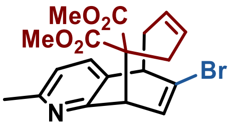

m.p. 126-128 °C.

TLC: R_f = 0.6 (90:10 pentane/EtOAc).

NMR Spectroscopy:

^{1}H NMR (400 MHz, $CDCl_3$): δ_H 7.36 (d, J = 7.9 Hz, 1H), 7.03 (d, J = 7.9 Hz, 1H), 6.81 (d, J = 6.3 Hz, 1H), 5.75 – 5.61 (m, 2H), 4.47 (d, J = 6.3 Hz, 1H), 3.97 (d, J = 10.1 Hz, 1H), 3.82 (s, 3H), 3.82 (s, 3H), 3.10 – 2.93 (m, 1H), 2.61 – 2.49 (m, 1H), 2.44 (s, 3H), 2.29 – 2.16 (m, 1H), 1.56 – 1.43 (m, 1H) ppm.

^{13}C NMR (101 MHz, $CDCl_3$): δ_C 170.3, 169.8, 155.9, 152.4, 135.0, 134.7, 130.2, 129.6, 128.7, 125.3, 122.2, 65.2, 53.0, 52.9, 49.2, 48.9, 33.9, 30.7, 24.3 ppm.

HRMS (ESI$^+$): m/z calc'd for $C_{19}H_{20}BrNO_4Na$ $[M+Na]^+$: 428.04679, found: 428.04690.

X-ray: **see Supplementary Figure 19.**

dimethyl-6-bromo-2-methyl-7-vinyl-6a,7,8,9a-tetrahydro-9*H*-cyclopenta[*h*]quinoline-9,9-dicarboxylate (4a)

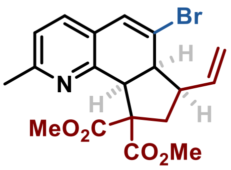

TLC: R_f = 0.7 (90:10 pentane/EtOAc).

NMR Spectroscopy:

^{1}H NMR (400 MHz, $CDCl_3$): δ_H 7.11 (d, J = 7.7 Hz, 1H), 6.90 (d, J = 7.7 Hz, 1H), 6.70 (s, 1H), 5.31 – 5.12 (m, 1H), 5.00 – 4.81 (m, 2H), 4.44 (d, J = 8.8 Hz, 1H), 3.82 (s, 3H), 3.80 (s, 3H), 3.62 (dd, J = 10.6, 8.7 Hz, 1H), 2.96 – 2.83 (m, 1H), 2.62 (ddd, J = 14.1, 8.3, 1.3 Hz, 1H), 2.39 (s, 3H), 2.12 – 2.01 (m, 1H) ppm.

^{13}C NMR (151 MHz, $CDCl_3$): δ_C 172.3, 170.1, 155.9, 150.7, 138.5, 133.0, 128.0, 126.8, 124.8, 121.9, 116.2, 64.7, 53.3, 52.3, 50.9, 50.7, 43.9, 39.0, 24.2 ppm.

HRMS (ESI$^+$): m/z calc'd for $C_{19}H_{20}BrNO_4Na$ [M+Na]$^+$: 428.04679, found: 428.04634.

X-ray: **see Supplementary Figure 22**.

Optimization studies

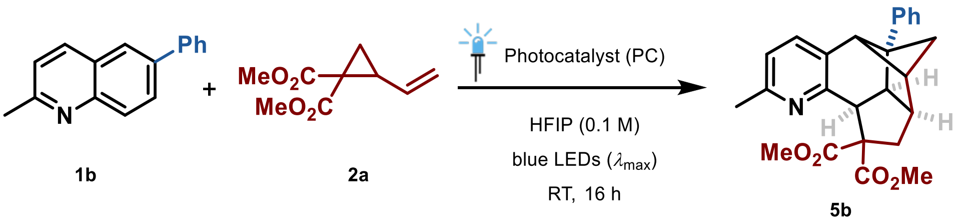

Supplementary Table 4: Variation of photocatalysts, catalyst loading and irradiation wavelength.

Variation in derivatives of TXT

Entry	Photocatalyst (PC)	Wavelength (nm)	mol% (PC)	Yield
1	TXT	405	5	30%
2	TXT-2-Cl	405	5	27%
3	ITX	405	5	22%
4	Xanthone	405	5	Not detected

Variation in mol% of Photocatalyst

Entry	Photocatalyst (PC)	Wavelength (nm)	mol% (PC)	Yield
1	TXT	405	10	44%
2	TXT-2-Cl	405	10	42%
3	ITX	405	10	32%
4	Xanthone	405	10	Not detected

Variation in derivatives of Ir

Entry	Photocatalyst (PC)	Wavelength (nm)	mol% (PC)	Yield
1	*fac*-Ir(dFppy)$_3$	450	2	Not detected
2	[Ir(ppy)$_2$dtbbpy]PF$_6$	450	2	10%
3	[Ir{dF(CF$_3$)(ppy)}$_2$bpy]PF$_6$	450	2	52%
4	*fac*-Ir(ppy)$_3$	450	2	Not detected

Variation in mol% of Ir and wavelength

Entry	Photocatalyst (PC)	Wavelength (nm)	mol% (PC)	Yield
1	Ir-F	425	1	50%
2	Ir-F	450	1	56%
3	Ir-F	450	2	54%
4	Ir-F	450	5	56%

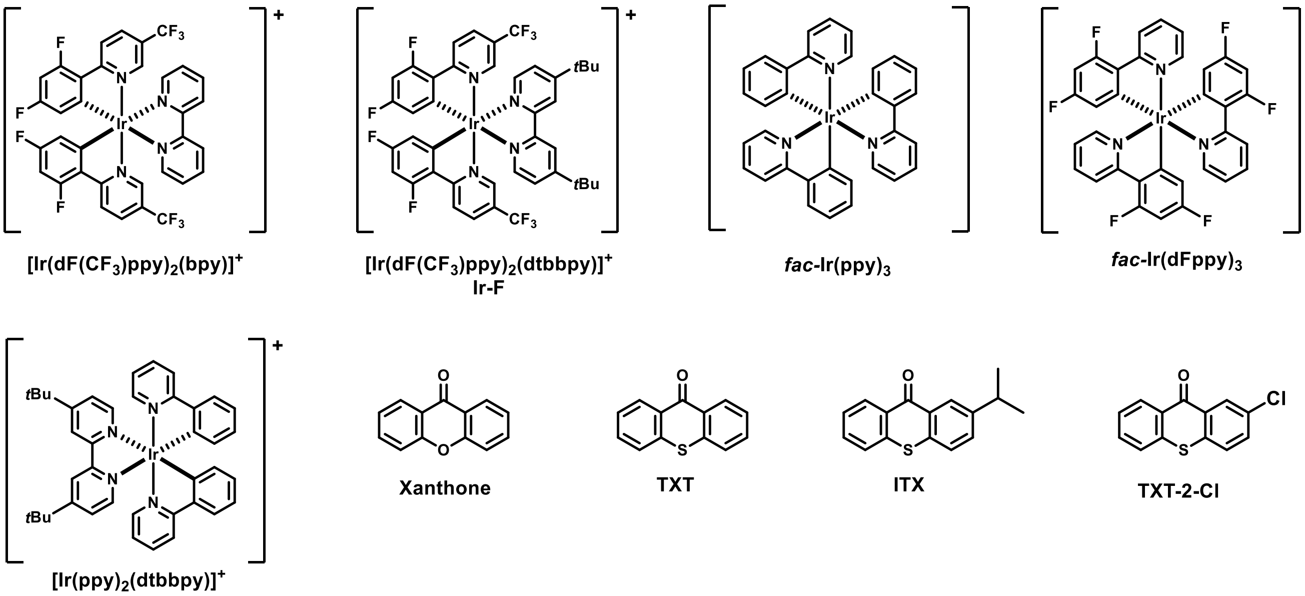

Note: Reactions were performed on 0.1 mmol scale. Yields were determined by crude NMR analysis using CH_2Br_2 as an internal standard.

Intermediate Studies

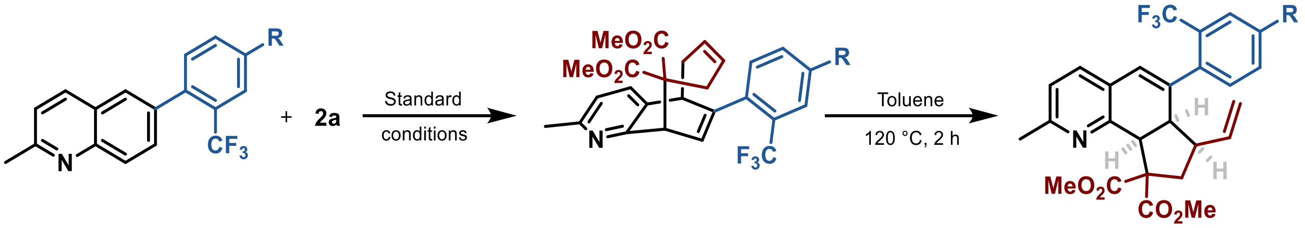

1e, R = H
1f, R = CF_3

3e, R = H, E_T = 68.4 kcal/mol
3f, R = CF_3 E_T = 65.6 kcal/mol

4e, R = H
4f, R = CF_3

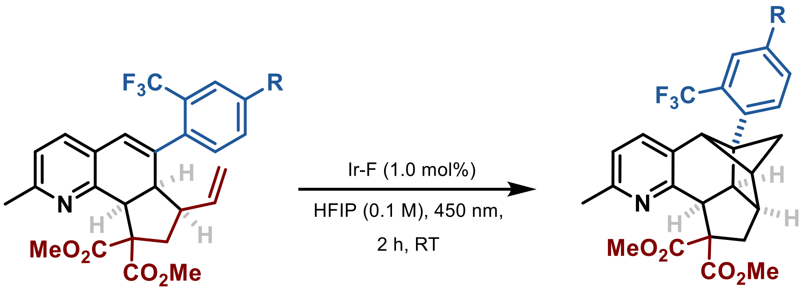

4e, R = H
4f, R = CF_3

5e, R = H
5f, R = CF_3

Following **General Procedure D**, in comparison to all other azaarenes examined, which directly furnished the final pyridine-fused pentacyclic product under visible-light irradiation, [5+4] cycloaddition products **3e** and **3f** (X-ray: **see Supplementary Figure 21**.) formed when quinolines **1e** and **1f** were utilized, respectively. Even after extension of light irradiation time, no further cascade [3,3]-rearrangement/[2+2] cycloaddition was observed. These results were validated by computational studies, where dynamic vertical triplet energies (DvTE) of [5+4] cycloaddition products **3e** (68.4 kcal/mol) and **3f** (65.6 kcal/mol) exceed that of the photocatalyst employed in the reaction, thereby preventing a subsequent photochemical excitation. This can be rationalized by reduced conjugation between the phenyl group and the double bond of olefin, which might be because of twisted conformation caused by steric of the ortho-substituents.

Subsequently, the [5+4] adducts **3e** and **3f** were heated in toluene at 120 °C for 2 h, affording [3,3]-sigmatropic rearrangement products **4e** and **4f**. This result also aligns with the computed barrier for the intermediates **3e** and **3f** (30.0 kcal/mol, 29.2 kcal/mol), considering pericyclic mechanism, making them inaccessible at room temperature. However, as proven by the experimental results, [3,3]-sigmatropic rearrangement product could be achieved by elevated temperature. Upon completion of the thermal step, compounds **4e** and **4f** underwent smooth photochemical [2+2] cycloaddition under blue LEDs irradiation (30 W, λ_{max} = 450 nm) in HFIP for 2 h, delivering **5e** and **5f.** This sequence confirms that, photochemically, only the [3,3]-rearrangement is inaccessible for substrates whose [5+4] cycloadducts possess higher triplet energies than photocatalyst. In contrast, the final [2+2] cycloaddition step remains photochemically viable once the thermal bypass is employed.

dimethyl-2-methyl-13-(2-(trifluoromethyl)phenyl)-5,6,9,11-tetrahydro-10*H*-5,11-ethenocyclonona[*b*]pyridine-10,10-dicarboxylate (3e)

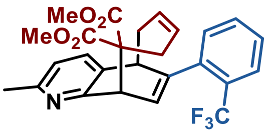

According to **General Procedure D**, the following compound was synthesized using **1e** (1.0 equiv., 0.2 mmol, 57.5 mg), **2a** (5.0 equiv., 1.0 mmol, 184.2 mg). The reaction mixture was irradiated with blue LEDs for 16 hours. After removing the solvent under reduced pressure, ^{1}H NMR was recorded using CH_2Br_2 as an internal standard to yield **3e** (NMR yield, 25%). The isolation was difficult due to other minor impurities.

dimethyl-2-methyl-6-(2-(trifluoromethyl)phenyl)-7-vinyl-6a,7,8,9a-tetrahydro-9*H*-cyclopenta[*h*]quinoline-9,9-dicarboxylate (4e)

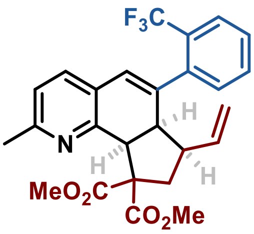

The crude product **3e** was subjected to reflux in toluene at 120 °C for 2 h. After removing the solvent under reduced pressure, pure **4e** was obtained (15.6 mg, 66%) as a white solid without the need for column chromatography.

Note: Yield of **4e** is mentioned with respect to NMR yield of **3e**

NMR Spectroscopy:

^{1}H NMR (599 MHz, $CDCl_3$): δ_H 7.68 (dd, *J* = 7.6, 1.8 Hz, 1H), 7.51 – 7.43 (m, 1H), 7.42 – 7.34 (m, 2H), 7.21 (d, *J* = 7.7 Hz, 1H), 6.98 – 6.87 (m, 1H), 6.36 (s, 1H), 5.30 – 5.15 (m, 1H), 4.84 – 4.82 (m, 1H), 4.81 – 4.80 (m, 1H), 4.50 (d, *J* = 7.6 Hz, 1H), 3.82 (s, 3H), 3.76 (s, 3H), 3.43 (dd, *J* = 9.9, 7.6 Hz, 1H), 2.87 – 2.75 (m, 1H), 2.68 (ddd, *J* = 14.2, 8.7, 1.2 Hz, 1H), 2.45 (s, 3H), 2.14 (dd, *J* = 14.3, 7.6 Hz, 1H) ppm.

^{13}C{^{19}F} NMR (151 MHz, $CDCl_3$): δ_C 172.4, 170.7, 155.9, 152.4, 141.3, 141.1, 137.3, 133.8, 131.4, 130.1, 128.7, 127.5, 126.8, 126.7, 126.6, 124.5, 121.7, 115.2, 64.4, 53.1, 52.3, 50.2, 47.0, 44.4, 39.4, 24.2 ppm.

^{19}F NMR (564 MHz, $CDCl_3$): δ_F -57.38 ppm.

HRMS (ESI$^+$): m/z calc'd for $C_{26}H_{24}F_3NO_4Na$ $[M+Na]^+$: 494.15496, found: 494.15486.

dimethyl-3-methyl-10-(2-(trifluoromethyl)phenyl)-7,8,8a,9,10,10a-hexahydro-5,8,10-(epimethanetriyl)cyclobuta[3,4]cycloocta[1,2-*b*]pyridine-6,6(5*H*)-dicarboxylate (5e)

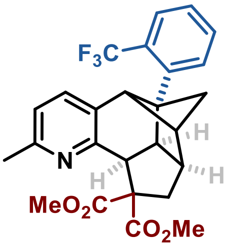

To an oven-dried 10.0 mL Schlenk tube equipped with a Teflon-coated magnetic stir bar was charged **4e** (1.0 equiv., 0.2 mmol, 94.3 mg) and $[Ir(dF(CF_3)ppy)_2(dtbbpy)][PF_6]$ (**Ir-F**) (1.0 mol%, 2.2 mg). The Schlenk tube was evacuated and backfilled with argon three times. Subsequently, under an argon atmosphere, HFIP (2.0 mL, 0.1 M) was added. The tube was then tightly sealed and irradiated with 30 W blue LEDs (λ_{max} = 450 nm) for 2 hours. After removal of the solvent, CH_2Br_2 was added as an internal standard, and ^{1}H NMR was recorded. The

crude product was purified by column chromatography on silica gel to yield the desired product **5e** (82.0 mg, 87%) as a white solid.

TLC: R_f = 0.2 (90:10 pentane/EtOAc).

NMR Spectroscopy:

^{1}H NMR (400 MHz, $CDCl_3$): δ_H 7.60 (dd, *J* = 7.9, 1.4 Hz, 1H), 7.41 (d, *J* = 7.7 Hz, 1H), 7.22 – 7.12 (m, 1H), 7.10 – 7.01 (m, 1H), 6.97 (d, *J* = 7.7 Hz, 1H), 6.54 (d, *J* = 7.9 Hz, 1H), 3.89 (d, *J* = 5.7 Hz, 1H), 3.78 (s, 3H), 3.75 – 3.66 (m, 4H), 3.18 – 3.05 (m, 1H), 2.88 – 2.79 (m, 1H), 2.71 (q, *J* = 8.7 Hz, 1H), 2.45 (s, 3H), 2.25 (ddd, *J* = 14.0, 8.8, 1.4 Hz, 1H), 2.08 – 2.02 (m, 2H), 1.81 (dd, *J* = 14.0, 8.3 Hz, 1H) ppm.

^{13}C{^{19}F} NMR (126 MHz, $CDCl_3$): δ_C 171.7, 169.4, 156.2, 155.9, 140.2, 135.5, 131.5, 130.2, 129.3, 128.3, 127.1, 126.5, 124.7, 121.7, 69.9, 52.9, 52.4, 52.2, 50.9, 50.3, 49.2, 48.9, 47.8, 44.8, 31.4, 24.5 ppm.

^{19}F NMR (376 MHz, CDCl3): δ_F -57.60 ppm.

HRMS (ESI$^+$): m/z calc'd for $C_{26}H_{24}NO_4F_3Na$ $[M+Na]^+$: 494.15496, found: 494.15522.

dimethyl(Z)-13-(2,4-bis(trifluoromethyl)phenyl)-2-methyl-5,6,7,11-tetrahydro-10H-5,11-ethenocyclonona[*b*]pyridine-10,10-dicarboxylate (3f)

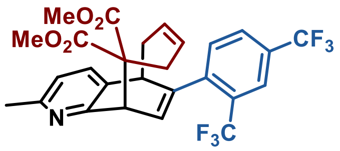

According to **General Procedure D**, the following compound was synthesized using **1f** (1.0 equiv., 0.2 mmol, 71.0 mg), **2a** (5.0 equiv., 1.0 mmol, 184.2 mg). The reaction mixture was irradiated with blue LEDs for 16 hours. Solvent was evaporated and purified by flash column chromatography (SiO_2; 100:0 to 90:10 pentane/EtOAc) to afford **3f** (17.3 mg, 16%) as a white solid.

TLC: R_f = 0.7 (80:20 pentane/EtOAc).

NMR Spectroscopy:

^{1}H NMR (400 MHz, $CDCl_3$): δ_H 7.87 (br. s, 1H), 7.74 (d, *J* = 8.0 Hz, 1H), 7.44 (d, *J* = 7.8 Hz, 1H), 7.36 (d, *J* = 8.1 Hz, 1H), 7.07 (d, *J* = 7.8 Hz, 1H), 6.57 (d, *J* = 6.3 Hz, 1H), 5.88 – 5.73 (m, 1H), 5.56 – 5.43 (m, 1H), 4.66 (d, *J* = 4.6 Hz, 1H), 4.12 (d, *J* = 9.8 Hz, 1H), 3.88 (s, 3H), 3.84 (s, 3H), 2.81 – 2.69 (m, 1H), 2.60 (dd, *J* = 14.2, 6.2 Hz, 1H), 2.48 (s, 3H), 2.36 (dd, *J* = 15.0, 6.2 Hz, 1H), 1.66 – 1.58 (m, 1H) ppm.

^{13}C{^{19}F} NMR (126 MHz, $CDCl_3$): δ_C 170.7, 170.1, 155.6, 153.5, 144.7, 138.4, 135.2, 135.2, 131.9, 130.7, 130.6, 130.0, 129.9, 129.3, 128.5, 123.9, 123.6, 123.5, 122.2, 65.0, 52.9, 52.7, 47.7, 44.9, 34.6, 30.8, 24.3 ppm.

^{19}F NMR (376 MHz, CDCl3): δ_F -58.20, -62.87 ppm.

HRMS (ESI$^+$): m/z calc'd for $C_{27}H_{23}F_6NO_4Na$ $[M+Na]^+$: 562.14235, found: 562.14223.

Note: Some trace amounts of unknown impurities could not be removed from the isolated product even after multiple rounds of purifications.

X-ray: **see Supplementary Figure 21**.

dimethyl-6-(2,4-bis(trifluoromethyl)phenyl)-2-methyl-7-vinyl-6a,7,8,9a-tetrahydro-9H-wcyclopenta[*h*]quinoline-9,9-dicarboxylate (4f)

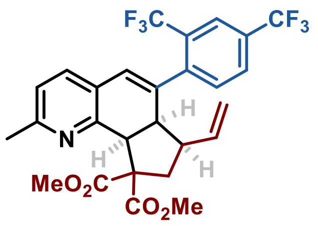

The isolated product **3f** (0.1 mmol, 53.9 mg) was subjected to reflux in toluene at 120 °C for 2 h. After removing the solvent under reduced pressure, pure **4f** was obtained (43.7 mg, 81%) as a white solid without the need of column chromatography. **m.p.** 142-145 °C.

^{1}H NMR (400 MHz, $CDCl_3$): δ_H 7.97 (s, 1H), 7.76 (dd, *J* = 8.1, 1.9 Hz, 1H), 7.59 (d, *J* = 8.1 Hz, 1H), 7.40 – 7.19 (m, 1H), 7.00 (d, *J* = 7.7 Hz, 1H), 6.42 (s, 1H), 5.47 – 5.13 (m, 1H), 4.89 (s, 1H), 4.87 – 4.84 (m, 1H), 4.52 (d, *J* = 7.6 Hz, 1H), 3.85 (s, 3H), 3.79 (s, 3H), 3.47 (dd, *J* = 9.8, 7.7 Hz, 1H), 2.94 – 2.77 (m, 1H), 2.71 (ddd, *J* = 14.2, 8.7, 1.2 Hz, 1H), 2.49 (s, 3H), 2.17 (dd, *J* = 14.2, 7.6 Hz, 1H) ppm.

^{13}C{^{19}F} NMR (126 MHz, $CDCl_3$): δ_C 172.2, 170.4, 156.4, 152.2, 144.8, 141.1, 135.7, 133.9, 131.0, 130.0, 129.6, 128.1, 127.5, 126.1, 123.7, 123.5, 123.4, 121.7, 115.4, 64.2, 53.0, 52.2, 49.9, 46.6, 44.3, 39.3, 24.1 ppm.

^{19}F NMR (376 MHz, CDCl3): δ_F -57.61, -62.75.ppm

HRMS (ESI$^+$): m/z calc'd for $C_{27}H_{23}F_6NO_4Na$ $[M+Na]^+$: 562.14235, found: 562.14234

dimethyl-10-(2,4-bis(trifluoromethyl)phenyl)-3-methyl-7,8,8a,9,10,10a-hexahydro-5,8,10-(epimethanetriyl)cyclobuta[3,4]cycloocta[1,2-*b*]pyridine-6,6(5*H*)-dicarboxylate (5f)

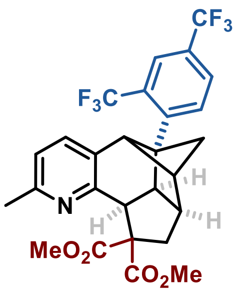

To an oven-dried 10.0 mL Schlenk tube equipped with a Teflon-coated magnetic stir bar was charged **4f** (1.0 equiv., 0.1 mmol, 53.9 mg) and $[Ir(dF(CF_3)ppy)_2(dtbbpy)][PF_6]$ (**Ir-F**) (1.0 mol%, 1.1 mg). The Schlenk tube

was evacuated and backfilled with argon three times. Subsequently, under positive argon atmosphere HFIP (1.0 mL, 0.1 M) and was added. The tube was tightly sealed and irradiated with 30 W blue LEDs (λ_{max} = 450 nm) for 2 hours. After removal of the solvent, CH_2Br_2 was added as an internal standard and ^{1}H NMR was recorded. The crude product was purified by flash column chromatography (SiO_2; 100:0 to 90:10 pentane/EtOAc) to afford **5f** (42.6 mg, 79%) as a white solid.

TLC: R_f = 0.3 (80:20 pentane/EtOAc).

NMR Spectroscopy:

^{1}H NMR (400 MHz, $CDCl_3$): δ_H 7.94 – 7.73 (m, 1H), 7.42 (d, J = 7.7 Hz, 1H), 7.37 – 7.28 (m, 1H), 6.99 (d, J = 7.7 Hz, 1H), 6.68 (d, J = 8.3 Hz, 1H), 3.88 (d, J = 5.7 Hz, 1H), 3.78 (s, 3H), 3.76 – 3.70 (m, 1H), 3.69 (s, 3H), 3.18 – 3.14 (m, 1H), 2.90 – 2.85 (m, 1H), 2.74 (q, J = 8.7 Hz, 1H), 2.46 (s, 3H), 2.25 (ddd, J = 14.1, 8.9, 1.4 Hz, 1H), 2.12 – 2.04 (m, 2H), 1.82 (dd, J = 14.1, 8.3 Hz, 1H) ppm.

^{13}C{^{19}F} NMR (126 MHz, $CDCl_3$): δ_C 171.6, 169.2, 156.7, 155.7, 144.7, 135.6, 130.0, 129.7, 129.2, 129.0, 128.3, 124.3, 123.9, 123.4, 121.9, 69.9, 53.0, 52.3, 52.2, 51.1, 50.6, 49.2, 48.8, 47.9, 44.8, 31.3, 24.5 ppm.

^{19}F NMR (376 MHz, CDCl3): δ_F -58.04, -63.10 ppm

HRMS (ESI$^+$): m/z calc'd for $C_{27}H_{23}F_6NO_4Na$ $[M+Na]^+$: 562.14235, found: 562.14180.

***Ortho*-substituent variation: 2-methyl-6-(*o*-tolyl)quinoline**

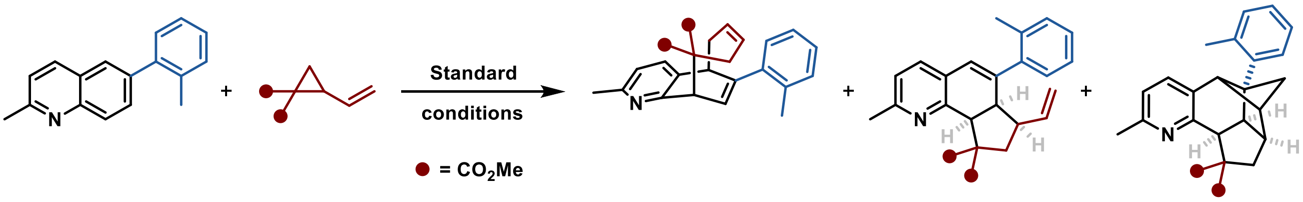

1as **2a** **3as**, 16% DvTE = 63.9 kcal/mol **4as**, 9% **5as**, 8%

According to **General Procedure D**, to an oven-dried 10.0 mL Schlenk tube equipped with a Teflon-coated magnetic stir bar was charged **1as** (1.0 equiv., 0.2 mmol, 46.7 mg), **2a** (5.0 equiv., 1.0 mmol, 184.2 mg). The Schlenk tube was evacuated and backfilled with argon three times. Subsequently, under positive argon atmosphere HFIP (2.0 mL, 0.1 M) and was added. The tube was tightly sealed and irradiated with 30 W blue LEDs (λ_{max} = 450 nm) for 48 hours. After removal of the solvent, CH_2Br_2 was added as an internal standard and ^{1}H NMR was recorded.

The crude product analysis reveals that mixture of products was obtained [5+4] cycloadduct (16%), [3,3]-rearranged product (9%) and [2+2] cascade product (8%). Moreover, dynamic vertical triplet energy calculations of [5+4] cycloadduct **3as** has been found to be higher (63.9 kcal/mol, **Supplementary Figure 18**) than that of Ir-F photocatalyst (61.8 kcal/mol), this endergonic process leading to slower rearrangement.

^1H NMR (400 MHz, $CDCl_3$)

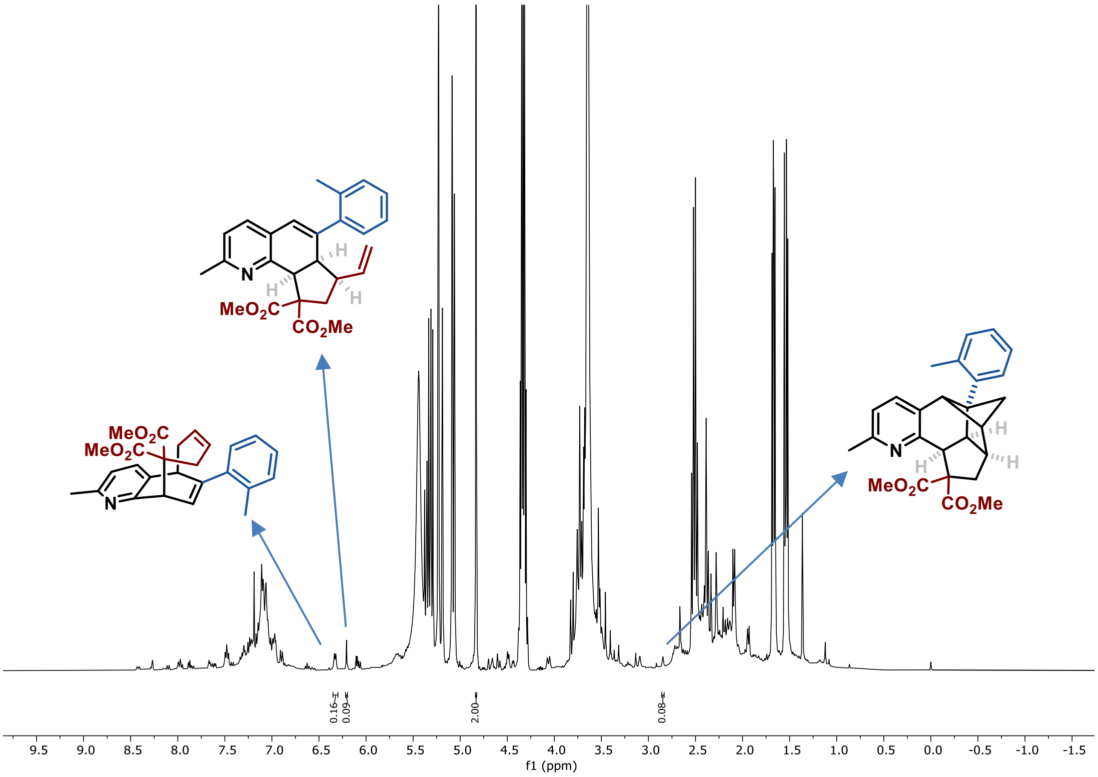

Note: The compounds were assigned in crude NMR by comparison with the related structures. The reaction was performed on 0.2 mmol using the standard reaction conditions (Reaction time: 48 h). ^1H NMR yield was calculated using CH_2Br_2 as an internal standard.

Synthesis of [5+4] cycloadduct (3b)

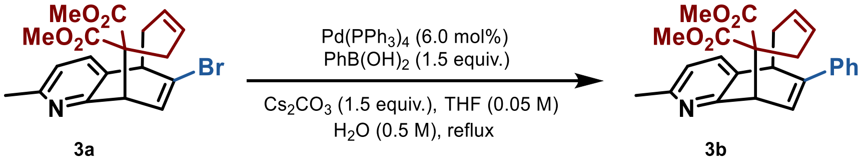

Following the modified literature procedure,[13] to an oven-dried 10.0 mL Schlenk tube equipped with a Teflon-coated magnetic stir bar **3a** (1.0 equiv., 0.1 mmol, 40.6 mg), phenylboronic acid (1.5 equiv., 0.15 mmol, 18.3 mg), Cs_2CO_3 (1.5 equiv., 0.15 mmol, 48.9 mg) and $Pd(PPh_3)_4$ (6.0 mol%, 6.9 mg) were added. The tube was evacuated and backfilled with argon three times. Under argon, THF (2.0 mL, 0.05 M) and H_2O (0.5 mL, 0.2 M) were added in the tube. The mixture was stirred at 65 °C for 2 hours, after which it was quenched with saturated aqueous NH_4Cl solution. The phases were separated and the aqueous layer was extracted with ethyl acetate, dried over anhydrous $MgSO_4$, and the solvent was removed under reduced pressure. Purified by flash column chromatography (SiO_2; 100:0 to 90:10 pentane/EtOAc) to afford the product **3b** (28.2 mg, 70%). **m.p.** 136-140 °C.

dimethyl-2-methyl-13-phenyl-5,6,9,11-tetrahydro-10*H*-5,11-ethenocyclonona[*b*]pyridine-10,10-dicarboxylate (3b)

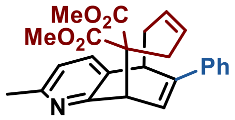

TLC: R_f = 0.2 (90:10 pentane/EtOAc).

NMR Spectroscopy:

^{1}H NMR (400 MHz, $CDCl_3$): δ_H 7.48 (d, *J* = 7.7 Hz, 1H), 7.42 – 7.37 (m, 2H), 7.36 – 7.30 (m, 2H), 7.29 – 7.22 (m, 1H), 7.05 (d, *J* = 7.8 Hz, 1H), 6.94 – 6.87 (m, 1H), 5.70 – 5.56 (m, 1H), 5.40 – 5.25 (m, 1H), 4.66 (d, *J* = 6.3 Hz, 1H), 4.30 (d, *J* = 10.3 Hz, 1H), 3.87 (s, 3H), 3.83 (s, 3H), 2.97 (ddd, *J* = 14.4, 10.5, 9.0 Hz, 1H), 2.62 – 2.51 (m, 1H), 2.47 (s, 3H), 2.35 (dd, *J* = 14.5, 7.0 Hz, 1H), 1.68 (dd, *J* = 14.1, 11.4 Hz, 1H) ppm.

^{13}C NMR (101 MHz, $CDCl_3$): δ_C 170.9, 170.2, 155.3, 153.8, 140.7, 140.0, 135.8, 135.4, 129.8, 129.4, 128.6, 127.4, 126.0, 125.9, 122.0, 65.2, 52.9, 52.7, 47.8, 42.5, 35.4, 30.6, 24.3 ppm.

HRMS (ESI$^+$): m/z calc'd for $C_{25}H_{25}NO_4Na$ $[M+Na]^+$: 426.16758, found: 426.16746.

X-ray: **see Supplementary Figure 20**.

Synthesis of the [3,3]-rearranged product (4b)

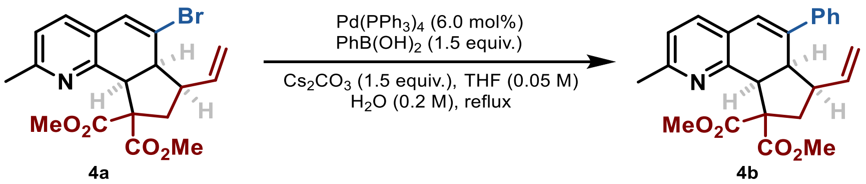

Following the modified literature procedure,[13] to an oven-dried 10.0 mL Schlenk tube equipped with a Teflon-coated magnetic stir bar **4a** (1.0 equiv., 0.2 mmol, 81.2 mg), phenylboronic acid (1.5 equiv., 0.3 mmol, 36.6 mg), Cs_2CO_3 (1.5 equiv., 0.3 mmol, 97.7 mg), and $Pd(PPh_3)_4$ (6.0 mol%, 13.9 mg) were added. The tube was evacuated and backfilled with argon three times. Under an argon atmosphere, THF (4.0 mL, 0.05 M) and H_2O (1.0 mL, 0.2 M) were added in the tube. The mixture was stirred at 65 °C for 2 hours, after which it was allowed to cool to room temperature and quenched with saturated aqueous NH_4Cl solution. The phases were separated and the aqueous layer was extracted with ethyl acetate, dried over anhydrous $MgSO_4$, and the solvent was removed under reduced pressure. Purified by flash column chromatography (SiO_2; 100:0 to 85:15 pentane/EtOAc) to affoard the product **4b** (60.5 mg, 75%).

TLC: R_f = 0.2 (90:10 pentane/EtOAc).

NMR Spectroscopy:

^{1}H NMR (400 MHz, $CDCl_3$): δ_H 7.55 – 7.43 (m, 2H), 7.41 – 7.30 (m, 2H), 7.31 – 7.20 (m, 2H), 6.94 (d, *J* = 7.7

Hz, 1H), 6.82 (s, 1H), 5.28 – 5.04 (m, 1H), 4.73 – 4.50 (m, 2H), 4.39 (d, J = 8.2 Hz, 1H), 3.86 (s, 3H), 3.83 (s, 3H), 3.82 – 3.76 (m, 1H), 2.97 (p, J = 9.1 Hz, 1H), 2.71 (dd, J = 14.0, 8.5 Hz, 1H), 2.44 (s, 3H), 2.15 (dd, J = 14.1, 8.6 Hz, 1H) ppm.

^{13}C NMR (101 MHz, $CDCl_3$): δ_C 172.7, 170.6, 155.4, 152.0, 139.8, 139.6, 137.7, 133.9, 128.5, 127.8, 127.4, 125.7, 122.4, 121.8, 115.0, 64.9, 53.2, 52.3, 49.9, 44.9, 44.3, 39.2, 24.2 ppm.

HRMS (ESI$^+$): m/z calc'd for $C_{25}H_{25}NO_4Na$ [M+Na]$^+$: 426.16758, found: 426.1675.

UV-vis Absorption Spectroscopy

UV-vis absorption spectra were measured on a Jasco V-730 spectrophotometer, equipped with a temperature control unit at 25 °C. The samples were recorded in Starna® fluorescence quartz cuvettes (type: 29-F, chamber volume = 1.40 mL, H × W × D = 48 mm × 12.5 mm × 12.5 mm, path length = 10 mm). The instrument parameters are as follows: response time = 0.06 sec, data interval = 1.0 nm, scan speed = 1000 nm/min.

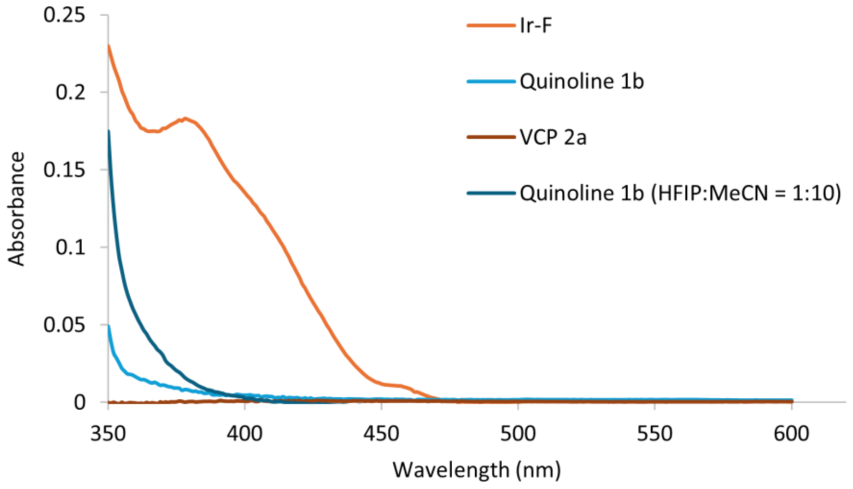

Supplementary Figure 8: UV-vis absorption spectra of **Ir−F** (0.1 mM), 2-methyl-6-phenylquinoline **1b** (0.1 mM), VCP **2a** (0.1 mM) in CH_3CN, and **1b** (0.1 mM) in HFIP:CH_3CN (1:10).

Stern-Volmer Luminescence Quenching Studies

Stern-Volmer luminescence quenching studies were performed to identify the relative quencher of the excited state photocatalyst in the reaction. For this purpose, the fluorescence of the photocatalyst [Ir(dF(CF_3)ppy)$_2$dtbbpy][PF_6] (**Ir-F**) was recorded in the presence of each reaction partner (quinoline **1b** and VCP **2a**), which can act as a quencher at varying concentrations. Measurements were carried out using a Jasco FP-8300 spectrofluorometer using Starna® fluorescence quartz cuvettes (type: 29-F, chamber volume = 1.400 mL, H × W × D = 48 mm × 12.5 mm × 12.5 mm, path length = 10 mm). All samples for the quenching studies were prepared inside an argon-filled glovebox using dry, degassed CH_3CN and HFIP. The following stock solutions were prepared prior to the measurements: photocatalyst (100 µM), quinoline (**1b**) and VCP (**2a**) (0.025

M). Quenching experiments were measured using 100 µL of the [Ir(dF(CF_3)ppy)$_2$dtbbpy][PF_6] (**Ir-F**) stock solution and varying concentrations of the quenchers under an argon atmosphere. The following parameters were used: data interval = 0.5 nm, scan speed = 500 nm/min, excitation wavelength λ_{Ex} = 405 nm, measured luminescence wavelength λ = 472 nm.

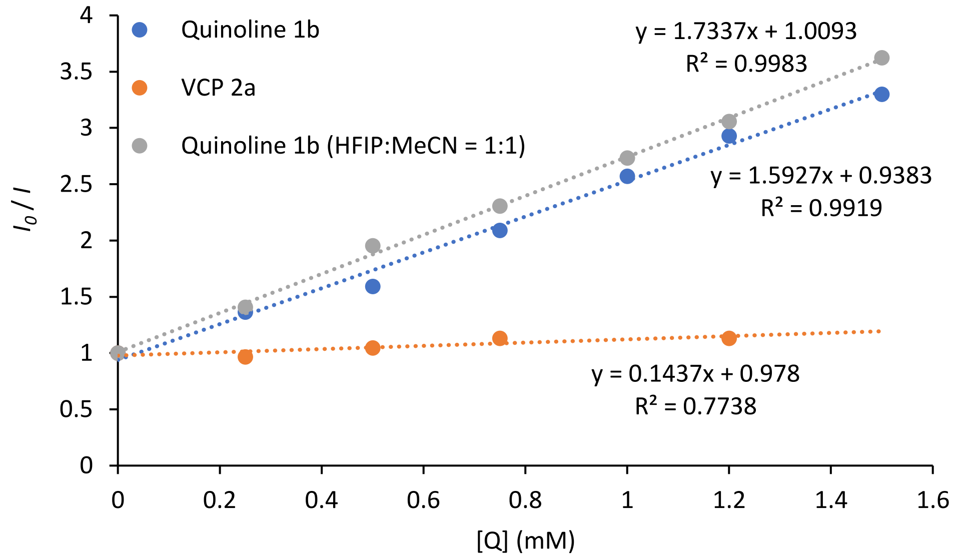

Supplementary Figure 9: Stern-Volmer luminescence quenching measurements using **Ir-F** as a photocatalyst.

Cyclic Voltammetry Analysis

The cyclic voltammograms (CVs) were measured with a STAT-I 400s electrochemical set-up by Metrohm DropSens. Three-electrode cell set up consists of Reference electrode: Ag/AgCl (2 M solution of LiCl in EtOH), working electrode: 3 mm glassy carbon disc electrode, and counter electrode: platinum wire. The following parameters were set prior to measurement: scan rate = 0.1 V/s, E_{step} = 0.002 V. A 50 mM solution of *n*-$Bu_4N(PF_6)$ in MeCN was used as electrolyte, which was degassed with argon inside the set-up. Before measuring the CV, all electrodes and the set-up were cleaned carefully. First, a blank CV and the CV of ferrocene were measured. Subsequently, 5 mM solution of each substrate was prepared in 20.0 mL electrolyte and the CV was recorded. The half peak potential of Fc^+/Fc was found to be + 0.52 V *vs* Ag/AgCl (2 M solution of LiCl in EtOH). The redox potential of both the substrates was referenced with respect to Fc^+/Fc by subtracting +0.52 V. The redox potential of **1b** was converted to the saturated calomel electrode (SCE) scale.[14]

$$E_{1/2}\ (\mathbf{1b}) = +1.29\ \text{V (vs Fc}^+\text{/ Fc) } +0.380\ \text{V} = +1.67\ \text{V (vs SCE)}$$

CV analysis showed that quinoline undergoes irreversible oxidation with a half peak oxidation potential of +1.29 V *vs* Fc^+/ Fc (+1.67 V *vs* SCE) for **1b**. Although, no redox activity was observed for **2a** within the measured potential window.

Considering the redox potential window of the used photocatalyst, **Ir–F** ($E_{1/2}$ $PC^{\cdot+}$/PC* = −0.89 V *vs* SCE and

$E_{1/2}$ PC*/PC$^{·-}$ = +1.21 V *vs* SCE)[15], oxidation or reduction of the quinoline **1b** by the excited photocatalyst is unfeasible.

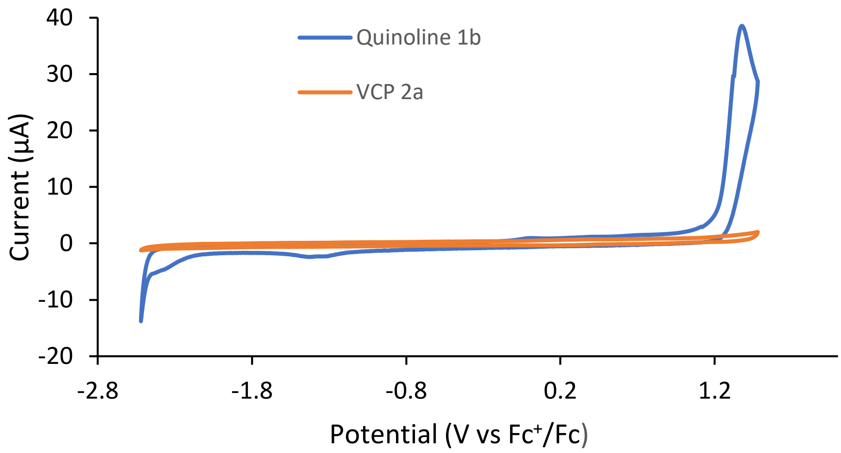

Supplementary Figure 10: Cyclic voltammetry of quinoline **1b** (5 mM), VCP **2a** (5 mM) in acetonitrile (50 mM *n*-$Bu_4N(PF_6)$.

Quantum Yield Analysis

Determination of the photon flux

First, the photon flux of blue LED (3 W, λ_{max} = 420 nm) was determined by ferrioxalate actinometry following a reported literature procedure by Yoon and coworkers.[16]

Both the solutions were prepared in volumetric flask and stored in the dark to prevent any unnecessary irradiation. The following procedure was performed in darkened lab.

Solution 1: $K_3[Fe(C_2O_4)_3]$ (737 mg, 1.50 mmol) dissolved in 0.05 M H_2SO_4 (10 mL).

Solution 2: 1,10-phenanthroline monohydrate (20 mg, 0.10 mmol) and anhydrous NaOAc (4.50 g) dissolved in 0.5 M H_2SO_4 (20.0 mL).

To determine the photon flux, the reduction of $[Fe(C_2O_4)_3]^{3-}$ to $[Fe(C_2O_4)_2]^{2-}$ is measured by irradiation over time. Six 10 mL Schlenk tubes equipped with stir bars, and each containing 1 mL of *solution 1* were prepared. Three of them were kept at a distance of 5 cm to 420 nm lamp and irradiated for 60 s, the other three were non-irradiated control samples. Subsequently, 175 µL of *solution 2* was added to all six samples (three irradiated, three non-irradiated) and stirred for 1 h in the complete darkness.

The absorbance of all samples was then recorded at 510 nm. The average absorption of three non-irradiated and three irradiated samples was used to calculate the generated amount of iron (II) ions according to Lambert–Beer law (**Supplementary Equation 1**), where V is sample volume, ΔA is the absorption difference between the irradiated and non-irradiated samples, l is the cuvette path length and ε is the molar absorptivity of the

ferrioxalate actinometer at 510 nm.

$$n\,(Fe^{2+}) = \frac{V \cdot \Delta A(510\text{ nm})}{l \cdot \varepsilon} = \frac{1.175 \cdot 10^{-3}\text{L} \cdot 2.481}{1\text{ cm} \cdot 11100\frac{\text{L}}{\text{mol} \cdot \text{cm}}} \tag{1}$$

$$= 2.626 \cdot 10^{-7}\text{mol}$$

The fraction of light absorbed s λ = 420 nm by the actinometer was calculated using **Supplementary equation 2**, where $A_{420\ nm}$ is the absorbance of *solution 1* at λ = 420 nm.

$$f = 1 - 10^{-A_{420\text{ nm}}} = 0.999 \tag{2}$$

Here, the absorbance $A_{420\text{ nm}}$ of **Solution 1** was > 3, which corresponds to the solution absorbing more than 99.9% of the photons (*f* > 0.999).

Next the photon flux can then be calculated using equation (3), where ϕ_F is the quantum yield of the ferrioxalate actinometer (1.13 at λ = 392 nm), and t is the irradiation time (60 s).

$$\phi_\text{q} = \frac{n(Fe^{2+})}{\phi_\text{F} \cdot t \cdot f} = \frac{2.626 \cdot 10^{-7}\text{mol}}{1.13 \cdot 60\text{ s} \cdot 0.999} = 3.877 \cdot 10^{-9}\text{ mol s}^{-1} \tag{3}$$

Determination of the reaction quantum yield

The reaction quantum yield was calculated using **Supplementary Equation 4**, where ϕ_q represents the photon flux, t is the irradiation time, and f_R is the fraction of light absorbed by the reaction. The value of f_R was determined by measuring the absorbance of a non-irradiated control reaction using **Supplementary Equation 3**. Reactions were performed according to **General Procedure D** on a 0.1 mmol scale and the reaction mixture was stirred under irradiation in the calibrated set-up (3 W, λ = 420 nm). The solvent was removed under reduced pressure and yields were determined by crude ^{1}H NMR analysis using CH_2Br_2 as an internal standard.

$$f_\text{R} = 1 - 10^{-A_{420\text{ nm}}} = 1 - 10^{-2.33} = 0.995$$

$$\phi = \frac{n_\text{product}}{\phi_\text{q} \cdot t \cdot f_\text{R}} = \frac{6 \cdot 10^{-6}\text{mol}}{3.877 \cdot 10^{-9}\frac{\text{mol}}{\text{s}} \cdot 10800\text{ s} \cdot 0.995} = 0.144 \tag{4}$$

$$\phi = \frac{n_\text{product}}{\phi_\text{q} \cdot t \cdot f_\text{R}} = \frac{9 \cdot 10^{-6}\text{mol}}{3.877 \cdot 10^{-9}\frac{\text{mol}}{\text{s}} \cdot 27000\text{ s} \cdot 0.995} = 0.086$$

	Time (s)	Yield	Quantum yield
1	10800	6%	ϕ = 0.14
2	27000	9%	ϕ = 0.09
		**Average**	ϕ = 0.11

Average quantum yield of the reaction was determined to be ϕ = 0.11, which indicates the EnT-catalyzed pathway and avoiding the involvement of radical chain mechanism.

Radical Trapping Studies

TEMPO trapping study

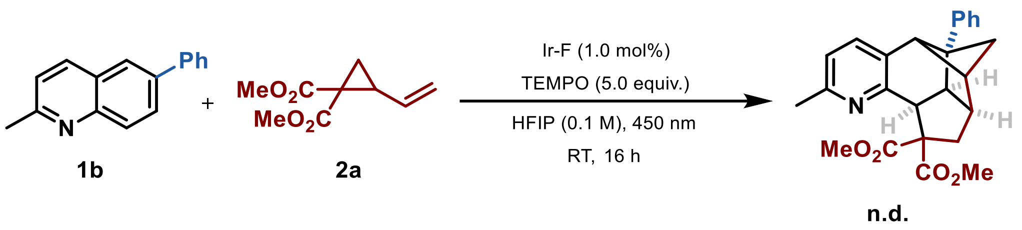

To an oven-dried 10.0 mL Schlenk tube equipped with a Teflon-coated magnetic stir bar was added [Ir{dF(CF$_3$)ppy}$_2$(dtbbpy)]PF$_6$ (**Ir-F**) (1.0 mol%, 1.1 mg), **1b** (1.0 equiv., 0.1 mmol, 21.9 mg), and (2,2,6,6-tetramethylpiperidin-1-yl)oxyl (**TEMPO**) (5.0 equiv., 0.5 mmol, 78.2 mg). The Schlenk tube was evacuated and backfilled with argon three times. Under an argon atmosphere, HFIP (1.0 mL) was added to the Schlenk tube followed by addition of **2a** (5.0 equiv., 0.5 mmol, 92.0 mg) and the tube was sealed tightly. The reaction mixture was irradiated with 30 W blue LEDs (λ_{max} = 450 nm) for 16 hours. Then, the solvent was removed under reduced pressure. The product formation was not observed by crude ^{1}H NMR analysis. HRMS analysis revealed that no TEMPO-trapping adduct of the proposed reaction-intermediates could be detected.

BHT trapping study

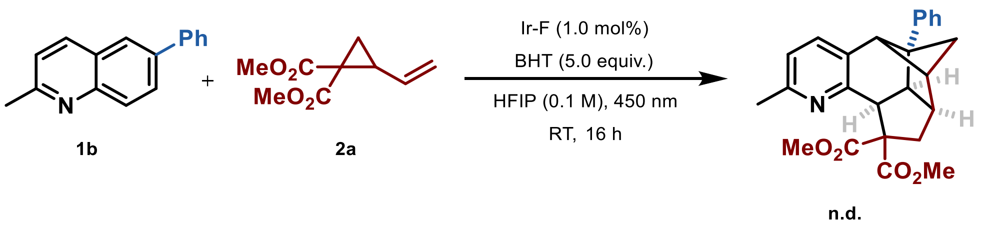

To an oven-dried 10.0 mL Schlenk tube equipped with a Teflon-coated magnetic stir bar was added [Ir{dF(CF$_3$)ppy}$_2$(dtbbpy)]PF$_6$ (**Ir-F**) (1.0 mol%, 1.1 mg), **1b** (1.0 equiv., 0.1 mmol, 21.9 mg), and 2,6-di-*tert*-butyl-4-methylphenol (**BHT**) (5.0 equiv., 0.50 mmol, 110.0 mg). The Schlenk tube was evacuated and backfilled with argon three times. Under argon, HFIP (1.0 mL) was added to the Schlenk tube followed by

addition of **2a** (5.0 equiv., 0.5 mmol, 92.0 mg) and the tube was sealed tightly. The reaction mixture was irradiated with 30 W blue LEDs (λ_{max} = 450 nm) for 16 hours. Then, the solvent was removed under reduced pressure. The product formation was not observed by crude ^{1}H NMR analysis. HRMS analysis revealed that no BHT-trapping adduct with quinoline **1b** could be detected. However, BHT-adduct with VCP was seen in HRMS measurement.

HRMS (ESI$^+$): m/z calc'd for BHT-adduct with VCP **2a** $C_{24}H_{36}O_5Na$ [M+Na]$^+$: 427.24550, found: 427.24436.

PRODUCT DIVERSIFICATION

Reduction of Diester Group (6a)

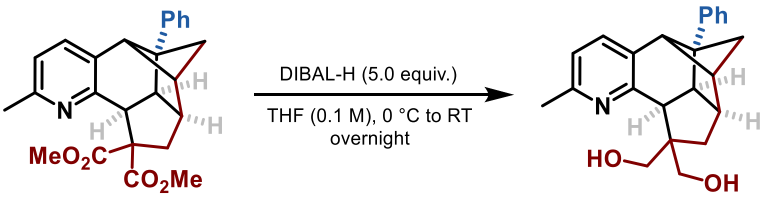

5b **6a**

To an oven-dried 10.0 mL Schlenk tube was added compound **5b** (1.0 equiv., 0.2 mmol, 80.7 mg). The tube was evacuated and backfilled with argon three times. Then, under an argon atmosphere, THF (2.0 mL) was added followed by addition of DIBAL-H (1.2 M in toluene, 5.0 equiv., 0.8 mL) was added at 0 °C. The tube was closed tightly and the reaction was carried out at room temperature overnight. After completion, H_2O was added to the reaction mixture. Then the reaction mixture was filtered over celite and washed with EtOAc. The phases were separated, the aqueous layer was extracted with ethyl acetate and dried over $MgSO_4$. Purified by flash column chromatography (SiO_2; 100:0 to 30:70 pentane/EtOAc) to afford **6a** (57.7 mg, 83%) as a white solid. **m.p.** 62-64 °C.

TLC: R_f = 0.2 (70:30 pentane/EtOAc).

3-methyl-10-phenyl-5,6,7,8,8a,9,10,10a-octahydro-5,8,10-(epimethanetriyl)cyclobuta[3,4]cycloocta[1,2-*b*]pyridine-6,6-diyl)dimethanol (6a)

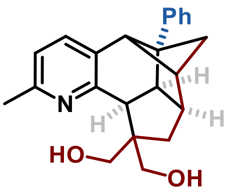

NMR Spectroscopy:

^{1}**H NMR** (400 MHz, $CDCl_3$): δ_H 7.36 (d, *J* = 7.7 Hz, 1H), 7.20 – 7.13 (m, 2H), 7.12 – 7.00 (m, 3H), 6.90 (d, *J* = 7.7 Hz, 1H), 5.98 (br. s, 1H), 3.95 (d, *J* = 6.1 Hz, 2H), 3.91 (d, *J* = 6.1 Hz, 1H), 3.82 (d, *J* = 8.1 Hz, 1H), 3.53 – 3.35 (m, 1H), 3.10 – 2.97 (m, 1H), 2.92 – 2.82 (m, 1H), 2.77 (q, *J* = 8.6 Hz, 1H), 2.71 (d, *J* = 12.1 Hz, 1H),

2.45 (s, 3H), 2.08 (dd, *J* = 6.8, 3.0 Hz, 1H), 1.73 (d, *J* = 6.8 Hz, 1H), 1.28 – 1.17 (m, 1H), 0.73 (dd, *J* = 13.4, 7.3 Hz, 1H) ppm.

^{13}C NMR (151 MHz, $CDCl_3$): δ_C 157.5, 155.6, 140.9, 136.8, 130.1, 128.3, 126.9, 126.5, 120.9, 70.4, 69.9, 59.5, 54.0, 51.2, 49.4, 48.8, 48.1, 46.2, 44.6, 30.3, 24.1 ppm.

HRMS (ESI^+): m/z calc'd for $C_{23}H_{25}NO_2Na$ $[M+Na]^+$: 370.17775, found: 370.17772.

Synthesis of Barbituric Acid Derivative (6b)

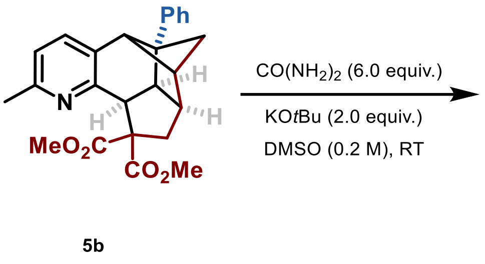

Following the modified literature procedure,[17] to an oven-dried 10.0 mL Schlenk tube compound **5b** (1.0 equiv., 0.2 mmol, 80.7 mg), $CO(NH_2)_2$ (6.0 equiv., 1.2 mmol, 72.1 mg) and KO*t*Bu (2.0 equiv., 0.4 mmol, 44.9 mg,) were added. The tube was evacuated and backfilled with argon three times. Then, under argon DMSO (1.0 mL) was added. The tube was closed tightly and the reaction was carried out at room temperature overnight. The reaction was diluted with ethyl acetate, the organic layer was washed with water and dried over $MgSO_4$ and purified by flash column chromatography (SiO_2; 100:0 to 20:80 pentane/EtOAc) to afford **6b** (38.3 mg, 48%) as a white solid.

TLC: R_f = 0.2 (40:60 pentane/EtOAc).

3'-methyl-10'-phenyl-7',8',8a',9',10',10a'-hexahydro-2H,5'H-spiro[pyrimidine-5,6'-[5,8,10](epimethanetriyl)cyclobuta[3,4]cycloocta[1,2-*b*]pyridine]-2,4,6(1H,3*H*)-trione (6b)

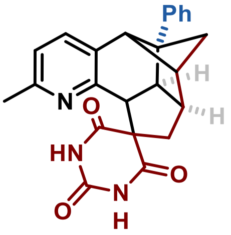

NMR Spectroscopy:

^{1}H NMR (400 MHz, DMSO-d_6): δ_H 11.09 (s, 1H), 10.99 (s, 1H), 7.40 (d, *J* = 7.7 Hz, 1H), 7.18 – 7.03 (m, 5H), 6.88 (d, *J* = 7.7 Hz, 1H), 3.67 (d, *J* = 6.0 Hz, 1H), 3.26 (ddd, *J* = 8.3, 6.1, 2.0 Hz, 1H), 3.18 (t, *J* = 2.3 Hz, 1H), 3.04 (q, *J* = 8.4 Hz, 1H), 2.94 (d, *J* = 2.9 Hz, 1H), 2.31 (s, 3H), 2.09 (dd, *J* = 6.8, 2.9 Hz, 1H), 1.81 (dd, *J* =

13.6, 9.0 Hz, 1H), 1.72 – 1.62 (m, 2H) ppm.

^{13}C NMR (101 MHz, DMSO-d$_6$): δ_C 173.2, 169.4, 155.8, 153.2, 151.3, 140.6, 136.4, 130.1, 128.6, 127.4, 126.8, 121.4, 68.4, 54.8, 54.1, 50.7, 50.2, 48.9, 48.6, 47.1, 28.0, 23.9 ppm.

HRMS (ESI$^+$): m/z calc'd for $C_{24}H_{22}N_3O_3$ [M+H]$^+$: 400.16557, found: 400.16569.

Selective Hydrogenation of Phenyl ring (6c)

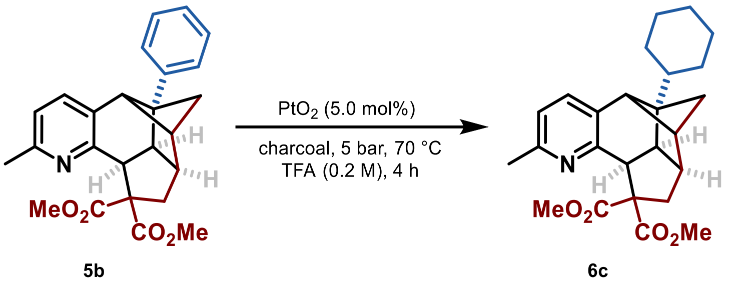

Following the modified literature procedure,[18] a 4.0 mL glass vial (screwcap with septum) was equipped with a stir bar, the compound **5b** (1.0 equiv., 0.2 mmol, 80.7 mg), charcoal (30.0 mg), PtO_2 (0.05 equiv., 0.01 mmol, 2.3 mg), TFA (1.0 mL). The glass vial was sonicated for 5 min. After that the vial was placed in a 150.0 mL stainless steel autoclave under air. The autoclave was pressurized and depressurized with hydrogen gas four times before the pressure was set to 5 bar. The reaction mixture was stirred at 70 °C for 4 h. Then the autoclave was carefully depressurized. The reaction mixture was quenched with aqueous $NaHCO_3$ solution (12.0 mL). Then the aqueous solution was extracted with CH_2Cl_2 (3 × 5.0 mL). The organic layer was dried over anhydrous $MgSO_4$ and filtered. The solvent was removed under reduced pressure and purified by flash column chromatography (SiO_2; 100:0 to 85:15 pentane/EtOAc) to afford **6c** (67.9 mg, 83%) as a colourless liquid.

TLC: R_f = 0.2 (90:10 pentane/EtOAc).

dimethyl-10-cyclohexyl-3-methyl-7,8,8a,9,10,10a-hexahydro-5,8,10-(epimethanetriyl)cyclobuta[3,4]cycloocta[1,2-*b*]pyridine-6,6(5*H*)-dicarboxylate (6c)

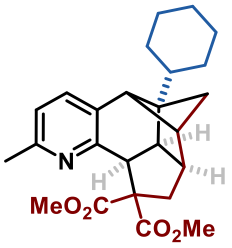

NMR Spectroscopy:

^{1}H NMR (400 MHz, CDCl$_3$): δ_H 7.20 (d, *J* = 7.7 Hz, 1H), 6.84 (d, *J* = 7.7 Hz, 1H), 4.08 (d, *J* = 5.8 Hz, 1H), 3.77 (d, *J* = 1.2 Hz, 3H), 3.69 (d, *J* = 1.1 Hz, 3H), 3.20 (ddd, *J* = 8.1, 5.8, 1.9 Hz, 1H), 2.72 – 2.68 (m, 1H), 2.65 – 2.58 (m, 1H), 2.46 – 2.38 (m, 4H), 2.13 (ddd, *J* = 14.0, 8.8, 1.4 Hz, 1H), 1.68 (dd, *J* = 13.9, 8.3 Hz, 1H), 1.64 – 1.52 (m, 6H), 1.36 (d, *J* = 12.3 Hz, 1H), 1.16 (d, *J* = 6.7 Hz, 1H), 1.06 – 0.80 (m, 5H) ppm.

^{13}**C NMR** (101 MHz, $CDCl_3$): δ_C 172.1, 169.6, 155.7, 155.5, 135.9, 130.1, 121.2, 69.8, 55.0, 52.8, 52.2, 49.6, 48.1, 47.3, 45.9, 43.9, 42.2, 37.2, 31.6, 29.7, 28.8, 26.5, 26.5, 26.4, 24.4 ppm.

HRMS (ESI$^+$): m/z calc'd for $C_{25}H_{31}NO_4Na$ $[M+Na]^+$: 432.21453, found: 432.21469.

Synthesis of Pyridine *N*-Oxide Derivative (6d)

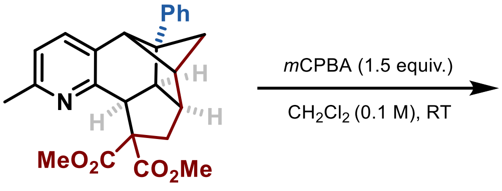

Following the modified literature procedure,[19] to an oven-dried 10.0 mL Schlenk tube compound **5b** (1.0 equiv., 0.3 mmol, 121.0 mg), *meta*-chloroperbenzoic acid (*m*CPBA, 1.5 equiv., 0.45 mmol, 77.6 mg) were added. The tube was evacuated and backfilled with argon three times and CH_2Cl_2 (0.1 M) was added to the tube. The reaction mixture was stirred at room temperature overnight. The reaction was quenched by saturated aqueous $NaHCO_3$ solution. The organic phase was collected with CH_2Cl_2 and solvent was removed under reduced pressure to give the product **6d** (119.5 mg, 95%). **m.p.** 236–238 °C.

6,6-bis(methoxycarbonyl)-3-methyl-10-phenyl-5,6,7,8,8a,9,10,10a-octahydro-5,8,10-(epimethanetriyl)cyclobuta[3,4]cycloocta[1,2-*b*]pyridine 4-oxide (6d)

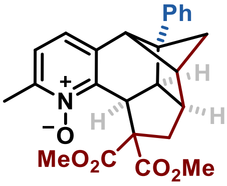

NMR Spectroscopy:

^{1}**H NMR** (400 MHz, $CDCl_3$): δ_H 7.22 – 7.15 (m, 2H), 7.15 – 7.09 (m, 1H), 7.08 – 7.00 (m, 3H), 6.97 (d, J = 7.8 Hz, 1H), 4.84 (d, J = 5.9 Hz, 1H), 3.88 (s, 3H), 3.64 (s, 3H), 3.40 (ddd, J = 8.2, 5.9, 1.9 Hz, 1H), 3.13 – 3.06 (m, 1H), 3.01 – 2.90 (m, 1H), 2.67 (q, J = 8.6 Hz, 1H), 2.43 (ddd, J = 13.7, 8.8, 1.3 Hz, 1H), 2.33 (s, 3H), 2.10 (dd, J = 7.0, 3.0 Hz, 1H), 1.82 (dd, J = 13.7, 8.3 Hz, 1H), 1.73 (d, J = 7.0 Hz, 1H) ppm.

^{13}**C NMR** (101 MHz, $CDCl_3$): δ_C 172.1, 169.4, 147.3, 146.5, 139.8, 134.7, 128.5, 126.9, 126.8, 124.5, 123.7, 67.3, 53.8, 53.1, 53.1, 50.8, 49.4, 49.2, 48.3, 44.7, 44.6, 33.6, 18.0 ppm.

HRMS (ESI$^+$): m/z calc'd for $C_{25}H_{25}NO_5Na$ $[M+Na]^+$: 442.16249, found: 442.16226.

COMPUTATIONAL STUDIES

Reaction Coordinate Calculations by DFT

Density functional theory (DFT) calculations were performed using the ORCA 6.0.1 software package[20] on the local high-performance computing cluster of the GLORIUS group, equipped with Intel® Xeon® Gold 6240 CPUs. Resulting structures were visualized in Avogadro[21] and Chemcraft. For the initial guess structure generation, a 3D conformer was generated using RDKit[22] starting from SMILES notation, followed by a conformer search using the GOAT[23] algorithm at the GFN2-xTB[24] level of theory.

Unrestricted DFT was used for all open-shell singlet and triplet calculations. We acknowledge that single-reference DFT methods have inherent limitations in describing the multi-configurational character of certain excited-state structures and especially events that involve changes of spins. However, given the structural complexity and size of the investigated systems, a good single-reference approach was employed to maintain computational feasibility. The spin contamination was monitored and now large deviation from the expected $< S^2 >$ values was observed.

Geometry optimizations, frequency calculations and intrinsic reaction coordinate (IRC) calculations were conducted using the ωB97X-D3 functional[25–27] and the def2-TZVP basis set.[28] No constraints on internal coordinates or symmetry were imposed. The geometries were confirmed to represent local minima on the potential energy surface by verifying the absence of imaginary frequencies after optimization. Transition states were confirmed by ensuring the presence of exactly one imaginary frequency, visualization of that frequency and conducting IRC calculations. Higher energy conformers were considered for transition state searches but are not explicitly shown in the main manuscript to keep clarity. Additional single point calculations for refining the electronic energies of stationary points were conducted at the ωB97X-D3/def2-QZVPPD level of theory.[25–28] All DFT calculations (optimization and single point refinements) included the def2/J auxiliary basis set,[29] the RIJCOSX approximation[30,31] and a CPCM implicit solvation model[32] with presets for toluene.

While this chosen method was not again benchmarked for this specific project, we argue that it is sufficiently accurate based on (a) benchmarks that we performed in earlier studies in our lab[33] and (b) more representatively, a recent report by the Paton group,[34] which extensively benchmarked a variety of different DFT functionals specifically for triplet state reactivity. In agreement with our qualitative observations, they undermined the broad and good performance of the employed wB97 functional family for the investigation of triplet state reactivities.

To determine the feasibility of triplet-triplet energy transfer processes, the calculation of dynamic vertical triplet energies was carried out according to a procedure by Paton and coworkers[35] in addition to the calculation of adiabatic triplet energies.

With a focus on FAIR data handling, all ORCA input scripts, optimized geometries and output files (including electronic energies and all thermodynamic corrections summaries) were uploaded and can be accessed through the IOChem-BD repository:[36] https://doi.org/10.19061/iochem-bd-6-603.

Structure assignments and species summary for calculated reaction coordinate

Structure Overview

3b: R = Ph
3e: R = 2-CF_3-Ph
3f: R = 2,4-CF_3-Ph

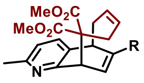

3: singlet and triplet

[1]TS-4
[1]TS-4b: R = Ph
[1]TS-4e: R = 2-CF_3-Ph
[1]TS-4f: R = 2,4-CF_3-Ph

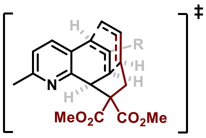

4b: R = Ph
4e: R = 2-CF_3-Ph
4f: R = 2,4-CF_3-Ph

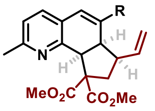

4: singlet and triplet;
two conformers each, vinyl group rotation)

[3]TS-Int-1
(6-*endo*-attack)

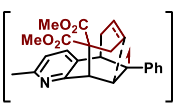

[3]Int-1 = [3]Int-2'

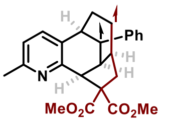

MECP-4b

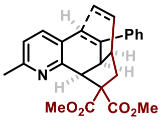

[3]TS-Int-2
5-*exo*-attack from
[3]4b (Conformer 2)

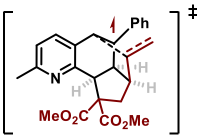

[3]TS-Int-2'
6-*endo*-attack from
[3]4b (Conformer 2)

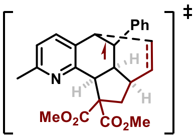

[3]TS-Int-2''
5-*endo*-attack from
[3]4b (Conformer 2)

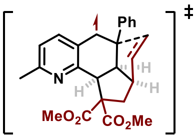

[3]Int-2

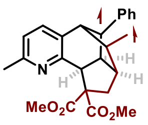

[3]Int-2'

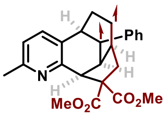

[3]Int-2''

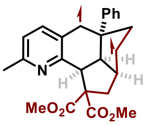

[3]Int-2'''

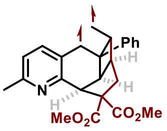

MECP-5b
(from **[3]Int-2**)

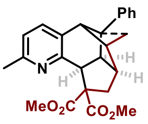

[1]5b
(from **[3]Int-2** via **MECP-5b**)
observed product

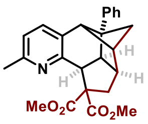

[1]5b'
(from **[3]Int-2'**)
not observed

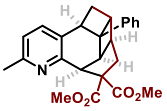

Energy summary

Supplementary Table 5: Summary of computed energies and thermodynamic corrections for all considered structures. All values given in Hartree.

Rxn Coor.	Structure	G_ TZVP	G_corrections TZVP	E_Singlepoint QZVPPD	G_total
1	3b S0	-1323.1039693	0.4021732	-1323.585899	-1323.1837262
1	3b T1	-1323.0151688	0.3961822	-1323.491012	-1323.0948303
1	3e S0	-1660.2155056	0.4034902	-1660.715063	-1660.3115727
1	3f S0	-1997.3313211	0.4038313	-1997.847756	-1997.4439249
2	TS-4e S0	-1660.1598165	0.4007290	-1660.65652	-1660.2557907
2	TS-4f S0	-1997.2756827	0.4011309	-1997.789304	-1997.3881732
2	TS-Int1 T1	-1323.0063474	0.3969172	-1323.482929	-1323.0860118
3	Int1 T1	-1323.0551762	0.3985778	-1323.533106	-1323.1345279
3	TS-4b S0	-1323.0499088	0.3998830	-1323.529442	-1323.1295595
4	MECP-4b			-1323.509025	
5	4b S0 Conf1	-1323.1129865	0.4000821	-1323.592807	-1323.1927251
5	4b S0 Conf2	-1323.1109442	0.4001425	-1323.590842	-1323.1906991
5	4b T1 Conf1	-1323.0379393	0.3945190	-1323.512141	-1323.1176219

**5**	**4b T1 Conf2**	-1323.0370485	0.3946155	-1323.511379	-1323.1167638
**6**	**TS-Int2 T1**	-1323.0157192	0.3952706	-1323.490716	-1323.0954452
**6**	**TS-Int2' T1**	-1323.0072334	0.3974168	-1323.484248	-1323.0868313
**6**	**TS-Int2'' T1**	-1323.0081907	0.3958932	-1323.483686	-1323.0877929
**6**	**TS-Int2''' T1**	-1323.0074744	0.3943574	-1323.481725	-1323.0873674
**7**	**Int2 T1**	-1323.0484155	0.3963809	-1323.524343	-1323.1279618
**7**	**Int2' T1**	-1323.0551728	0.3985692	-1323.533123	-1323.1345538
**7**	**Int2'' T1**	-1323.0501983	0.3972239	-1323.526992	-1323.1297686
**7**	**Int2''' T1**	-1323.0221605	0.3954356	-1323.497502	-1323.1020664
**8**	**MECP-5b**			-1323.504599	
**9**	**5b S0**	-1323.1179996	0.4060057	-1323.603553	-1323.1975477
**9**	**5b' S0**	-1323.0952075	0.4057176	-1323.580733	-1323.1750158

Spin density analysis

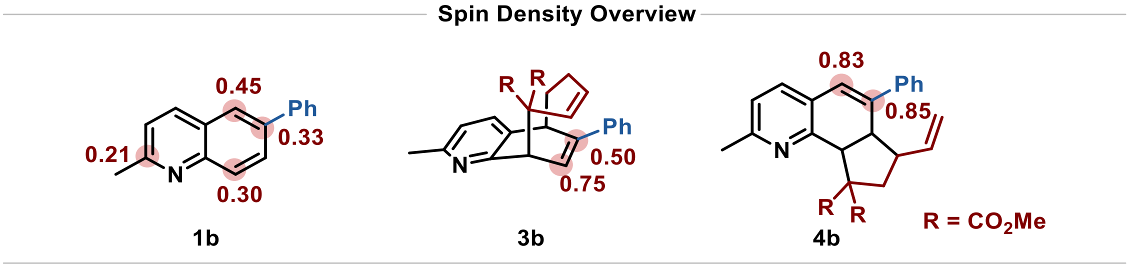

Supplementary Figure 11: Mulliken spin population of **1b**, **3b**, and **4b**

Comparison of SMD and CPCM solvation models

As suggested during the revisions of this manuscript, we investigated the influence of the solvation model on our results. To do so, we performed additional single point calculations using the SMD solvation model and directly compared it to our previous results using the CPCM model (**Supplementary Table 6**). We found a very strong correlation (R^2 = 0.9996) between the results of both solvation models, with the SMD model delivering a nearly constant shift in energy of ca. 0.510 hartree for every structure (**Supplementary Figure 12**). When inspecting the difference of relative Δ*G* values (comparing CPCM and SMD solvation), we found only minor fluctuations (mean 0.3 kcal/mol, absolute mean 0.4 kcal/mol), well below the computational error. Even when considering the "largest" difference between the SMD and CPCM model (ground state **4b**, adiabatic triplet energy increase of +0.9 kcal/mol), the overall picture of the proposed mechanism does not change at all, as relative barriers remain almost unchanged.

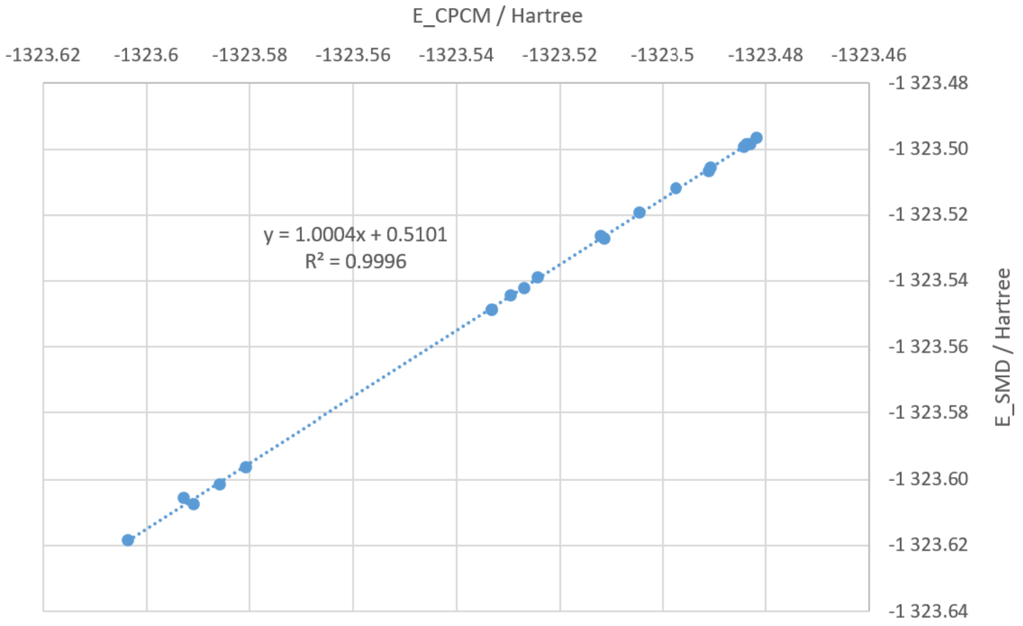

Supplementary Figure 12: Correlation between single point energies using the CPCM and SMD model for optimized structures shows constant shift of absolute energy, but no major systematic and random deviation.

Supplementary Table 6: Single point energy comparison using the CPCM and SMD model for every optimized structure.

Structure	E_sp_CPCM / hartree	E_sp_SMD / hartree	rel. difference / kcal mol^{-1}
3b S0	-1323.585899	-1323.601334	0.0
3b T1	-1323.491012	-1323.506645	-0.1
TS-Int1 T1	-1323.482929	-1323.498508	-0.1
Int1 T1	-1323.533106	-1323.54868	-0.1
TS-4b S0	-1323.529442	-1323.544236	0.4
4b S0 Conf1	-1323.592807	-1323.60556	1.7
4b S0 Conf2	-1323.590842	-1323.607532	-0.8
4b T1 Conf1	-1323.512141	-1323.526317	0.8
4b T1 Conf2	-1323.511379	-1323.527095	-0.2
TS-Int2 T1	-1323.490716	-1323.505464	0.4
TS-Int2' T1	-1323.484248	-1323.499305	0.2
TS-Int2'' T1	-1323.483686	-1323.498616	0.3
TS-Int2''' T1	-1323.481725	-1323.496447	0.4
Int2 T1	-1323.524343	-1323.53898	0.5
Int2' T1	-1323.533123	-1323.548707	-0.1
Int2'' T1	-1323.526992	-1323.542137	0.2
Int2''' T1	-1323.497502	-1323.511847	0.7
MECP-5b	-1323.504599	-1323.519278	0.5
5b S0	-1323.603553	-1323.618488	0.3
5b' S0	-1323.580733	-1323.596166	0.0

Investigation of the influence of aromatic substitutions

As correctly pointed out by one of the reviewers of this work, electron-rich arenes at the 6-position of the quinoline were observed to remain unreactive under our reaction conditions. To investigate the influence of aromatic substitutions (at 6-position) on the properties of the quinoline starting materials, adiabatic triplet energies, oxidation potentials (following this procedure[37]) and vertical ionization potentials were calculated for a row of model substrates (**Supplementary Figure 13**).

	E_T / kcal mol	E_ox vs. SCE / V	IP / kcal mol
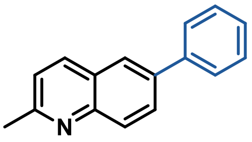	60.1	2.32	162.4
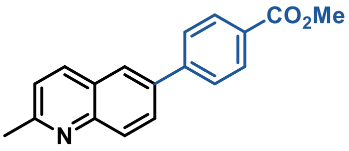	59.4	2.44	165.5
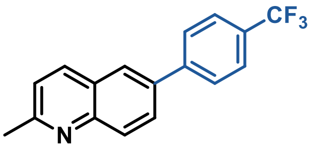	59.9	2.51	167.0
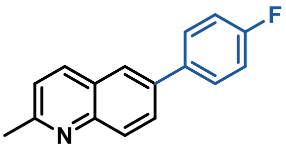	59.7	2.32	163.4
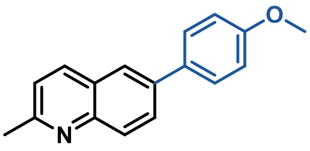	58.9	1.92	154.5

Supplementary Figure 13: Adiabatic triplet energies, oxidation potentials and vertical ionization potentials of selected substrates.

Noteworthy, no dependence of the triplet energy of the starting material on the arene substitution was observed, in line with the localization of the spin densities within the quinoline bicycle rather than the arene at 6-position. However, the introduction of electron-donating groups strongly reduced the oxidation potential to ~1.9 V vs. SCE, compared to ca. 2.5 V vs. SCE in case of electron-withdrawing groups. Considering Lewis/Bronsted acid activation under our reaction conditions (which was not modelled in the above comparison for simplicity), this opens the possibility for photocatalyst quenching *via* an electron transfer mechanism (reductive PC quenching).

Therefore, we hypothesize that the scope limitation of electron-rich arenes stems from preferred unproductive reductive deactivation of the photocatalyst, outcompeting the desired energy transfer mechanism. This is in line with the observation of remaining starting material rather than decomposition.

Dynamic Vertical Triplet Energies

Dynamic vertical triplet energies were calculated using an adapted method originally developed by Paton and colleagues.[35] To obtain initial MD structures, geometry optimizations and high precision frequency calculations (at the M06-2X/6-311++G(d,p) level of theory)[38,39] were carried out using Gaussian 16[40] on the Palma II high performance computing cluster of the University of Münster. Based on this result, 100 MD trajectories were generated using the *milo* package developed by ESS and colleagues[41] interfering with Gaussian 16, also on the Palma II cluster. The milo MD simulations were run for 200 fs at the same level of theory with a step size of 1 fs at a temperature of 298.15 K. Snapshots of the geometry were extracted every 5 fs from every simulation, yielding 4000 geometries in total.

For each of the geometries, ORCA single point calculation job scripts (at M06-2X/6-311++G(d,p)) level of theory, D3zero dispersion correction, CPCM model for MeCN were automatically generated for the singlet and triplet state and executed in parallel on the local computing cluster of the GLORIUS group.

Vertical singlet-triplet gaps were extracted for every snapshot. The collection of vertical gaps was subsequently plotted in a histogram using *matplotlib*[42] and approximated by Kernel-Density Estimation (KDE) in *scikit-learn*.[43] The corresponding cumulative distribution function (CDF) was calculated, and dynamic vertical triplet energies were determined as the energy including 0.8% of the distribution. 5.2.1. Optimized geometries for the MD simulation initialization

All optimized geometries that were used to initialize the MD simulations can be found along with their Gaussian and milo input and output files in the IOChem-BD repository using the collection link provided in chapter **5.1.**

Vertical triplet energy distributions and DvTEs

DvTE of 3b

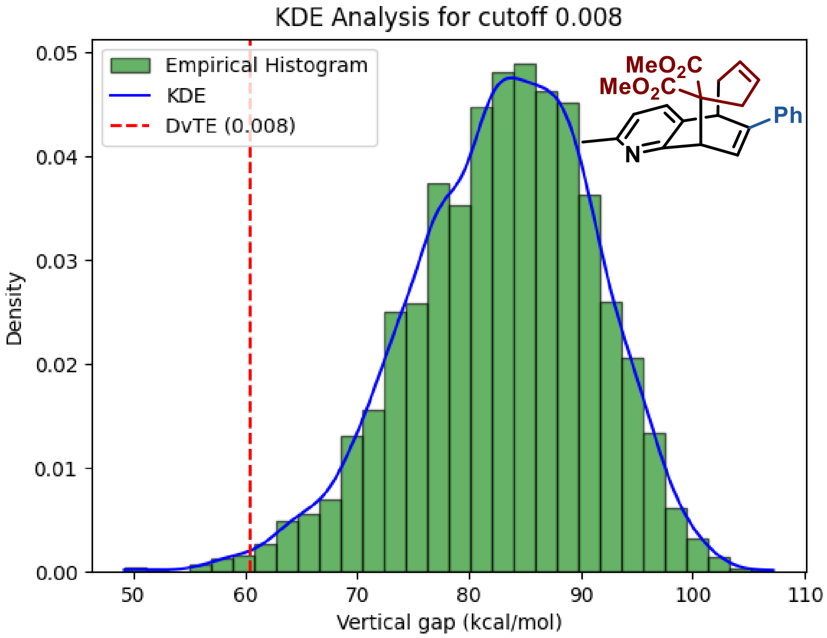

Supplementary Figure 14: Vertical triplet energy distribution of **3b**. Fitted using kernel density estimation and DvTE was determined to include 0.8% of the distribution (DvTE = 60.5 kcal/mol)

DvTE of 3e

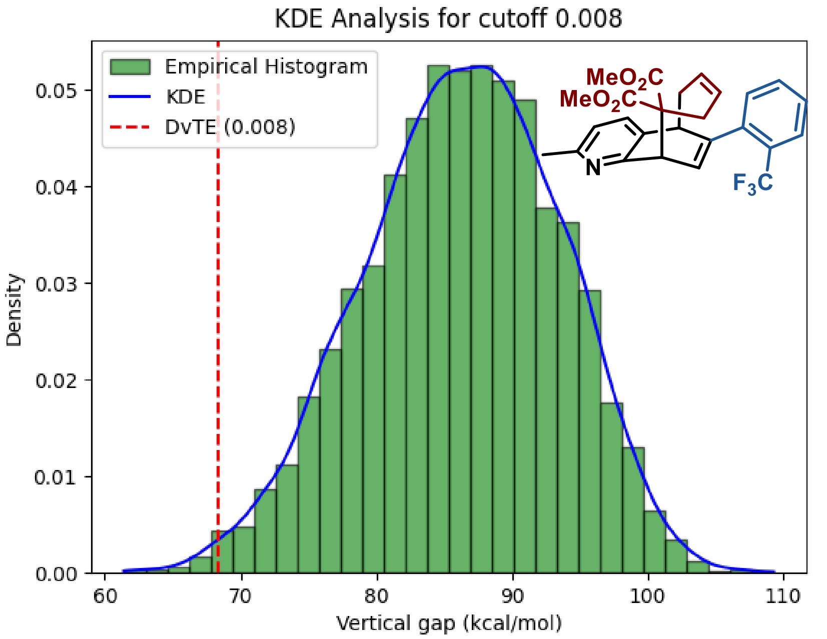

Supplementary Figure 15: Vertical triplet energy distribution of **3e**. Fitted using kernel density estimation and DvTE was determined to include 0.8% of the distribution (DvTE = 68.4 kcal/mol).

DvTE of 3f

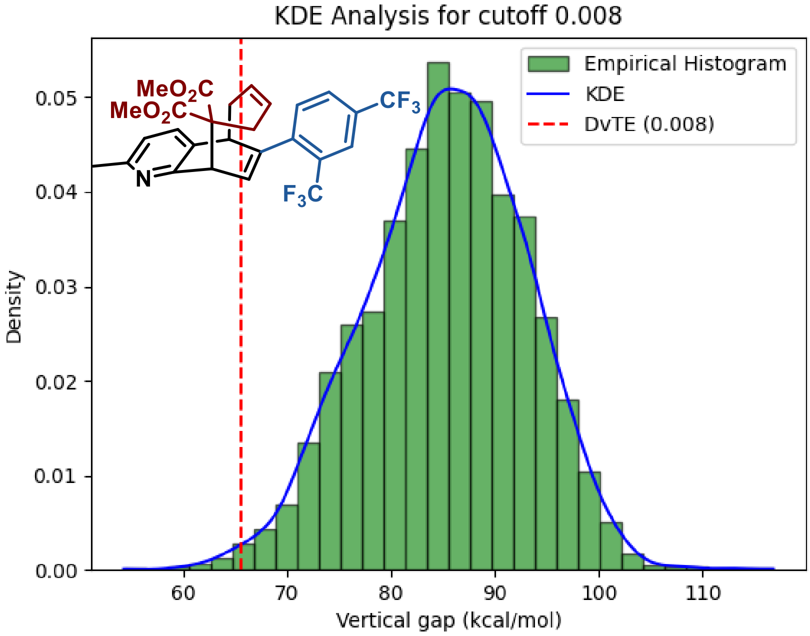

Supplementary Figure 16: Vertical triplet energy distribution of 3d. Fitted using kernel density estimation and DvTE was determined to include 0.8% of the distribution (DvTE = 65.6 kcal/mol).

DvTE of 4b

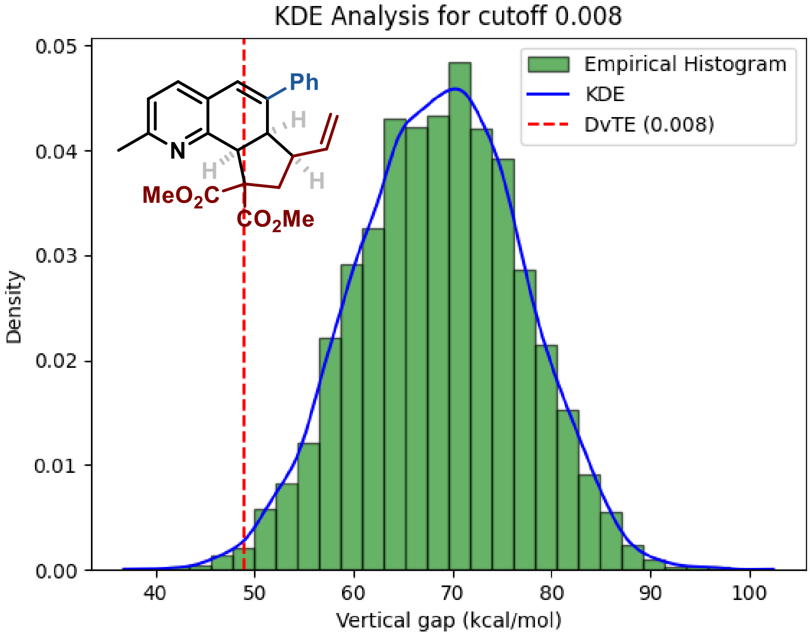

Supplementary Figure 17: Vertical triplet energy distribution of **4b**. Fitted using kernel density estimation and DvTE was determined to include 0.8% of the distribution (DvTE = 48.9 kcal/mol).

DvTE of 3as

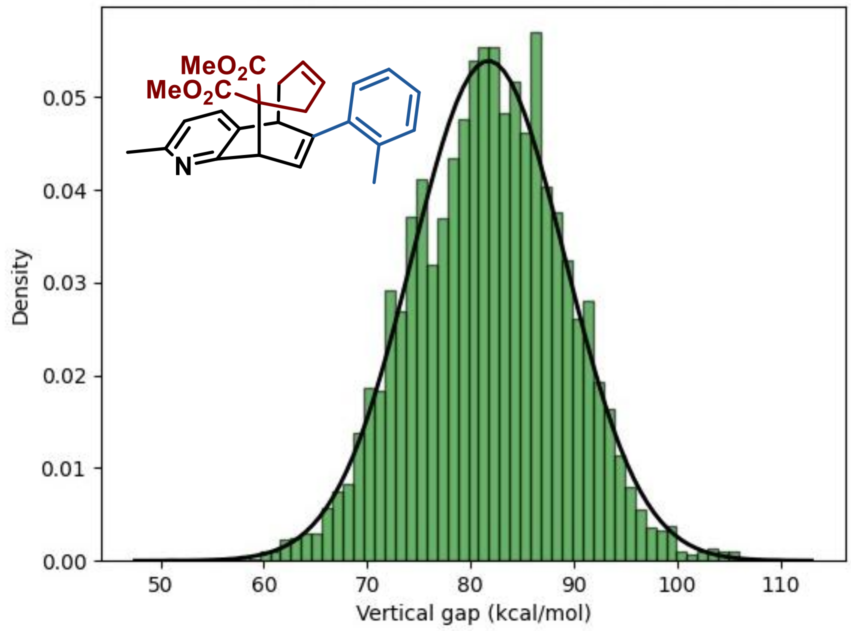

Supplementary Figure 18: Vertical triplet energy distribution of **3as**. Fitted using kernel density estimation and DvTE was determined to include 0.8% of the distribution (DvTE = 63.9 kcal/mol).

Acknowledgements

All calculations in Gaussian16 that were made for this publication were performed on the HPC cluster PALMA II of the University of Münster, subsidized by the DFG (INST 211/667-1).

CRYSTAL STRUCTURES

X-ray crystal structure analysis of 3a: A colourless, plate-shaped crystal was mounted on a MiTeGen micromount with perfluoroether oil. The crystals were crystallised from DCM and pentane. Data for **3a** were collected from a single crystal in 2.53 hours at 100(2) K on a Bruker D8 VENTURE KAPPA diffractometer with a microfocus sealed tube using a multilayer mirror as monochromator and a Bruker PHOTON III CPAD detector. The diffractometer used Mo K_α radiation (λ = 0.71073 Å). All data were integrated with SAINT V8.41, yielding 68712 reflections of which 7600 where independent and 91.6% were greater than $2\sigma(F^2)$.[44] A Multi-Scan absorption correction using SADABS 2016/2 was applied.[45] The structure was solved by Intrinsic Phasing methods with SHELXT 2018/2 and refined by full-matrix least-squares methods against F^2 using SHELXL-2019/2.[46,47] All non-hydrogen atoms were refined with anisotropic displacement parameters. All hydrogen atoms were refined isotropic on calculated positions using a riding model with their U_{iso} values constrained to 1.5 times the U_{eq} of their pivot atoms for terminal sp^3 carbon atoms and 1.2 times for all other carbon atoms.

Crystallographic data for the structures reported in this paper have been deposited with the Cambridge Crystallographic Data Centre.[48] CCDC 2502653 contain the supplementary crystallographic data for this paper. These data can be obtained free of charge from The Cambridge Crystallographic Data Centre via www.ccdc.cam.ac.uk/structures. This report and the CIF file were generated using FinalCif.

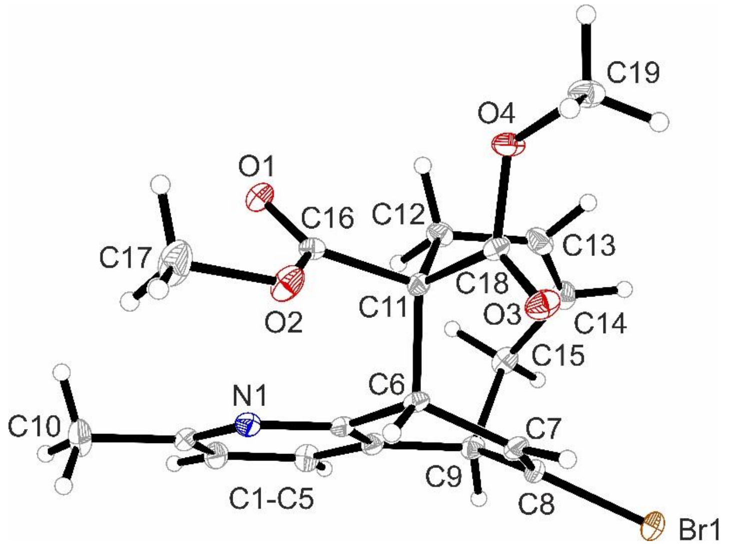

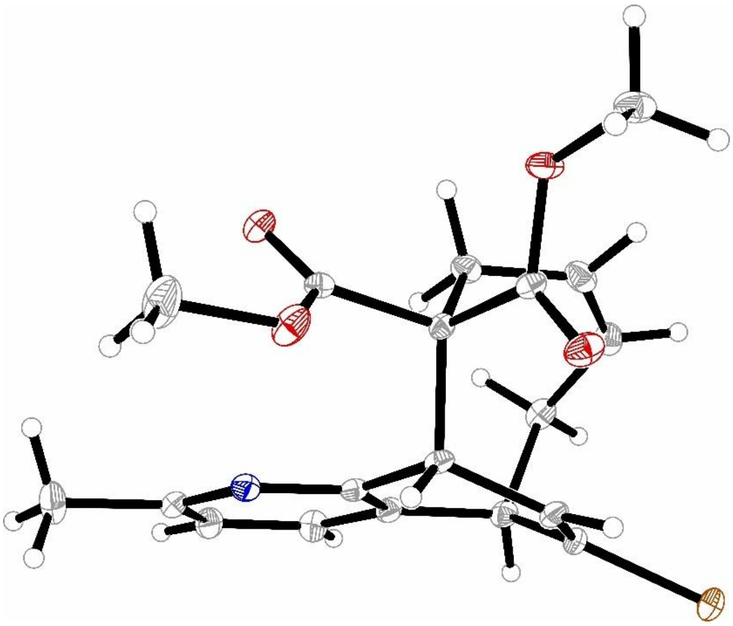

Supplementary Figure 19: Crystal structure of compound **3a**. Only one molecule (molecule named with suffix B) of two found in the asymmetric unit is shown.
Thermal ellipsoids are shown at 50% probability.

X-ray crystal structure analysis of 3b: A colourless, prism-shaped crystal was mounted on a MiTeGen micromount with perfluoroether oil. The crystals were crystallised from DCM and pentane. Data for **3b** were

collected from a single crystal in 2.68 hours at 100(2) K on a Bruker D8 VENTURE KAPPA diffractometer with a microfocus sealed tube using a multilayer mirror as monochromator and a Bruker PHOTON III CPAD detector. The diffractometer was equipped with an Oxford Cryostream 1000 low temperature device and used Mo K_α radiation (λ = 0.71073 Å). All data were integrated with SAINT V8.41, yielding 32548 reflections of which 4313 where independent and 97.5% were greater than $2\sigma(F^2)$.[44] A Multi-Scan absorption correction using SADABS 2016/2 was applied.[45] The structure was solved by Intrinsic Phasing methods with SHELXT 2018/2 and refined by full-matrix least-squares methods against F^2 using SHELXL-2019/2.[46,47] All non-hydrogen atoms were refined with anisotropic displacement parameters. All hydrogen atoms were refined isotropic on calculated positions using a riding model with their U_{iso} values constrained to 1.5 times the U_{eq} of their pivot atoms for terminal sp^3 carbon atoms and 1.2 times for all other carbon atoms. Crystallographic data for the structures reported in this paper have been deposited with the Cambridge Crystallographic Data Centre.[48] CCDC 2502658 contain the supplementary crystallographic data for this paper. These data can be obtained free of charge from The Cambridge Crystallographic Data Centre via www.ccdc.cam.ac.uk/structures. This report and the CIF file were generated using FinalCif.

*Special refinement details for **3b**:* One COOMe group is disordered over two positions. Several restraints (SIMU, SAME, SADI and ISOR) were used in order to improve refinement stability.

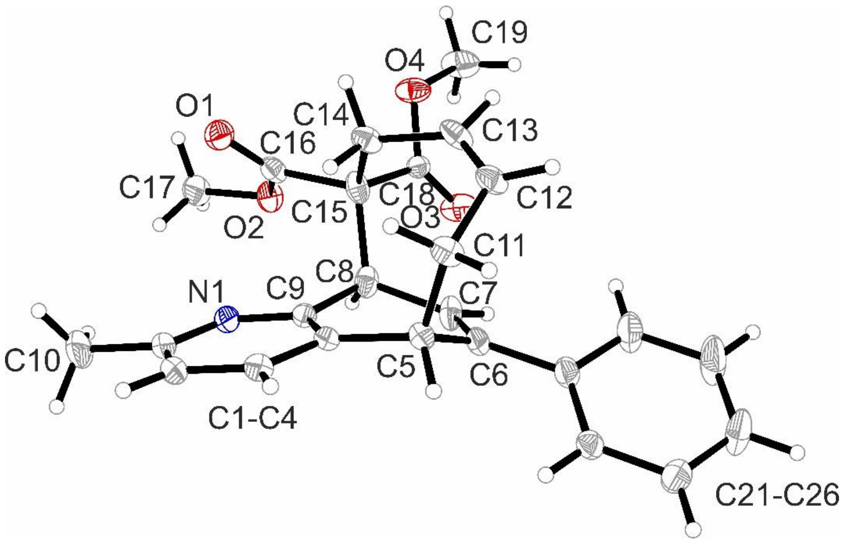

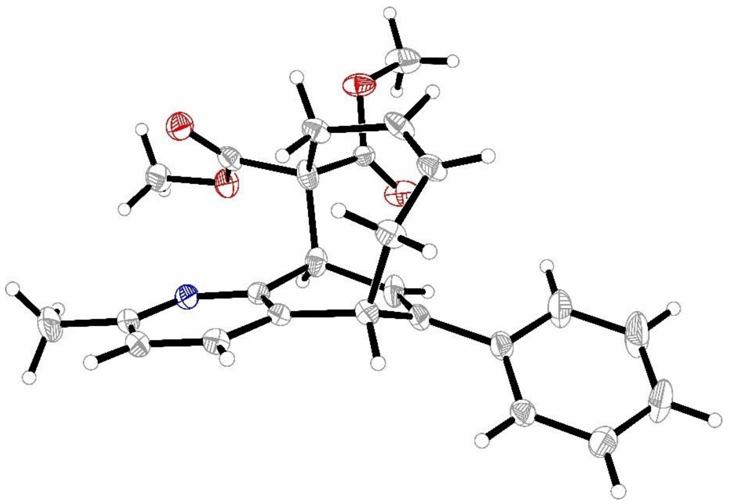

Supplementary Figure 20: Crystal structure of compound **3b**.
Thermal ellipsoids are shown at 50% probability.
Compound **3b** crystallized “by chance” with only one enantiomer in the unit cell, in the chiral space group *C*2.

X-ray crystal structure analysis of 3f: A colourless, prism-shaped crystal was mounted on a MiTeGen micromount with perfluoroether oil. The crystals were crystallised from DCM and pentane. Data for **3f** were collected from a single crystal in 6.19 hours at 100(2) K on a Bruker D8 VENTURE KAPPA diffractometer with a microfocus sealed tube using a multilayer mirror as monochromator and a Bruker PHOTON III CPAD detector. The diffractometer used Mo K_α radiation (λ = 0.71073 Å). All data were integrated with SAINT V8.41, yielding 83911 reflections of which 5621 where independent and 76.5% were greater than $2\sigma(F^2)$.[44] A Multi-Scan absorption correction using SADABS 2016/2 was applied.[45] The structure was solved by Intrinsic Phasing methods with SHELXT 2018/2 and refined by full-matrix least-squares methods against F^2 using SHELXL-2019/2.[46,47] All non-hydrogen atoms were refined with anisotropic displacement parameters. All hydrogen atoms were refined isotropic on calculated positions using a riding model with their U_{iso} values constrained to 1.5 times the U_{eq} of their pivot atoms for terminal sp^3 carbon atoms and 1.2 times for all other carbon atoms. Crystallographic data for the structures reported in this paper have been deposited with the Cambridge Crystallographic Data Centre.[48] CCDC 2502654 contain the supplementary crystallographic data for this paper. These data can be obtained free of charge from The Cambridge Crystallographic Data Centre via www.ccdc.cam.ac.uk/structures. This report and the CIF file were generated using FinalCif.

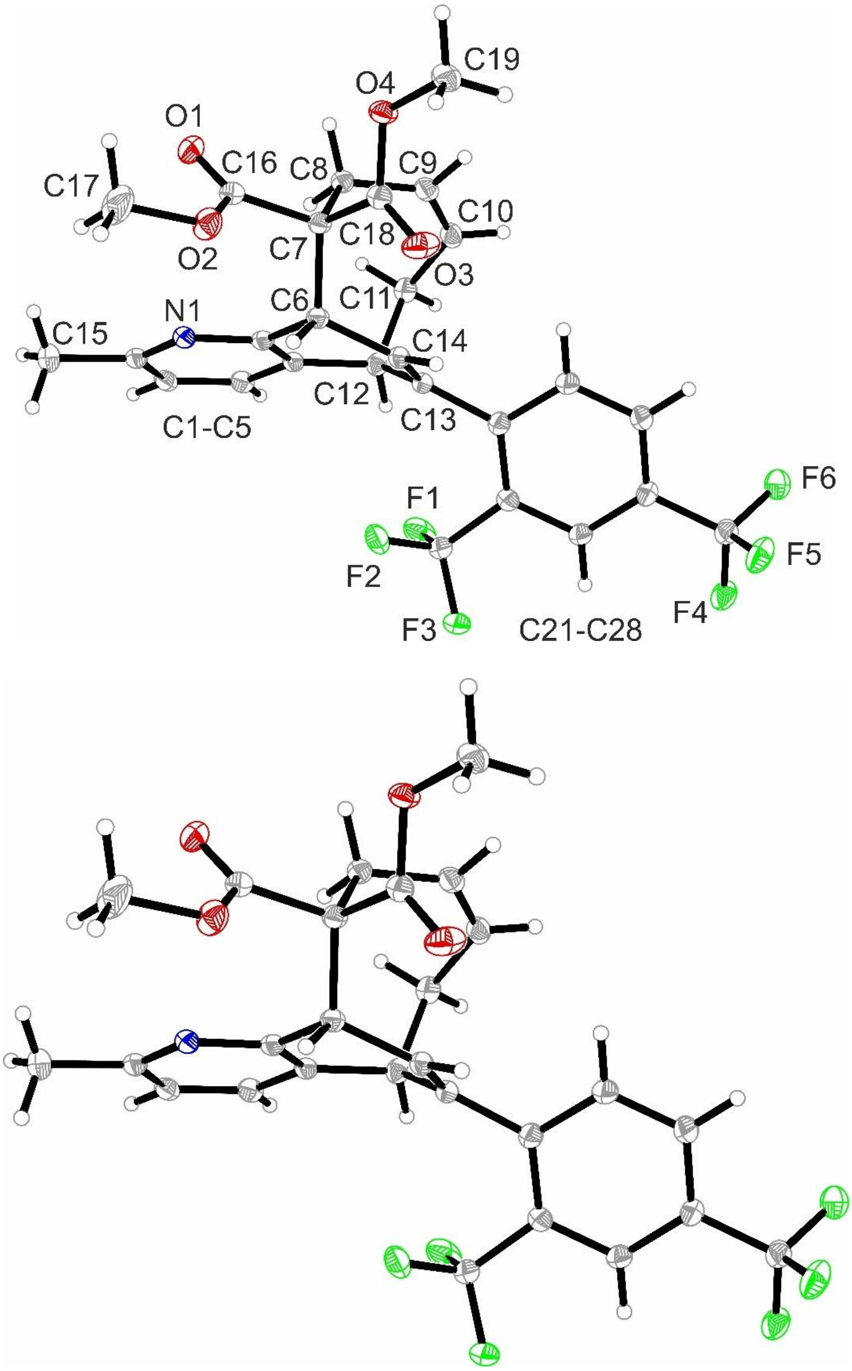

Supplementary Figure 21: Crystal structure of compound **3f**.
Thermal ellipsoids are shown at 50% probability.

X-ray crystal structure analysis of 4a: A colourless, prism-shaped crystal was mounted on a MiTeGen micromount with perfluoroether oil. The crystals were crystallised from DCM and pentane. Data for **4a** were collected from a single crystal in 2.43 hours at 100(2) K on a Bruker D8 VENTURE KAPPA diffractometer with a microfocus sealed tube using a multilayer mirror as monochromator and a Bruker PHOTON III CPAD detector. The diffractometer used Mo K_{α} radiation (λ = 0.71073 Å). All data were integrated with SAINT V8.41, yielding 45740 reflections of which 3898 where independent and 97.1% were greater than $2\sigma(F^2)$.[44] A Multi-

Scan absorption correction using SADABS 2016/2 was applied.[45] The structure was solved by Intrinsic Phasing methods with SHELXT 2018/2 and refined by full-matrix least-squares methods against F^2 using SHELXL-2019/2.[46,47] All non-hydrogen atoms were refined with anisotropic displacement parameters. All hydrogen atoms were refined isotropic on calculated positions using a riding model with their U_{iso} values constrained to 1.5 times the U_{eq} of their pivot atoms for terminal sp^3 carbon atoms and 1.2 times for all other carbon atoms. Crystallographic data for the structures reported in this paper have been deposited with the Cambridge Crystallographic Data Centre.[48] CCDC 2502652 contain the supplementary crystallographic data for this paper. These data can be obtained free of charge from The Cambridge Crystallographic Data Centre via www.ccdc.cam.ac.uk/structures. This report and the CIF file were generated using FinalCif.

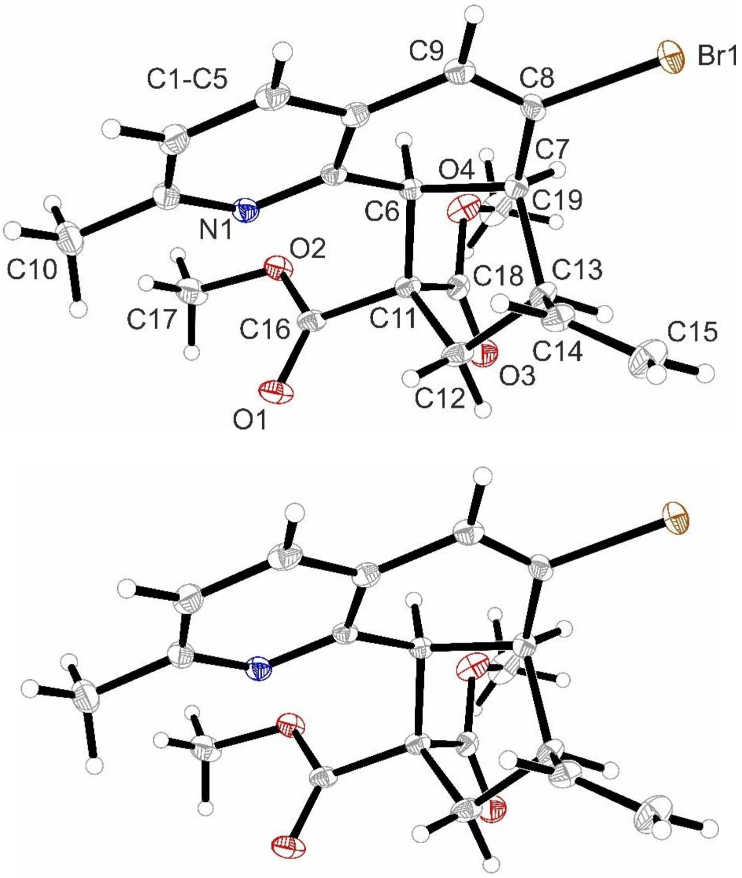

Supplementary Figure 22: Crystal structure of compound **4a**.
Thermal ellipsoids are shown at 50% probability.

X-ray crystal structure analysis of 5h: A colourless, prism-shaped crystal was mounted on a loop with perfluoroether oil. The crystals were crystallised from DCM and pentane. Data for **5h** were collected from a single crystal in 18.58 hours at 100(2) K on a Bruker D8 VENTURE KAPPA diffractometer with a microfocus sealed tube using a multilayer mirror as monochromator and a Bruker PHOTON III CPAD detector. The diffractometer was equipped with an Oxford Cryostream 1000 low temperature device and used CuK_{α} radiation

(λ = 1.54178 Å). All data were integrated with SAINT V8.41, yielding 72720 reflections of which 4441 where independent and 97.0% were greater than 2σ(F^2).[44] A Multi-Scan absorption correction using SADABS 2016/2 was applied.[45] The structure was solved by Intrinsic Phasing methods with SHELXT 2018/2 and refined by full-matrix least-squares methods against F^2 using SHELXL-2019/2.[46,47] All non-hydrogen atoms were refined with anisotropic displacement parameters. All hydrogen atoms were refined isotropic on calculated positions using a riding model with their U_{iso} values constrained to 1.5 times the U_{eq} of their pivot atoms for terminal sp^3 carbon atoms and 1.2 times for all other carbon atoms. Crystallographic data for the structures reported in this paper have been deposited with the Cambridge Crystallographic Data Centre.[48] CCDC 2502657 contain the supplementary crystallographic data for this paper. These data can be obtained free of charge from The Cambridge Crystallographic Data Centre via www.ccdc.cam.ac.uk/structures. This report and the CIF file were generated using FinalCif.

<u>*Special refinement details for* ***5h****:*</u> The *p*-CF_3-Ph substituent is disordered over three positions. Several restraints (SIMU, SAME, SADI and ISOR) were used in order to improve refinement stability.

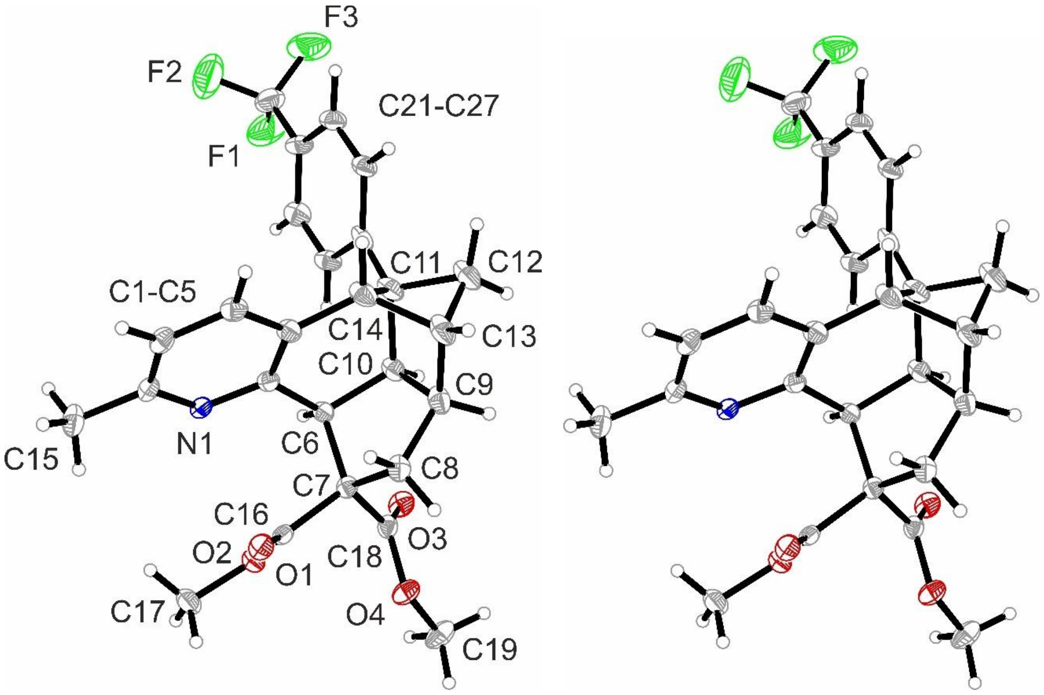

Supplementary Figure 23: Crystal structure of compound **5h**.
Thermal ellipsoids are shown at 50% probability.

X-ray crystal structure analysis of 5ad: A colourless, prism-shaped crystal was mounted on a MiTeGen micromount with perfluoroether oil. The crystals were crystallised from DCM and pentane. Data for **5ad** were collected from a single crystal in 3.55 hours at 100(2) K on a Bruker D8 VENTURE KAPPA diffractometer with a microfocus sealed tube using a multilayer mirror as monochromator and a Bruker PHOTON III CPAD

detector. The diffractometer used Mo K_{α} radiation (λ = 0.71073 Å). All data were integrated with SAINT V8.41, yielding 36370 reflections of which 4525 where independent and 86.8% were greater than $2\sigma(F^2)$.[44] A Multi-Scan absorption correction using SADABS 2016/2 was applied.[45] The structure was solved by Intrinsic Phasing methods with SHELXT 2018/2 and refined by full-matrix least-squares methods against F^2 using SHELXL-2019/2.[46,47] All non-hydrogen atoms were refined with anisotropic displacement parameters. All hydrogen atoms were refined isotropic on calculated positions using a riding model with their U_{iso} values constrained to 1.5 times the U_{eq} of their pivot atoms for terminal sp^3 carbon atoms and 1.2 times for all other carbon atoms. Crystallographic data for the structures reported in this paper have been deposited with the Cambridge Crystallographic Data Centre.[48] CCDC 2502655 contain the supplementary crystallographic data for this paper. These data can be obtained free of charge from The Cambridge Crystallographic Data Centre via www.ccdc.cam.ac.uk/structures. This report and the CIF file were generated using FinalCif.

Special refinement details for **5ad**: A part of a H2O molecule was found in the asymmetric unit and could not be satisfactorily refined. The *PLATON* SQUEEZE tool[49] was therefore used to remove mathematically the effect of the solvent. The quoted formula and derived parameters are not included the squeezed solvent molecule.

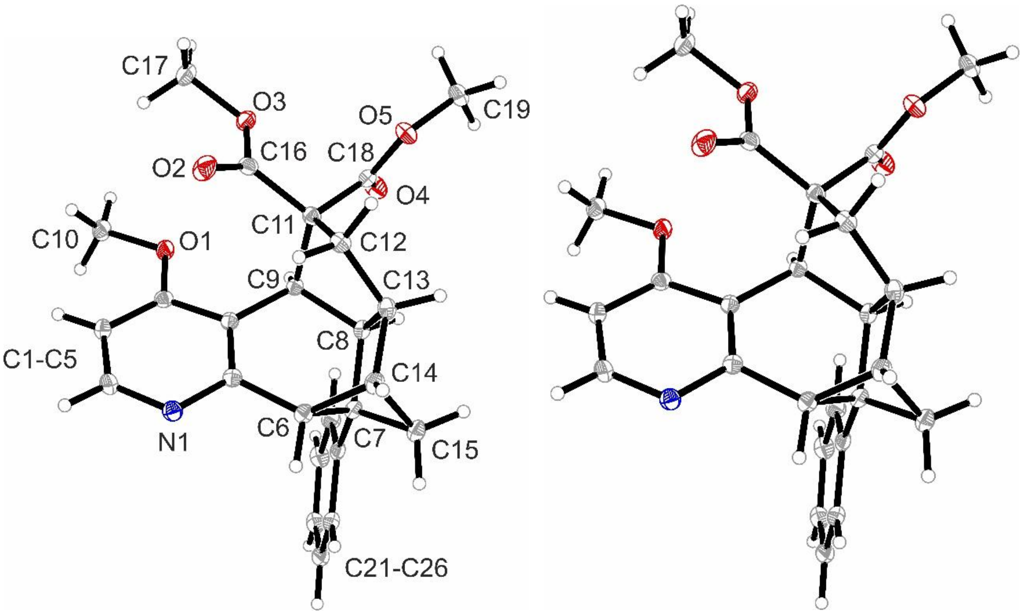

Supplementary Figure 24: Crystal structure of compound **5ad**.
Thermal ellipsoids are shown at 50% probability.

X-ray crystal structure analysis of 5ag: A colourless, plate-shaped crystal was mounted on a loop with perfluoroether oil. The crystals were crystallised from DCM and pentane. Data for **5ag** were collected from a single crystal in 17.78 hours at 100(2) K on a Bruker D8 VENTURE KAPPA diffractometer with a microfocus sealed tube using a multilayer mirror as monochromator and a Bruker PHOTON III CPAD detector. The diffractometer was equipped with an Oxford Cryostream 1000 low temperature device and used Mo K_{α} radiation (λ = 0.71073 Å). All data were integrated with SAINT V8.41, yielding 46440 reflections of which 9032 where

independent and 65.4% were greater than 2σ(F^2).[44] A Multi-Scan absorption correction using SADABS 2016/2 was applied.[45] The structure was solved by Intrinsic Phasing methods with SHELXT 2018/2 and refined by full-matrix least-squares methods against F^2 using SHELXL-2019/2.[46,47] All non-hydrogen atoms were refined with anisotropic displacement parameters. All hydrogen atoms were refined isotropic on calculated positions using a riding model with their U_{iso} values constrained to 1.5 times the U_{eq} of their pivot atoms for terminal sp^3 carbon atoms and 1.2 times for all other carbon atoms. Crystallographic data for the structures reported in this paper have been deposited with the Cambridge Crystallographic Data Centre.[48] CCDC 2502656 contain the supplementary crystallographic data for this paper. These data can be obtained free of charge from The Cambridge Crystallographic Data Centre via www.ccdc.cam.ac.uk/structures. This report and the CIF file were generated using FinalCif.

*Special refinement details for **5ag**:* One dichloromethane molecule is disordered over two positions. Several restraints (SIMU, SAME, SADI and ISOR) were used in order to improve refinement stability.

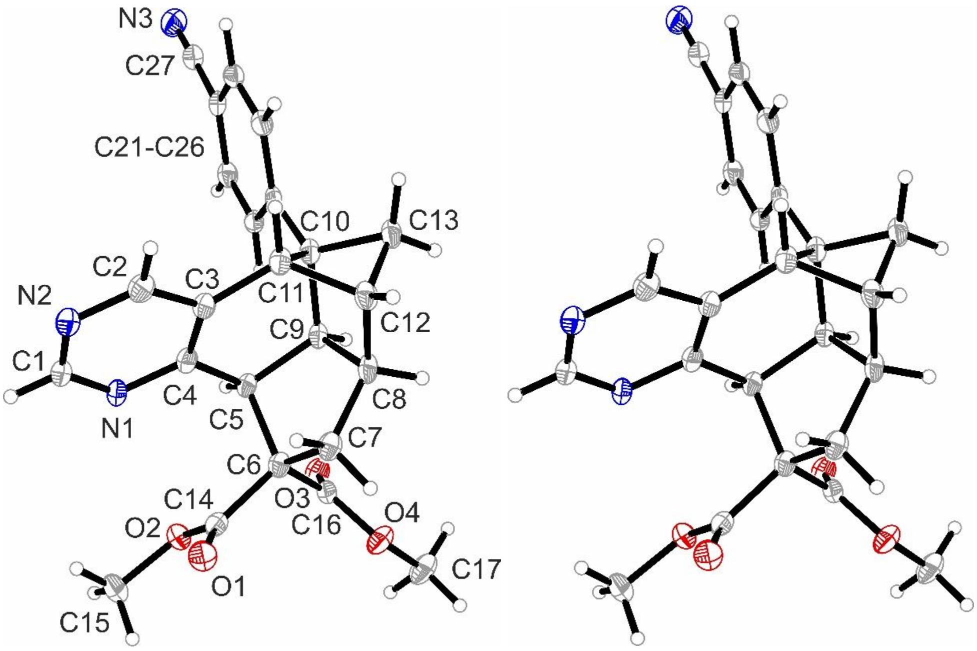

Supplementary Figure 25: Crystal structure of compound **5ag**. Only one molecule (molecule named with suffix A) of two found in the asymmetric unit is shown.
Thermal ellipsoids are shown at 50% probability.

X-ray crystal structure analysis of 5ao: A colourless, plate-shaped crystal was mounted on a MiTeGen micromount with perfluoroether oil. The crystals were crystallised from DCM and pentane. Data for **5ao** were collected from a single crystal in 2.78 hours at 100(2) K on a Bruker D8 VENTURE KAPPA diffractometer with a microfocus sealed tube using a multilayer mirror as monochromator and a Bruker PHOTON III CPAD detector. The diffractometer used Mo K_{α} radiation (λ = 0.71073 Å). All data were integrated with SAINT V8.41, yielding 29999 reflections of which 5106 where independent and 84.1% were greater than 2σ(F^2).[44] A Multi-

Scan absorption correction using SADABS 2016/2 was applied.[45] The structure was solved by Intrinsic Phasing methods with SHELXT 2018/2 and refined by full-matrix least-squares methods against F^2 using SHELXL-2019/2.[46,47] All non-hydrogen atoms were refined with anisotropic displacement parameters. All hydrogen atoms were refined with isotropic displacement parameters. Some of their coordinates were refined freely and some on calculated positions using a riding model with their U_{iso} values constrained to 1.5 times the U_{eq} of their pivot atoms for terminal sp^3 carbon atoms and 1.2 times for all other carbon atoms. Crystallographic data for the structures reported in this paper have been deposited with the Cambridge Crystallographic Data Centre.[48] CCDC 2502659 contain the supplementary crystallographic data for this paper. These data can be obtained free of charge from The Cambridge Crystallographic Data Centre via www.ccdc.cam.ac.uk/structures. This report and the CIF file were generated using FinalCif.

*Special refinement details for **5ao***: The hydrogen at N1 atom was refined freely but with N-H distance restraint (DFIX).

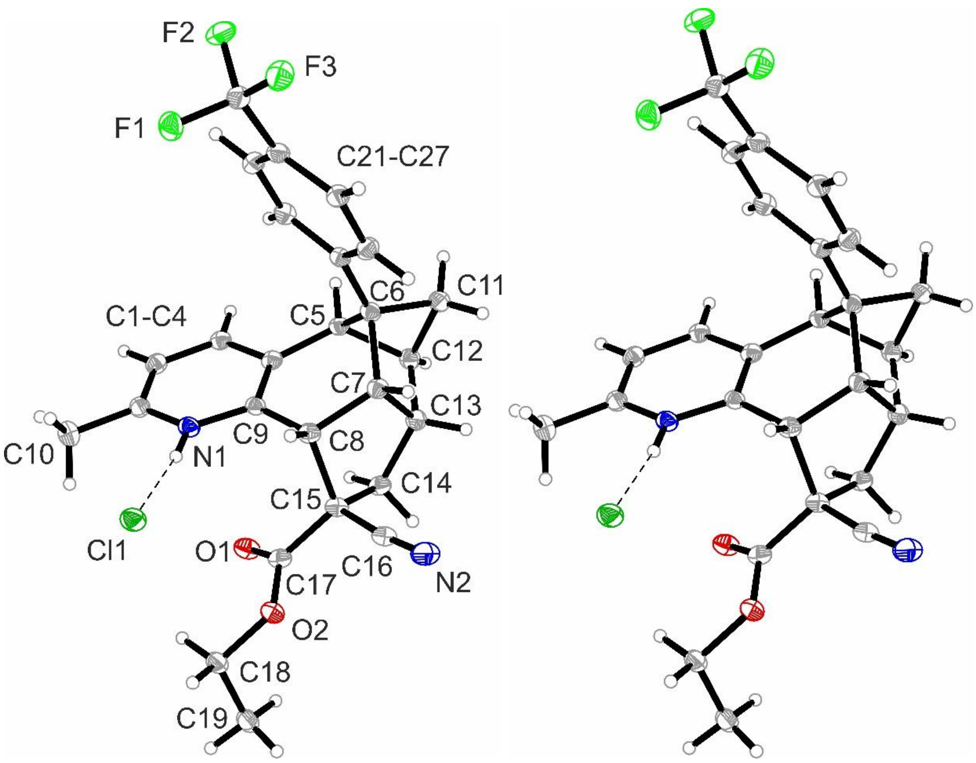

Supplementary Figure 26: Crystal structure of compound **5ao**.
Thermal ellipsoids are shown at 50% probability.
Compound **5ao'** crystallized "by chance" with only one enantiomer
in the unit cell, in the chiral space group $P2_12$

Supplementary Table 7: Crystal data and structure refinement for compounds **3a**, **3b**, **3f** and **4a**:

CCDC number	**3a -** 2502653		**3f -** 2502654	**4a -** 2502652
Empirical formula	$C_{19}H_{20}BrNO_4$	$C_{25}H_{25}NO_4$	$C_{27}H_{23}F_6NO_4$	$C_{19}H_{20}BrNO_4$
Formula weight	406.27	403.46	539.46	406.27
Temperature [K]	100(2)	100(2)	100(2)	100(2)
Crystal system	monoclinic	monoclinic	monoclinic	monoclinic
Space group (number)	$P2_1/c$ (14)	$C2$ (5)	$C2/c$ (15)	$P2_1/n$ (14)
a [Å]	14.4978(7)	25.2596(6)	45.5403(17)	10.8112(6)
b [Å]	7.5996(3)	6.9396(2)	8.0714(3)	17.4261(9)
c [Å]	32.7748(13)	11.6918(3)	13.2845(4)	11.0987(7)
α [°]	90	90	90	90
β [°]	99.795(2)	96.6470(10)	92.4720(10)	118.479(2)
γ [°]	90	90	90	90
Volume [Å^3]	3558.4(3)	2035.70(9)	4878.5(3)	1837.94(18)
Z	8	4	8	4
ρ_{calc} [gcm^{-3}]	1.517	1.316	1.469	1.468
μ [mm^{-1}]	2.334	0.089	0.128	2.259
F(000)	1664	856	2224	832
Crystal size [mm^3]	0.03×0.08×0.12	0.081×0.121×0.158	0.07×0.072×0.123	0.17×0.2×0.22
Crystal colour	colourless	colourless	colourless	colourless
Crystal shape	plate	prism	prism	prism
Radiation	Mo K_α (λ=0.71073 Å)	Mo K_α (λ=0.71073 Å)	Mo K_α (λ=0.71073 Å)	Mo K_α (λ=0.71073 Å)
2θ range [°]	5.05 to 53.69 (0.79 Å)	4.50 to 53.54 (0.79 Å)	5.13 to 55.11 (0.77 Å)	4.67 to 53.54 (0.79 Å)
Index ranges	$-18 \le h \le 18$ $-9 \le k \le 9$ $-40 \le l \le 41$	$-32 \le h \le 32$ $-8 \le k \le 8$ $-14 \le l \le 14$	$-58 \le h \le 58$ $-10 \le k \le 10$ $-17 \le l \le 17$	$-13 \le h \le 13$ $-22 \le k \le 22$ $-14 \le l \le 14$
Reflections collected	68712	32548	83911	45740
Independent reflections	7600 R_{int} = 0.0601 R_{sigma} = 0.0330	4313 R_{int} = 0.0372 R_{sigma} = 0.0238	5621 R_{int} = 0.0906 R_{sigma} = 0.0349	3898 R_{int} = 0.0458 R_{sigma} = 0.0242
Completeness to θ = 25.242°	99.6	99.8	99.9	99.7
Data / Restraints / Parameters	7600 / 0 / 457	4313 / 237 / 312	5621 / 0 / 346	3898 / 0 / 229
Absorption correction Tmin/Tmax (method)	0.767 / 0.933 (Multi-Scan)	0.986 / 0.993 (Multi-Scan)	0.984 / 0.991 (Multi-Scan)	0.636 / 0.700 (Multi-Scan)
Goodness-of-fit on F^2	1.237	1.047	1.038	1.200
Final R indexes [I≥2σ(I)]	R_1 = 0.0528 wR_2 = 0.1147	R_1 = 0.0339 wR_2 = 0.0869	R_1 = 0.0382 wR_2 = 0.0862	R_1 = 0.0299 wR_2 = 0.0711

Final R indexes [all data]	R_1 = 0.0568<br>wR_2 = 0.1162	R_1 = 0.0349<br>wR_2 = 0.0882	R_1 = 0.0570<br>wR_2 = 0.0971	R_1 = 0.0308<br>wR_2 = 0.0715
Largest peak/hole [eÅ^{-3}]	0.63/−1.26	0.20/−0.21	0.32/−0.25	0.65/−0.53
Flack X parameter	-	0.4(2)		

Table S8: Crystal data and structure refinement for compounds **5h**, **5ad**, **5ag** and **5ao**:

CCDC number	**5h -** 2502657	**5ad-** 2502655	**5ag -** 2502656	**5ao-** 2502659
Empirical formula	$C_{26}H_{24}F_3NO_4$	$C_{25}H_{25}NO_5$	$C_{24.50}H_{22}ClN_3O_4$	$C_{26}H_{24}ClF_3N_2O_2$
Formula weight	471.46	419.46	457.9	488.92
Temperature [K]	100(2)	100(2)	100(2)	100(2)
Crystal system	monoclinic	triclinic	triclinic	orthorhombic
Space group (number)	$C2/c$ (15)	$P\overline{1}$ (2)	$P\overline{1}$ (2)	$P2_12_12_1$ (19)
a [Å]	20.2516(9)	8.6806(5)	13.2595(19)	8.8611(7)
b [Å]	13.6623(6)	9.8665(5)	13.282(2)	14.1211(9)
c [Å]	18.2134(8)	13.2035(6)	14.433(2)	17.7054(13)
α [°]	90	105.473(2)	112.080(4)	90
β [°]	115.736(2)	93.660(2)	94.222(5)	90
γ [°]	90	101.196(2)	110.403(4)	90
Volume [Å^3]	4539.5(4)	1061.09(10)	2145.5(6)	2215.5(3)
Z	8	2	4	4
ρ_{calc} [gcm^{-3}]	1.38	1.313	1.418	1.466
μ [mm^{-1}]	0.923	0.091	0.217	0.226
F(000)	1968	444	956	1016
Crystal size [mm^3]	0.101×0.135×0.201	0.064×0.161×0.212	0.034×0.071×0.198	0.044×0.285×0.299
Crystal colour	colourless	colourless	colourless	colourless
Crystal shape	prism	prism	plate	plate
Radiation	CuK_α<br>(λ=1.54178 Å)	Mo K_α<br>(λ=0.71073 Å)	Mo K_α<br>(λ=0.71073 Å)	Mo K_α<br>(λ=0.71073 Å)
2θ range [°]	8.08 to 144.26<br>(0.81 Å)	4.39 to 53.64<br>(0.79 Å)	3.81 to 53.46<br>(0.79 Å)	5.14 to 55.03<br>(0.77 Å)
Index ranges	$-24 \le h \le 24$<br>$-16 \le k \le 16$<br>$-22 \le l \le 22$	$-11 \le h \le 10$<br>$-12 \le k \le 12$<br>$-16 \le l \le 16$	$-15 \le h \le 16$<br>$-16 \le k \le 16$<br>$-18 \le l \le 18$	$-11 \le h \le 11$<br>$-18 \le k \le 17$<br>$-23 \le l \le 22$
Reflections collected	72720	36370	46440	29999
Independent reflections	4441<br>R_{int} = 0.0355<br>R_{sigma} = 0.0201	4525<br>R_{int} = 0.0621<br>R_{sigma} = 0.0365	9032<br>R_{int} = 0.0862<br>R_{sigma} = 0.0728	5106<br>R_{int} = 0.0802<br>R_{sigma} = 0.0532
Completeness to θ = 25.242°	99.7	99.9	99.2	99.9
Data / Restraints / Parameters	4441 / 431 / 481	4525 / 0 / 283	9032 / 49 / 600	5106 / 1 / 313

Absorption correction Tmin/Tmax (method)	0.836 / 0.913 (Multi-Scan)	0.981 / 0.994 (Multi-Scan)	0.958 / 0.993 (Multi-Scan)	0.936 / 0.990 (Multi-Scan)
Goodness-of-fit on F^2	1.061	1.042	1.015	1.027
Final R indexes [I≥2σ(I)]	R_1 = 0.0410 wR_2 = 0.1029	R_1 = 0.0368 wR_2 = 0.0919	R_1 = 0.0547 wR_2 = 0.1243	R_1 = 0.0413 wR_2 = 0.0936
Final R indexes [all data]	R_1 = 0.0418 wR_2 = 0.1034	R_1 = 0.0429 wR_2 = 0.0971	R_1 = 0.0968 wR_2 = 0.1458	R_1 = 0.0545 wR_2 = 0.1012
Largest peak/hole [eÅ^{-3}]	0.32/−0.21	0.31/−0.22	0.63/−0.72	0.24/−0.42
Flack X parameter	-	-	-	-0.06(4)

SPECTROSCOPIC DATA

^{1}H NMR (400 MHz, $CDCl_3$) of **S1**

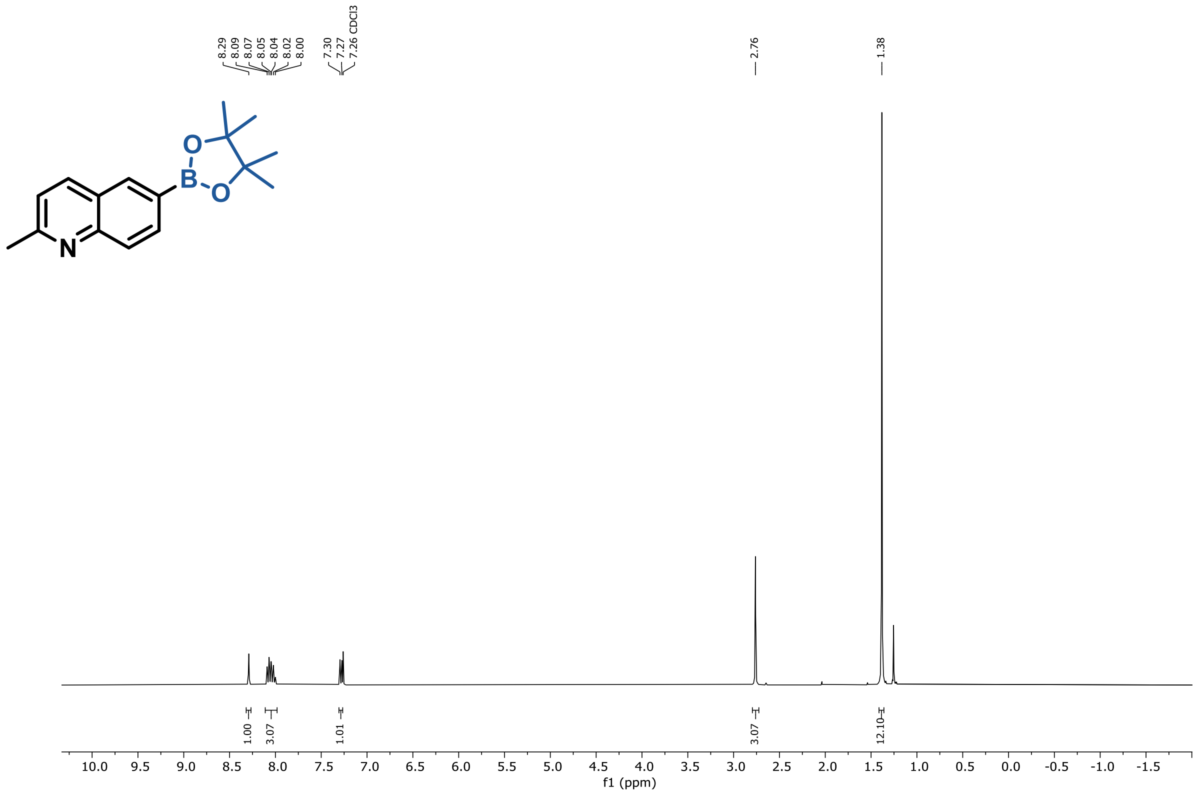

^{13}C NMR (101 MHz, $CDCl_3$) of **S1**

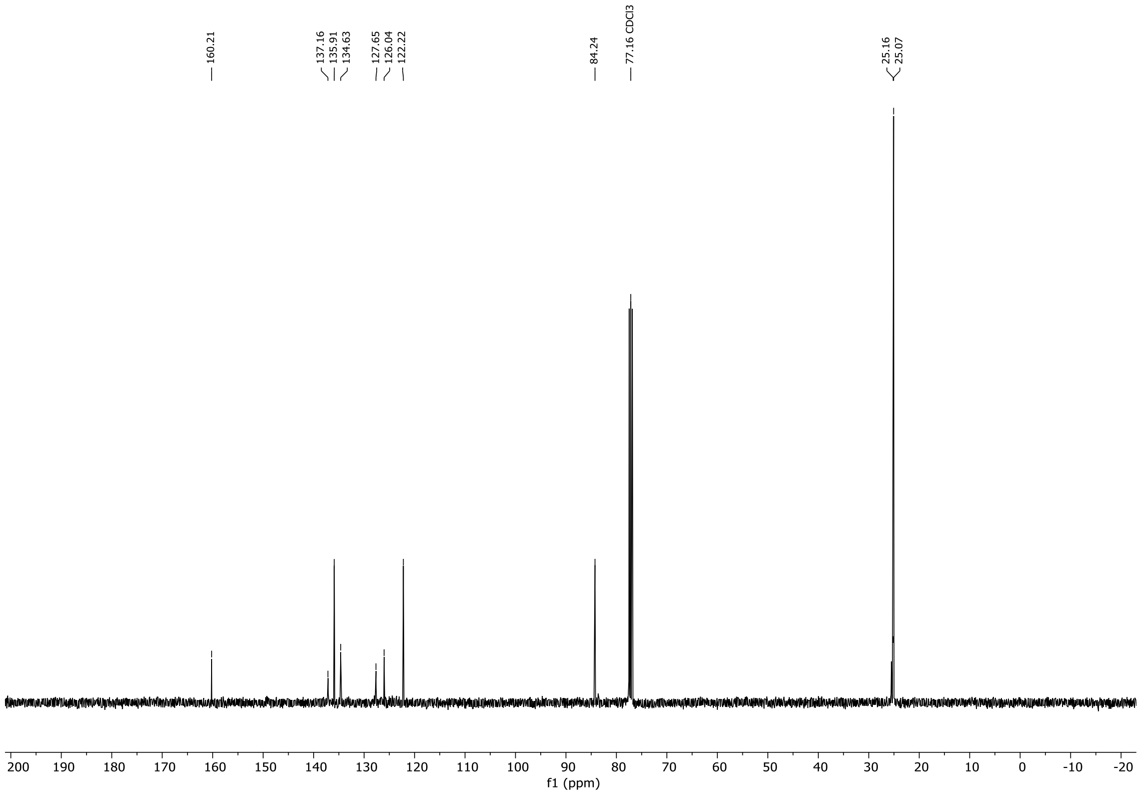

^{11}B NMR (128 MHz, $CDCl_3$) of **S1**

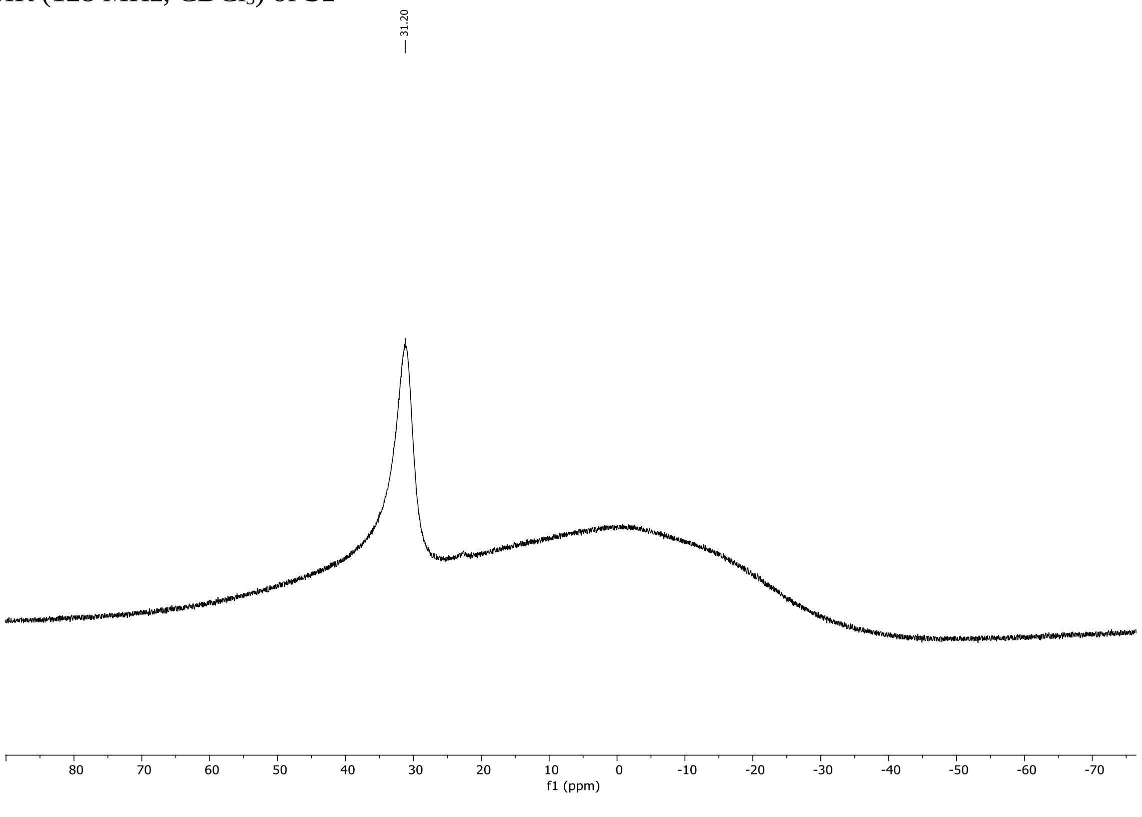

^{1}H NMR (400 MHz, $CDCl_3$) of **1b**

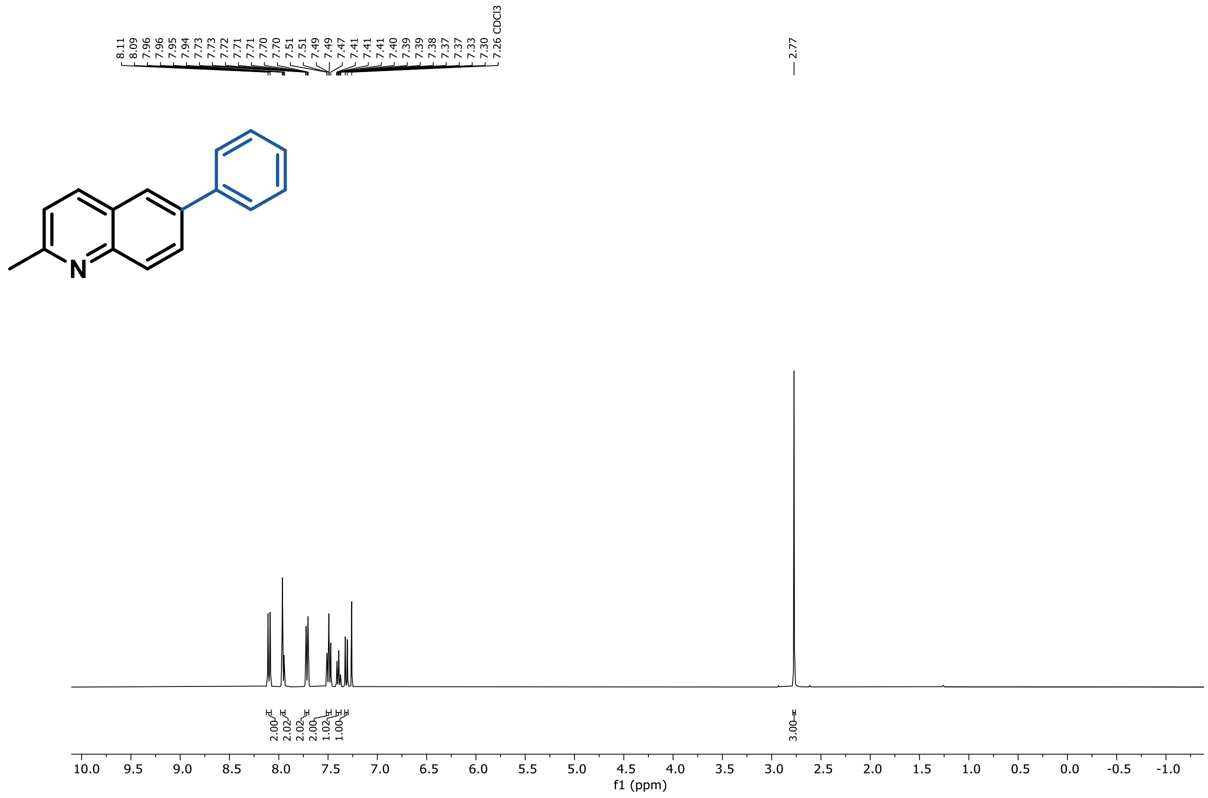

^{13}C NMR (101 MHz, $CDCl_3$) of **1b**

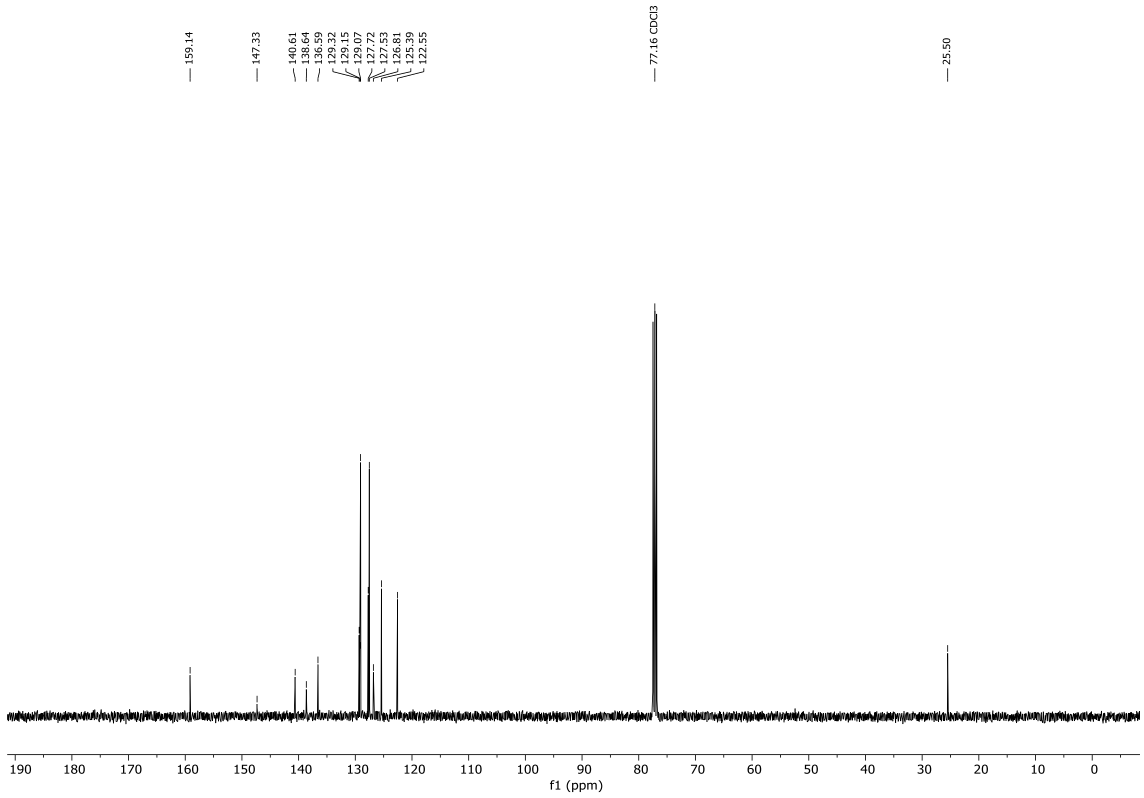

^{1}H NMR (400 MHz, $CDCl_3$) of **1e**

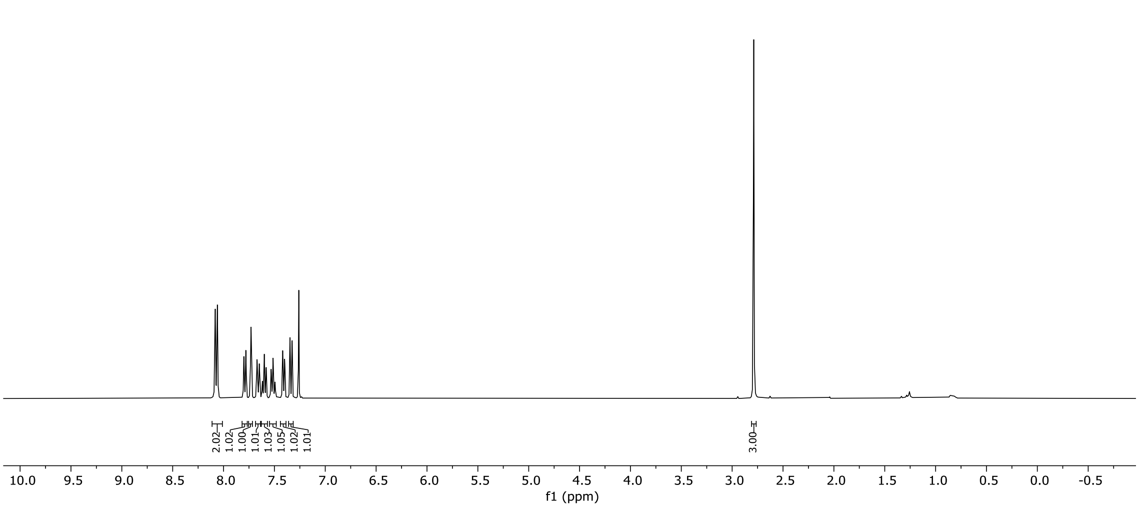

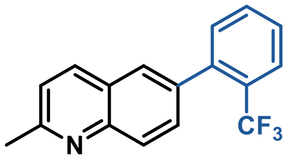

$^{13}C\{^{19}F\}$NMR (126 MHz, $CDCl_3$) of **1e**

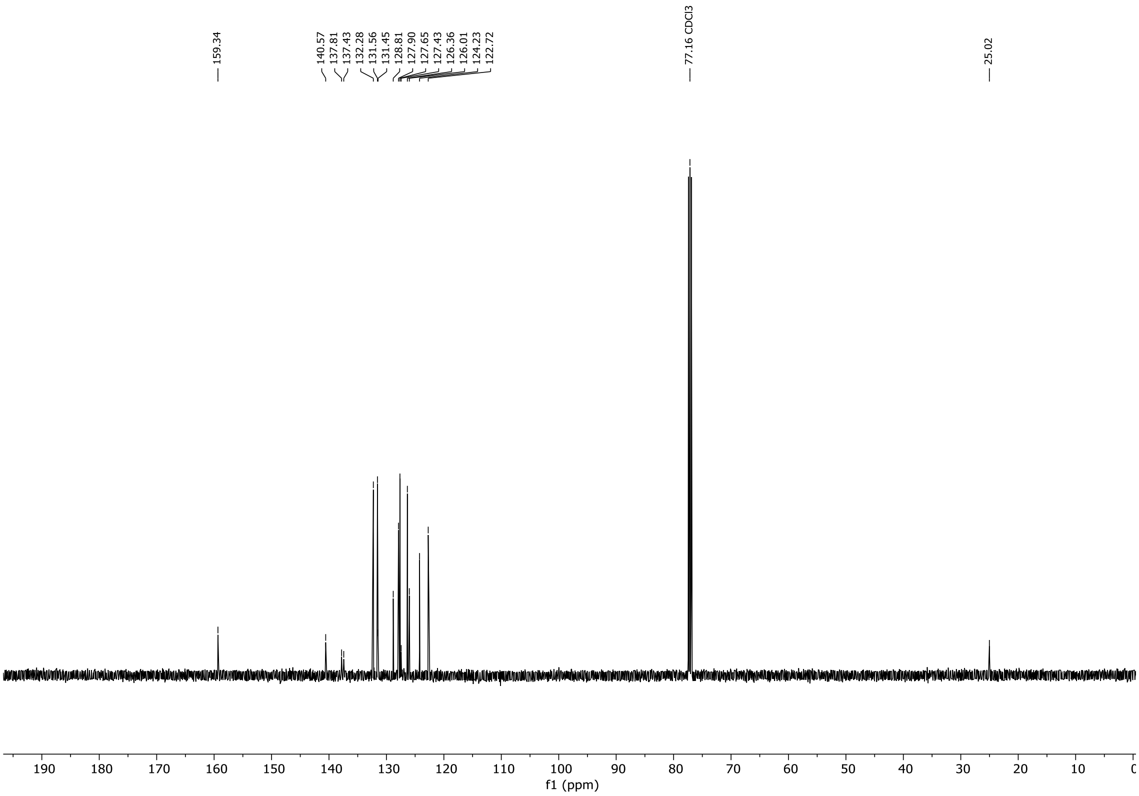

^{19}F NMR (376 MHz, $CDCl_3$) **1e**

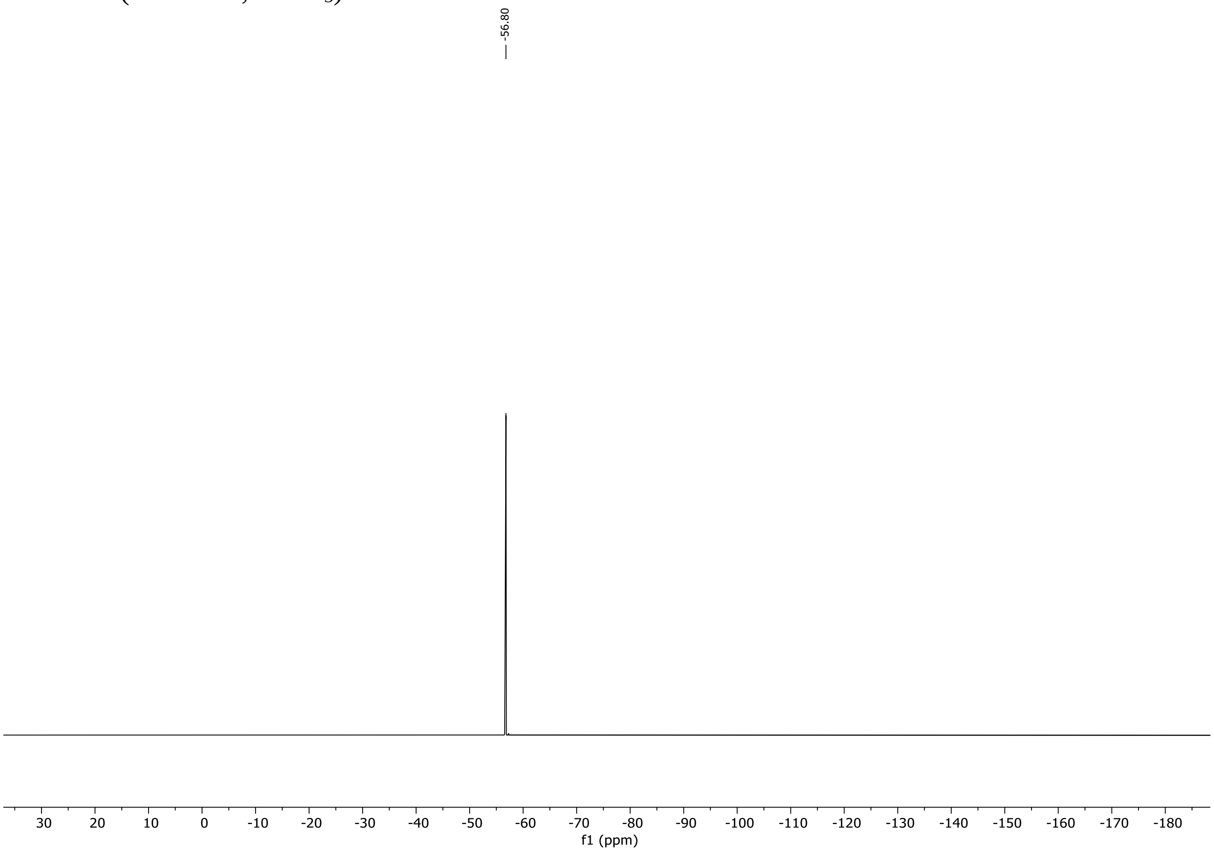

^{1}H NMR (400 MHz, $CDCl_3$) of **1f**

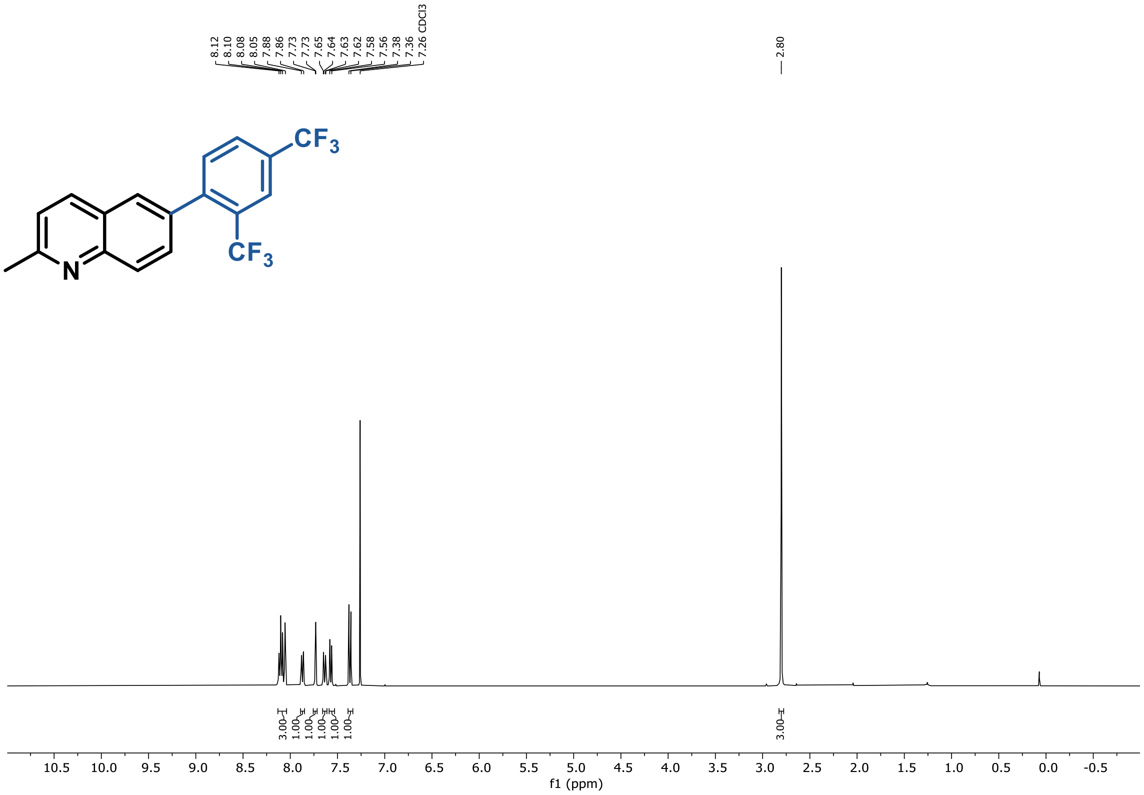

$^{13}C\{^{19}F\}$ NMR (126 MHz, $CDCl_3$) of **1f**

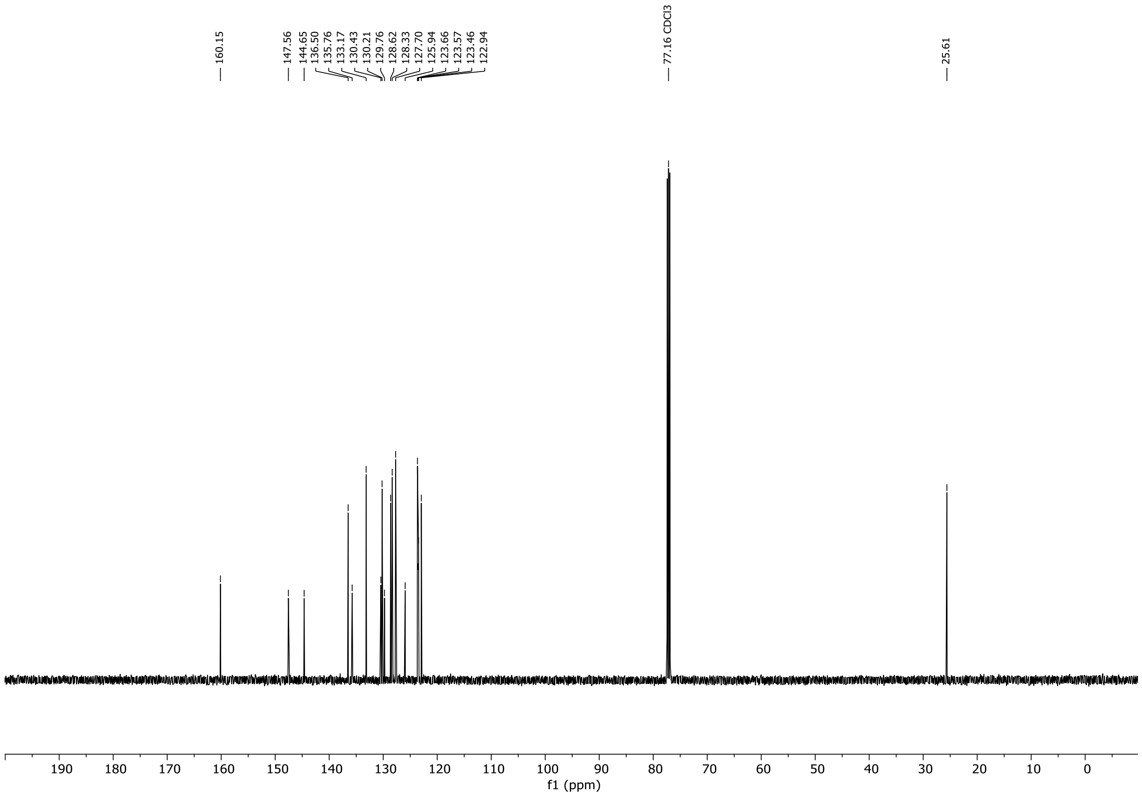

^{19}F NMR (376 MHz, $CDCl_3$) of **1f**

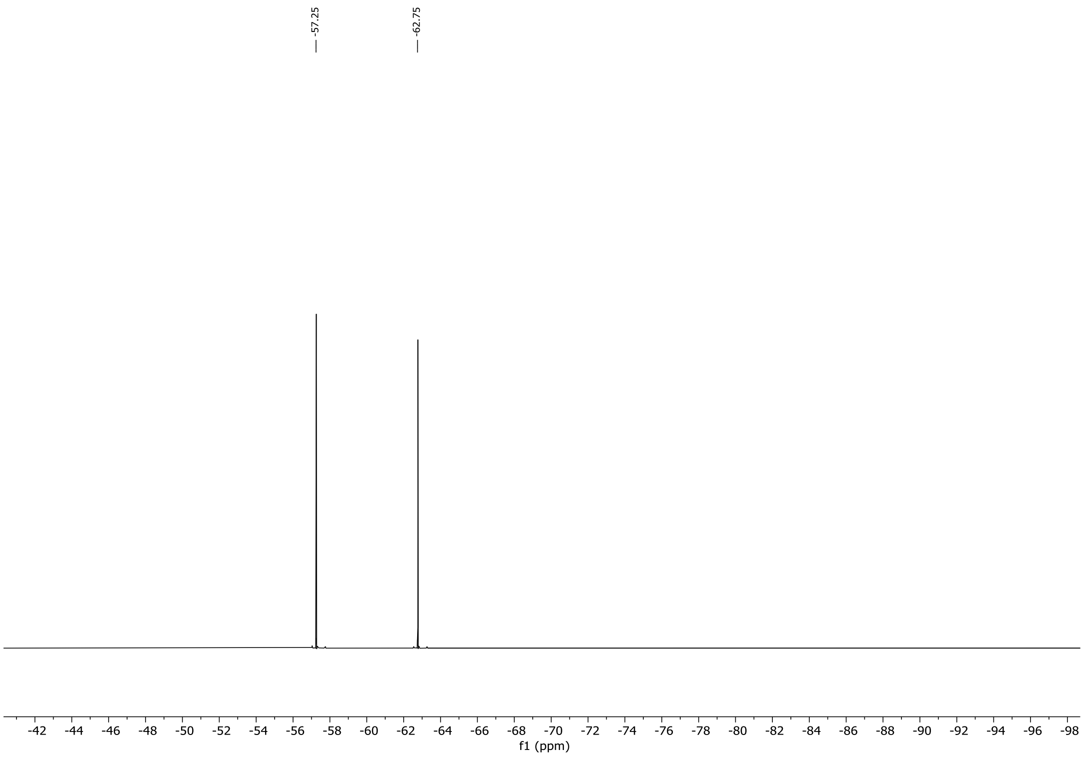

^{1}H NMR (400 MHz, $CDCl_3$) of **1g**

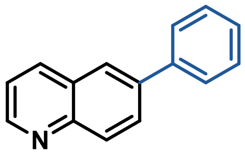

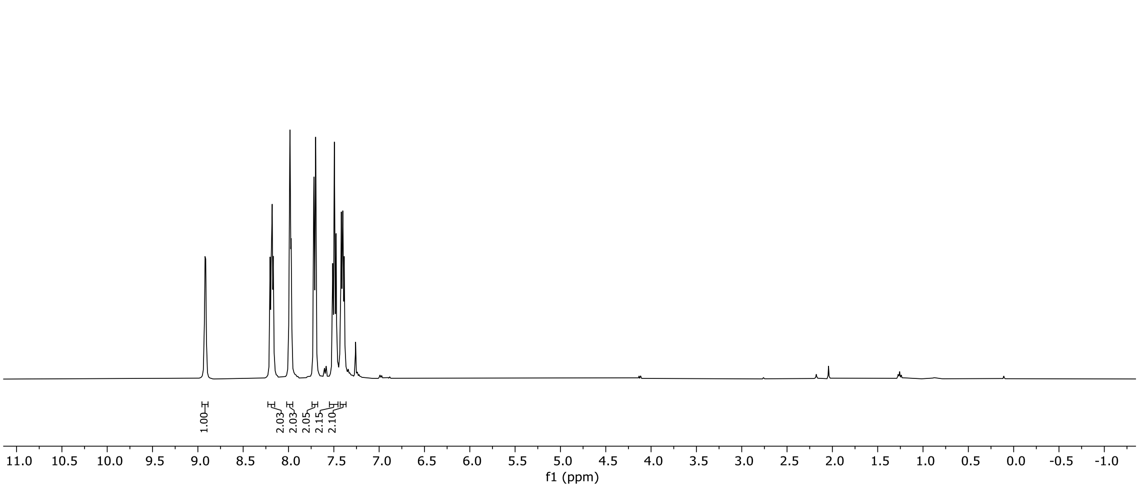

^{13}C NMR (101 MHz, $CDCl_3$) of **1g**

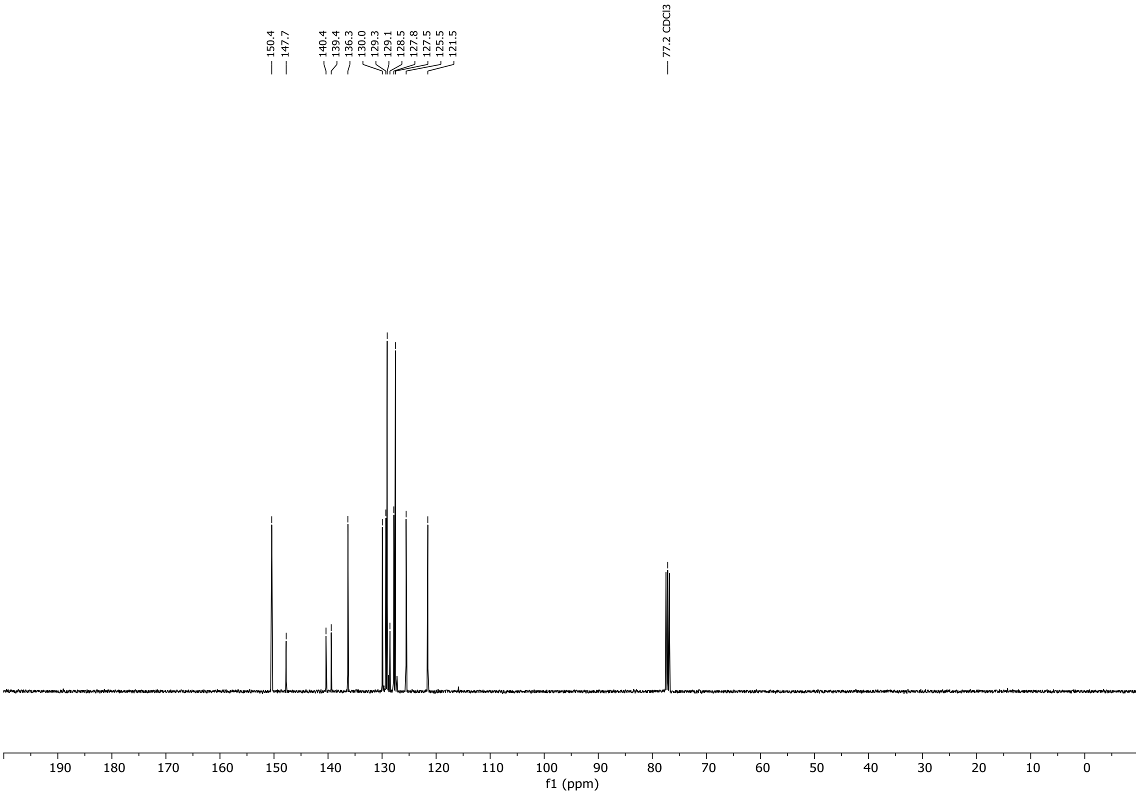

^{1}H NMR (400 MHz, $CDCl_3$) of **1h**

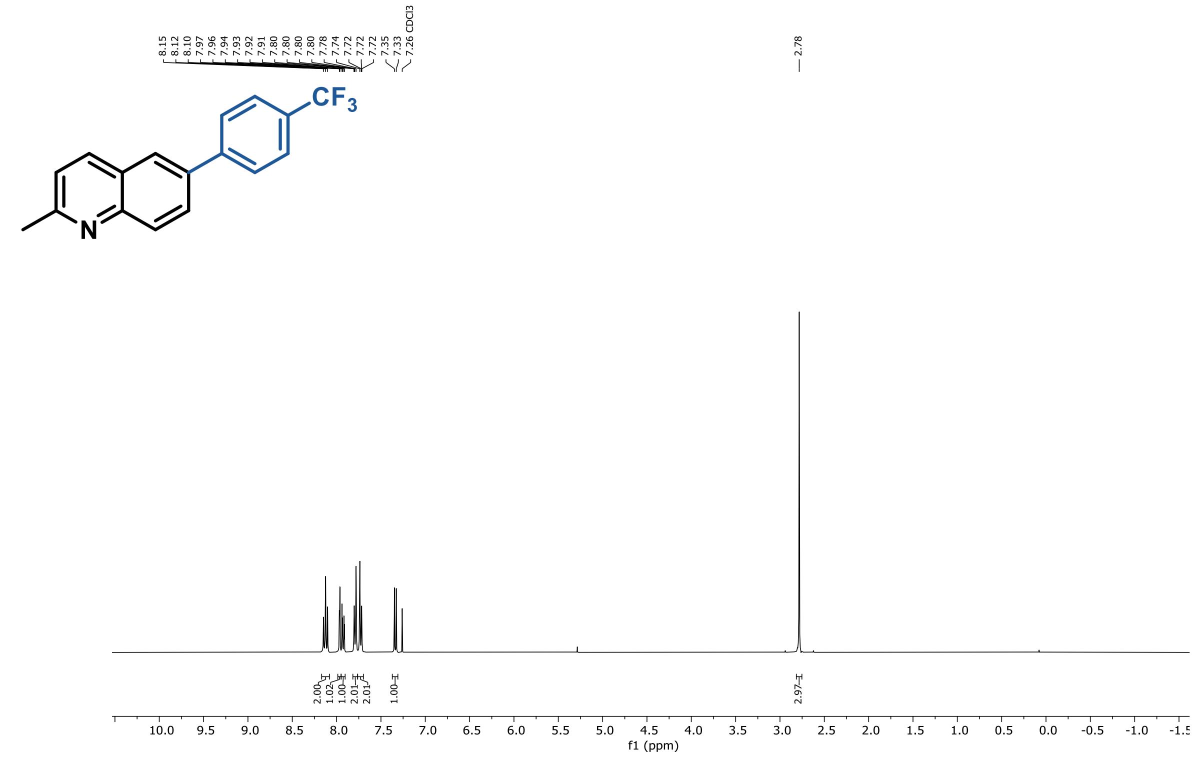

$^{13}C\{^{19}F\}$ NMR (126 MHz, $CDCl_3$) of **1h**

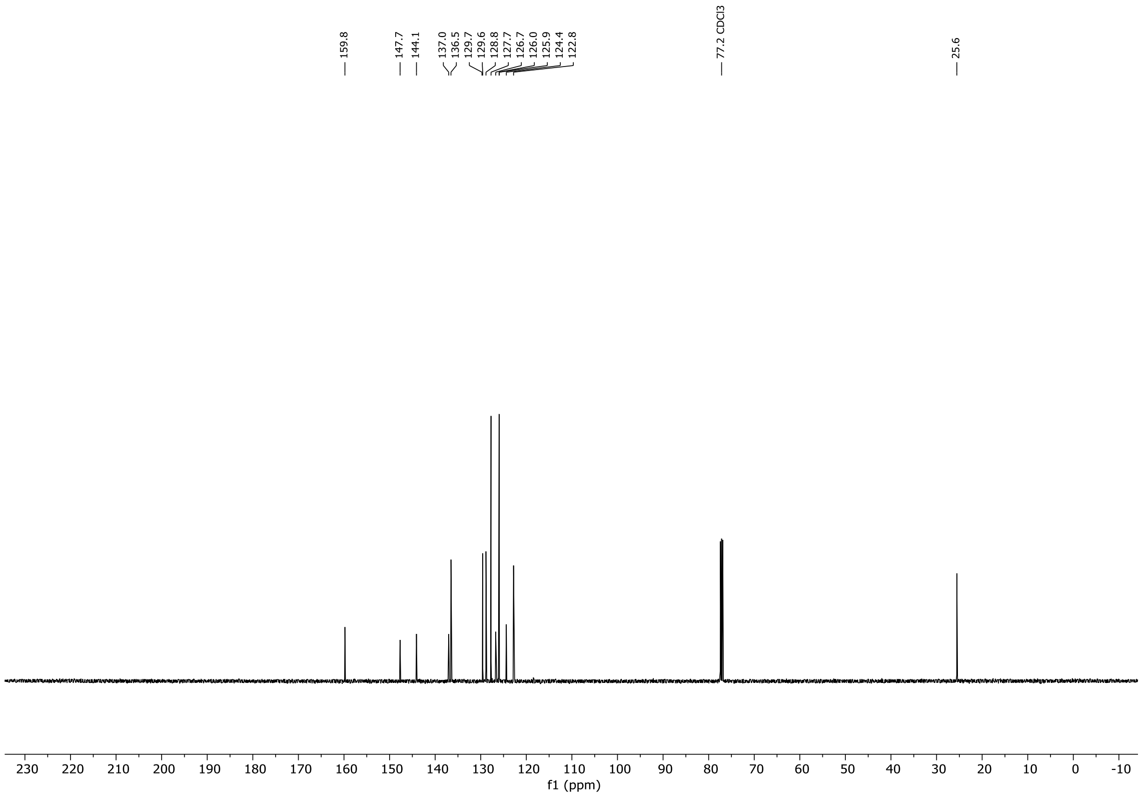

^{19}F NMR (376 MHz, $CDCl_3$) of **1h**

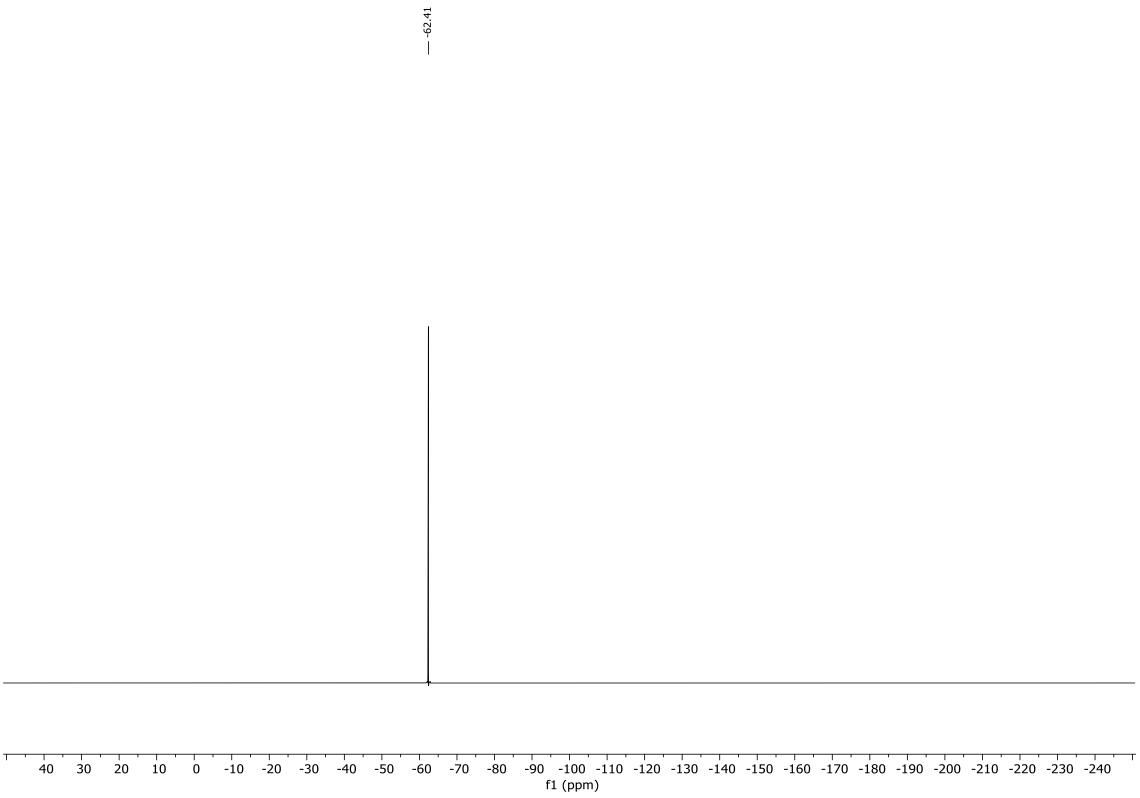

^{1}H NMR (400 MHz, $CDCl_3$) of **1i**

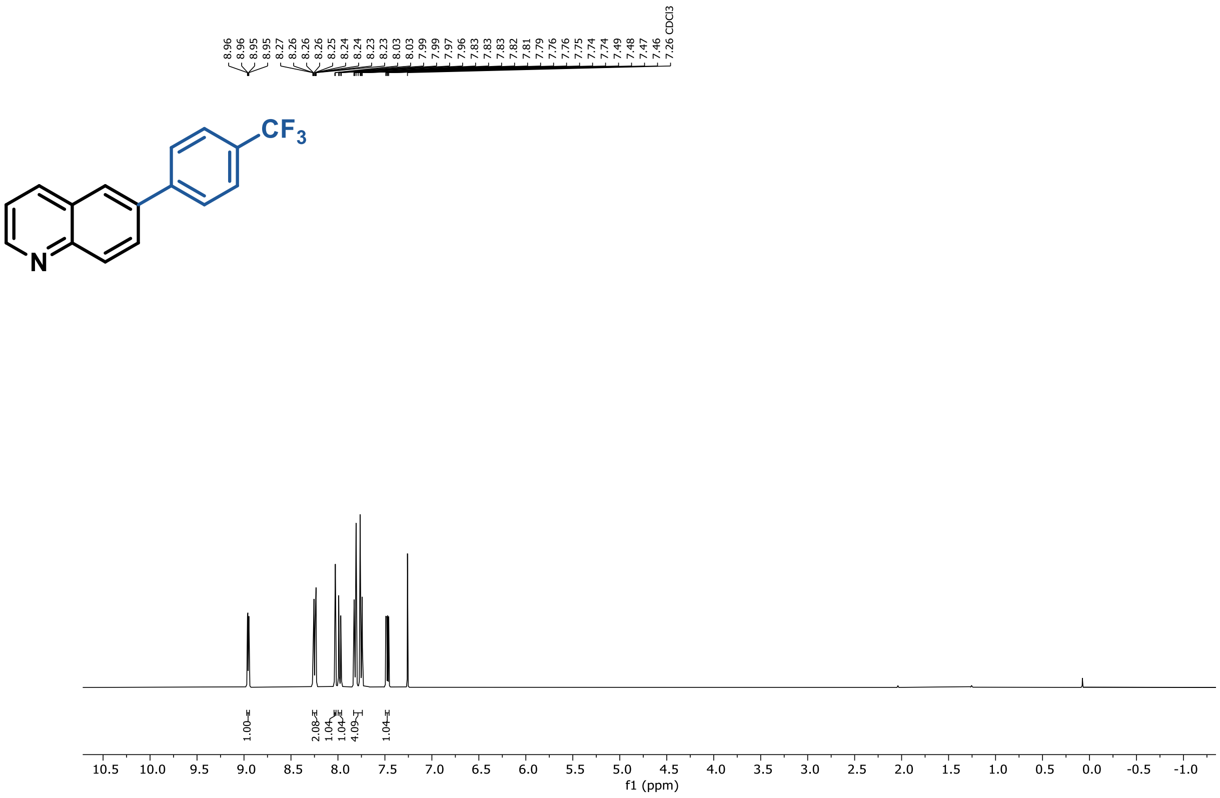

$^{13}C\{^{19}F\}$ NMR (126 MHz, $CDCl_3$) of **1i**

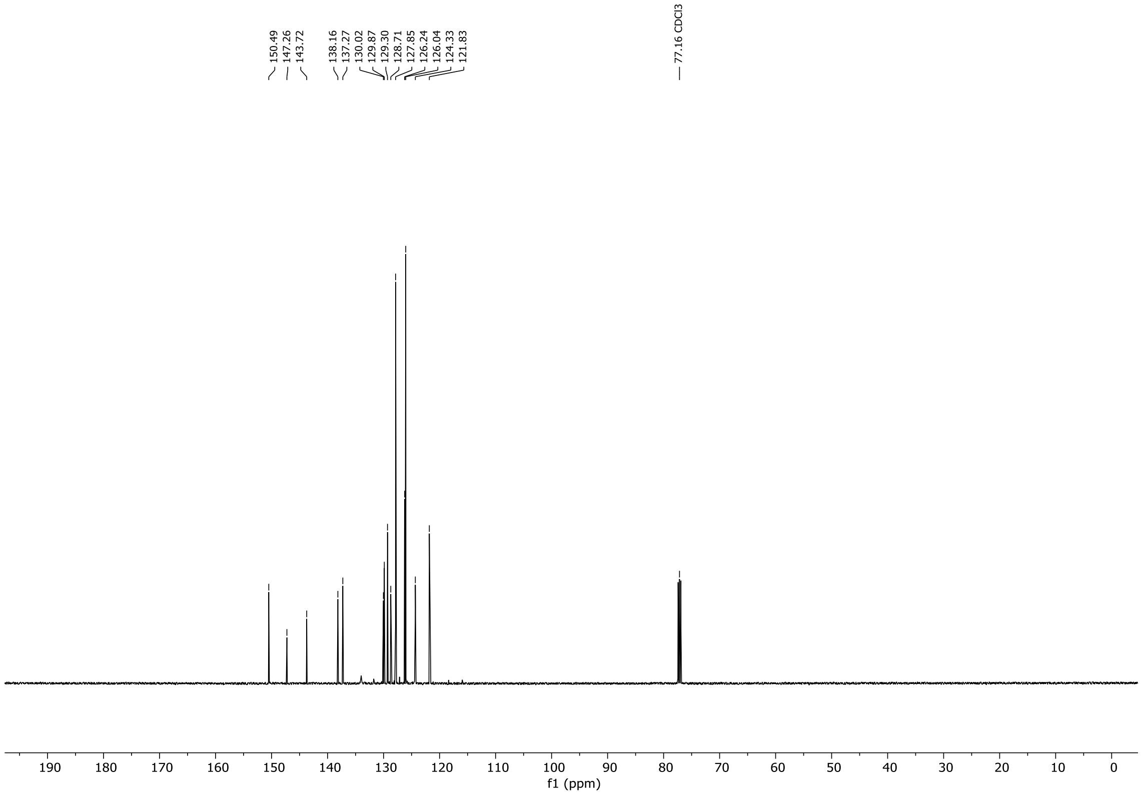

^{19}F NMR (470 MHz, $CDCl_3$) of **1i**

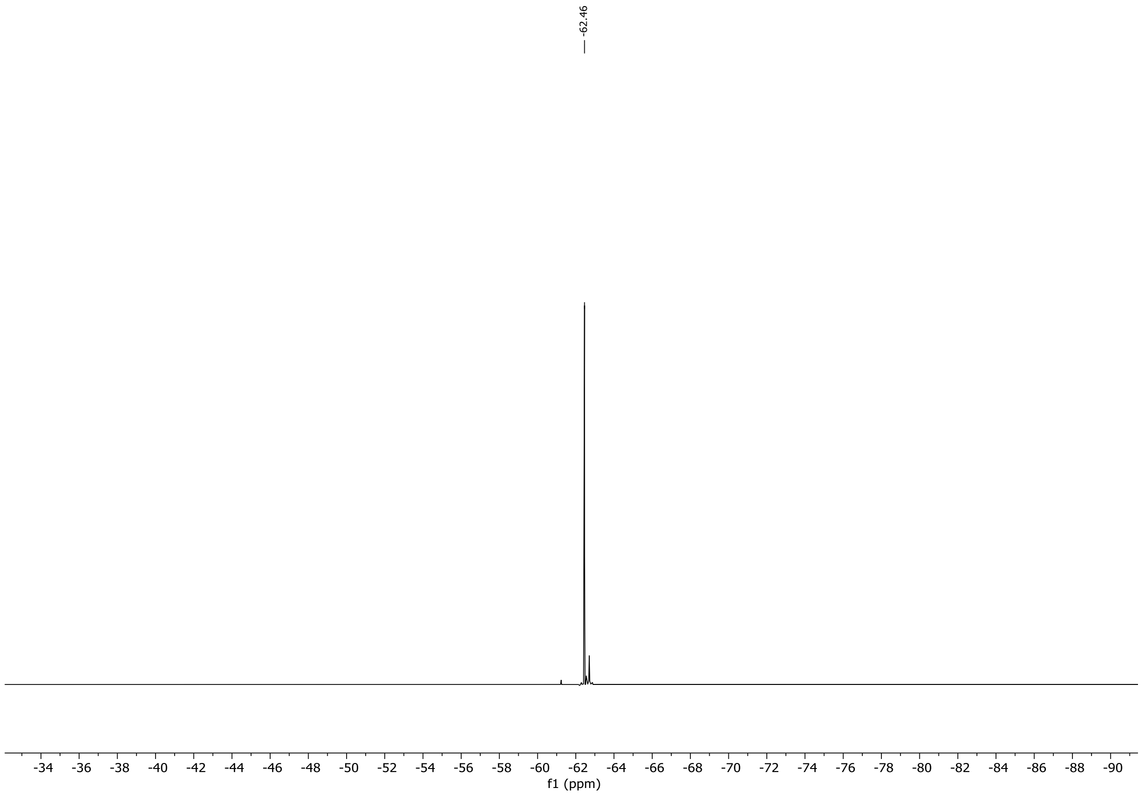

^{1}H NMR (400 MHz, $CDCl_3$) of **1j**

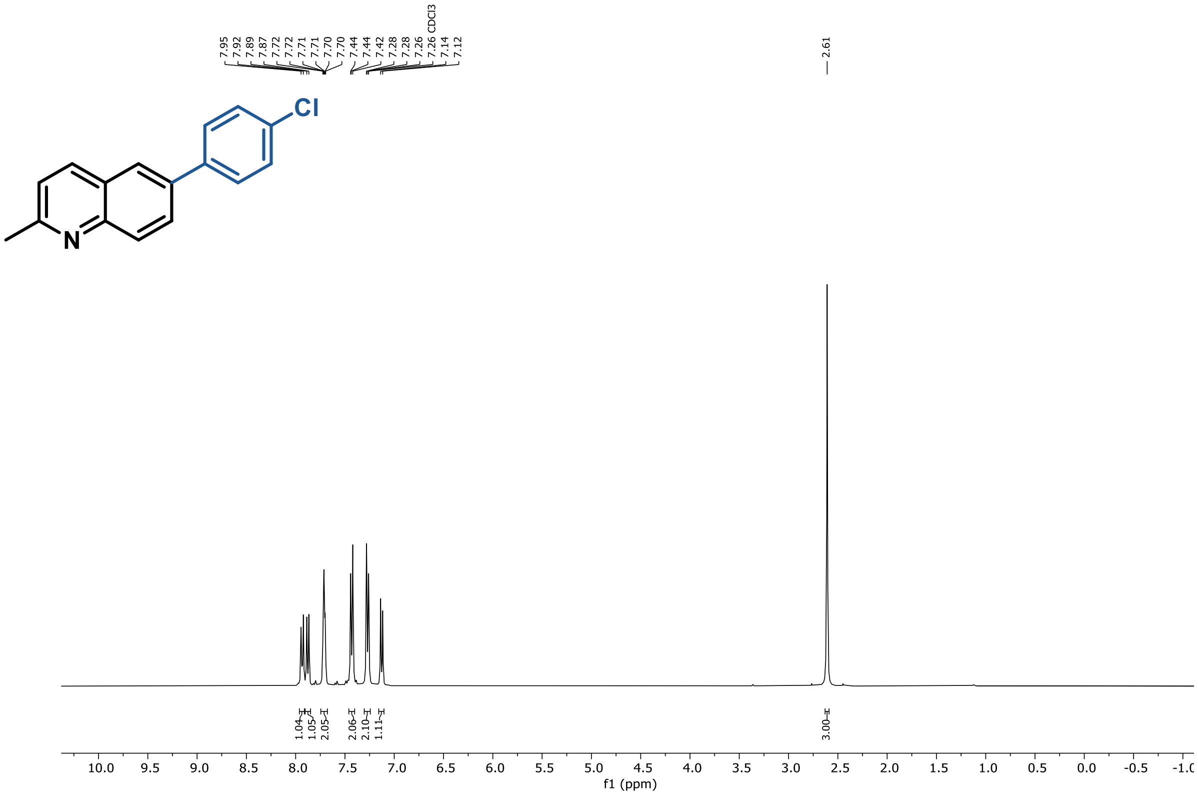

^{13}C NMR (101 MHz, $CDCl_3$) of **1j**

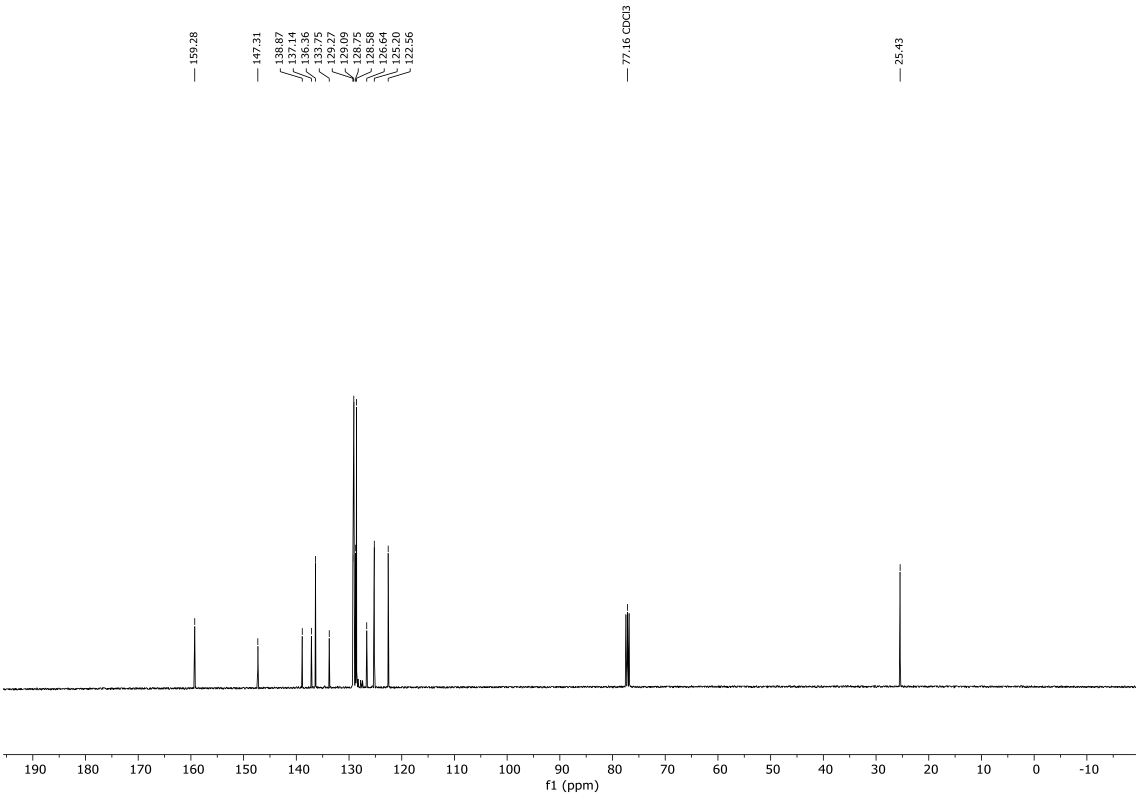

^{1}H NMR (400 MHz, $CDCl_3$) of **1k**

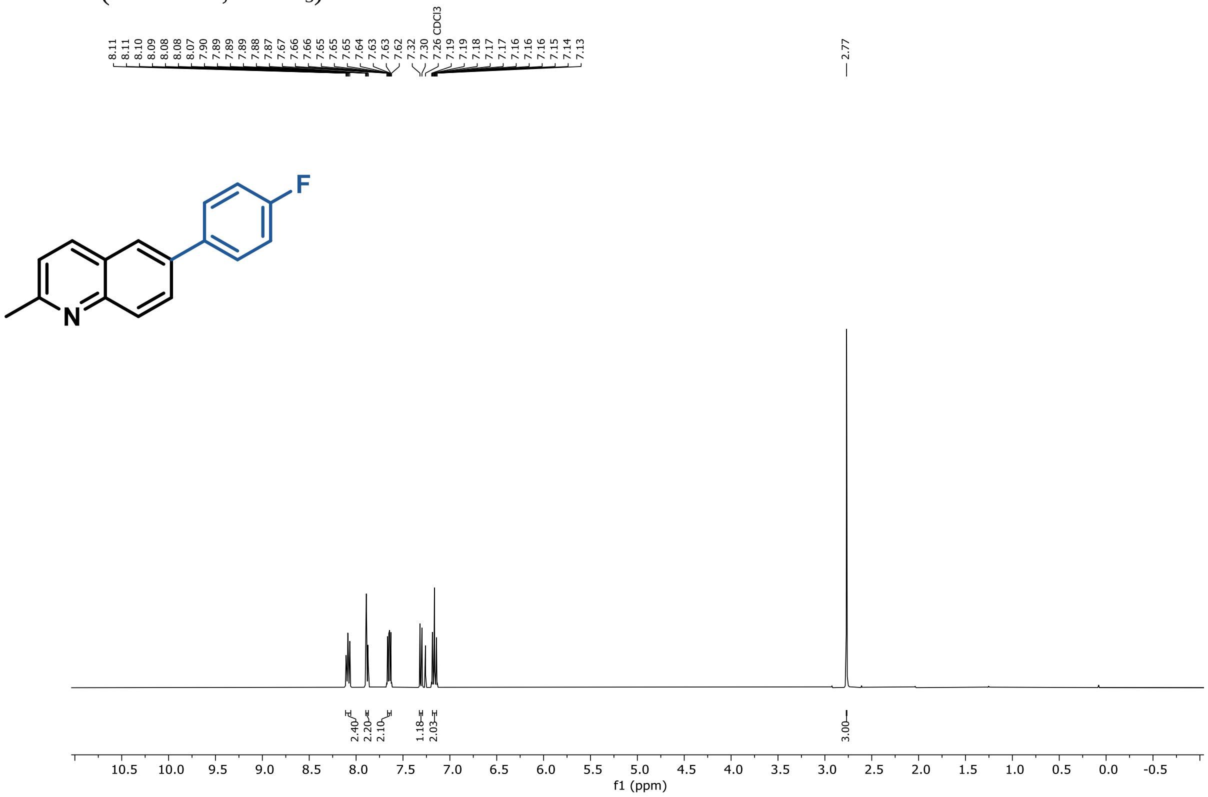

^{13}C{^{19}F} NMR (126 MHz, $CDCl_3$) of **1k**

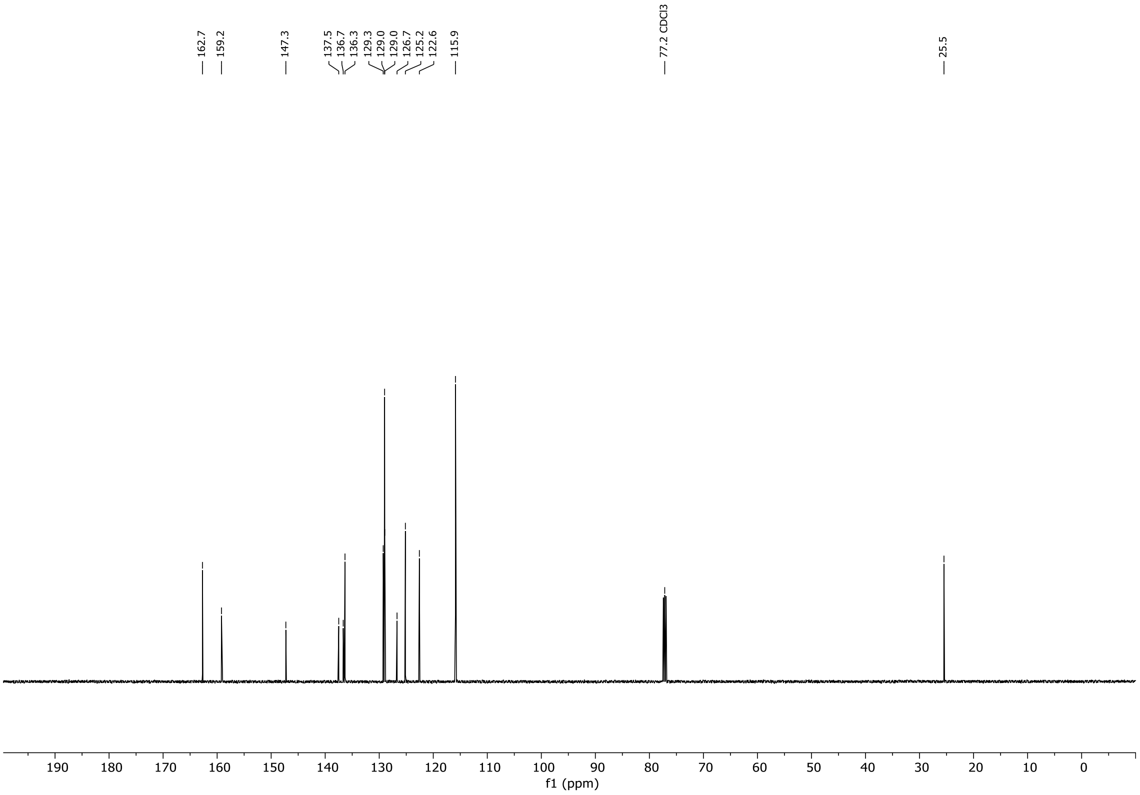

^{19}F NMR (376 MHz, $CDCl_3$) of **1k**

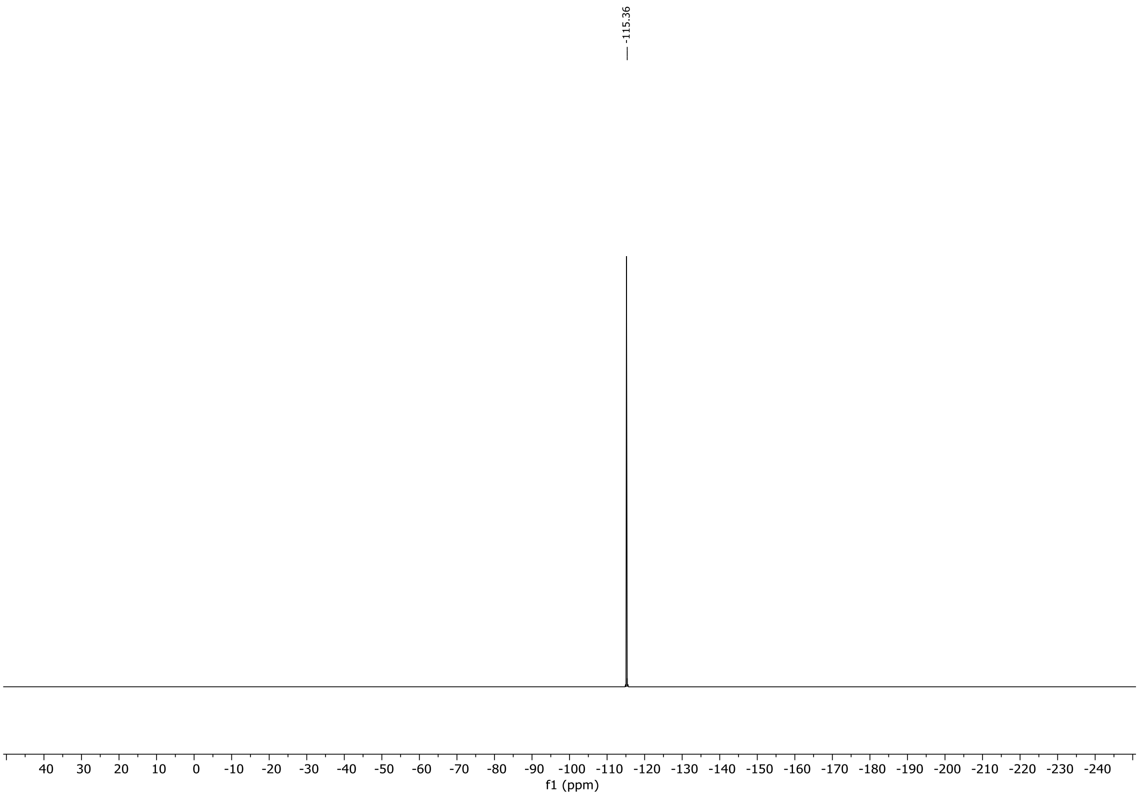

^{1}H NMR (400 MHz, $CDCl_3$) of **1l**

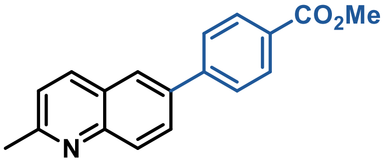

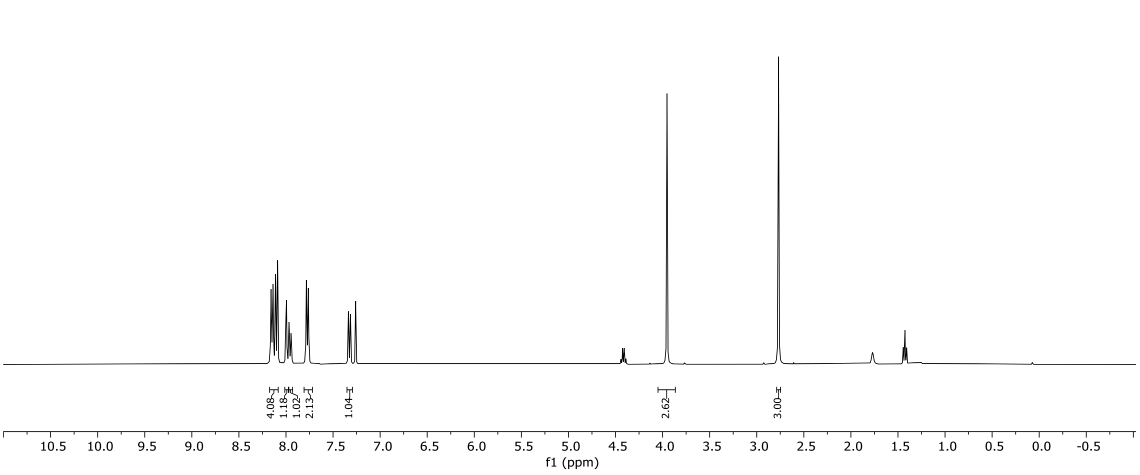

^{13}C NMR (101 MHz, $CDCl_3$) of **1l**

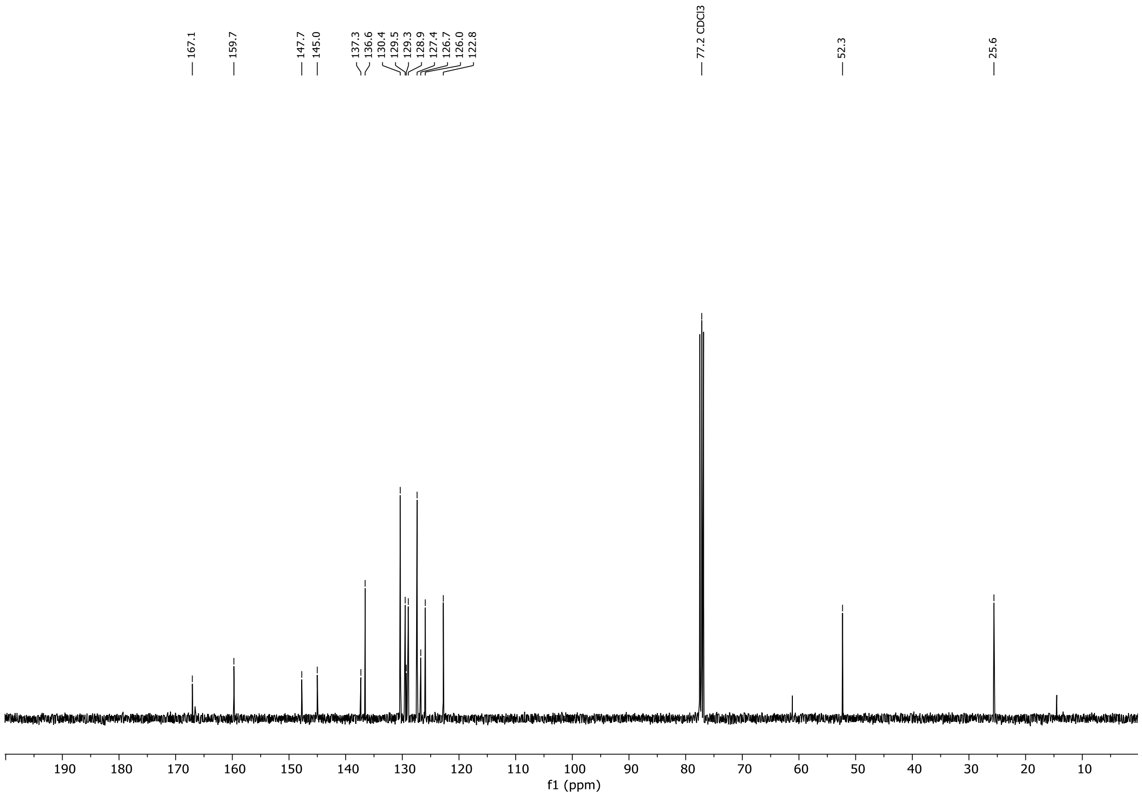

^{1}H NMR (400 MHz, $CDCl_3$) of **1m**

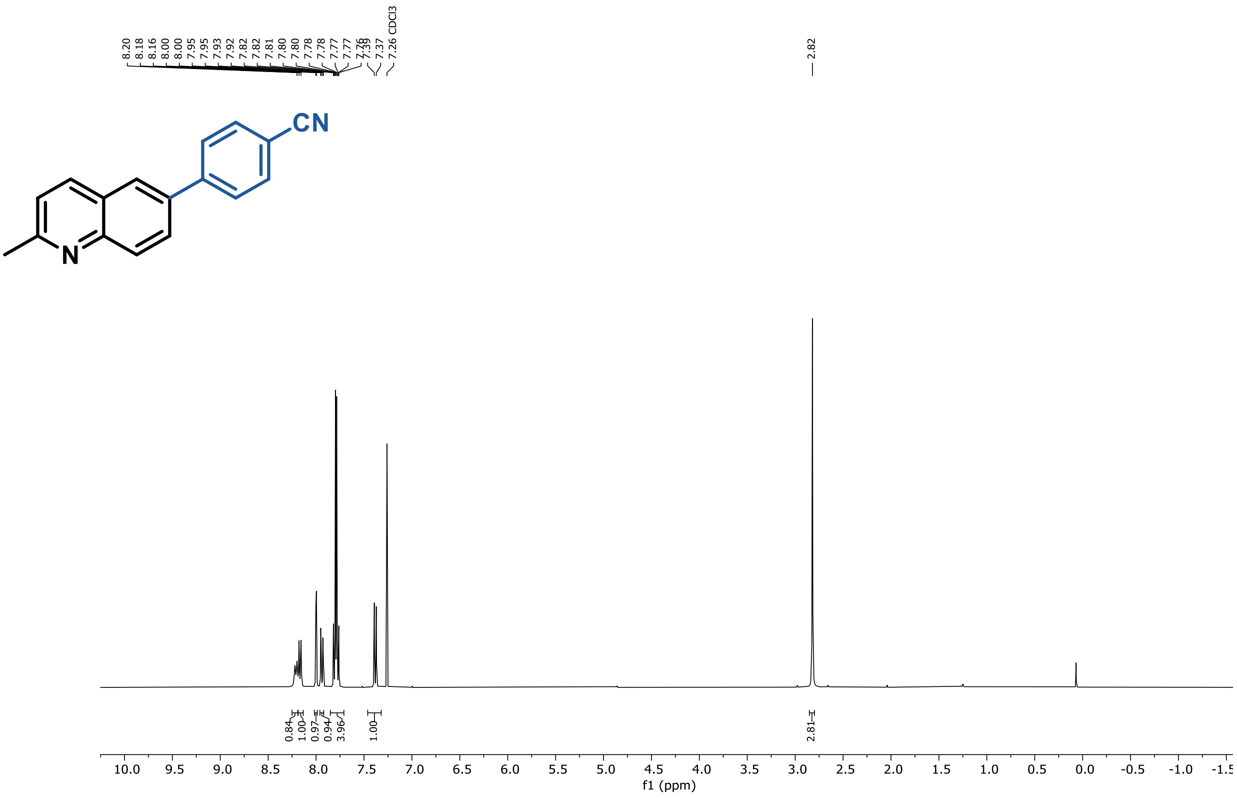

^{13}C NMR (101 MHz, CDCl$_3$) of **1m**

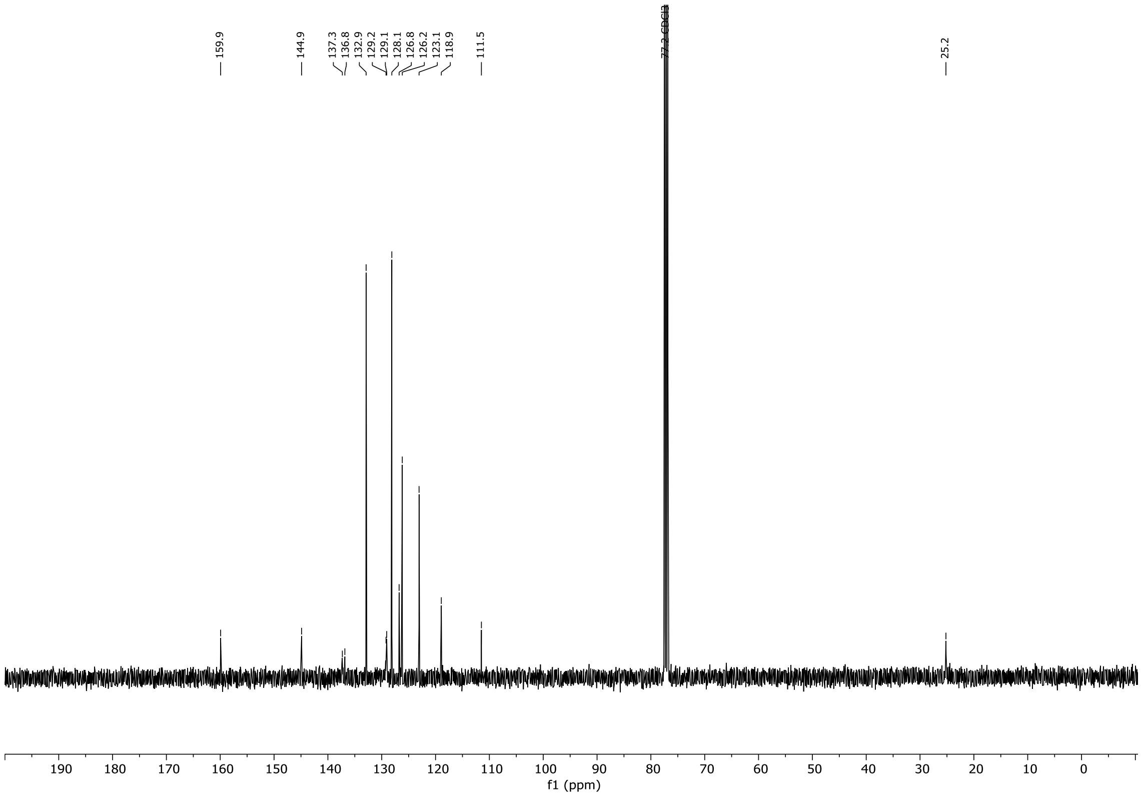

^{1}H NMR (400 MHz, CDCl$_3$) of **1n**

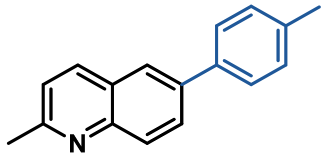

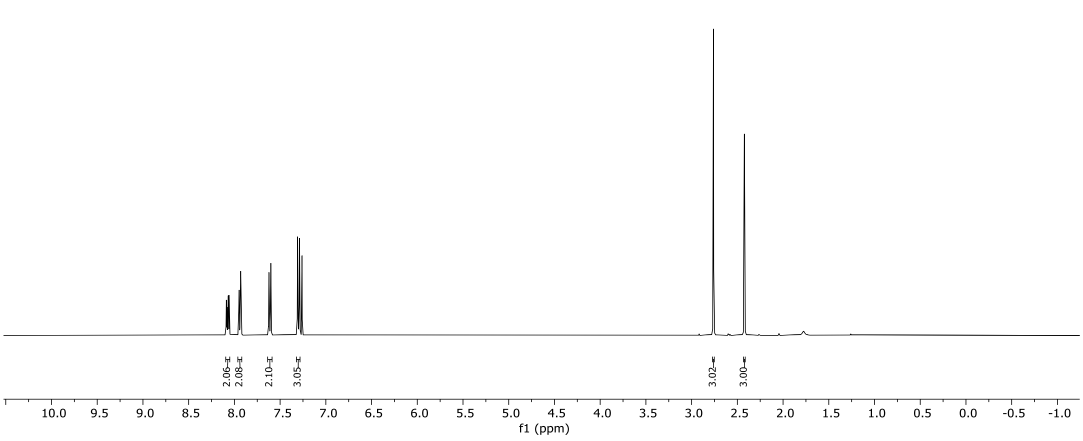

^{13}C NMR (101 MHz, $CDCl_3$) of **1n**

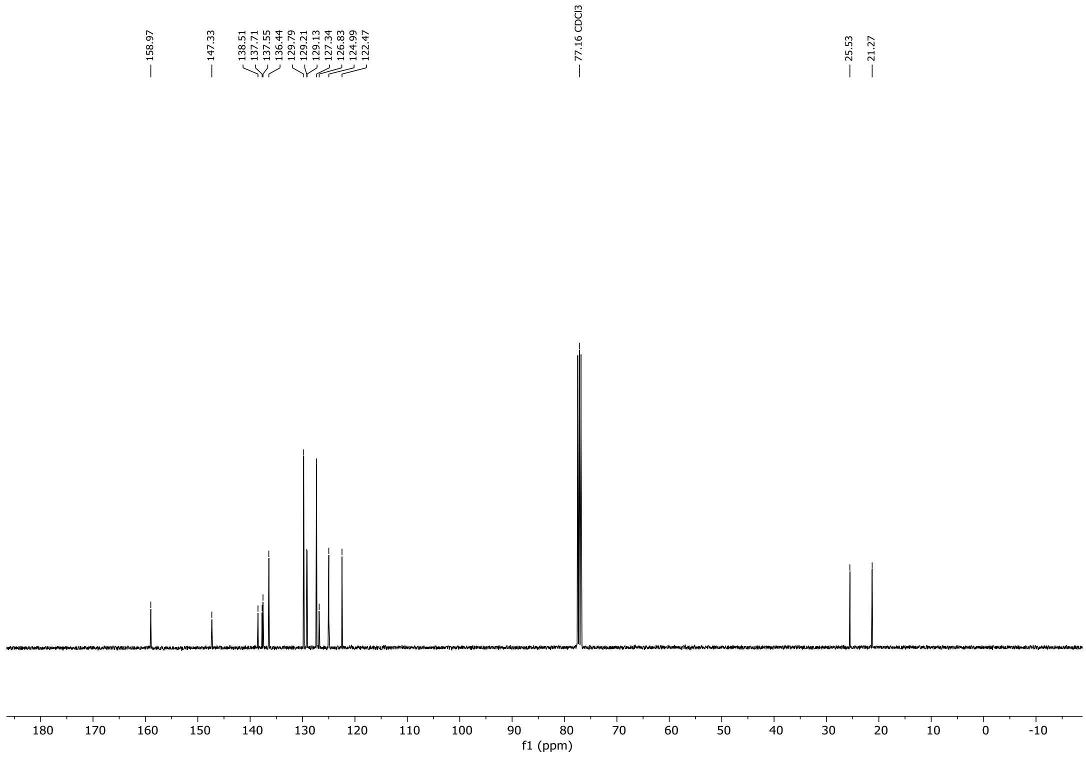

^{1}H NMR (400 MHz, $CDCl_3$) of **1o**

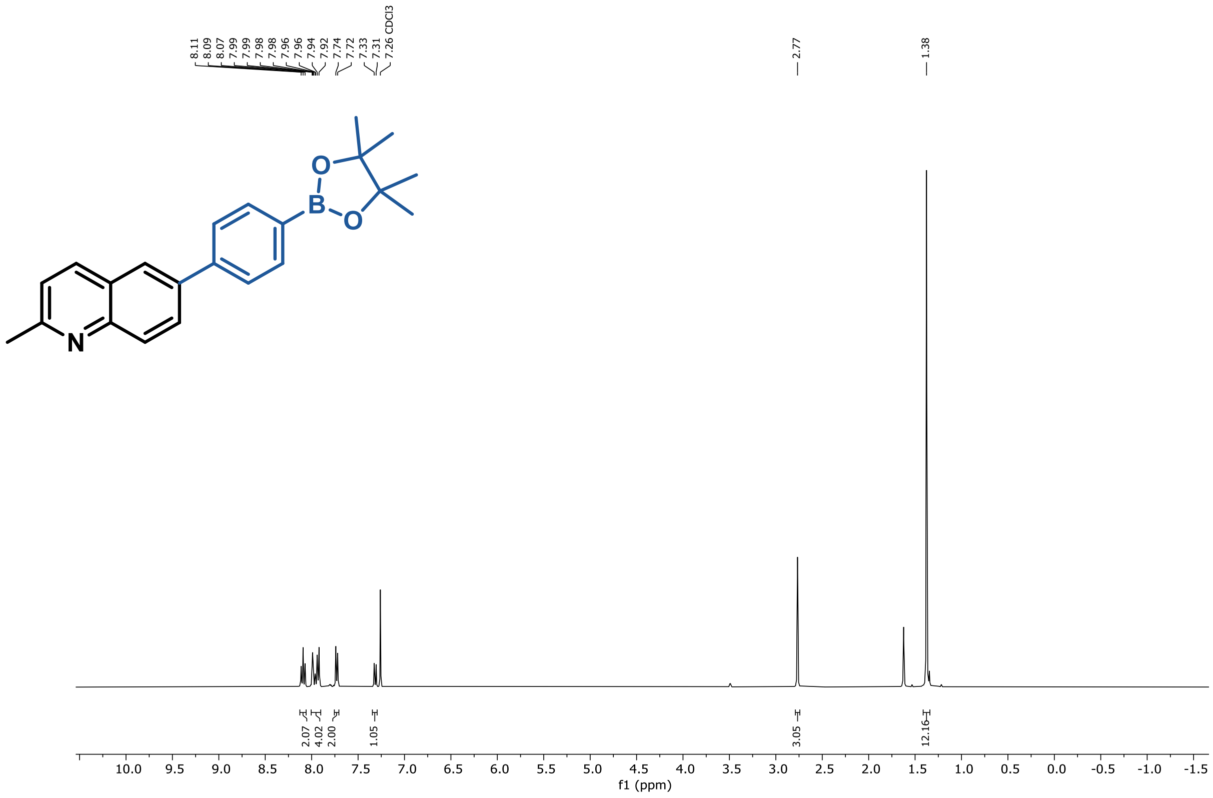

^{13}C NMR (101 MHz, $CDCl_3$) of **1o**

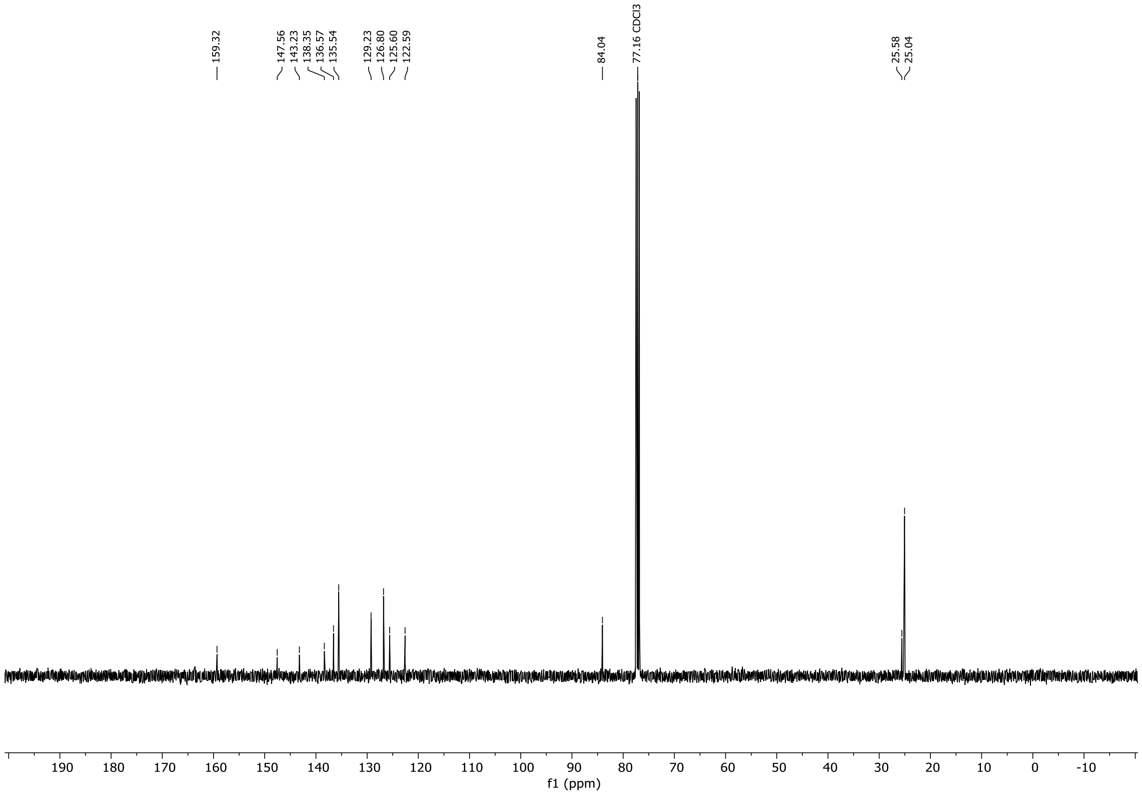

^{11}B NMR (128 MHz, $CDCl_3$) of **1o**

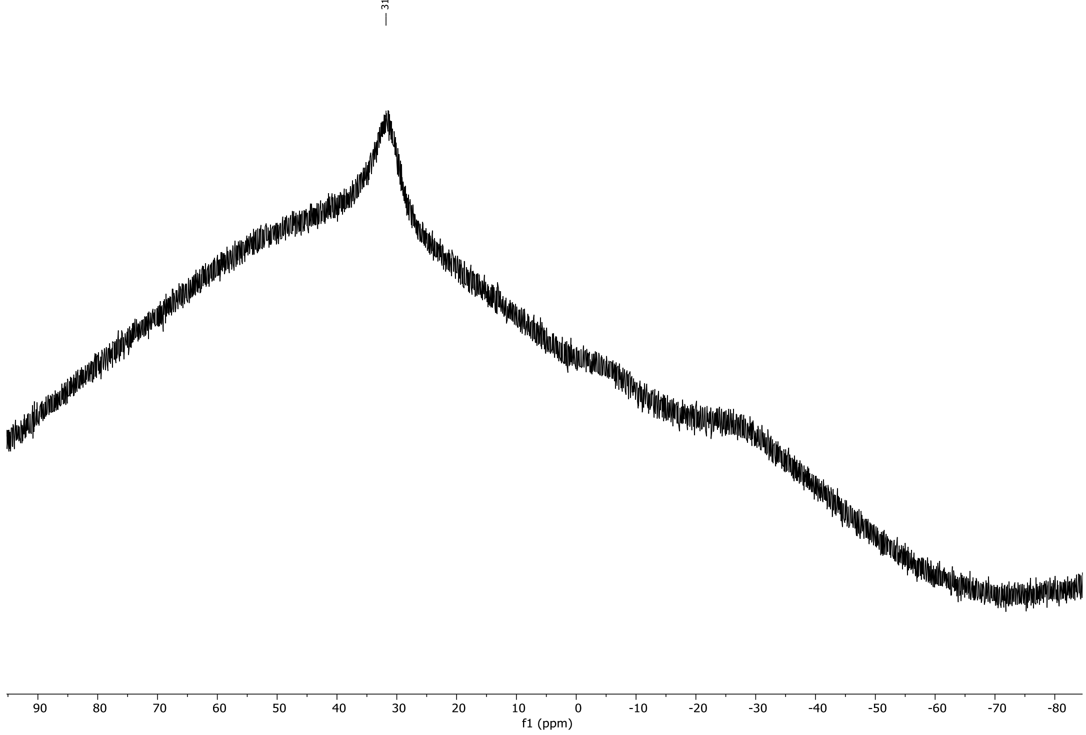

^{1}H NMR (400 MHz, $CDCl_3$) of **1p**

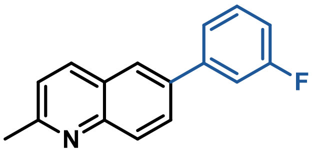

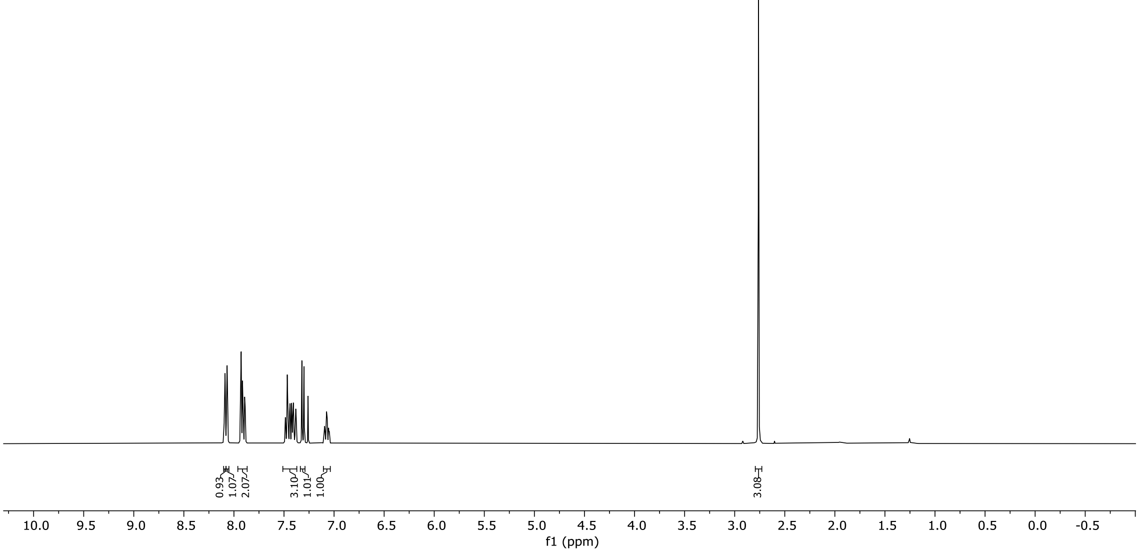

^{13}C{^{19}F} NMR (126 MHz, $CDCl_3$) of **1p**

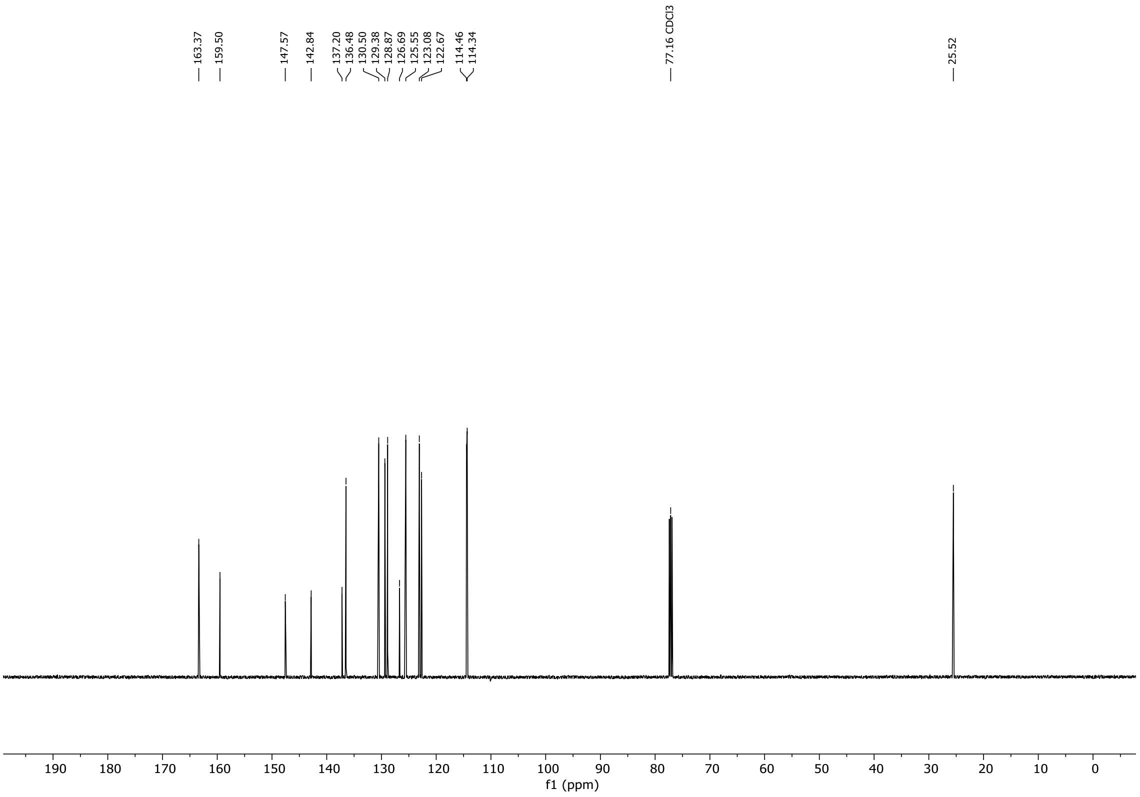

^{19}F NMR (376 MHz, $CDCl_3$) **1p**

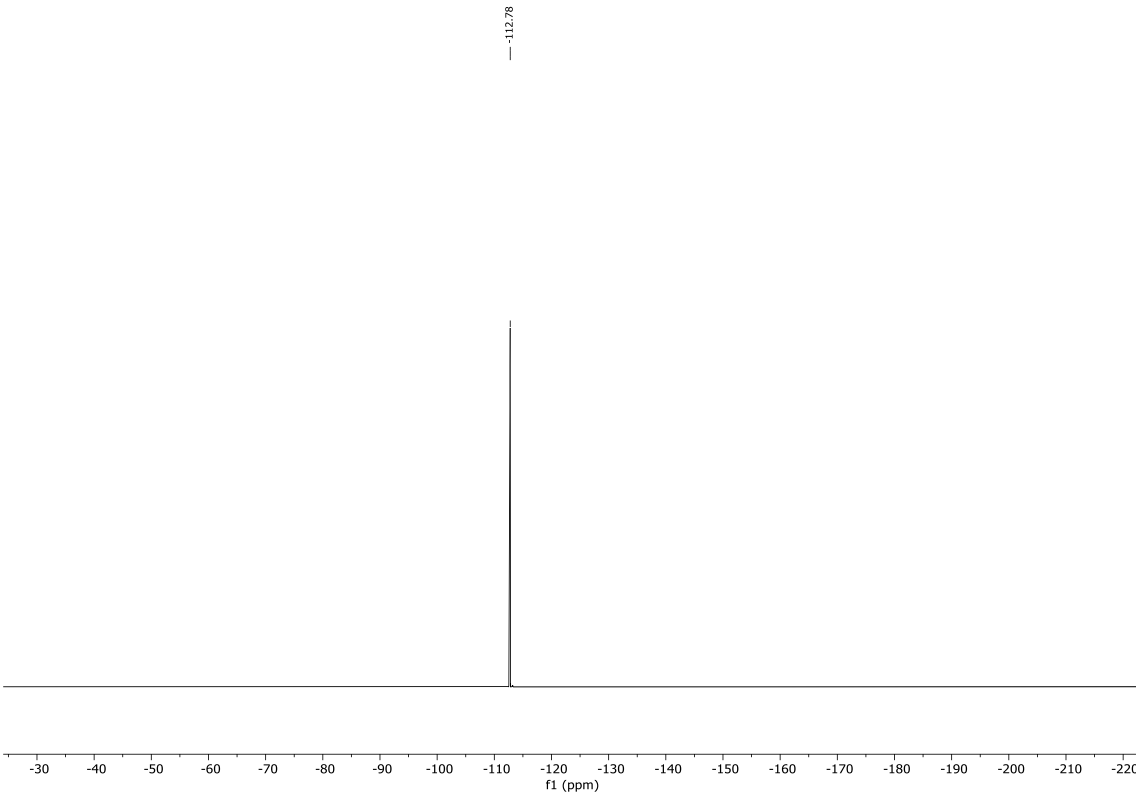

^{1}H NMR (400 MHz, $CDCl_3$) of **1q**

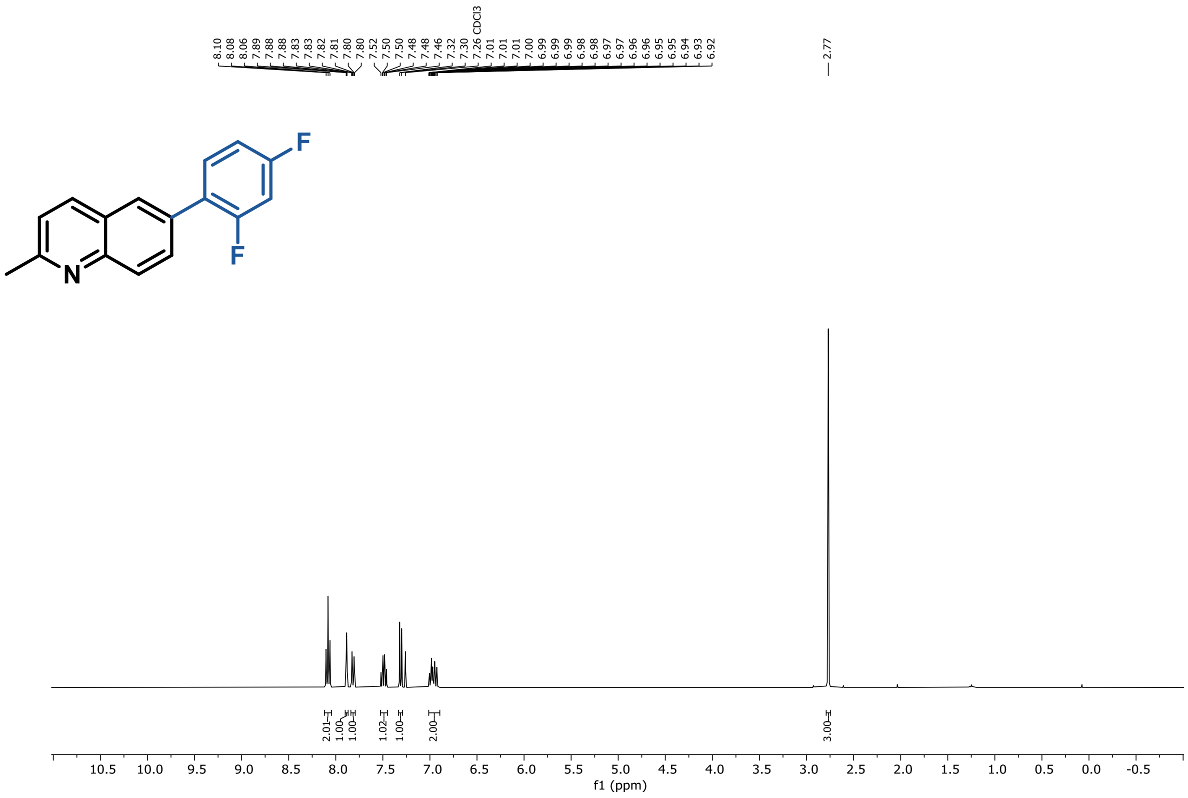

$^{13}C\{^{19}F\}$ NMR (126 MHz, $CDCl_3$) of **1q**

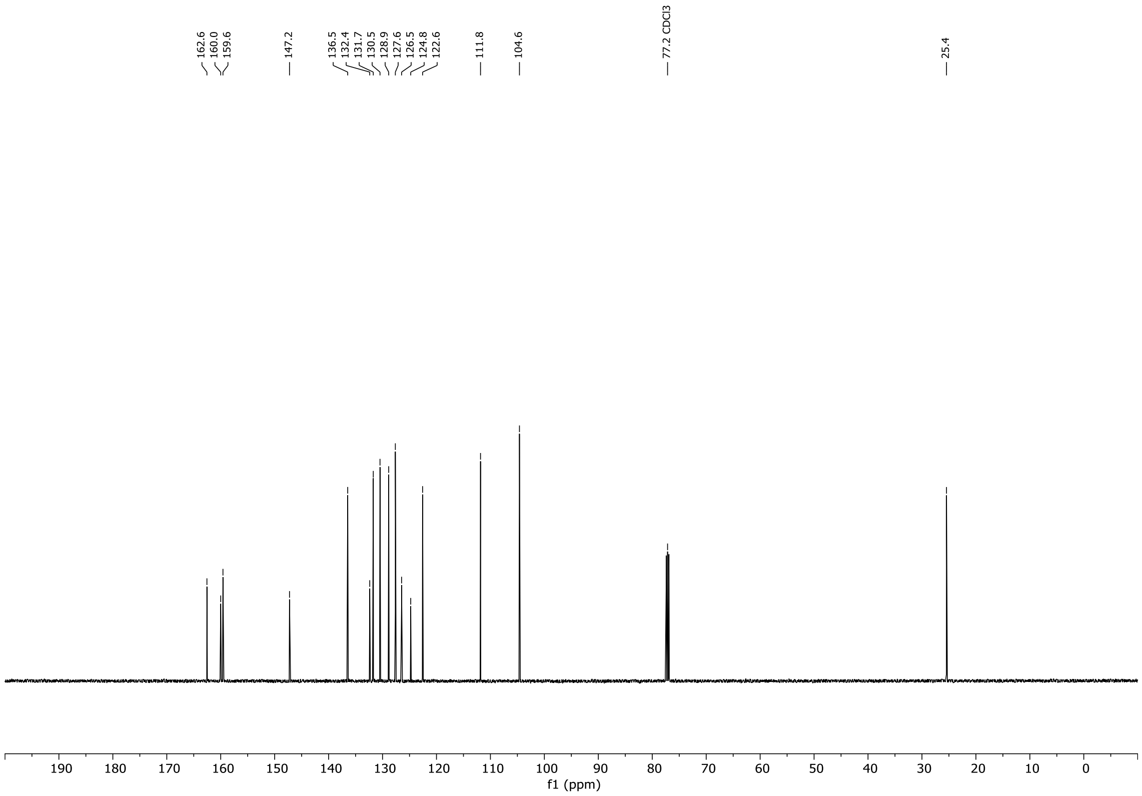

^{19}F NMR (376 MHz, $CDCl_3$) of **1q**

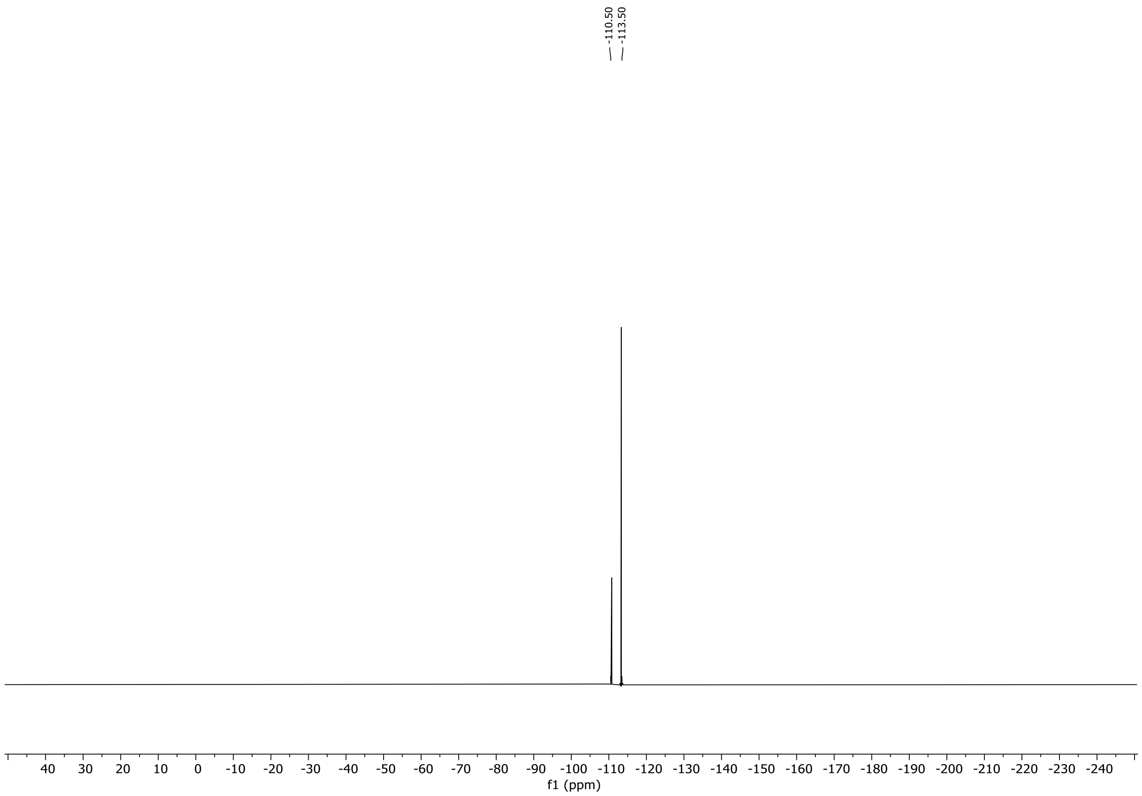

^{1}H NMR (400 MHz, $CDCl_3$) of **1r**

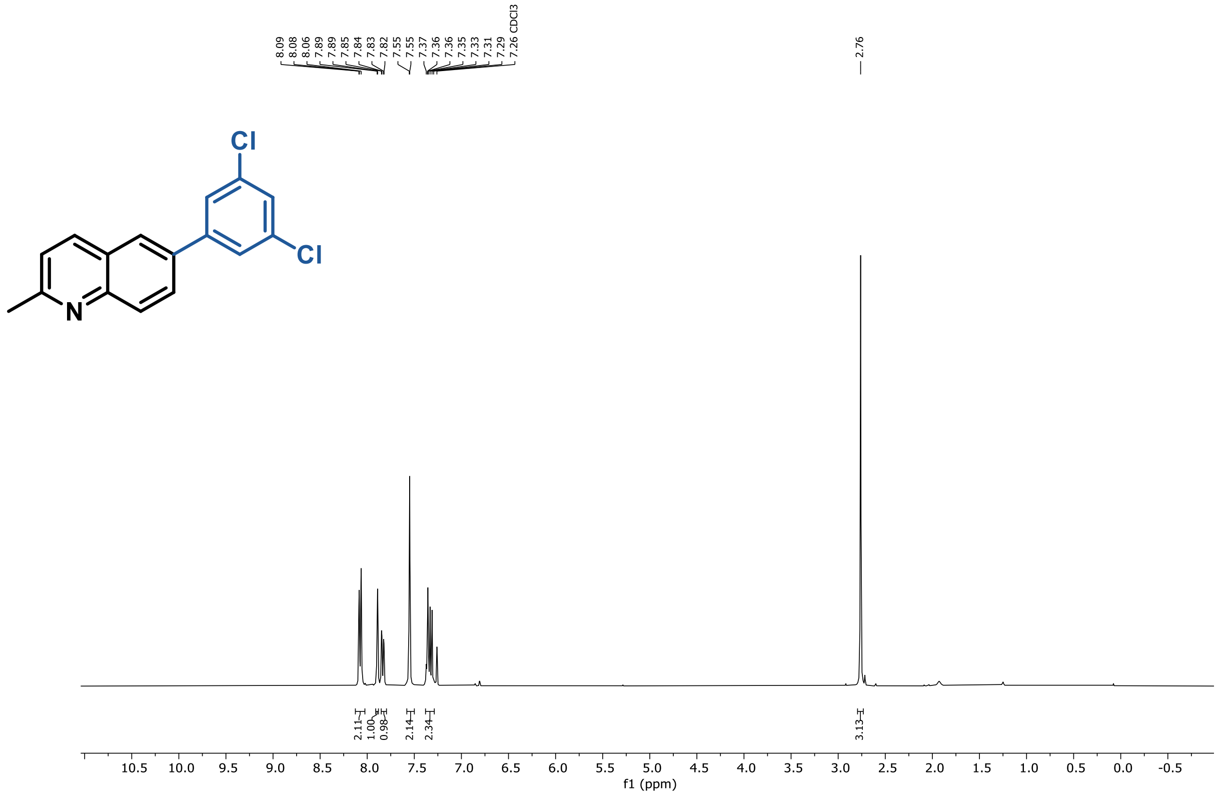

^{13}C NMR (101 MHz, $CDCl_3$) of **1r**

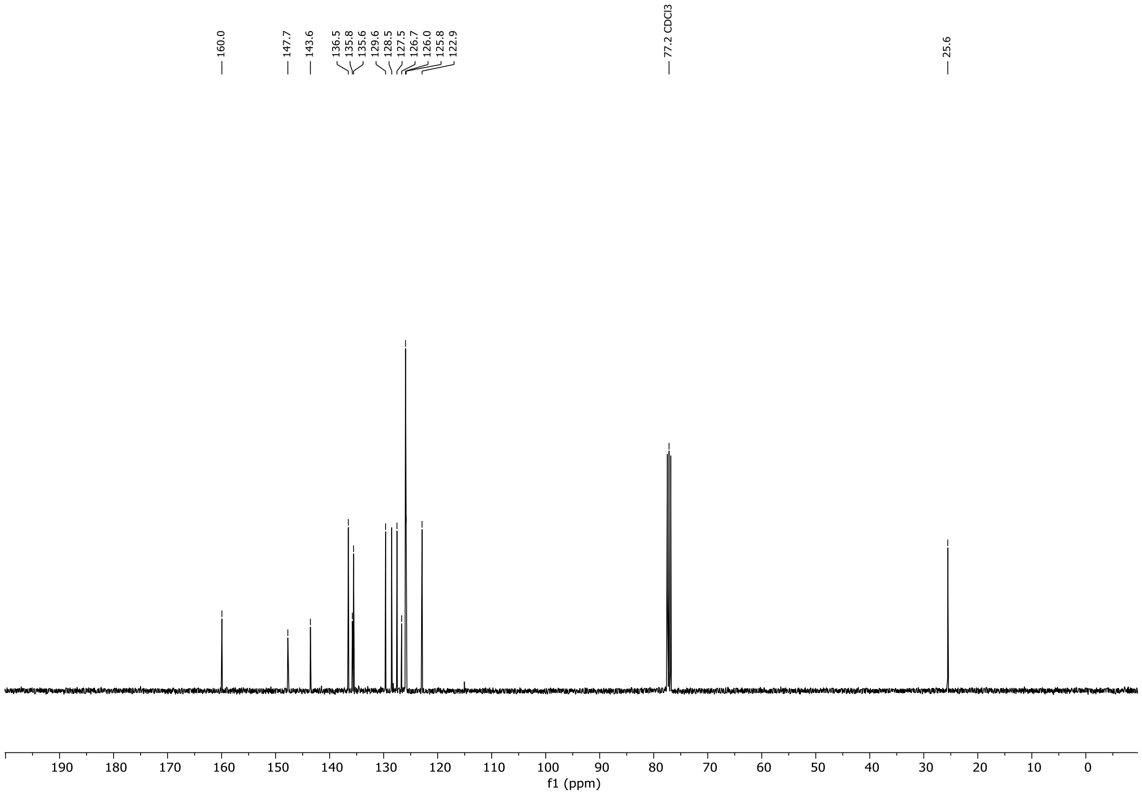

^{1}H NMR (400 MHz, $CDCl_3$) of **1s**

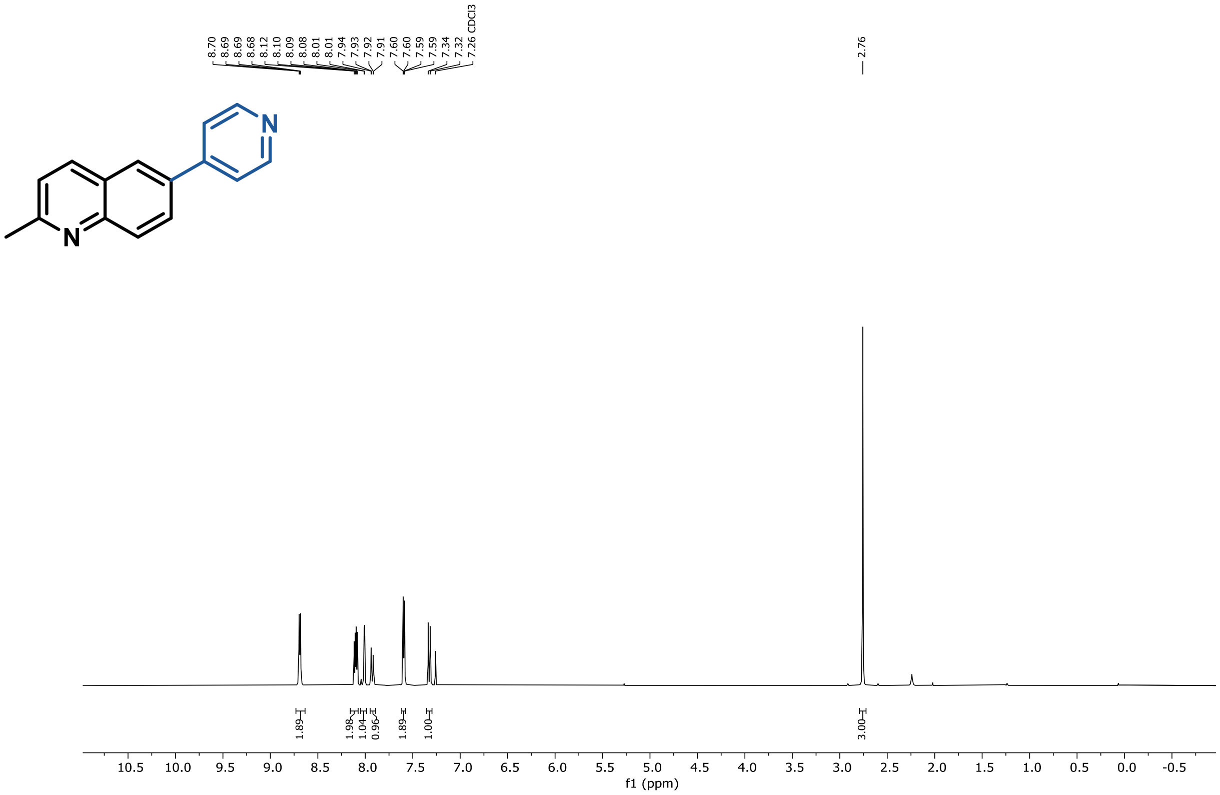

^{13}C NMR (101 MHz, $CDCl_3$) of **1s**

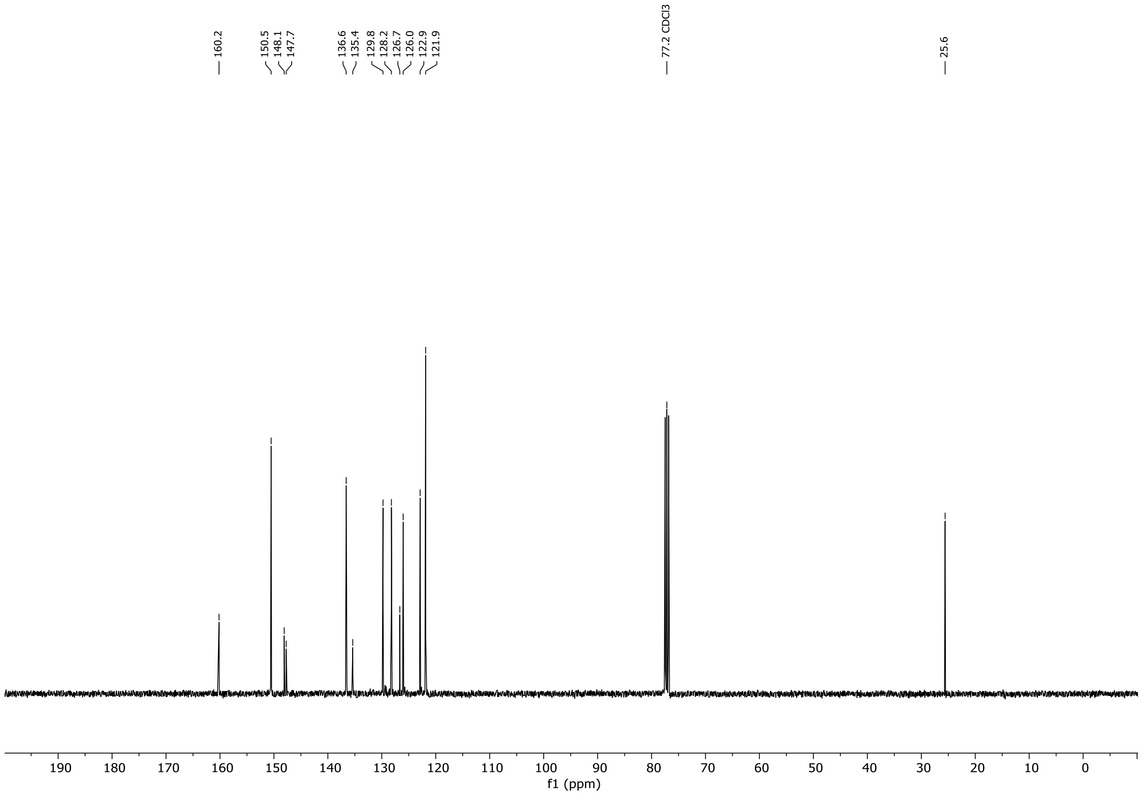

^{1}H NMR (400 MHz, $CDCl_3$) of **1t**

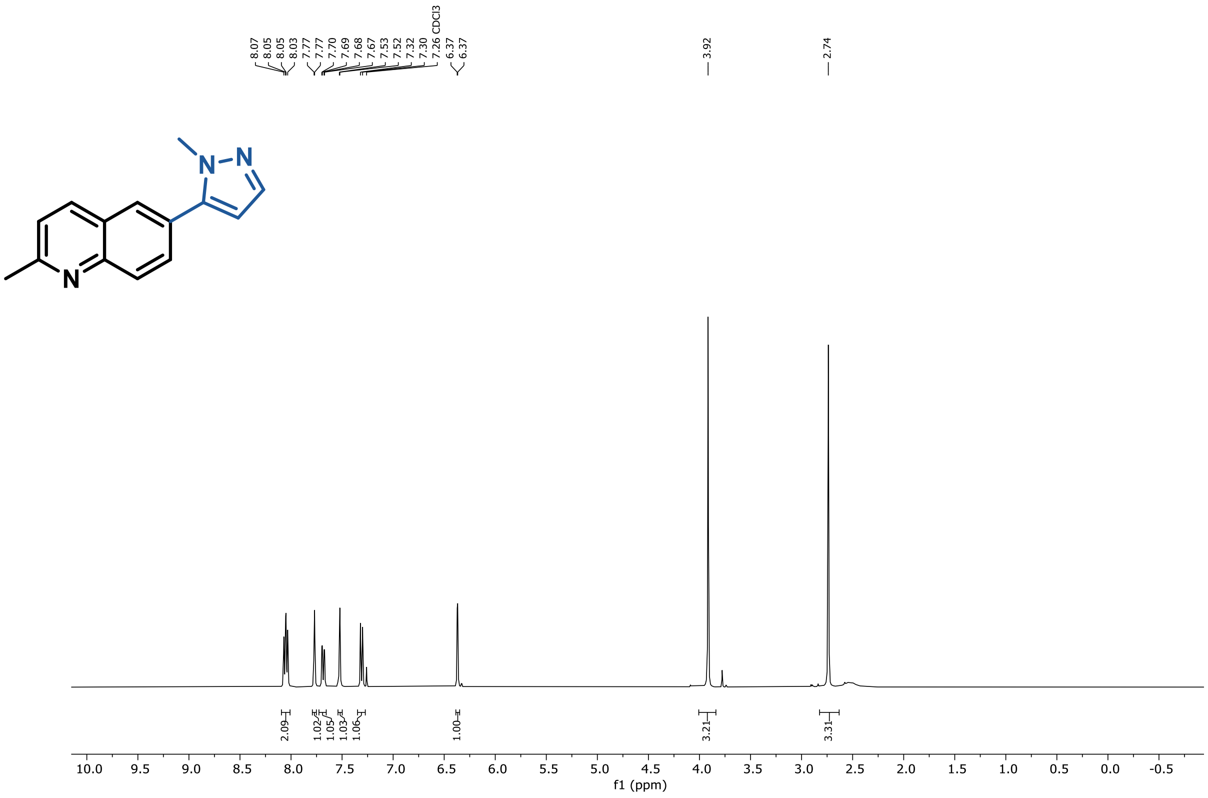

^{13}C NMR (101 MHz, $CDCl_3$) of **1t**

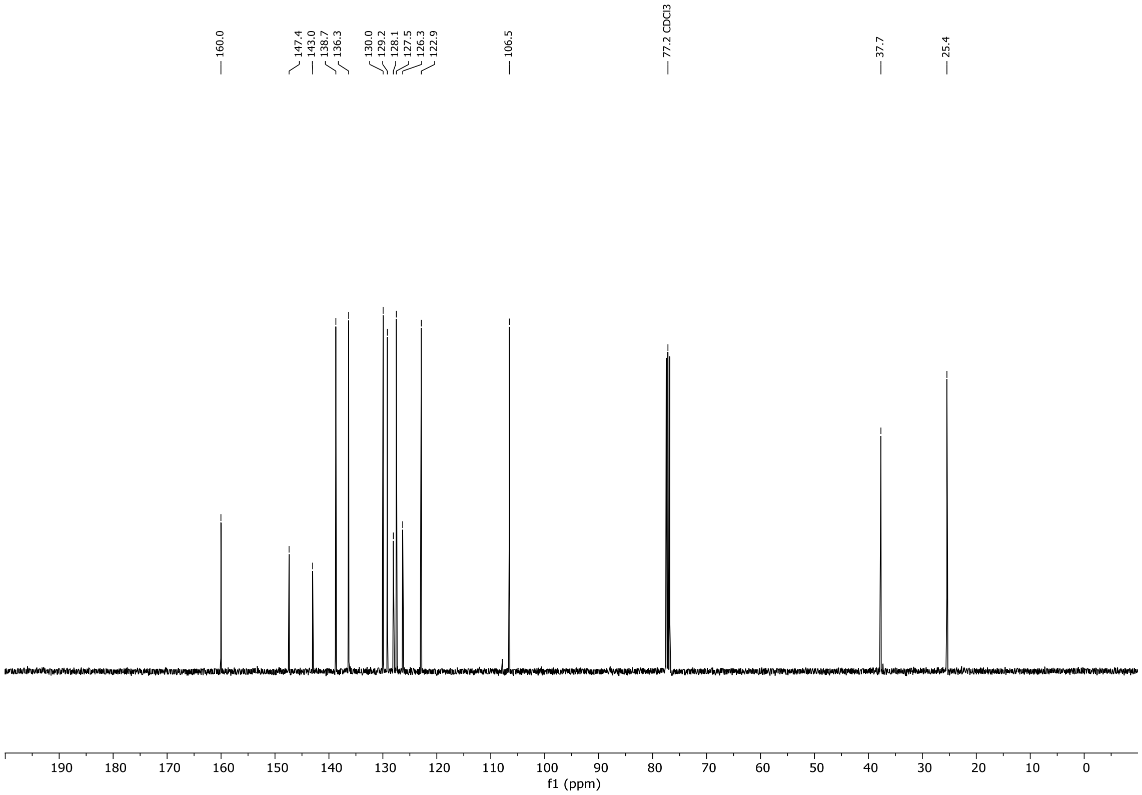

^{1}H NMR (400 MHz, $CDCl_3$) of **1u**

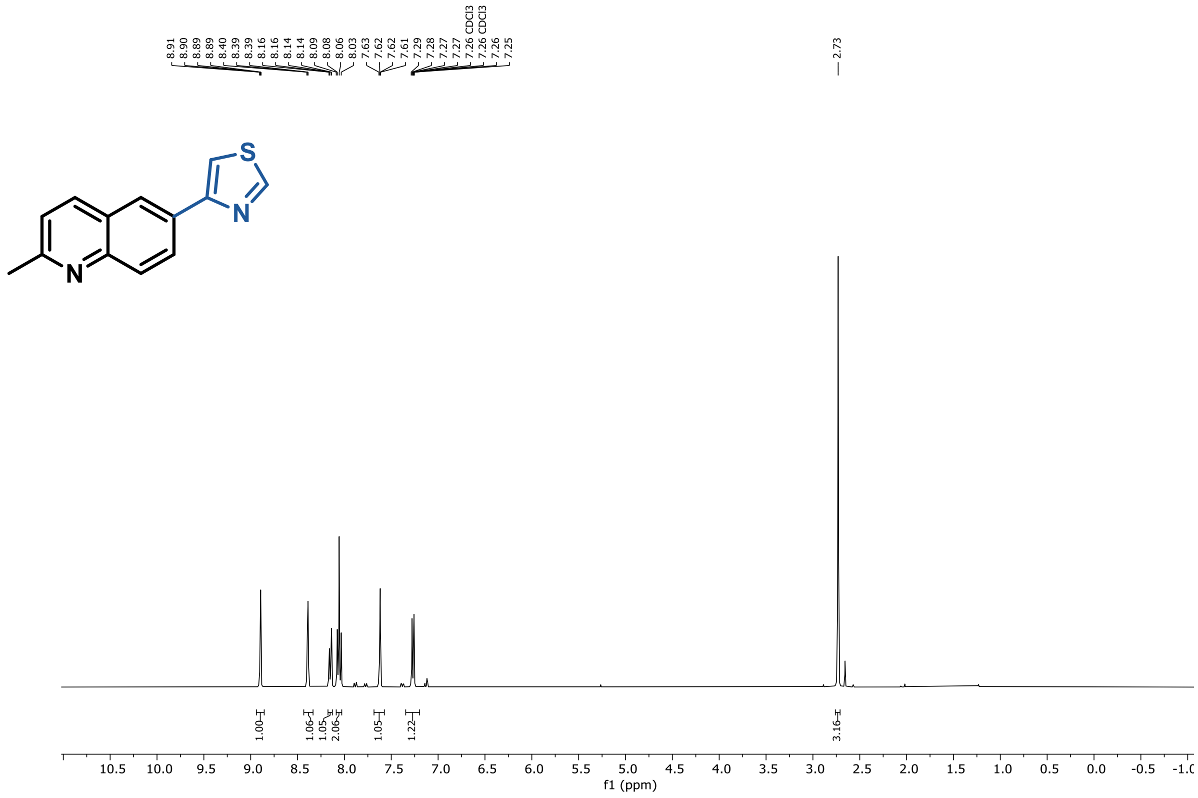

^{13}C NMR (101 MHz, $CDCl_3$) of **1u**

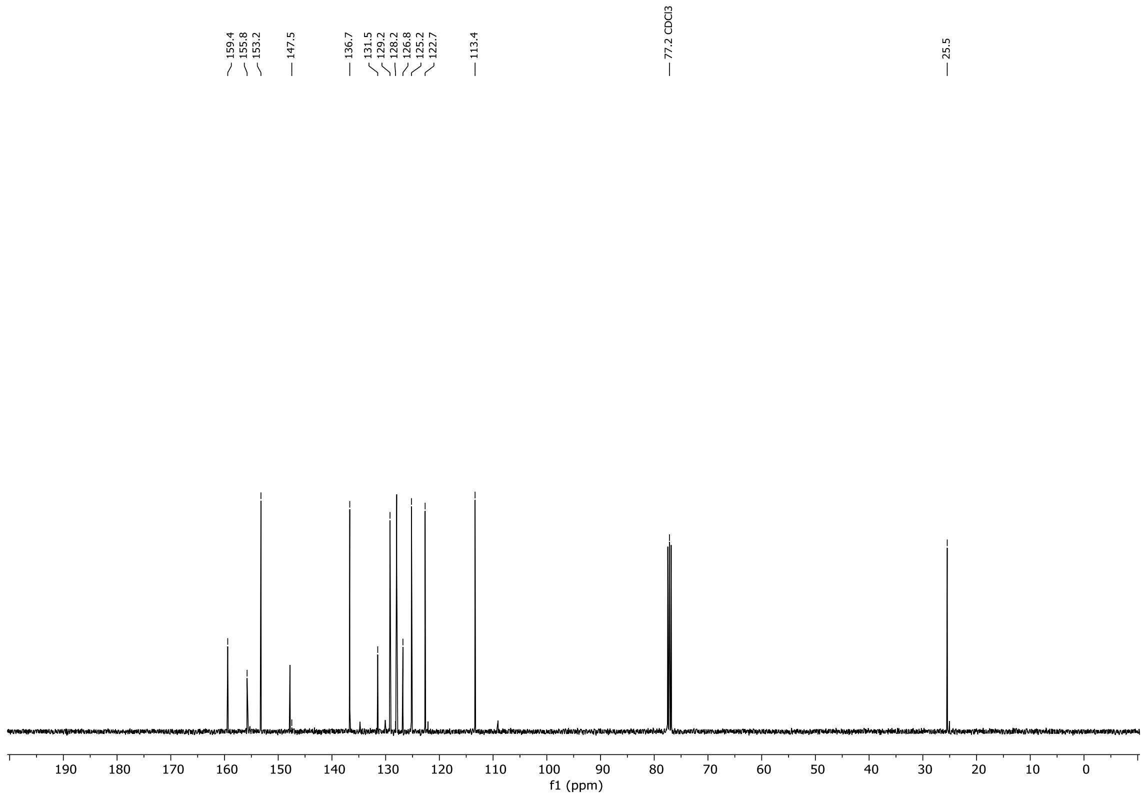

^{1}H NMR (400 MHz, $CDCl_3$) of **1v**

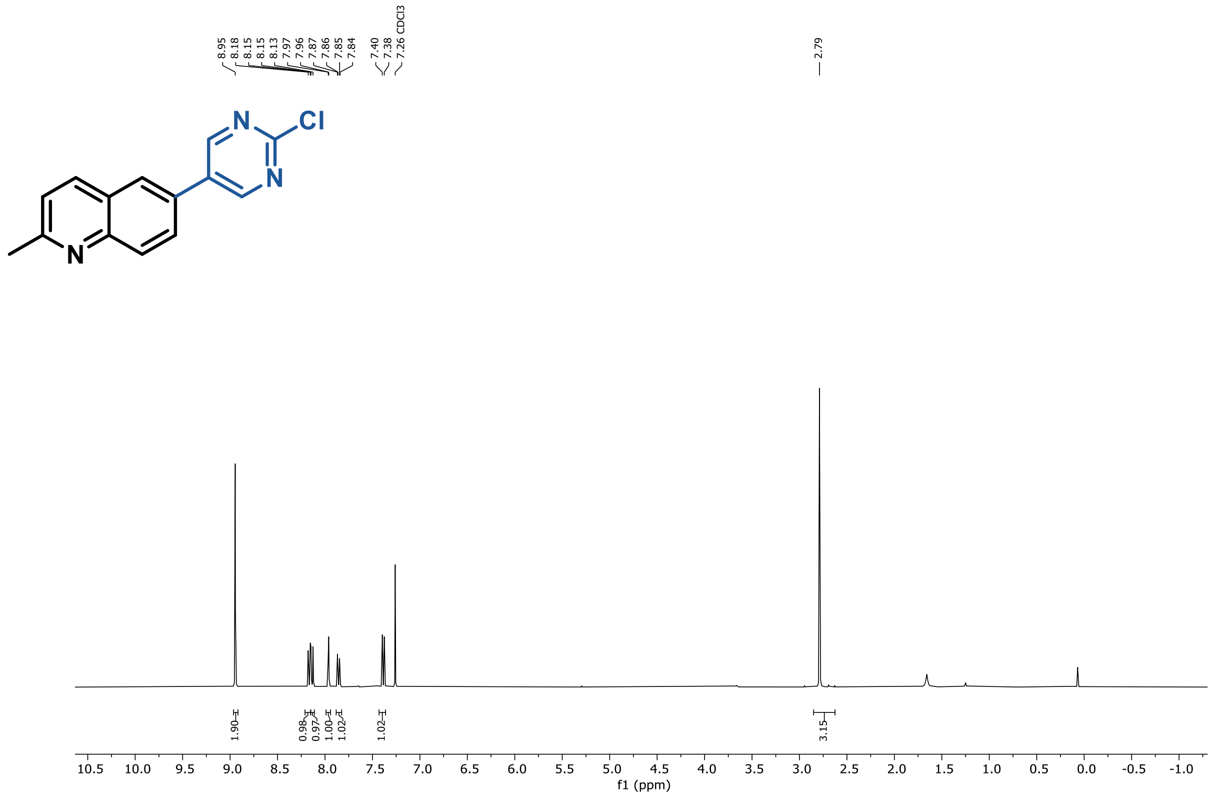

^{13}C NMR (101 MHz, $CDCl_3$) of **1v**

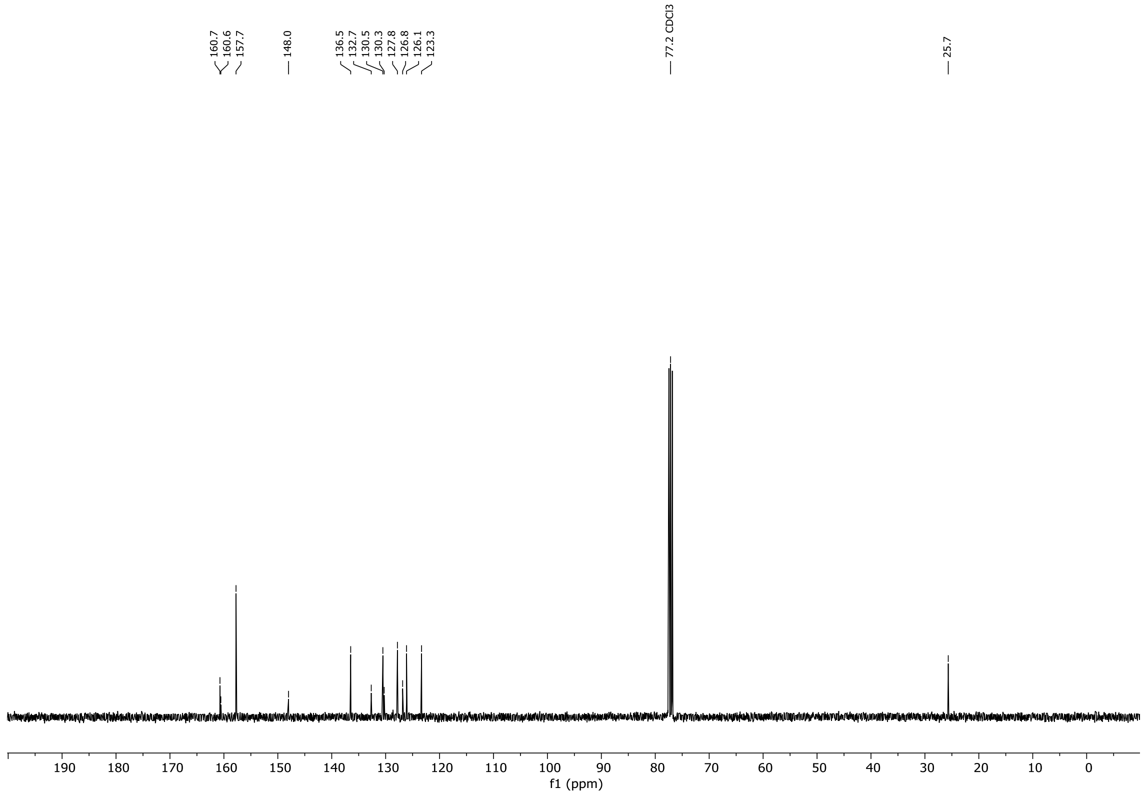

^{1}H NMR (400 MHz, $CDCl_3$) of **S2**

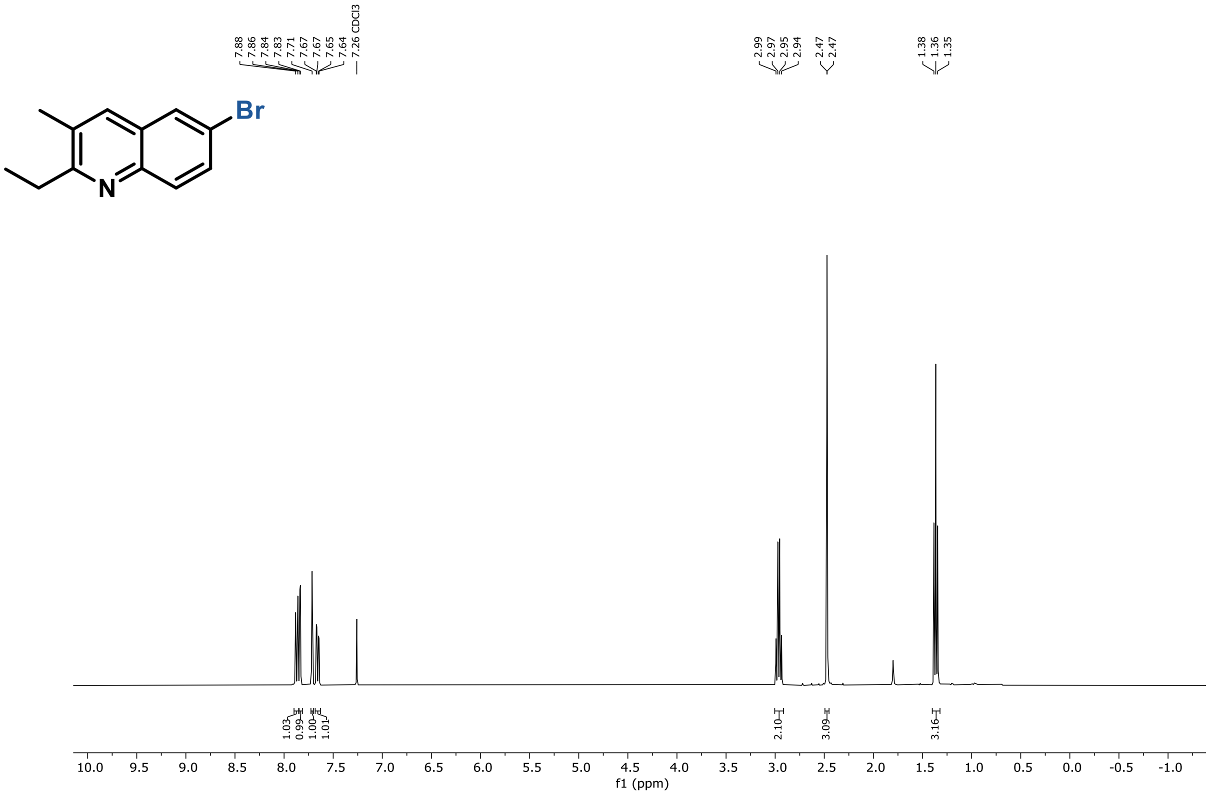

^{13}C NMR (101 MHz, $CDCl_3$) of **S2**

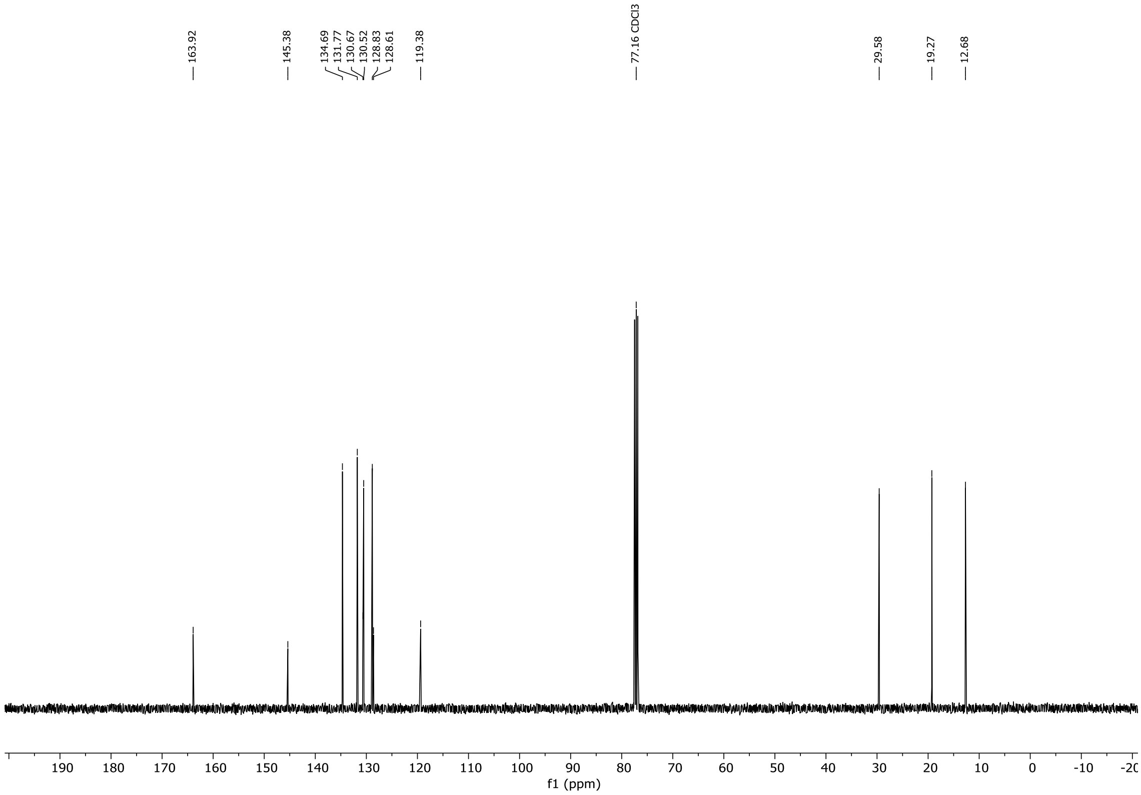

^{1}H NMR (400 MHz, $CDCl_3$) of **1x**

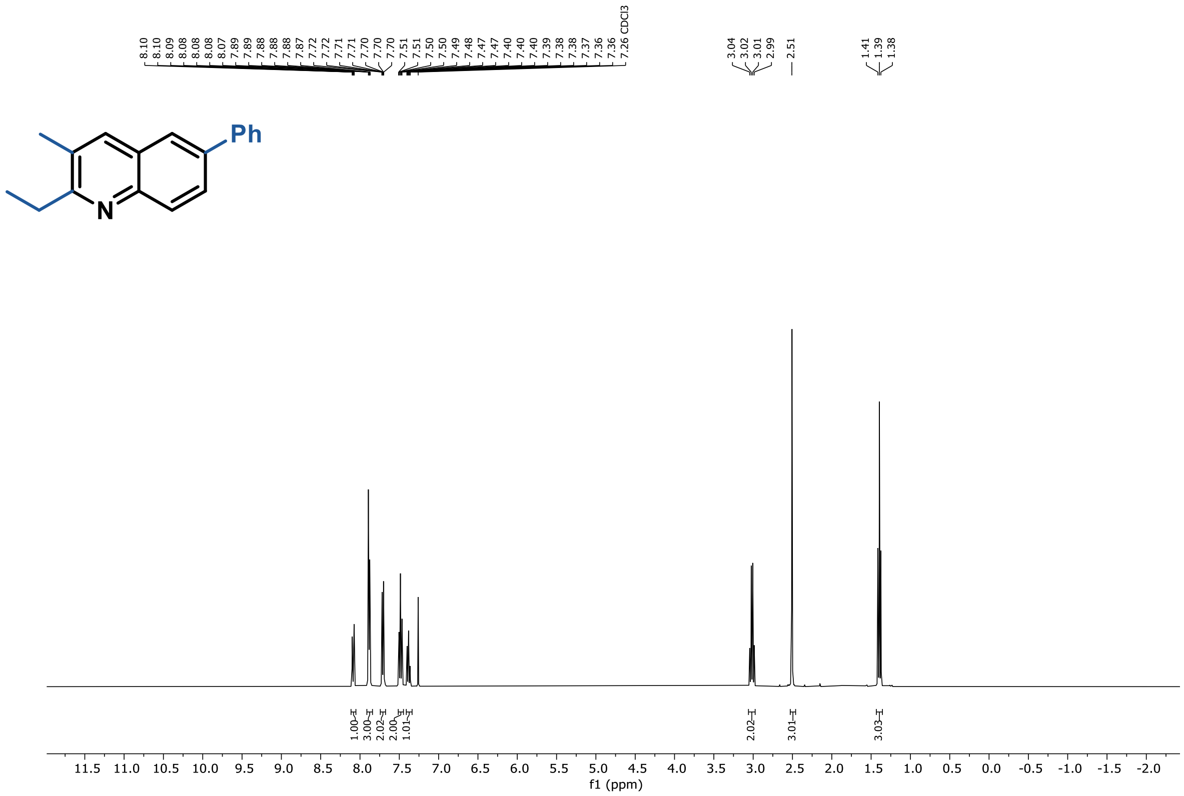

^{13}C NMR (101 MHz, $CDCl_3$) of **1x**

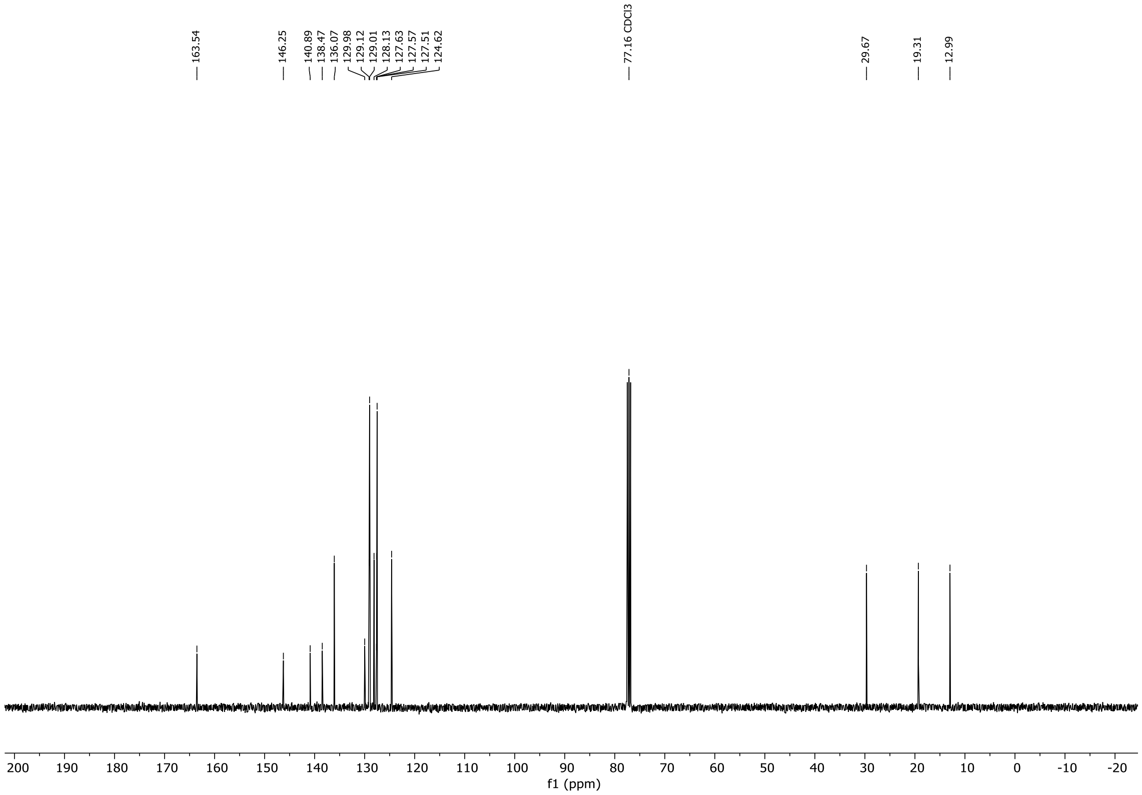

^{1}H NMR (400 MHz, $CDCl_3$) of **1y**

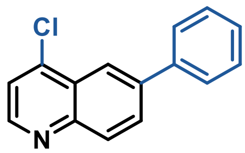

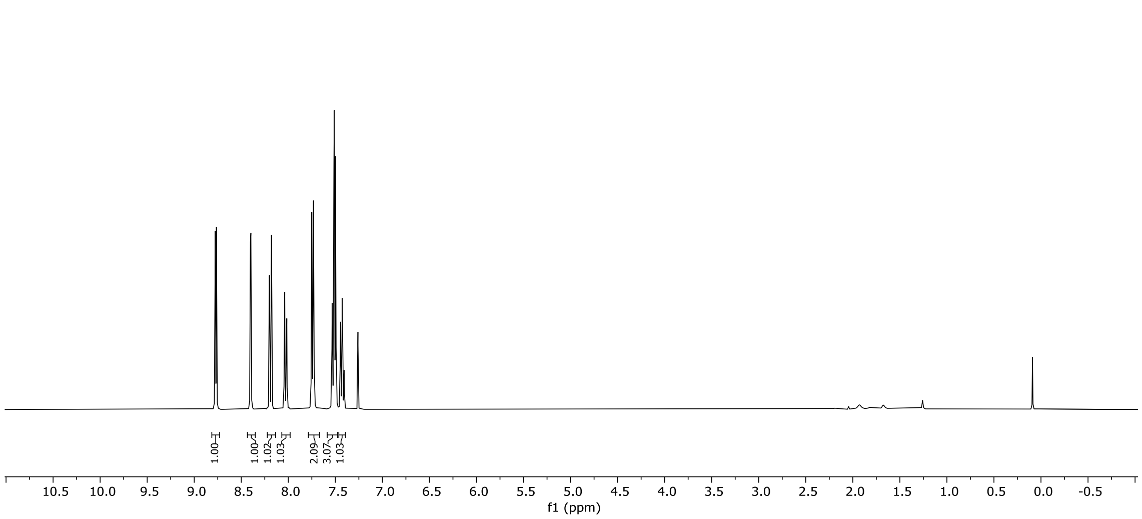

^{13}C NMR (101 MHz, $CDCl_3$) of **1y**

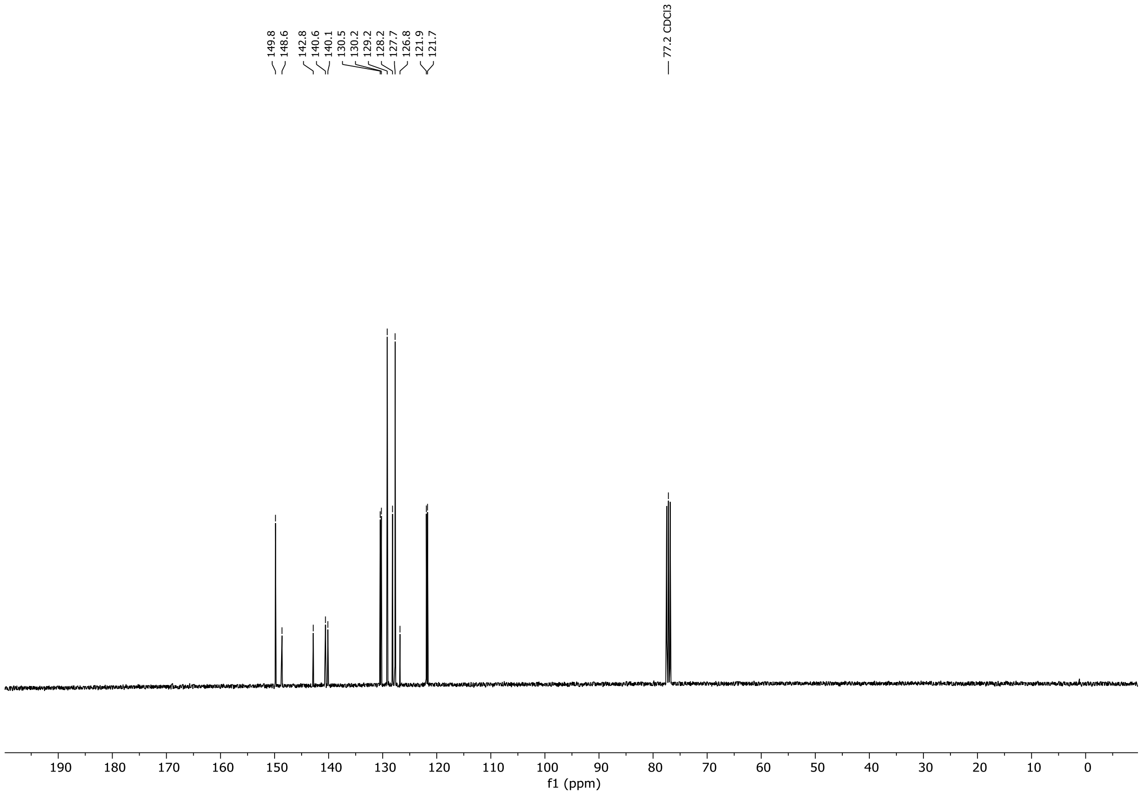

^{1}H NMR (400 MHz, $CDCl_3$) of **1z**

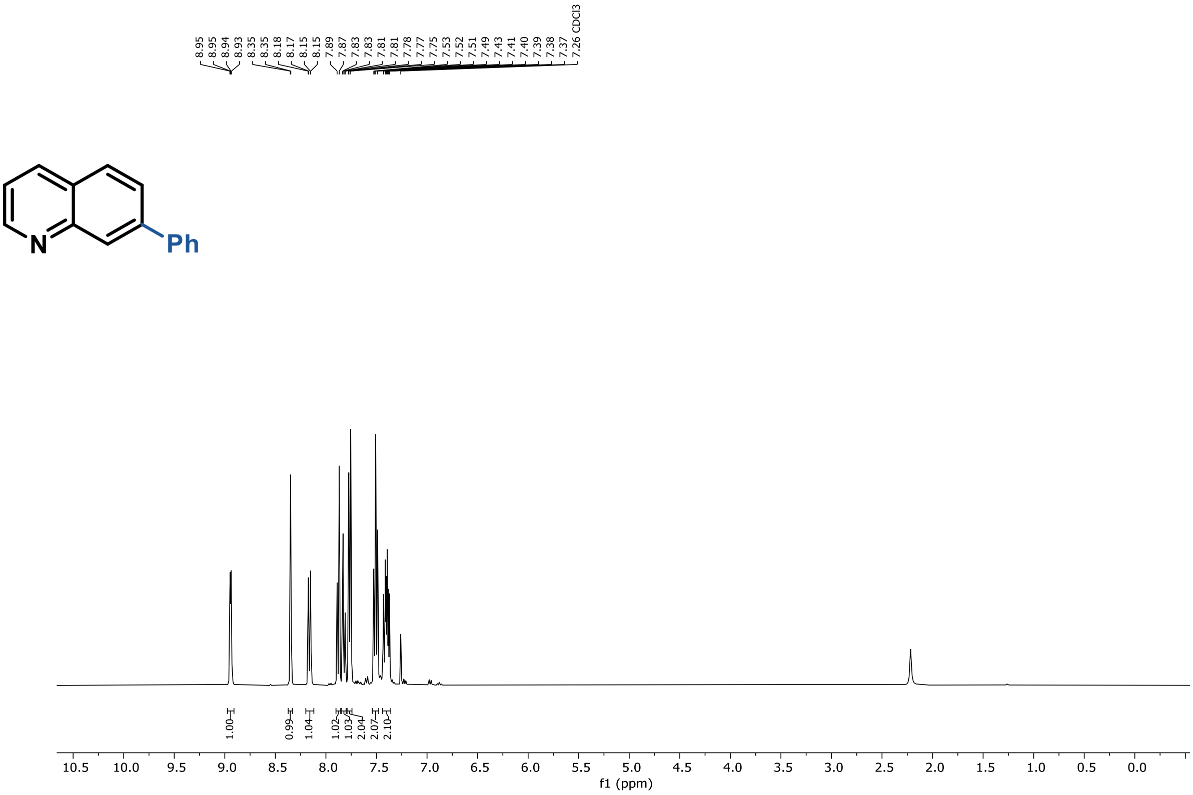

^{13}C NMR (101 MHz, $CDCl_3$) of **1z**

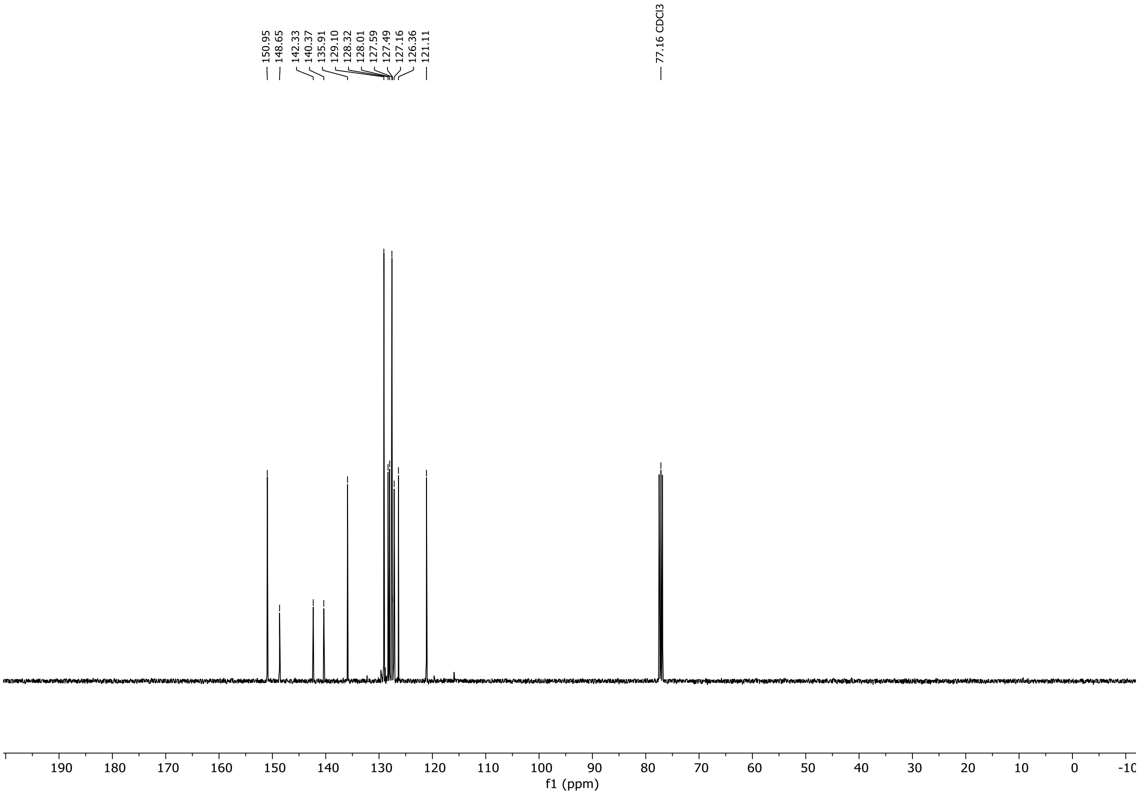

^{1}H NMR (400 MHz, $CDCl_3$) of **1aa**

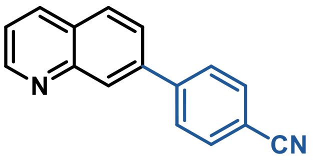

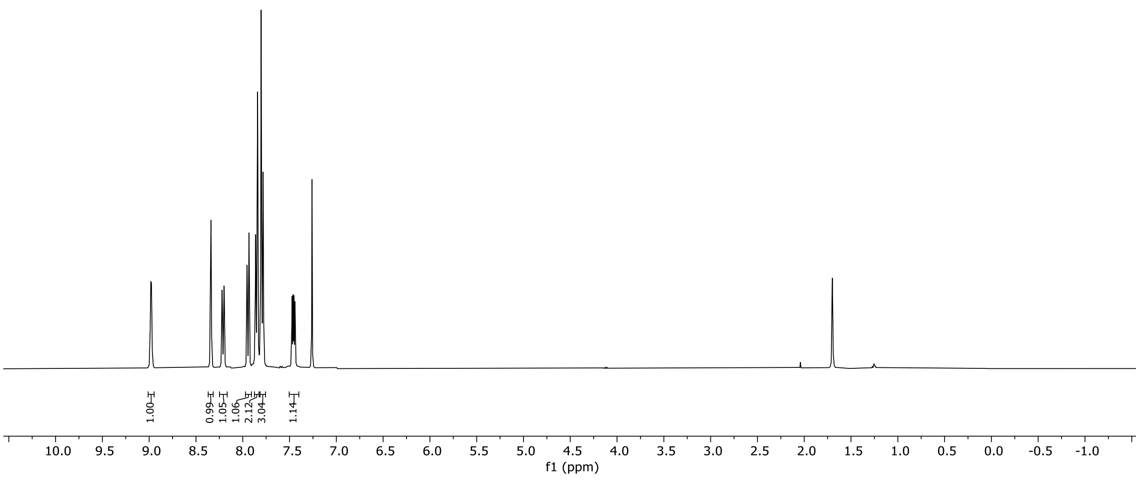

^{13}C NMR (101 MHz, $CDCl_3$) of **1aa**

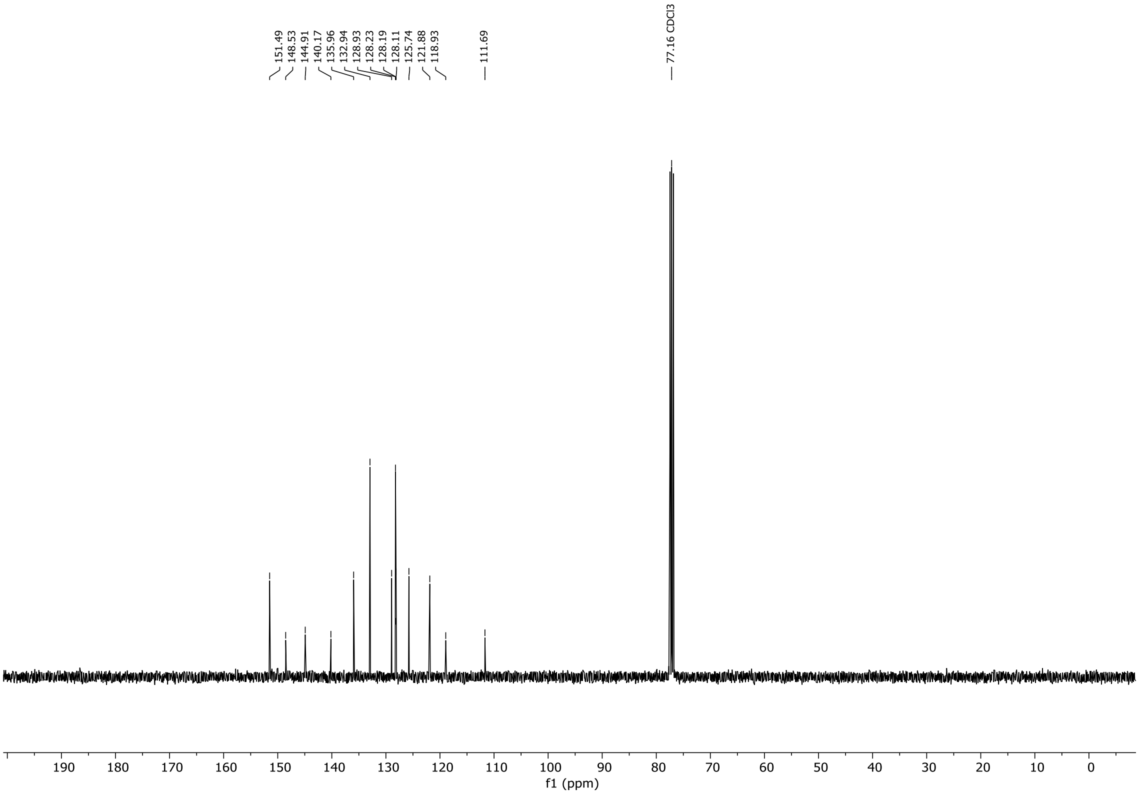

^{1}H NMR (400 MHz, $CDCl_3$) of **1ab**

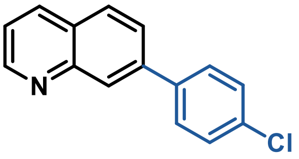

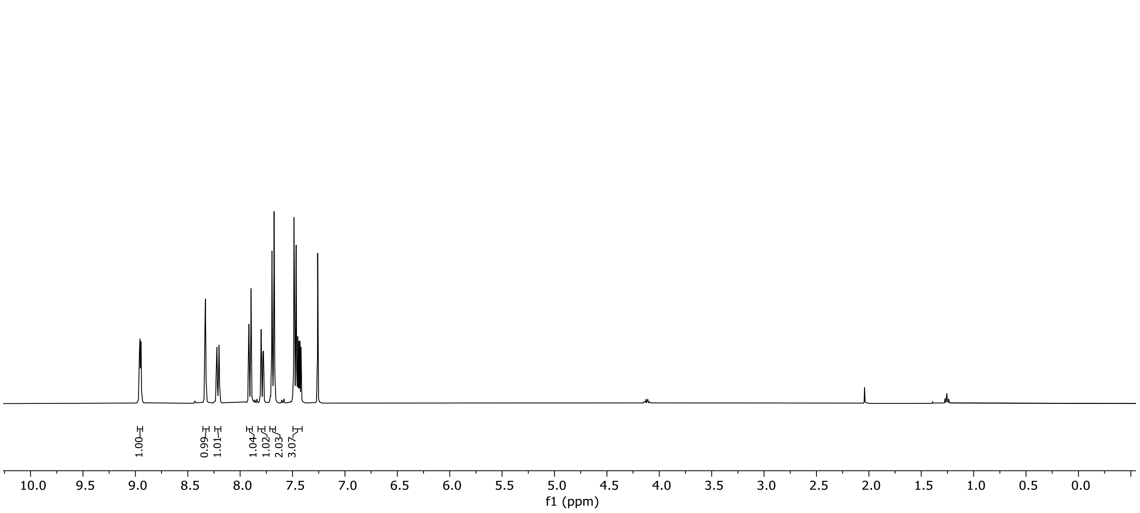

^{13}C NMR (101 MHz, $CDCl_3$) of **1ab**

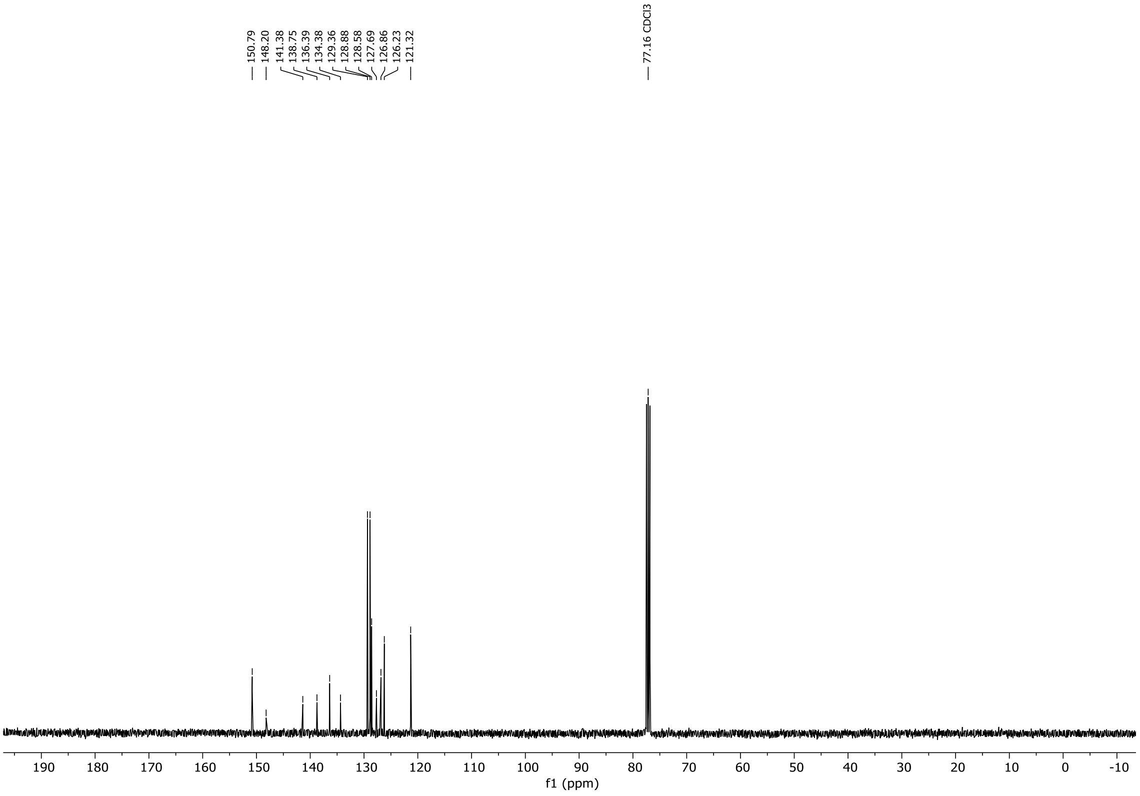

^{1}H NMR (400 MHz, $CDCl_3$) of **1ac**

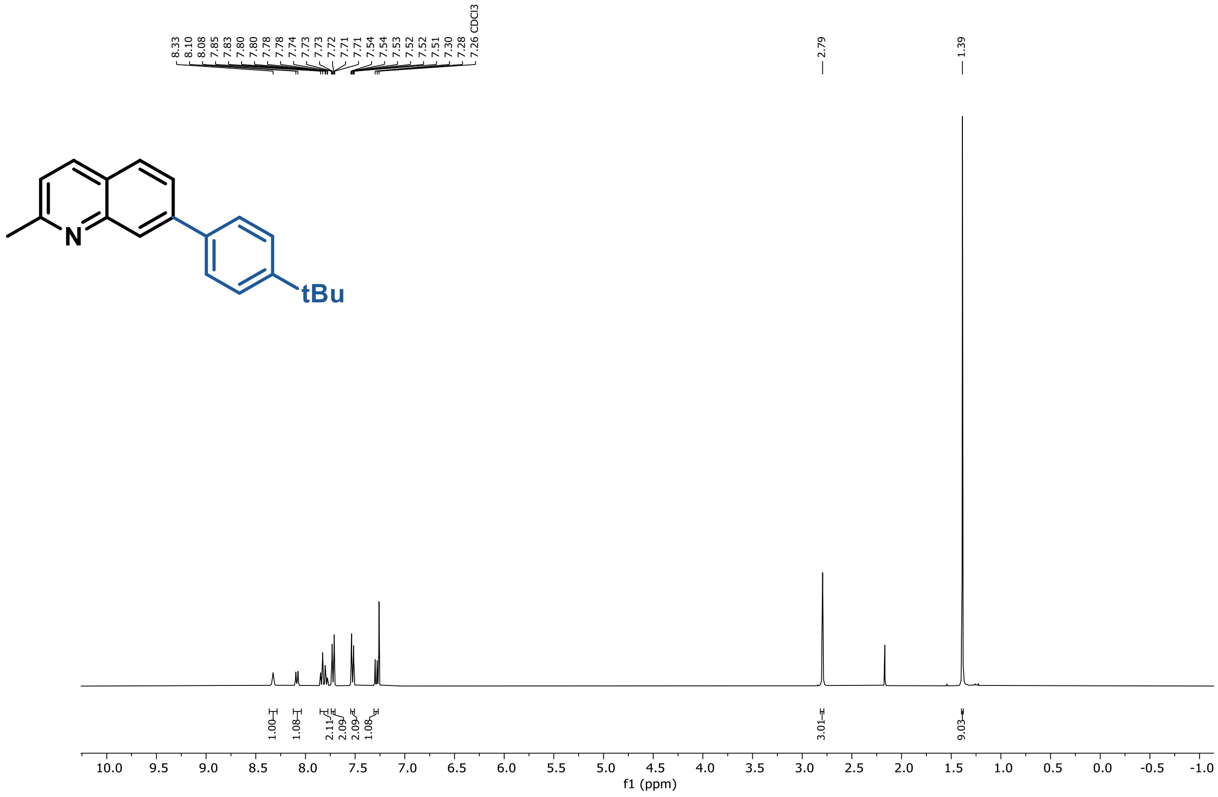

^{13}C NMR (101 MHz, $CDCl_3$) of **1ac**

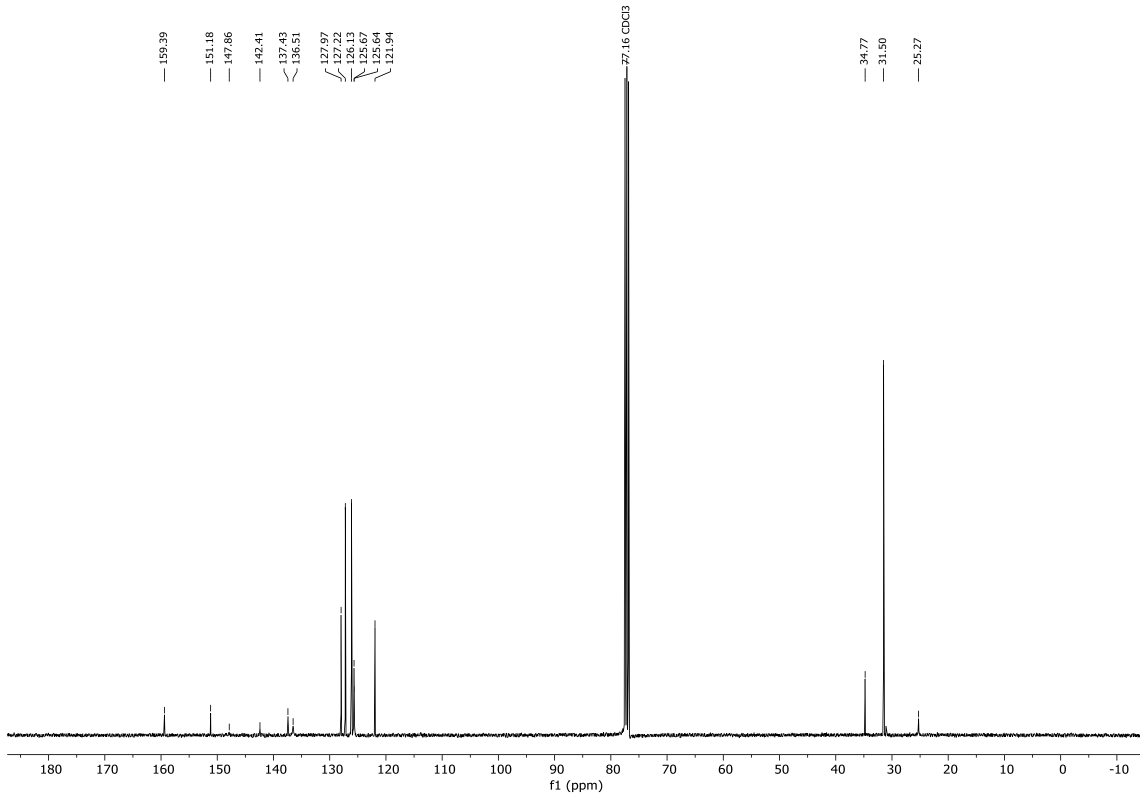

^{1}H NMR (400 MHz, $CDCl_3$) of **S3**

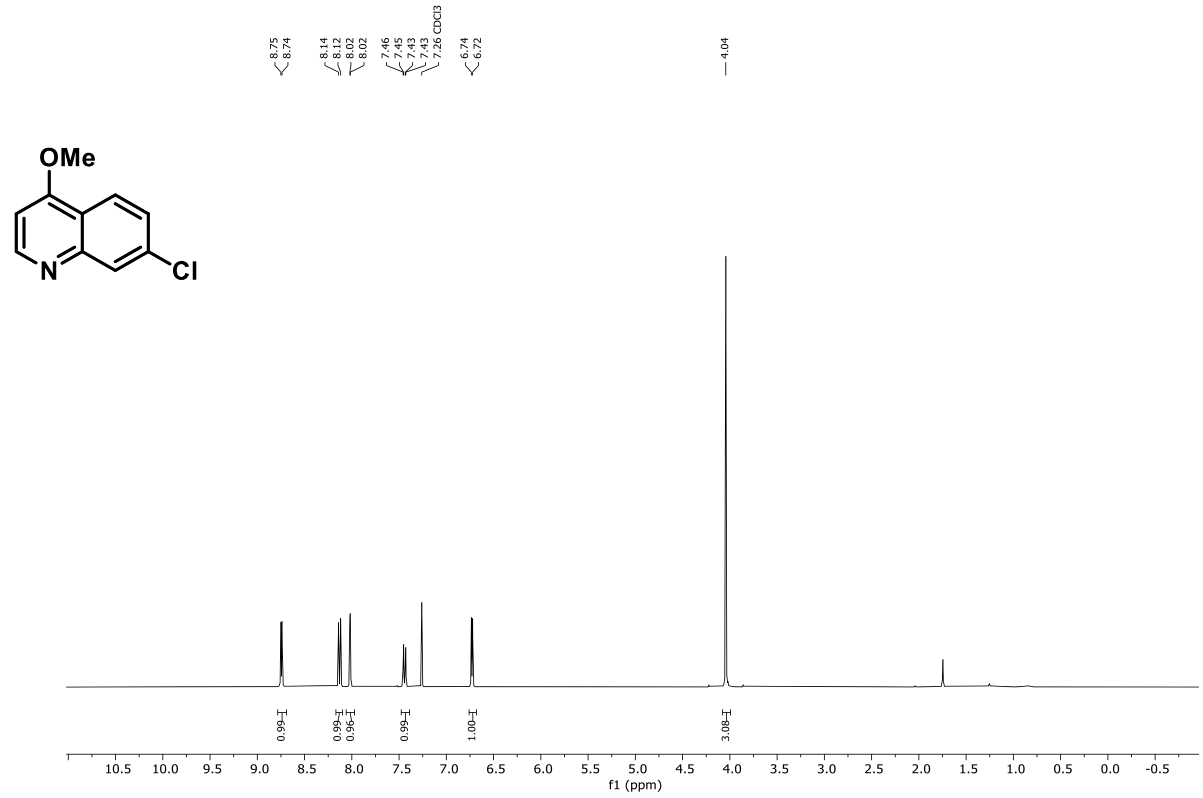

^{13}C NMR (101 MHz, $CDCl_3$) of **S3**

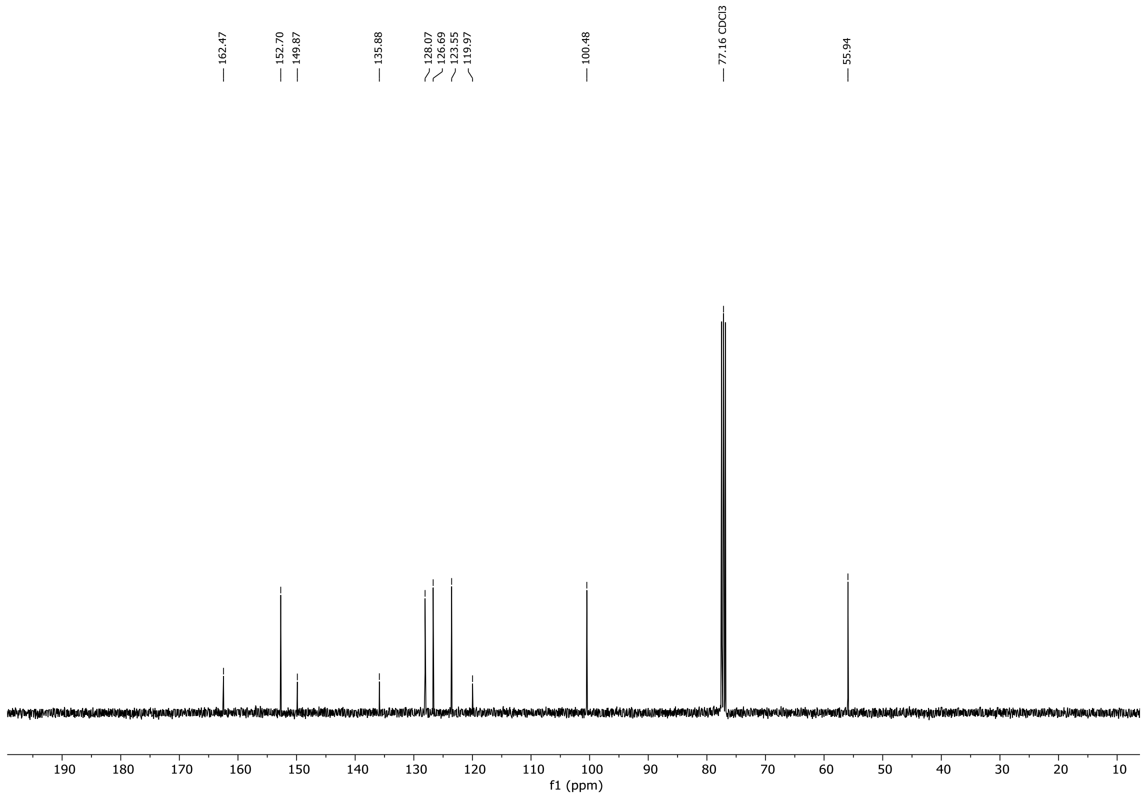

^{1}H NMR (400 MHz, $CDCl_3$) of **1ad**

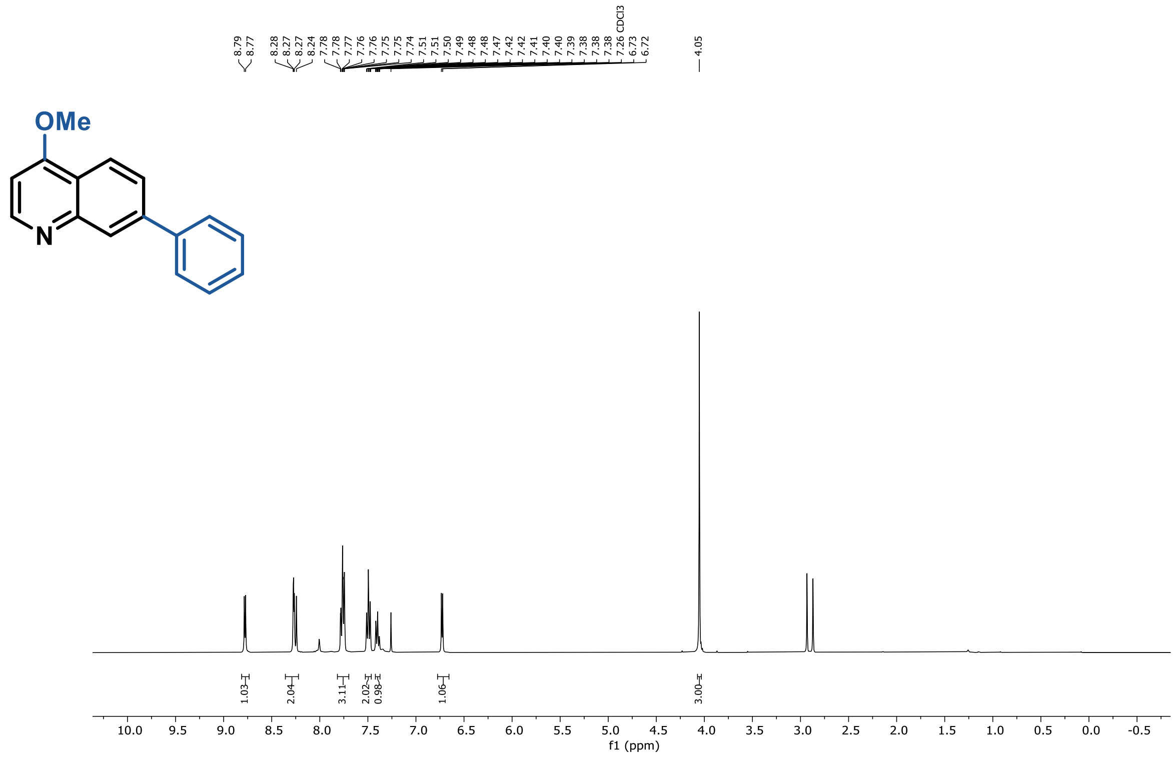

^{13}C NMR (101 MHz, $CDCl_3$) of **1ad**

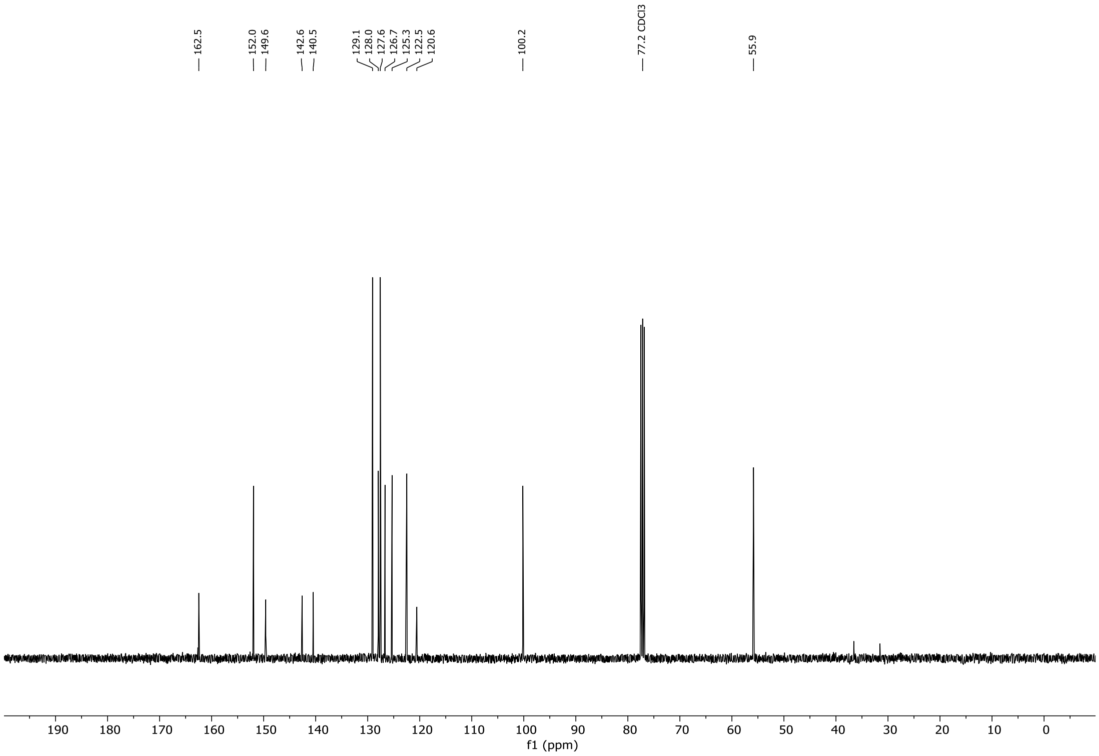

^{1}H NMR (400 MHz, $CDCl_3$) of **1ae**

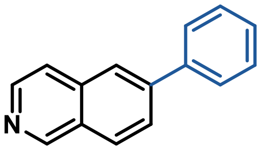

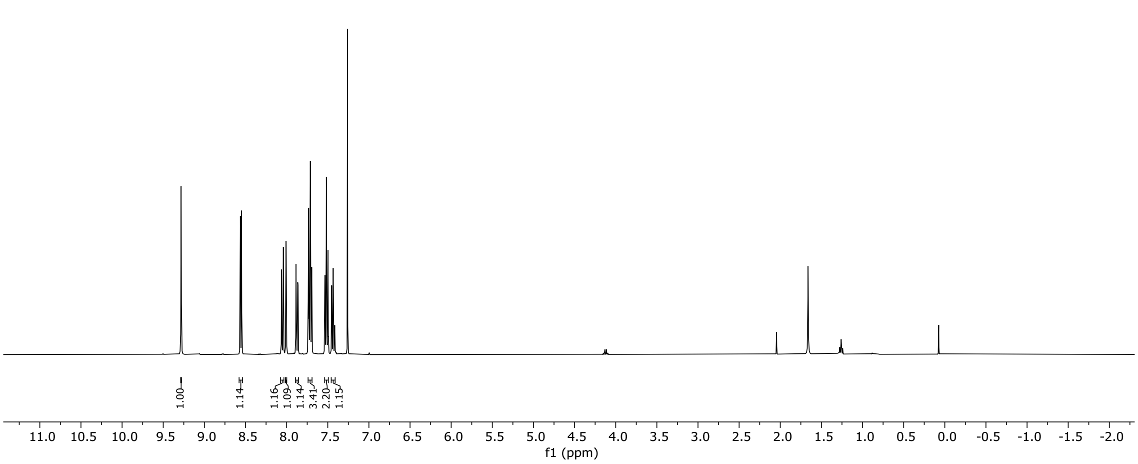

^{13}C NMR (101 MHz, $CDCl_3$) of **1ae**

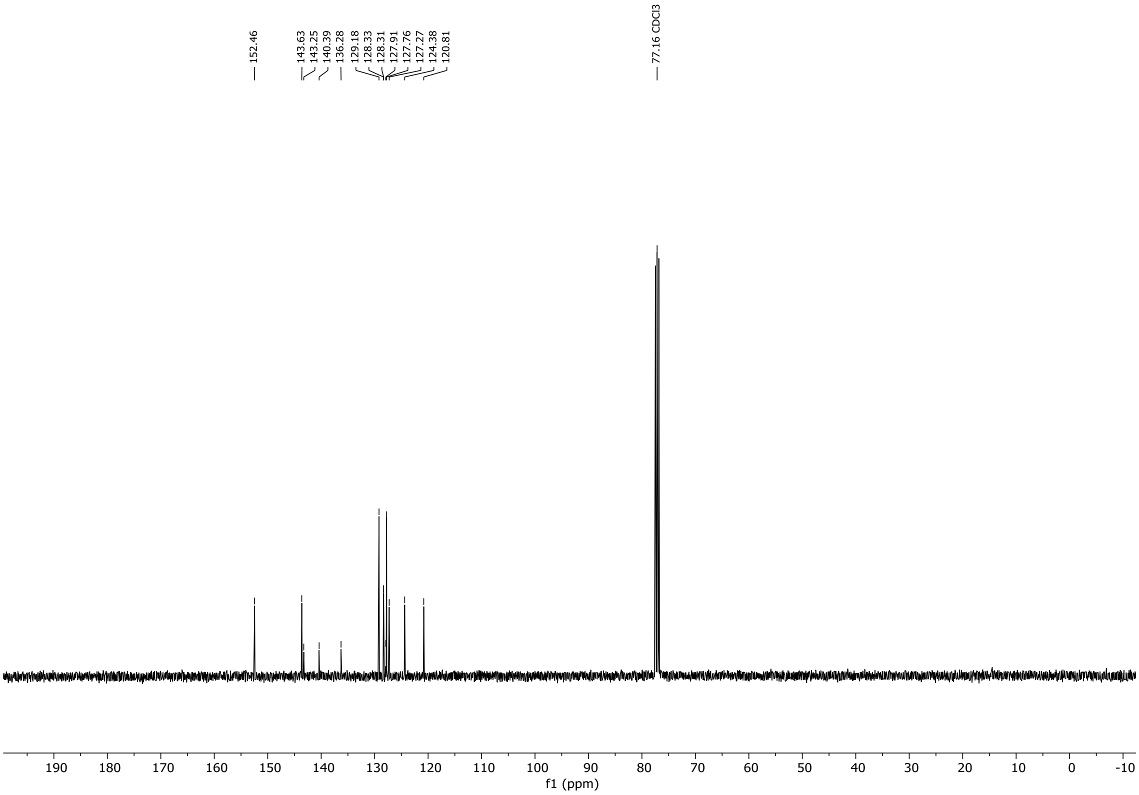

^{1}H NMR (599 MHz, $CDCl_3$) of **1af**

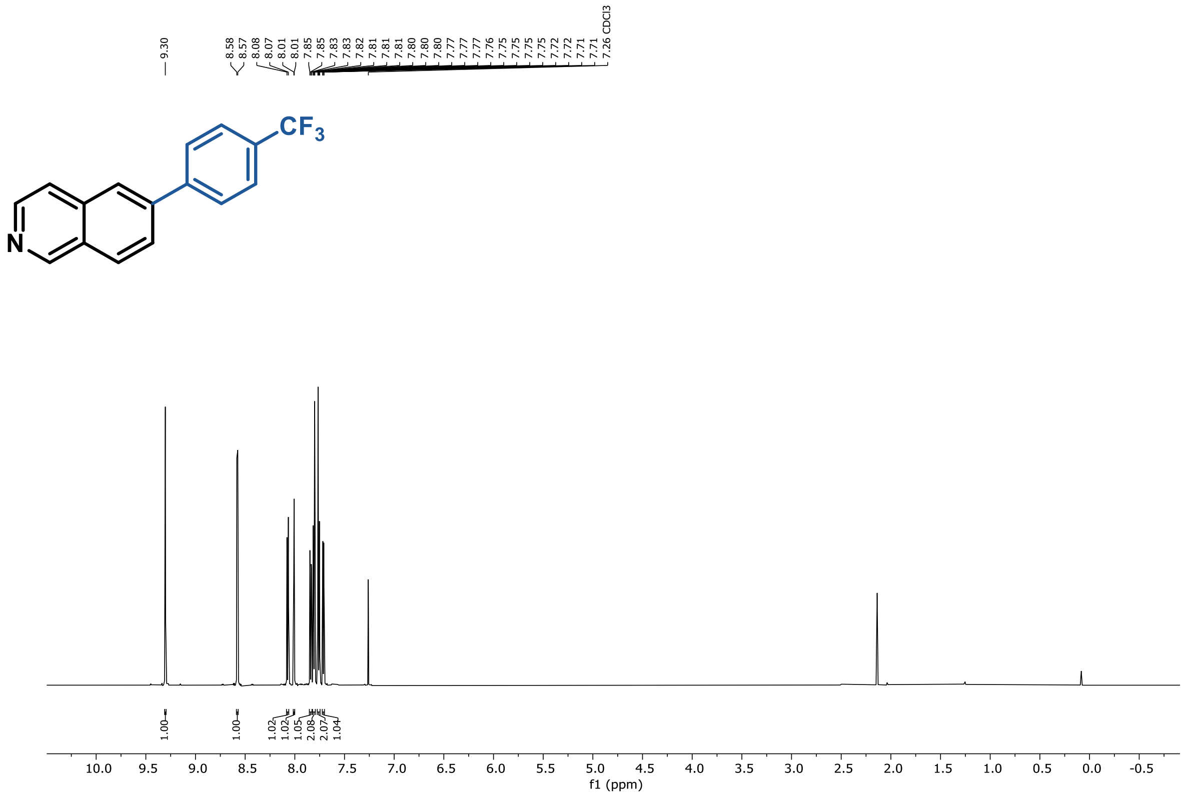

^{13}C NMR (151 MHz, $CDCl_3$) of **1af**

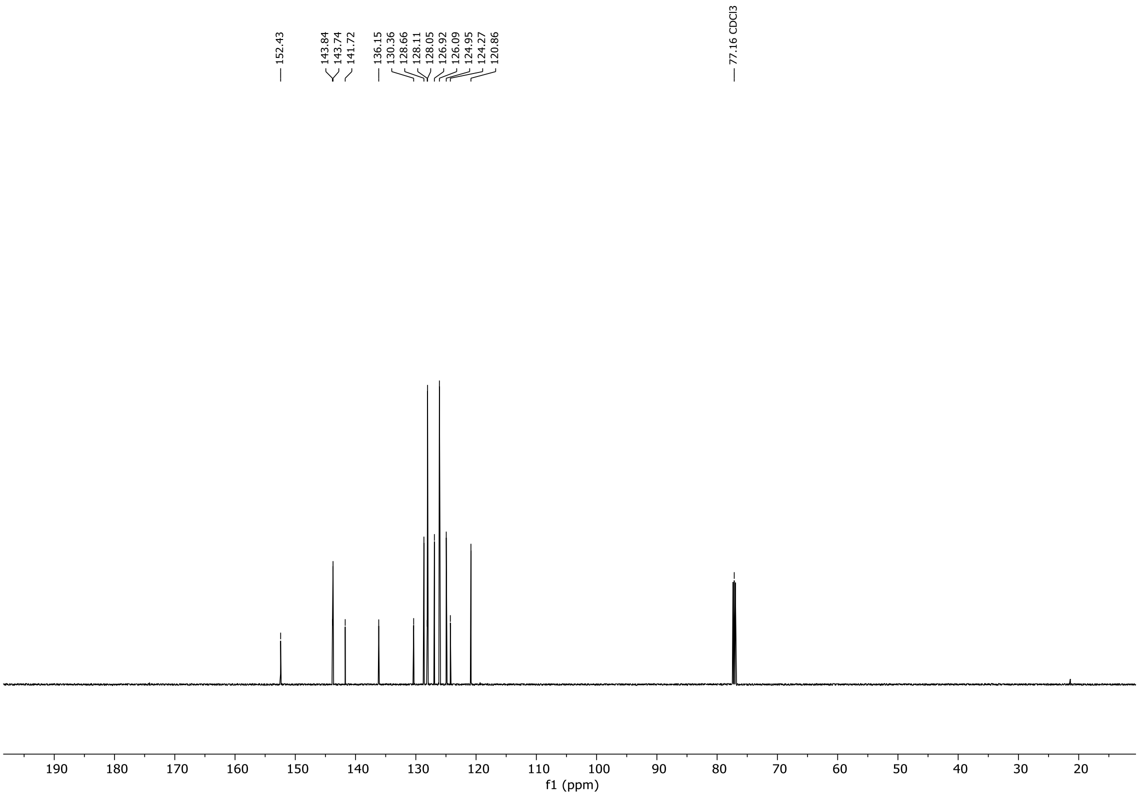

^{19}F NMR (564 MHz, $CDCl_3$) of **1af**

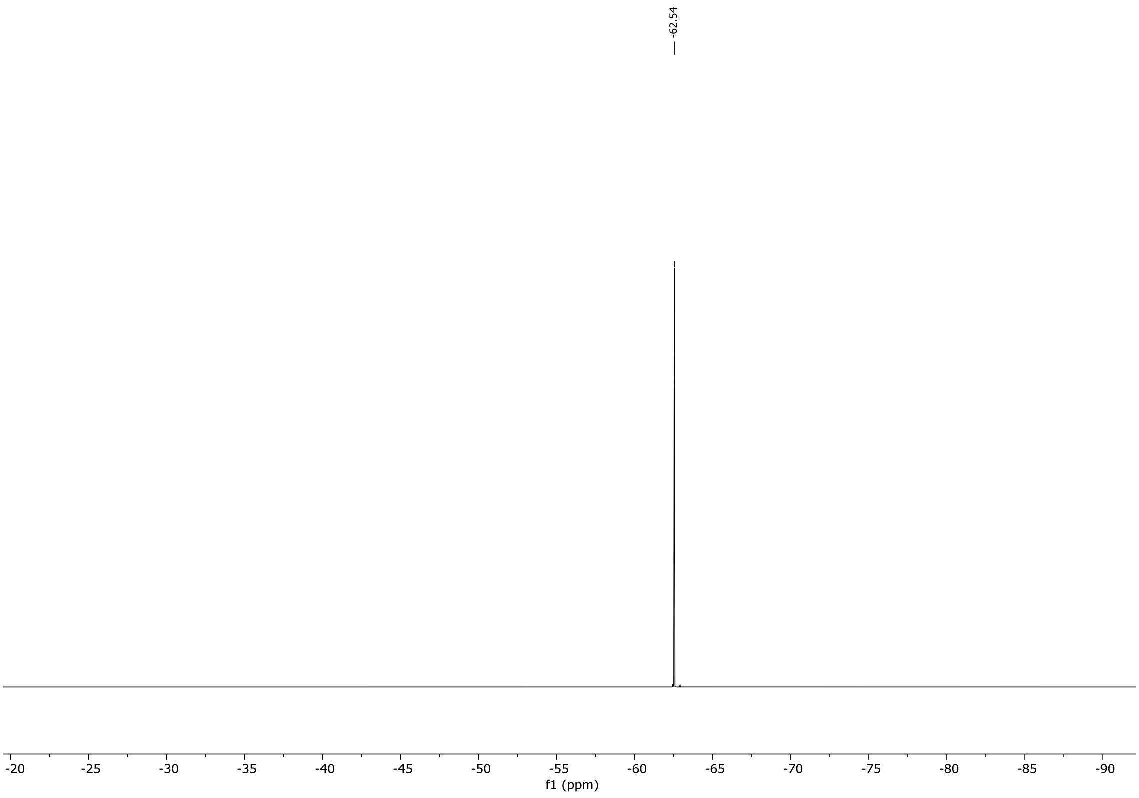

^{1}H NMR (400 MHz, $CDCl_3$) of **1ag**

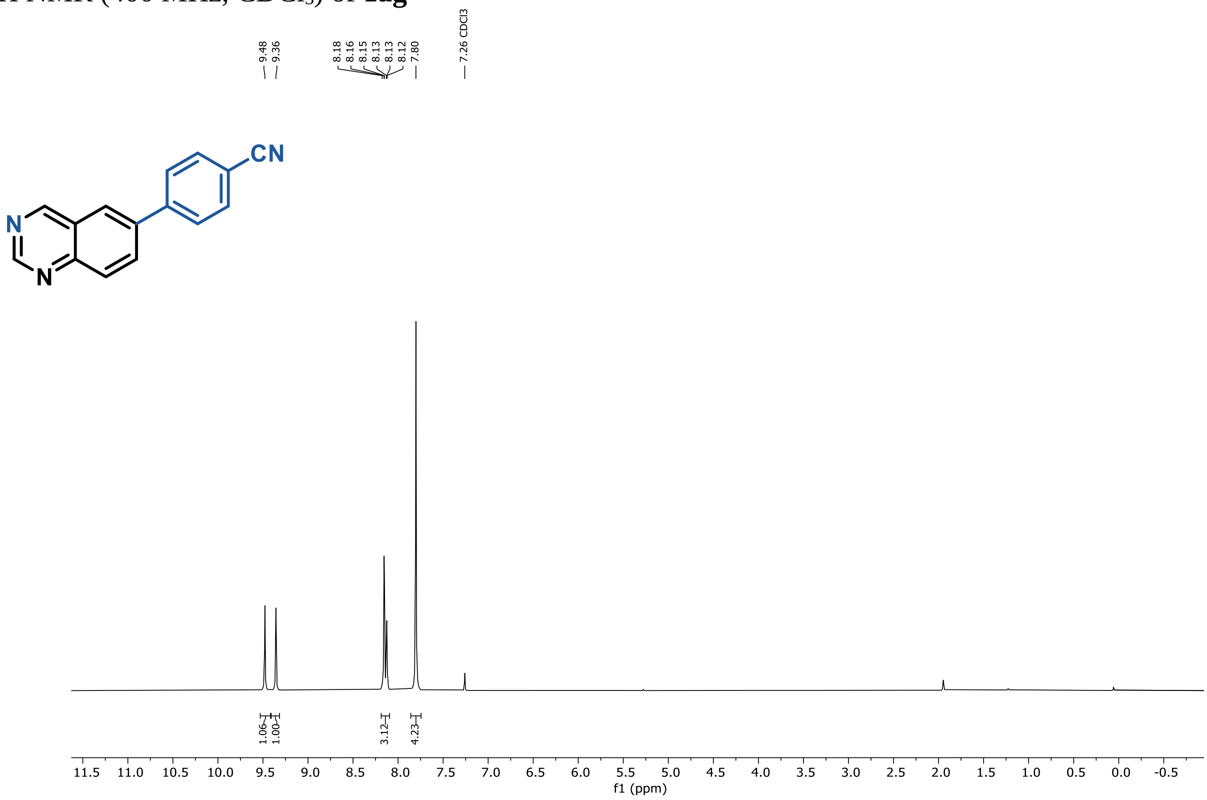

^{13}C NMR (101 MHz, $CDCl_3$) of **1ag**

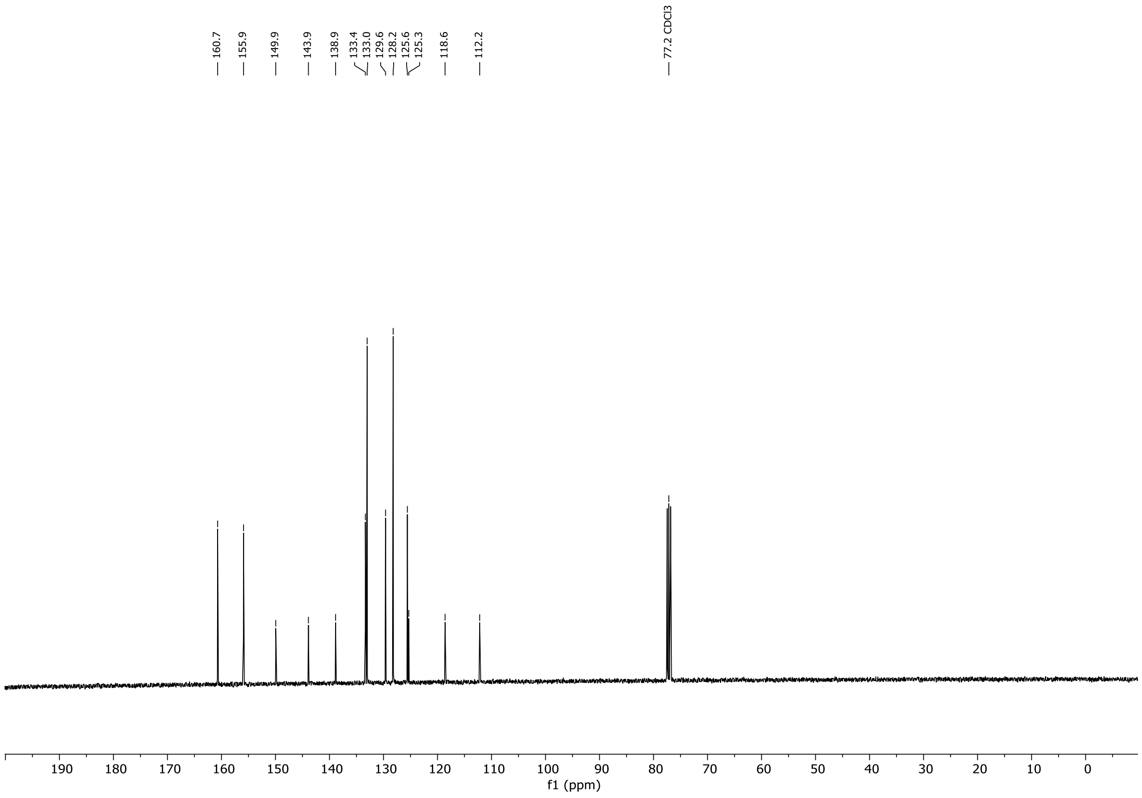

^{1}H NMR (400 MHz, $CDCl_3$) of **1ah**

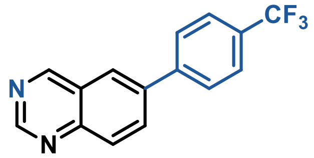

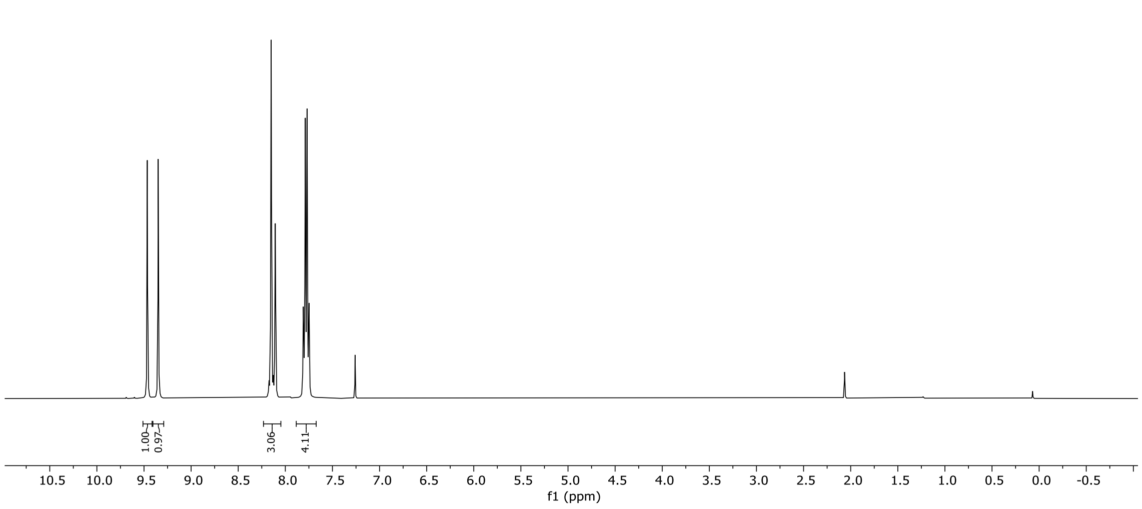

$^{13}C\{^{19}F\}$ NMR (126 MHz, $CDCl_3$) of **1ah**

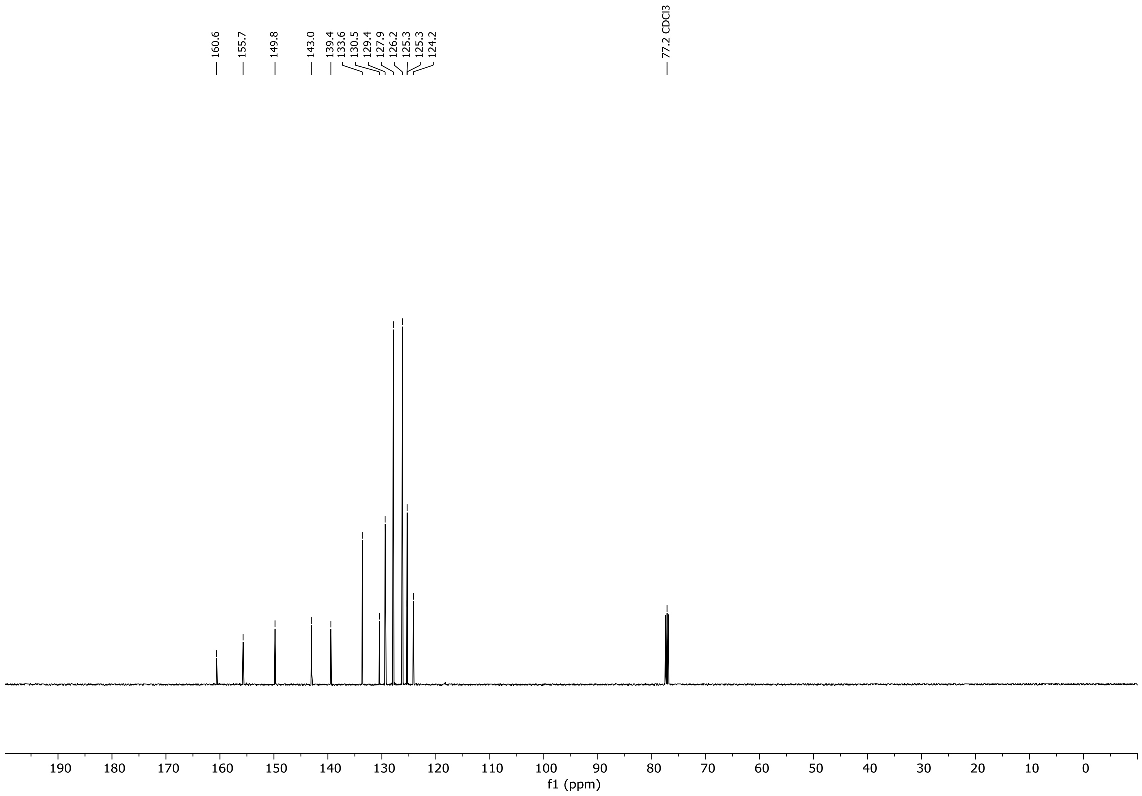

^{19}F NMR (376 MHz, $CDCl_3$) of **1ah**

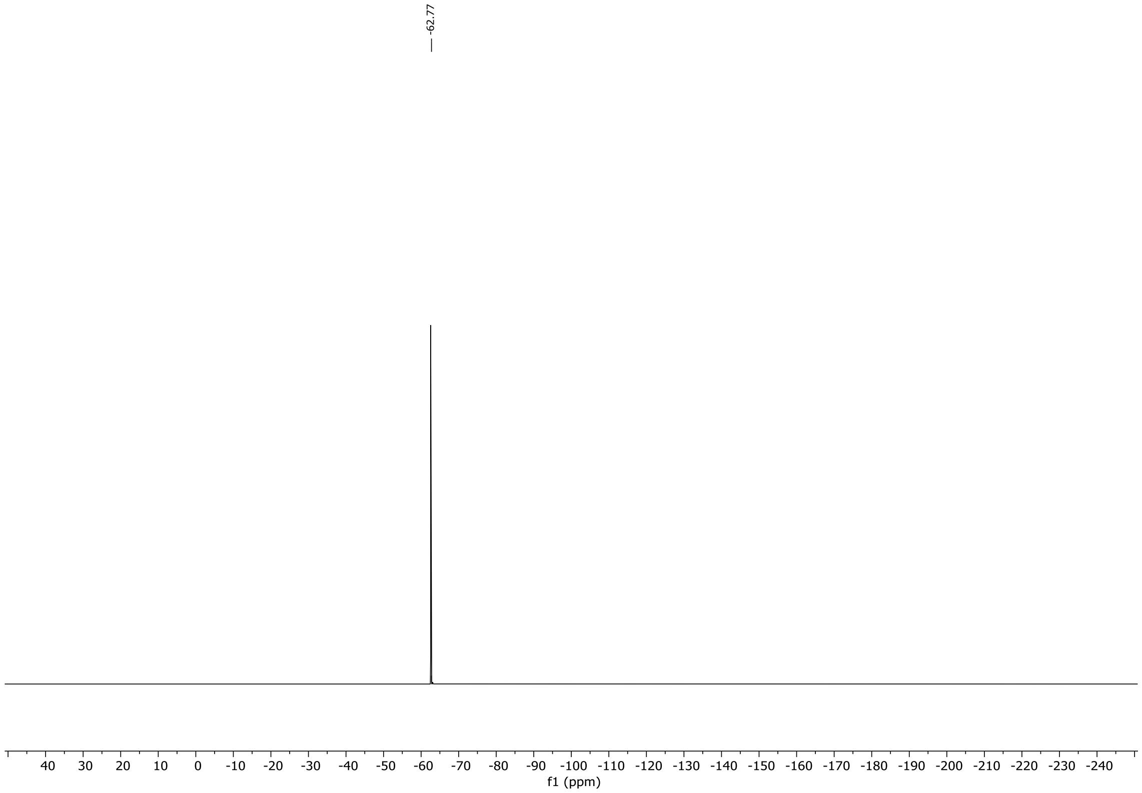

^{1}H NMR (400 MHz, $CDCl_3$) of **1ai**

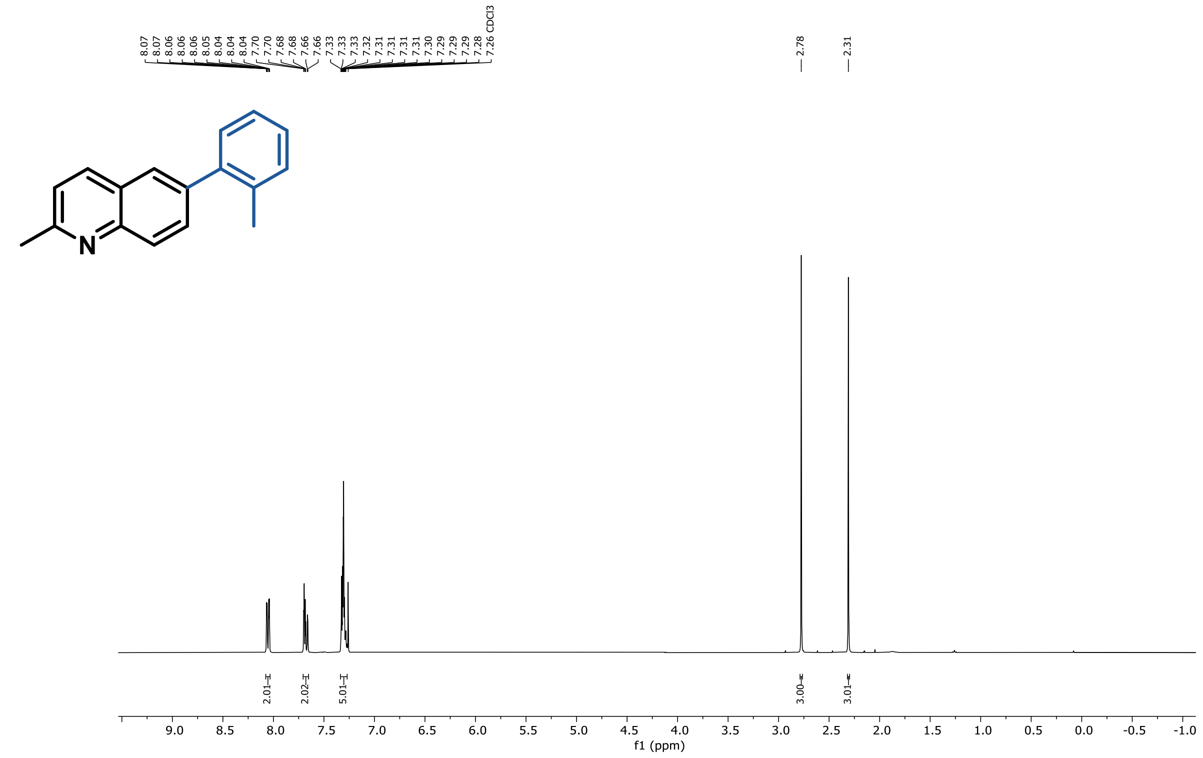

^{13}C NMR (101 MHz, $CDCl_3$) of **1ai**

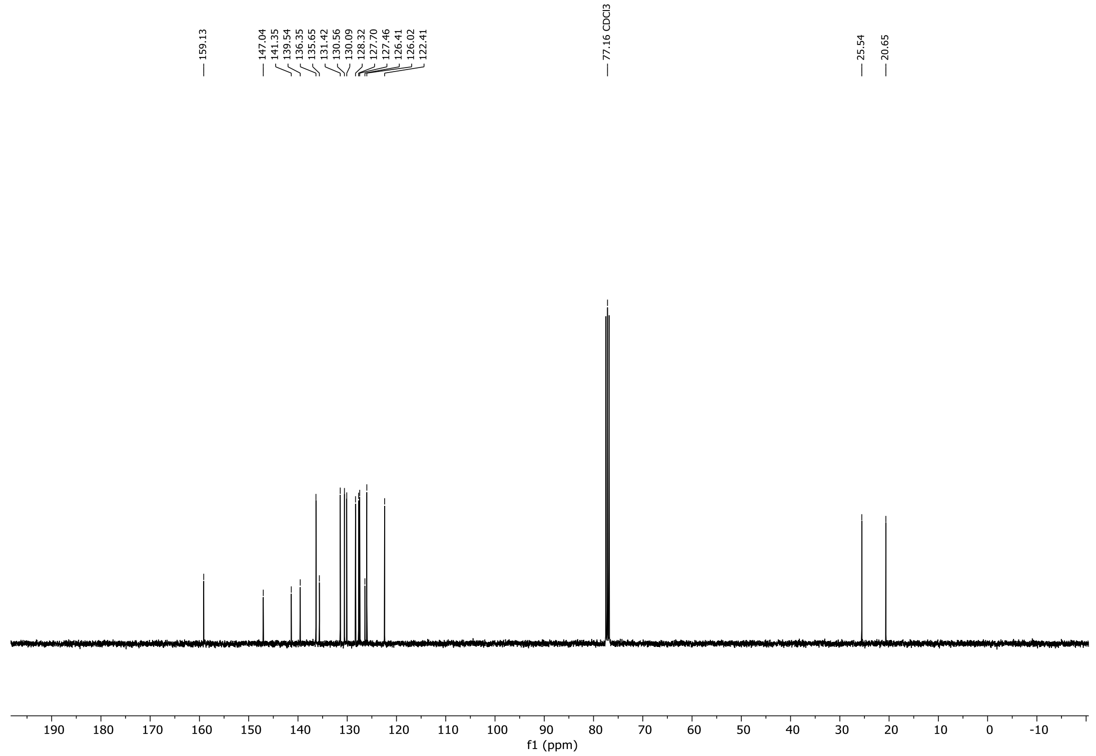

^{1}H NMR (400 MHz, $CDCl_3$) of **1aj**

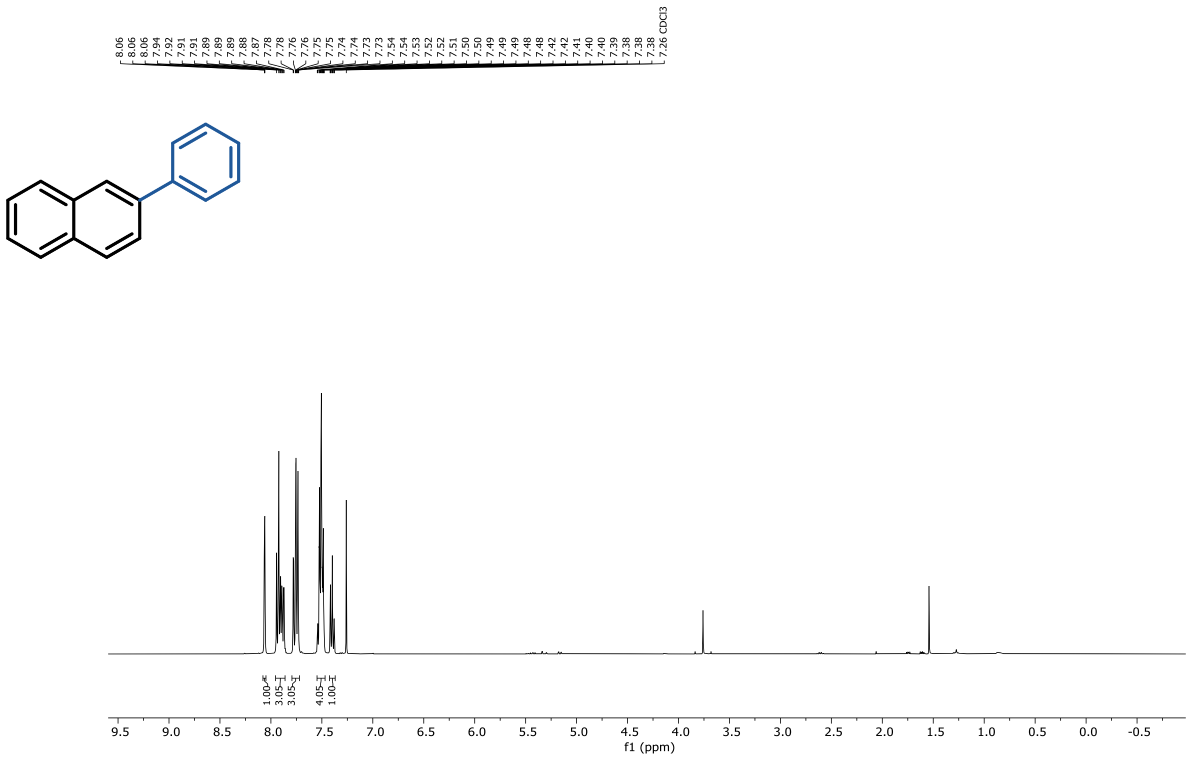

^{13}C NMR (101 MHz, $CDCl_3$) of **1aj**

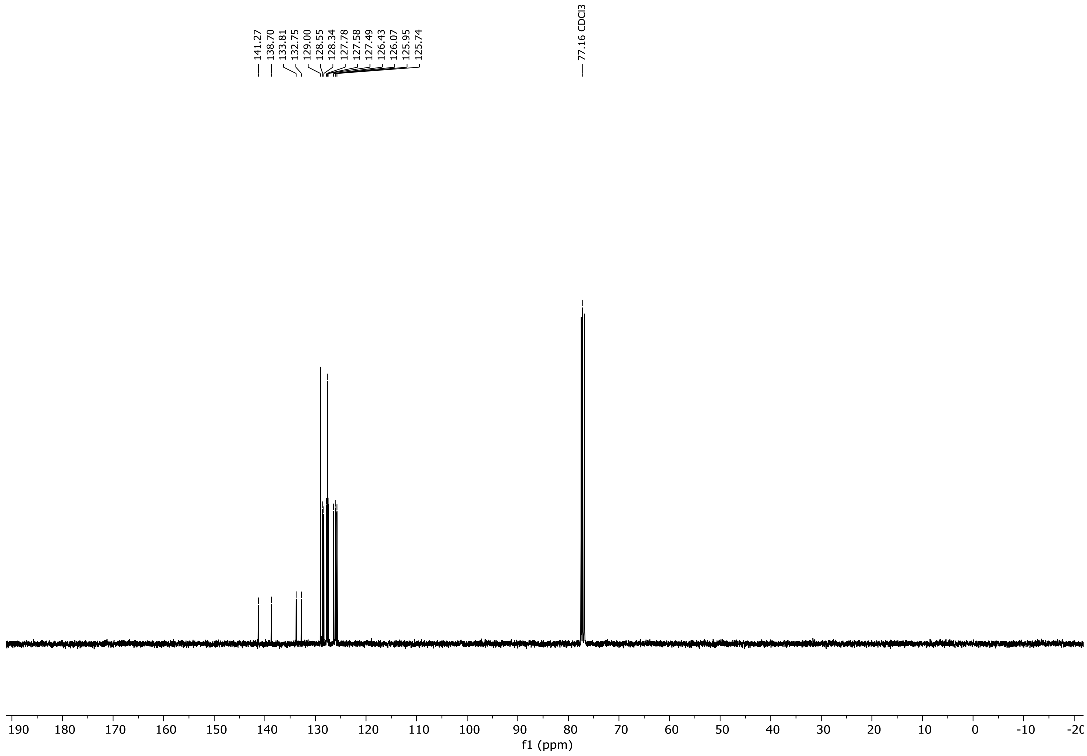

^{1}H NMR (400 MHz, $CDCl_3$) of **2a**

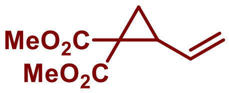

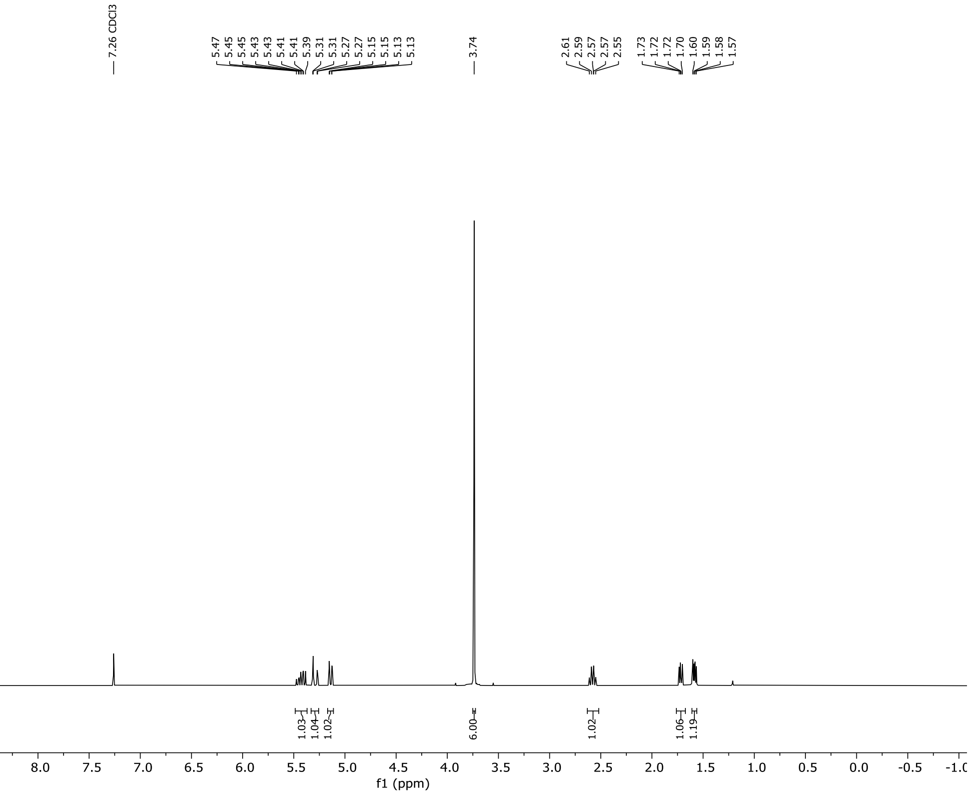

^{13}C NMR (101 MHz, $CDCl_3$) of **2a**

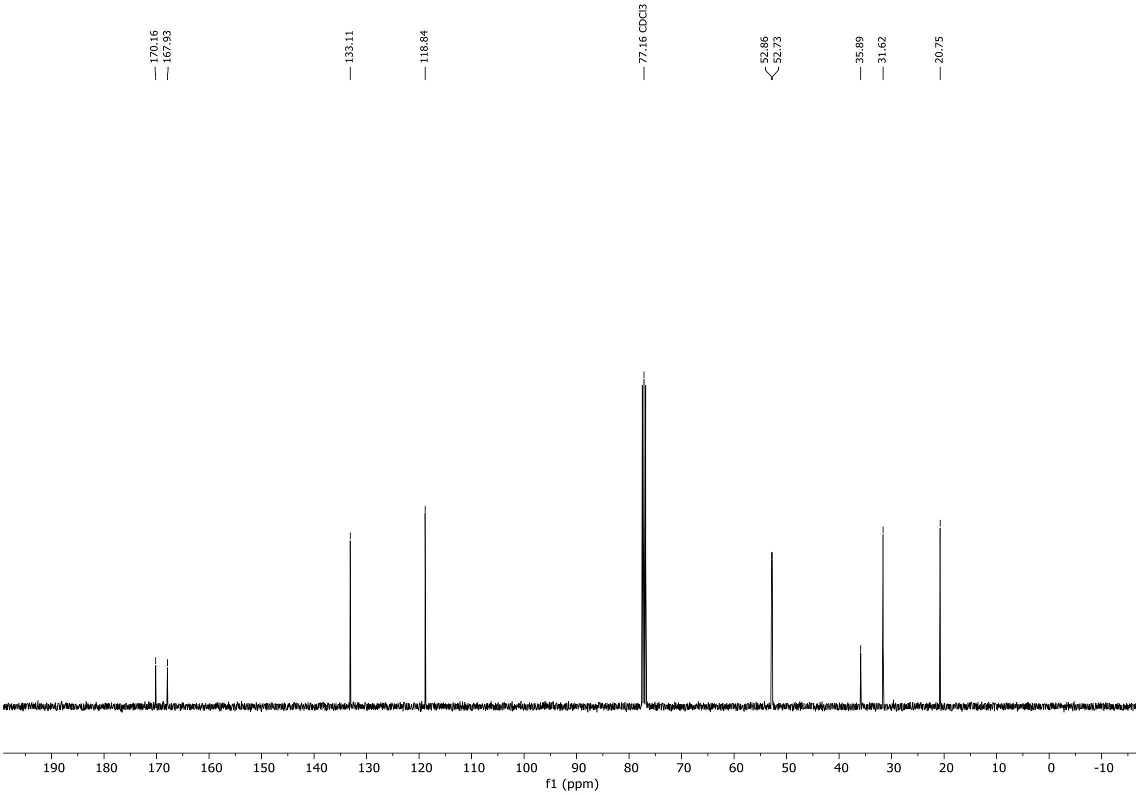

^{1}H NMR (400 MHz, $CDCl_3$) of **2b**

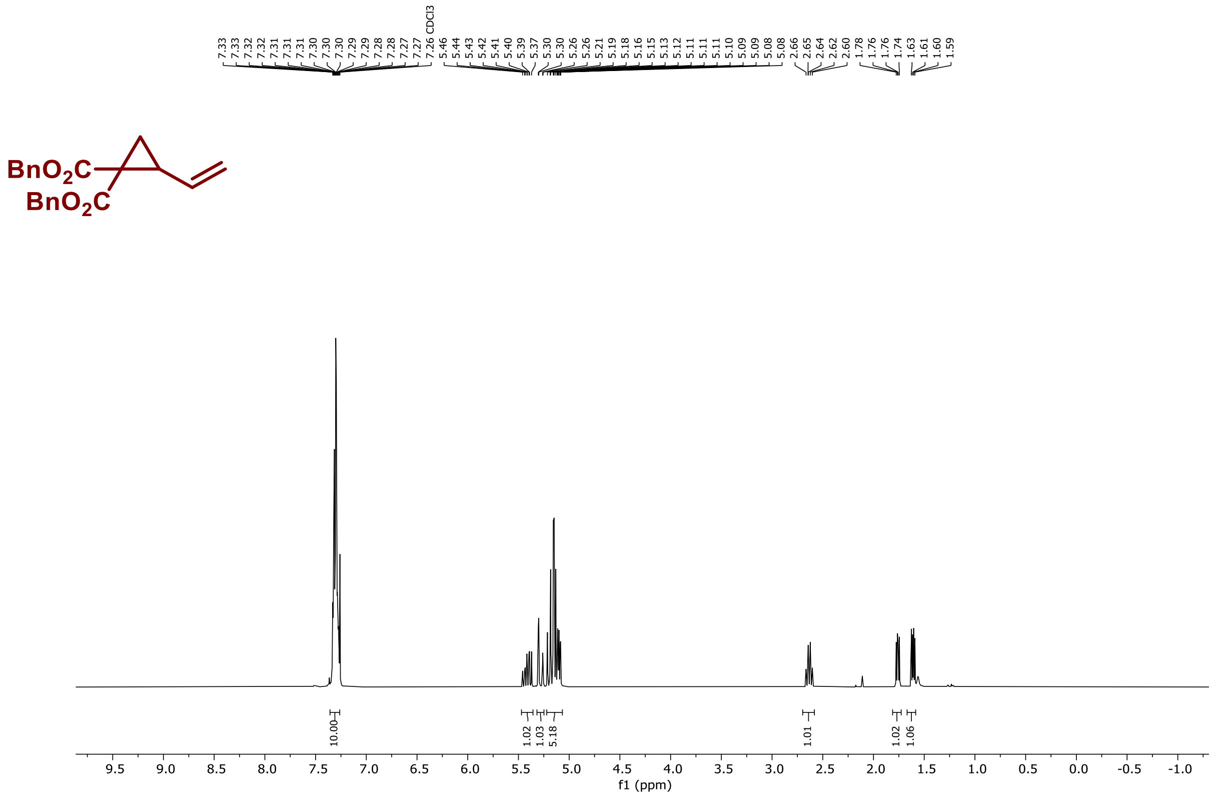

^{13}C NMR (101 MHz, $CDCl_3$) of **2b**

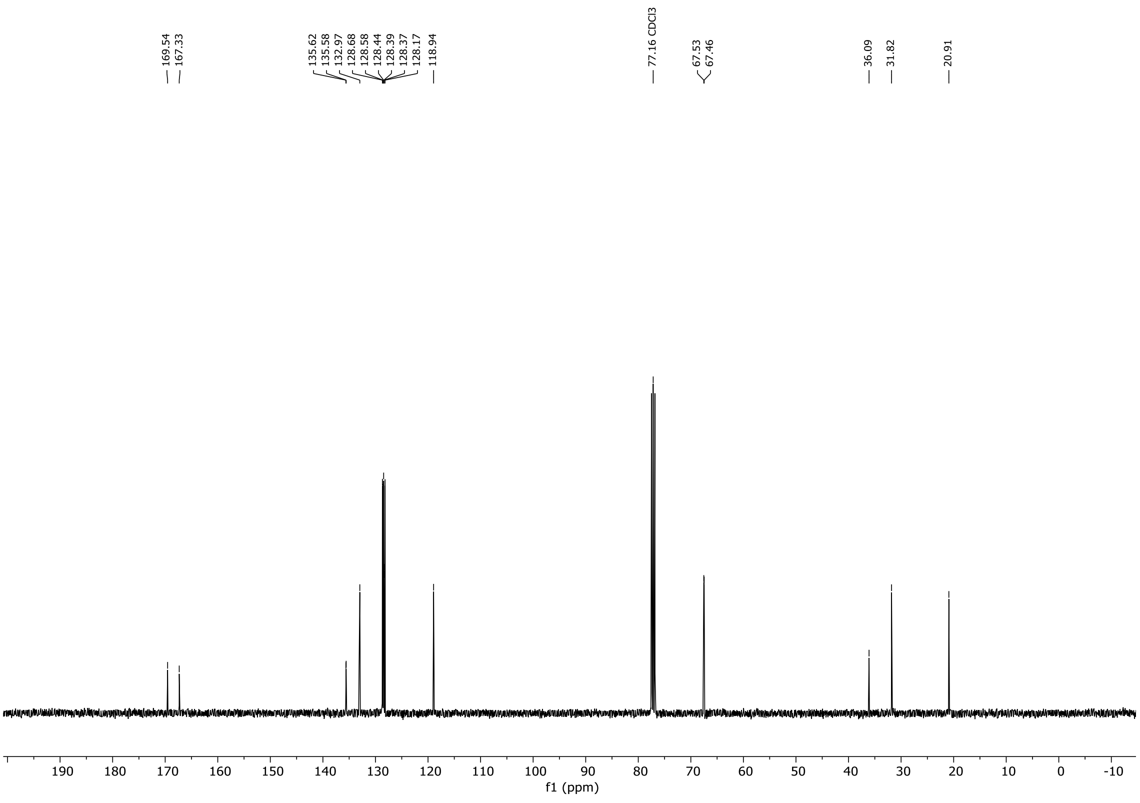

^{1}H NMR (400 MHz, $CDCl_3$) of **2c**

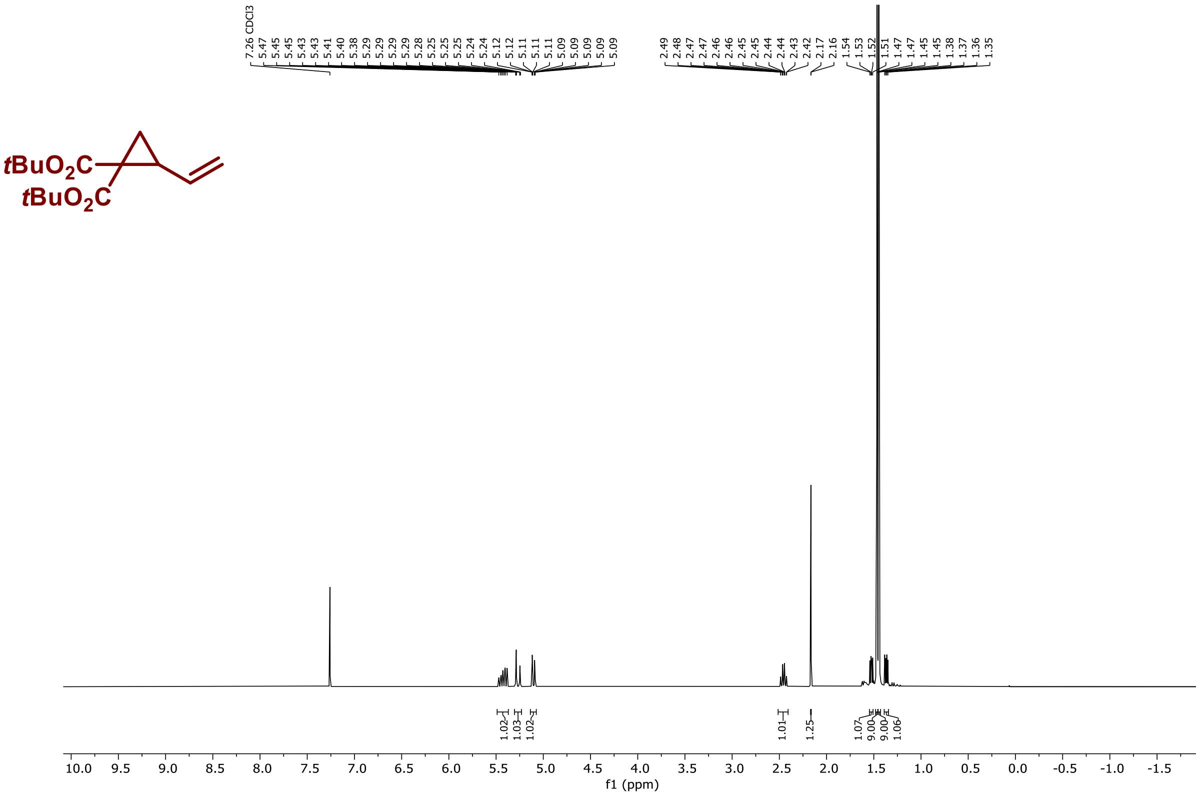

^{13}C NMR (101 MHz, $CDCl_3$) of **2c**

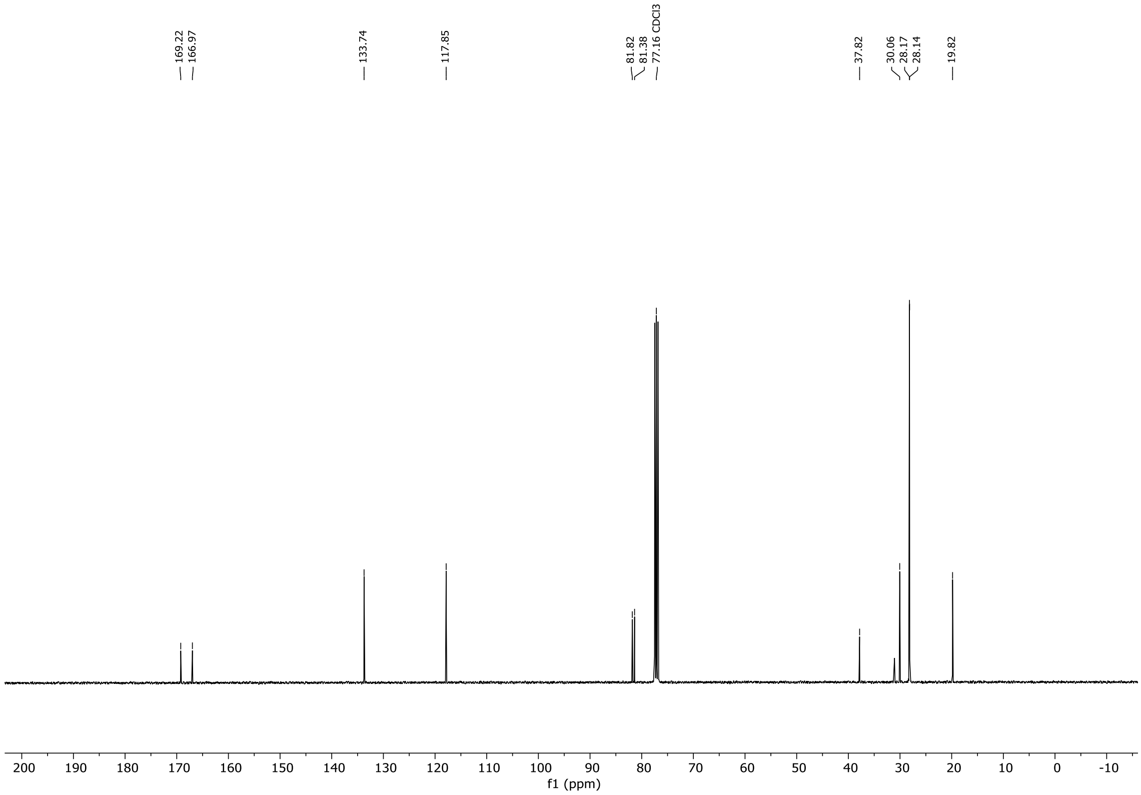

^{1}H NMR (400 MHz, $CDCl_3$) of **2d**

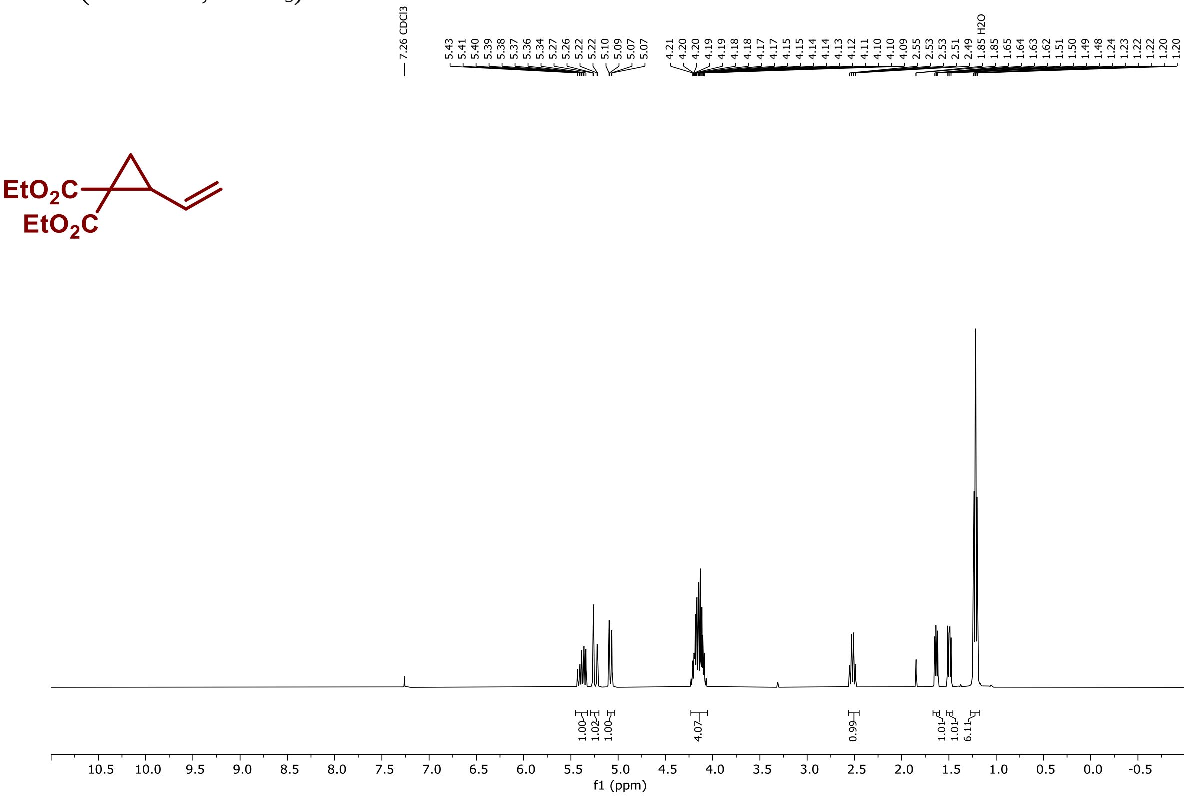

^{13}C NMR (101 MHz, $CDCl_3$) of **2d**

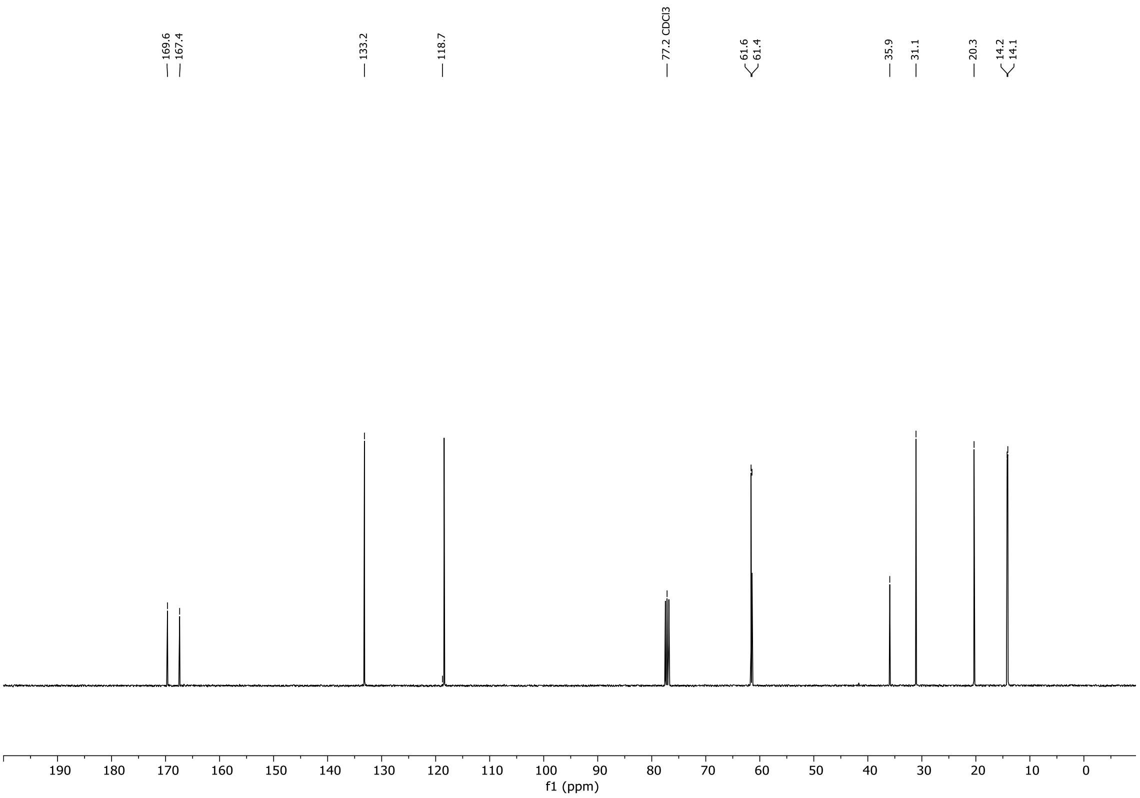

^{1}H NMR (400 MHz, $CDCl_3$) of **2f**

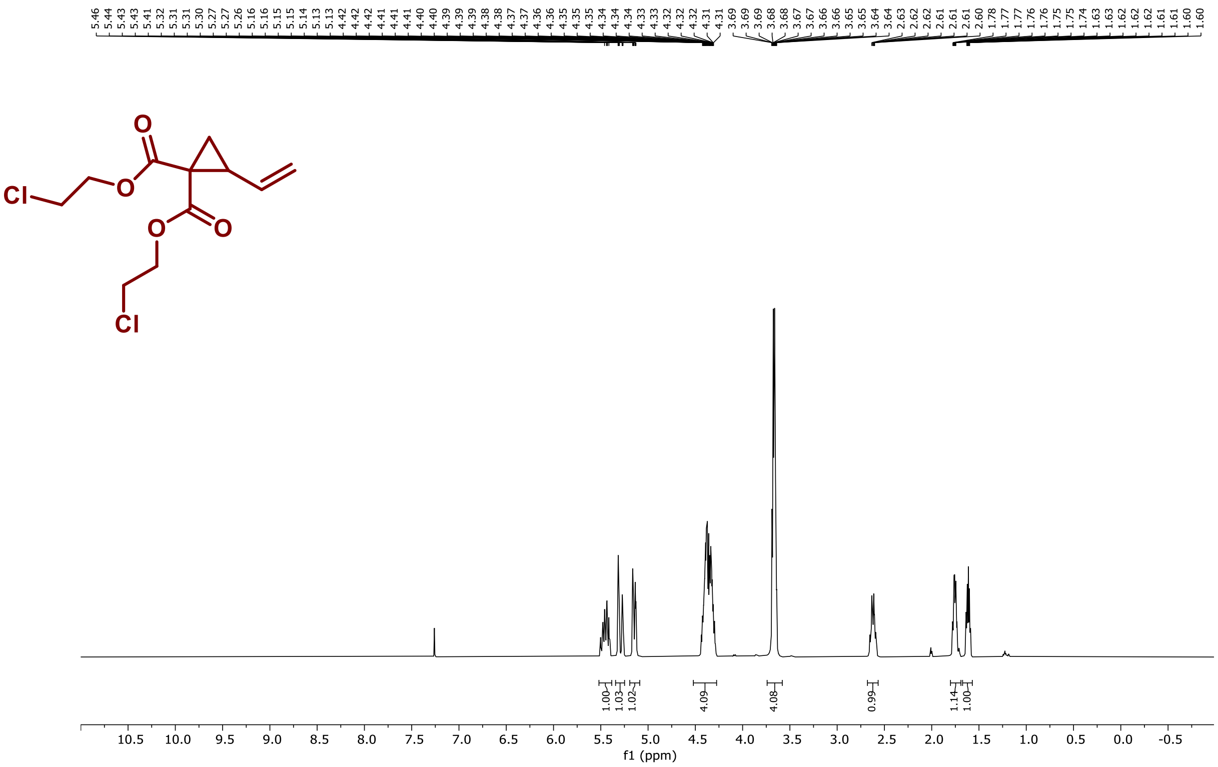

^{13}C NMR (101 MHz, $CDCl_3$) of **2f**

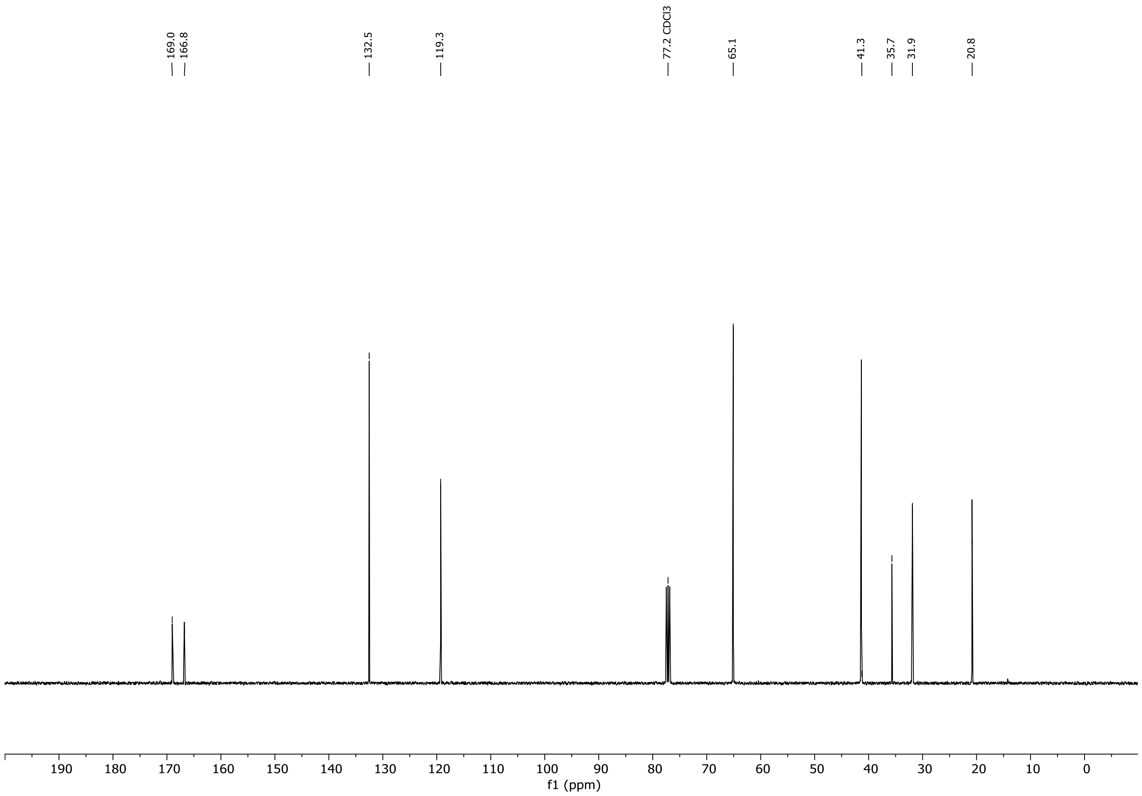

^{1}H NMR (400 MHz, $CDCl_3$) of **2g**

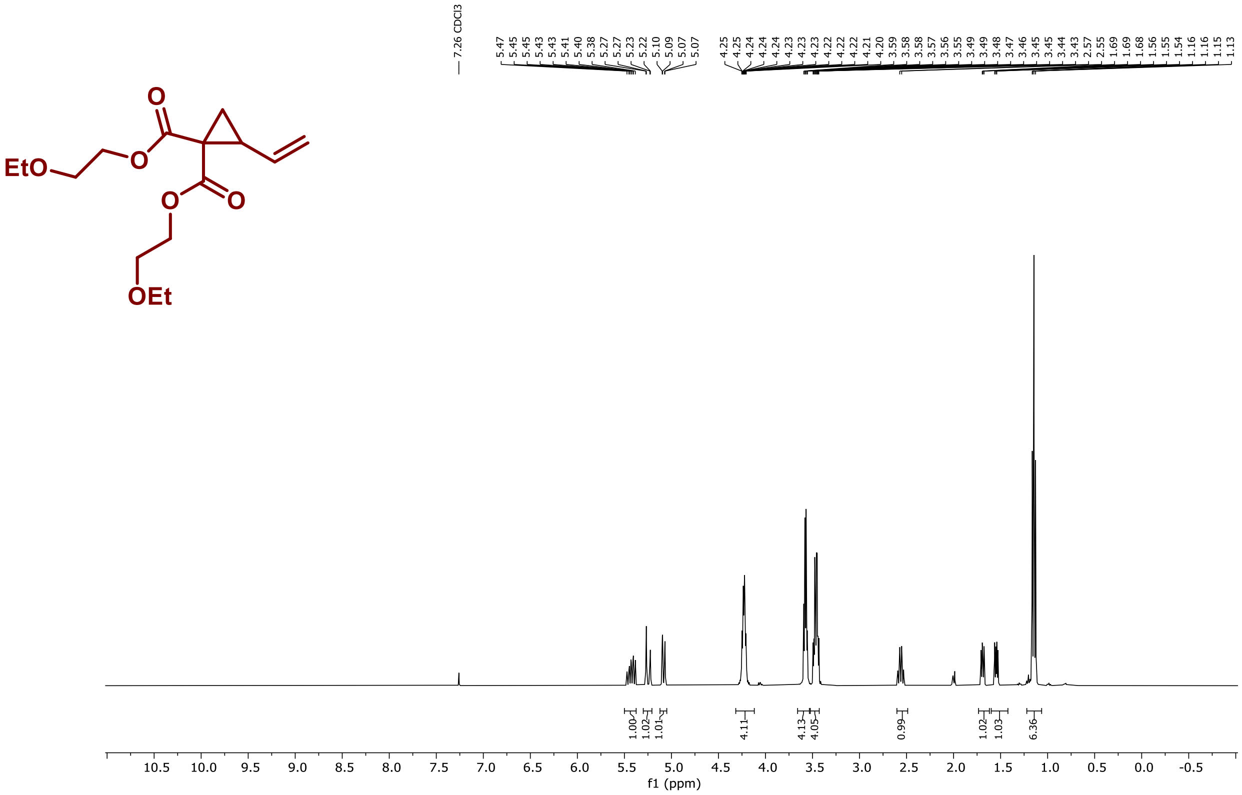

^{13}C NMR (101 MHz, $CDCl_3$) of **2g**

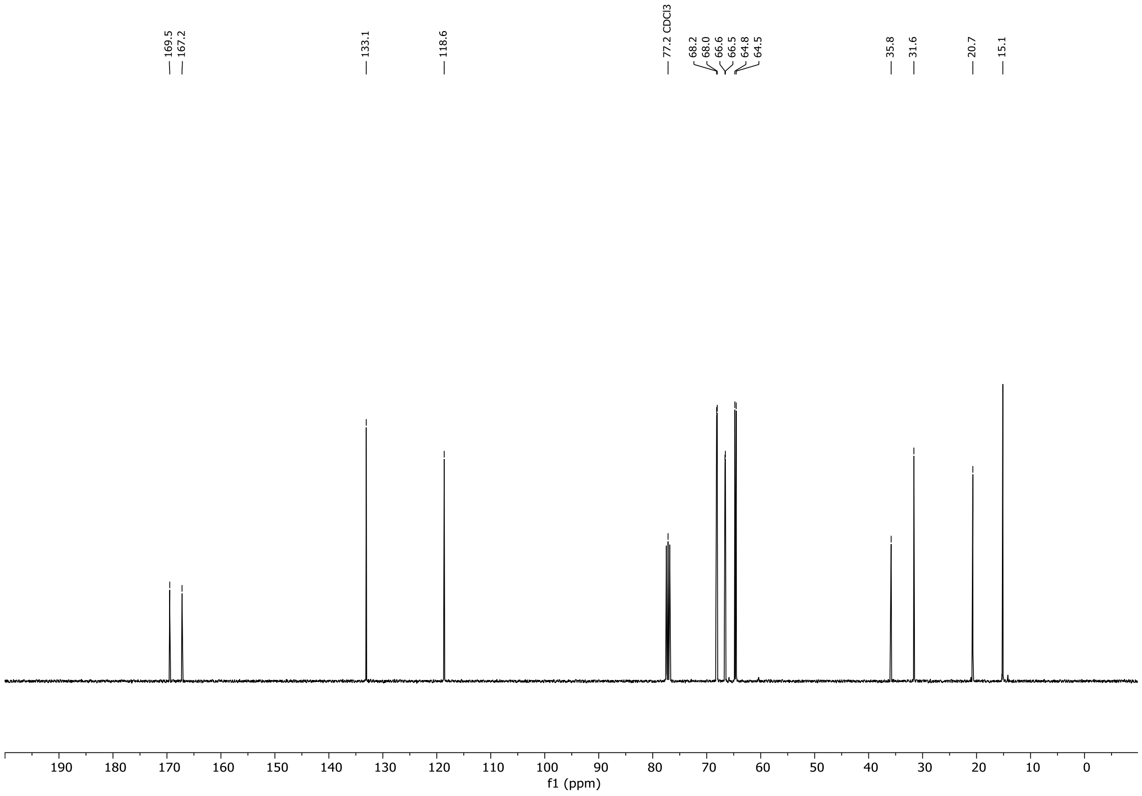

^{1}H NMR (400 MHz, $CDCl_3$) of **2h**

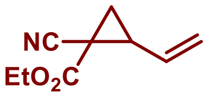

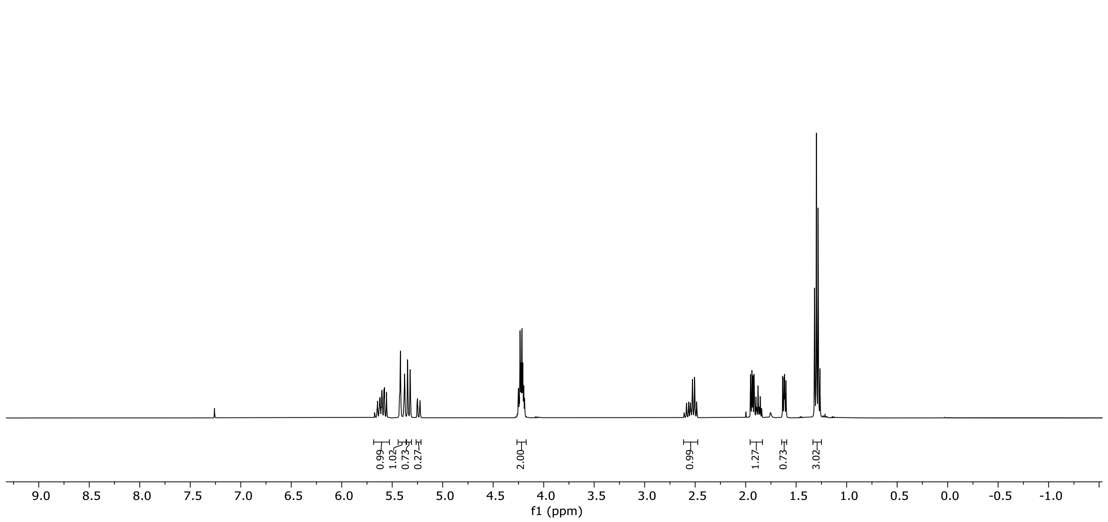

^{13}C NMR (101 MHz, $CDCl_3$) of **2h**

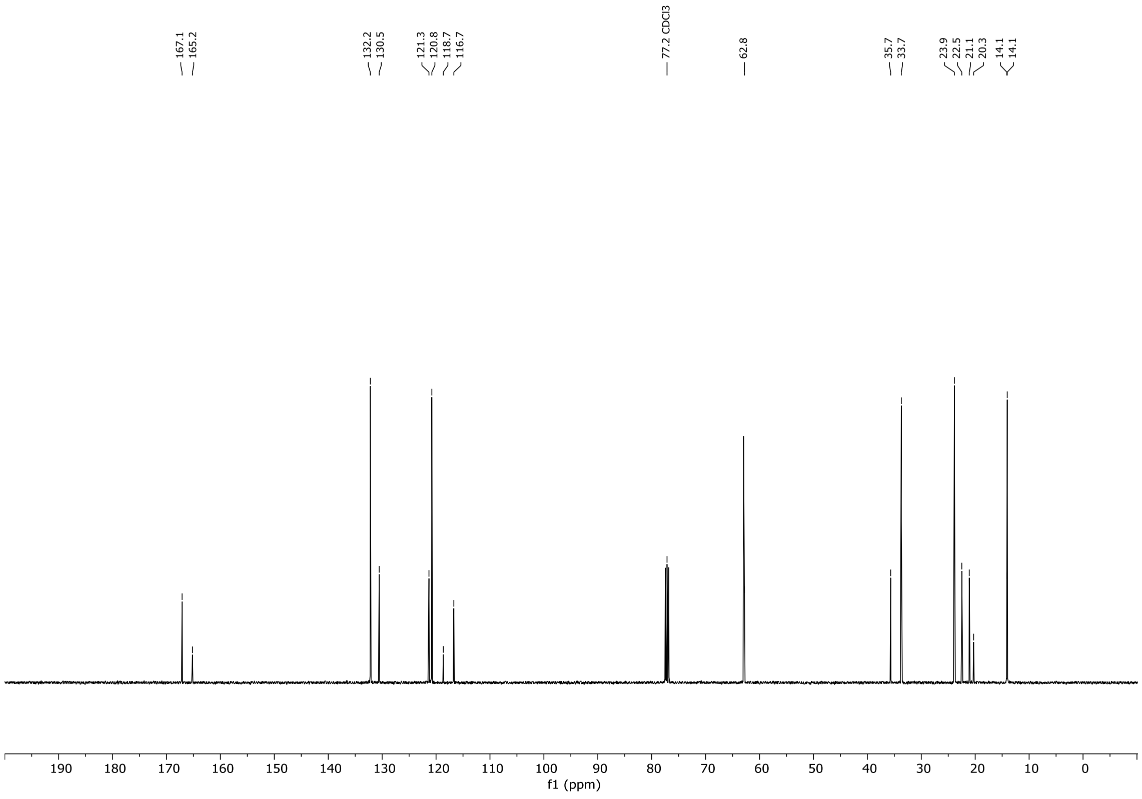

^{1}H NMR (400 MHz, $CDCl_3$) of **2i**

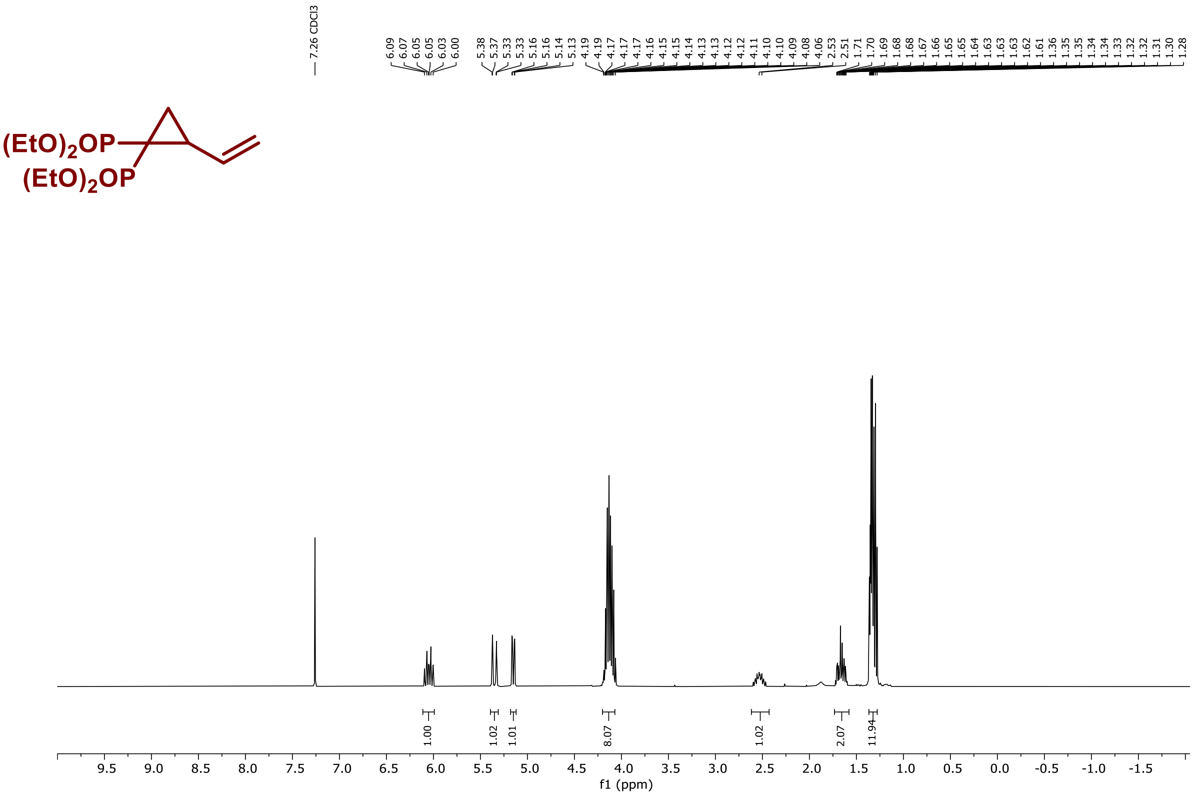

^{13}C NMR (101 MHz, $CDCl_3$) of **2i**

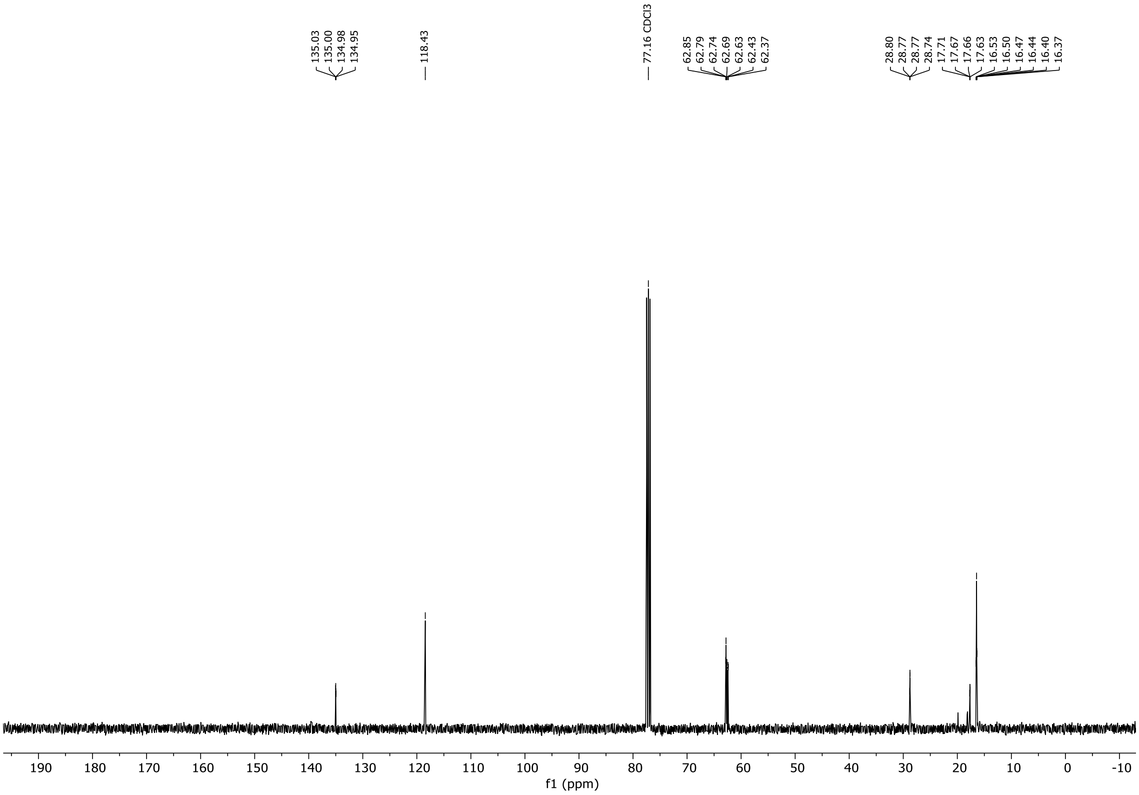

^{31}P NMR (162 MHz, $CDCl_3$) of **2i**

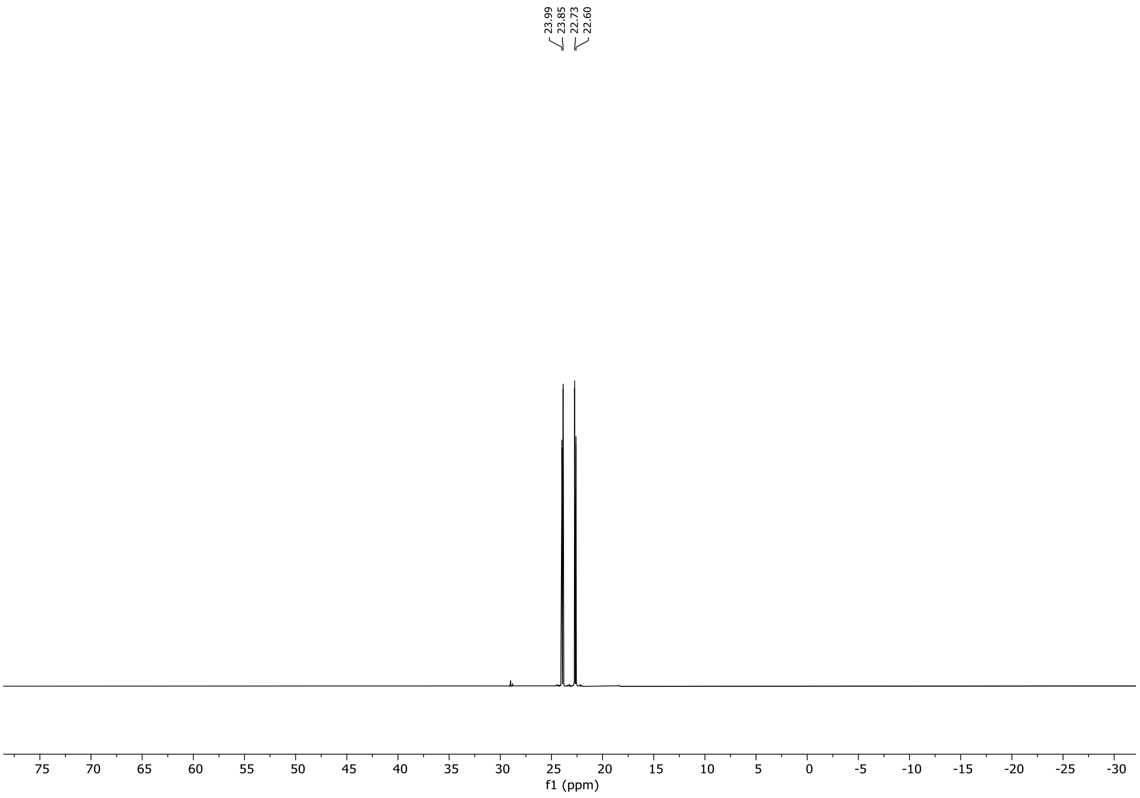

^{1}H NMR (400 MHz, $CDCl_3$) of **S6**

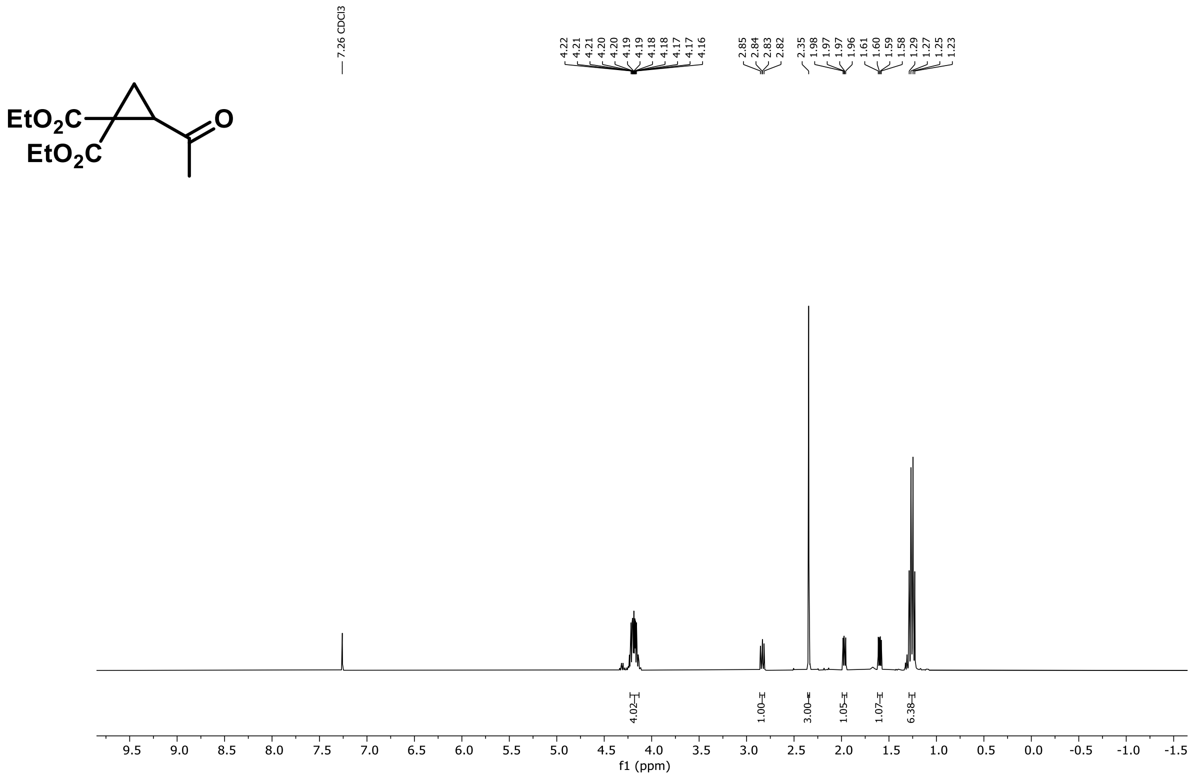

^{13}C NMR (101 MHz, $CDCl_3$) of **S6**

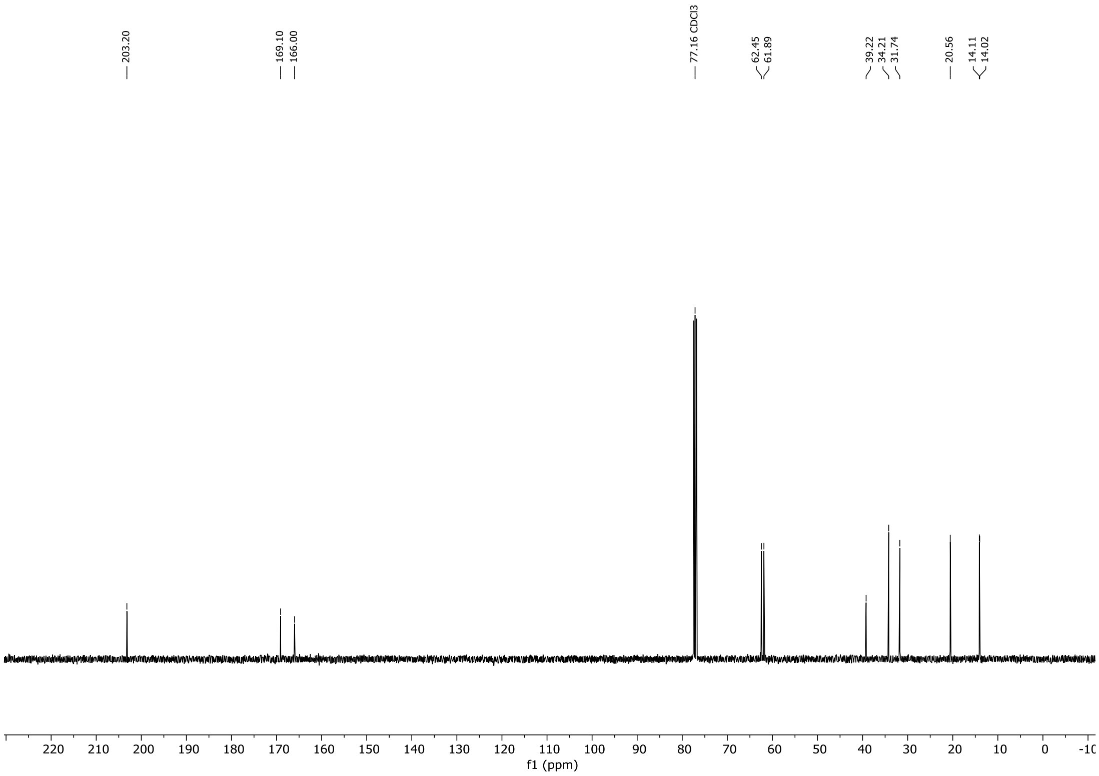

^{1}H NMR (400 MHz, $CDCl_3$) of **2j**

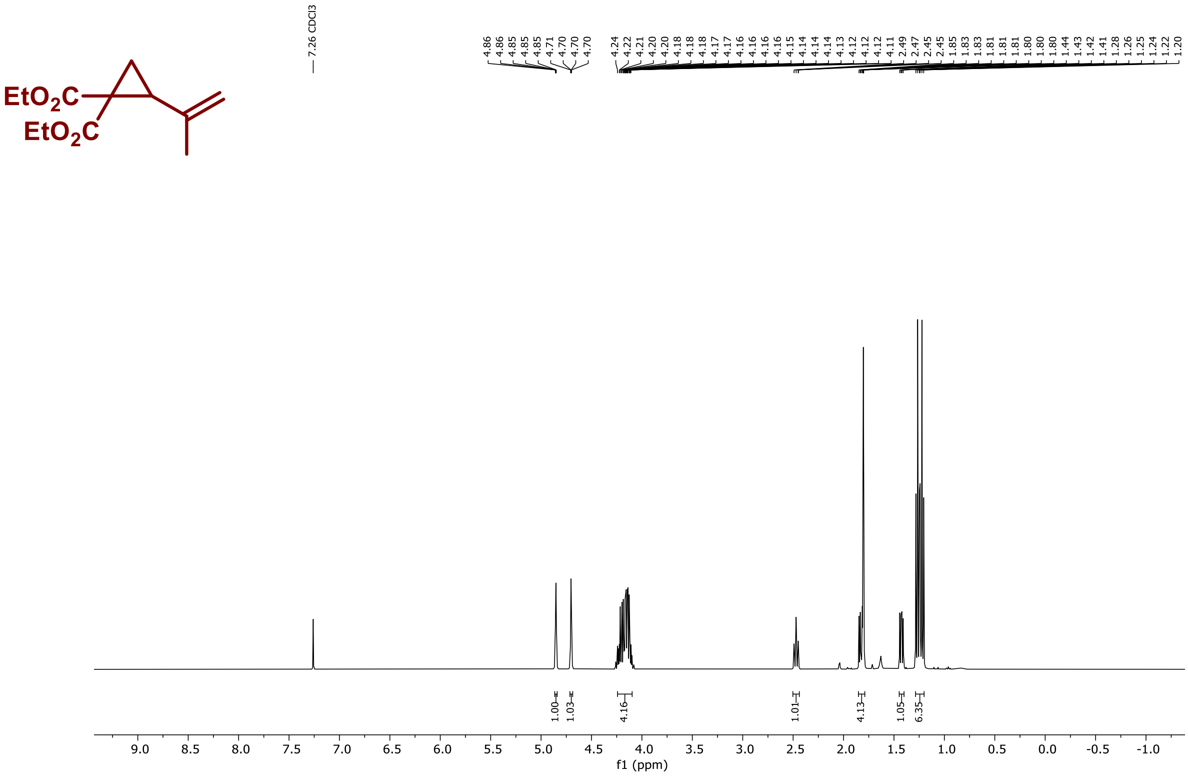

^{13}C NMR (101 MHz, $CDCl_3$) of **2j**

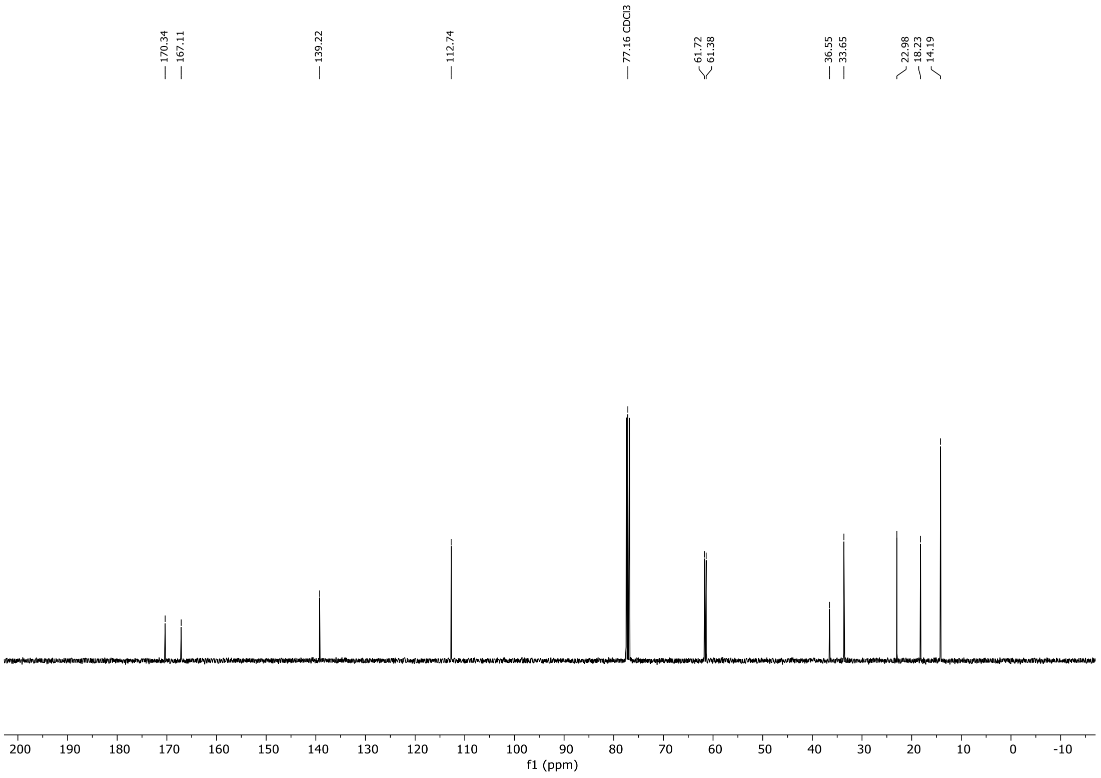

^{1}H NMR (400 MHz, $CDCl_3$) of **3a**

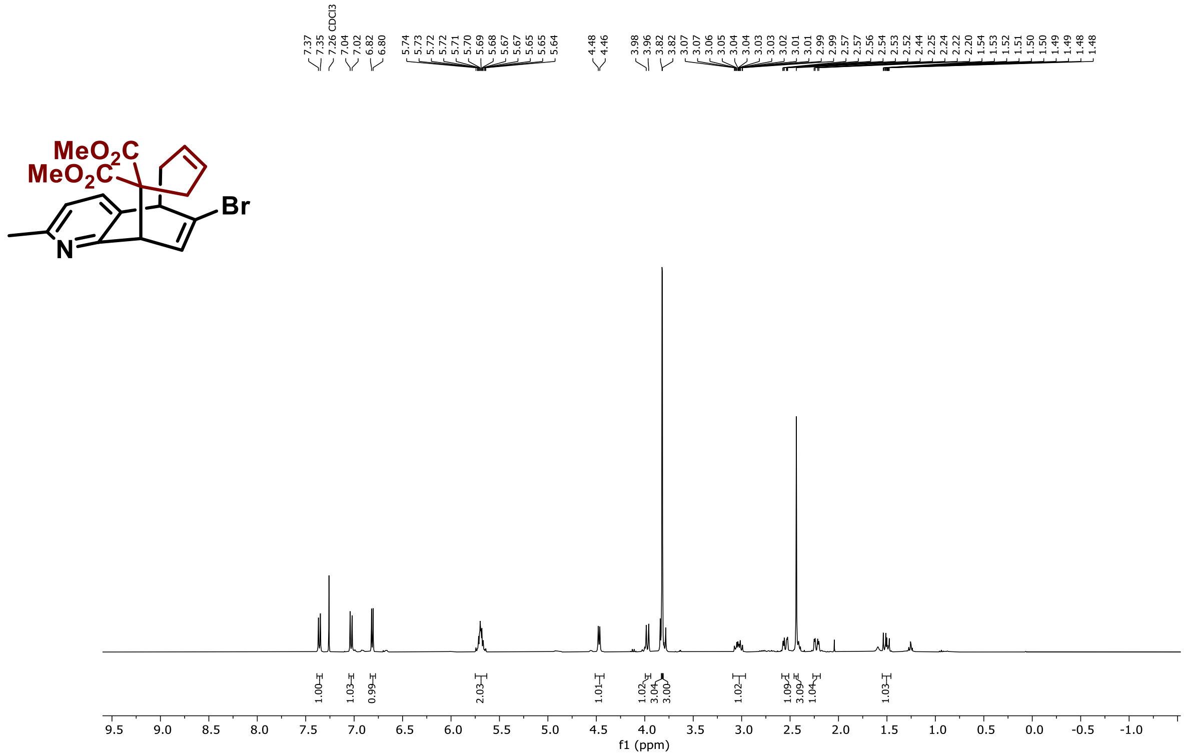

^{13}C NMR (101 MHz, $CDCl_3$) of **3a**

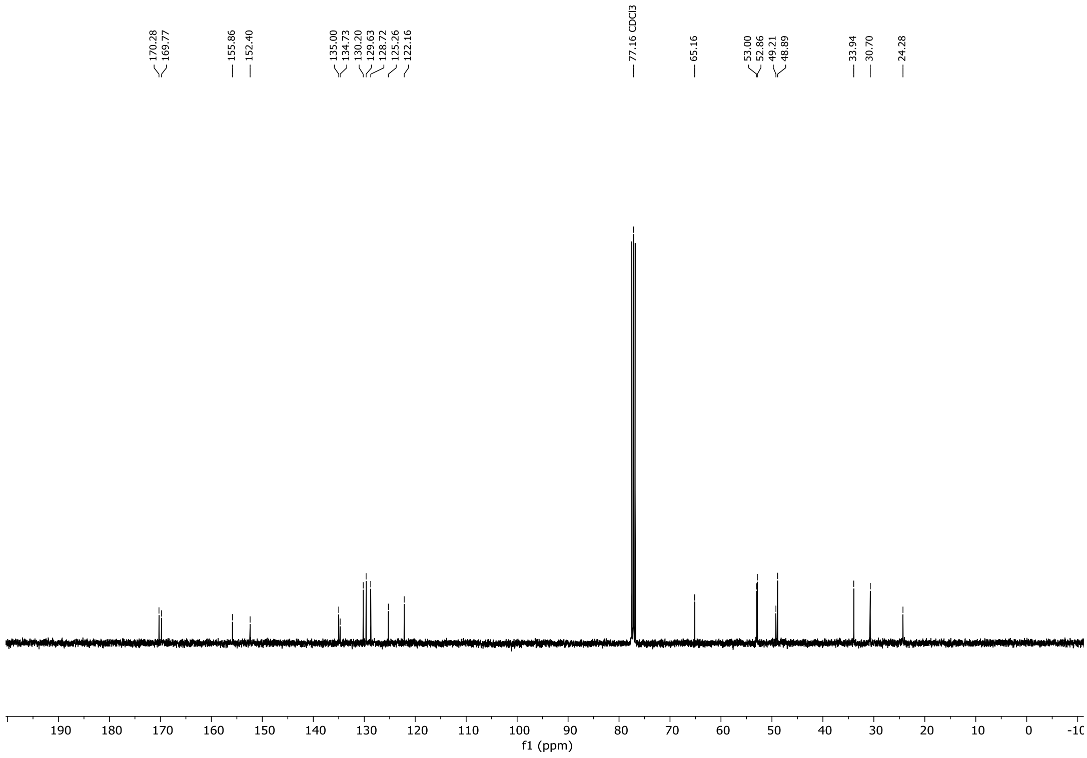

^{1}H NMR (400 MHz, $CDCl_3$) of **4a**

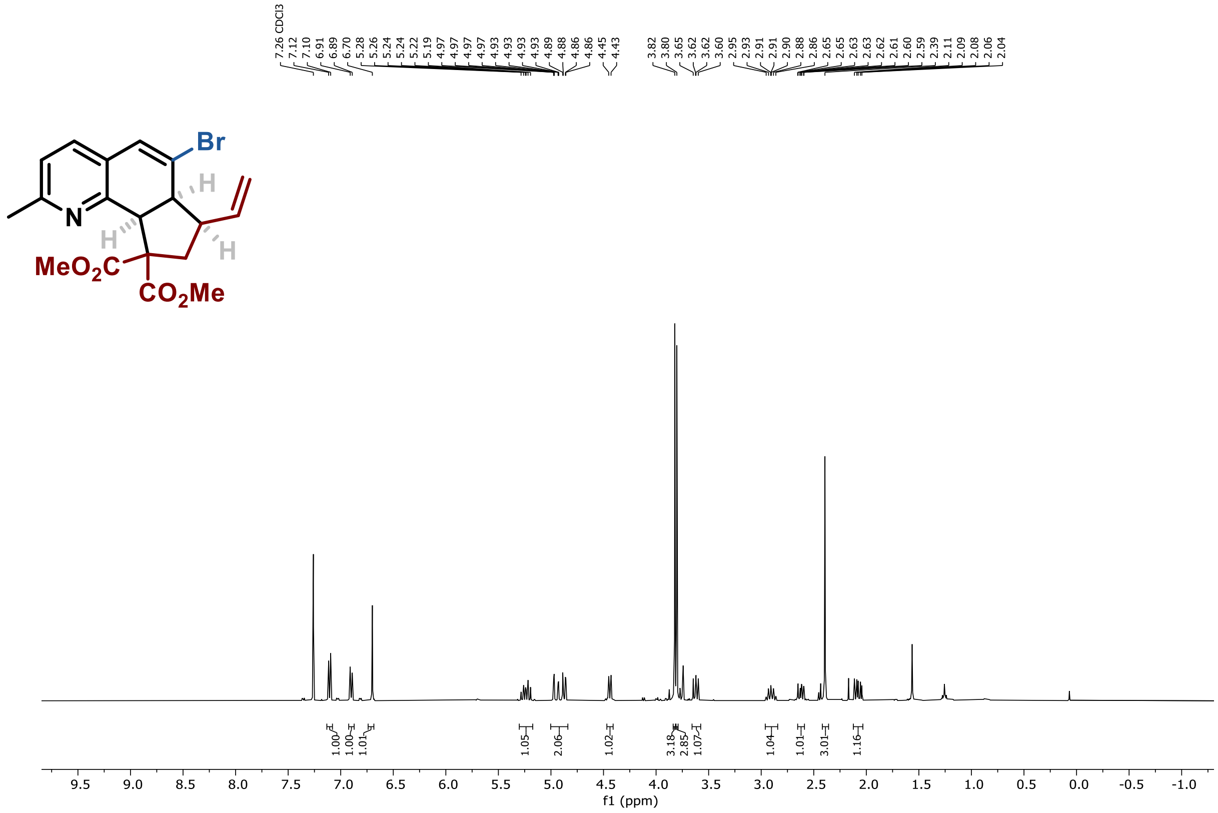

^{13}C NMR (151 MHz, $CDCl_3$) of **4a**

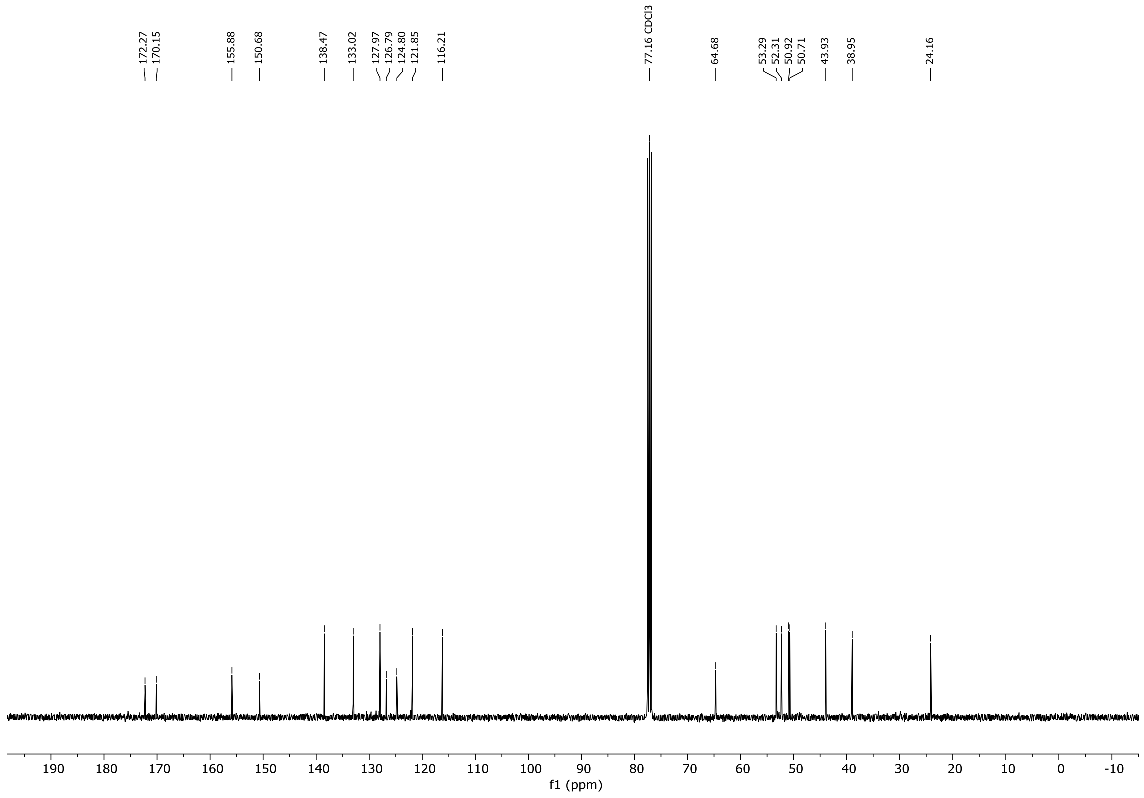

^{1}H NMR (400 MHz, $CDCl_3$) of **4e**

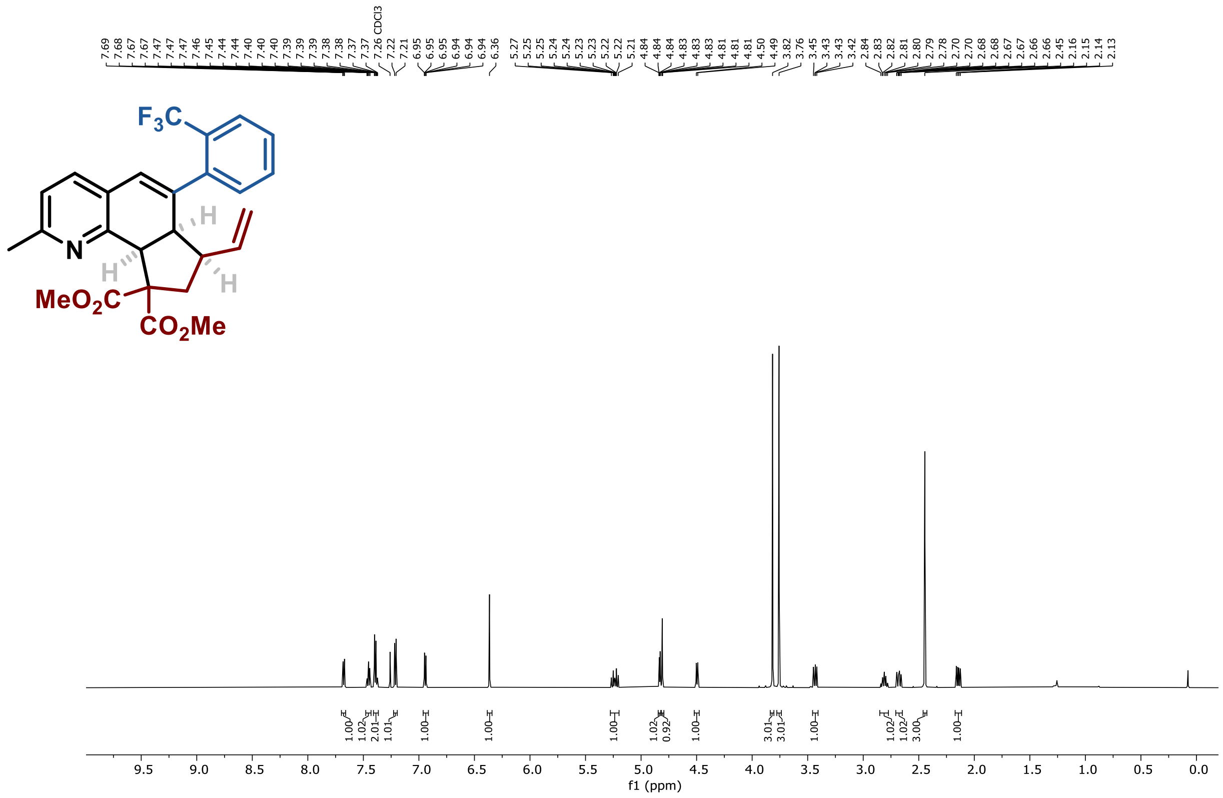

$^{13}C\{^{19}F\}$ NMR (151 MHz, $CDCl_3$) of **4e**

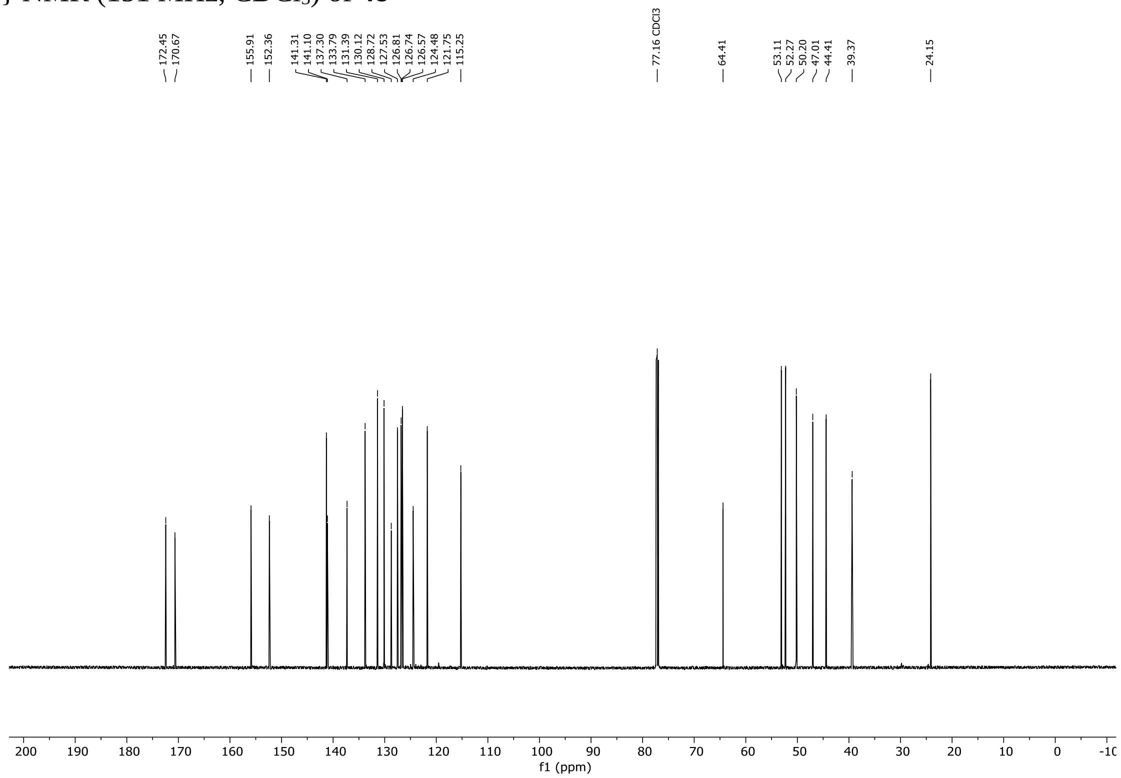

^{19}F NMR (564 MHz, $CDCl_3$) **4e**

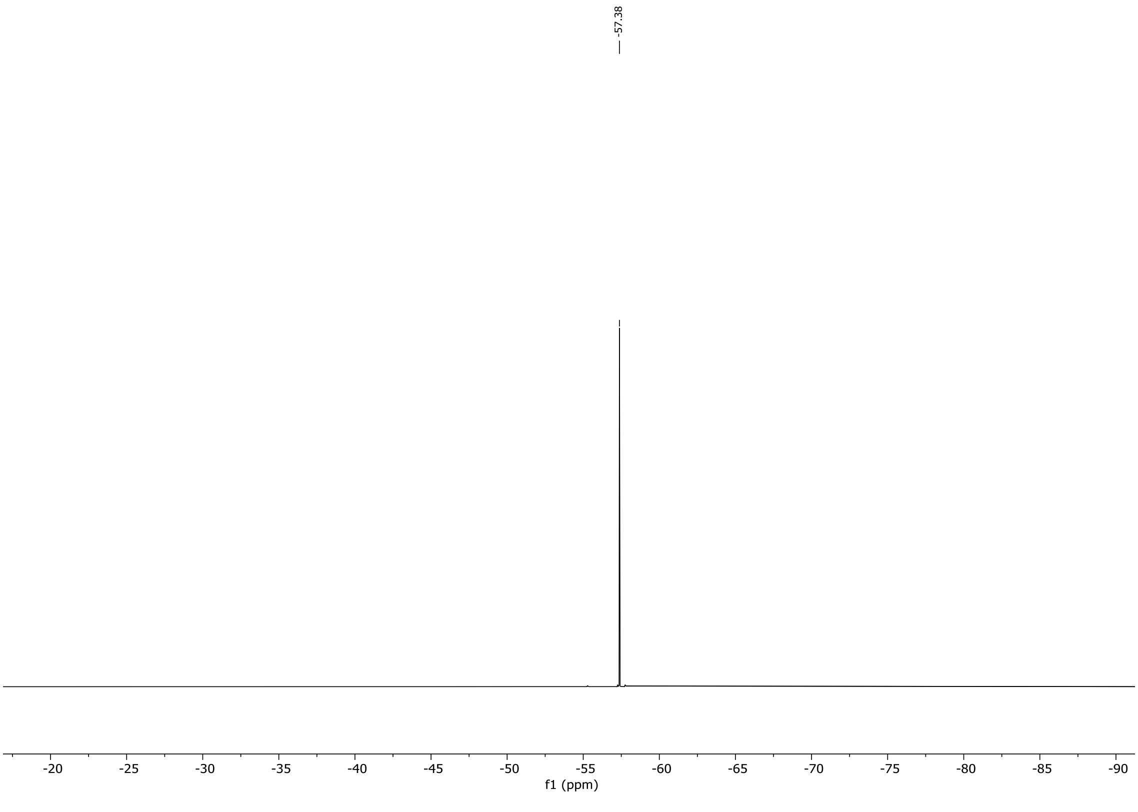

^{1}H NMR (400 MHz, $CDCl_3$) of **5e**

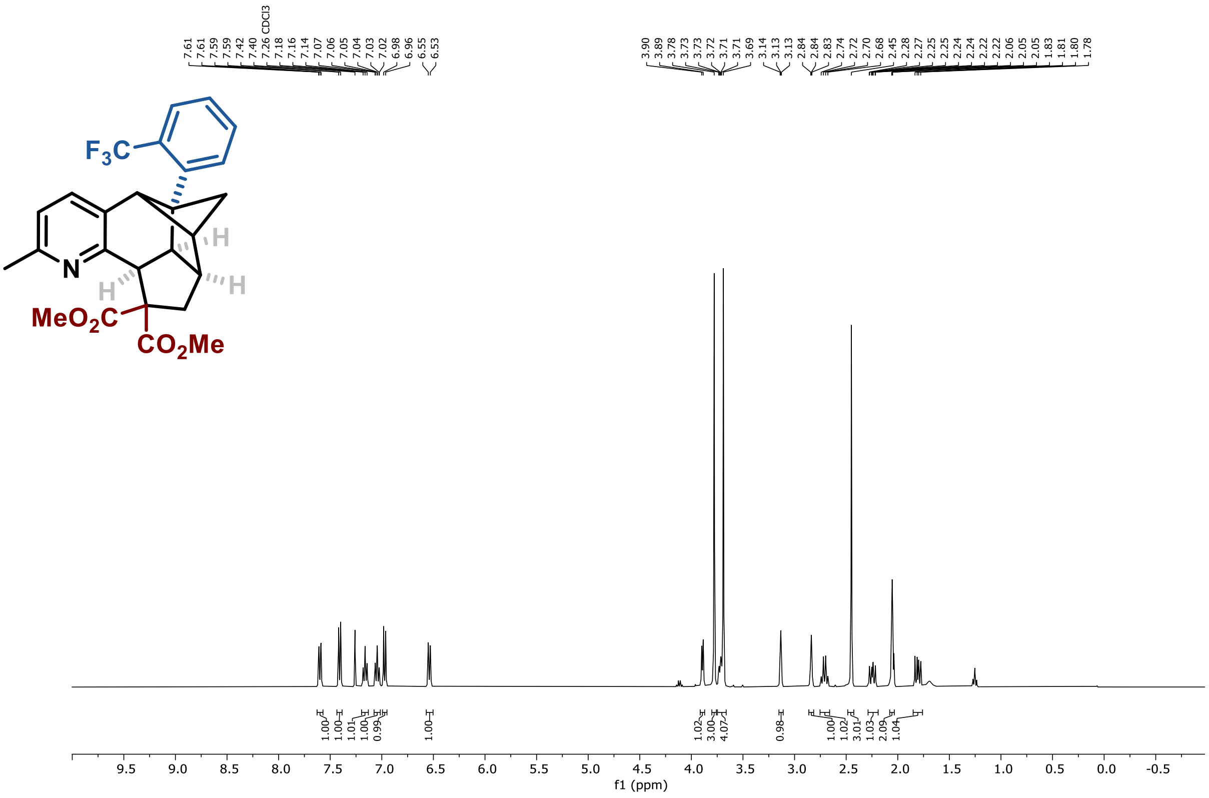

$^{13}C\{^{19}F\}$ NMR (126 MHz, $CDCl_3$) of **5e**

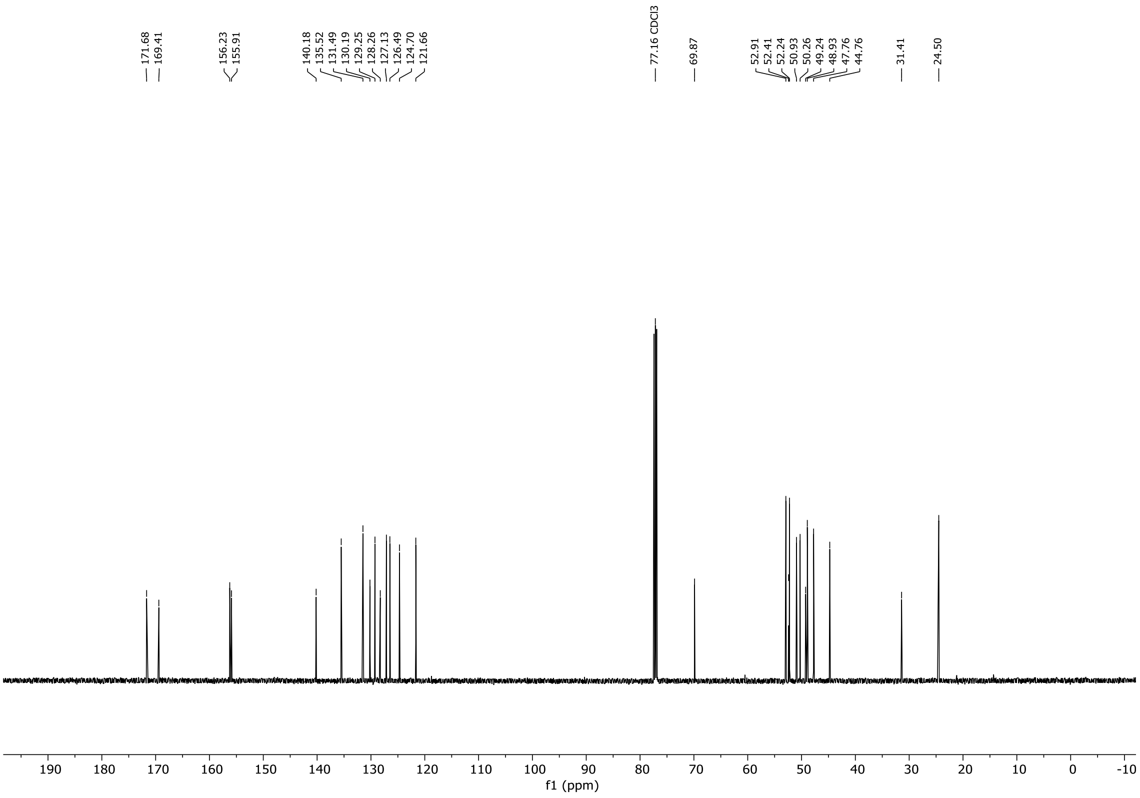

^{19}F NMR (376 MHz, $CDCl_3$) of **5e**

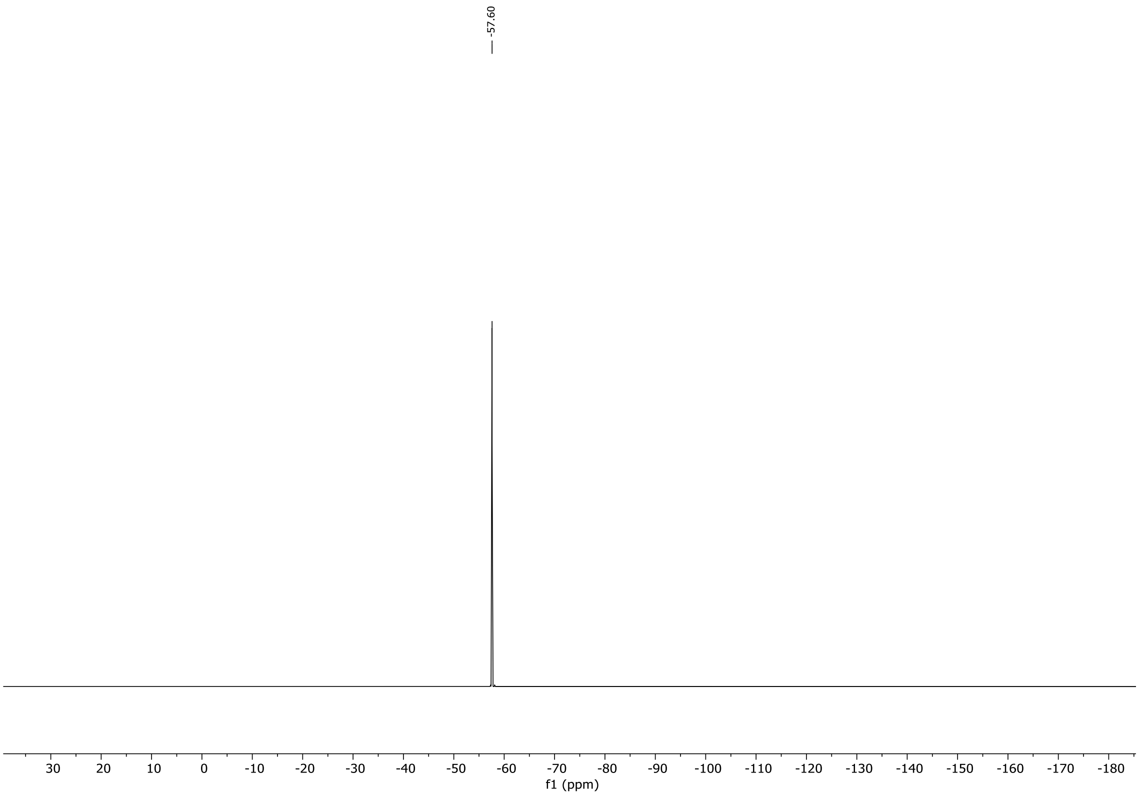

^{1}H NMR (400 MHz, $CDCl_3$) of **3f**

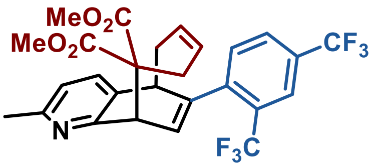

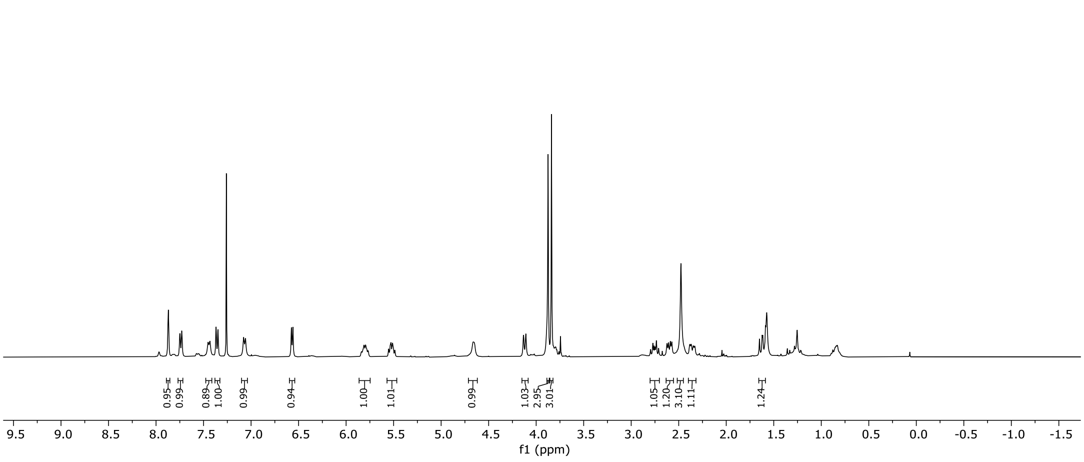

$^{13}C\{^{19}F\}$ NMR (126 MHz, $CDCl_3$) of **3f**

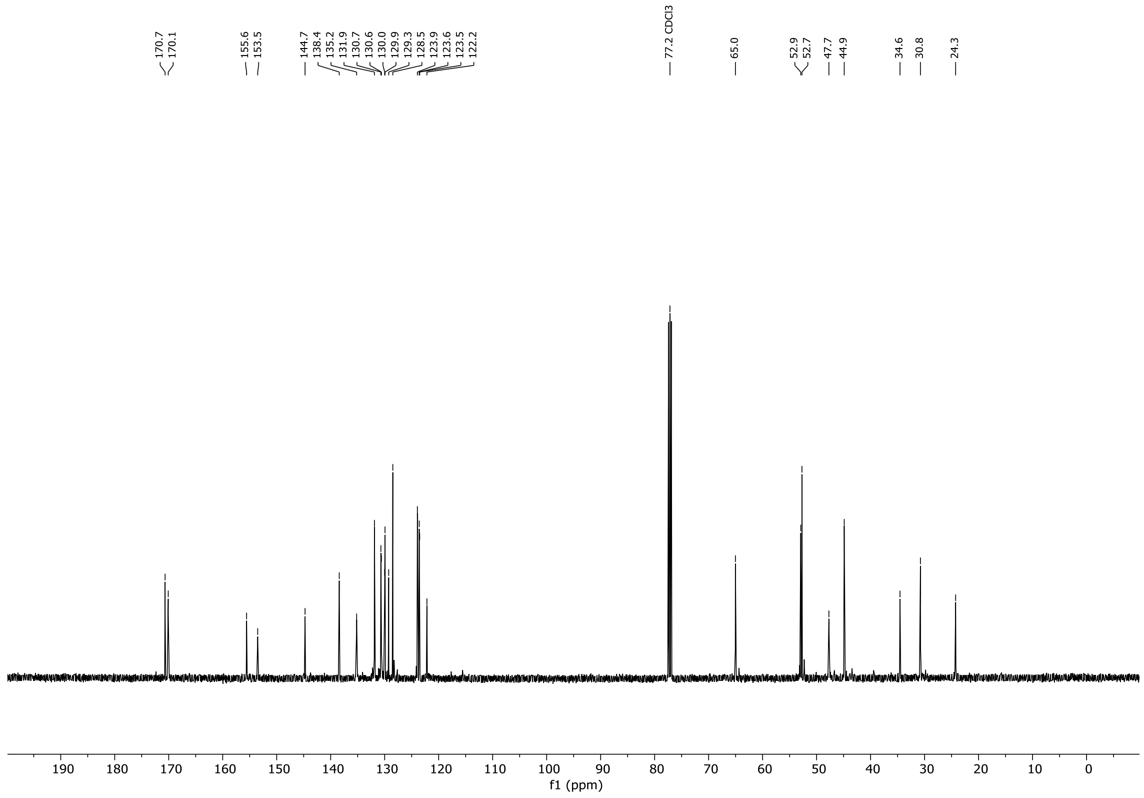

^{19}F NMR (376 MHz, $CDCl_3$) of **3f**

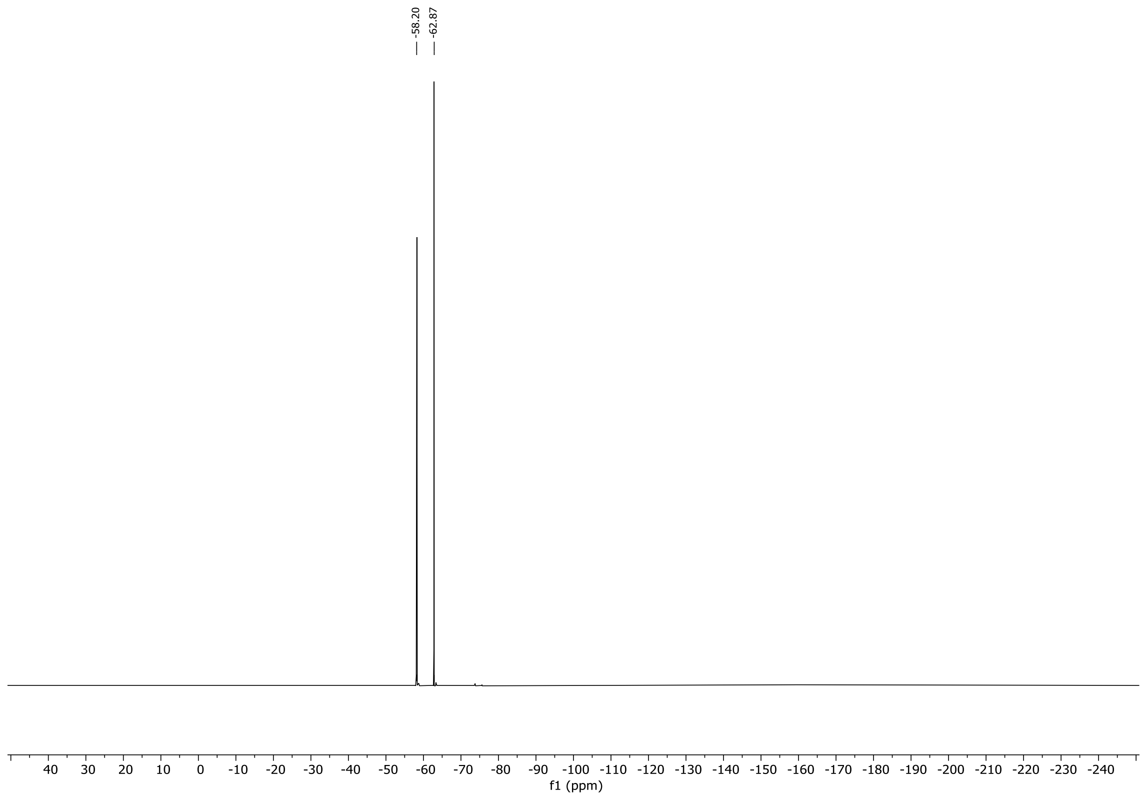

^{1}H NMR (400 MHz, $CDCl_3$) of **4f**

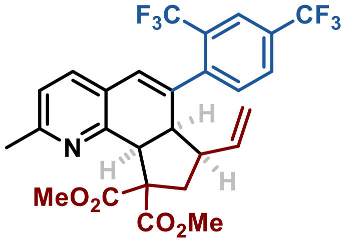

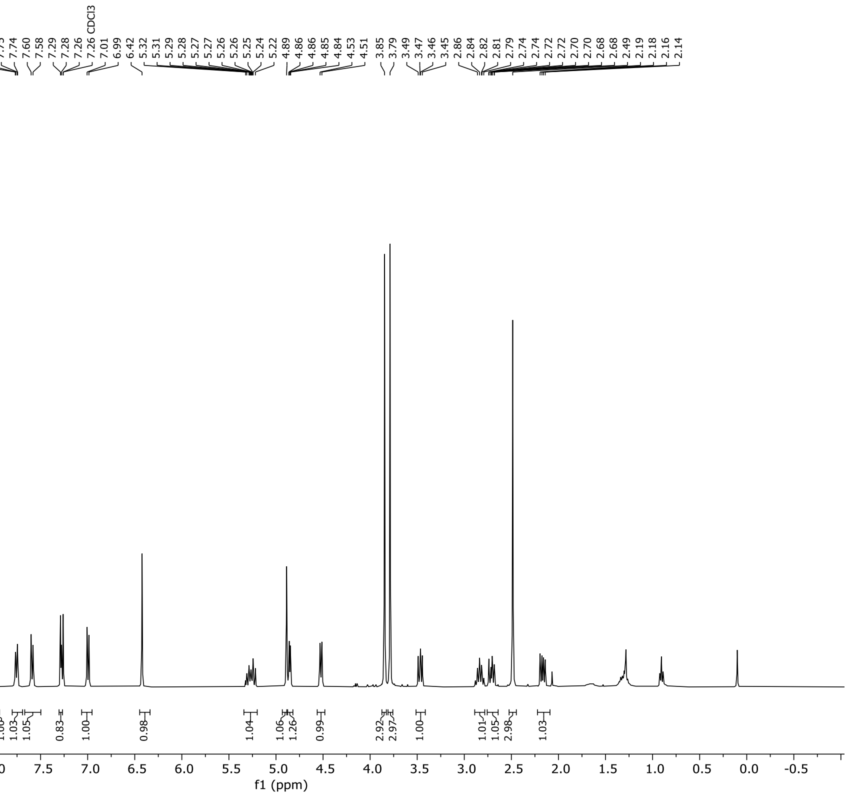

$^{13}C\{^{19}F\}$ NMR (126 MHz, $CDCl_3$) of **4f**

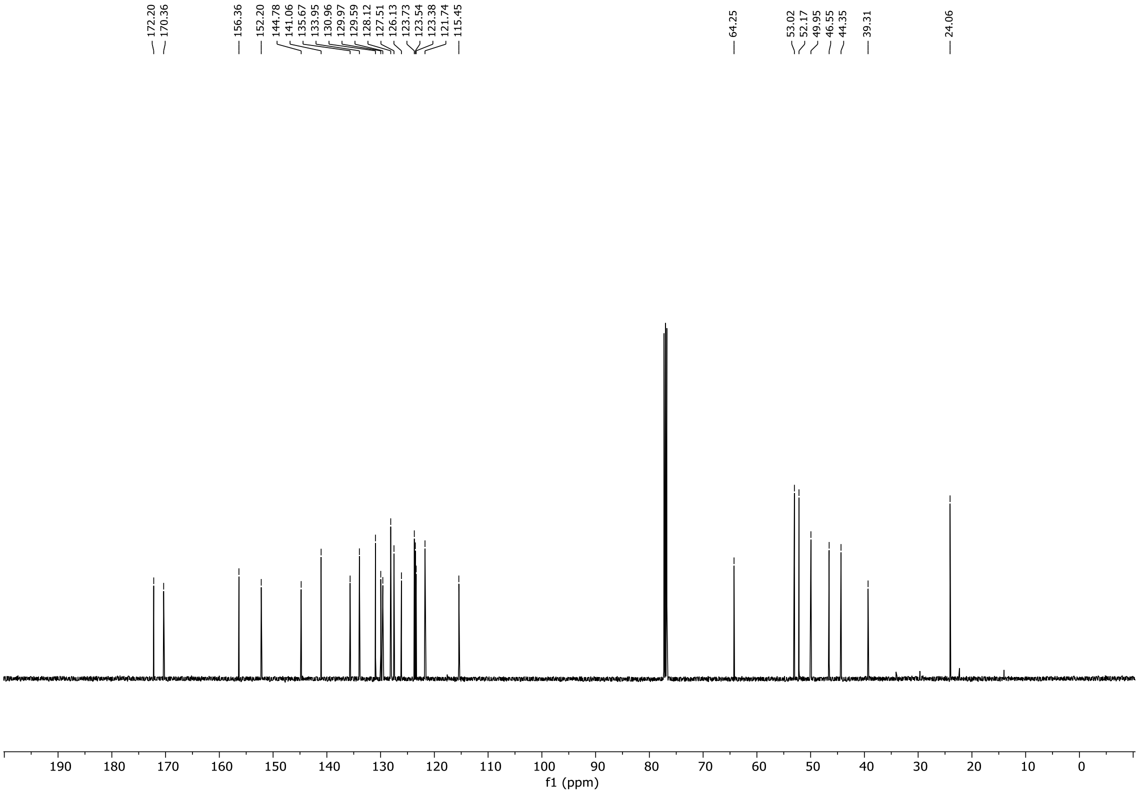

^{19}F NMR (376 MHz, $CDCl_3$) of **4f**

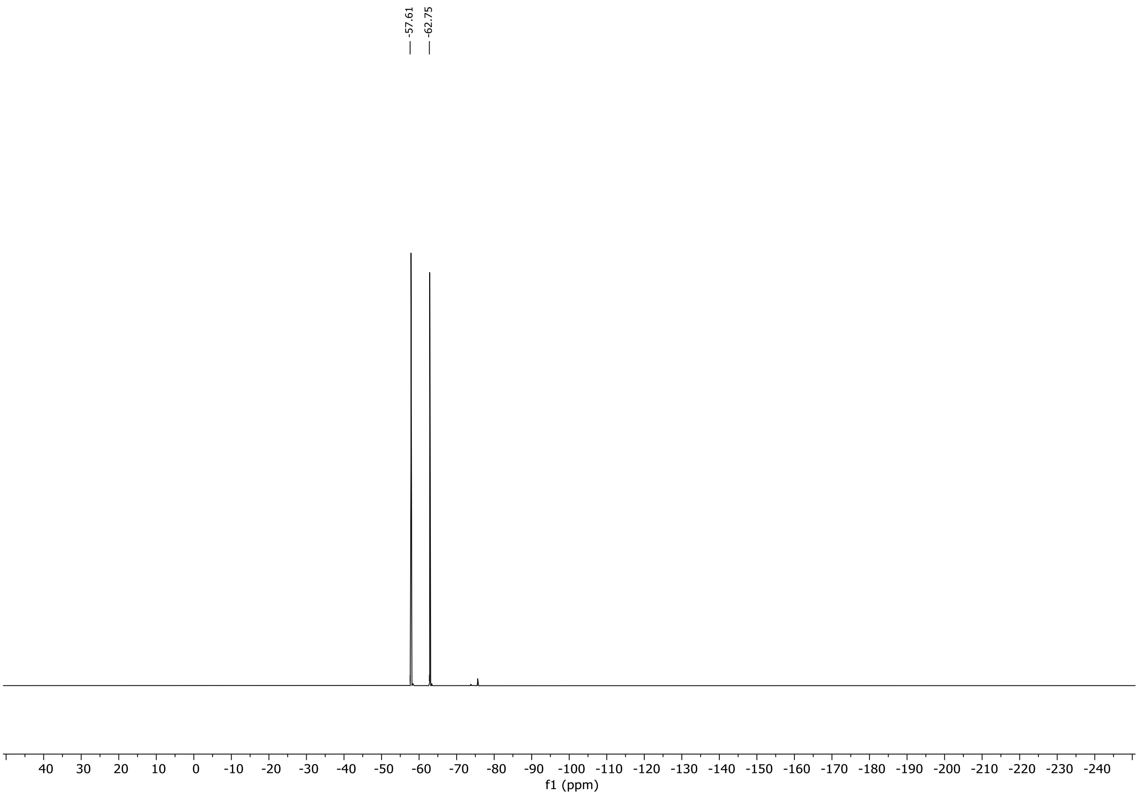

^{1}H NMR (400 MHz, $CDCl_3$) of **5f**

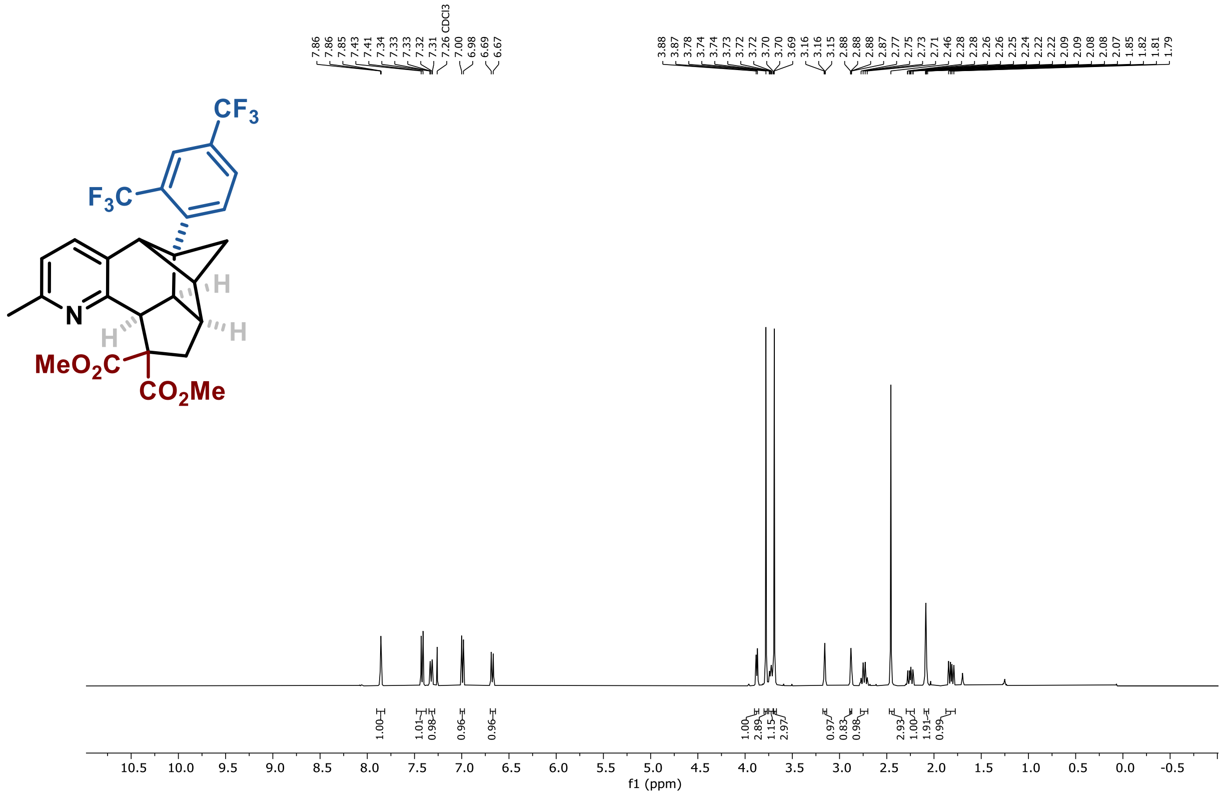

$^{13}C\{^{19}F\}$ NMR (126 MHz, $CDCl_3$) of **5f**

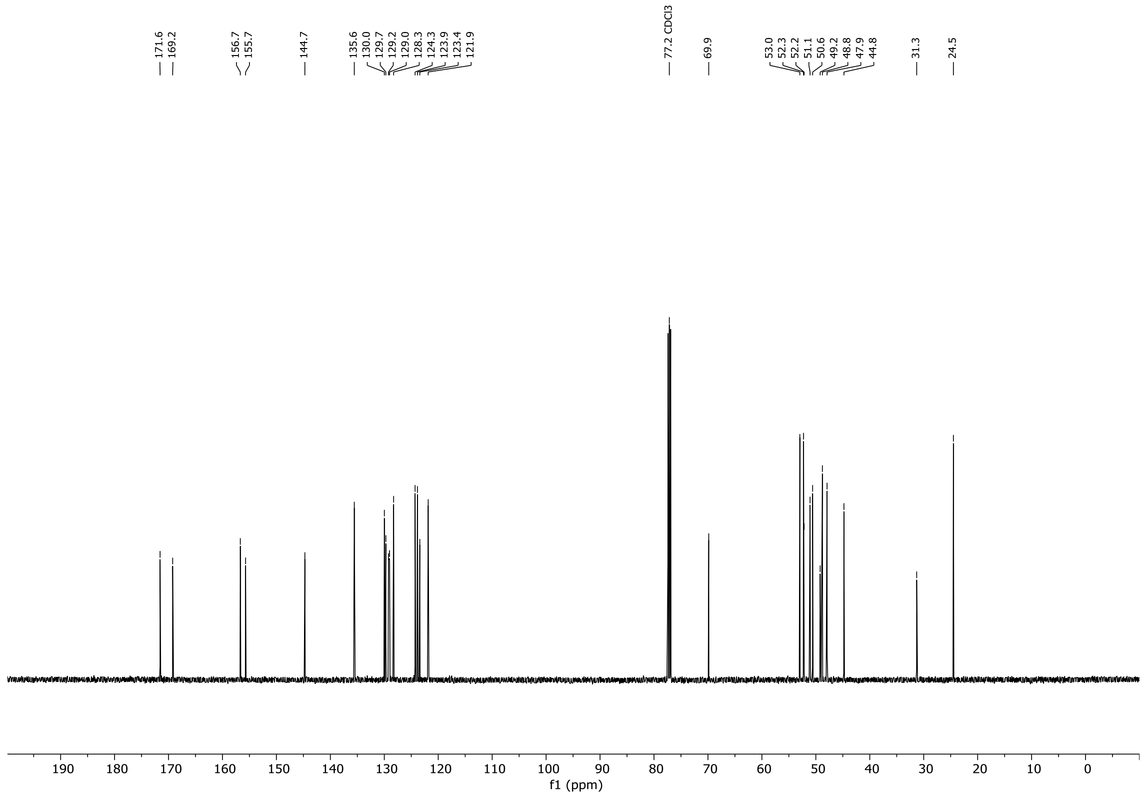

^{19}F NMR (376 MHz, $CDCl_3$) of **5f**

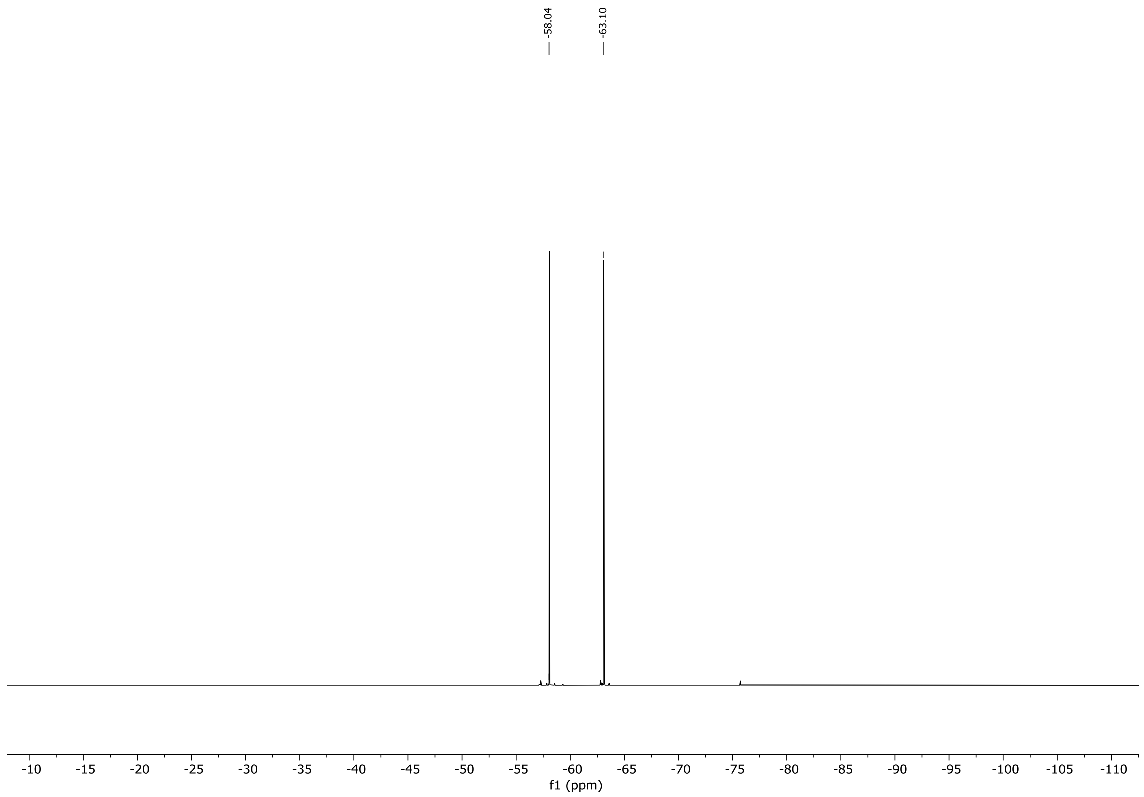

^{1}H NMR (400 MHz, $CDCl_3$) of **3b**

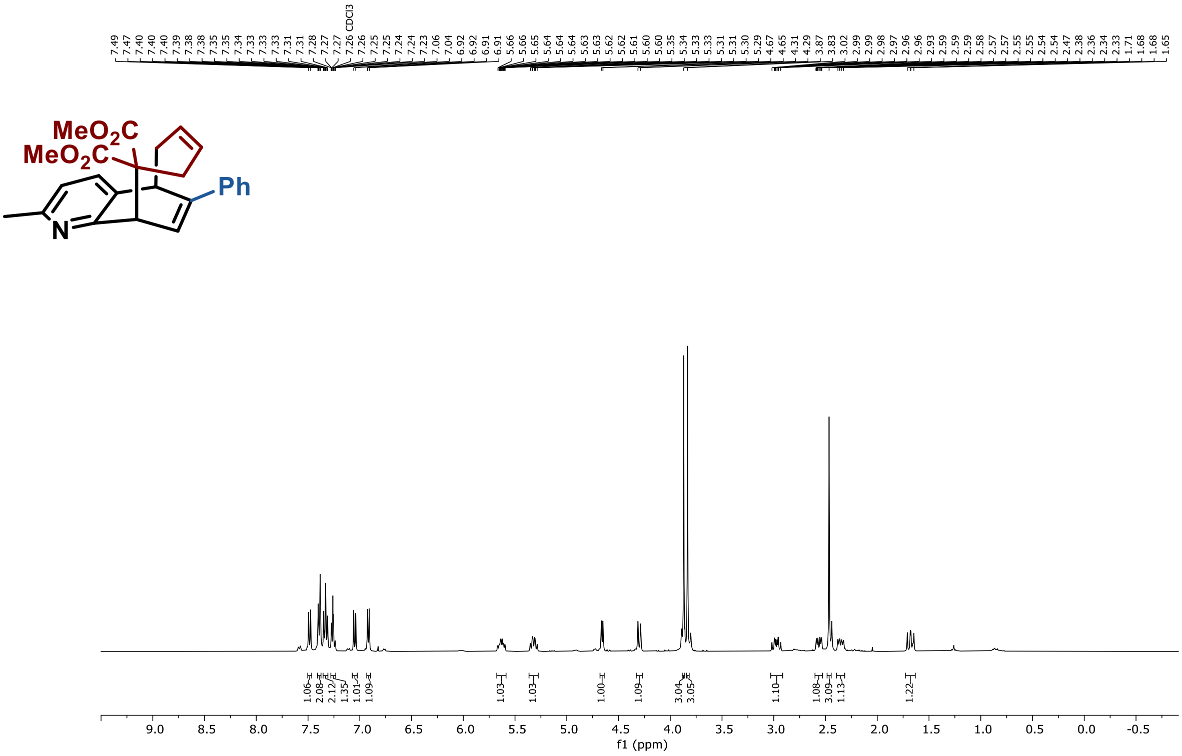

^{13}C NMR (151 MHz, $CDCl_3$) of **3b**

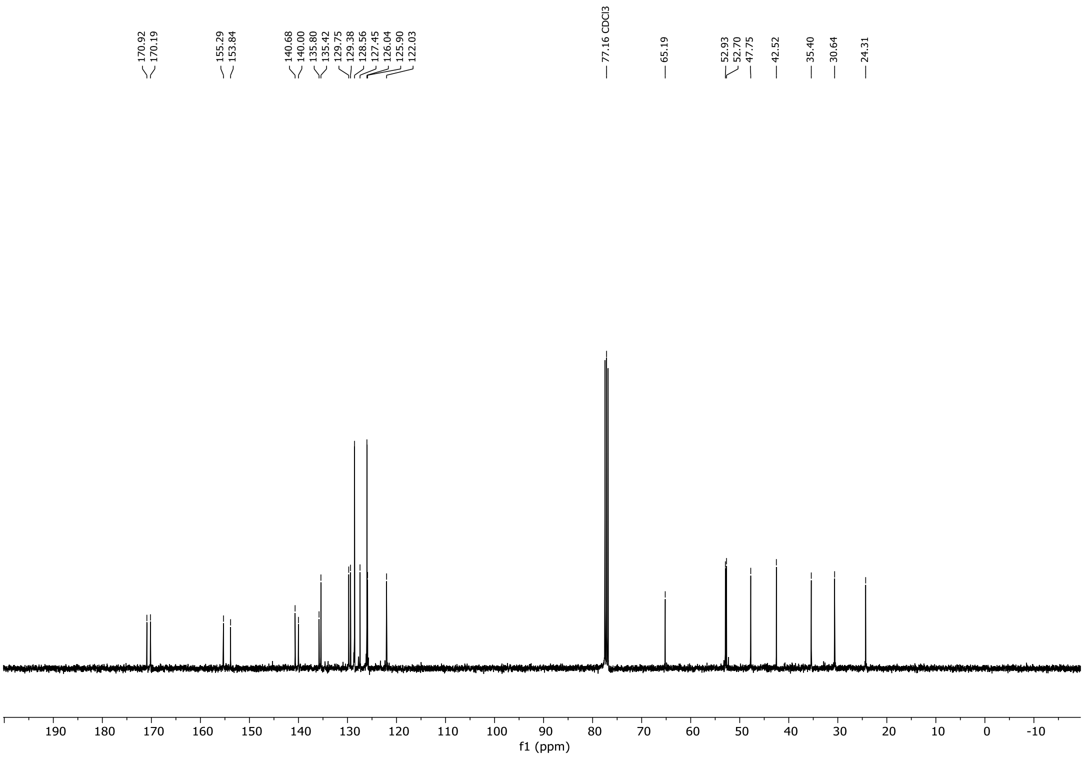

^{1}H NMR (400 MHz, $CDCl_3$) of **4b**

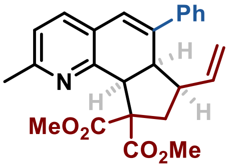

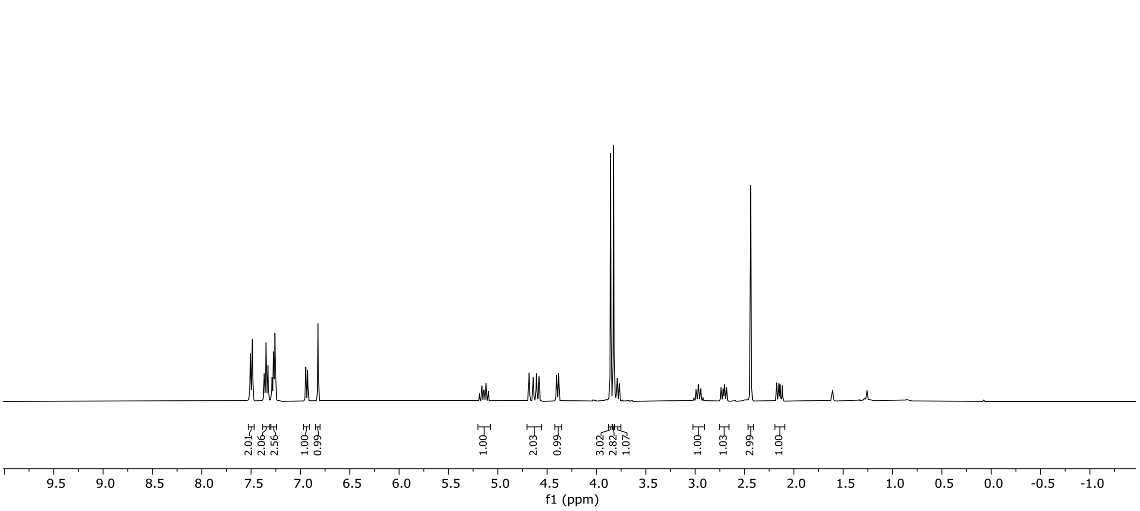

^{13}C NMR (151 MHz, $CDCl_3$) of **4b**

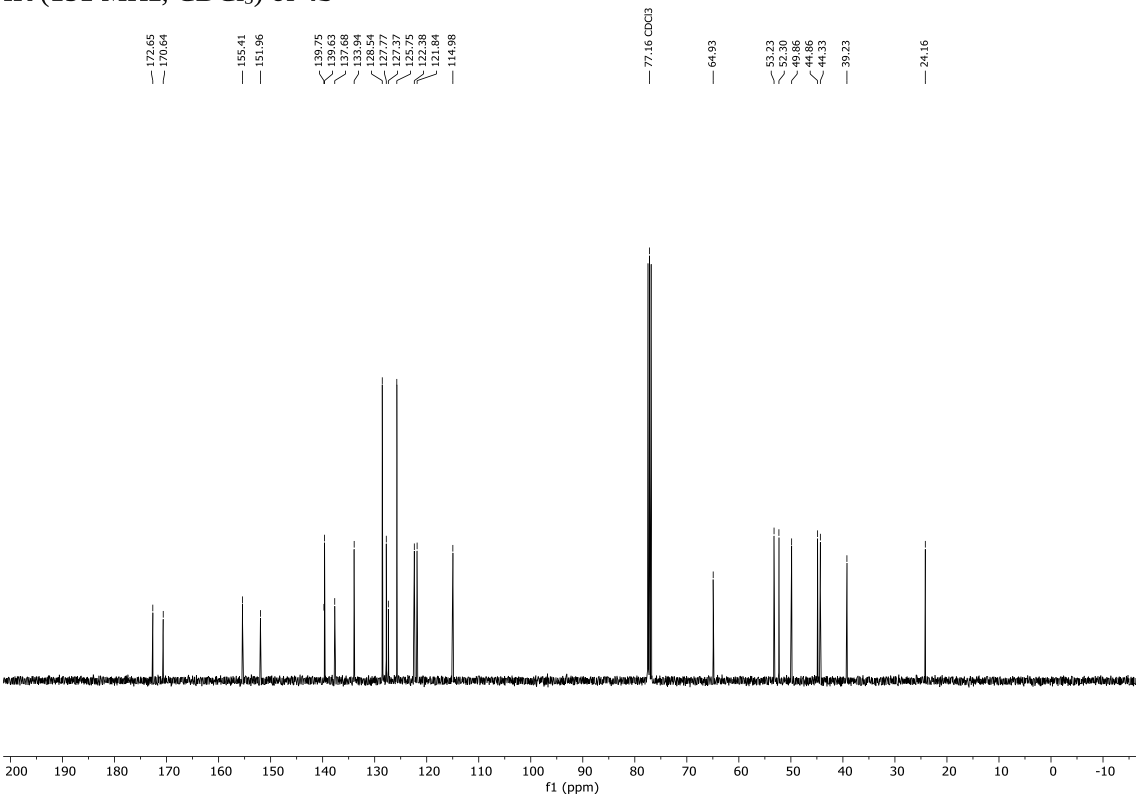

^{1}H NMR (400 MHz, $CDCl_3$) of **5b**

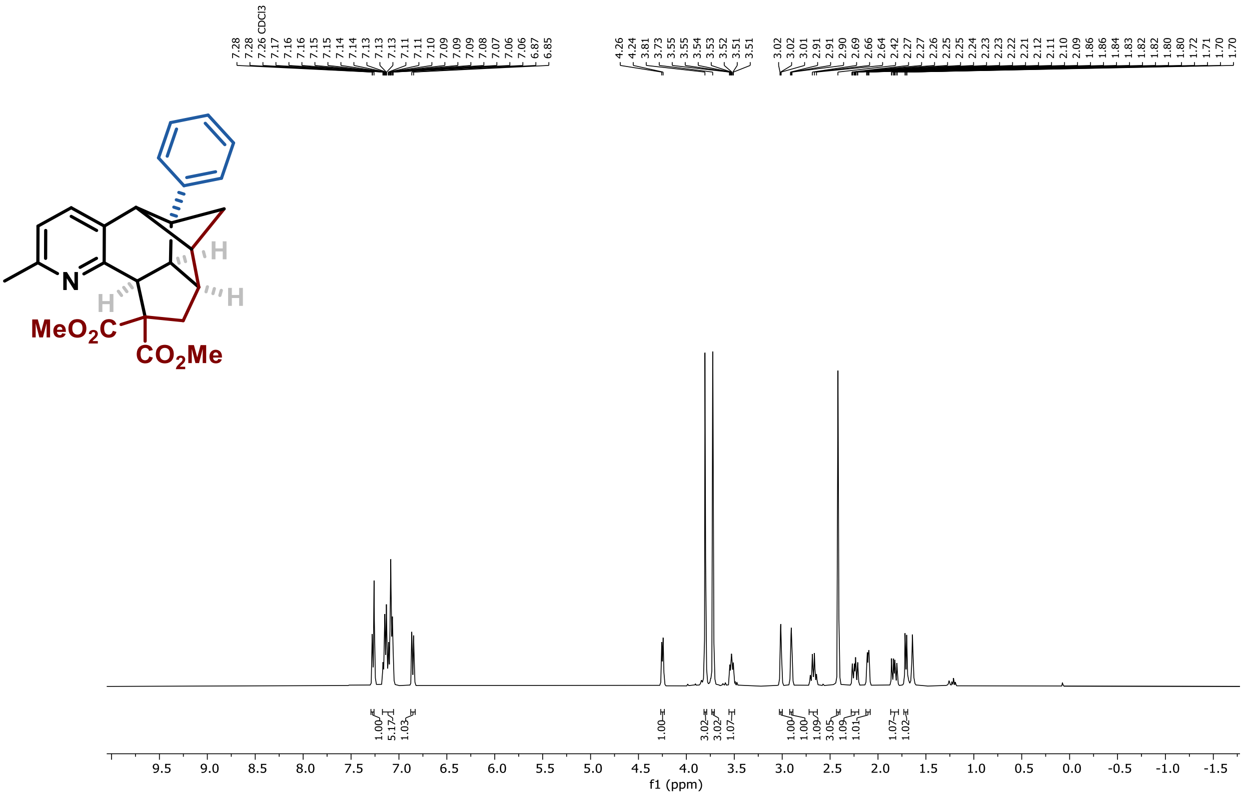

^{13}C NMR (101 MHz, $CDCl_3$) of **5b**

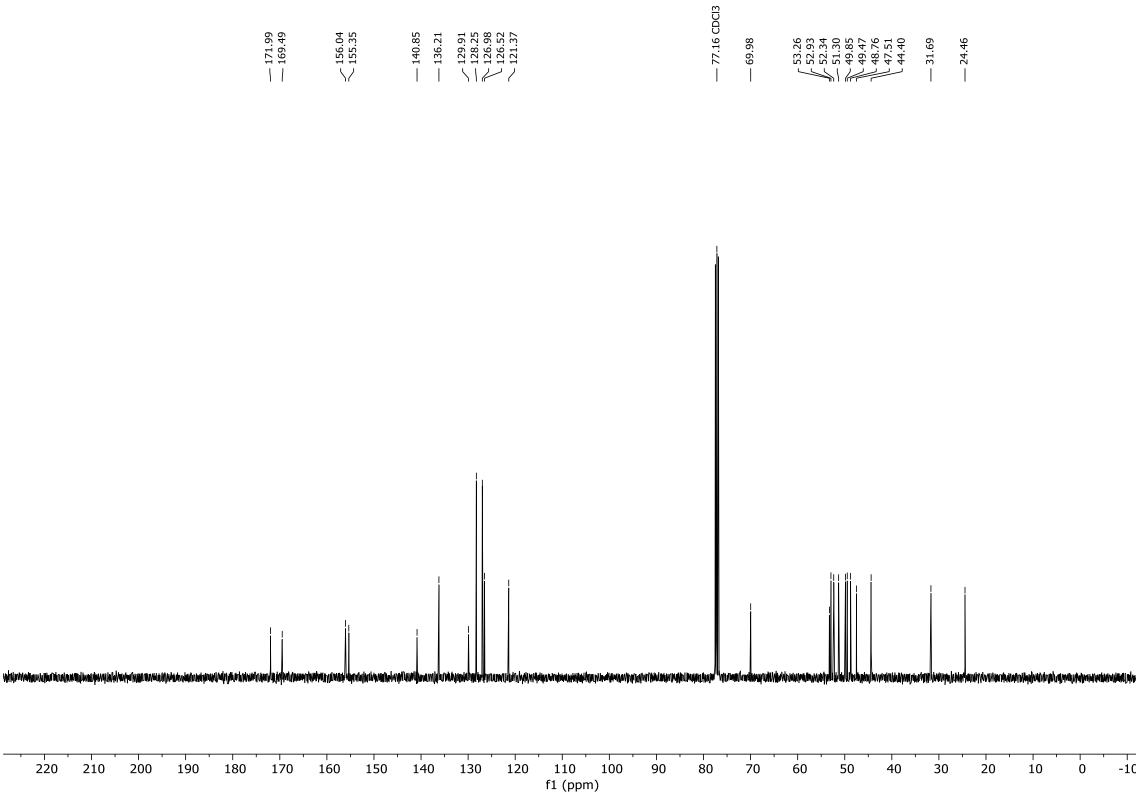

^{1}H, ^{1}H COSY (599 Hz, $CDCl_3$) of **5b**

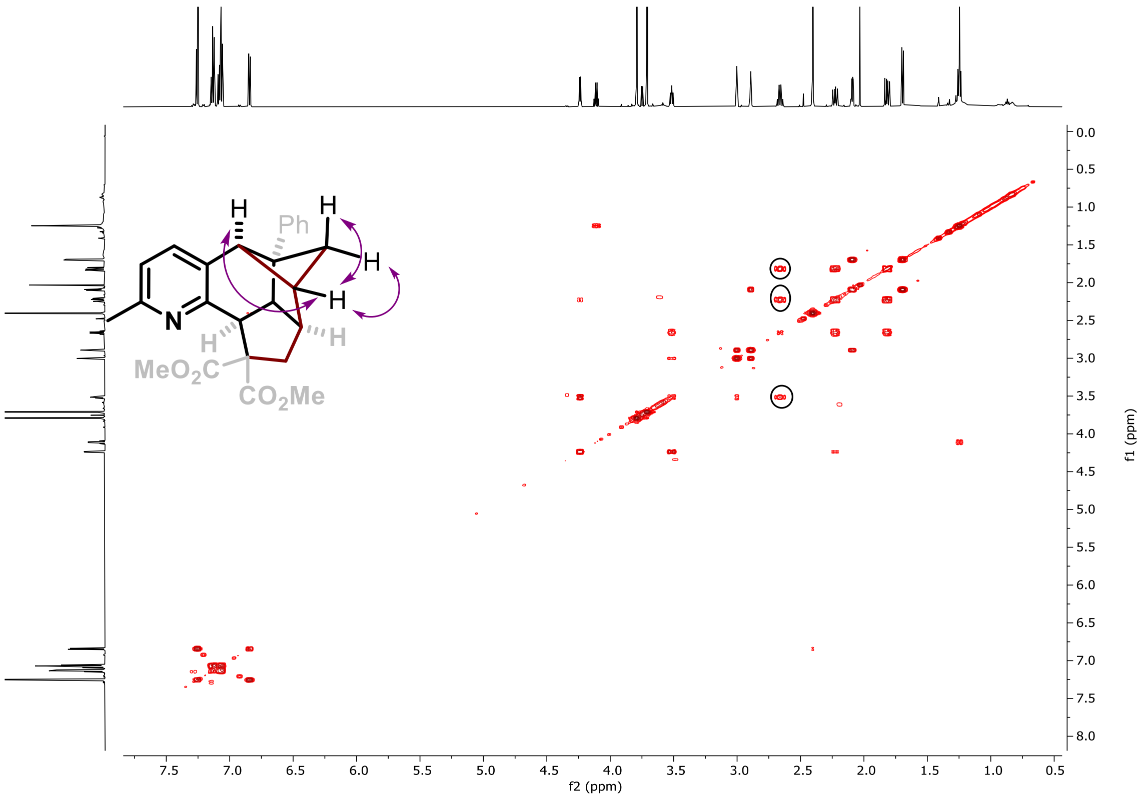

^{1}H, ^{13}C HSQC (599 Hz, $CDCl_3$) of **5b**

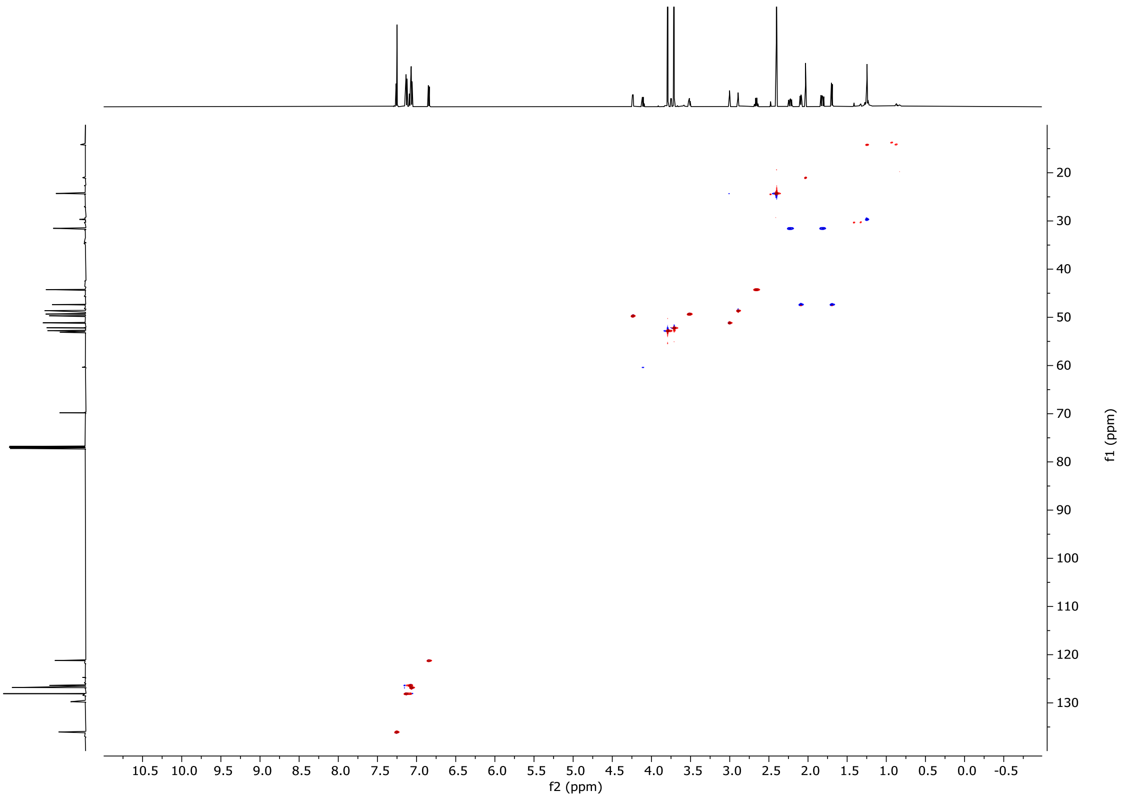

^{1}H, ^{13}C HMBC (599 Hz, $CDCl_3$) of **5b**

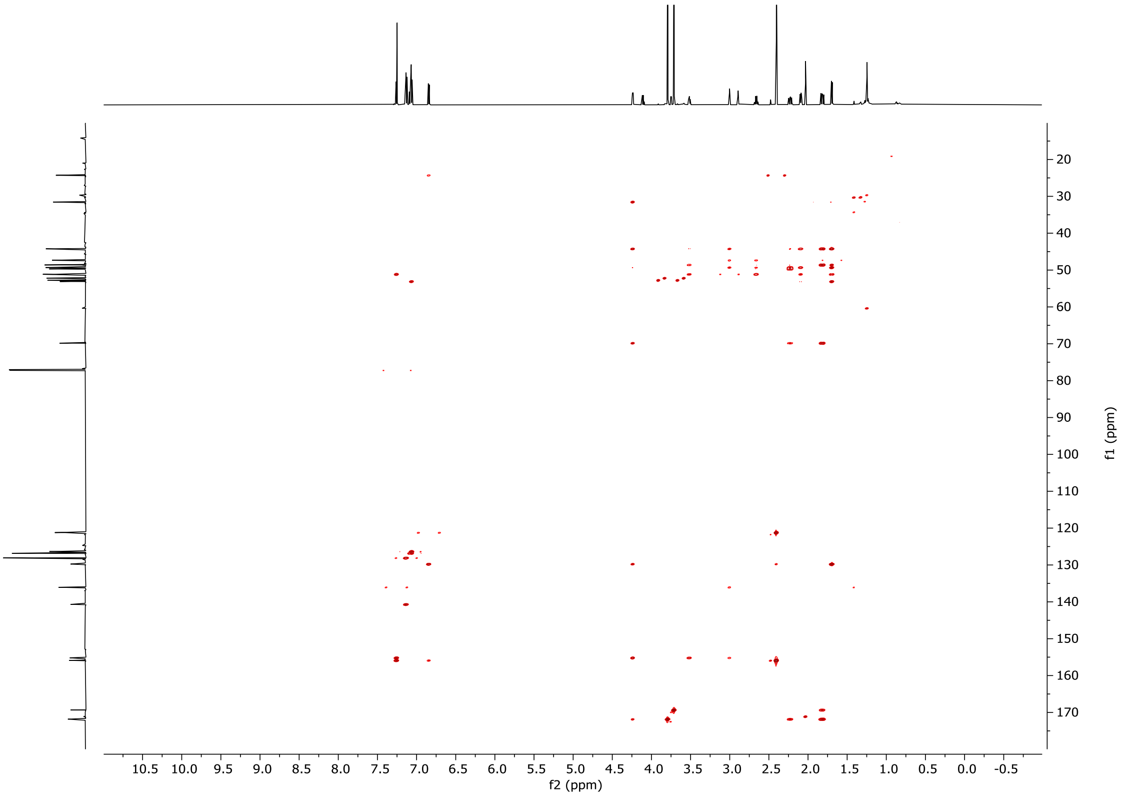

^{1}H NMR (400 MHz, $CDCl_3$) of **5g**

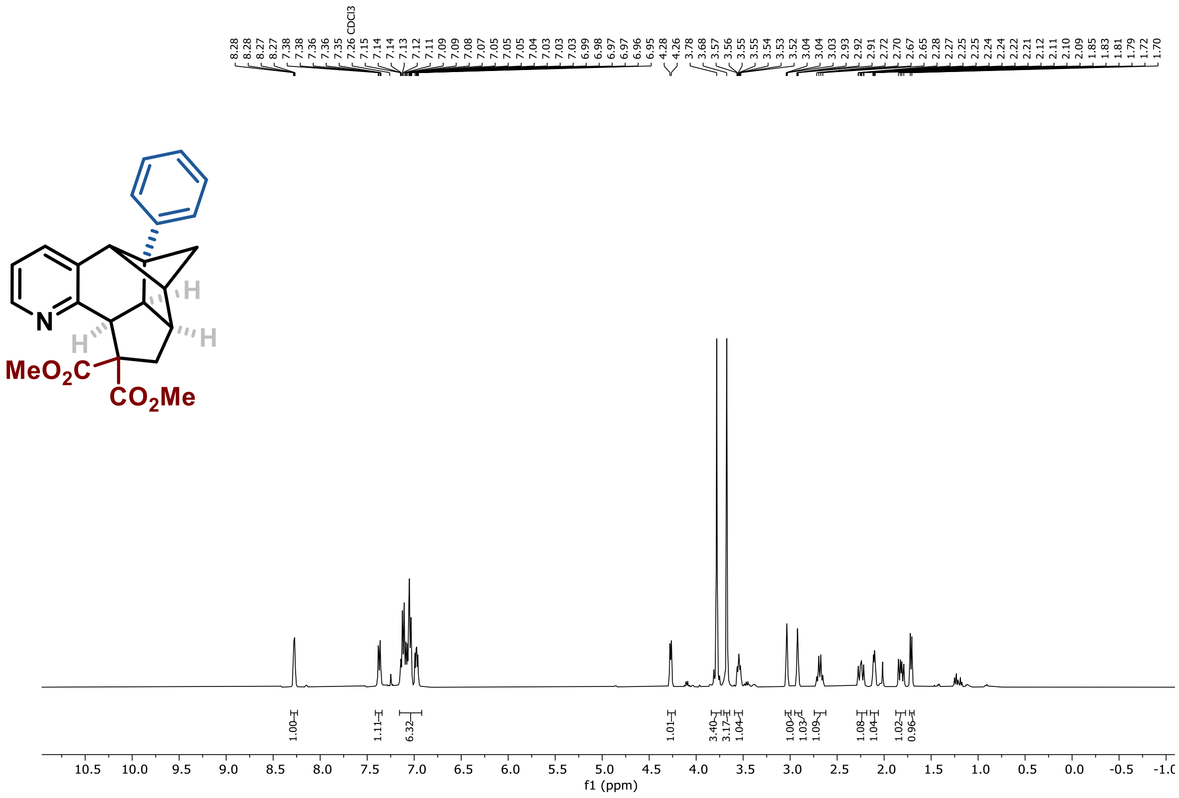

^{13}C NMR (101 MHz, $CDCl_3$) of **5g**

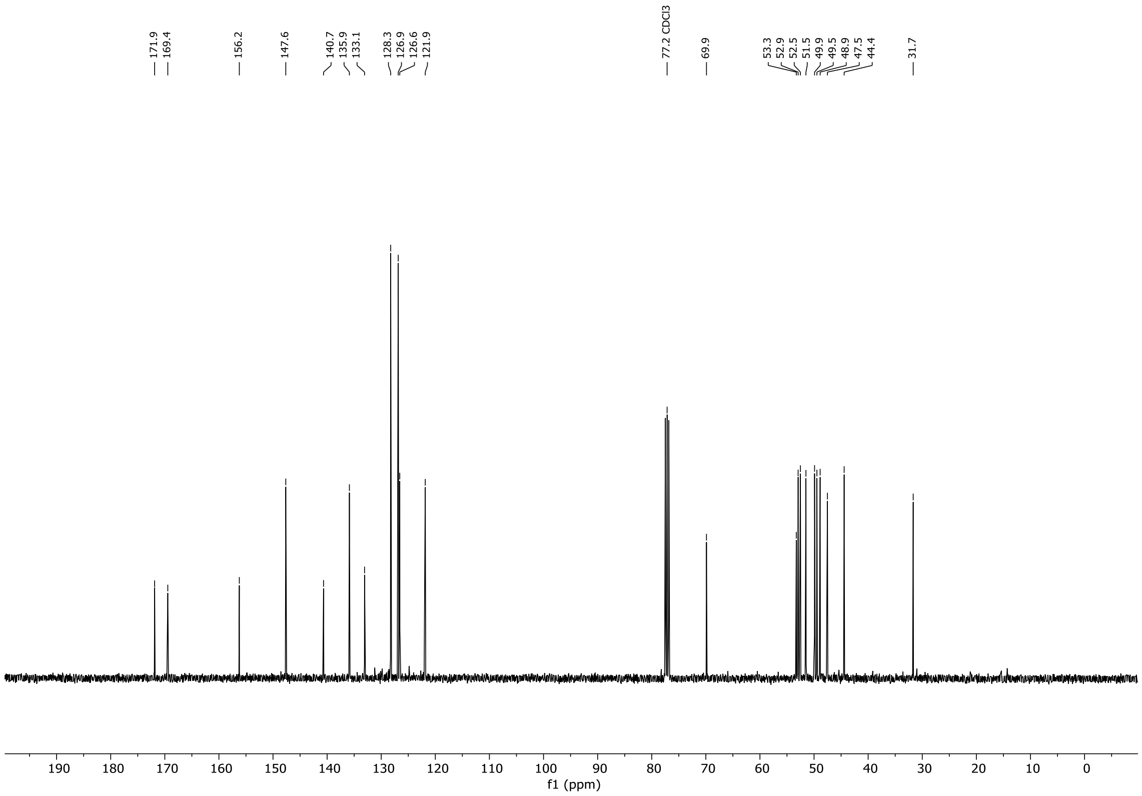

^{1}H NMR (400 MHz, $CDCl_3$) of **5h**

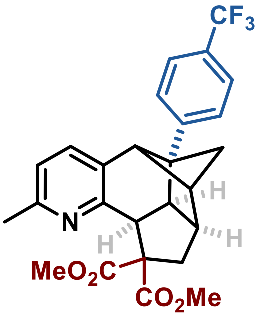

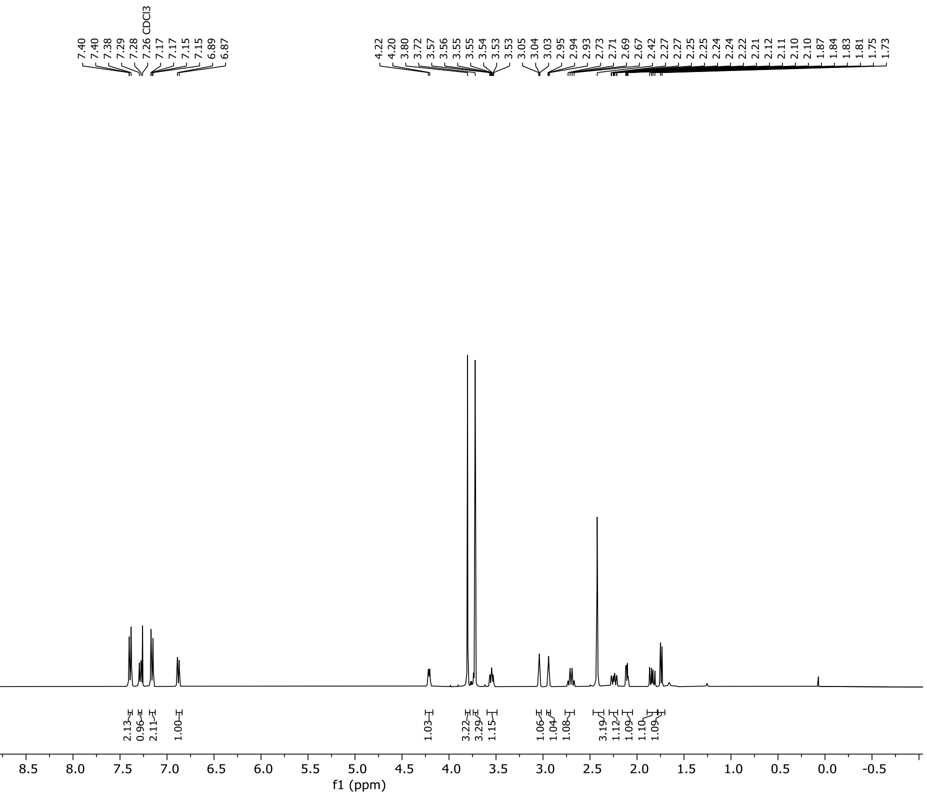

$^{13}C\{^{19}F\}$ NMR (151 MHz, $CDCl_3$) of **5h**

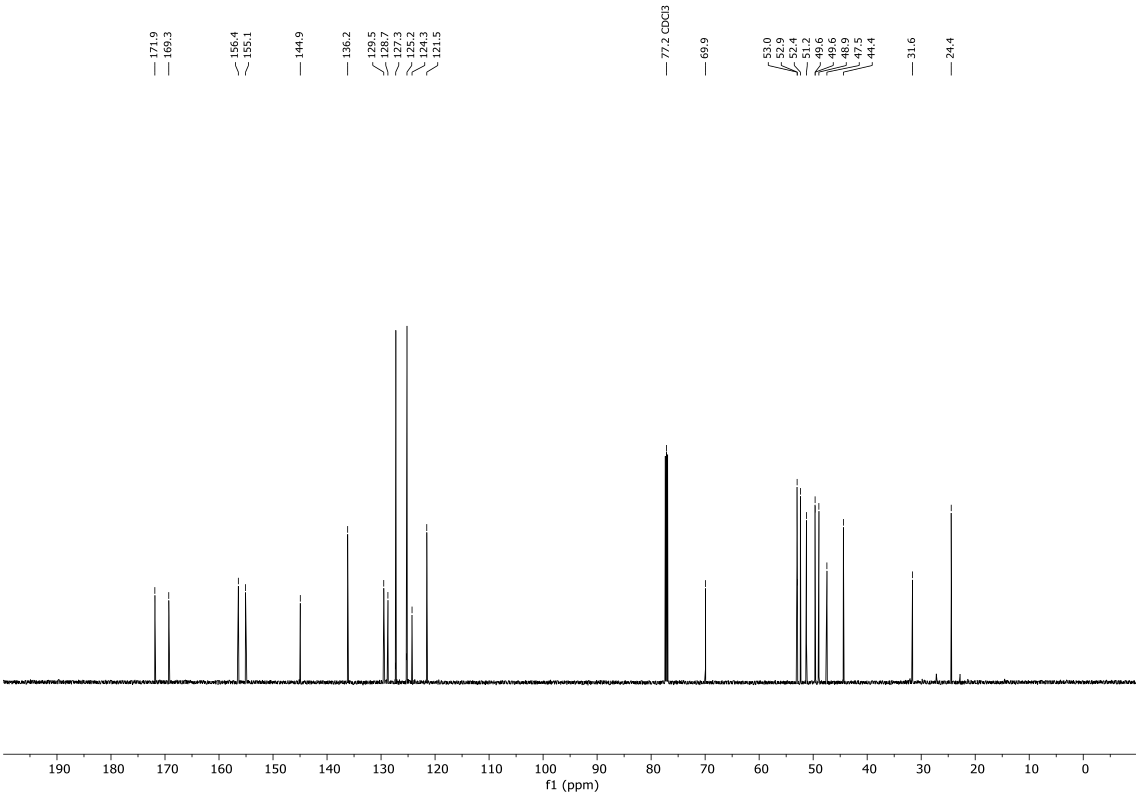

^{19}F NMR (376 MHz, $CDCl_3$) of **5h**

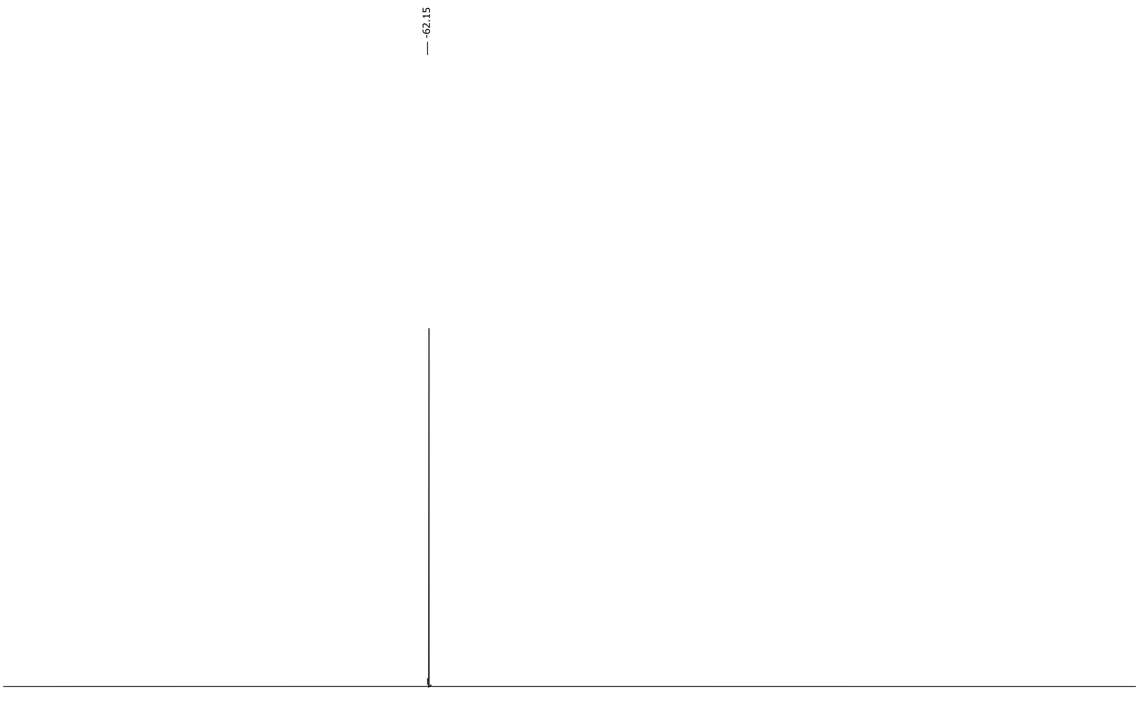

^{1}H NMR (400 MHz, $CDCl_3$) of **5i**

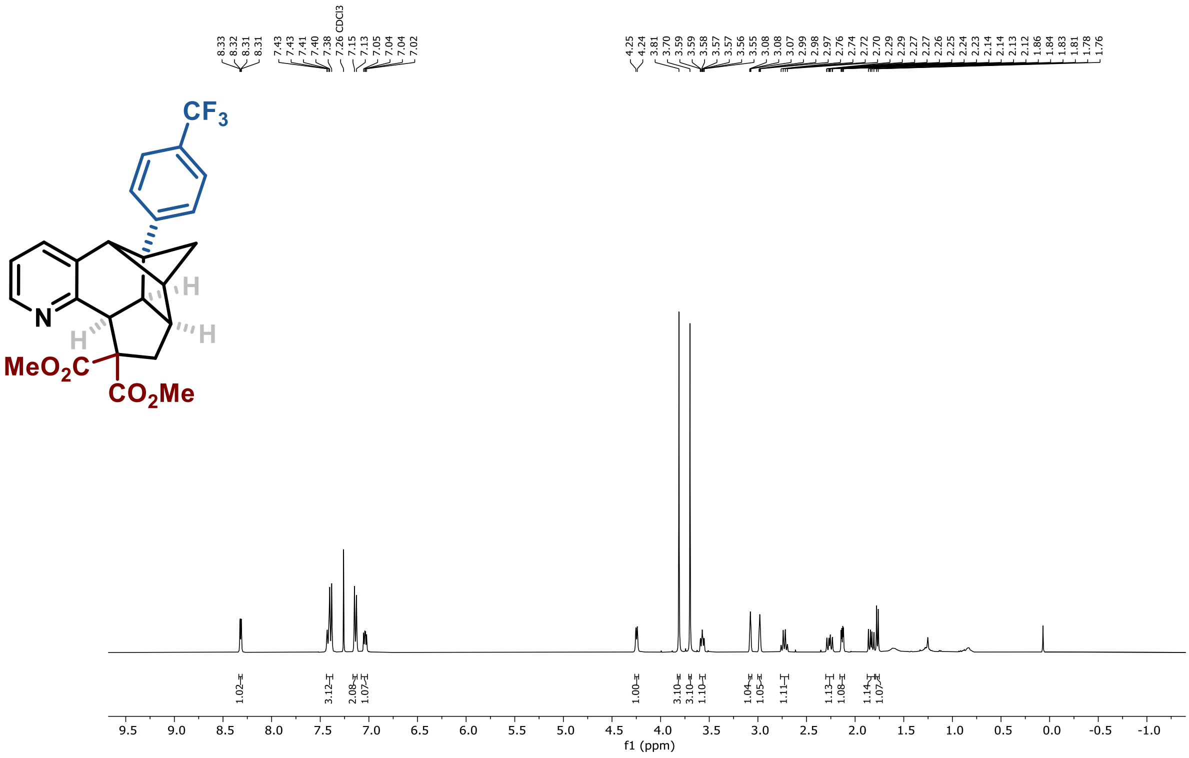

^{13}C{^{19}F} NMR (126 MHz, $CDCl_3$) of **5i**

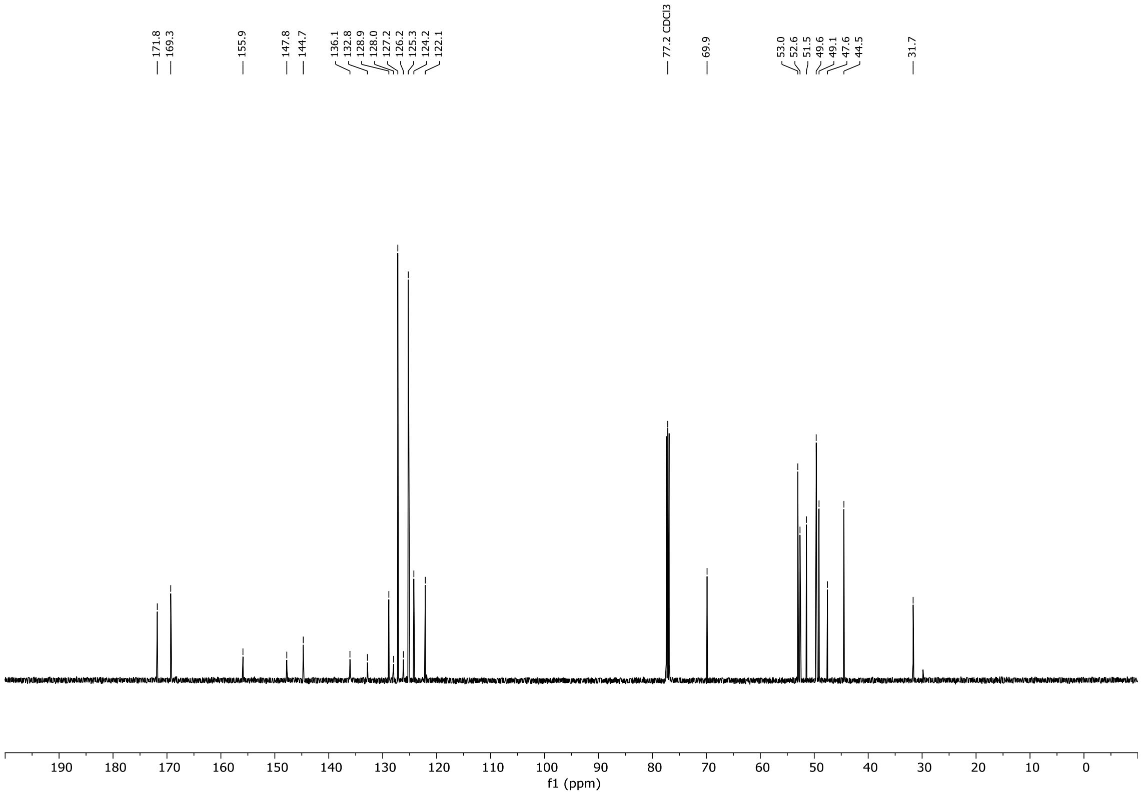

^{19}F NMR (376 MHz, $CDCl_3$) of **5i**

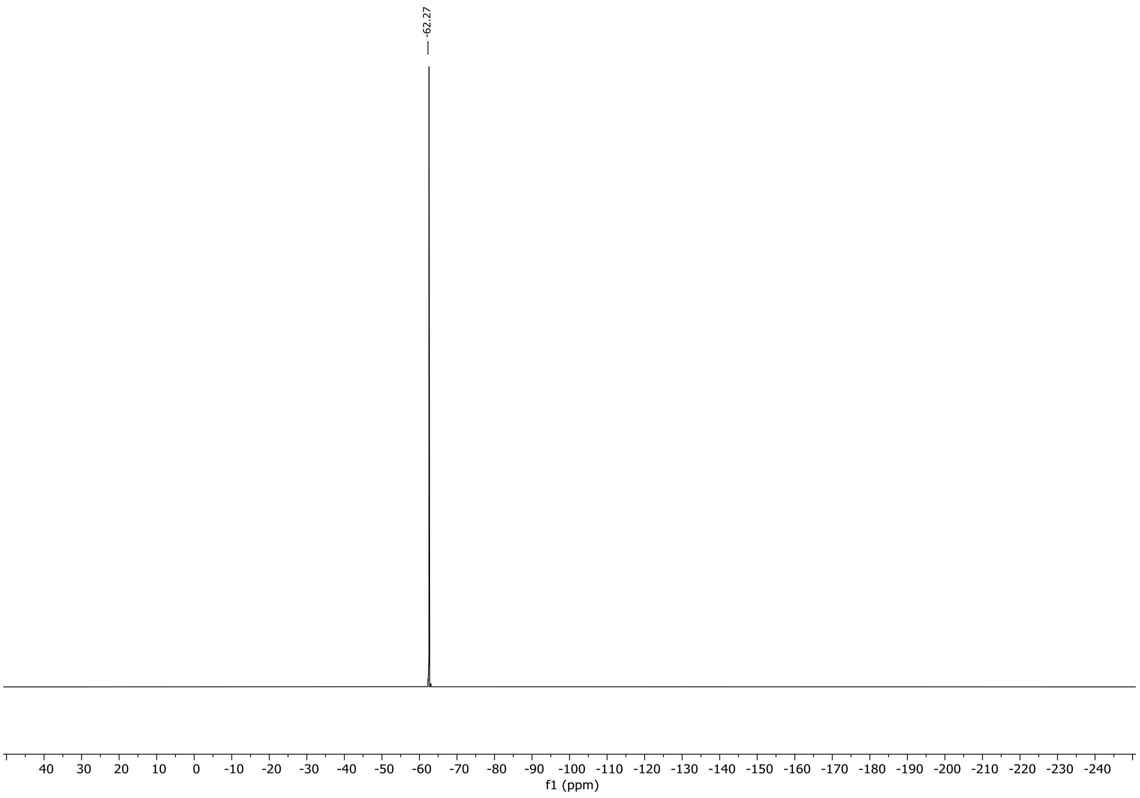

^{1}H NMR (400 MHz, $CDCl_3$) of **5j**

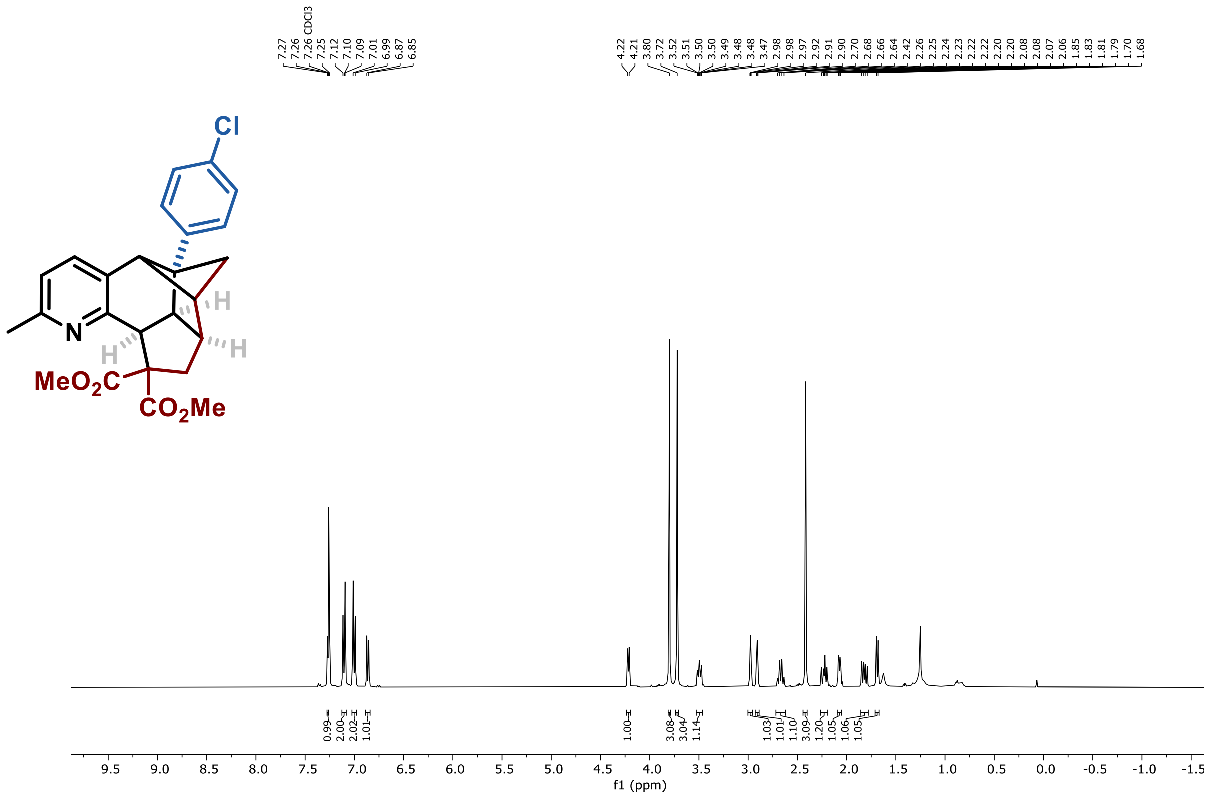

^{13}C NMR (101 MHz, $CDCl_3$) of **5j**

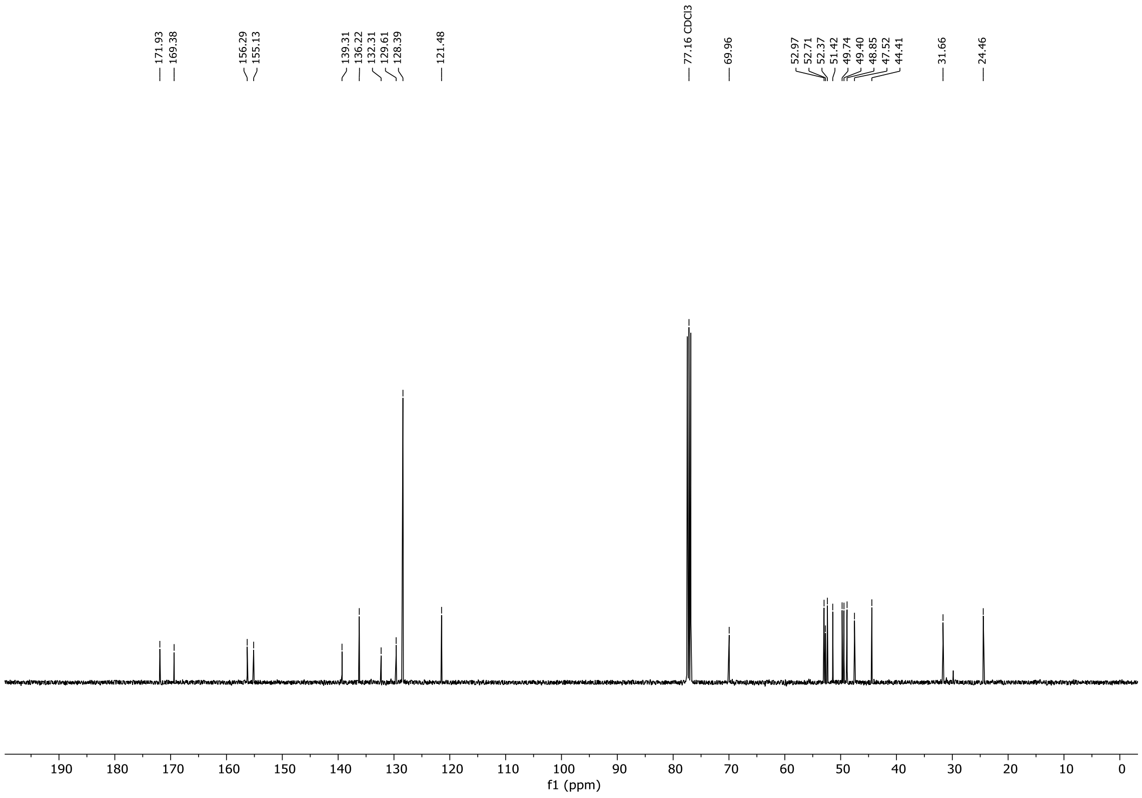

^{1}H NMR (400 MHz, $CDCl_3$) of **5k**

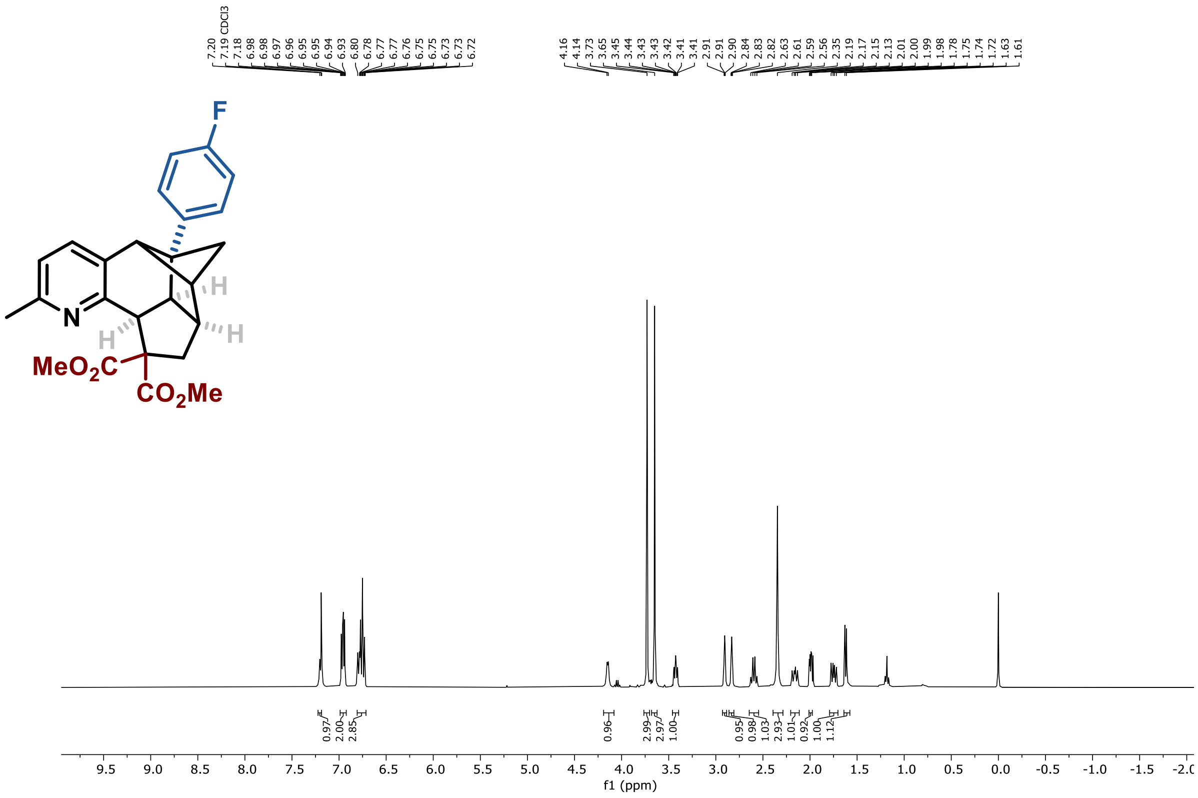

$^{13}C\{^{19}F\}$ NMR (126 MHz, $CDCl_3$) of **5k**

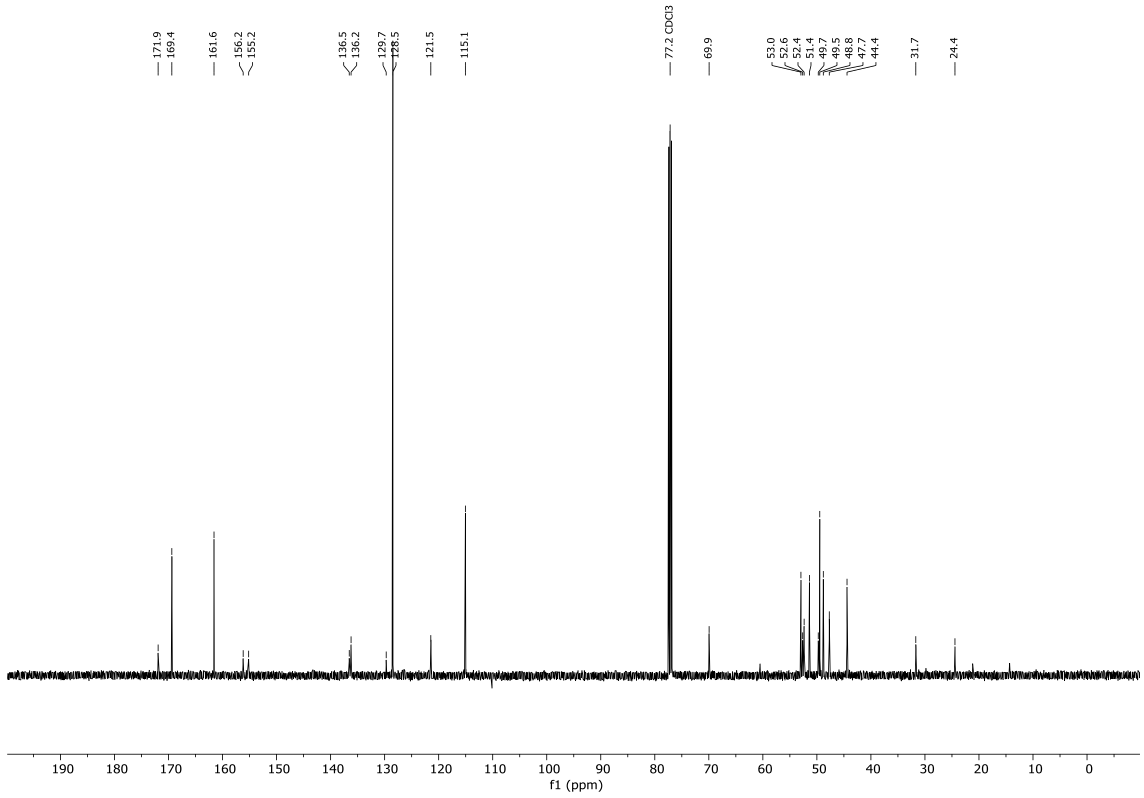

^{19}F NMR (376 MHz, $CDCl_3$) of **5k**

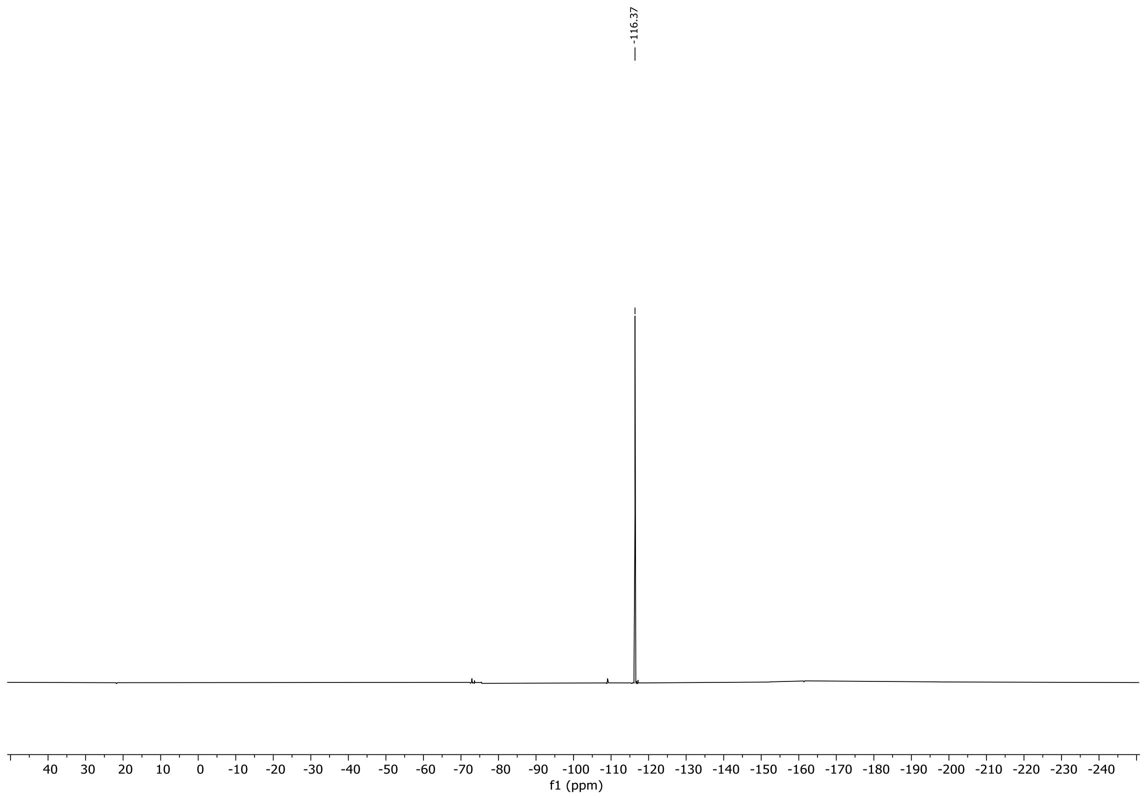

^{1}H NMR (400 MHz, $CDCl_3$) of **5l**

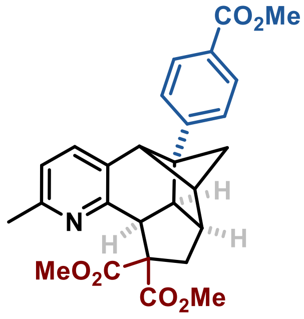

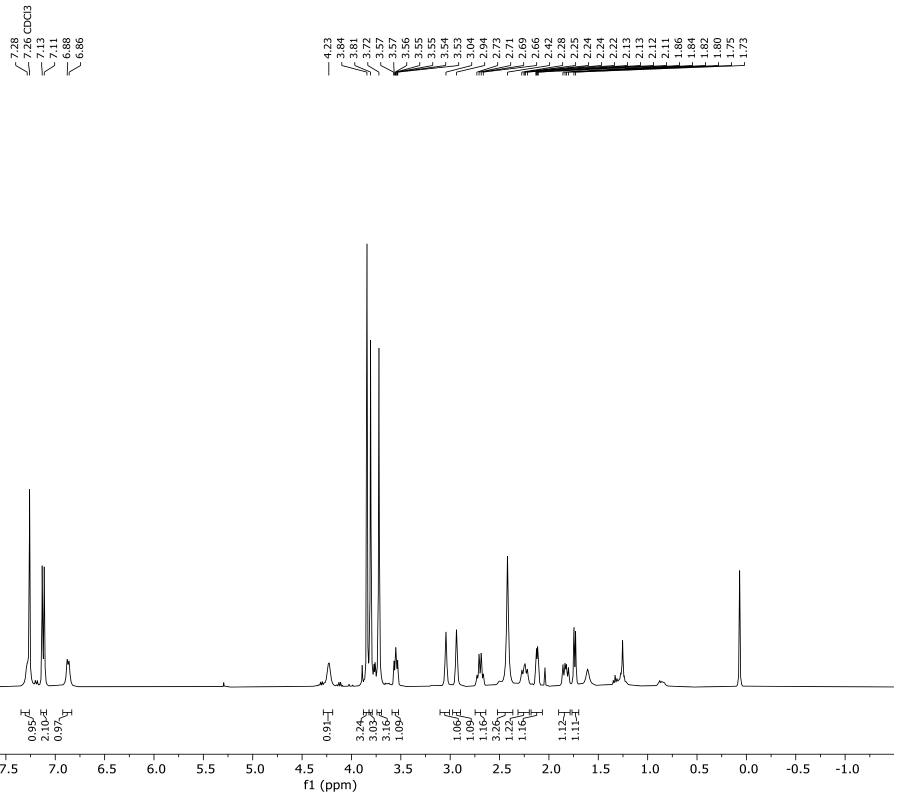

^{13}C NMR (101 MHz, $CDCl_3$) of **5l**

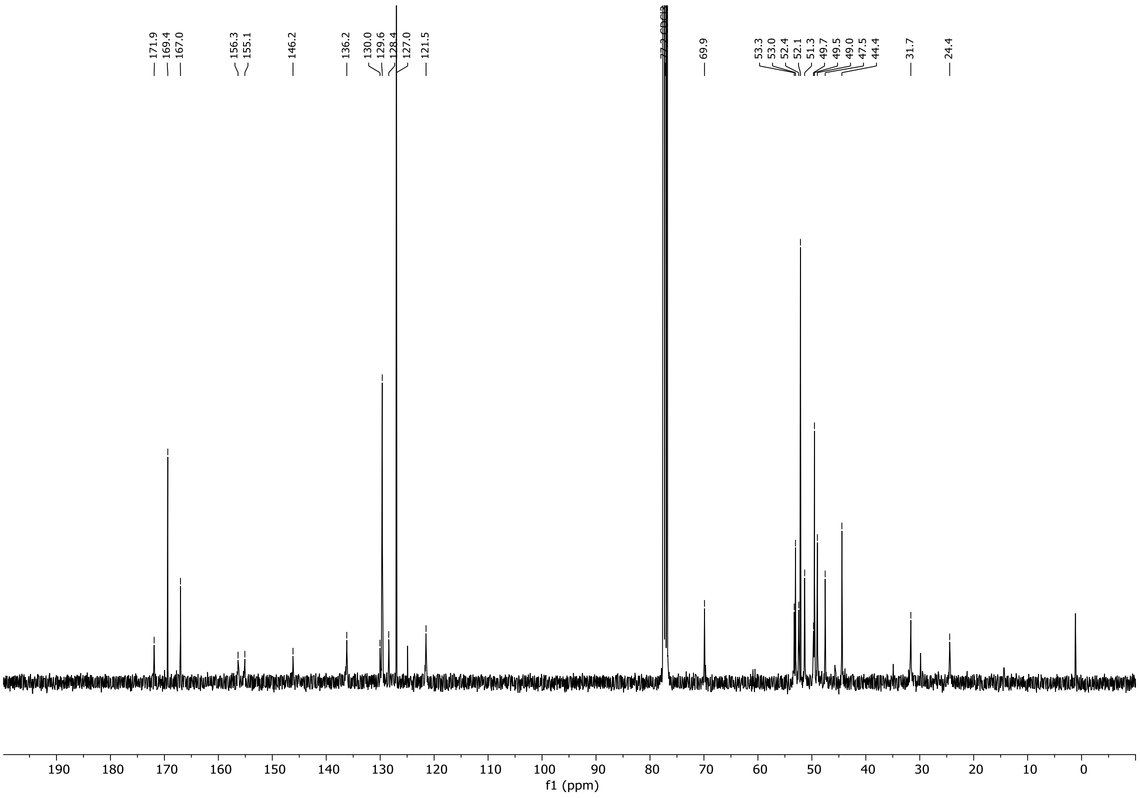

^{1}H NMR (400 MHz, $CDCl_3$) of **5m**

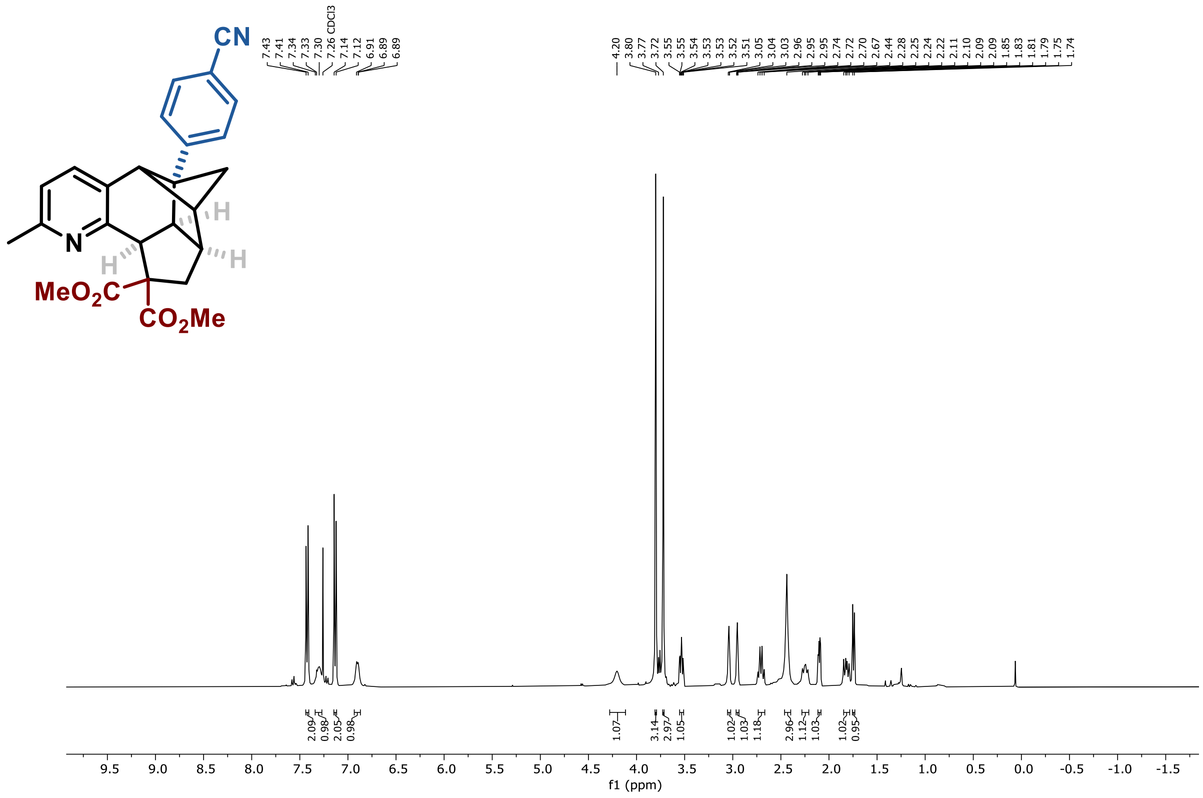

^{13}C NMR (101 MHz, $CDCl_3$) of **5m**

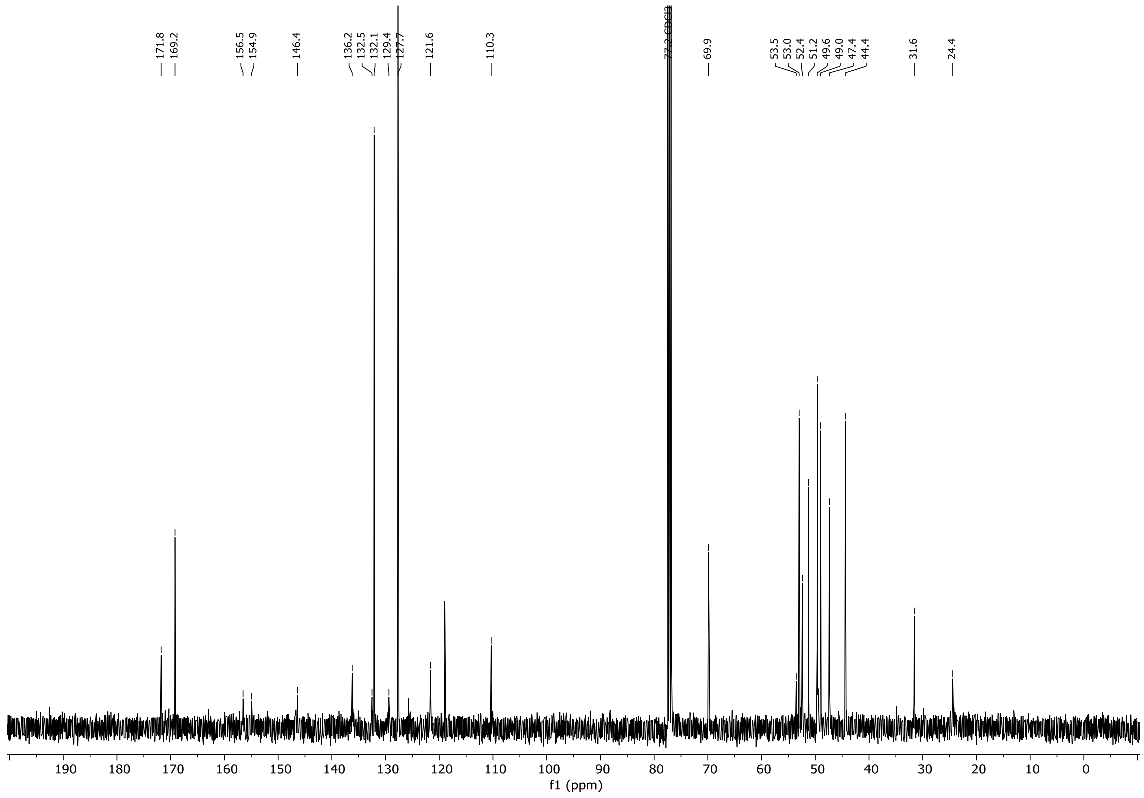

^{1}H NMR (400 MHz, $CDCl_3$) of **5n**

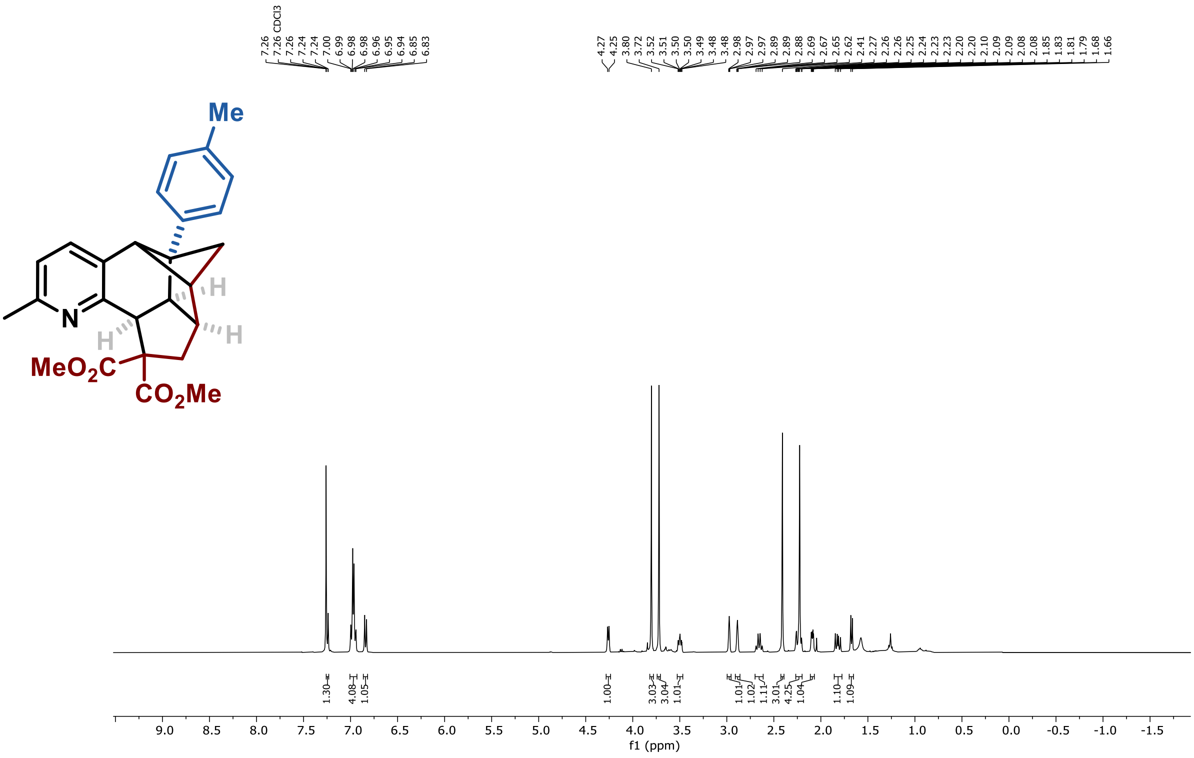

^{13}C NMR (101 MHz, $CDCl_3$) of **5n**

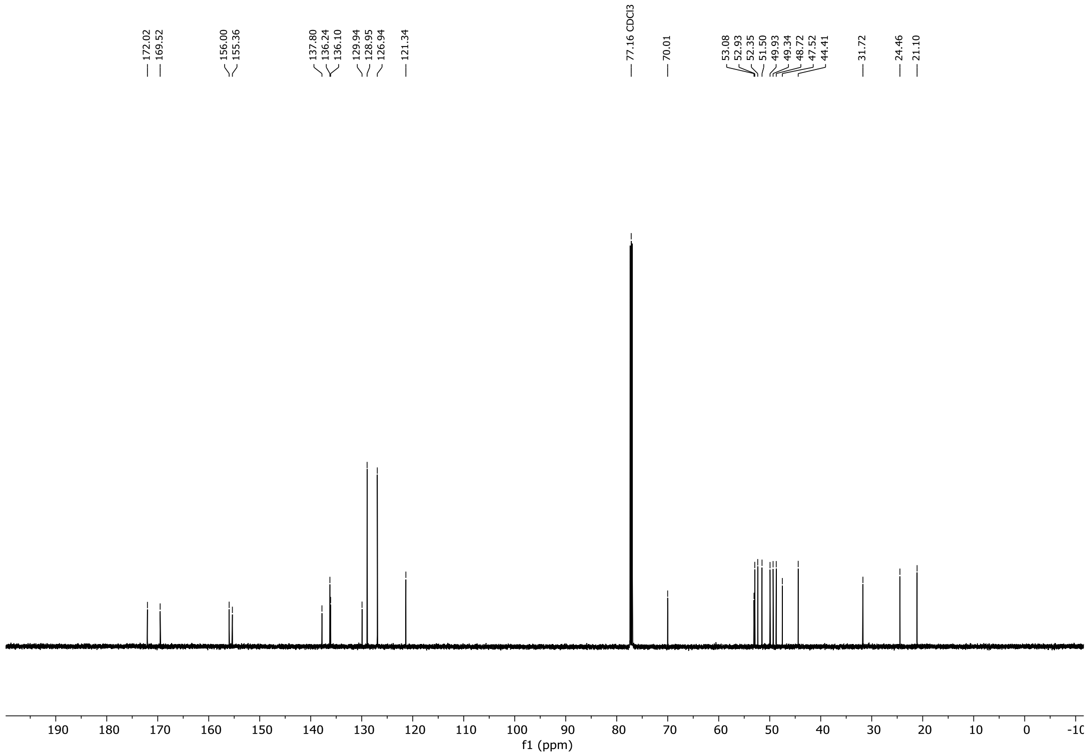

^{1}H NMR (400 MHz, $CDCl_3$) of **5o**

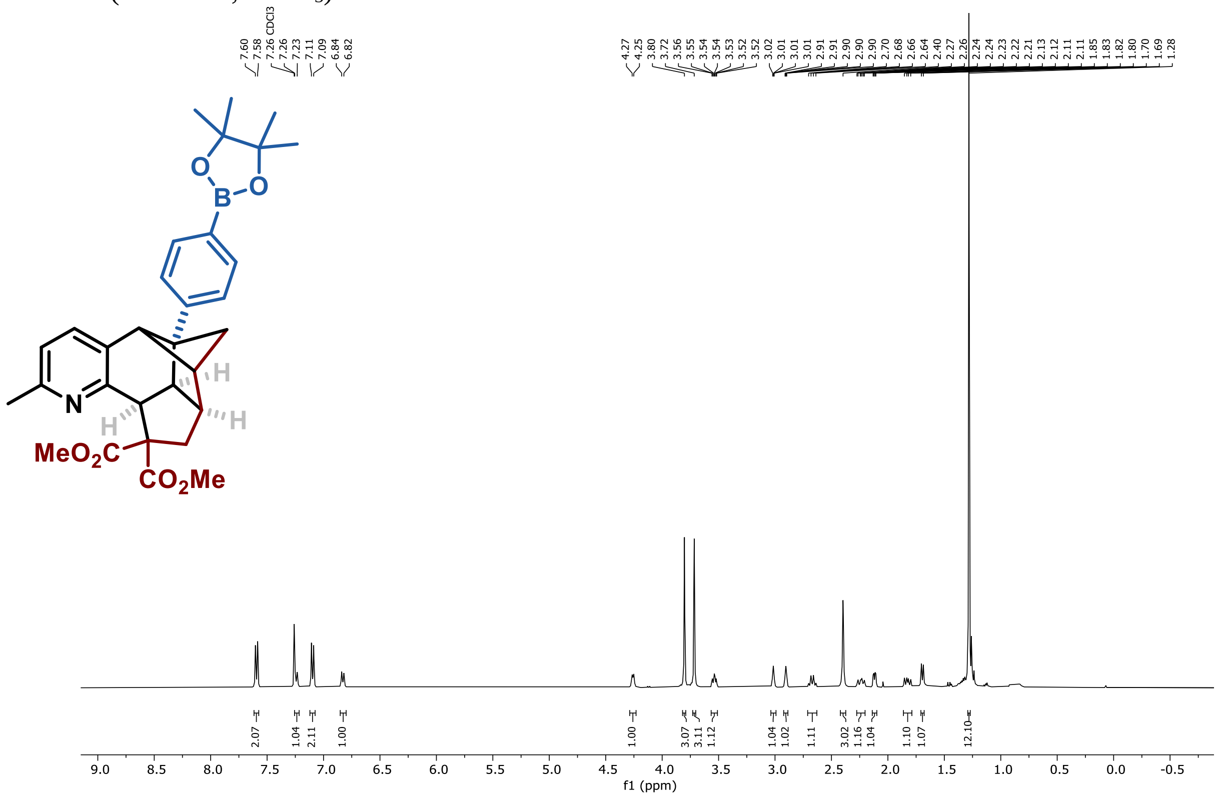

^{13}C NMR (101 MHz, $CDCl_3$) of **5o**

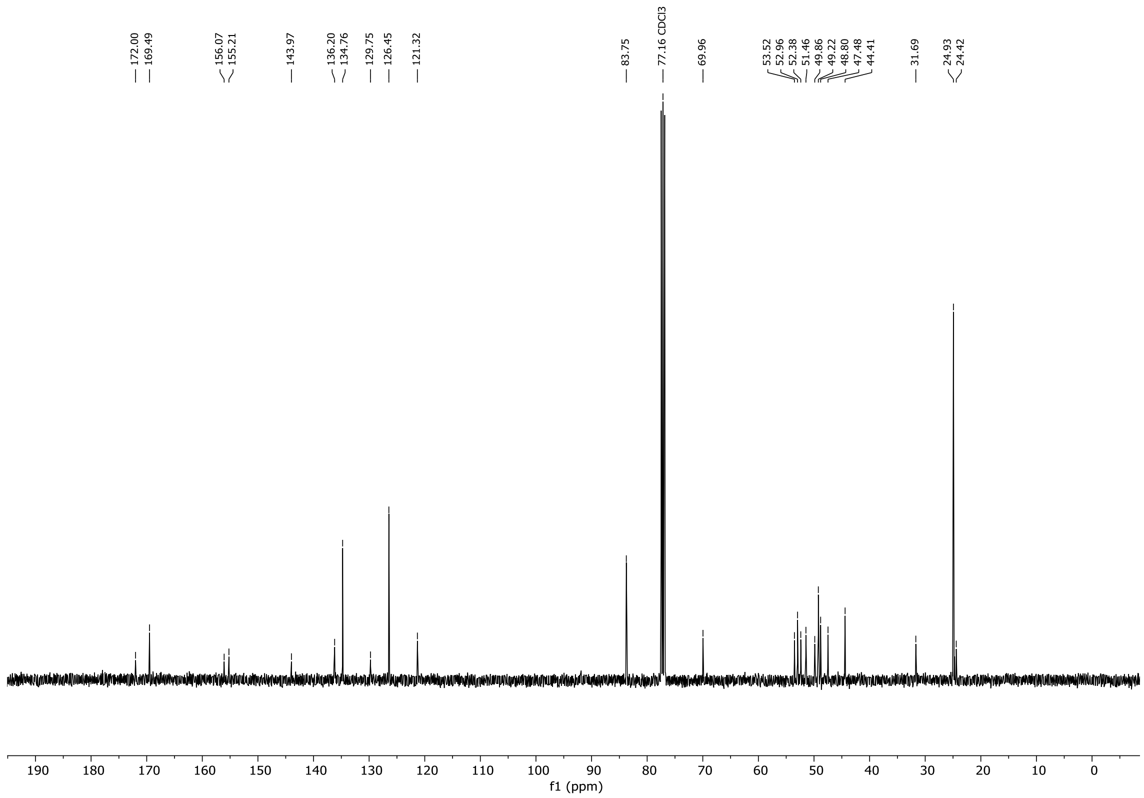

^{11}B NMR (128 MHz, $CDCl_3$) of **5o**

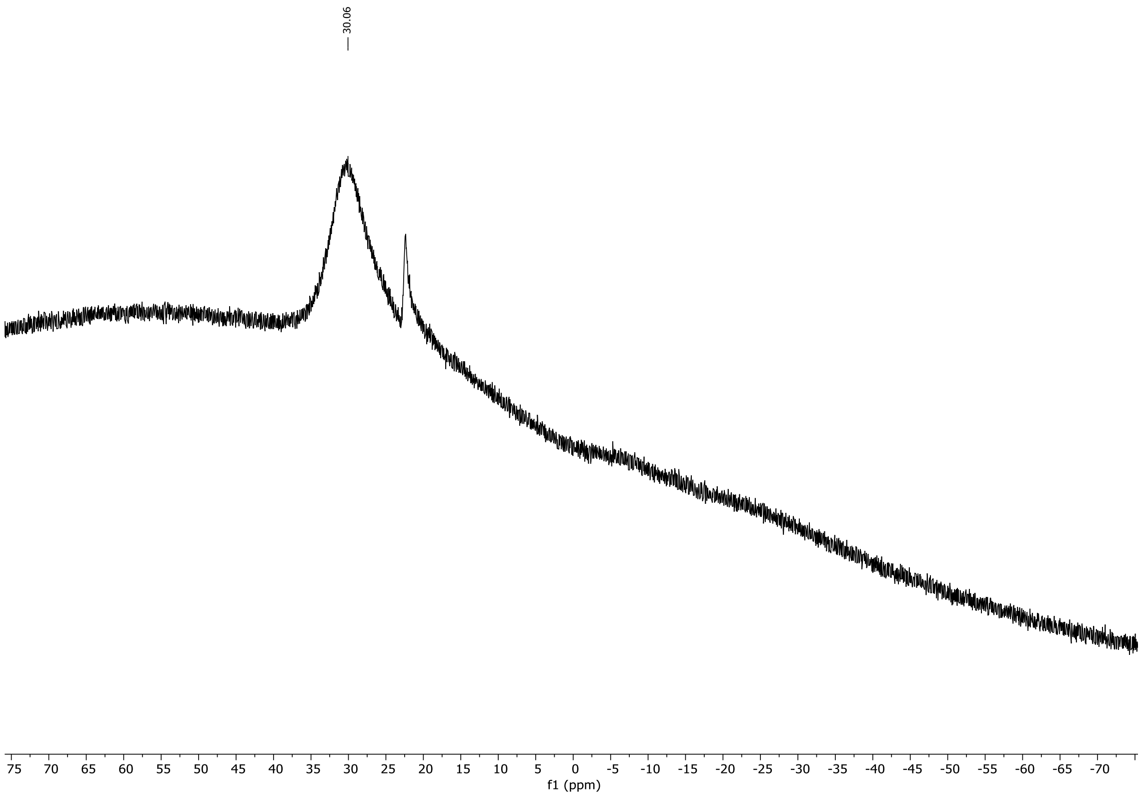

^{1}H NMR (400 MHz, $CDCl_3$) of **5p**

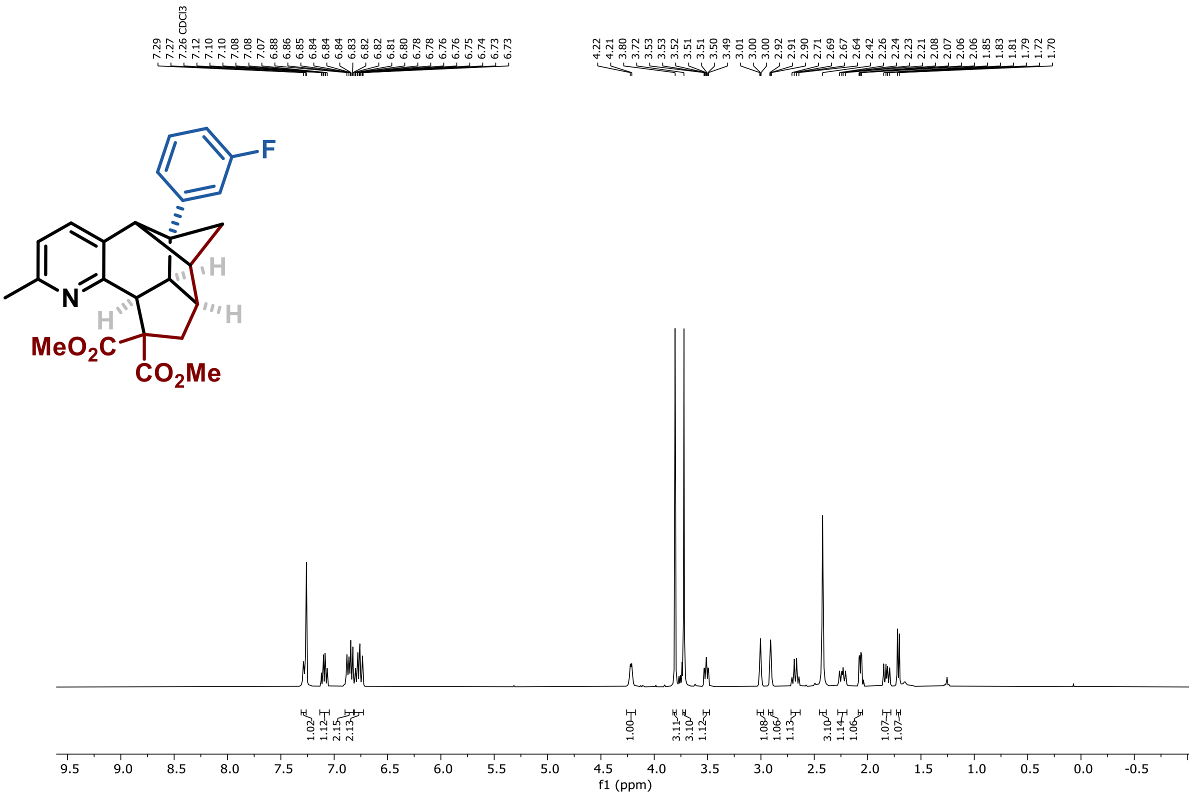

$^{13}C\{^{19}F\}$ NMR (126 MHz, $CDCl_3$) of **5p**

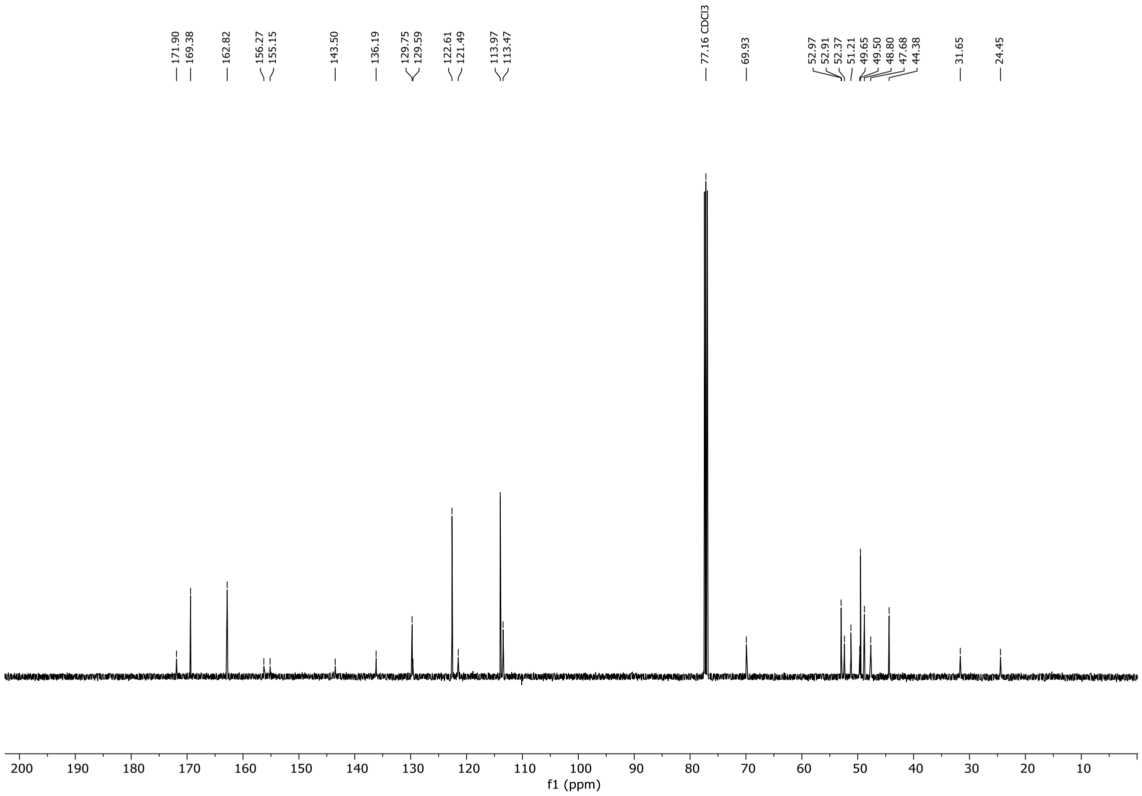

^{19}F NMR (376 MHz, $CDCl_3$) of **5p**

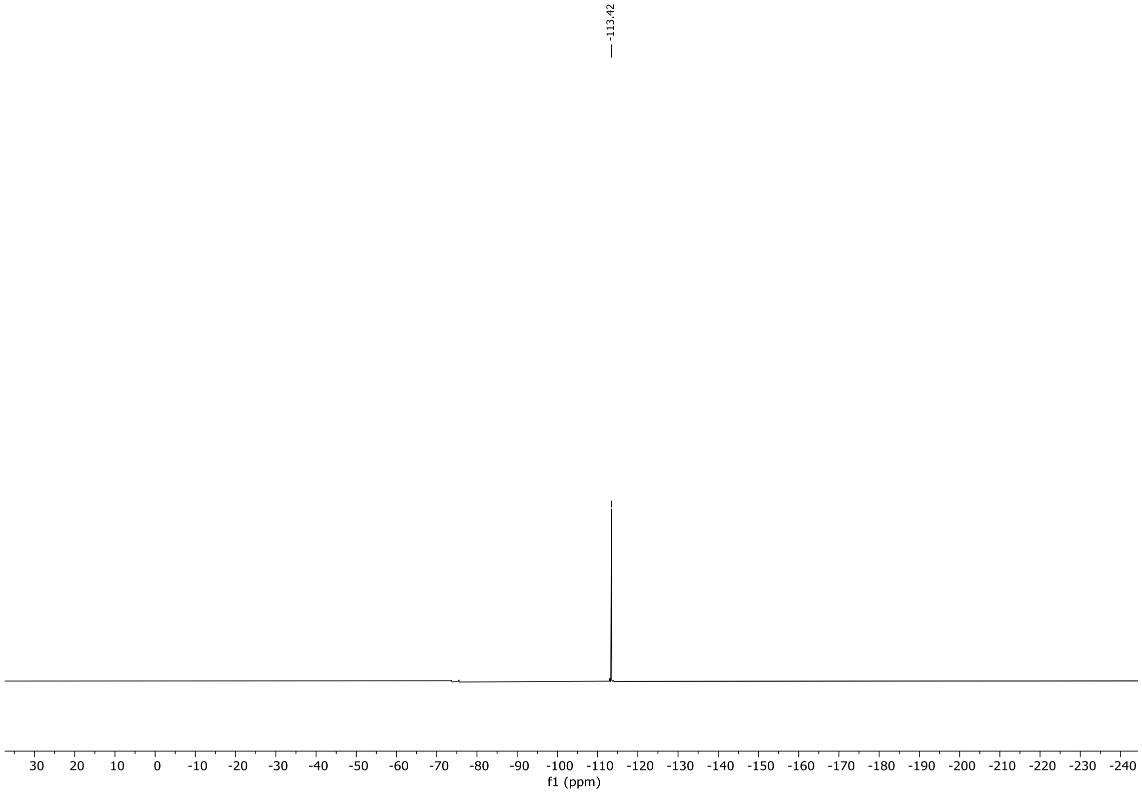

^{1}H NMR (400 MHz, $CDCl_3$) of **5q**

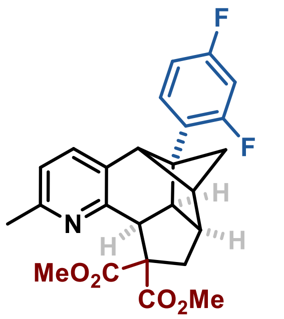

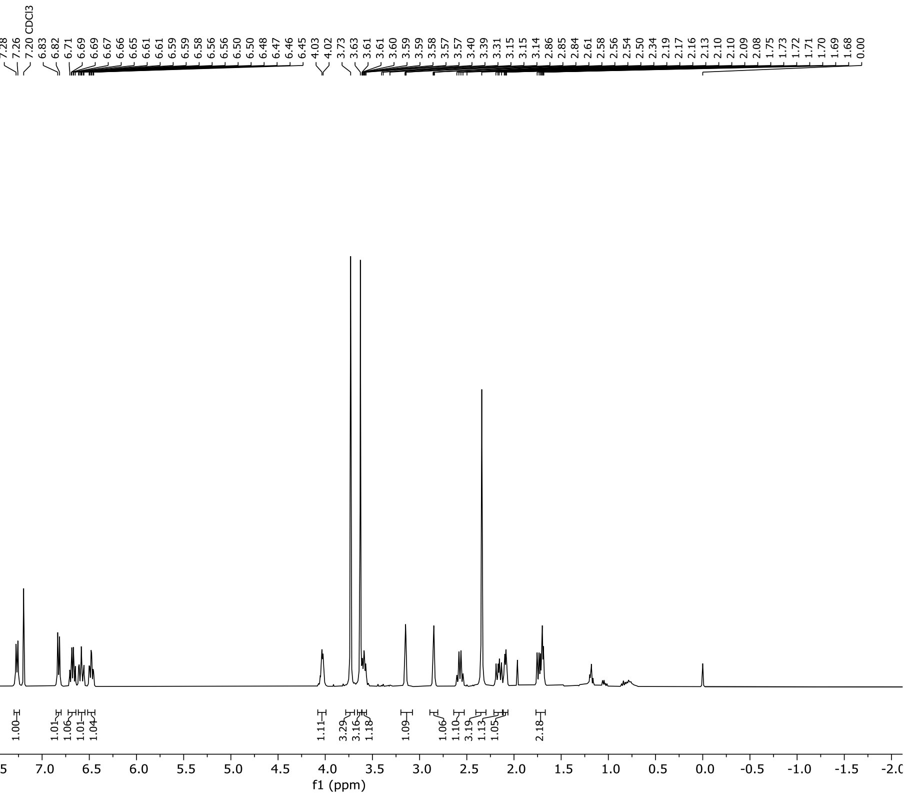

^{13}C{^{19}F} NMR (126 MHz, $CDCl_3$) of **5q**

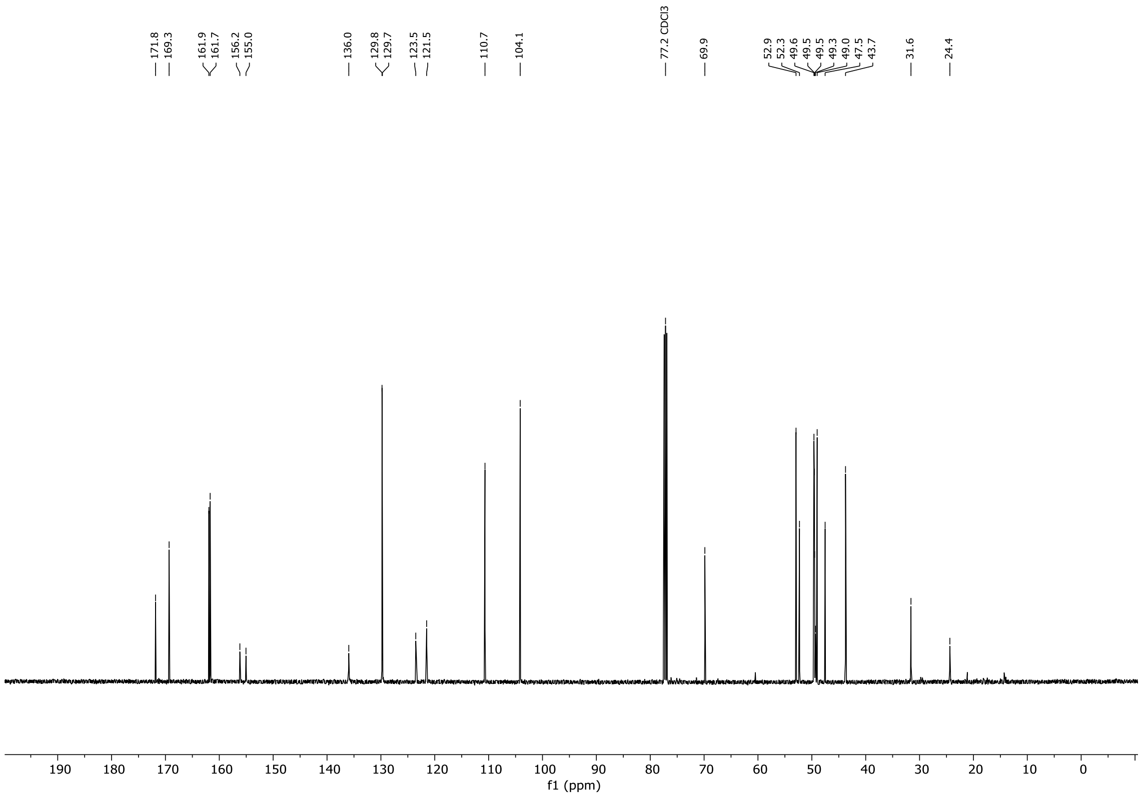

^{19}F NMR (376 MHz, $CDCl_3$) of **5q**

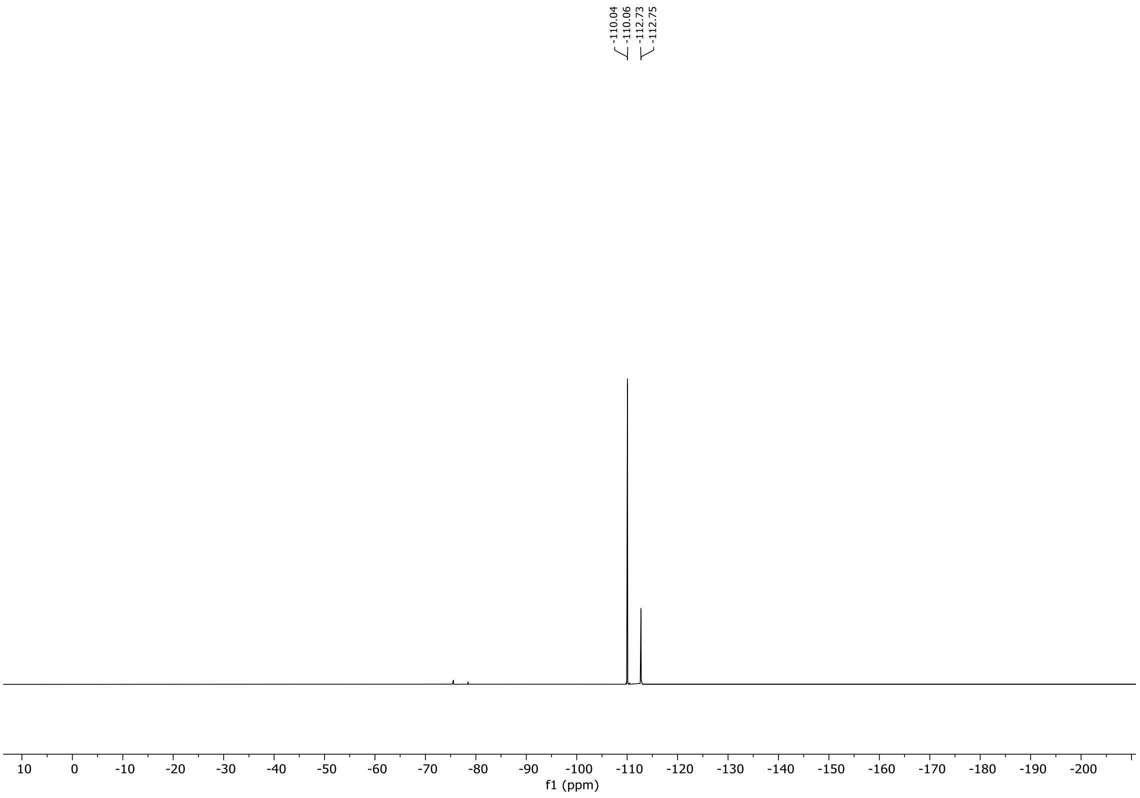

^{1}H NMR (400 MHz, $CDCl_3$) of **5r**

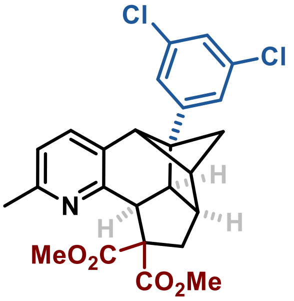

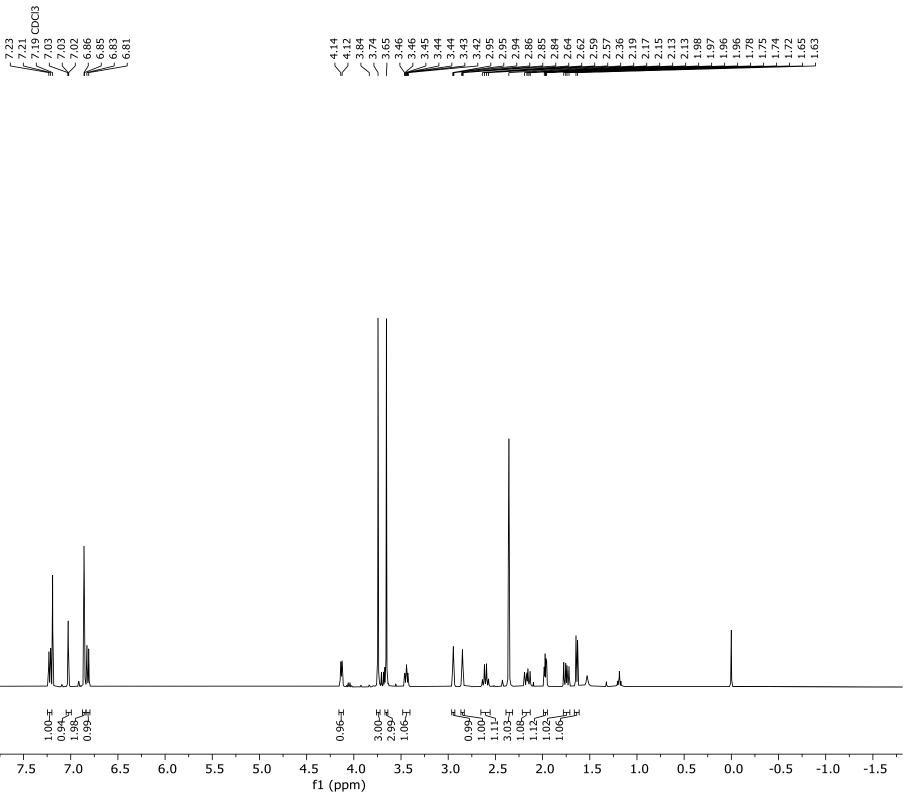

^{13}C NMR (101 MHz, $CDCl_3$) of **5r**

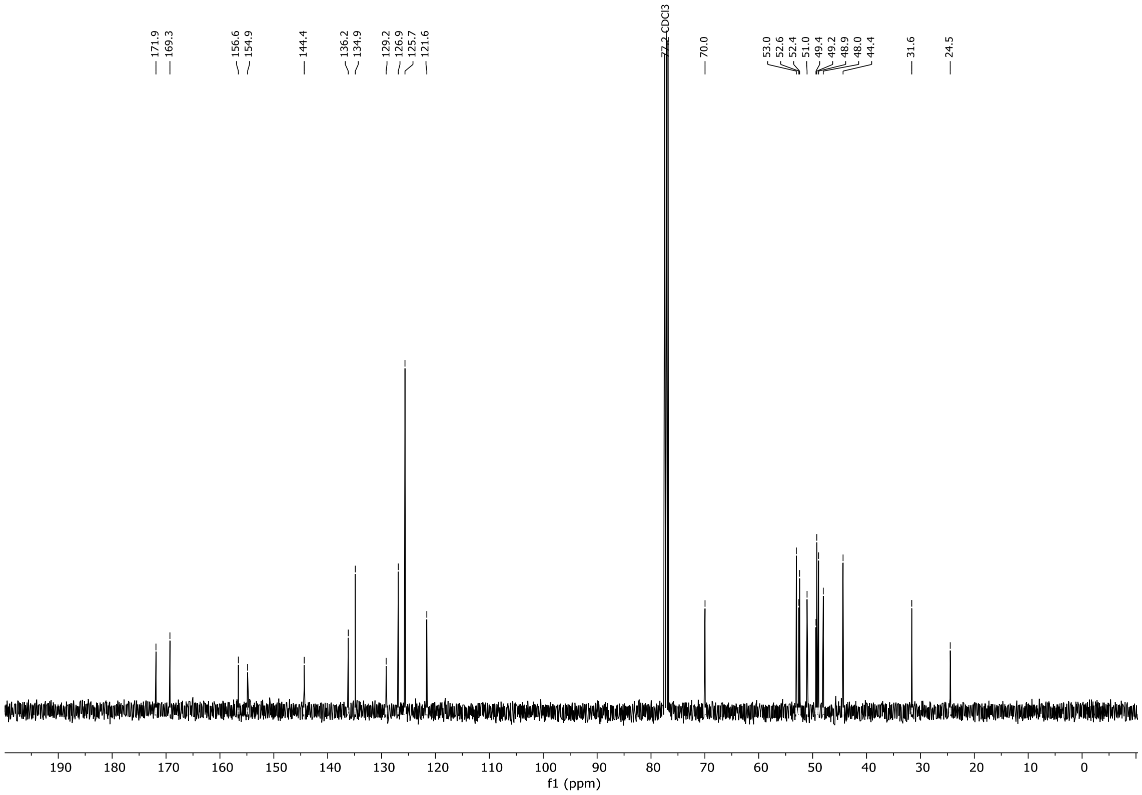

^{1}H NMR (400 MHz, $CDCl_3$) of **5s**

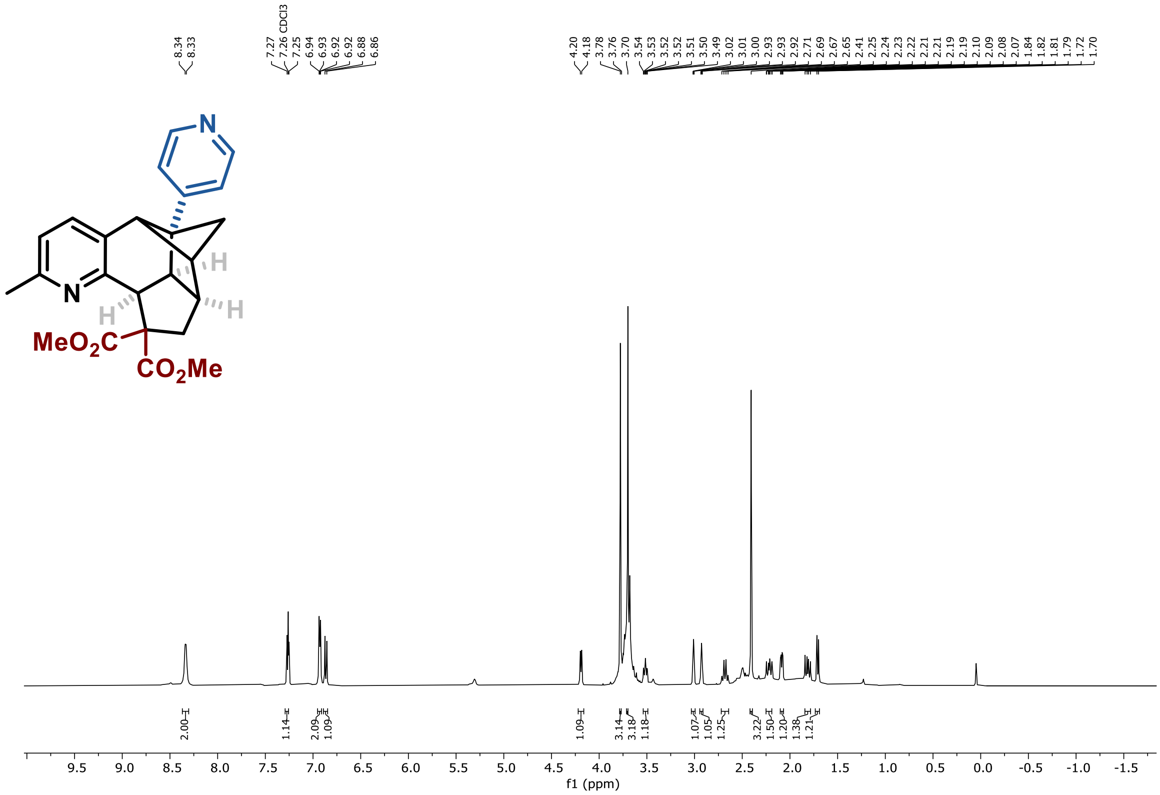

^{13}C NMR (101 MHz, $CDCl_3$) of **5s**

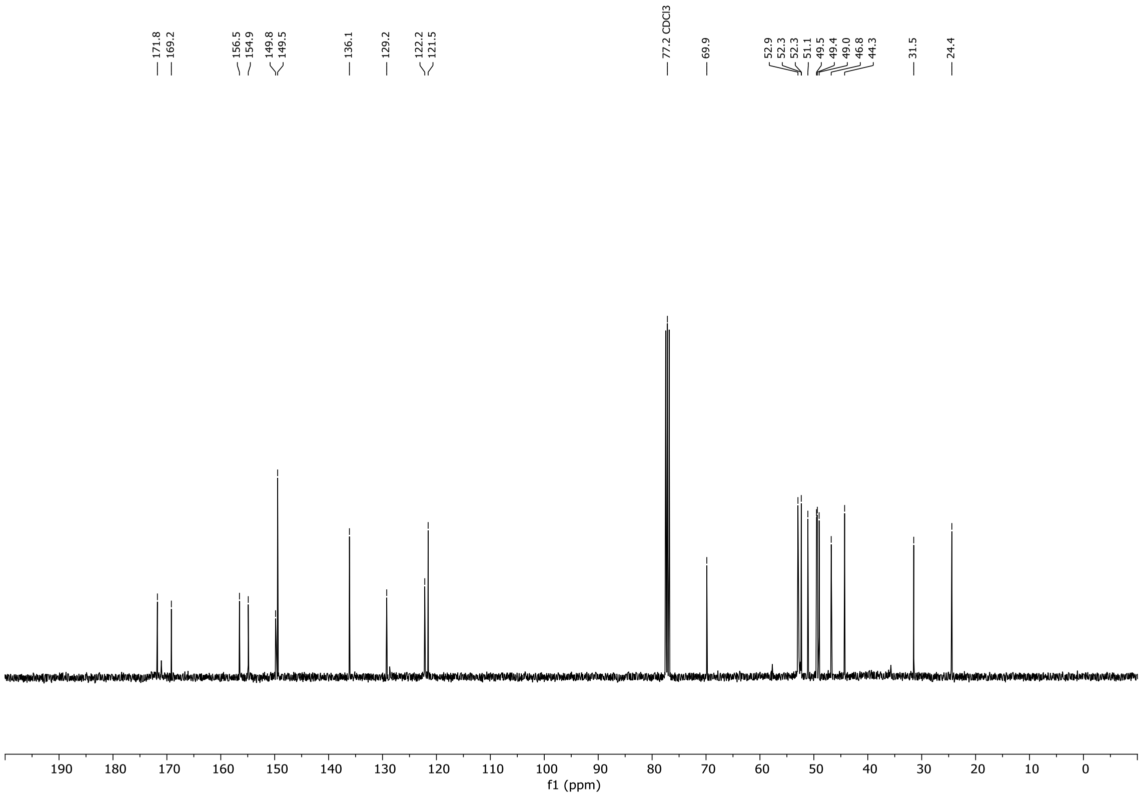

^{1}H NMR (400 MHz, $CDCl_3$) of **5t**

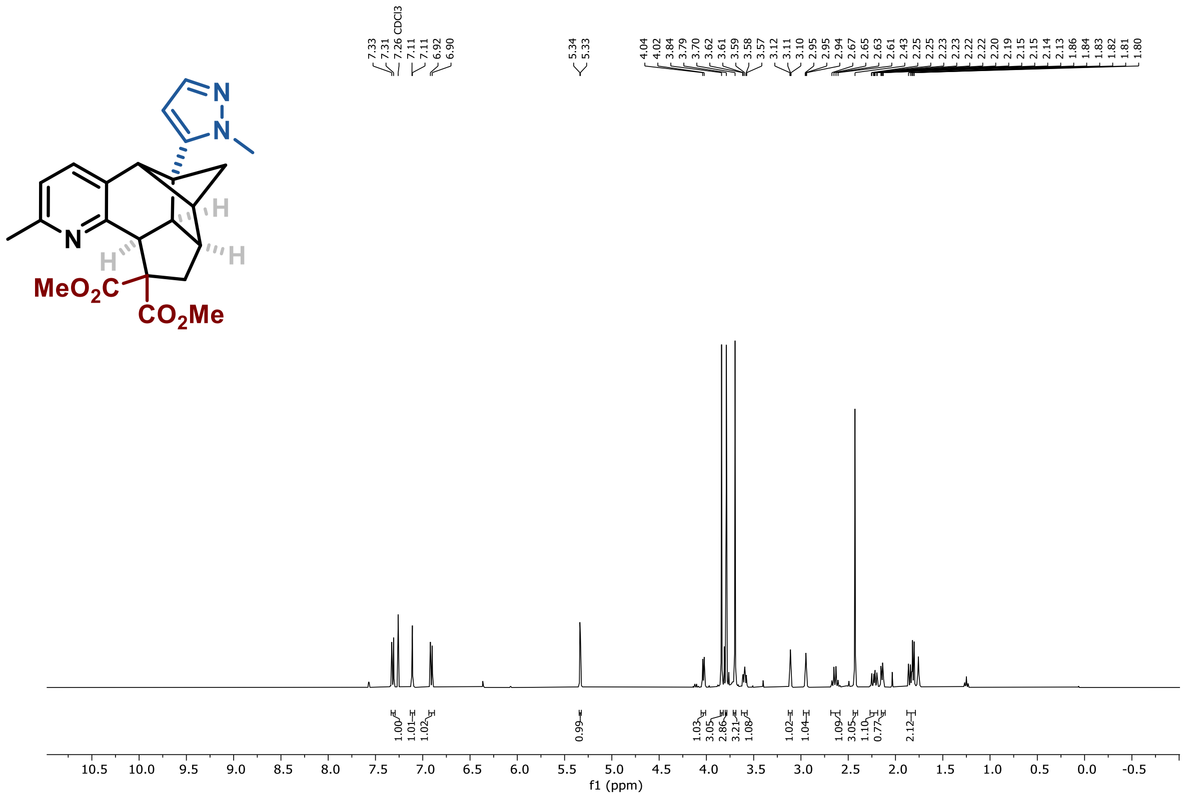

^{13}C NMR (101 MHz, $CDCl_3$) of **5t**

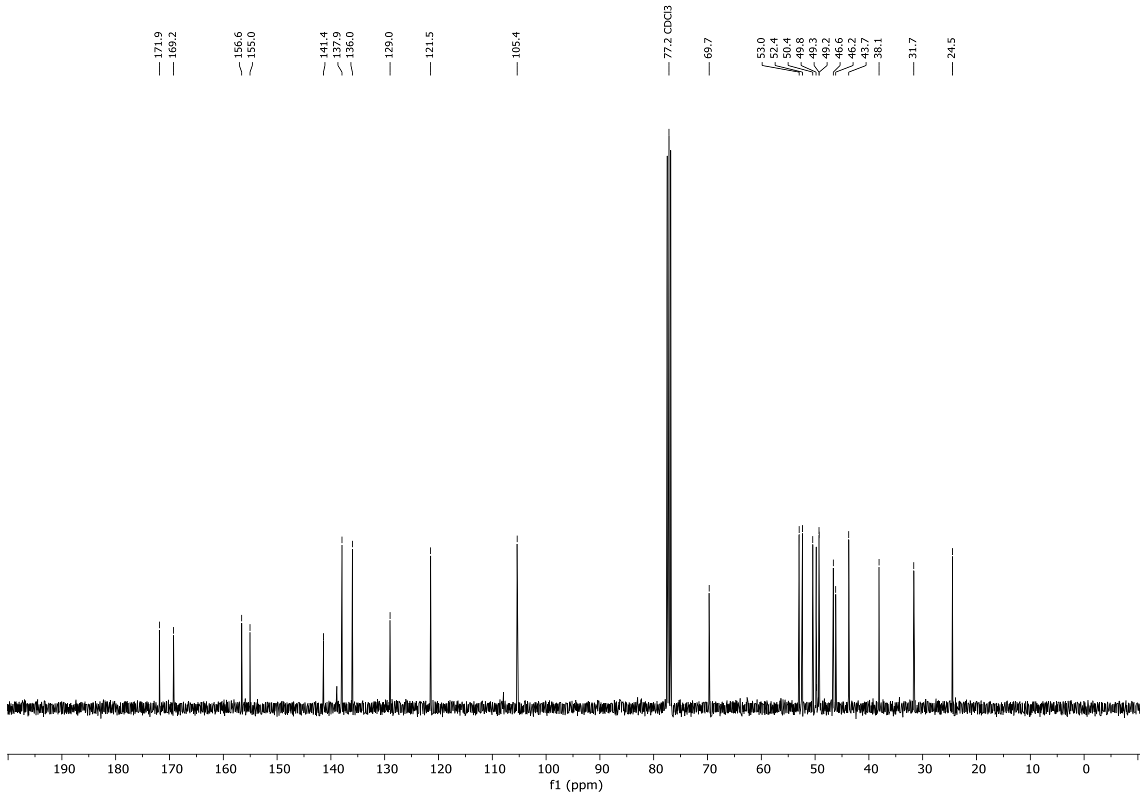

^{1}H NMR (400 MHz, $CDCl_3$) of **5u**

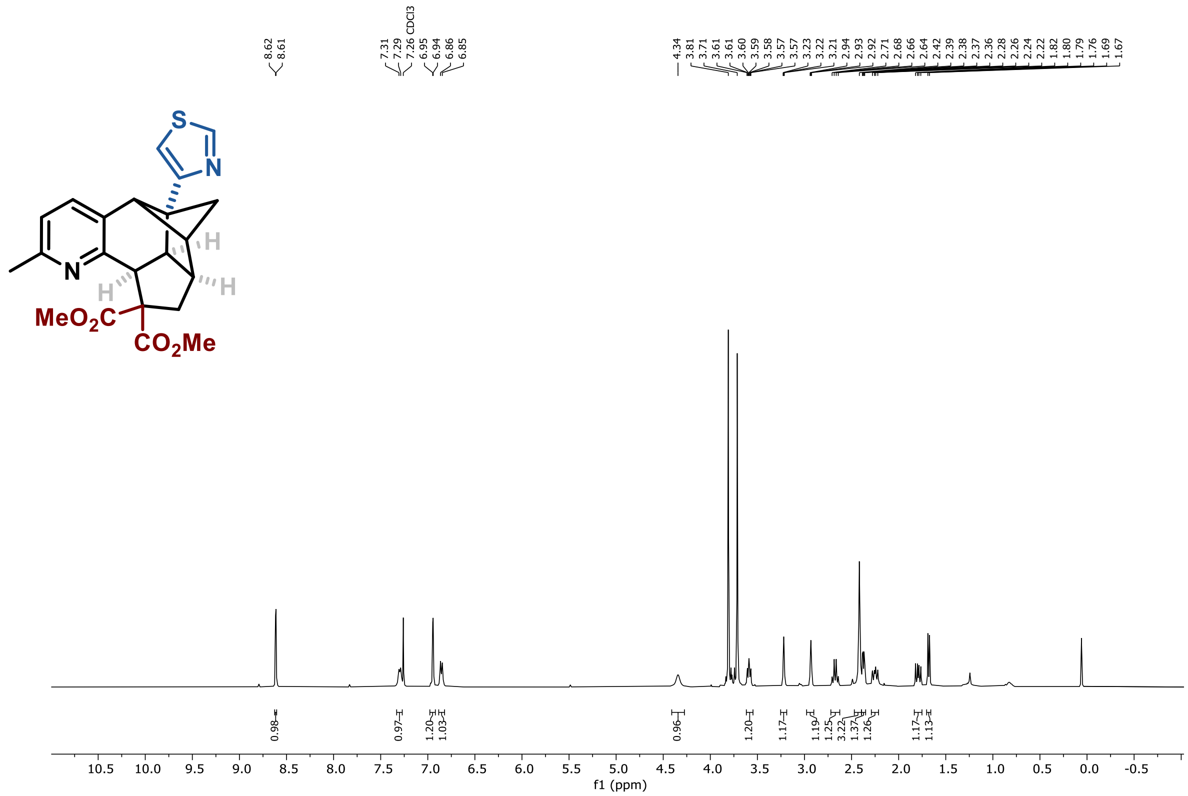

^{13}C NMR (101 MHz, $CDCl_3$) of **5u**

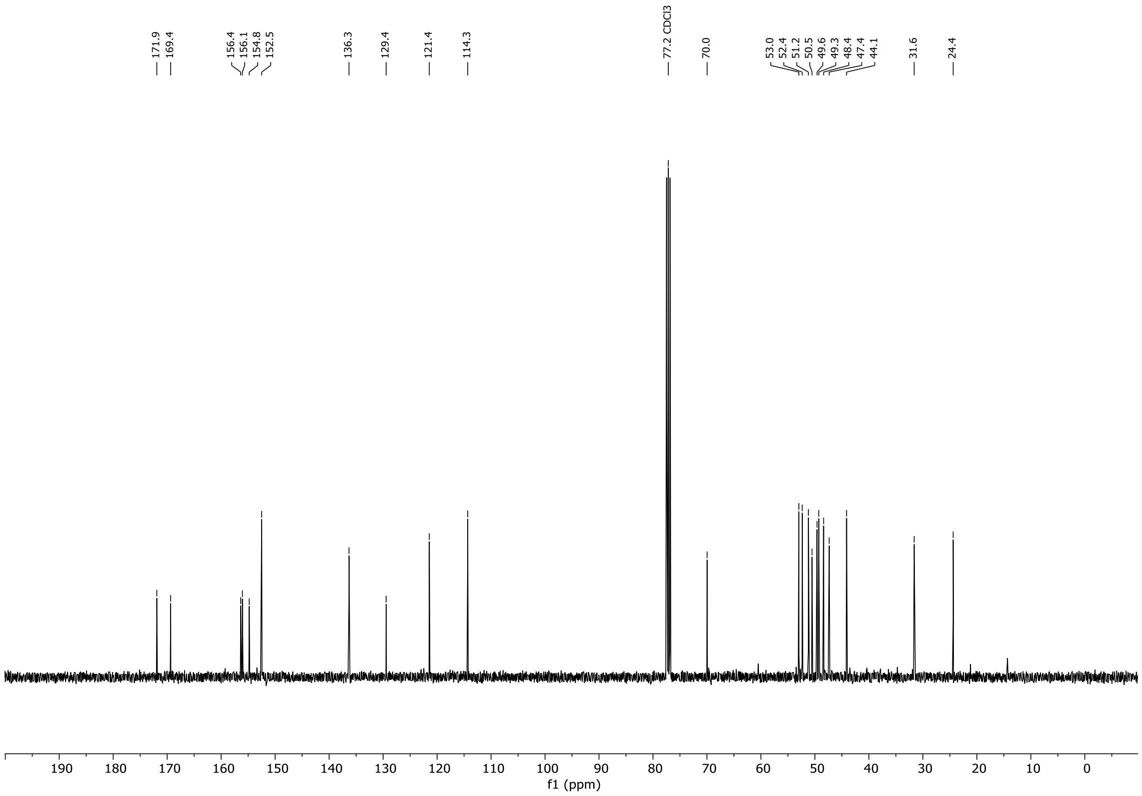

^{1}H NMR (400 MHz, $CDCl_3$) of **5v**

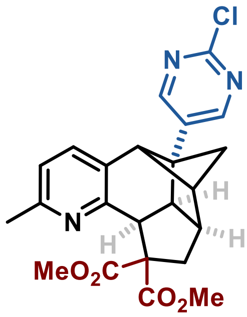

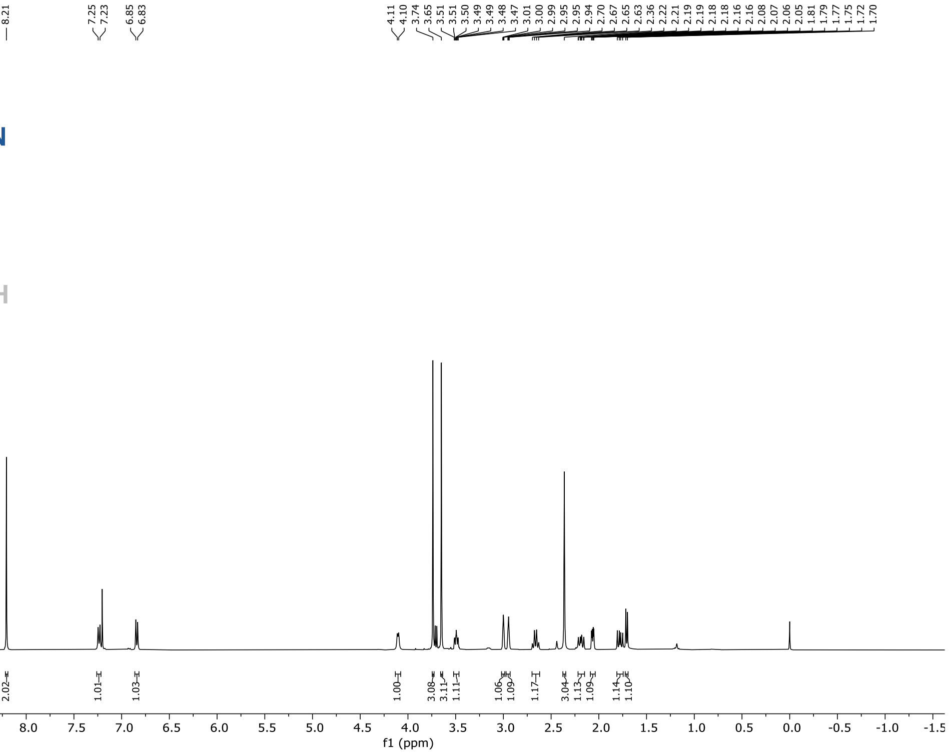

^{13}C NMR (101 MHz, $CDCl_3$) of **5v**

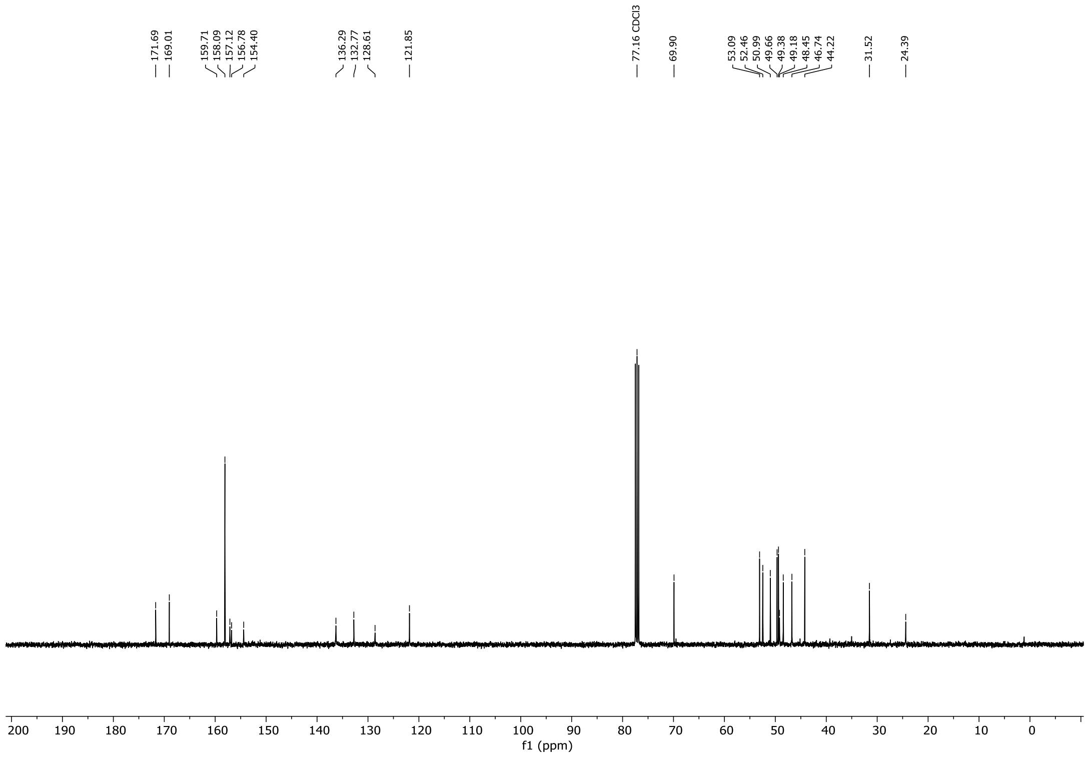

^{1}H NMR (400 MHz, $CDCl_3$) of **5x**

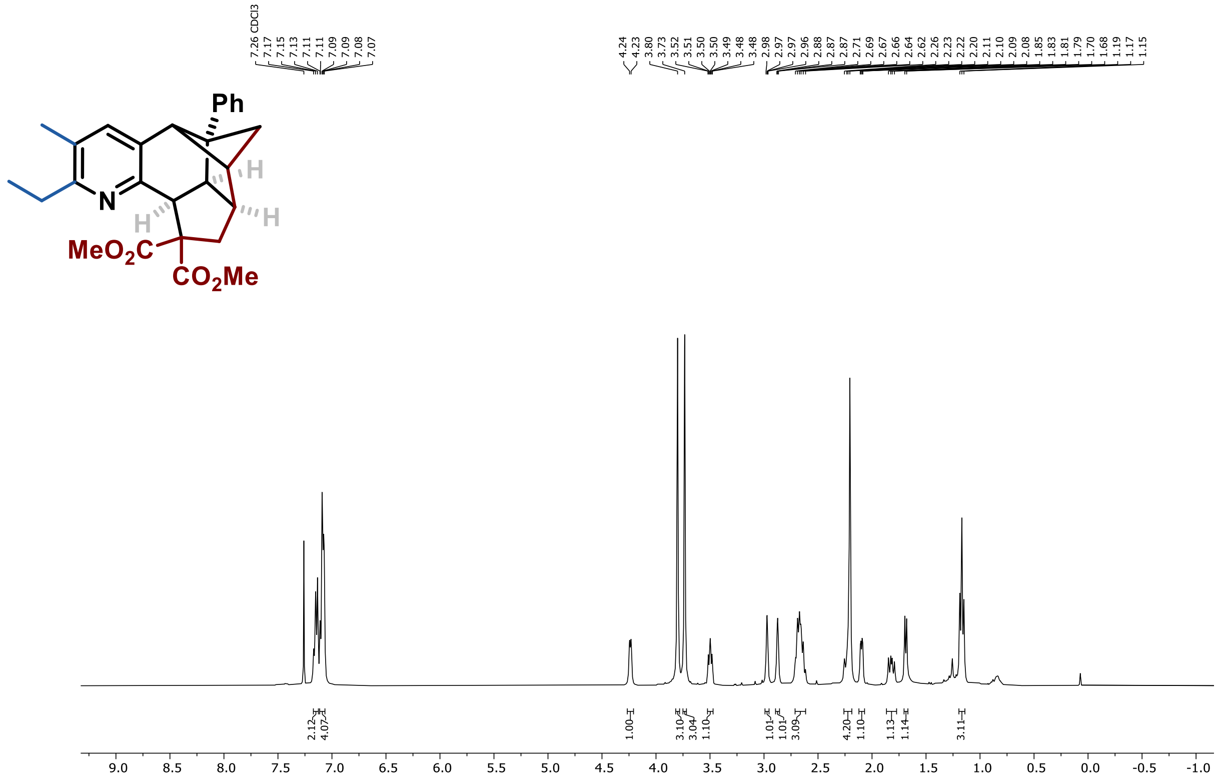

^{13}C NMR (101 MHz, $CDCl_3$) of **5x**

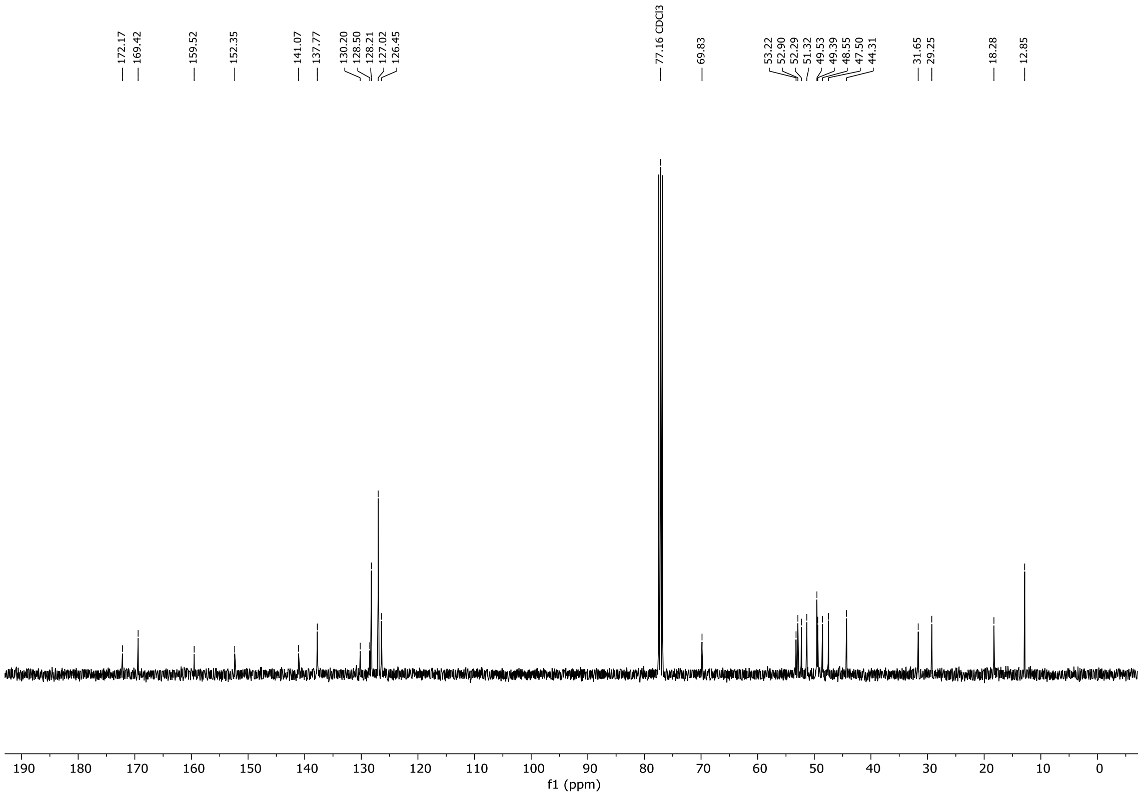

^{1}H NMR (400 MHz, $CDCl_3$) of **5y**

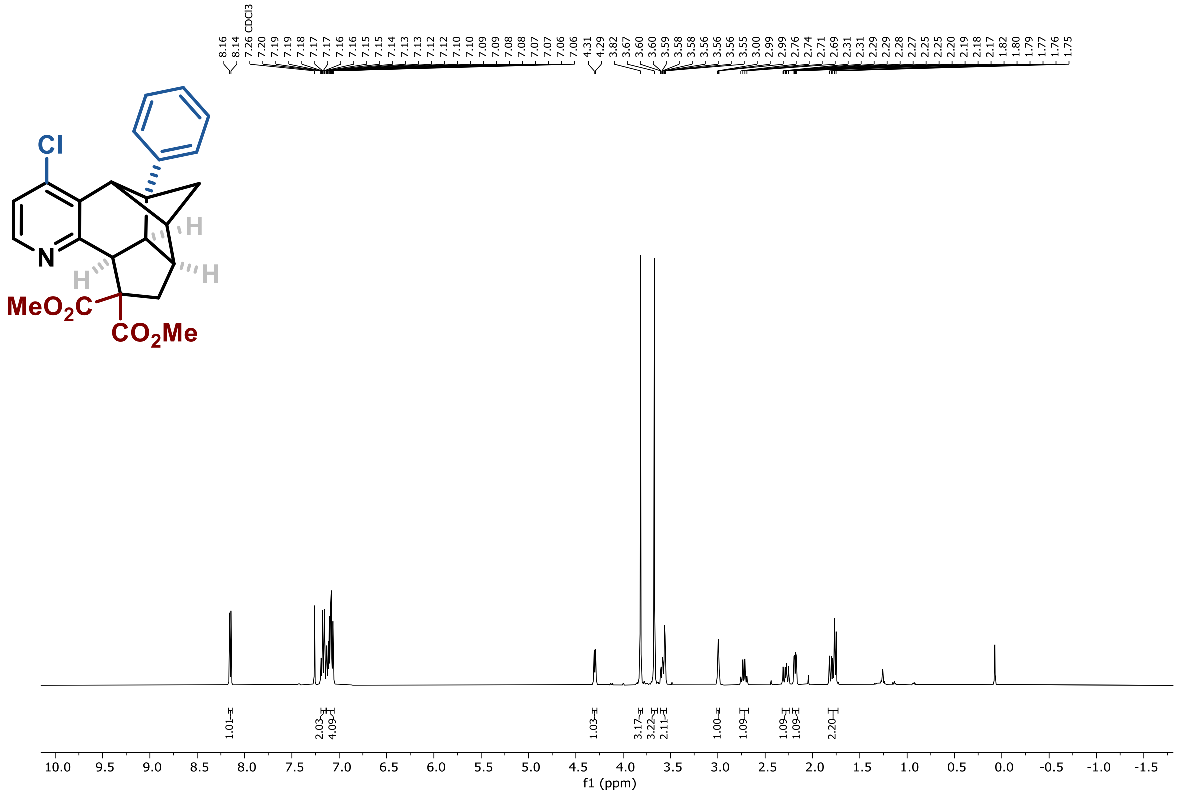

^{13}C NMR (101 MHz, $CDCl_3$) of **5y**

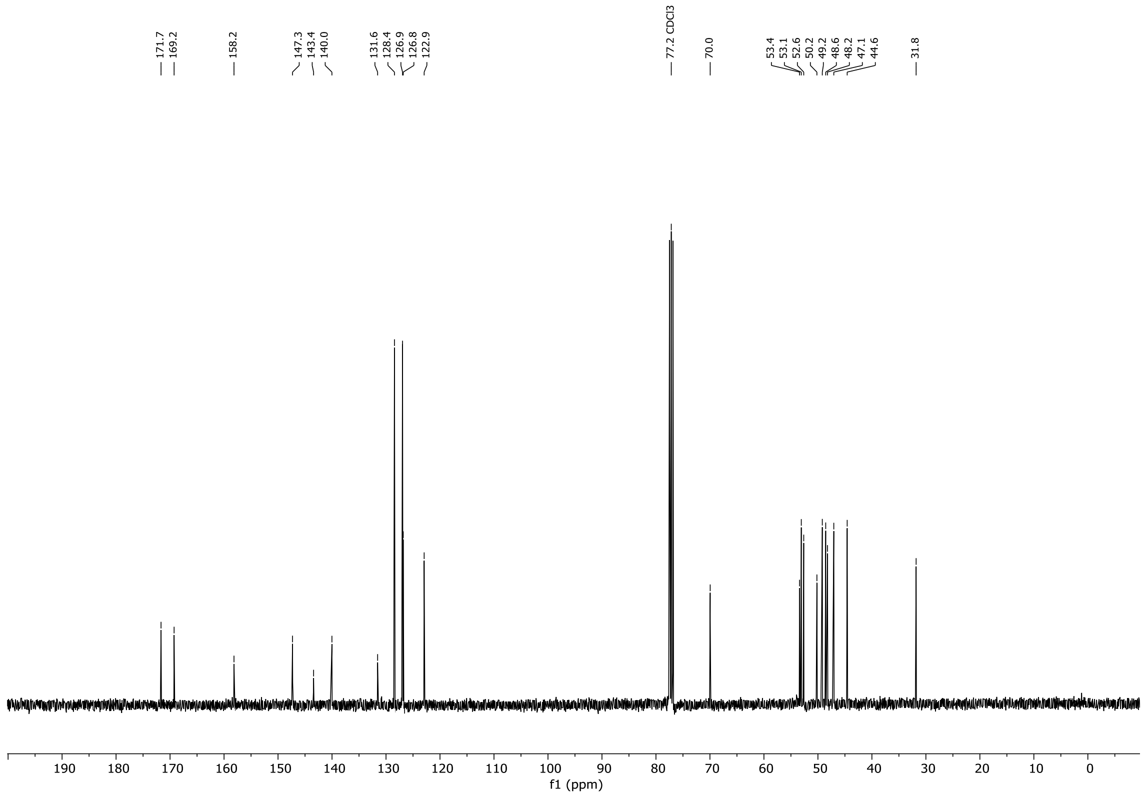

^{1}H NMR (400 MHz, $CDCl_3$) of **5z**

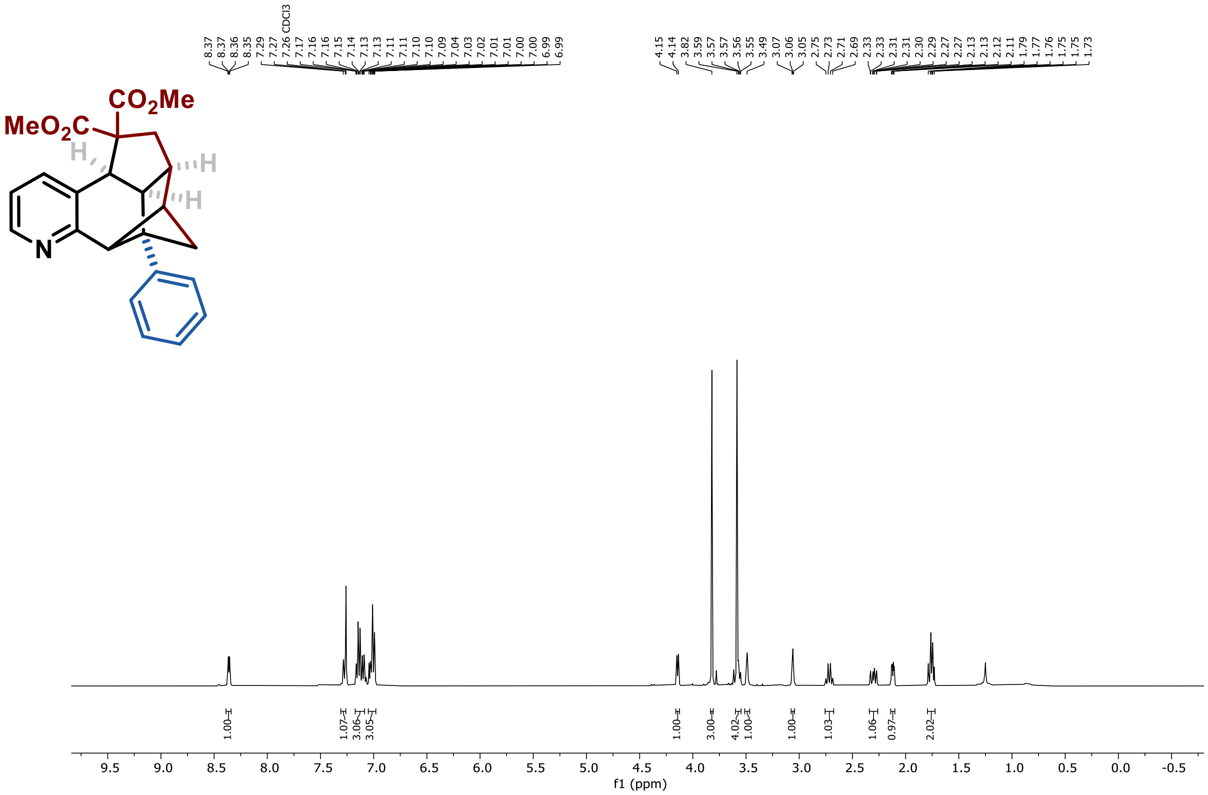

^{13}C NMR (101 MHz, $CDCl_3$) of **5z**

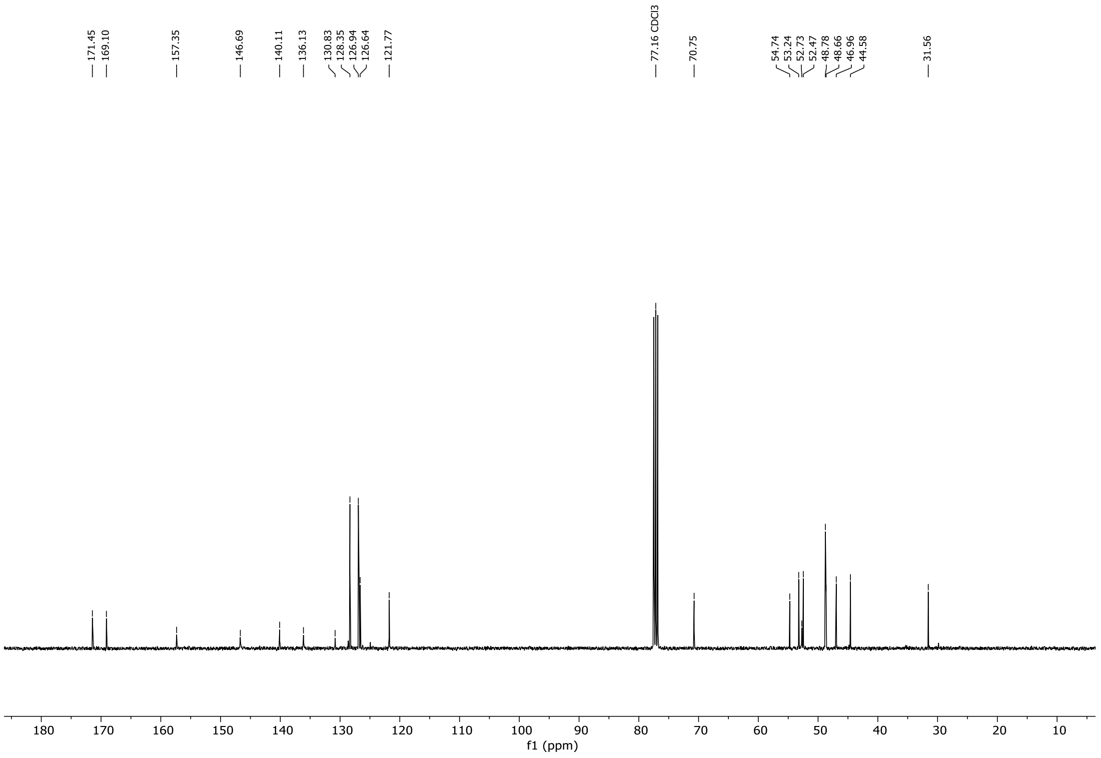

^{1}H NMR (599 MHz, $CDCl_3$) of **5aa**

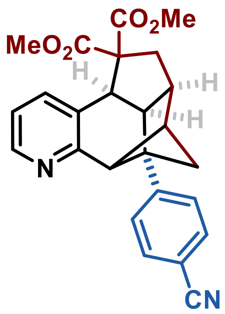

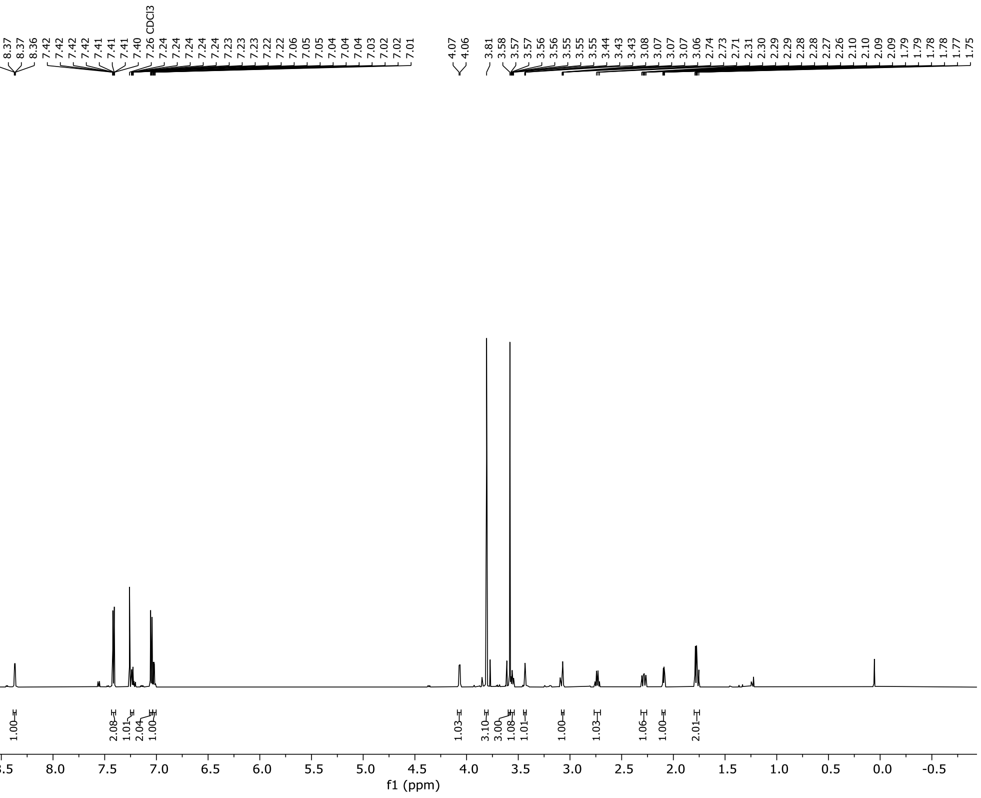

^{13}C NMR (101 MHz, $CDCl_3$) of **5aa**

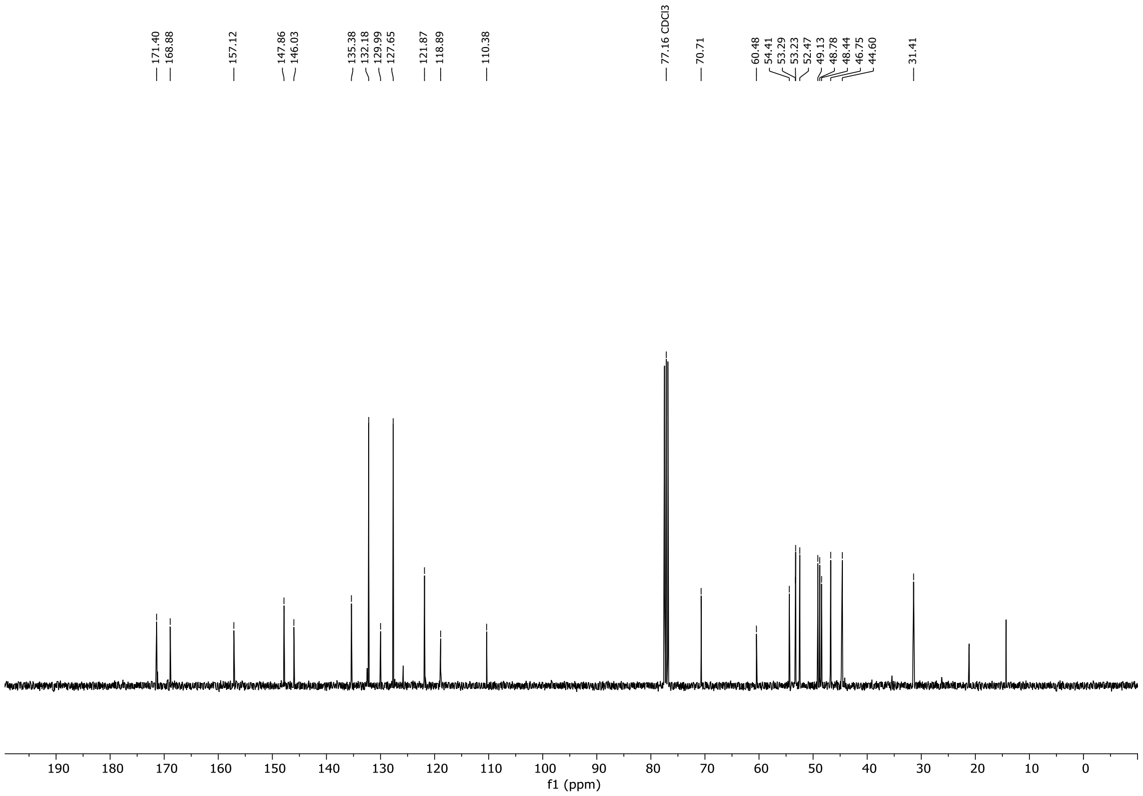

^{1}H NMR (400 MHz, $CDCl_3$) of **5ab**

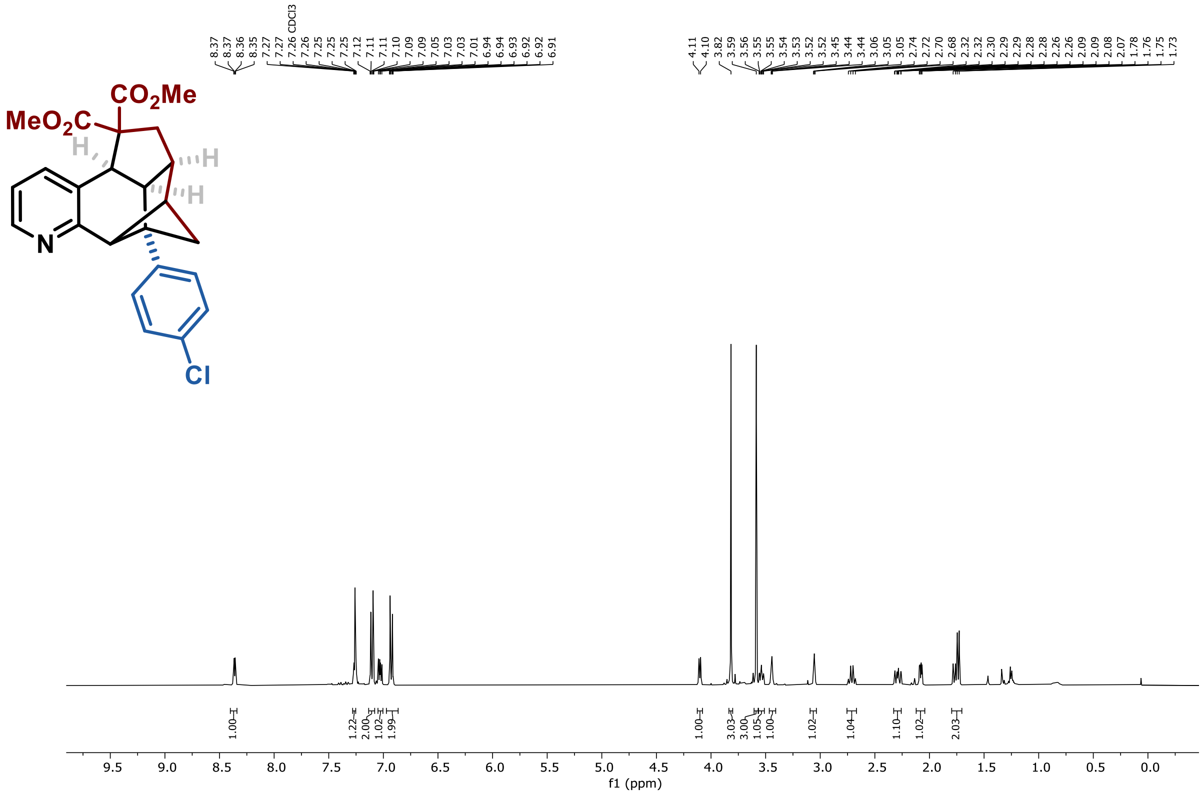

^{13}C NMR (101 MHz, $CDCl_3$) of **5ab**

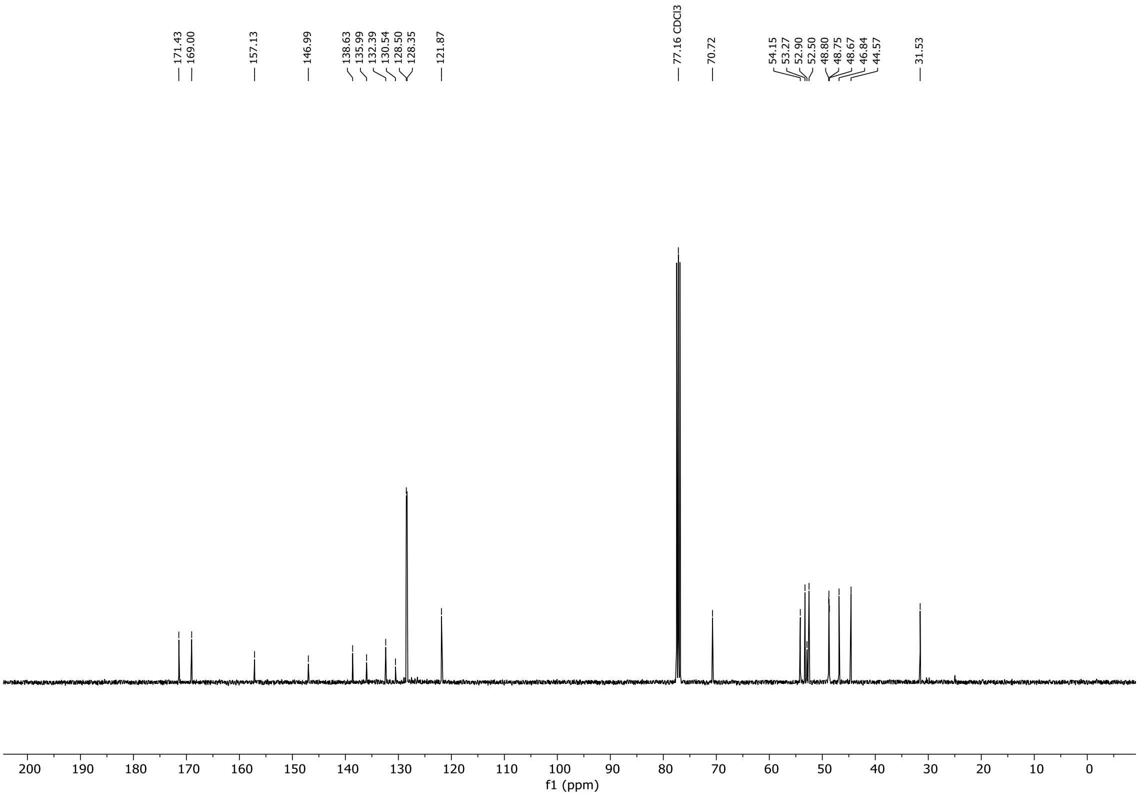

^{1}H NMR (400 MHz, $CDCl_3$) of **5ac**

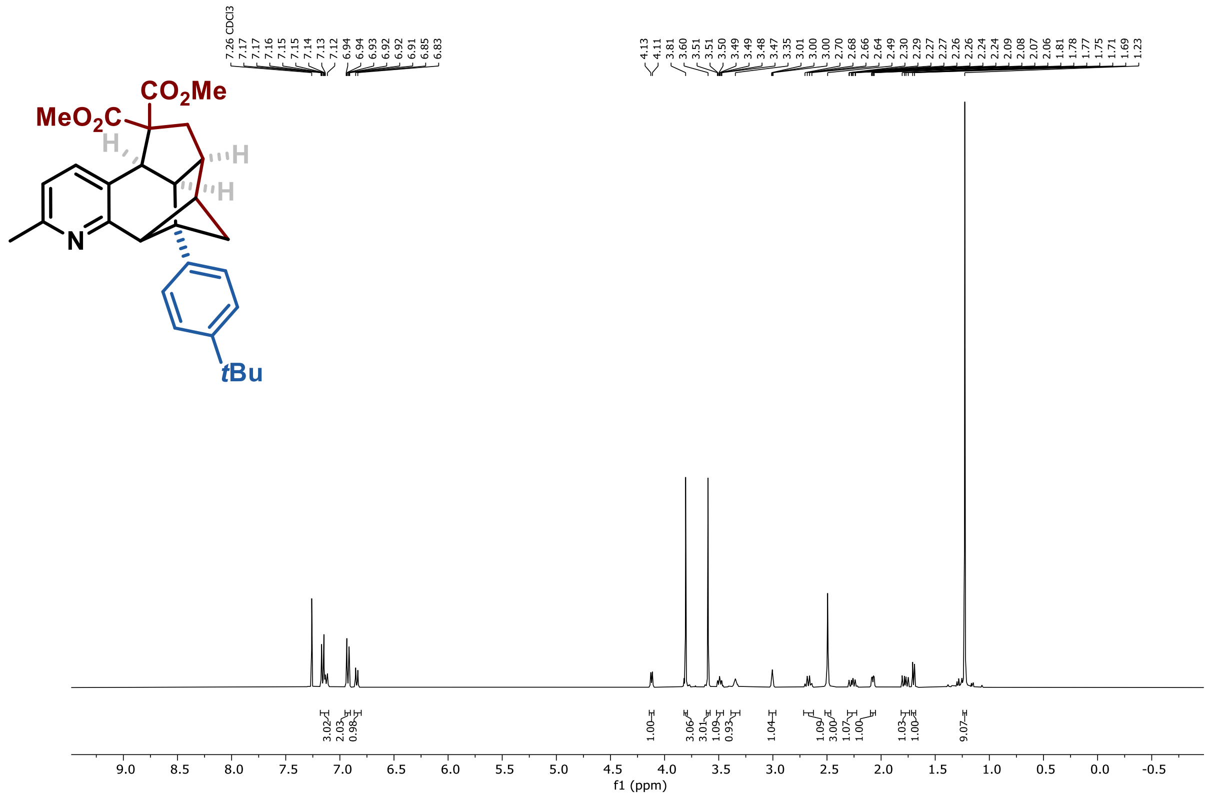

^{13}C NMR (101 MHz, $CDCl_3$) of **5ac**

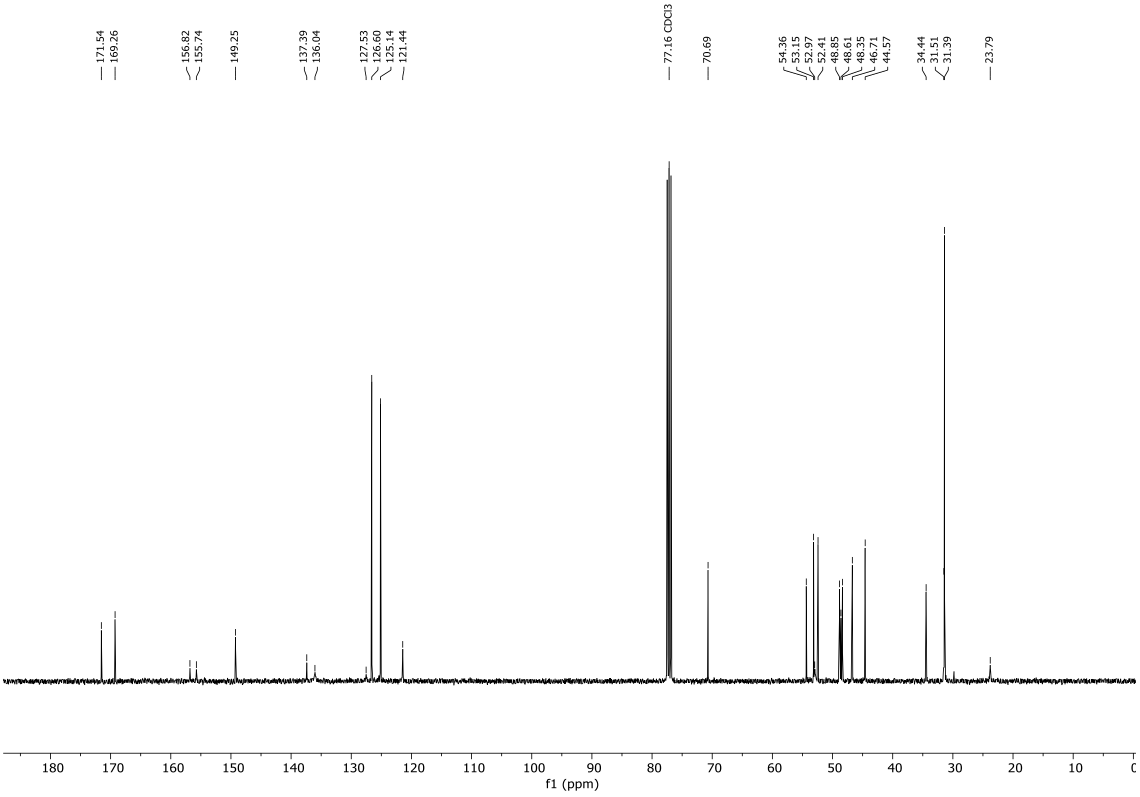

^{1}H NMR (400 MHz, $CDCl_3$) of **5ad**

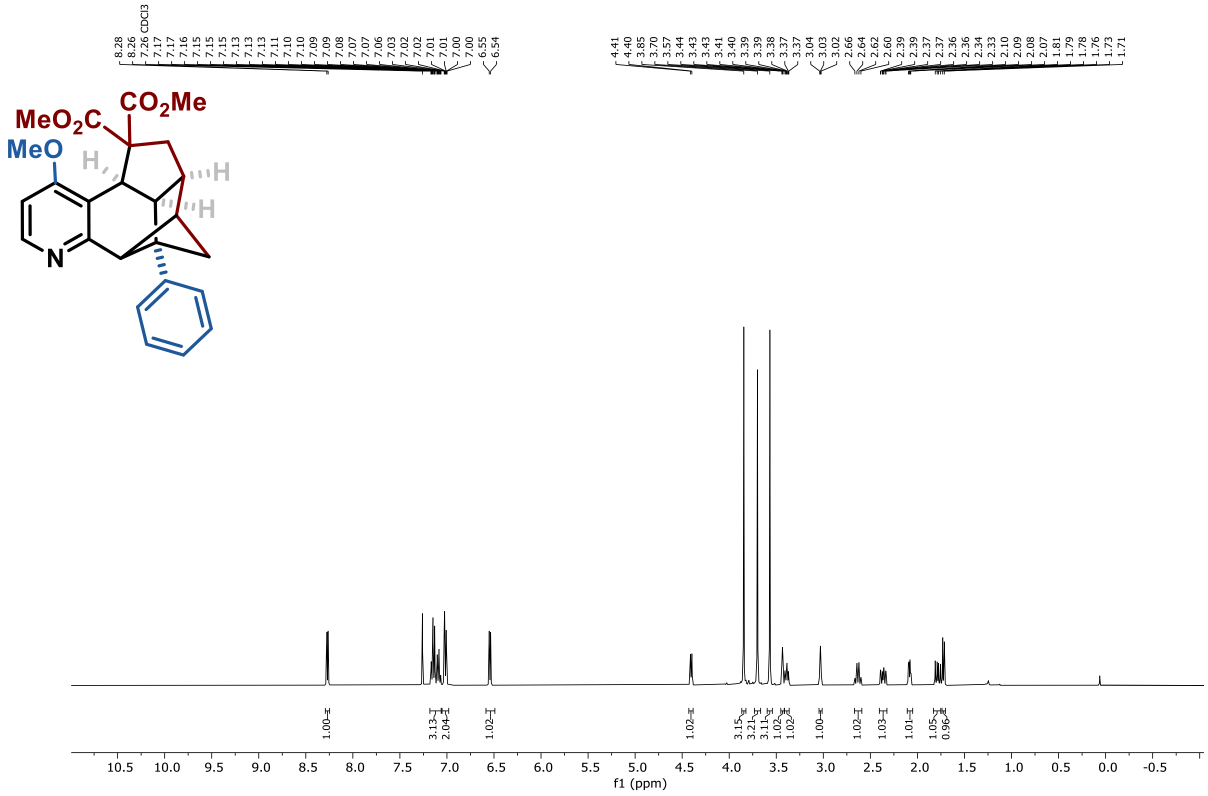

^{13}C NMR (101 MHz, $CDCl_3$) of **5ad**

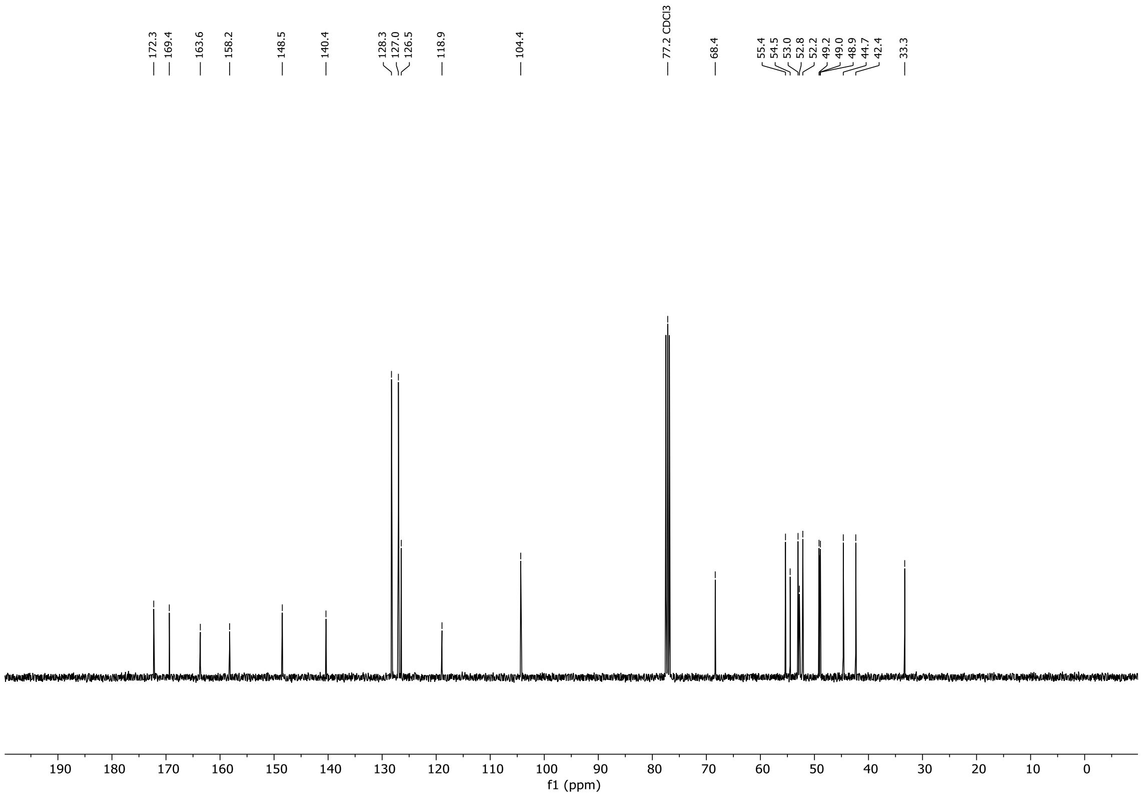

^{1}H, ^{1}H COSY (599 Hz, $CDCl_3$) of **5ab**

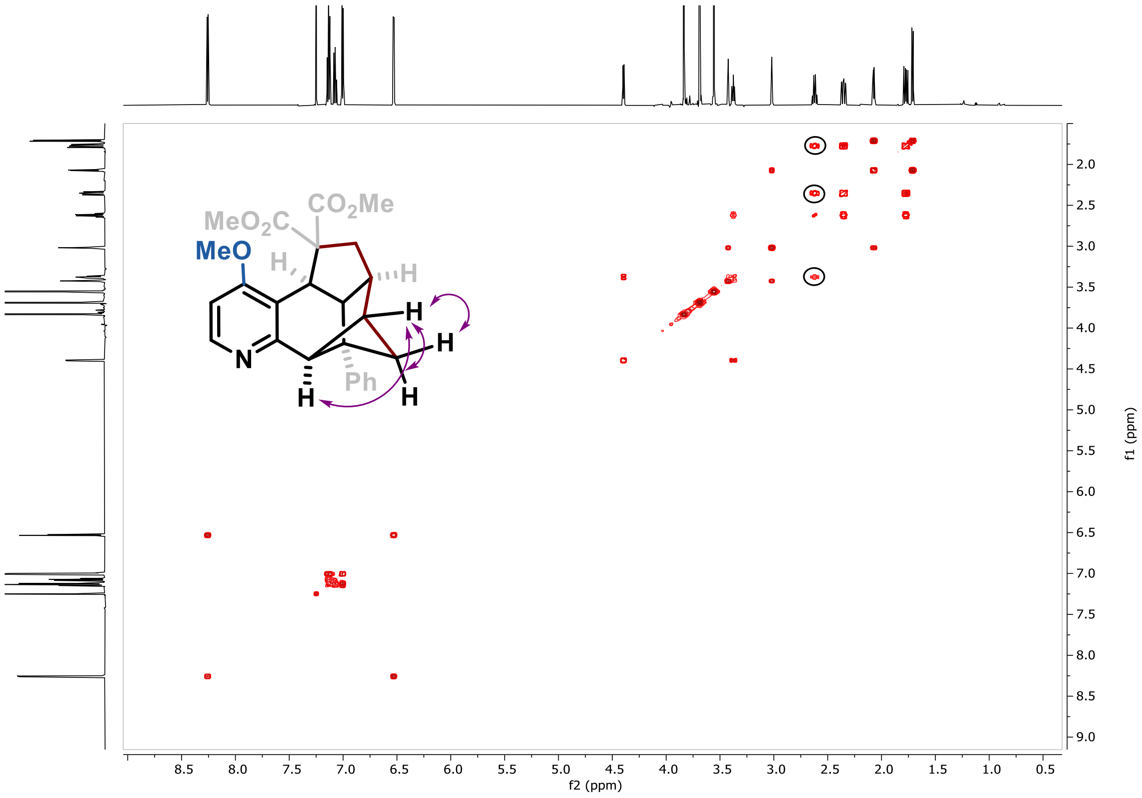

^{1}H, ^{13}C HSQC (599 Hz, $CDCl_3$) of **5ad**

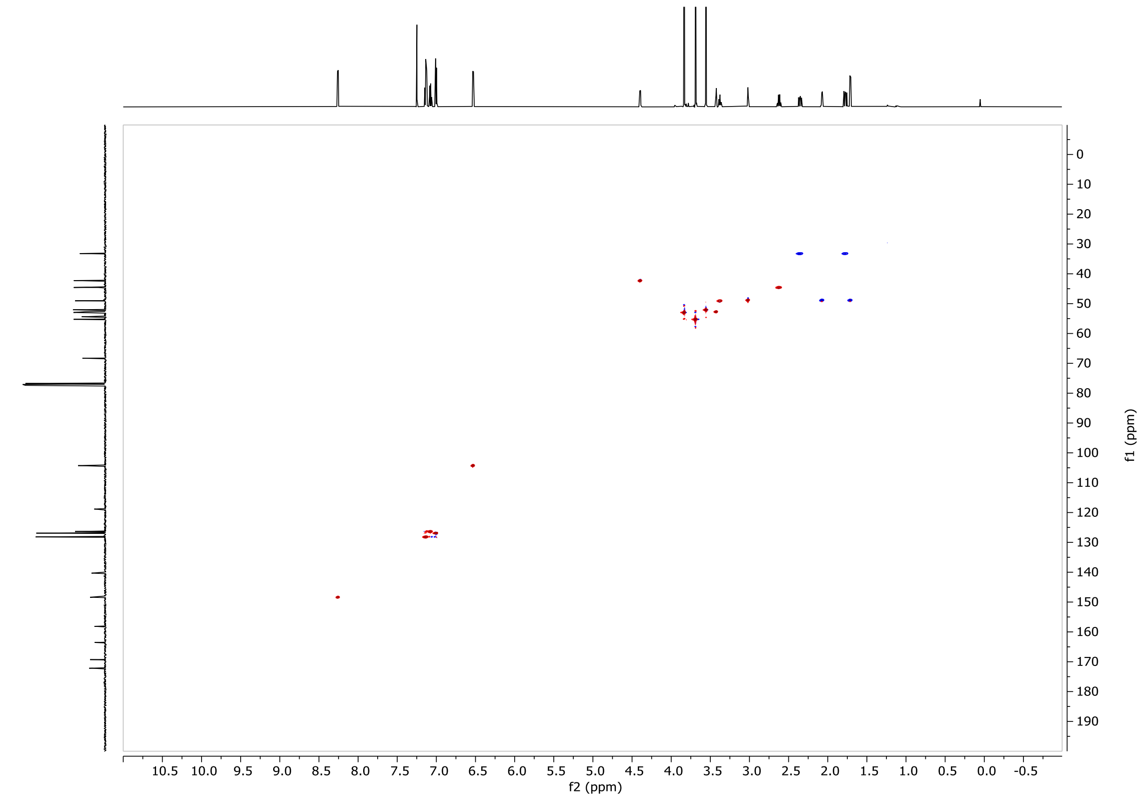

^{1}H, ^{13}C HMBC (599 Hz, $CDCl_3$) of **5ad**

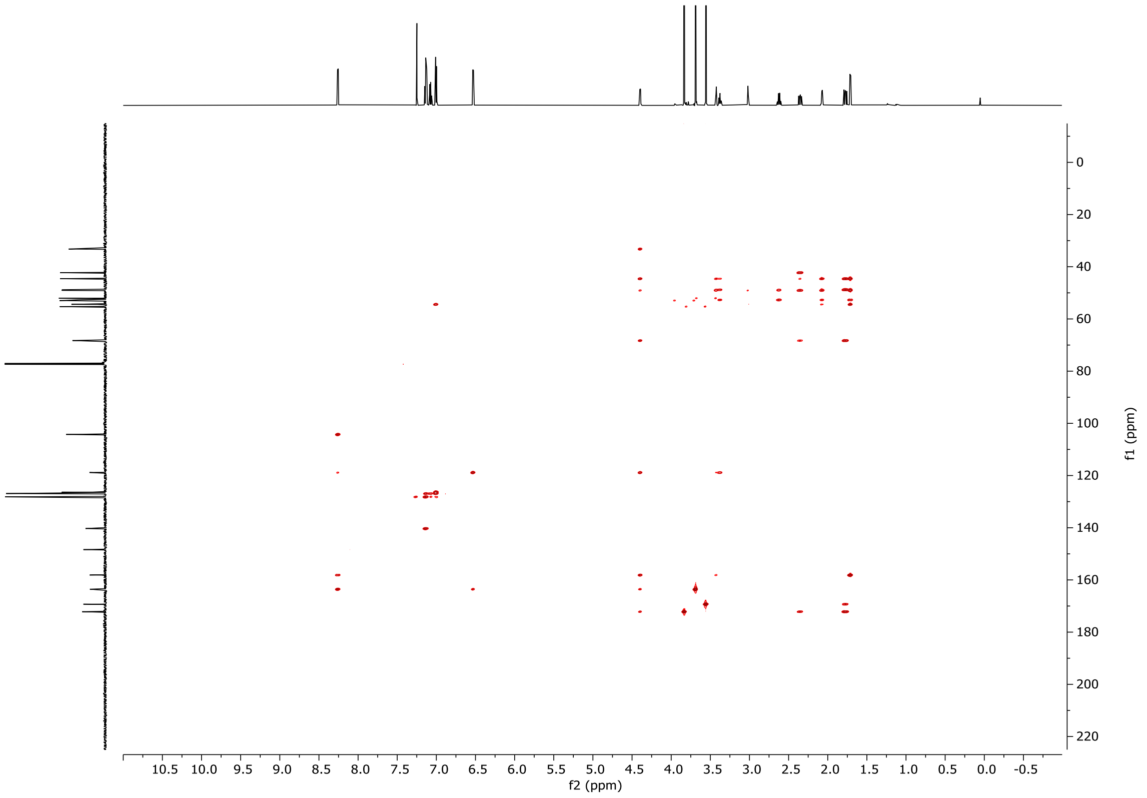

^{1}H NMR (400 MHz, $CDCl_3$) of **5ae**

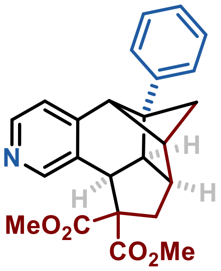

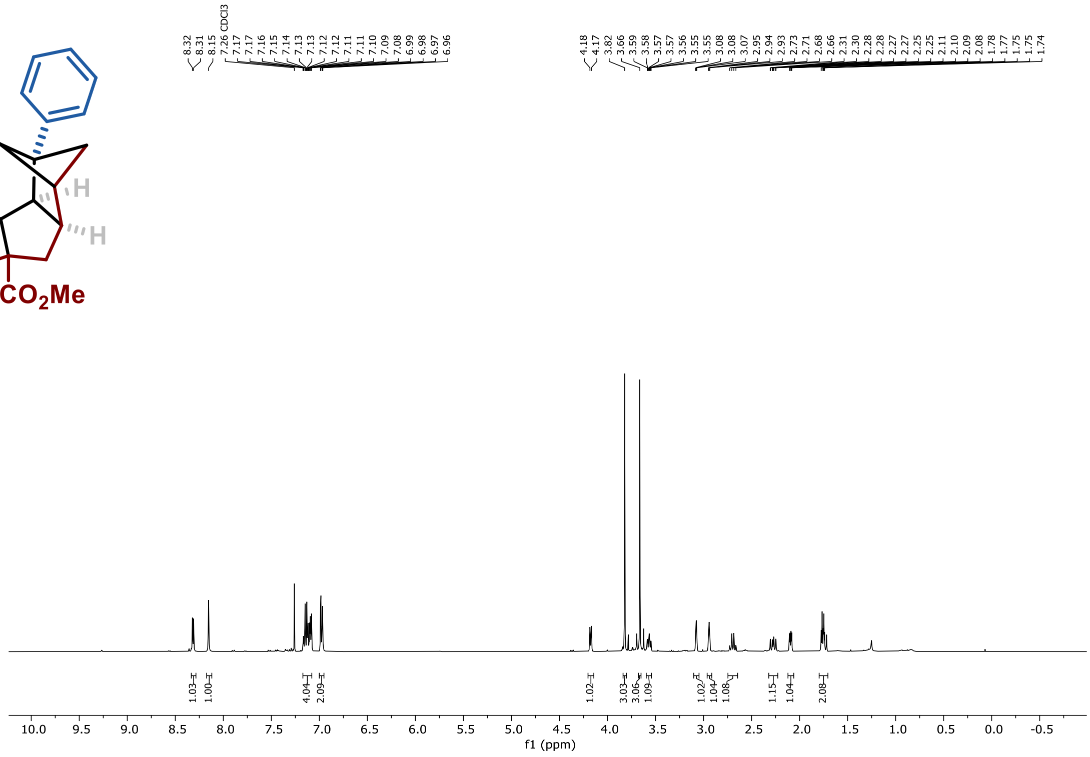

^{13}C NMR (101 MHz, $CDCl_3$) of **5ae**

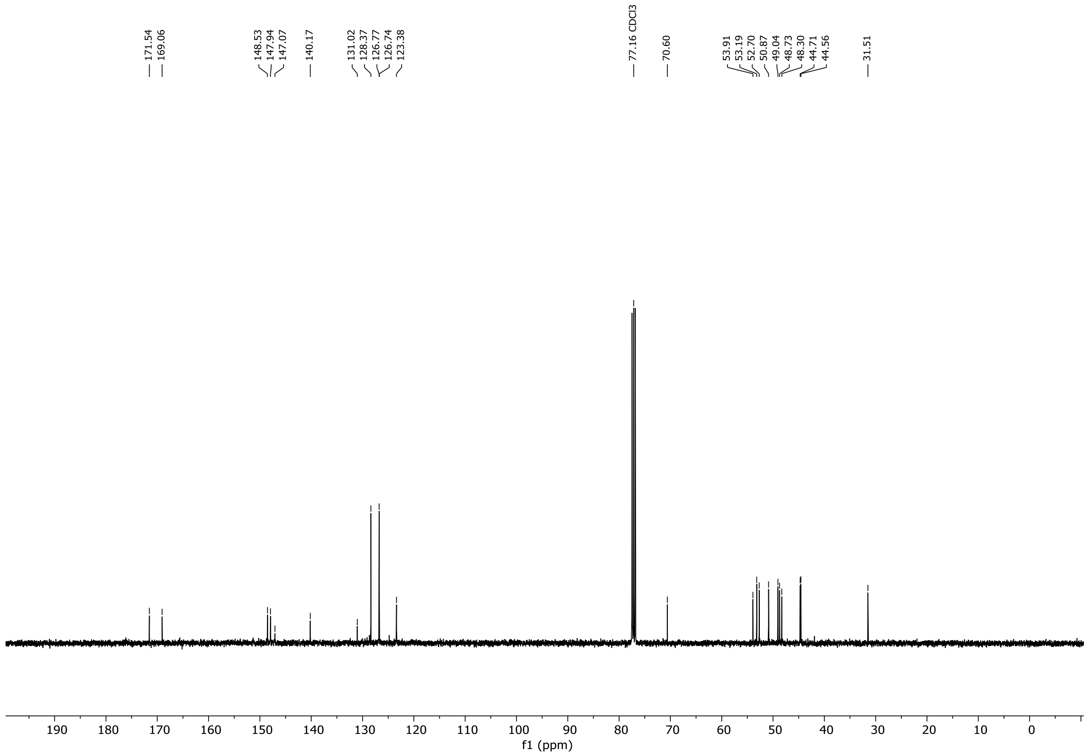

^{1}H NMR (400 MHz, $CDCl_3$) of **5af**

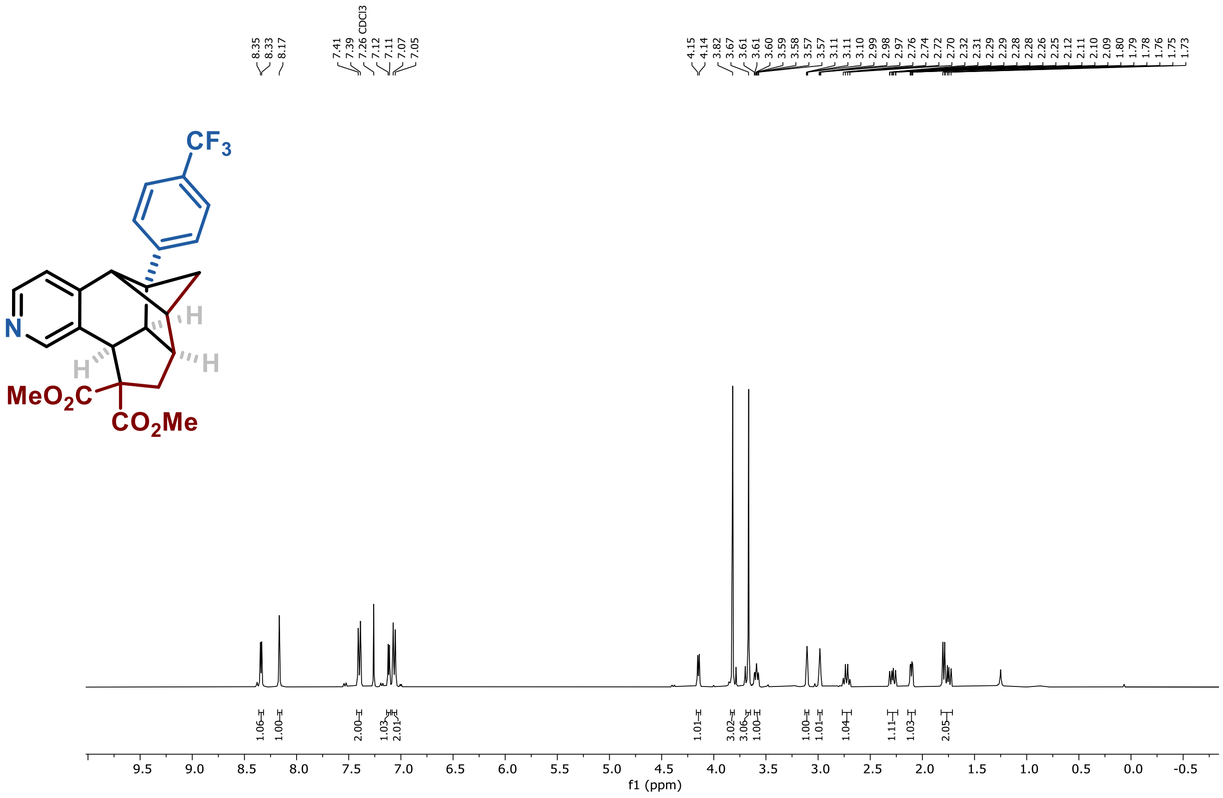

$^{13}C\{^{19}F\}$ NMR (126 MHz, $CDCl_3$) of **5af**

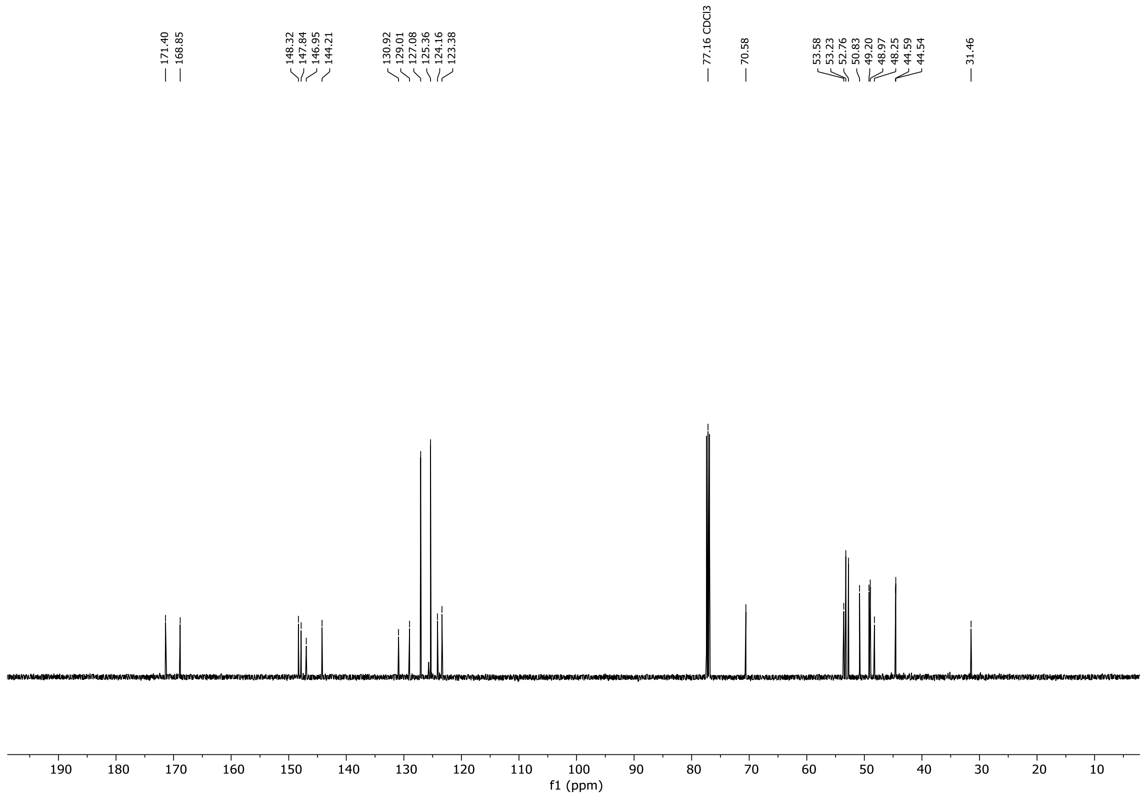

^{19}F NMR (376 MHz, $CDCl_3$) **5af**

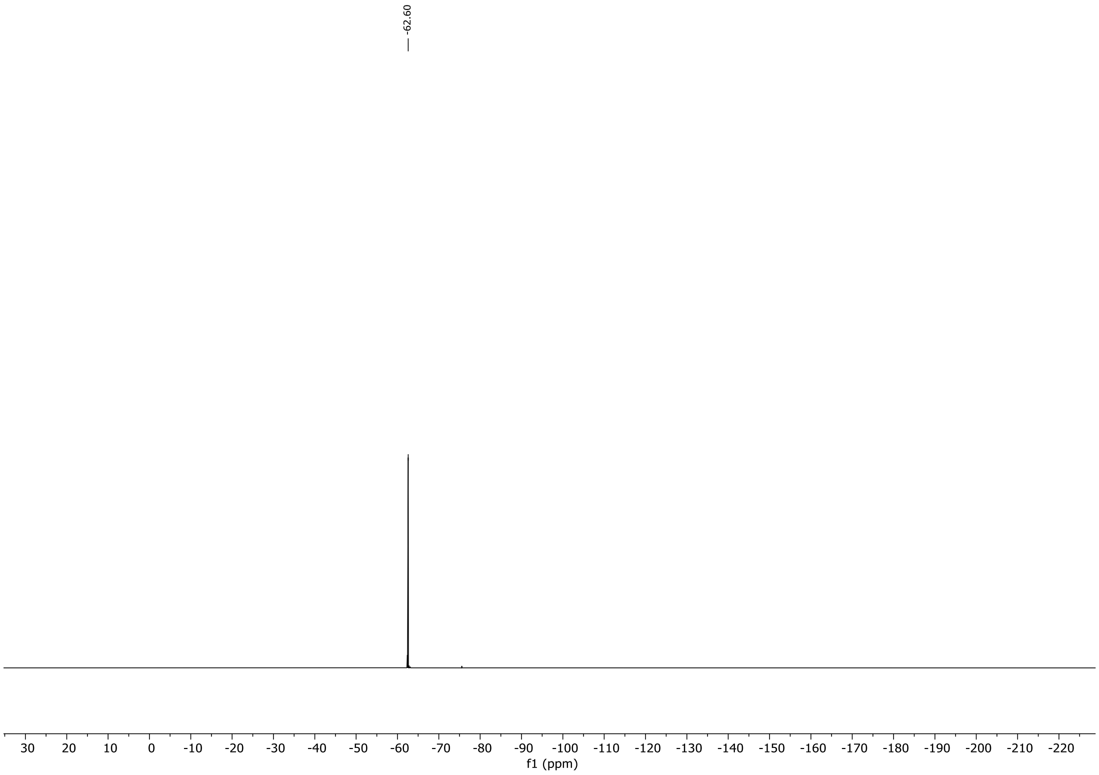

^{1}H NMR (400 MHz, $CDCl_3$) of **5ag**

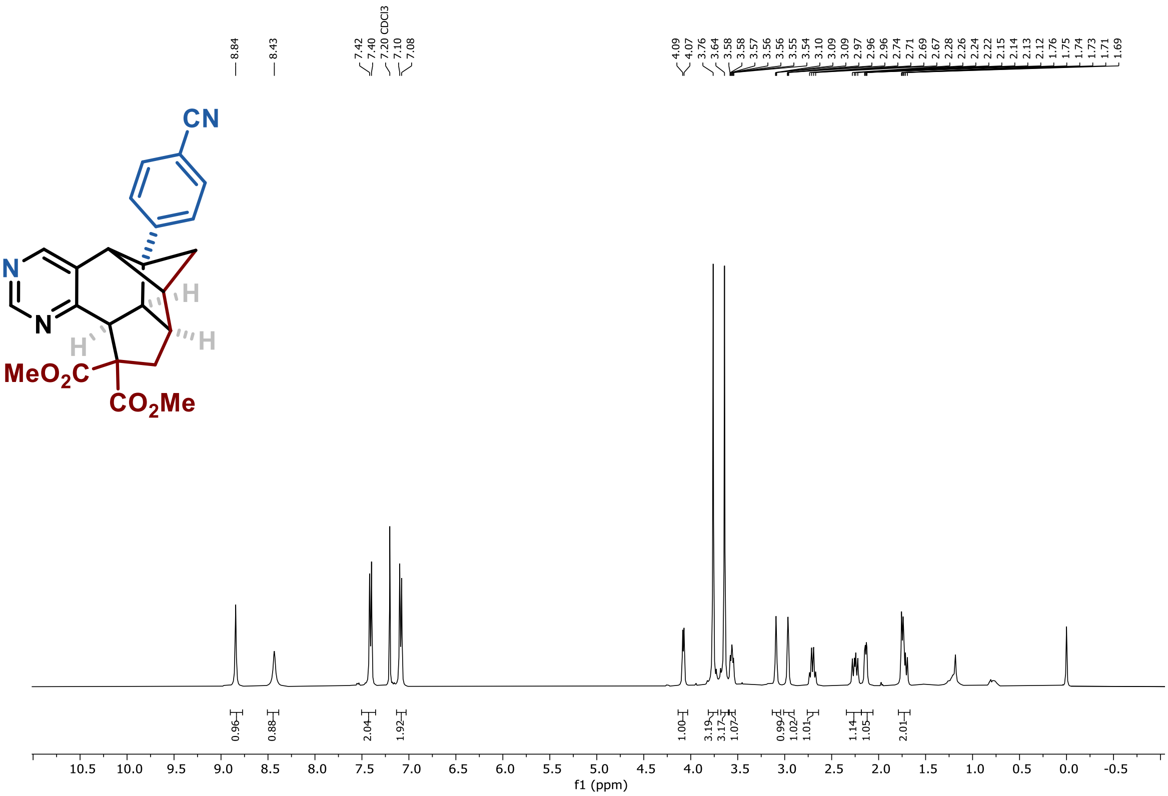

^{13}C NMR (101 MHz, $CDCl_3$) of **5ag**

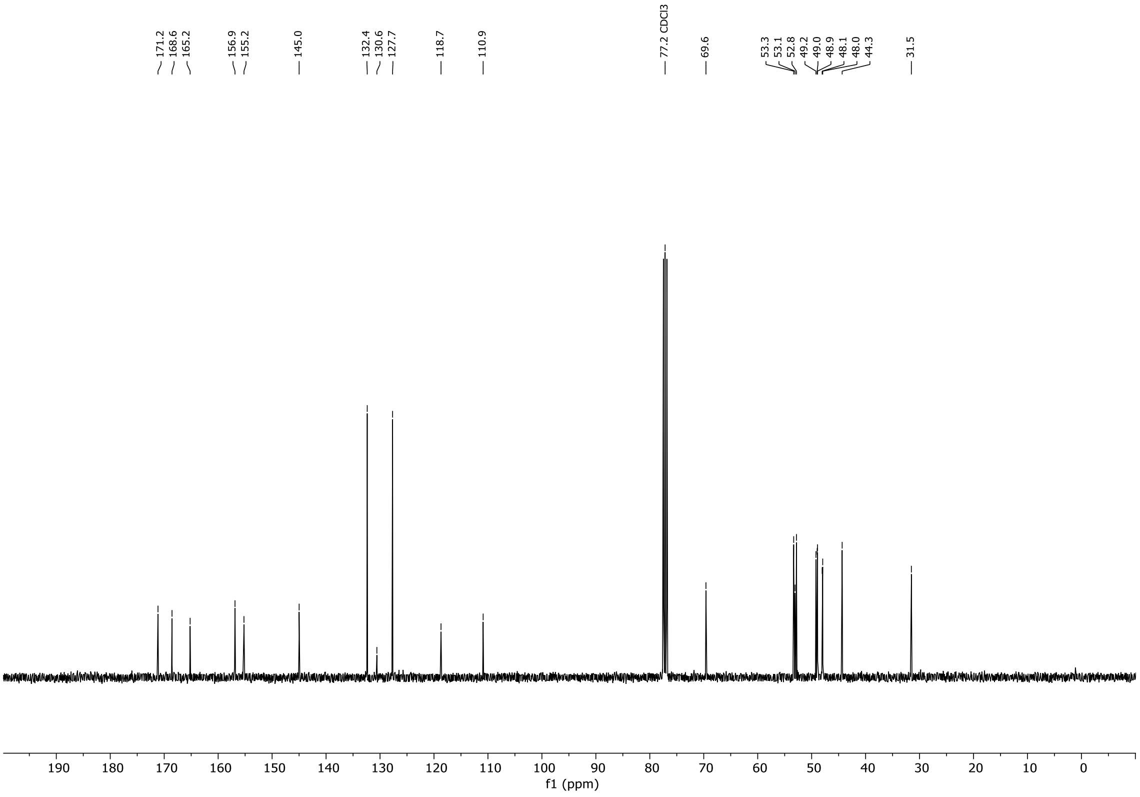

^{1}H NMR (400 MHz, $CDCl_3$) of **5ah**

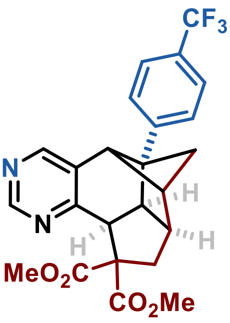

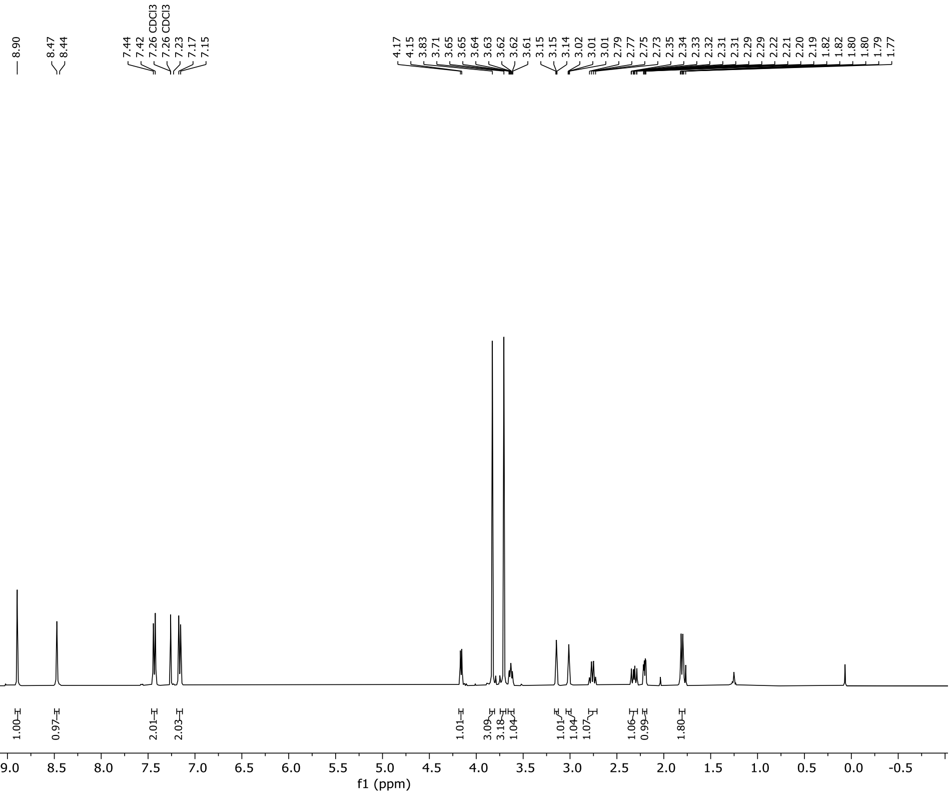

$^{13}C\{^{19}F\}$ NMR (126 MHz, $CDCl_3$) of **5ah**

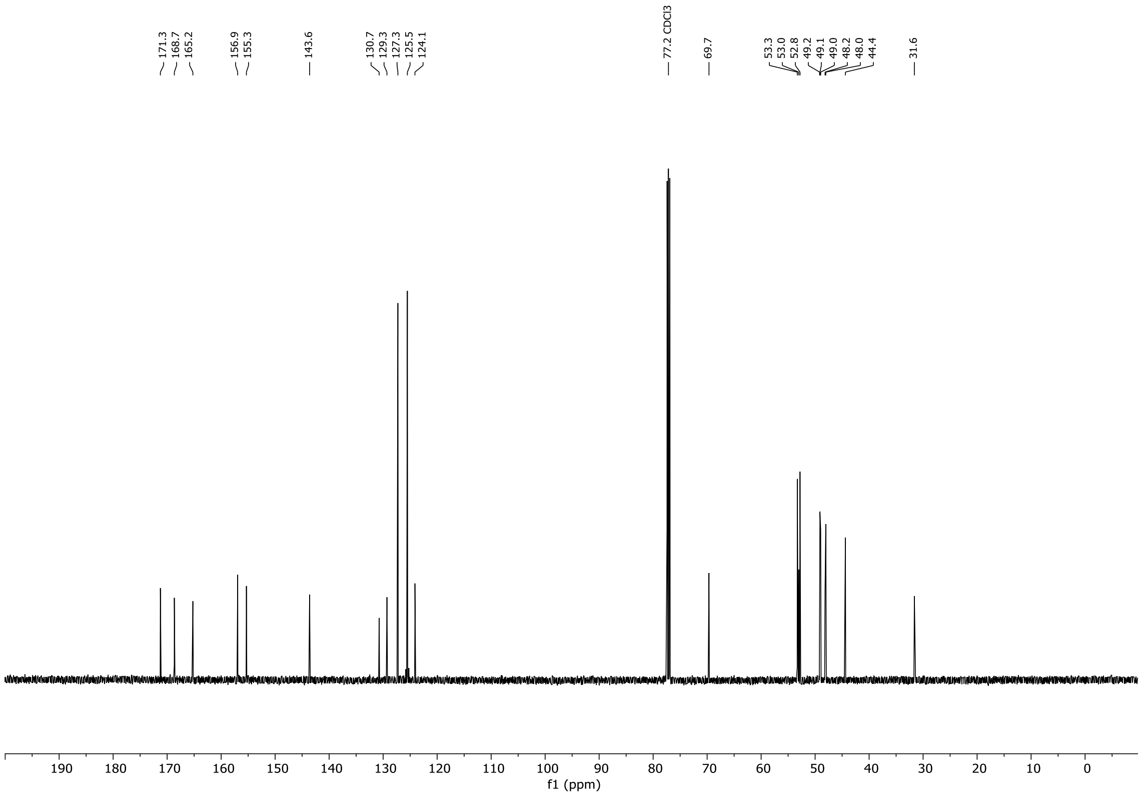

^{19}F NMR (376 MHz, $CDCl_3$) **5ah**

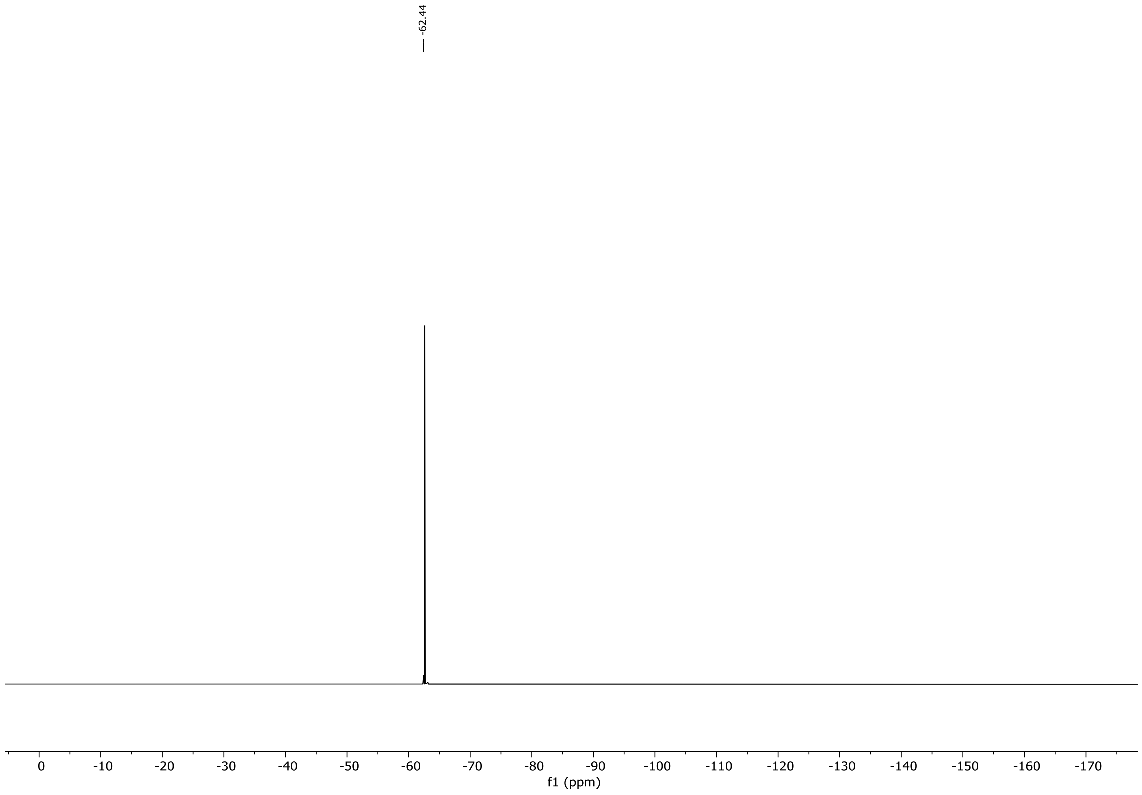

^{1}H NMR (400 MHz, $CDCl_3$) of **5ai**

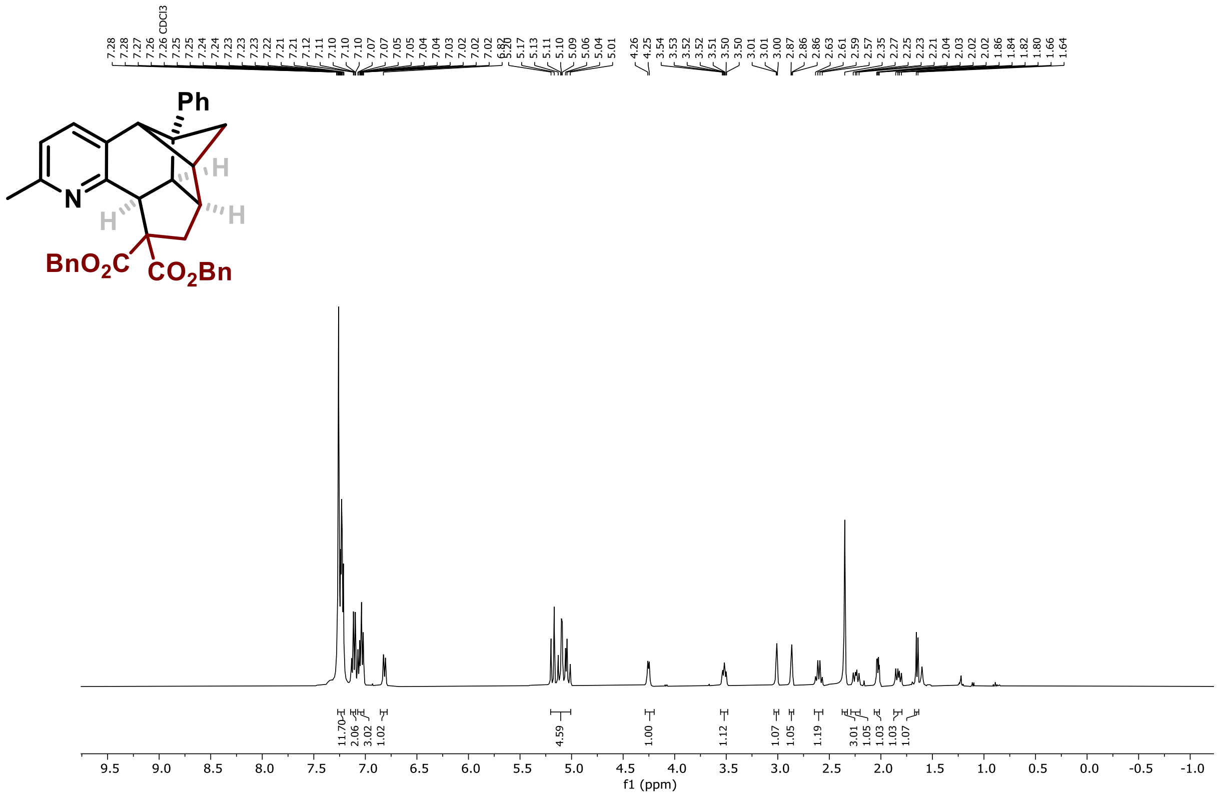

^{13}C NMR (101 MHz, $CDCl_3$) of **5ai**

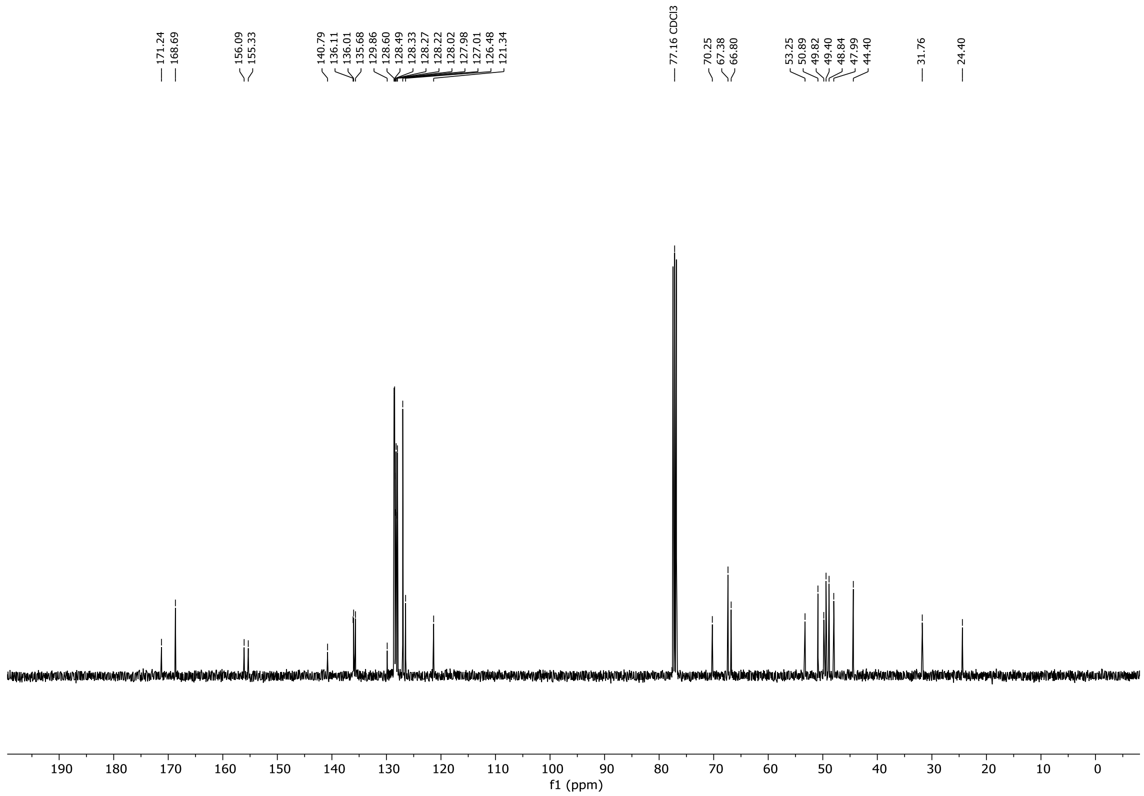

^{1}H NMR (400 MHz, $CDCl_3$) of **5aj**

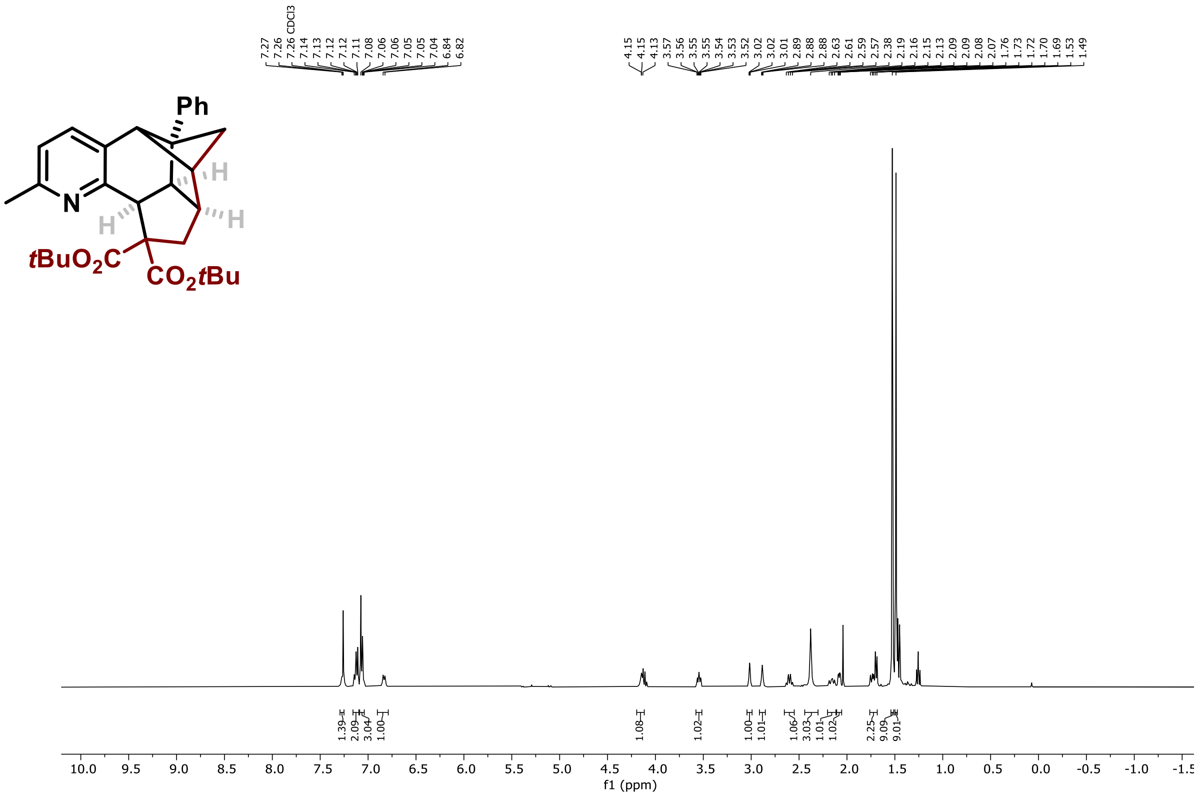

^{13}C NMR (101 MHz, $CDCl_3$) of **5aj**

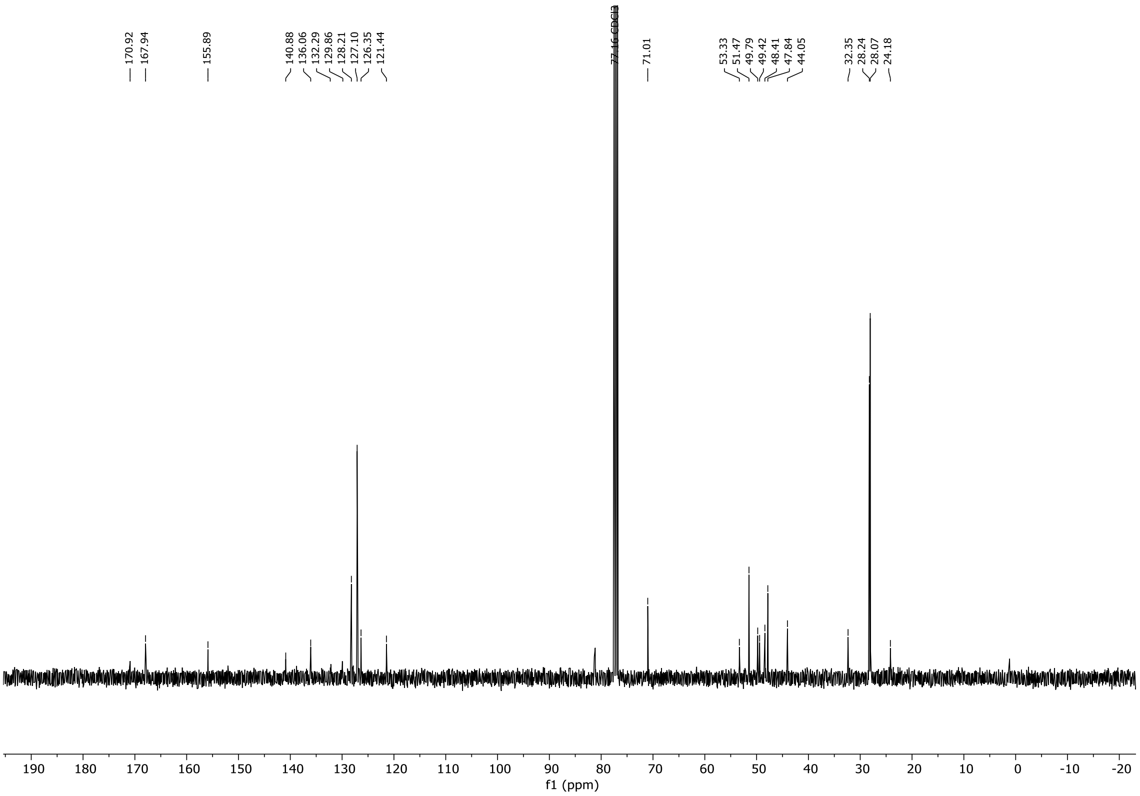

^{1}H NMR (400 MHz, $CDCl_3$) of **5ak**

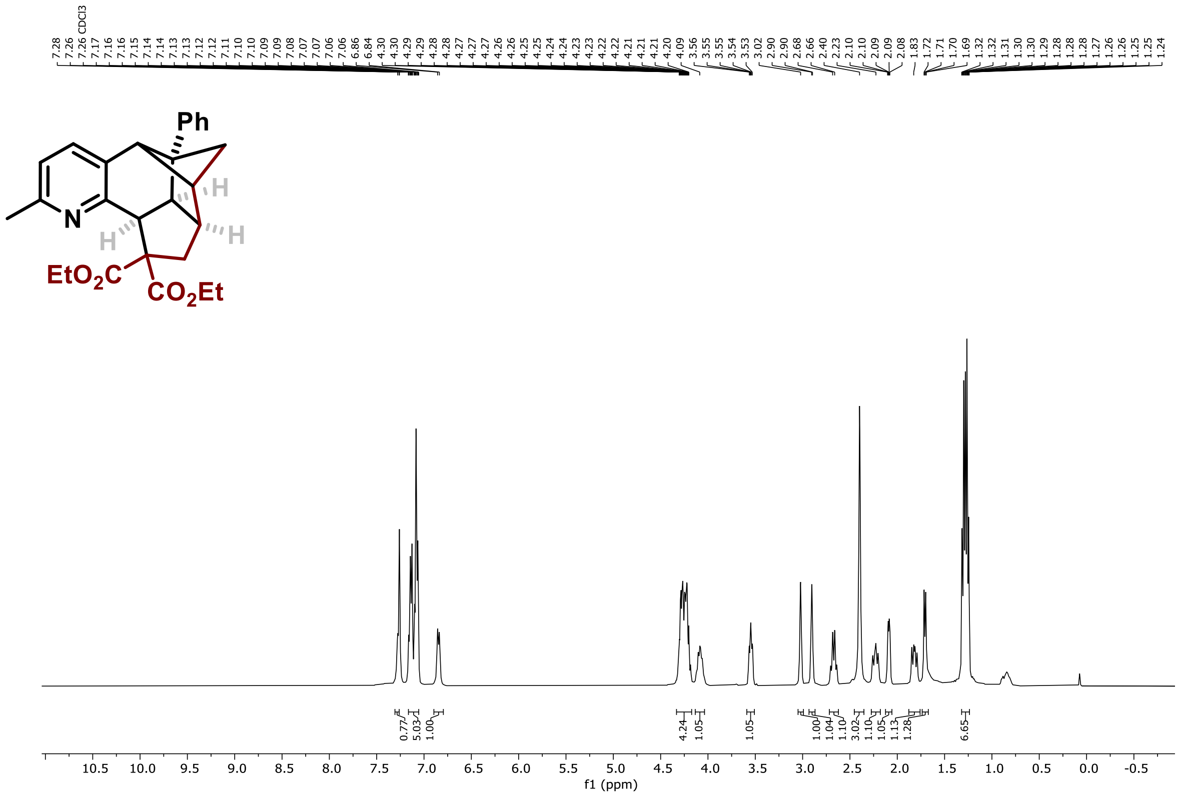

^{13}C NMR (101 MHz, $CDCl_3$) of **5ak**

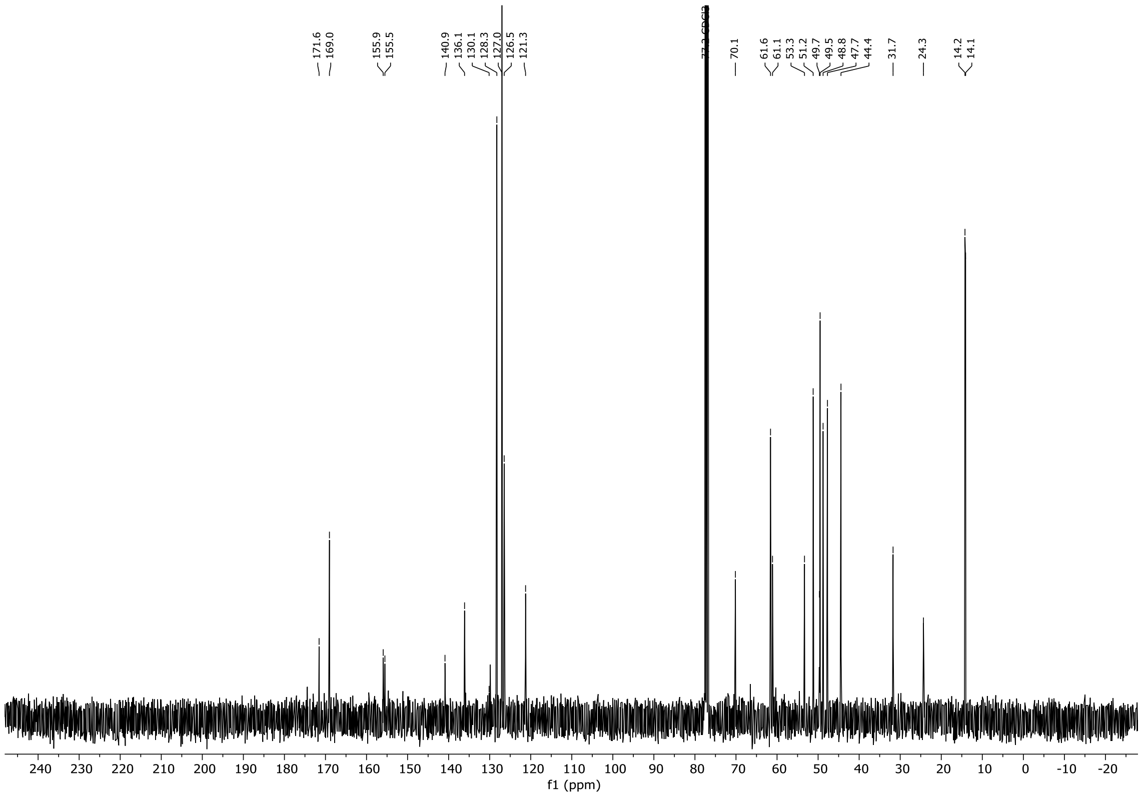

^{1}H NMR (400 MHz, $CDCl_3$) of **5al**

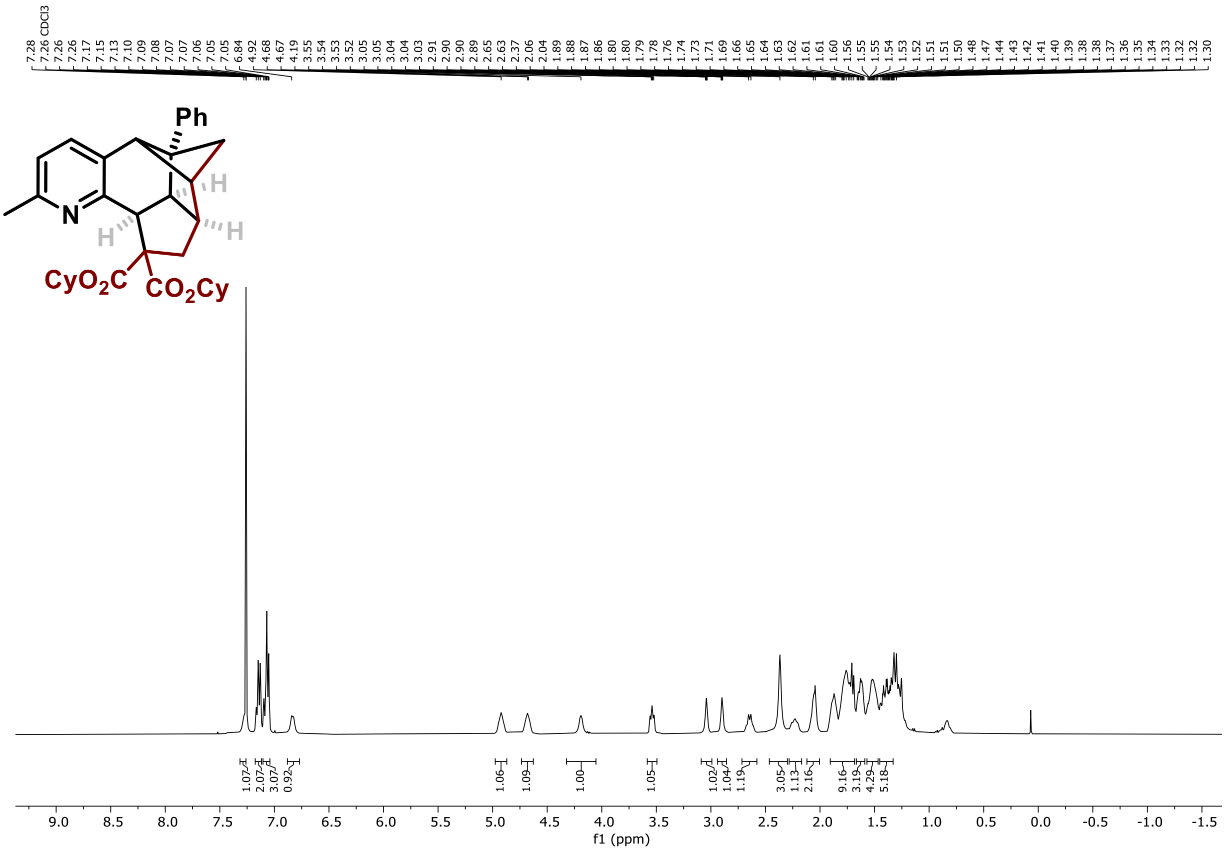

^{13}C NMR (101 MHz, $CDCl_3$) of **5al**

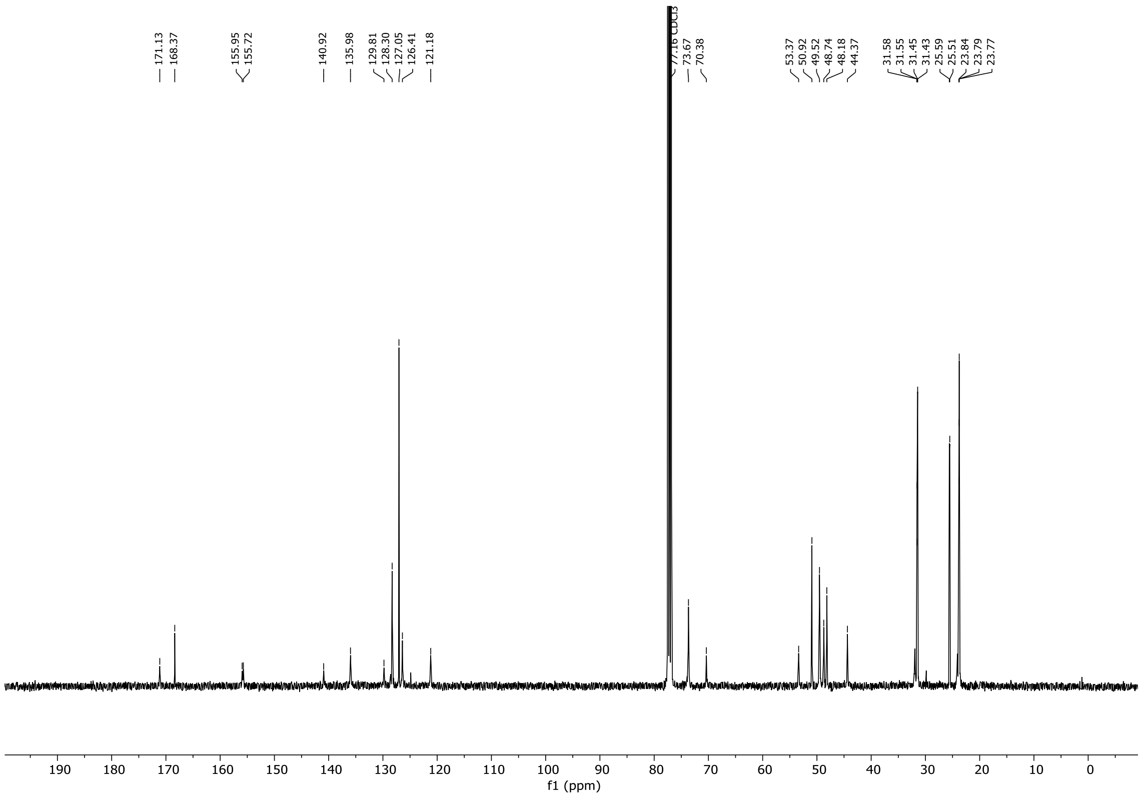

^{1}H NMR (400 MHz, $CDCl_3$) of **5am**

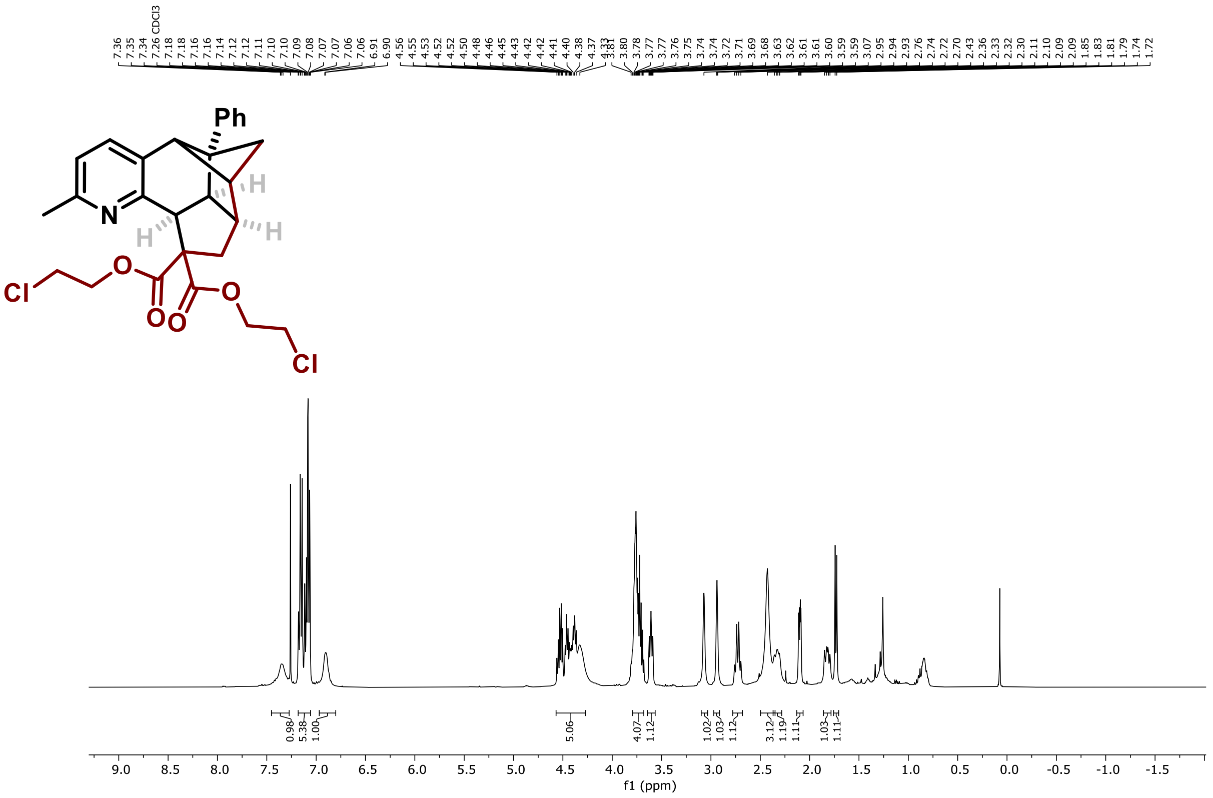

^{13}C NMR (101 MHz, $CDCl_3$) of **5am**

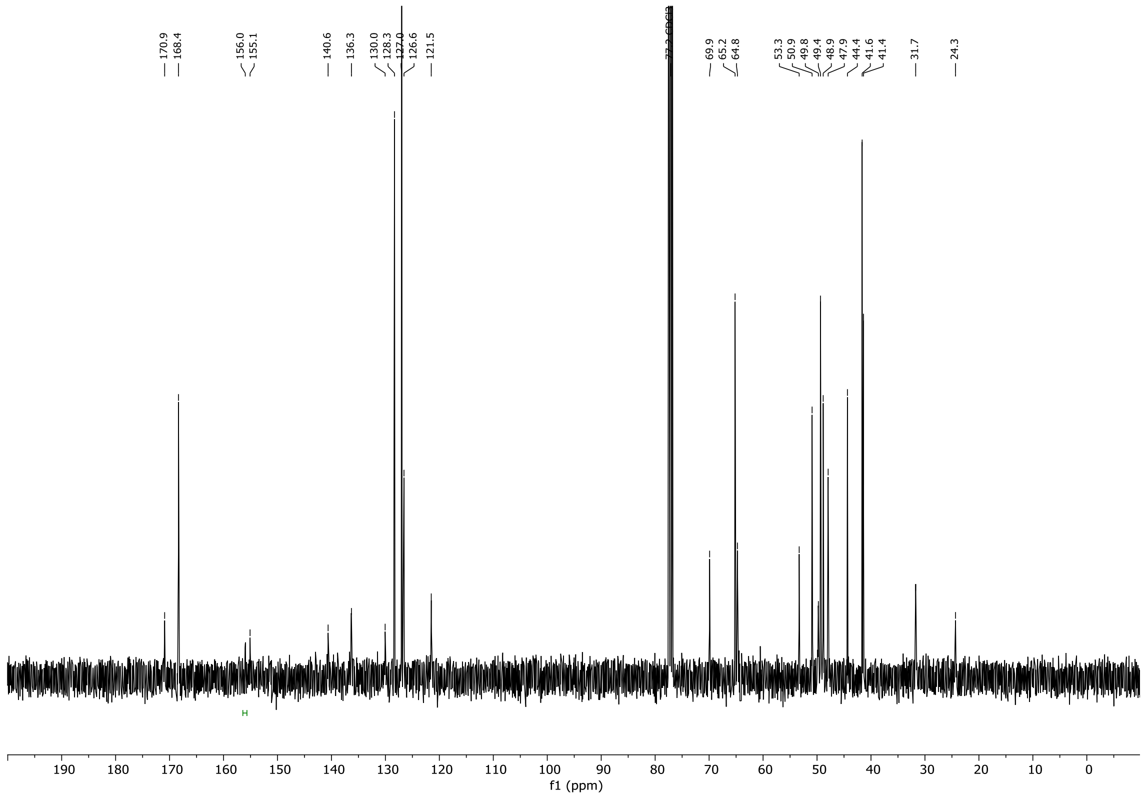

^{1}H NMR (400 MHz, $CDCl_3$) of **5an**

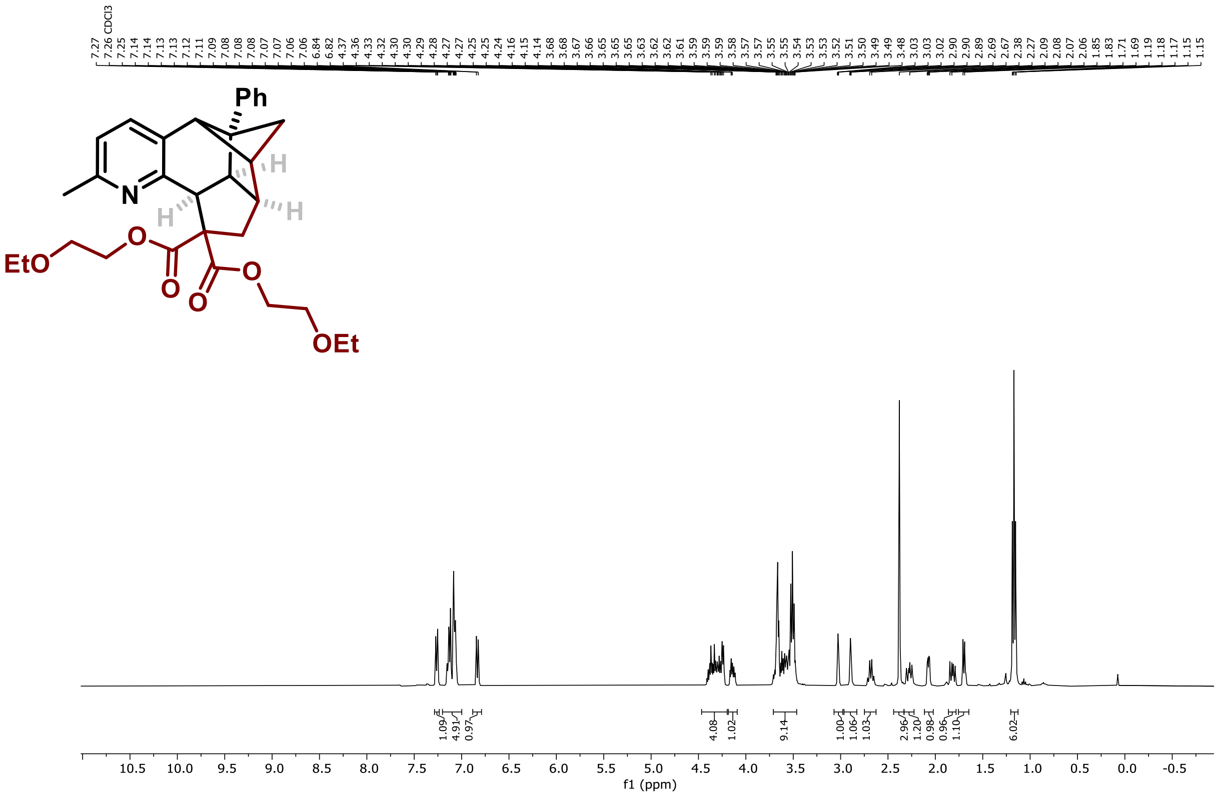

^{13}C NMR (101 MHz, $CDCl_3$) of **5an**

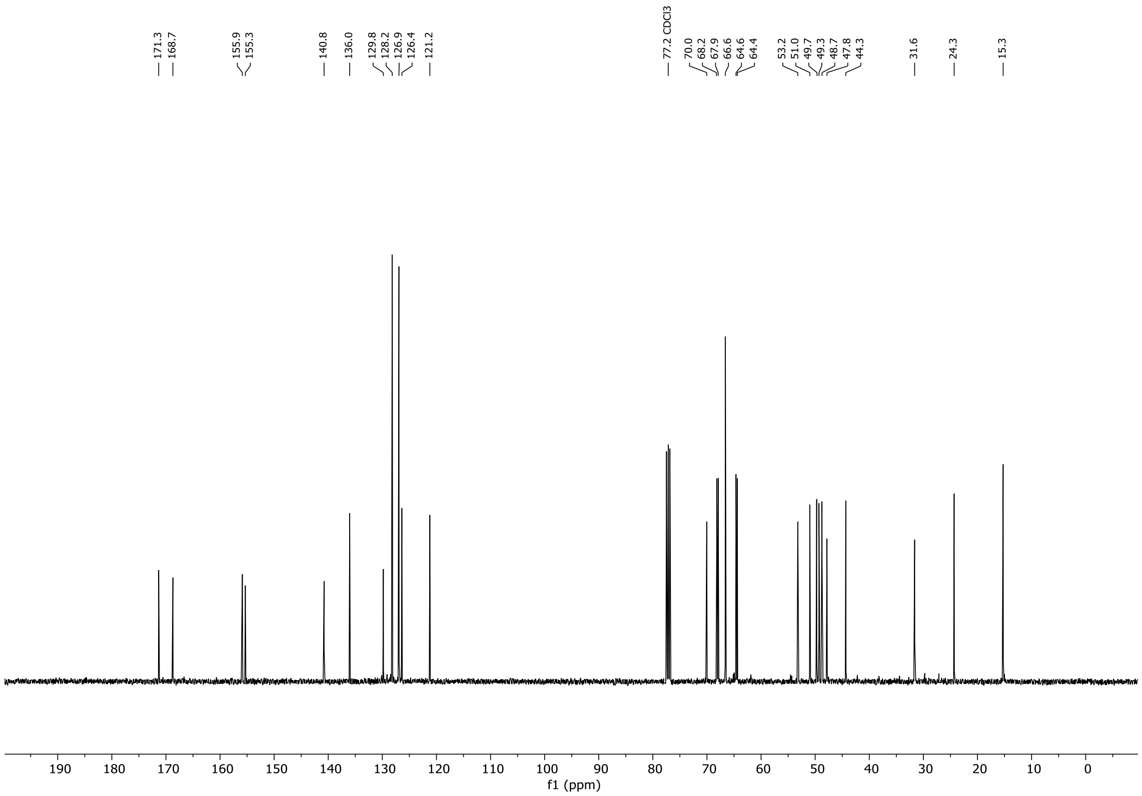

^{1}H NMR (400 MHz, $CDCl_3$) of **5ao** (Major diastereoisomer)

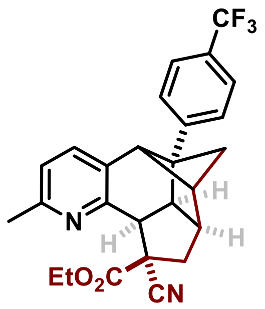

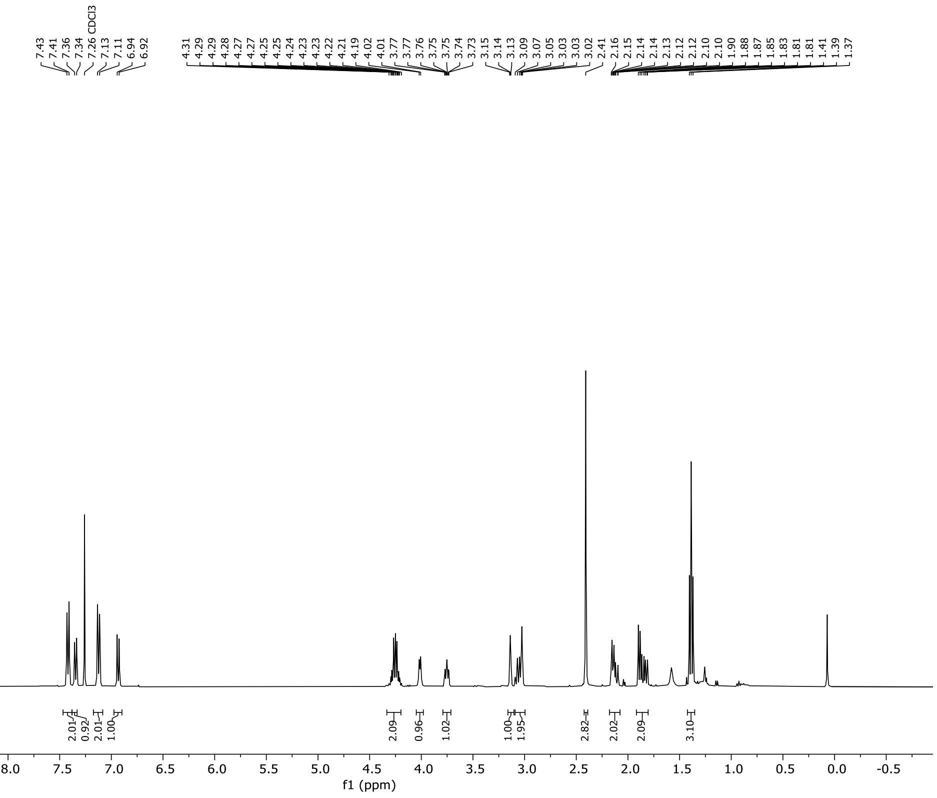

^{13}C NMR (101 MHz, $CDCl_3$) **5ao** (Major diastereoisomer)

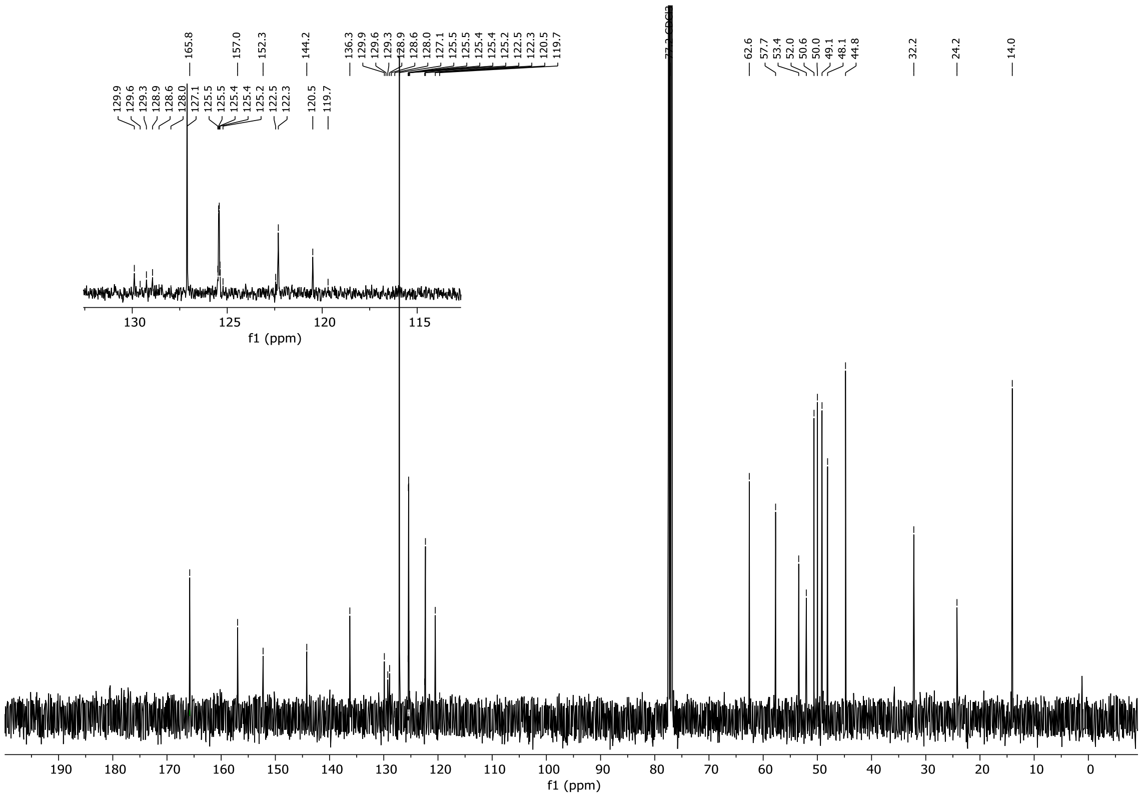

^{19}F NMR (376 MHz, $CDCl_3$) **5ao** (Major diastereoisomer)

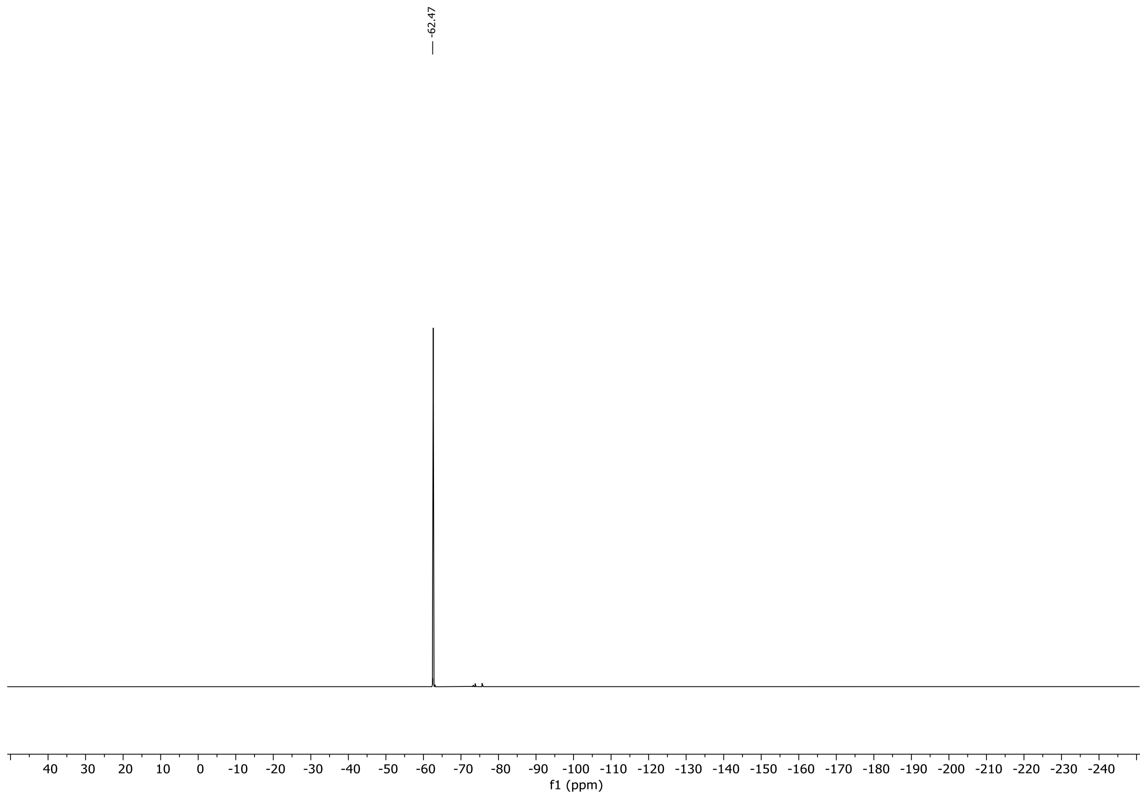

^{1}H NMR (400 MHz, $CDCl_3$) of **5ao** (Minor diastereoisomer)

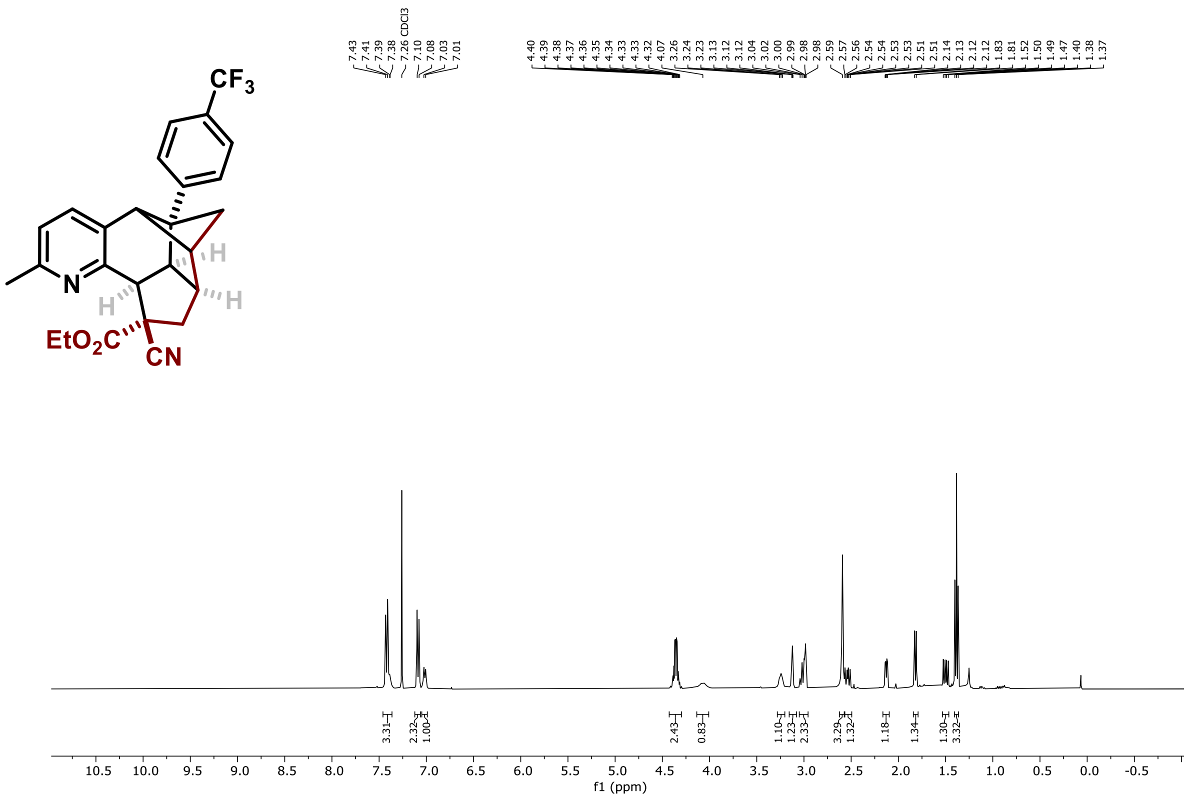

^{13}C NMR (101 MHz, $CDCl_3$) **5ao** (Minor diastereoisomer)

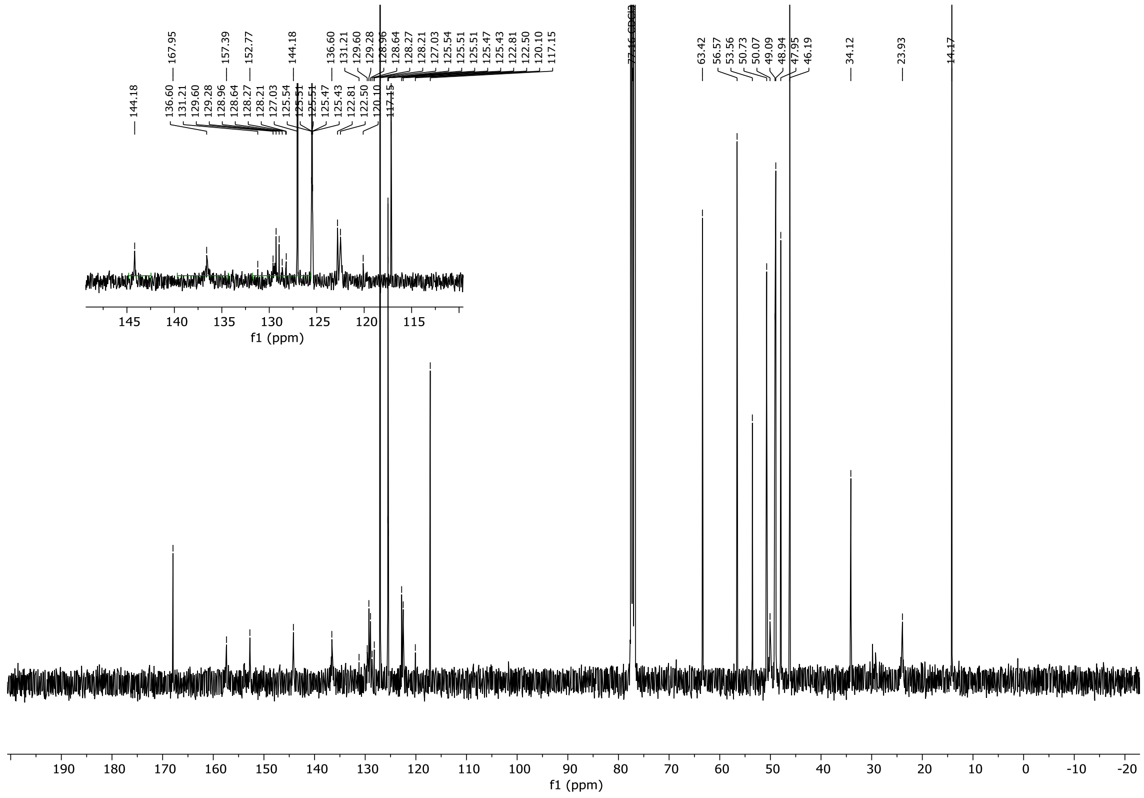

^{19}F NMR (376 MHz, $CDCl_3$) **5ao** (Minor diastereoisomer)

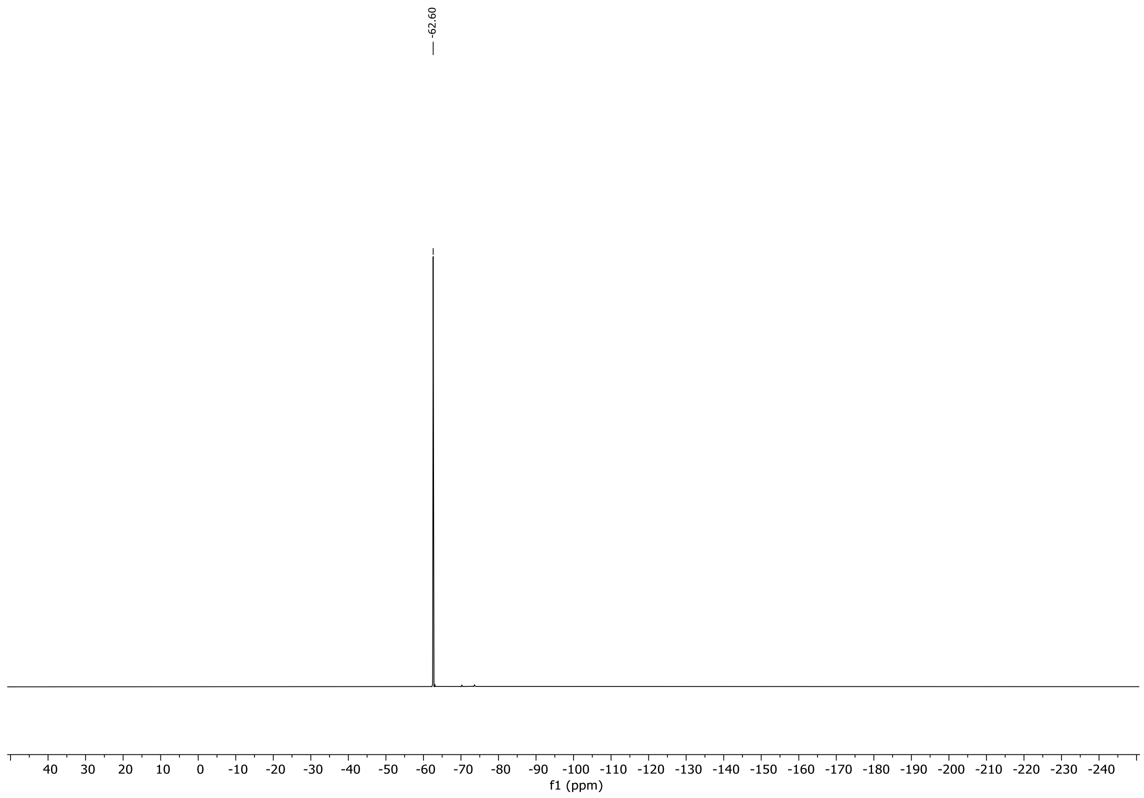

^{1}H NMR (400 MHz, $CDCl_3$) of **5ap**

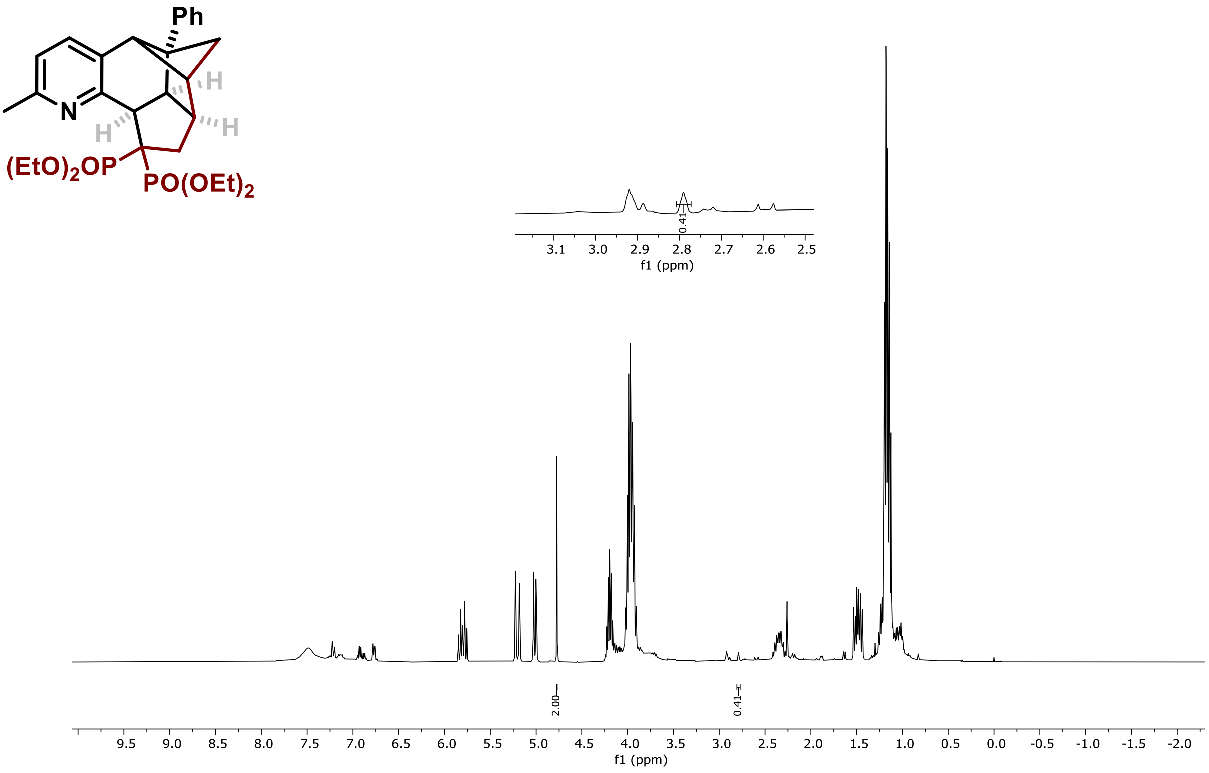

^{1}H NMR (599 MHz, $CDCl_3$) of **5aq**

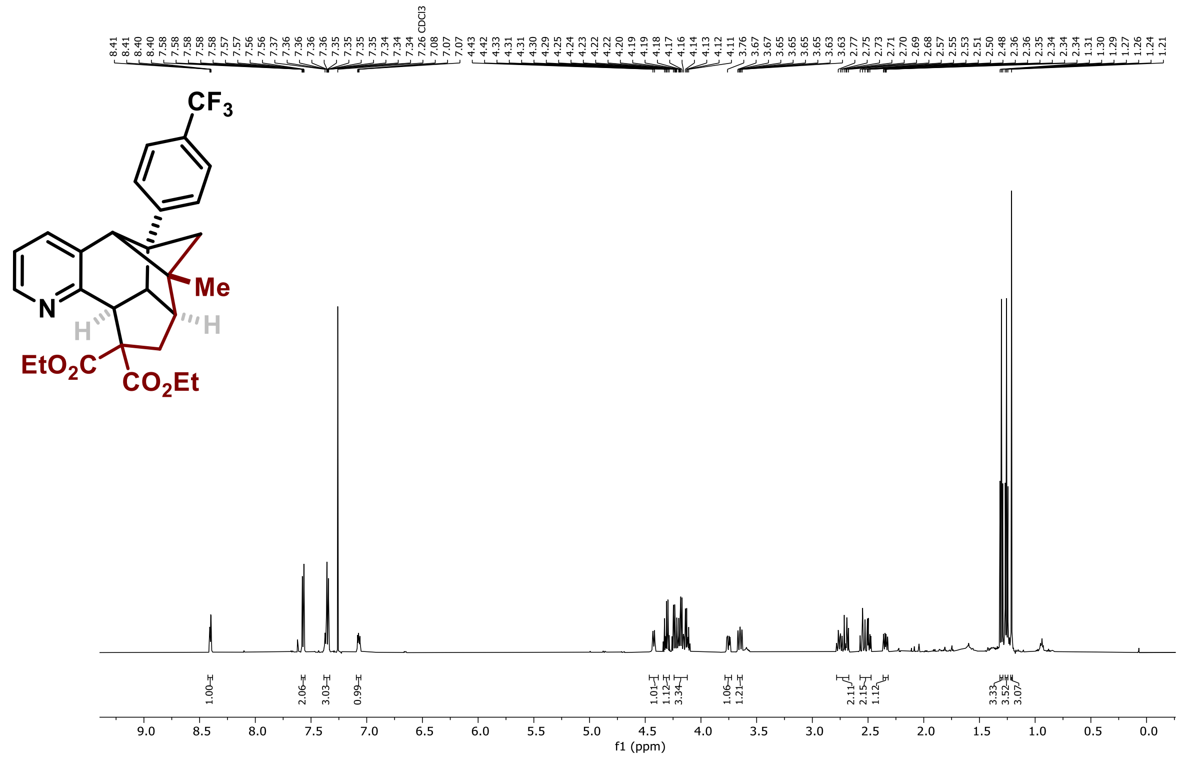

$^{13}C\{^{19}F\}$ NMR (151 MHz, $CDCl_3$) of **5aq**

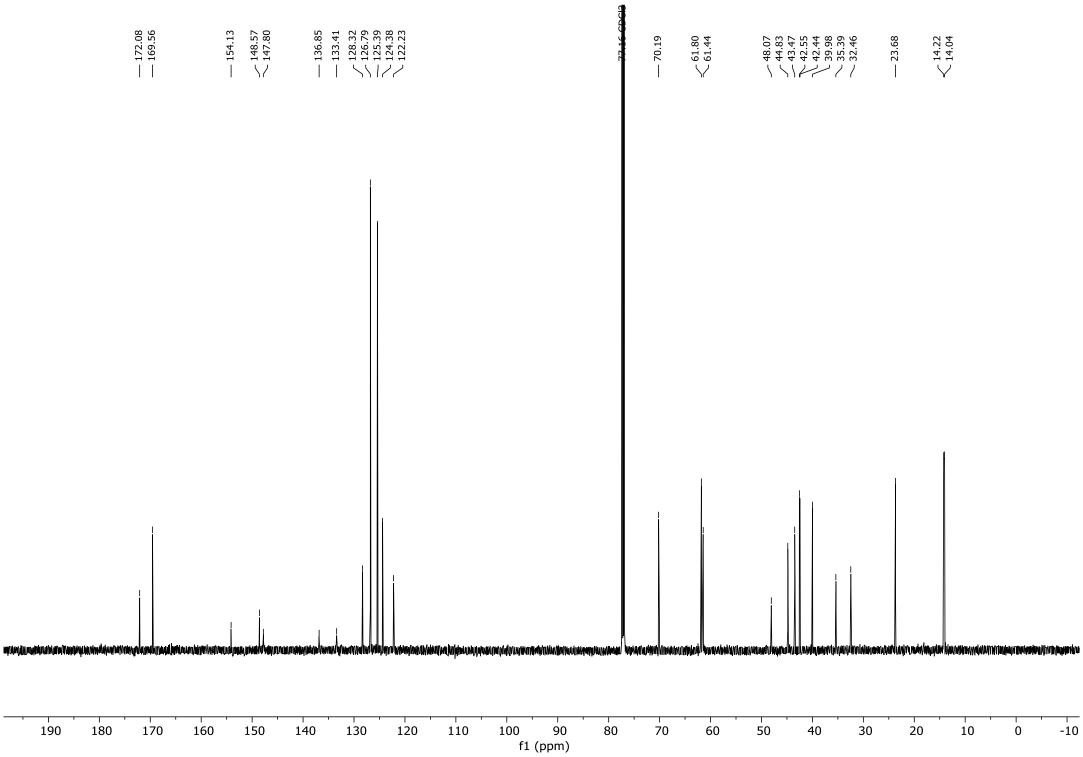

^{19}F NMR (563 MHz, $CDCl_3$) **5aq**

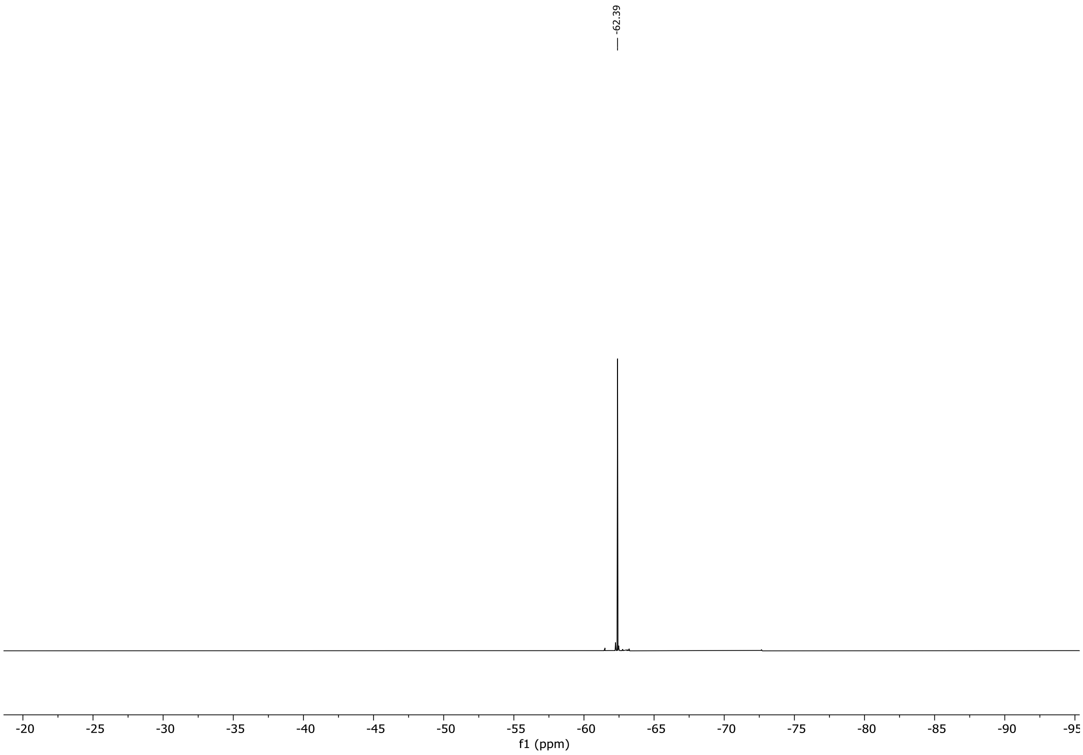

^{1}H NMR (400 MHz, $CDCl_3$) of **5ar**

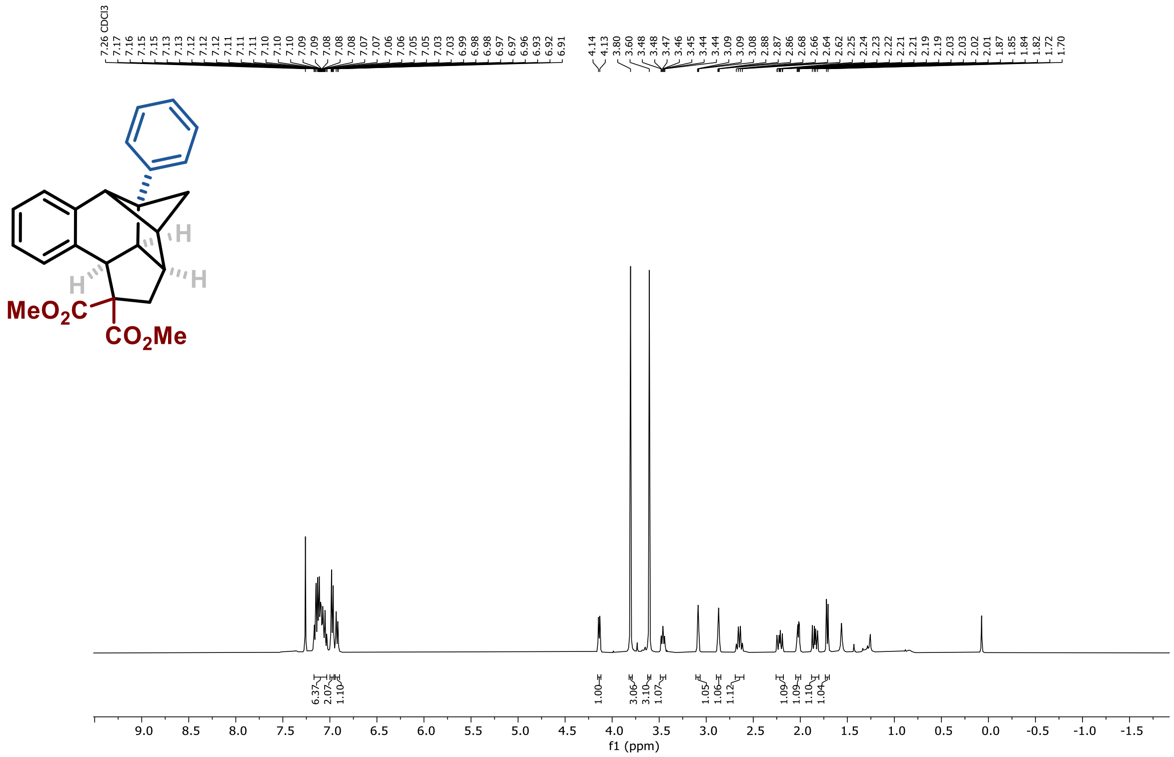

^{13}C NMR (101 MHz, $CDCl_3$) of **5ar**

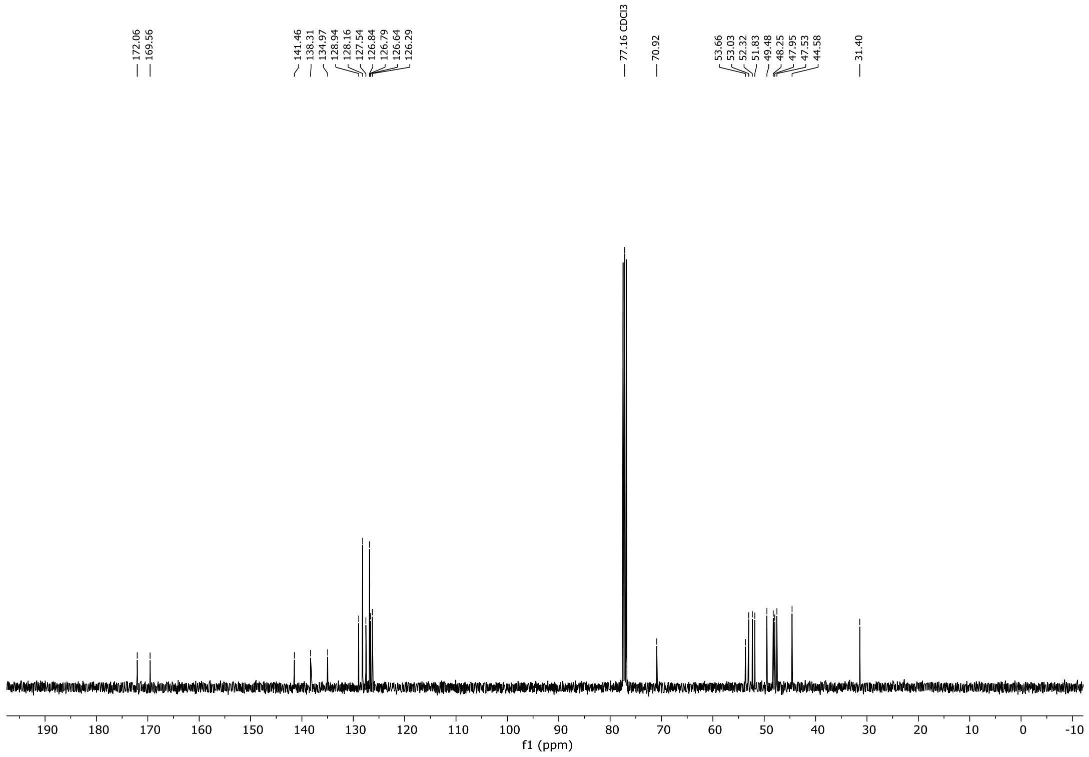

^{1}H NMR (400 MHz, $CDCl_3$) of **6a**

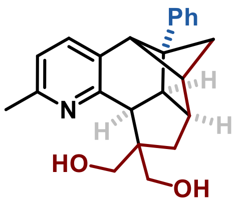

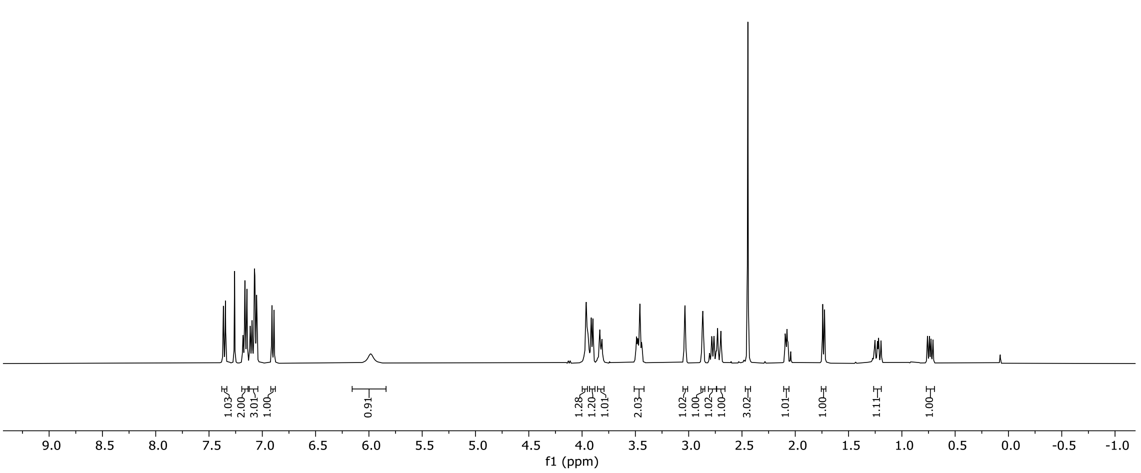

^{13}C NMR (151 MHz, $CDCl_3$) of **6a**

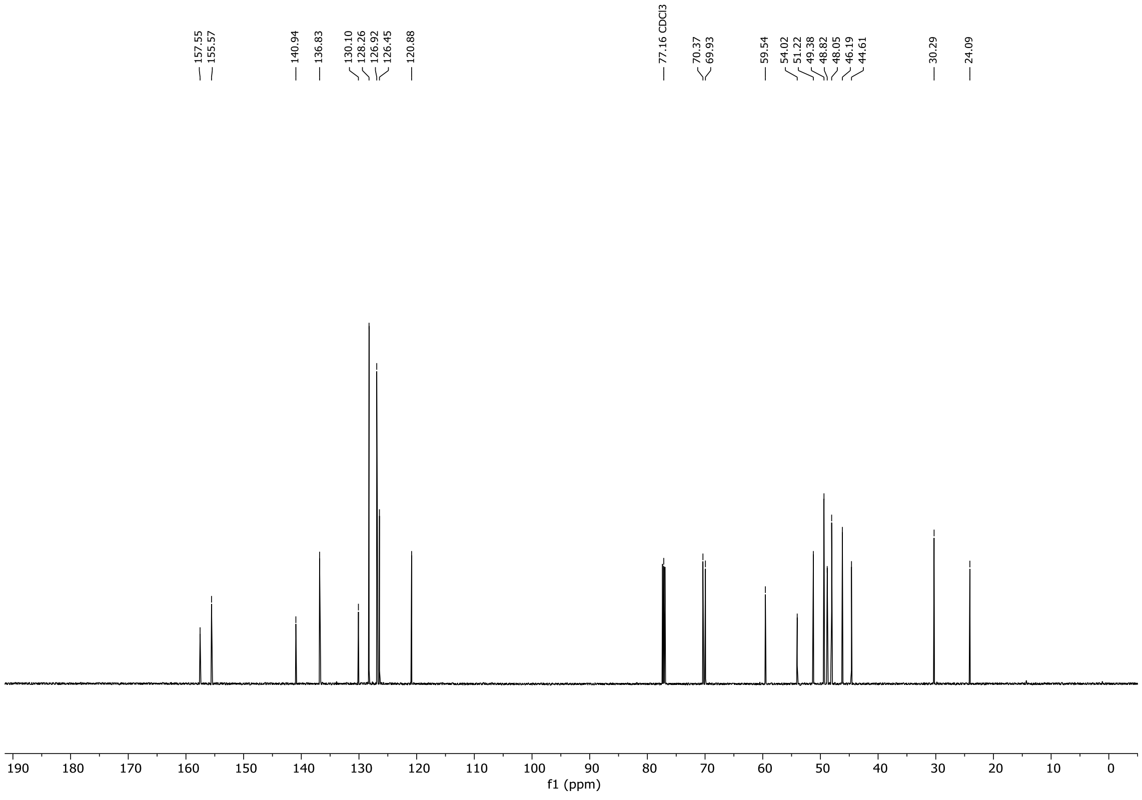

^{1}H NMR (400 MHz, DMSO-d$_6$) of **6b**

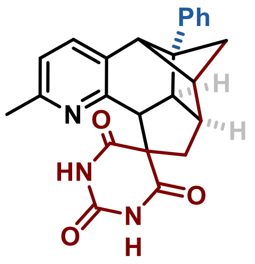

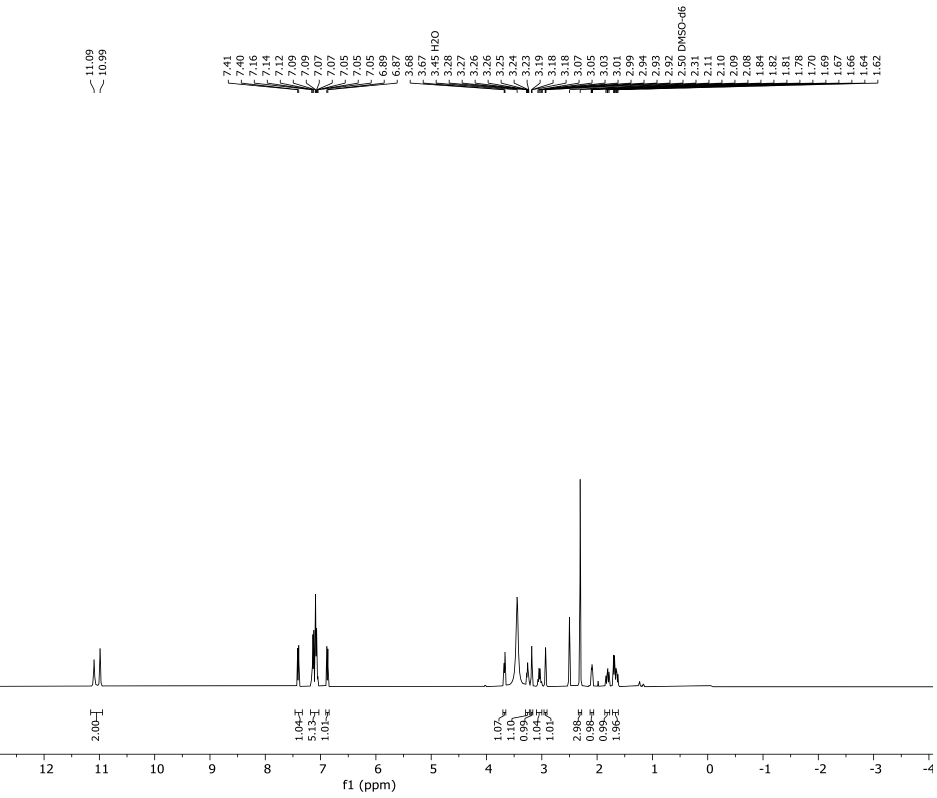

^{13}C NMR (101 MHz, DMSO-d$_6$) of **6b**

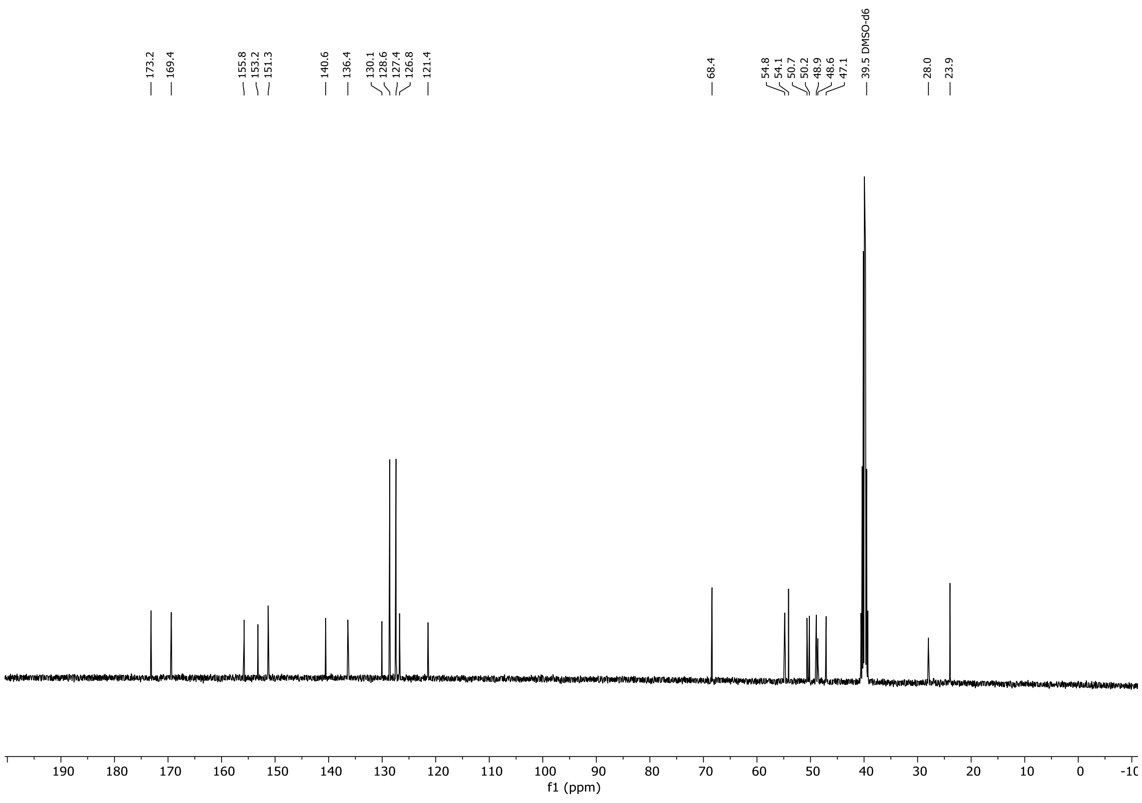

^{1}H NMR (400 MHz, $CDCl_3$) of **6c**

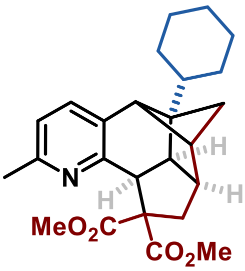

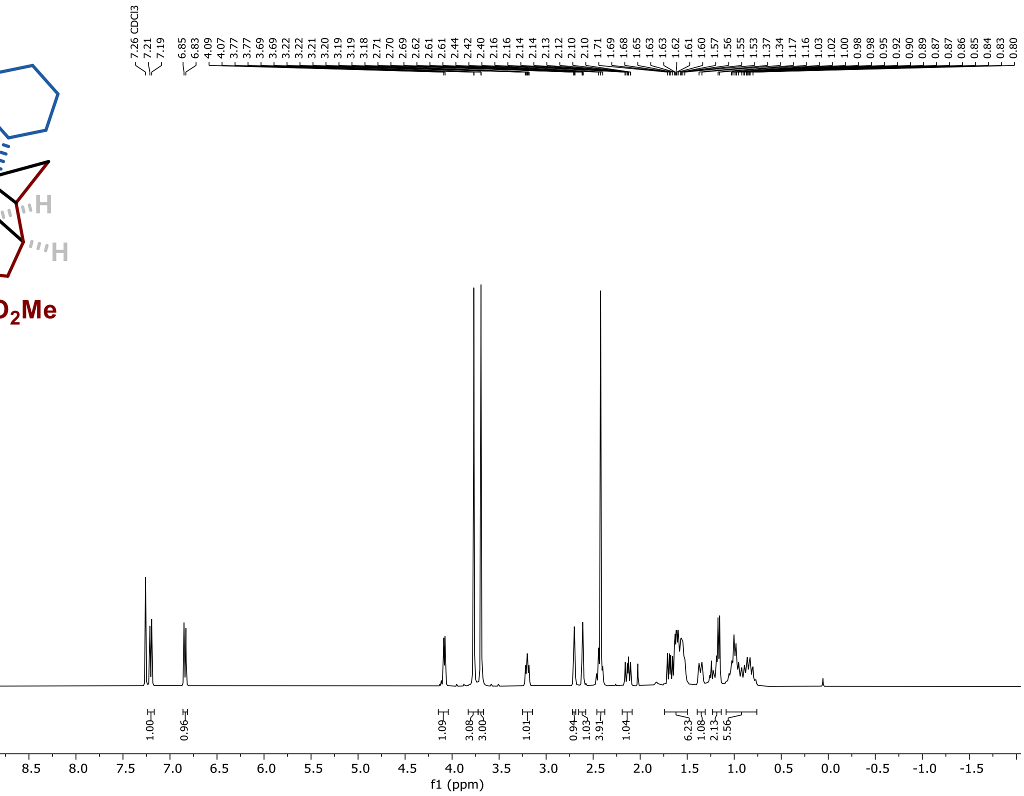

^{13}C NMR (101 MHz, $CDCl_3$) of **6c**

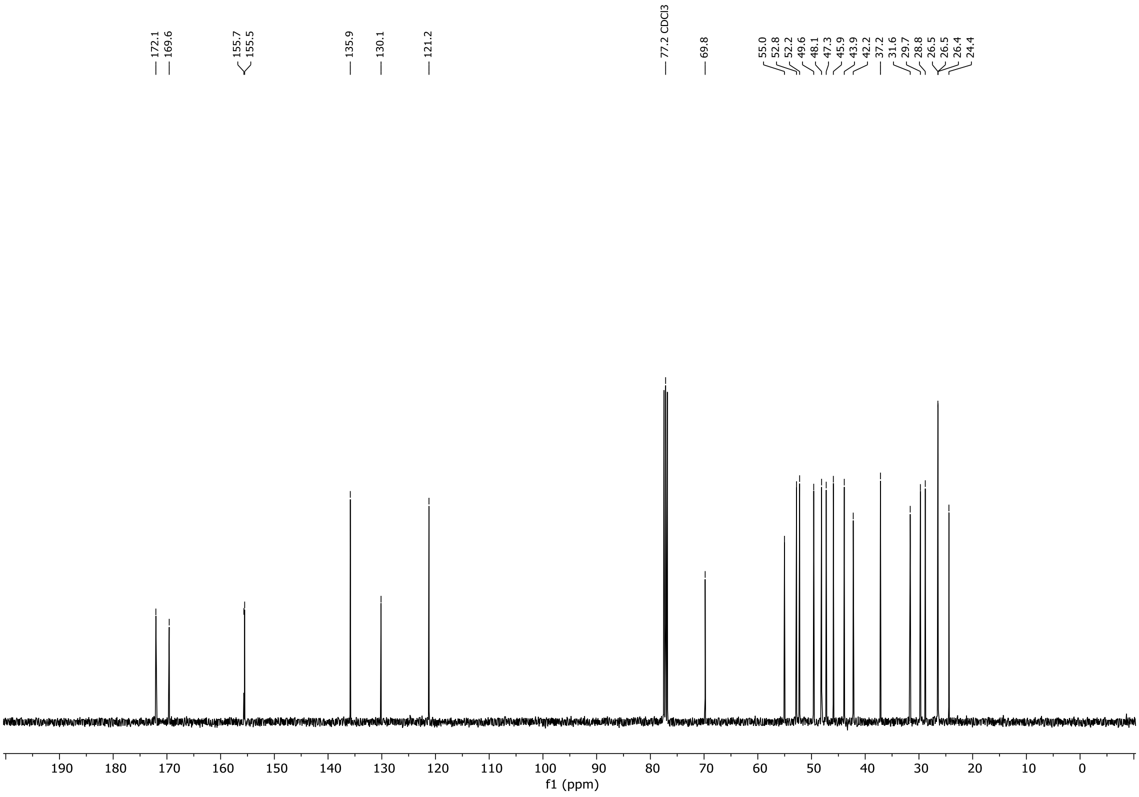

^{1}H NMR (400 MHz, $CDCl_3$) of **6d**

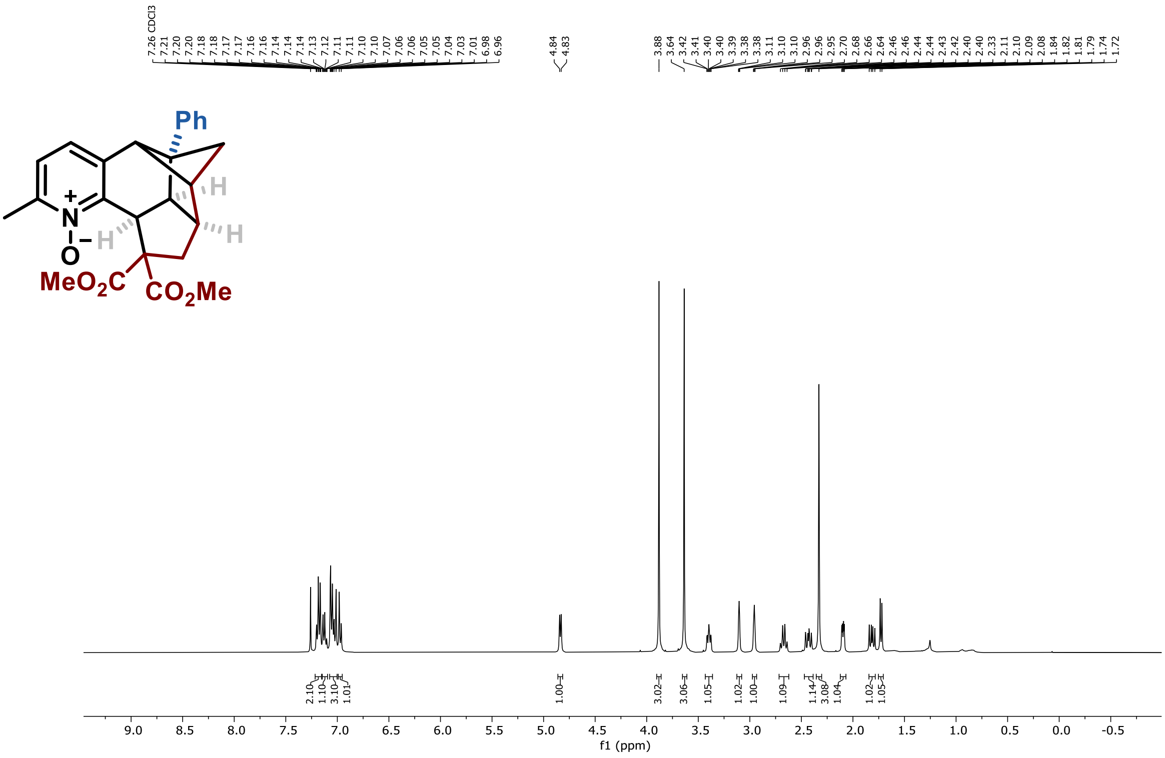

^{13}C NMR (101 MHz, $CDCl_3$) of **6d**

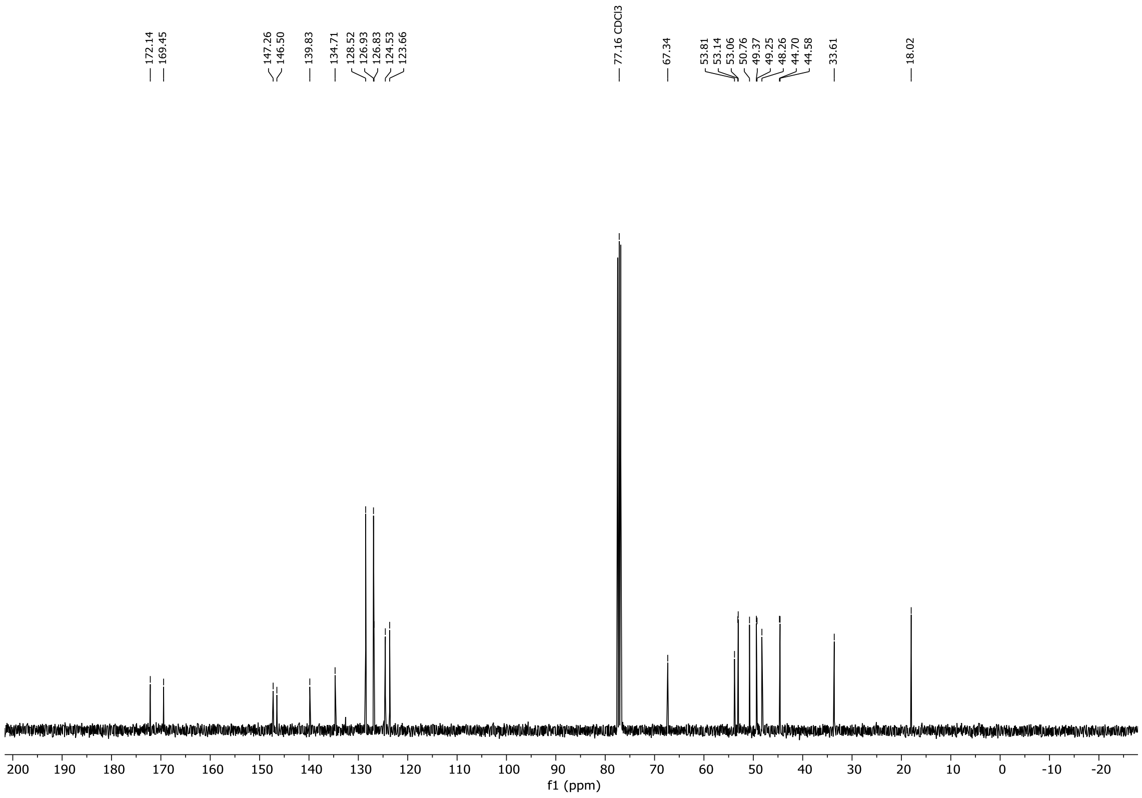

SUPPLEMENTARY REFERENCES

1. Kleinmans, R. *et al.* ortho-Selective Dearomative [2π + 2σ] Photocycloadditions of Bicyclic Aza-Arenes. *J. Am. Chem. Soc.* **145**, 12324–12332 (2023).

2. Luo, C. *et al. Ruthenium-Catalyzed Carbocycle-Selective Hydrogenation of Fused Heteroarenes* (2024).

3. Ma, J. *et al.* Facile access to fused 2D/3D rings via intermolecular cascade dearomative [2 + 2] cycloaddition/rearrangement reactions of quinolines with alkenes. *Nat. Catal.* **5**, 405–413 (2022).

4. Tsukanov, S. V., Marks, L. R. & Comins, D. L. Studies toward the Synthesis of Lepadiformine A. *J. Org. Chem.* **81**, 10433–10443 (2016).

5. Archer, G., Cavalère, P., Médebielle, M. & Merad, J. Photoredox Generation of Isothiouronyl Radical Cations: A New Platform in Covalent Radical Catalysis. *Angew. Chem., Int. Ed.* **61**, e202205596 (2022).

6. Chowdhury, D., Ghosh, S., Reddy, K. P., Yamijala, S. S. & Baidya, M. Cobalt(III)-Catalyzed Free-Amine-Directed Site-Selective Allylation in 2-Aminobiaryls with Vinyl Cyclopropanes. *ACS Catal.* **13**, 12543–12552 (2023).

7. Chen, D.-F., Boyle, B. M., McCarthy, B. G., Lim, C.-H. & Miyake, G. M. Controlling Polymer Composition in Organocatalyzed Photoredox Radical Ring-Opening Polymerization of Vinylcyclopropanes. *J. Am. Chem. Soc.* **141**, 13268–13277 (2019).

8. Chen, D.-F., Bernsten, S. & Miyake, G. M. Organocatalyzed Photoredox Radical Ring-Opening Polymerization of Functionalized Vinylcyclopropanes. *Macromolecules* **53**, 8352–8359 (2020).

9. Moreau, P. & Maffei, M. A stereoselective palladium-catalyzed synthesis of amino alkenyl geminal bisphosphonates. *Tetrahedron Lett.* **45**, 743–746 (2004).

10. Miura, K., Fujisawa, N. & Hosomi, A. Indium(III) chloride-promoted intramolecular addition of allylstannanes to alkynes. *J. Org. Chem.* **69**, 2427–2430 (2004).

11. Collins, K. D. & Glorius, F. A robustness screen for the rapid assessment of chemical reactions. *Nat. Chem.* **5**, 597–601 (2013).

12. Pitzer, L., Schäfers, F. & Glorius, F. Rapid Assessment of the Reaction-Condition-Based Sensitivity of Chemical Transformations. *Angew. Chem., Int. Ed.* **58**, 8572–8576 (2019).

13. Kalt, M.-M. *et al.* Palladium-catalysed synthesis of arylnaphthoquinones as antiprotozoal and antimycobacterial agents. *Eur. J. Med. Chem.* **207**, 112837 (2020).

14. Pavlishchuk, V. V. & Addison, A. W. Conversion constants for redox potentials measured versus different reference electrodes in acetonitrile solutions at 25°C. *Inorganica Chimica Acta* **298**, 97–102 (2000).

15. Joshi-Pangu, A. *et al.* Acridinium-Based Photocatalysts: A Sustainable Option in Photoredox Catalysis. *J. Org. Chem.* **81**, 7244–7249 (2016).

16. Cismesia, M. A. & Yoon, T. P. Characterizing Chain Processes in Visible Light Photoredox Catalysis. *Chem. Sci.* **6**, 5426–5434 (2015).

17. Mondal, S., Debnath, S., Lo, R. & Maity, S. Photoredox Activation of Donor-Acceptor Cyclopropanes: Distonic

Radical Cation Reactivity in [3+2] Cycloaddition Reactions. *Angew. Chem., Int. Ed.* **64**, e202419426 (2025).

18. Zhang, F., Sasmal, H. S., Rana, D. & Glorius, F. Switchable and Chemoselective Arene Hydrogenation for Efficient Late Stage Applications. *J. Am. Chem. Soc.* **146**, 18682–18688 (2024).

19. Ma, J. *et al.* Photochemical intermolecular dearomative cycloaddition of bicyclic azaarenes with alkenes. *Science* **371**, 1338–1345 (2021).

20. Neese, F. Software update: The ORCA program system—Version 5.0. *WIREs Comput. Mol. Sci.* **12** (2022).

21. Hanwell, M. D. *et al.* Avogadro: an advanced semantic chemical editor, visualization, and analysis platform. *J. Cheminform.* **4**, 17 (2012).

22. Greg Landrum *et al. rdkit/rdkit: 2025_09_1 (Q3 2025) beta Release* (Zenodo, 2025).

23. Souza, B. de. GOAT: A Global Optimization Algorithm for Molecules and Atomic Clusters. *Angew. Chem., Int. Ed.* **64**, e202500393 (2025).

24. Bannwarth, C., Ehlert, S. & Grimme, S. GFN2-xTB-An Accurate and Broadly Parametrized Self-Consistent Tight-Binding Quantum Chemical Method with Multipole Electrostatics and Density-Dependent Dispersion Contributions. *J. Chem. Theory Comput.* **15**, 1652–1671 (2019).

25. Chai, J.-D. & Head-Gordon, M. Long-range corrected hybrid density functionals with damped atom-atom dispersion corrections. *Phys. Chem. Chem. Phys.* **10**, 6615–6620 (2008).

26. Grimme, S., Antony, J., Ehrlich, S. & Krieg, H. A consistent and accurate ab initio parametrization of density functional dispersion correction (DFT-D) for the 94 elements H-Pu. *J. Chem. Phys.* **132**, 154104 (2010).

27. Lim, I. S., Schwerdtfeger, P., Metz, B. & Stoll, H. All-electron and relativistic pseudopotential studies for the group 1 element polarizabilities from K to element 119. *J. Chem. Phys.* **122**, 104103 (2005).

28. Weigend, F. & Ahlrichs, R. Balanced basis sets of split valence, triple zeta valence and quadruple zeta valence quality for H to Rn: Design and assessment of accuracy. *Phys. Chem. Chem. Phys.* **7**, 3297–3305 (2005).

29. Stoychev, G. L., Auer, A. A. & Neese, F. Automatic Generation of Auxiliary Basis Sets. *J. Chem. Theory Comput.* **13**, 554–562 (2017).

30. Helmich-Paris, B., Souza, B. de, Neese, F. & Izsák, R. An improved chain of spheres for exchange algorithm. *J. Chem. Phys.* **155**, 104109 (2021).

31. Neese, F. An improvement of the resolution of the identity approximation for the formation of the Coulomb matrix. *J. Comput. Chem.* **24**, 1740–1747 (2003).

32. Barone, V. & Cossi, M. Quantum Calculation of Molecular Energies and Energy Gradients in Solution by a Conductor Solvent Model. *J. Phys. Chem. A* **102**, 1995–2001 (1998).

33. Schlosser, L., Rana, D., Pflüger, P., Katzenburg, F. & Glorius, F. EnTdecker - A Machine Learning-Based Platform for Guiding Substrate Discovery in Energy Transfer Catalysis. *J. Am. Chem. Soc.* **146**, 13266–13275 (2024).

34. Hughes, W., Popescu, M. & Paton, R. *Fundamental Study of Density Functional Theory Applied to Triplet State Reactivity: Introduction of the TRIP50 Dataset* (2025).

35. Popescu, M. V. & Paton, R. S. Dynamic Vertical Triplet Energies: Understanding and Predicting Triplet Energy Transfer. *Chem* **10**, 3428–3443 (2024).

36. Álvarez-Moreno, M. *et al.* Managing the computational chemistry big data problem: the ioChem-BD platform. *J. Chem. Inf. Model.* **55**, 95–103 (2015).

37. Roth, H., Romero, N. & Nicewicz, D. Experimental and Calculated Electrochemical Potentials of Common Organic Molecules for Applications to Single-Electron Redox Chemistry. *Synlett* **27**, 714–723 (2016).

38. Krishnan, R., Binkley, J. S., Seeger, R. & Pople, J. A. Self-consistent molecular orbital methods. XX. A basis set for correlated wave functions. *J. Chem. Phys.* **72**, 650–654 (1980).

39. Zhao, Y. & Truhlar, D. G. The M06 suite of density functionals for main group thermochemistry, thermochemical kinetics, noncovalent interactions, excited states, and transition elements: two new functionals and systematic testing of four M06-class functionals and 12 other functionals. *Theor. Chem. Acc.* **120**, 215–241 (2008).

40. Gaussian 16, Revision B.01, Frisch, M.J., Trucks, G.W., Schlegel, H.B., Scuseria, G.E., Robb, M.A., Cheeseman, J.R. *et al.*

41. M. S. Teynor, N. Wohlgemuth, L. Carlson, J. Huang, S. L. Pugh, B. O. Grant, R. S. Hamilton, R. Carlsen, and D. H. Ess. Milo, Revision 1.0.3.

42. Hunter, J. D. Matplotlib: A 2D Graphics Environment. *Comput. Sci. Eng.* **9**, 90–95 (2007).

43. Pedregosa, F. *et al.* Scikit-learn: Machine Learning in Python. *J. Mach. Learn. Res.* **12**, 2828 (2011).

44. Bruker (2024) APEX6 Version 2024.9-0, SAINT, V8.41, Bruker AXS Inc., Madison, Wisconsin, USA.

45. Krause, L., Herbst-Irmer, R., Sheldrick, G. M. & Stalke, D. Comparison of silver and molybdenum microfocus X-ray sources for single-crystal structure determination. *J. Appl. Crystallogr.* **48**, 3–10 (2015).

46. Sheldrick, G. M. SHELXT - integrated space-group and crystal-structure determination. *Acta Crystallogr.* **71**, 3–8 (2015).

47. Sheldrick, G. M. Crystal structure refinement with SHELXL. *Acta Crystallogr.* **71**, 3–8 (2015).

48. Groom, C. R., Bruno, I. J., Lightfoot, M. P. & Ward, S. C. The Cambridge Structural Database. *Acta Crystallogr.* **72**, 171–179 (2016).

49. Spek, A. L. PLATON SQUEEZE: a tool for the calculation of the disordered solvent contribution to the calculated structure factors. *Acta Crystallogr.* **71**, 9–18 (2015).
