# Supplementary material for: Triple energy transfer-enabled dearomative cycloaddition/rearrangement cascade of bicyclic azaarenes to structurally complex products
Source: Nat Catal. 2026 Jul 9;9(7):750–60. doi: 10.1038/s41929-026-01566-z (PMC13395622; doi:10.1038/s41929-026-01566-z)
Supplement: Supplementary file 2 — CCDC_Nr for all the crystal structures. [file 41929_2026_1566_MOESM2_ESM.pdf]

Von: CCDC Deposit <deposit_reply@ccdc.cam.ac.uk>
An: Constantin G. Daniliuc <cdani_01@uni-muenster.de>

Datum: 13.11.2025 11:11
Betreff: CCDC Depository Request

Typ: Text/Html
Zeichensatz: utf-8

Dies ist eine vereinfachte Darstellung des HTML-Inhalts!

Dear Depositor,

Thank you for depositing your crystal structure(s) via the joint CCDC/FIZ Karlsruhe deposition service.

The data have been assigned the following deposition numbers which can either be quoted as CCDC Numbers or CSD Numbers. A CCDC Number is usually quoted for an organic or metal-organic structure, whereas a CSD Number is usually quoted for an inorganic structure.

CCDC XXXXXXX-YYYYYYY (generally used for organic and metal-organic structures)

CSD XXXXXXX-YYYYYYY (generally used for inorganic structures)

Deposition Number 2502652-2502659

Summary of Data - Deposition Number 2502652

Compound Name:
Data Block Name: data_glo10899_4a
Unit Cell Parameters: a 10.8112(6) b 17.4261(9) c 11.0987(7) P21/n
----

Summary of Data - Deposition Number 2502653

Compound Name:
Data Block Name: data_glo10900_3a
Unit Cell Parameters: a 14.4978(7) b 7.5996(3) c 32.7748(13) P21/c
----

Summary of Data - Deposition Number 2502654

Compound Name:
Data Block Name: data_glo10802_3f
Unit Cell Parameters: a 45.5403(17) b 8.0714(3) c 13.2845(4) C2/c
----

Summary of Data - Deposition Number 2502655

Compound Name:
Data Block Name: data_glo10903_5ad
Unit Cell Parameters: a 8.6806(5) b 9.8665(5) c 13.2035(6) P-1

Summary of Data - Deposition Number 2502656

Compound Name:
Data Block Name: data_glo10853_5ag
Unit Cell Parameters: a 13.2595(19) b 13.282(2) c 14.433(2) P-1

Summary of Data - Deposition Number 2502657

Compound Name:
Data Block Name: data_glo10775_5h

Unit Cell Parameters: a 20.2516(9) b 13.6623(6) c 18.2134(8) C2/c
----

Summary of Data - Deposition Number 2502658

Compound Name:
Data Block Name: data_glo10923_3b
Unit Cell Parameters: a 25.2596(6) b 6.9396(2) c 11.6918(3) C2

Summary of Data - Deposition Number 2502659

Compound Name:
Data Block Name: data_glo10846_5ao
Unit Cell Parameters: a 8.8611(7) b 14.1211(9) c 17.7054(13) P212121

After publication your data will be made available through our joint Access Structures service. In addition, organic and metal-organic experimental structures will be curated into the [a1]Cambridge Structural Database and inorganic experimental structures will be curated into the [a2]Inorganic Crystal Structure Database.

If you selected “Publish in a Database” your data will be immediately published through our joint Access Structures service.

Please note, if any of these structures are not published within one year from today and we cannot contact you to discuss the matter, then we may publish the data directly through the CSD as a *CSD Communication* or the ICSD as an *ICSD Communication*.

If we have any queries relating to the data then we may contact you later.

Kind Regards,

The CCDC and FIZ Karlsruhe Deposition Teams

Email: [a3]deposit@ccdc.cam.ac.uk
The Cambridge Crystallographic Data Centre
[a4]https://www.ccdc.cam.ac.uk
For more information about CSD Communications see:
[a5]https://www.ccdc.cam.ac.uk/Community/Depositastructure/CSDCommunications/
FIZ Karlsruhe
[a6]www.fiz-karlsruhe.de

The CCDC and FIZ Karlsruhe are delighted to be working together on shared deposition and access services for crystallographic data across all domains of chemistry

More details can be found in our press release: [a7]https://www.ccdc.cam.ac.uk/News/List/2018-07-new-joint-services/

[a1] https://www.ccdc.cam.ac.uk/Solutions/CSDSystem/Pages/CSD.aspx
[a2] http://www2.fiz-karlsruhe.de/icsd_home.html
[a3] mailto:deposit@ccdc.cam.ac.uk
[a4] http://www.ccdc.cam.ac.uk/
[a5] https://www.ccdc.cam.ac.uk/Community/Depositastructure/CSDCommunications/
[a6] http://www.fiz-karlsruhe.de/
[a7] https://www.ccdc.cam.ac.uk/News/List/2018-07-new-joint-services/

perMail – © 2001–2020 Rainer Perske + Universität Münster, Germany
